# Anti-Markovnikov alkene hydroalkylation via iron photocatalysis

**DOI:** 10.1038/s41929-026-01600-0

**Published:** 2026-09-15

**Authors:** Shih-Chieh Kao, Kang-Jie Bian, Jess C. Tang, Shijin Yu, Yongchen Wang, Ying Chen, Xiaowei Chen, Julian G. West

**Affiliations:** Department of Chemistry, Biosciences Research Collaborative, Rice University, Houston, TX USA

**Keywords:** Photocatalysis, Homogeneous catalysis, Photocatalysis, Synthetic chemistry methodology

## Abstract

Hydroalkylation provides a direct approach to C(*sp*^3^)–C(*sp*^3^) bond formation from abundant alkenes, yet general methods for linear-selective hydroalkylation remain limited, particularly for simple alkyl groups such as methyl and ethyl. Here we introduce traceless radical polarity reversal, in which a removable electron-withdrawing group promotes radical alkene addition and is subsequently deleted in situ under the reaction conditions. Using a visible-light-promoted iron-and-thiol dual-catalytic system, this strategy enables hydroalkylation reactions that were previously difficult to access, including hydromethylation, hydroethylation and hydrocyclobutylation, with high linear selectivity from simple malonic acids. The method also provides direct access to *gem*-monofluoroalkyl and *gem*-difluoroalkyl motifs from fluorinated malonic acids and enables efficient synthesis of bioactive targets, including a GPR119 agonist. Preliminary mechanistic studies support a dual-catalytic radical pathway involving visible-light-induced homolysis and hydrogen-atom transfer processes.

**Figure:**
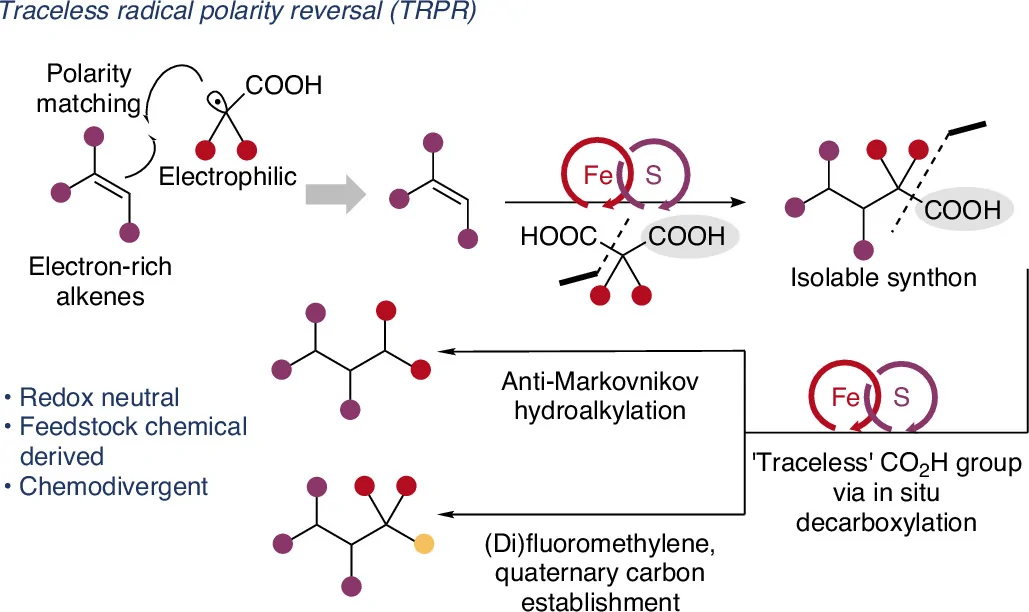

## Abstract (web summary)

Hydroalkylation, the addition of a C–H across an alkene bond, is an ideal method for making C(*sp*^3^)–C(*sp*^3^) bonds; however, linear selectivity is challenging. Here the authors report on achieving this with malonic acids as the alkyl donor reagents using iron photocatalysis.

## Main

Forging C(*sp*^3^)–C(*sp*^3^) bonds is of fundamental importance in organic chemistry and is key to achieving the synthesis of natural products and pharmaceuticals in a convergent way^1,2^. Similarly, C(*sp*^3^)–C(*sp*^3^) coupling reactions are also of great interest to the petrochemical industry, where controllably extending hydrocarbons with low carbon numbers to higher carbon numbers could contribute to the production of diesel and jet fuel^3–5^. Further, the landscape of medicinal chemistry has been transformed by the strategic incorporation of (di)fluoromethylene units into aliphatic chains. Serving as adept bioisosteres in late-stage functionalization^6^, these units have influenced the modulation of bioactive molecules’ biological and physiological activities. This innovative approach has led to substantial enhancements in therapeutic profiles, with marked improvements in bioactivity^7^. In tandem with this fluorine-based strategy, a conceptual advance has occurred in drug discovery towards the enrichment of small molecules with C(*sp*^3^)-hybridized carbon atoms, allowing researchers to unlock the ability to target elusive therapeutic proteins. The introduction of quaternary carbon motifs has paved the way for exploring these uncharted chemical territories, resulting in bioactive compounds with heightened three dimensionality. These architecturally intricate structures, characterized by their restricted conformational landscape, have demonstrated improvements in potency, selectivity and metabolic stability, thus propelling the field of drug development into realms of possibility^8^.

**Fig. 1:**
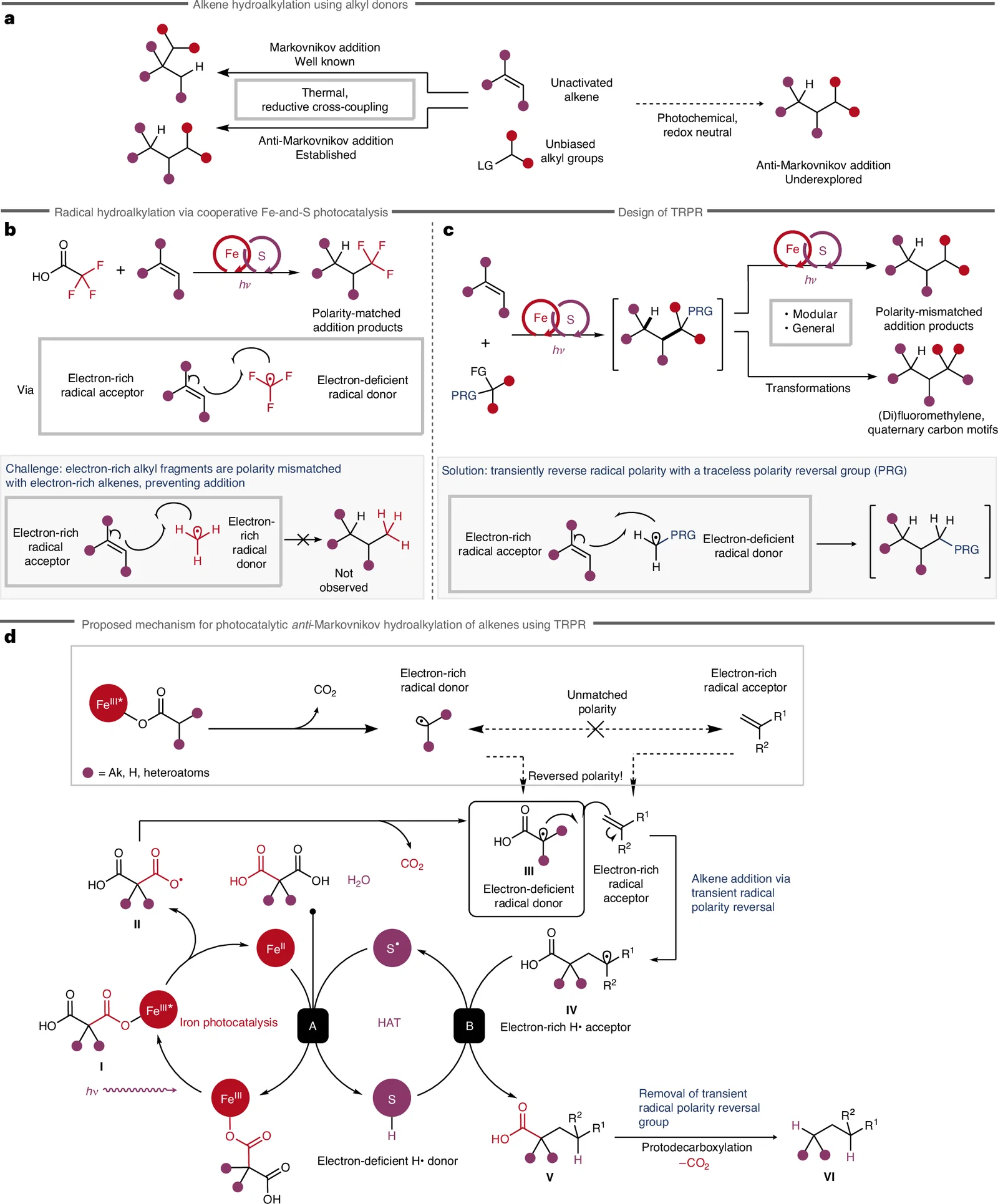
Reaction design and development of alkene anti-Markovnikov hydroalkylation. **a**, Current strategies for alkene hydroalkylation and limitations in linear-selective C(*sp*^3^)–C(*sp*^3^) bond formation. **b**, Radical polarity matching governs selective alkene hydrofunctionalization. **c**, Concept of TRPR for anti-Markovnikov hydroalkylation. **d**, Proposed dual-catalytic mechanism for iron-and-thiol-promoted hydroalkylation via TRPR. LG, leaving group; FG, functional group; *h*, Planck’s constant; *ν*, frequency.

Thus, the development of new methods to convergently construct C(*sp*^3^)–C(*sp*^3^) bonds with linear selectivity is in high demand. Amid a broad spectrum of methodologies employed for the synthesis of C(*sp*^3^)–C(*sp*^3^) bonds, hydroalkylation of unactivated alkenes has been recognized as a direct and atom-economical strategy^9–12^. Recent progress in the catalytic hydroalkylation of unactivated alkenes has largely been achieved by Dao et al., Green et al., Chen et al., Wang et al. and Wang et al. under reductive cross-coupling conditions and often employing metal-catalysed hydrogen-atom transfer (MHAT) pathways where transient alkyl radicals are generated from unactivated olefins under thermal activation via simple metal hydride insertion events, representing an effective approach to chemoselectively prepare selective branched (Markovnikov) hydroalkylation products^13–17^ (Fig. 1a). In parallel, linear (anti-Markovnikov) selective alkene hydroalkylation through organometallic metal hydride mechanisms has been demonstrated by Wang et al., Lu et al., Zhou et al. and Tong et al. as a potent approach for facilitating C(*sp*^3^)–C(*sp*^3^) couplings^18–21^. However, the broader adoption of these methods in medicinal chemistry, in which the rapid diversification of molecules, three dimensionality and scaffold hopping are key for lead compound development, remains limited by the need for extensive ligand optimization to achieve high regio- and chemoselectivity, as well as by the use of the premodification of alkylation partners with non-atom-economical and expensive activating reagents or electrochemical equipment^22–25^ (Fig. 1a).

Moreover, organometallic hydroalkylation mechanisms themselves introduce additional practical challenges. The organometallic intermediates generated following metal hydride insertion into unactivated alkenes often exhibit limited reactivity towards electrophilic coupling partners, frequently necessitating the use of excess alkene substrate. In many cases, directing auxiliaries are also required, adding extra installation and removal steps to the synthetic sequence. Furthermore, the generation of the active metal hydride species typically relies on stoichiometric quantities of expensive hydride donors, such as silanes. Taken together, these limitations highlight the need for a general hydroalkylation strategy that employs readily available alkylation partners while avoiding the constraints associated with conventional organometallic approaches. Such methods remain highly desirable across synthetic and medicinal chemistry.

A complementary mechanistic approach to MHAT and organometallic alkene hydroalkylation is alkyl radical addition to the alkene starting material, generating a new radical that can then be quenched via a hydrogen-atom transfer (HAT) step to furnish the hydroalkylation product. An advantage of this approach is its inherent linear selectivity, with radical addition often proceeding to generate the more substituted successor radical. We have recently shown that this strategy is amenable to alkene hydrofluoroalkylation (Fig. 1b), allowing for the addition of electron-deficient fluoroalkyl radicals derived from carboxylic acids to unactivated alkenes with high efficiency and linear selectivity using synergistic iron-and-thiol co-catalysis^26^. This reaction proceeds under mild conditions and engages a wide range of fluoroalkyl carboxylic acids; however, it is unable to engage electron-rich alkyl carboxylic acids in hydroalkylation, with these reactions simply resulting in decarboxylative protonation of the acids, with the alkene starting material untouched.

The reason for this dearth of radical hydroalkylation reactions can be traced to radical polarity matching, a fundamental property of radical alkene addition reactions^27–32^. Indeed, the addition of electron-deficient electrophilic radicals (for example, •CF_3_) to unactivated, electron-rich alkenes (for example, 1-hexene) or electron-rich nucleophilic radicals (for example, •CH_3_) to electron-deficient alkenes (for example, methyl acrylate) proceeds rapidly and efficiently due to the polarity matching of both partners. By contrast, the addition of electron-deficient radicals to electron-deficient alkenes or electron-rich radicals to unactivated, electron-rich alkenes is slow and inefficient, often competing with more-rapid side reactions such as HAT. Thus, hydroalkylation of unactivated alkenes by simple alkyl radicals is prevented due to a radical polarity mismatch, a fundamental property inherent to these specific coupling partners (Fig. 1b). State-of-the-art work on the radical hydroalkylation of unactivated alkenes was developed with the use of alkyl coupling partners bearing radical stabilizing groups (electron-withdrawing groups or heteroatoms) to match the polarity with unactivated alkenes^33–43^. While the matching-polarity requirement for the addition step was achieved to obtain the desired hydroalkylation products in these cases, the additional synthetic steps needed to install and transform these electron-withdrawing groups have restrained the generality of these methods. Overall, radical anti-Markovnikov hydroalkylation of unactivated alkenes does not yet have a general solution, particularly one allowing controllable installation of simple alkyl groups or functionalized large fragments for further diversifications or the efficient preparation of valuable molecules, making this reaction class desirable.

To overcome this long-standing problem, we introduce a mechanistic strategy termed traceless radical polarity reversal (TRPR; Fig. 1c). Unlike classical umpolung approaches, which permanently alter substrate polarity through functional-group interconversion, or polarity-reversal catalysis strategies that rely on catalyst-controlled modulation of reactivity, TRPR temporarily embeds a removable polarity-reversal group directly within the radical precursor itself. In this scheme, the electron-rich alkyl radical is temporarily rendered electron deficient via a removable polarity-reversal functional group, enabling streamlined addition to unactivated alkenes. Following polarity-matched radical addition, the polarity-reversal group is removed in situ, allowing for hitherto-inaccessible reactions such as radical hydroethylation and hydromethylation of unactivated alkenes to be achieved, and providing an approach for the facile construction of C(*sp*^3^)–C(*sp*^3^) bond-rich scaffolds. Ideally, the alkyl radical precursor would be a simple and abundant starting material and could be activated using simple and earth-abundant catalysts, allowing this reaction to be performed cheaply and sustainably. In a parallel investigation, we wondered if the polarity-reversal group could be maintained and used to diversify the hydroalkylation product, enabling the extension of this approach to traditionally inaccessible molecular scaffolds.

To realize this approach, we envisioned malonic acids as ideal TRPR precursors for catalytic alkene hydroalkylation under cooperative iron-and-thiol co-catalysis, proceeding via two catalytic phases: alkene hydrofunctionalization to produce a carboxylic acid intermediate, followed by removal of the polarity-reversal carboxylic acid group via decarboxylative protonation. Within our envisioned framework (Fig. 1d), the first phase begins with homolytic cleavage of the O–Fe bond of the alkyl malonate intermediate **I**, which produces the carboxyl radical **II** and a lower-valent iron species through visible-light-induced homolysis (VLIH). Following this homolysis, the extrusion of CO_2_ from carboxyl radical **II** allows access to α-carboxyalkyl radical species **III**, which can engage in polarity-matched radical addition onto the electron-rich alkene, providing nucleophilic, traceless carbon-centred radical intermediate **IV**, which produces carboxylic acid intermediate **V** via polarity-matched thiol HAT^26^ and generates an oxidizing thiyl radical in a parallel manifold (step B). This thiyl radical can then reoxidize the lower-valent iron intermediate while receiving a proton from another equivalent of acid substrate or H_2_O, closing both catalytic cycles. Next, the adduct **V** engages in the same catalytic phase of the reaction to provide the desired hydroalkylation product **VI** via protodecarboxylation, as we previously reported in the literature^44^. Additionally, if the catalytic cycle can be controllably stopped after the hydrocarboxylalkylation step, various value-added one-carbon motifs can be accessed through the derivatization of intermediate **V** by using other decarboxylative functionalization reactions, allowing malonic acids to serve as versatile one-carbon linkers for complex synthesis.

Herein we report a photocatalytic anti-Markovnikov hydroalkylation of alkenes enabled by synergistic iron photocatalysis and thiol HAT catalysis (Fig. 1a). Leveraging TRPR, alkyl malonic acids function as electron-rich alkyl equivalents that overcome polarity-mismatched radical addition, enabling anti-Markovnikov hydroalkylation reactions including hydroethylation, hydromethylation and hydrocycloalkylation without the limitations commonly associated with MHAT or organometallic metal hydride pathways. Furthermore, we showcase the impact of our method on streamlining bioactive molecule synthesis and alternative disconnections for producing a GPR119 agonist^45^. Additionally, we identify conditions that allow cycloalkane-1,1-dicarboxylic acids and mono- or difluoromalonic acids to serve as alternative quaternary C-1, monofluoromethylene or difluoromethylene motifs. They facilitate straightforward, robust and modular preparations of C(*sp*^3^)-rich scaffolds^8^, such as monofluoroalkyl, *gem*-difluoroalkyl and quaternary carbons, through the incorporation of commercial alkene C(*sp*^2^) building blocks via alkene hydroalkyl carboxylation. Further, this design proceeds using cheap and sustainable earth-abundant element catalysts while avoiding the need for stoichiometric activating reagents and generating carbon dioxide as a benign by-product, providing a direct and redox-neutral approach to afford hydroalkylation products.

## Results

### Establishing conditions for anti-Markovnikov hydroalkylation

To develop this linear-selective hydroalkylation, we investigated a series of conditions using pent-4-en-1-yl benzoate as the model substrate, methyl malonic acid (**MA-1**) as an ethyl (Et) source and earth-abundant iron salts and organic thiols as catalysts under light irradiation at 40 °C (Table 1). We found that iron nitrate in combination with bis(2,4,6-triisopropylphenyl) disulfide (TRIP disulfide) can facilitate this redox-neutral hydroethylation process efficiently, giving the desired product (**2**) in 71% yield with only traces remaining of the carboxylic acid adduct (**2′**), confirming our hypothesis that TRPR can enable efficient polarity-mismatched alkene hydroalkylation (Table 1, entry 1). We next sought to investigate key reaction parameters of this protocol by performing control experiments, revealing that iron, disulfide and light irradiation are all required, with no conversion observed in the absence of any one of them, supporting our mechanistic proposal (Table 1, entries 2–3). Furthermore, decreasing the intensity of irradiation showed a light dependency of the conversion of acid intermediate **2′** to **2**, an observation in line with our previous work on photocatalytic hydrodecarboxylation^44^ (Table 1, entry 4). The decarboxylation of malonic acid (**MA-1**) proceeds in the absence of the base, yielding the acid intermediate **2′** in a high yield while only trace amounts of product **2** are observed (Table 1, entry 5). We hypothesize that malonate coordinating to the iron centre to form iron–malonate species is insensitive to the catalytic loadings of the base due to the easily dissociable acidic proton of malonic acid (p*K*_a__1_ ≈ 2.83, where *K*_a_ is the acid dissociation constant), thereby being able to provide an α-carboxyalkyl radical for alkene addition through the iron-mediated VLIH decarboxylation of malonic acid. However, the less acidic proton of the carboxylic acid intermediate **2′** (p*K*_a_ ≈ 4.76) suggests that the formation of iron carboxylate species, through coordination of the carboxylate intermediate with the iron centre for hydrodecarboxylation, might require base promotion. On the other hand, possibly due to competitive deprotonation of the thiol, higher base loadings might impede the subsequent HAT process, resulting in lower yields of **2** and **2′** (Table 1, entry 6). Adequate reaction time and the appropriate loading of malonic acid (**MA-1**) are also crucial to achieve higher yields of product **2** (Table 1, entries 7 and 8). Screening different iron salts demonstrated that iron nitrate is the most efficient catalyst (Table 1, entries 9–14). Evaluating solvents indicated that acetonitrile is the most effective solvent for this reaction, with others resulting in diminished or absent reactivity (Table 1, entries 15–19); additionally, insufficient water in the co-solvent system hindered the reaction, resulting in full recovery of starting material (Table 1, entry 20).

**Table 1:** Investigation of reaction parameters of alkene anti*-*Markovnikov hydroalkylation


Entry	Deviation from the standard conditions	Yield of 2 (%)^a^	Yield of 2′ (%)^a^
1	None	71	Trace
2	No iron salt, no disulfide	0	0
3	No light, no light at 40 °C	0	0
4	25% intensity	52	16
5	No base	Trace	86
6	Na_2_CO_3_ (30 mol%)	37	Trace
7	24 h	40	40
8	MA-1 (1.0–2.0 equiv.)	31–52	Trace
9	Fe(OAc)_2_	35	Trace
10	Fe_2_(SO_4_)_3_ · 5H_2_O	62	Trace
11	Fe(OTf)_3_	56	Trace
12	FeCl_3_ · 6H_2_O	70	Trace
13	Fe_2_(C_2_O_4_)_3_ · 6H_2_O	Trace	Trace
14	Fe(acac)_3_	7	Trace
15	DCE/H_2_O	0	0
16	H_2_O	0	0
17	Acetone/H_2_O	63	Trace
18	MeOH/H_2_O	51	13
19	THF/H_2_O	26	Trace
20	MeCN/H_2_O = 9:1	0	0

Reaction conditions were as follows: alkene (0.1 mmol, 1.0 equiv.), **MA-1** (4.0 equiv.), Fe(NO_3_)_3_ ‧ 9H_2_O (15 mol%), TRIP disulfide (25 mol%), Na_2_CO_3_ (10 mol%) and solvent (0.1 M), 48 h, 40 °C, N_2_, 390 nm Kessil blue LED (52 W).

^a^The ^1^H NMR yield is determined by using CH_2_Br_2_ as an internal standard. TRIPSH, 2,4,6-triisopropylbenzenethiol; (TRIPS)_2_, bis(2,4,6-triisopropylphenyl)disulfide; Ac, acetyl; Bz, benzoyl; acac, acetylacetonate; DCE, dichloroethane.

### Scope and diversification of *anti*-Markovnikov hydroalkylation

With these conditions in hand, we next explored the substrate scope of our hydroethylation protocol (Table 2). The 4-phenyl-1-butene proceeded smoothly in our protocol to obtain hydroethylation product **1** in a good yield. Aryl-substituted alkenes demonstrated a slight dependence on the electronic properties of the aryl substituent in our protocol, delivering the corresponding hydroethylation products **2**–**4** in moderate to good yields, with the remaining mass balances being unreacted carboxylic acid adducts and starting materials. Phthalimides and sulfonamides bearing acidic N–H protons (**5** and **6**), oxidatively labile sulfides and alcohols (**7** and **10**) and a coordinating tetrahydropyran (**8**) and ester (**9**) are tolerated, giving moderate to high yields of hydroethylation products. A 1,1-disubstituted alkene bearing an alcohol underwent hydroalkylation effectively to produce the corresponding product **10**, possibly due to the formation of a more stable tertiary alkyl radical intermediate in the reaction. Heterocyclic substrates containing both nitrogen and oxygen produced hydroethylated products **11**–**15** in moderate yields, supporting the application of this method in medicinal chemistry. Notably, boronic esters, valuable building blocks in organic synthesis, are compatible with the reaction to give the desired product **16** in 59% yield. In addition to highly functionalized substrates, our protocol is able to linearly extend long-chain aliphatic alkenes to the corresponding aliphatic hydrocarbons **17**–**20**, including direct conversion of 1-nonene to *n*-undecane **20**. Electron-withdrawing heteroatom-substituted olefins, such as vinyl sulfone and vinyl halides (X = Cl, F), could be ethylated to yield the desired products **21**–**23**, providing a method to access monohalomethylene motifs. Notably, our strategy effectively trapped α-oxy and α-amino radicals using TRIP thiol (bond dissociation energy of S–H, BDE_S–H_ ≈ 87 kcal mol^−1^) with moderate exothermicity^46^, where the resulting alkane products have lower bond dissociation energies (BDE_α-C–H_ ≈ 90 kcal mol^−1^) of their hydrogen atoms adjacent to oxygen and nitrogen atoms due to the radical stabilizing effect of these heteroatoms, thereby providing direct access to such heteroatom-functionalized hydroethylation products, **24** and **25**. However, dimethylphenylvinylsilane and isopropenyl pinacol boronate presented limitations, resulting in the formation of unidentified high-polarity mixtures, possibly due to the decomposition of the starting materials. To testing our method in late-stage functionalization contexts, we investigated a range of alkenes obtained from readily available active pharmaceutical ingredients and natural products. Vinclozolin, a common dicarboximide fungicide, and eugenol, a component of natural oils, can be directly hydroethylated at their innate olefinic moeities, delivering products **26** and **27** in moderate yields, as can non-steroidal anti-inflammatory drug derivatives of loxoprofen and naproxen (**28** and **30**). Importantly, the remaining mass balance for these reactions was unreacted starting materials, suggesting that the low yields were due to reduced reactivity and not intrinsic incompatibility. A derivative of common herbicide 2,4-dichlorophenoxyacetic acid (**29**) demonstrated good efficiency in the reaction. Interestingly, terpenoid natural product (–)-carvone led to double hydroethylation, forming highly branched product **31**. By contrast, product **32** can be afforded from nootkatone without affecting its intrinsic α,β-unsaturated enone, suggesting sterics to be an important parameter. Gibberellic acid derivative (**33**) underwent the reaction specifically at the geminal alkene with excellent diastereoselectivity, albeit in a moderate yield.

**Table 2:**
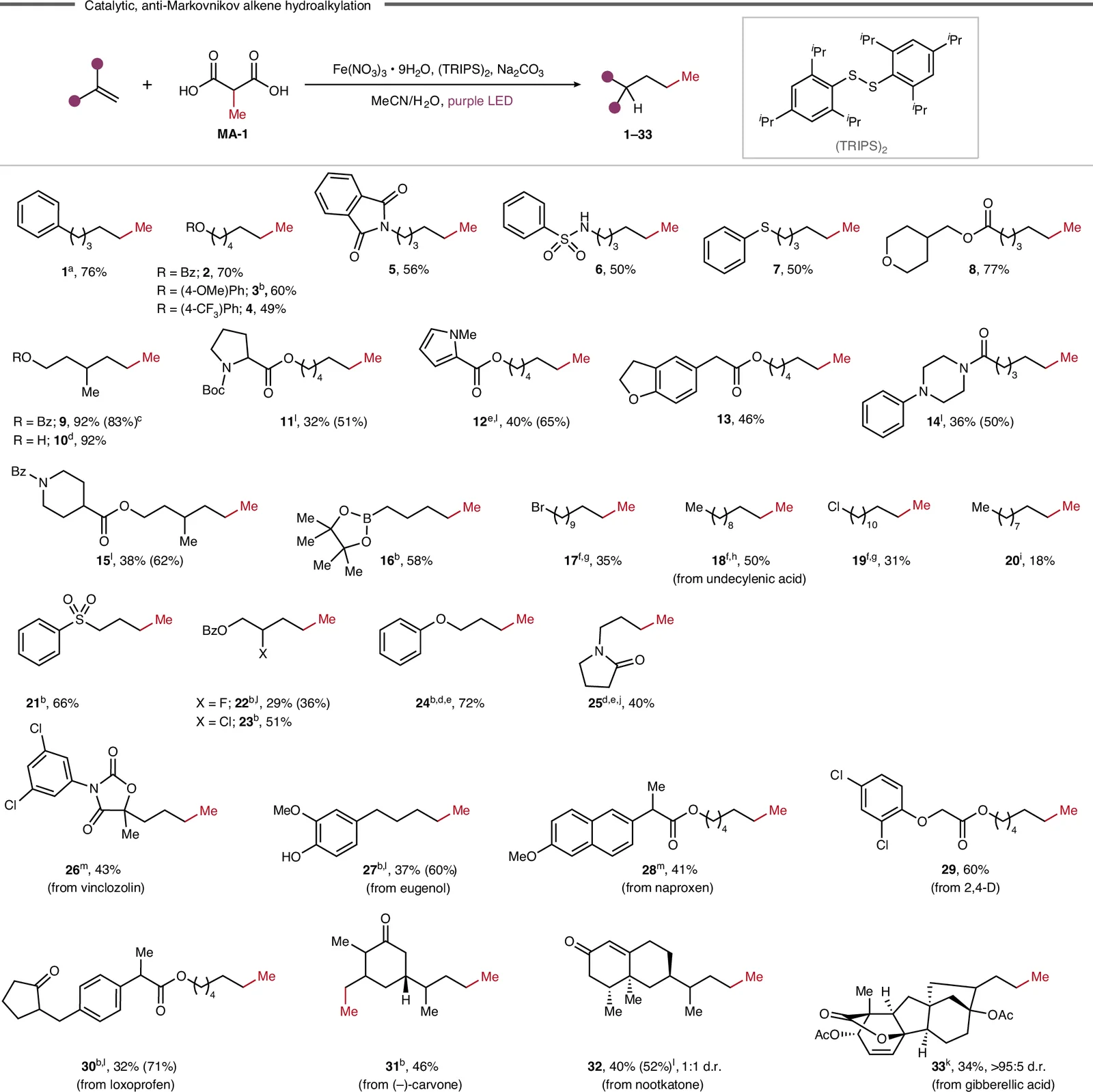
Scope of photocatalytic anti-Markovnikov hydroethylation of alkenes with malonic acids

Standard conditions were as follows: alkene (0.1 mmol, 1.0 equiv.), **MA-1** (4.0 equiv.), Fe(NO_3_)_3_ ‧ 9H_2_O (15 mol%), TRIP disulfide (25 mol%), Na_2_CO_3_ (10 mol%) and MeCN/H_2_O = 1:1 (0.1 M), 48 h, N_2_, 40 °C, 390 nm Kessil purple LED (52 W). 2,4-D, 2,4-dichlorophenoxyacetic acid.

^a^The ^1^H NMR yield is determined by using 1,3,5-trimethoxybenzene as an internal standard.

^b^TRIPSH (10 mol%) was used.

^c^Reaction was performed on 0.5 g scale.

^d^CH_2_Br_2_ was added as an internal standard (NMR yield).

^e^**MA-1** (3.0 equiv.) was used.

^f^GC yield by using cyclododecane as as internal standard.

^g^0.05 M, MeCN/H_2_O = 1:1.

^h^**MA-1** (3.0 equiv.) for the first 24 h, and then another 3.0 equiv. of **MA-1** was added to react for another 24 h.

^i^Fe(NO_3_)_3_ ‧ 9H_2_O (25 mol%), **MA-1** (6.0 equiv.).

^j^Fe(OAc)_2_ (5 mol%), TRIPSH (20 mol%).

^k^Solvent system (MeCN/H_2_O/PhCF_3_ = 0.5 ml:0.5 ml:0.05 ml) was used.

^l^Overall yields reported in parenthesis; product yields were optimized through a telescoped two-step sequence; procedures are reported in Supplementary Table 12.

^m^Solvent system (MeCN/H_2_O/EtOAc = 0.5 ml:0.5 ml:0.05 ml) was used.

**Fig. 2:**
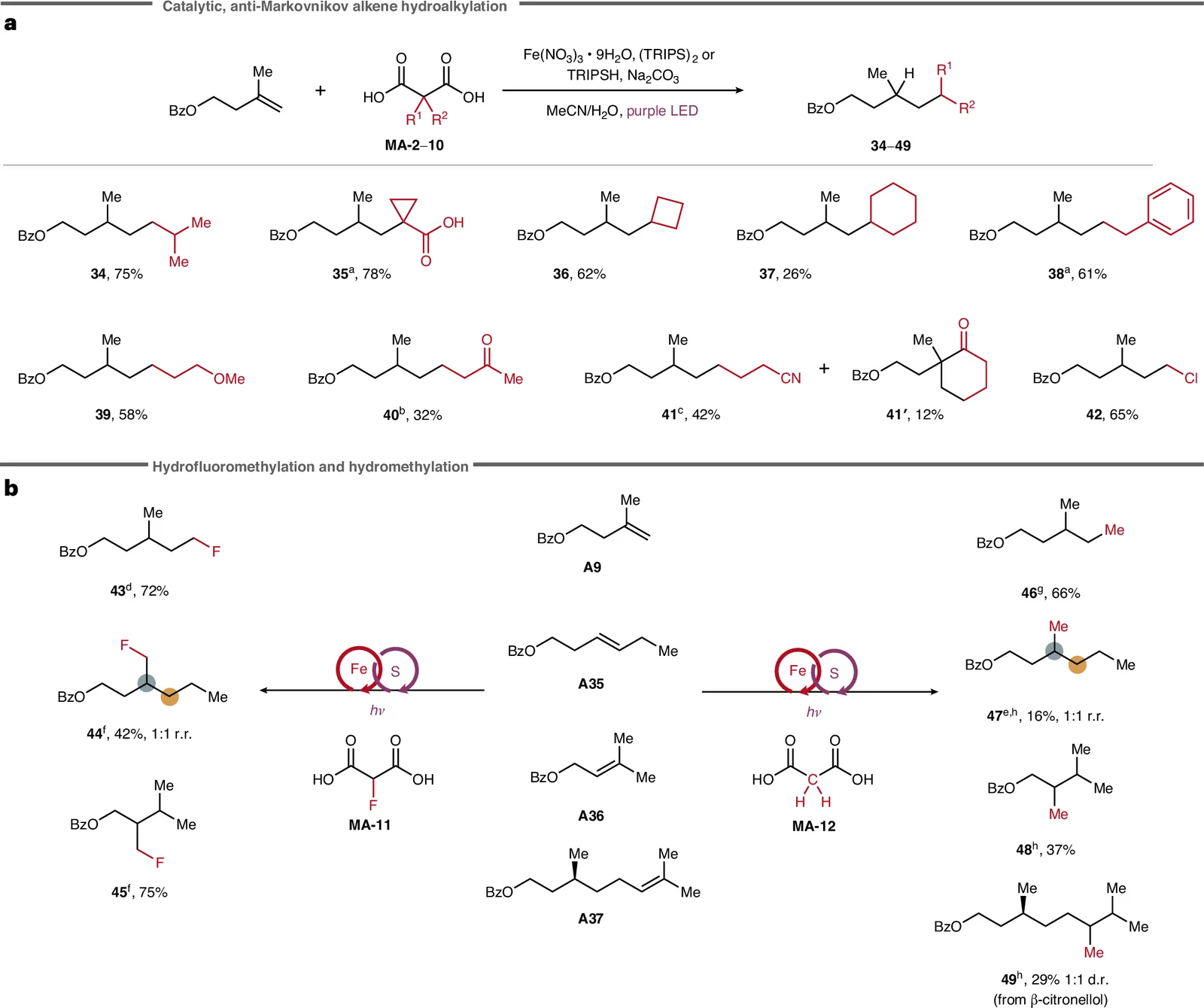
Substrate scope with various malonic acids for hydroalkylating alkenes. **a**, Hydroalkylation of alkenes. **b**, Hydrofluoromethylation and hydromethylation of alkenes. Standard conditions were as follows: alkene (0.1 mmol, 1.0 equiv.), malonic acids (4.0 equiv.), Fe(NO_3_)_3_ ‧ 9H_2_O (15 mol%), TRIP disulfide (25 mol%), Na_2_CO_3_ (10 mol%) and MeCN/H_2_O = 1:1 (0.1 M), 48 h, N_2_, 40 °C, 390 nm Kessil purple LED (52 W). ^a^TRIPSH (10 mol%) was used. ^b^Solvent system (MeCN/H_2_O/DCE = 0.5 ml:0.5 ml:0.05 ml) was used. ^c^0.2 M, (MeCN/H_2_O = 1:1). ^d^Fe(NO_3_)_3_ ‧ 9H_2_O (10 mol%), **MA-11** (3.0 equiv.), TRIP disulfide (20 mol%), MeCN/H_2_O = 9:1 (0.1 M), 40 °C for the first 24 h, then another 3.0 equiv. of **MA-11** was added to react for another 24 h. ^e^CH_2_Br_2_ was added as an internal standard (NMR yield). ^f^The Supplementary Information contains a detailed synthetic procedure for the hydrofluoromethylation of **A35** and **A36**. ^g^The Supplementary Information contains a detailed synthetic procedure for the hydromethylation of **A9**. ^h^The Supplementary information contains a detailed synthetic procedure for the hydromethylation of **A35**, **A36** and **A37**. r.r., regioisomeric ratio.

Having established the generality of alkene components, we next explored the scope of malonic acids using alkene (**A9**) as a starting material (Fig. 2a). Isopropyl, benzyl and methoxyethyl malonic acids reacted to provide corresponding linear alkylated products **34**, **38** and **39**. The prevalent 1,1-disubstituted cyclopropane unit found in therapeutic agents can be obtained using our protocol via decarboxylative hydroalkylation^47^; however, the intermediate cyclopropane carboxylic acid product **35** formed from cyclopropane-1,1-dicarboxylic acid cannot be further hydrodecarboxylated under our conditions, potentially owing to the iron-mediated direct photodecarboxylation being unable to overcome a high energy barrier of generating *sp*^2^-like and unstablized (no delocalizing substituents, for example, –COOH, attached to the radical site) free cyclopropyl radical (*exo* orbitals of *sp*^2.28^; angle 116°)^48^ from cyclopropane carboxylic acid without the incorporation of NHP esters as activating groups^49^. By contrast, cyclobutane-1,1-dicarboxylic acid proceeded smoothly to the desired hydrocyclobutanation product **36** in a good yield. Cyclohexane-1,1-dicarboxylic acid exhibits moderate reactivity to form product **37** with remaining, mostly unreacted alkene, potentially due to the modestly nucleophilic character and steric hindrance of the radical intermediate^28^. Methyl ketone product **40** was also accessible from the corresponding substituted malonic acid. Intriguingly, cyanoethyl malonic acid produces both hydroalkylation product **41** and annulation product **41′**, presumably due to radical cyclization following alkene addition. Traditionally challenging hydromonohalomethylation of unactivated alkenes can be tackled through our protocol^26,50^, furnishing a hydrochloromethylation product (**42**; Fig. 2a) and hydrofluoromethylation products (**43**–**45**; Fig. 2b) using the corresponding chloro- and fluoromalonic acids. In the early stage of reaction optimization on the hydrofluoromethylation of **A9**, we observed that the protodecarboxylation of α-fluorocarboxylic acid **MA-11** intermediate **62** was totally inhibited under both room temperature (r.t.) and heated conditions when a 1:1 MeCN/H_2_O solvent system was adopted, affording **62** in a high yield (Table 3a). To acquire the hydrofluoromethylation product, we later discovered that the desired tandem process can be achieved via overnight reaction with a 9:1 MeCN/H_2_O solvent, delivering desired product **43**, albeit in a low yield (~45%) as measured using NMR. Further analysis of this reaction revealed a substantial amount of unreacted alkene starting material, leading us to pursue a second addition of α-fluoromalonic acid **MA-11** in the middle of the reaction progress. This second addition allowed unreacted alkene to be further consumed, elevating the overall product yields of hydrofluoromethylation (**43** in 72% yield; Fig. 2b). Additionally, we present an approach to access olefin anti-Markovnikov hydromethylation with our strategy, delivering hydromethylation products (**46**–**49**; Fig. 2b) by using malonic acid^44^. While possible, hydromethylation presents a particular challenge because it requires a telescoped two-step sequence in which the reaction conditions must be altered mid-process to obtain the desired product. This complication arises from the same issue encountered in hydrofluoromethylation: the alkene addition step proceeds readily, but the protodecarboxylation step is inhibited in a 1:1 MeCN/H_2_O solvent system. By contrast, the use of a 9:1 MeCN/H_2_O system is ineffective for carboxylalkyl radical addition to the alkene using non-fluorinated malonic acids (Table 1, entry 20); however, this system is efficient for the protodecarboxylation of acid intermediates. Overall, these results provided a pathway to increase the three dimensionality of abundant alkene molecules and allowed the preparation of a variety of traditionally inaccessible chemical spaces^8^. While they are general, electron-rich α-methoxy or α-acetamido malonic acids are ineffective for hydroalkylation, potentially due to a mismatch in polarity with unactivated alkenes^28^, and the incorporation of tetra-substituted alkenes, for example, 2,3-dimethyl-2-butene, was also unsuccessful, possibly owing to their innately large steric hindrance.

**Table 3:**
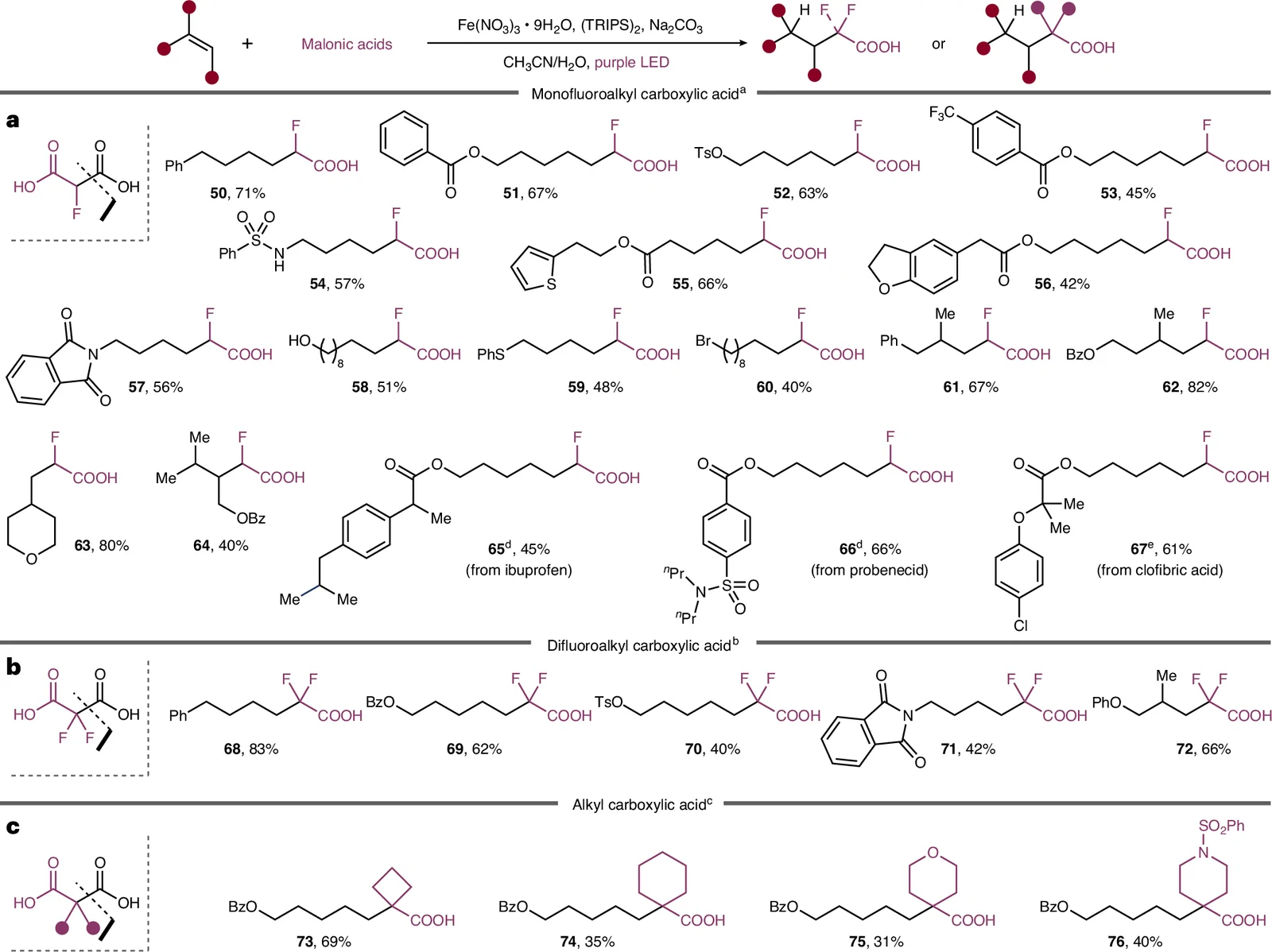
Substrate scope of hydroalkyl carboxylation of alkenes

Standard conditions were as follows: alkene (0.1 mmol, 1.0 equiv.), malonic acids (3.0 equiv.), Fe(NO_3_)_3_ ‧ 9H_2_O (10 mol%), (TRIPS)_2_ (10 mol%), Na_2_CO_3_ (10 mol%) and MeCN/H_2_O = 1:1 (0.1 M) or MeCN/H_2_O (4:1, 0.1 M), 24 or 36 h, N_2_, r.t., 390 nm Kessil purple LED (13 W).

^a^MeCN/H_2_O = 1:1 (0.1 M), 24 h.

^b^MeCN/H_2_O (4:1, 0.1 M), 24 h.

^c^MeCN/H_2_O (1:1, 0.1 M), 36 h.

^d^**MA-11** (5 equiv.), 48 h.

^e^48 h. ^*n*^Pr, propyl; Bn, benzyl; Ts, toluenesulfonyl.

**Fig. 3:**
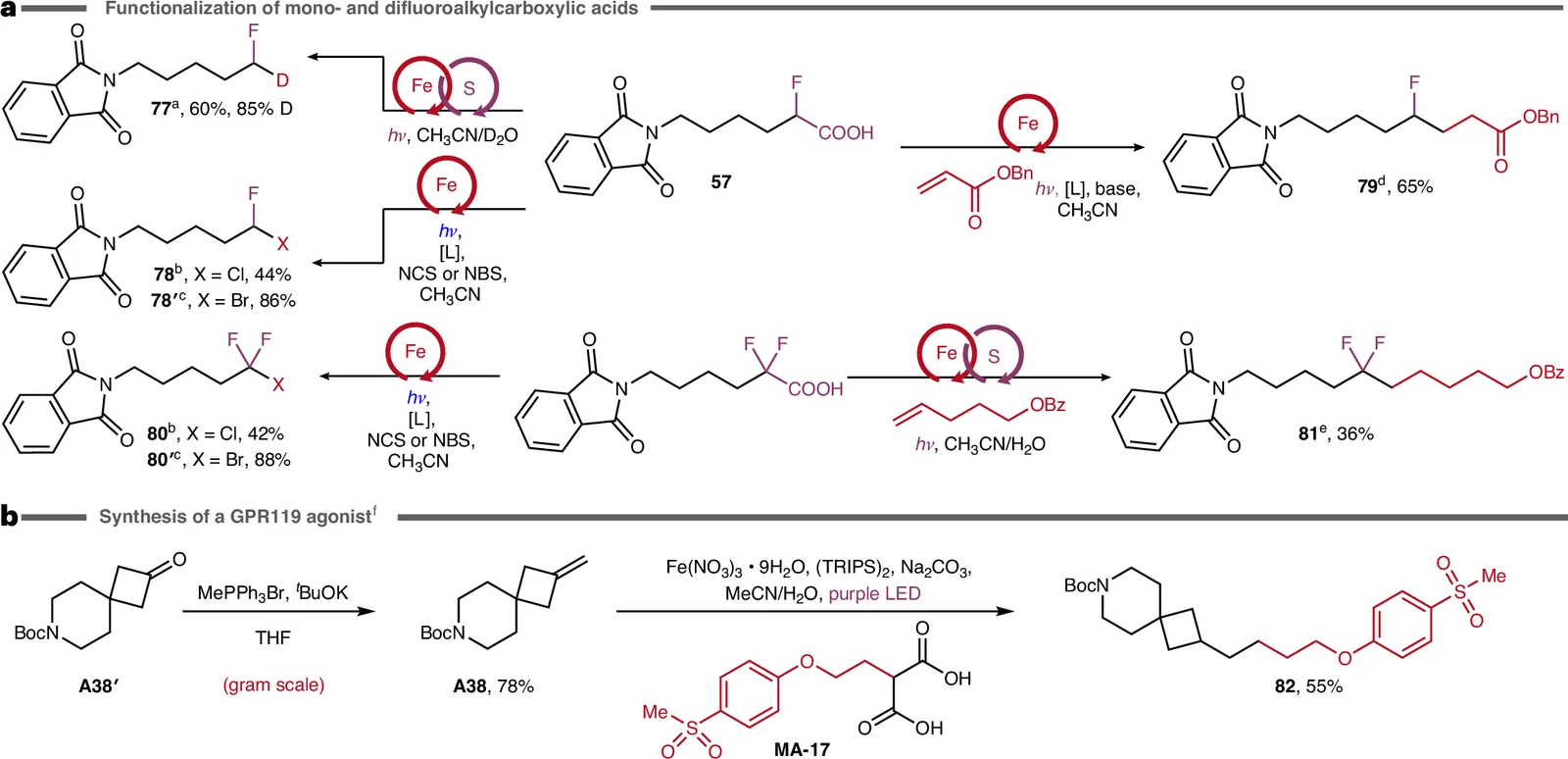
Synthetic utility of the established alkene hydroalkylation and hydroalkyl carboxylation. **a**, Fuctionalization of mono- and difluoroalkyl carboxylic acids. **b**, Synthesis of a GPR119 agonist. ^a^Fe(NO_3_)_3_ ‧ 9H_2_O (10 mol%), (TRIPS)_2_ (10 mol%), carboxylic acid **57** (0.1 mmol) and Na_2_CO_3_ (10 mol%), MeCN/D_2_O (9:1, 0.1 M), 390 nm Kessil light (13 W), N_2_, r.t. and 48 h. ^b^Fe(NO_3_)_3_ ‧ 9H_2_O (10 mol%), DMBP (10 mol%), **57** or **71** (0.1 mmol), Na_2_CO_3_ (10 mol%), NCS (2 equiv.), MeCN (0.1 M), 427 nm Kessil light (11 W), N_2_, r.t. and 24 h. ^c^Footnote b was followed but with NBS (2 equiv.). ^d^FeCl_2_ (5 mol%), dien (5 mol%), **57** (0.1 mmol), DIPEA (1.0 equiv.), benzyl acrylate (1.5 equiv.), MeCN (0.05 M), 390 nm Kessil light (13 W), N_2_, r.t. and 48 h. ^e^Fe(NO_3_)_3_ ‧ 9H_2_O (10 mol%), (TRIPS)_2_ (10 mol%), **71** (3.0 equiv), Na_2_CO_3_ (10 mol%), **A2** (0.1 mmol), MeCN/H_2_O (9:1, 0.1 M), 390 nm Kessil light (26 W), N_2_, r.t. and 48 h. ^f^The mixture of MePPh_3_Br (16.0 mmol, 1.3 equiv.), ^*t*^BuOK (18.8 mmol, 1.5 equiv.), **A38′** (12.5 mmol, 1 equiv.), 0 °C–r.t. and 20 h; Fe(NO_3_)_3_ · 9H_2_O (0.15 mmol, 15 mol%), Na_2_CO_3_ (0.1 mmol, 10 mol%), (TRIPS)_2_ (0.2 mmol, 20 mol%), **MA-17** (3 mmol, 3.0 equiv.) and **A38** (1 mmol, 1 equiv.) in MeCN/H_2_O = 1:1 (0.1 M), 48 h, N_2_, 40 °C, 390 nm Kessil purple LED (52 W). The Supplementary Information contains more-detailed synthetic procedures. Boc, *tert*-butyloxycarbonyl; DMBP, 4,4′-dimethoxy-2,2′-bipyridine; dien, diethylenetriamine; DIPEA, *N*,*N*-diisopropylethylamine; L, ligand; THF, tetrahydrofuran; NBS, N-bromosuccinimide; NCS, N-chlorosuccinimide.

The thorough investigations on alkene hydroalkylation via our general and modular strategy, leveraging synergistic iron-and-thiol co-catalysis and malonic acids as TRPR precursors, further encouraged us to explore the utility of malonic acids as valuable yet inexpensive C-1 linkers, accessing an underexplored chemical space with value-added –CHF–, –CF_2_– and –C(4°)– motifs in drug discovery by using cheap and accessible olefin chemical feedstocks. Through careful control of reaction time, light intensity and co-solvent ratio, a variety of alkenes were all tolerated well and delivered α-fluorocarboxylic acids in good to high yields (Table 3a). Aryl-substituted (**50**, **51** and **53**), sulfonamide (**54**) and sulfonate (**52**) alkenes proceeded smoothly to give the desired products in moderate to good yields. Heterocyclic substrates containing sulfur (**55**), oxygen (**56**) and nitrogen (**57**) all showed good reactivities, providing the expected products at moderate to good yields, and demonstrating the viability of this strategy in pharmaceutical synthesis. Oxidatively labile alcohols and sulfides (**58** and **59**), and reductively labile bromides (**60**), produced the corresponding products at comparable yields. Alkenes featuring different substitution patterns (**61**–**64**) underwent hydrofluoromethylcarboxylation well to produce the corresponding products in useful yields. Notably, selected complex alkenes derived from drug molecules, for example, ibuprofen, probenecid acid and clofibric acid, were found to be well tolerated under standard conditions, affording the corresponding α-fluorocarboxylic acids **65**–**67** in moderate to good yields. The introduction of –CF_2_– units to organic molecules to function as important bioisosteres to diverse functional groups has been gathering enormous interest in the pharmaceutical industry, leading us to explore the installation of this group^7^. We further showcased the practicability of our modular strategy for achieving the precursors, that is, alkyldifluorocarboxylic acids, of such motifs by simply replacing fluoromalonic acid with also-easily-accessible difluoromalonic acid (Table 3b). Various unactivated alkenes were all accommodated under standard conditions to give the desired products **68**–**72**. Leveraging insights from our previous work^44^ and findings in the optimization of hydroethlyation (Supplementary Table 7), we discovered that vigorous control of reaction temperature (room temperature < 30 °C) and light intensity (13 W) allows the protodecarboxylation step of the alkyl carboxylic acid intermediates to be suppressed, enabling direct synthesis of carboxylic acids. This approach allows different cycloalkane-1,1-dicarboxylic acids to be used as C(4°) synthons for the hydroalkyl carboxylation, furnishing products with C(*sp*^3^)-rich scaffolds **73**–**76** in moderate to good yields, and providing an efficient and sustainable method for increasing the three dimensionality of the ubiquitous alkene building blocks (Table 3c). Finally, with a series of carboxylic acids in hand, our study then focused on the selective activation of the remaining carboxyl groups on their skeletons by choosing compound **57** as a model substrate through various iron-mediated direct decarboxylative functionalization methods (Fig. 3a). First, employing the cooperative iron-and-thiol catalysis deuterodecarboxylation protocol allowed compound **57** to be advanced into deutero-containing fluoroalkane **77** with high deuterium incorporation. Next, the use of *N*-chlorosuccinimide (NCS) or *N*-bromosuccinimide (NBS) as both oxidant and halogenating reagent allowed *gem*-di(hetero)haloalkanes **78** and **78′** to be obtained via iron-catalysed direct decarboxylative halogenation protocols. Similarly, (hetero)halodifluoroalkanes **80** and **80′** were able to be prepared in moderate to high yields through the same protocols. Further, following an iron-catalysed decarboxylative Giese reaction protocol, we were able to transform carboxylic acid **57** to the corresponding secondary fluoroalkane product **65** in good yield with an electron-deficient olefin, benzyl acrylate, as a coupling partner. Notably, difluoromalonic acid could act as an alternative –CF_2_– surrogate, successfully connecting two different unactivated alkenes to deliver *gem*-difluoroalkane product **81** via two sequential synergistic iron-and-thiol catalysis reactions (anti-Markovnikov hydroalkyldifluorocarboxylation and hydrodifluoromethylation). Finally, the synthesis of a bioactive molecule GPR119 agonist **82** drew our attention as an opportunity to demonstrate the applicability of this strategy^38^. To improve the reported linear synthetic route involving eight steps of olefinations, functional-group transformations and reductions for constructing its alkyl spacer, we envisioned applying anti-Markovnikov hydroalkylation to an azaspiro alkene **A38** with a 4-(methylsulfonyl)phenoxy-containing malonic acid **MA-17** for rapidly forging the four-carbon chain spacer of GPR119 agonist **82**. The hydroalkylation proceeded smoothly to convergently access target **82** in 55% yield (Fig. 3b), suggesting an opportunity of our strategy to modularly prepare related analogues by using different malonic acid alkyl donors.

### Mechanistic basis of anti-Markovnikov hydroalkylation

**Fig. 4:**
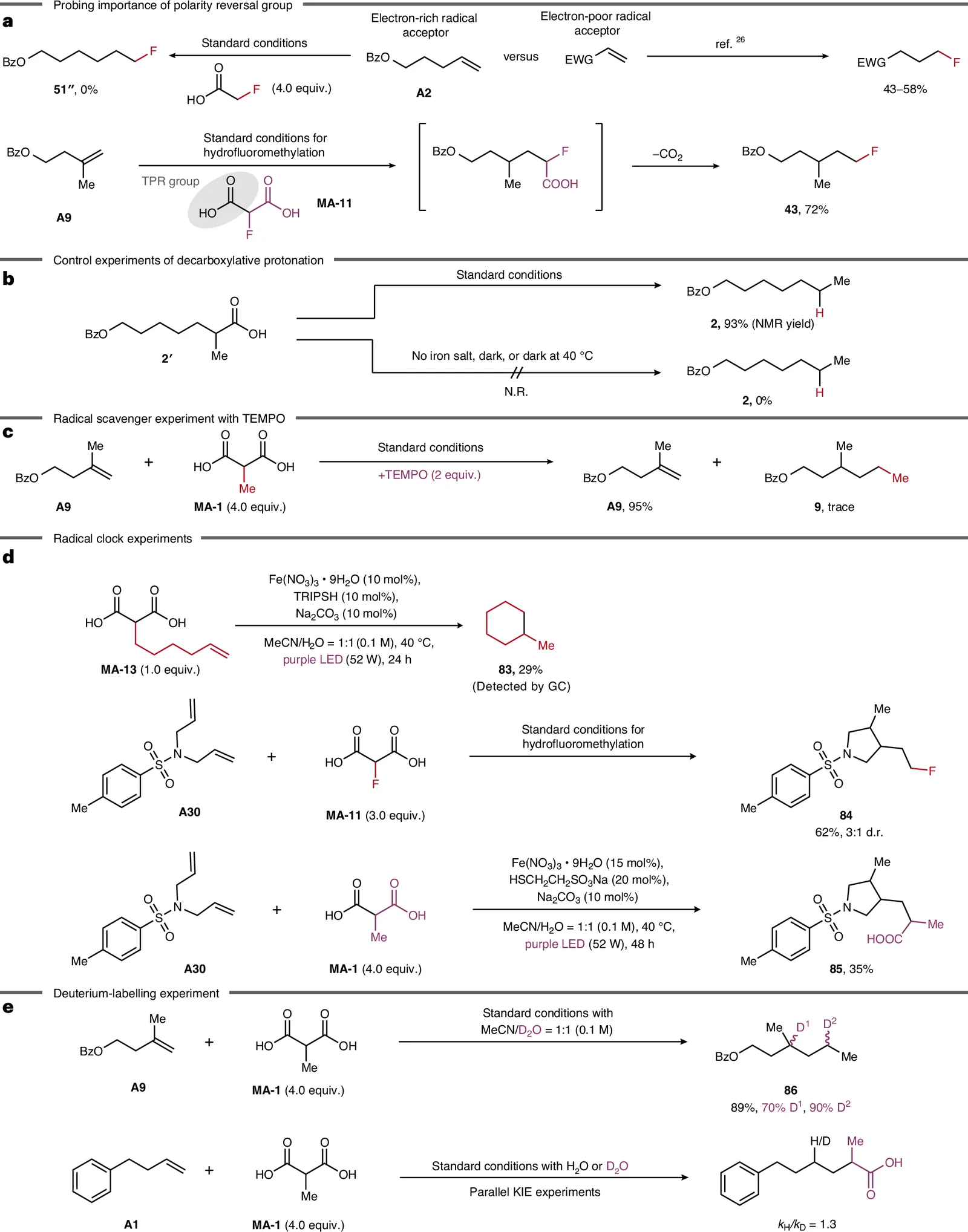
Mechanistic studies of alkene hydroalkylation. **a**, Importance of the transient polarity-reversal group in enabling alkene hydroalkylation. **b**, Control experiments supporting light- and iron-dependent hydrodecarboxylation. **c**, Radical trapping experiments support the involvement of a radical pathway. **d**, Radical clock studies support alkyl radical generation and a thiol-mediated HAT process. **e**, Deuterium-labelling and KIE studies support the proposed dual-catalytic mechanism. EWG, electron-withdrawing group; N.R., no reaction.

To acquire preliminary mechanistic insights into our synergistic hydroalkylation protocol, we first aimed to obtain insight into the importance of the polarity-reversal group for alkene addition (Fig. 4a). When alkene (**A2**) and α-fluoroacetic acid were subjected to our standard conditions, we were unable to observe fluoroalkyl radical addition to form desired hydrofluoroalkylation product **51″** after decarboxylation. This result is consistent with our previous work, where the relatively nucleophilic fluoromethyl radical was able to add efficiently only to electron-poor alkenes^26^. Knowing that the resulting outcome of **A2** hydrofluoroalkylation was dedicated to the potential polarity-mismatched addition, we then envisioned the further introduction of a carboxyl group as a transient polarity-reversal (TPR) group into our alkylating reagent, with α-fluoroacetic acid now becoming α-fluoromalonic acid **MA-11**, to tackle this problem. Herein, when alkene **A9** was reacted with α-fluoromalonic acid **MA-11** under standard conditions, we were finally able to afford the desired product **43** in 72% yield, demonstrating the essential function of the polarity-reversal carboxyl group in alkene hydroalkylation and further supporting our TRPR mechanistic proposal. Subsequent ultraviolent–visible (UV–vis) analysis revealed the formation of potential iron–malonate reactive species and their ability to undergo photolysis under visible-light irradiation (Supplementary Figs. 19 and 20). These findings, in conjunction with control experiments (Table 1, entries 2–4) and previous studies demonstrating the inner-sphere photo-redox process of iron(III) malonate^51,52^, suggest that the iron(III)-mediated oxidation of malonic acids followed by decarboxylation to generate α-carboxyalkyl radicals under our conditions is a photochemical process. Additional control reactions further demonstrated that both iron catalyst and light irradiation are essential for the hydrodecarboxylation in the second step of our reaction. The absence of either component resulted in the full recovery of the starting material and no formation of the desired hydroethylation product **2**, which was otherwise obtained in a high yield (Fig. 4b). Next, inclusion of radical scavenger 2,2,6,6-tetramethyl-1-piperidinyloxy (TEMPO) in the system drastically suppressed the reaction, with a high recovery of starting material (**A9**), supporting the involvement of radical intermediates (Fig. 4c). To further support a radical pathway, we applied three different radical clock substrates. Alkene-bearing malonic acid substrate **MA-13** underwent intramolecular 6-*exo*-trig cyclization to form product **83** in 29% yield as measured by gas chromatography (GC), supporting the generation of α-carboxyl radical species from malonic acid through iron-mediated VLIH (Fig. 4. and Supplementary Fig. 5). *N*-tosylated-tethered diene (**A30**) was subjected to our hydrofluoromethylation protocol to obtain 5-*exo*-trig product **84**, and to our hydroethylation protocol by the use of sodium 2-mercaptoethanesulfonate instead of TRIP disulfide to prevent the formation of undesired thiol-trapped 5-*exo*-trig product, to afford 5-*exo*-trig carboxylic acid **85**, further supporting the intermediacy of radicals and revealing that the thiol HAT step is slower than the rate constant of 2 × 10^5^ s^−1^ (approximate, for 5-*exo*-trig), a result consistent with our hydrofluoroalkylation study^6^ (Fig. 4d). Next, a deuterium-labelling study was performed to track the major proton source for the reaction (Fig. 4. and Supplementary Fig. 7). As expected from our proposed mechanism, replacing H_2_O with D_2_O in our standard conditions resulted in a high yield of deuterated product **86** with high deuterium incorporation in both positions participating in thiol-mediated HAT under TRPR, again supporting our reaction design. Further kinetic isotope effect (KIE) studies were conducted to examine the initial HAT step. Parallel reactions using H_2_O and D_2_O yielded a non-significant KIE value of 1.3 (ref. ^53^; Fig. 4. and Supplementary Table 8). This finding, along with our previous KIE studies on the hydrodecarboxylation step (rate constants with H and D, *k*_H_/*k*_D_ ≈ 1)^44^, suggests that neither HAT process is likely to be rate determining in our reaction. To gain further kinetic insights into our dual-catalytic system, we performed preliminary initial rate experiments focusing on the radical addition step (Supplementary Tables 9 and 10). These experiments revealed a zeroth-order dependence on alkene, acid and disulfide, and a first-order dependence on iron and light intensity. Additionally, our previous kinetic studies demonstrated that hydrodecarboxylation is light limited for aliphatic acids, showing zeroth-order behaviour for all reaction components and a rate acceleration with the use of multiple light-emitting diode (LED) lamps^44^. Together, these data suggest that the VLIH decarboxylation of iron(III) malonate species, generated under a saturated loading of malonic acid, potentially has a rate-determining character and that the rate is influenced by light intensity. To examine whether our hydroalkylation reaction is a chain process or a closed-cycle process, the standard reaction of **A1** was conducted using alternating intervals of light and dark; we observed that the formation of both carboxylic acid intermediate **87** and hydroalkylation product **1** occurred only during periods of constant irradiation (Supplementary Fig. 14). Further facile quantum-yield experiments of the standard reaction of **A1** were also performed, providing low quantum yields (*ϕ*) for both the formation of **87** (*ϕ* = 0.5%) and the protodecarboxylation of **87** to **1** (*ϕ* = 0.44%; Supplementary Information, in the section on quantum-yield measurement). These two experiments demonstrated that the potential radical chain pathway is less likely, and that continuous light irradiation is essential for pushing both carboxylalkyl radical addition and protodecarboxylation to completion. Lastly, we examined the reaction progression of alkene hydroalkylation using three stereoelectronically differentiated malonic acid derivatives (**MA-1**, **MA-4** and **MA-11**) by ^1^H NMR spectroscopy (Supplementary Figs. 16–18). At the beginning of the reaction, the ^1^H NMR data indicated a slower formation of the carboxylic acid intermediates (**87** and **89**) and a slower consumption of the starting materials (**A1** and **A9**) when the more electron-rich alkyl donors **MA-1** and **MA-4** were used. By contrast, employing the more electron-deficient alkyl donor **MA-11** resulted in a faster decay of starting material **A9** and a more rapid appearance of the corresponding carboxylic acid intermediate **62**. The moderate nucleophilicity and steric hindrance of the carboxylalkyl radical derived from **MA-4** likely contributed to its slower radical addition rate relative to **MA-1**, leading to a lower overall yield of the alkyl carboxylic acid intermediate **89**. Furthermore, the initial formation of the hydroalkylation products (**1** and **36**) was observed at approximately 12–15 h for both **MA-1** and **MA-4**, and in each case, these products became the major species in the reaction mixture after roughly 30 h. By contrast, the electron-deficient carboxylfluoromethyl radical generated from **MA-11** appeared to undergo radical addition much more rapidly, reaching the maximum concentration of intermediate **62** within approximately 2 h, followed by the onset of conversion of starting material **A9** into the hydrofluoromethylation product **43**. Ultimately, product **43** became the major species in the reaction mixture after 18 h, which is faster than the time required for the **MA-1** and **MA-4** cases.

## Conclusions

In summary, we demonstrated a modular redox-neutral protocol for anti-Markovnikov hydroalkylation of alkenes with malonic acids as coupling partners using TRPR, a mechanistic approach leveraging the synergy of earth-abundant iron and thiol co-catalysts. Moreover, we further highlighted the modularity of this strategy for the introduction of (di)fluoromethylene and quaternary carbon motifs by the employment of (di)fluoromalonic acids and cycloalkane-1,1-dicarboxylic acids as the corresponding C-1 surrogates, providing an opportunity to approach underexplored chemical spaces using abundant chemical feedstocks. We anticipate this TRPR strategy may enable additional hydrofunctionalizaion reactions using inexpensive, earth-abundant metal catalysis and may enable sustainable syntheses of value-added chemicals in both modern synthetic and industrial chemistry research.

## Methods

The following general procedure was used for iron-catalysed anti-Markovnikov alkene hydroalkylation. To an oven-dried 16 ml borosilicate glass tube containing a magnetic stir bar, we added Fe(NO_3_)_3_ · 9H_2_O (15 mol%), Na_2_CO_3_ (10 mol%), bis(2,4,6-triisopropylphenyl)disulfide (25 mol%) or 2,4,6-triisopropylthiophenol (10 mol%), malonic acid (3.0–4.0 equiv.) and, optionally, alkene (0.1 mmol, 1.0 equiv.; added at the beginning if solid). The tube was sealed and the vial was evacuated and backfilled with N_2_ (repeated four times). MeCN (0.5 ml) was added into the tube, followed by, optionally, alkene (0.1 mmol, 1.0 equiv.; added at this stage if liquid) and water (0.5 ml) via syringe under N_2_. The reaction mixture was pre-stirred for 15 min and then placed under a 390 nm Kessil lamp at 40 °C to react for 48 h. Following this, the crude mixture was concentrated and purified by preparative thin-layer or silica gel chromatography to give the corresponding hydroalkylated product.

## Supplementary information

Supplementary Information Supplementary Figs. 1–234, Tables 1–12, Methods and references.

## Data Availability

Data in the main text or the Supplementary Information are available from the corresponding author upon request.
