# Supplementary material for: Anti-Markovnikov alkene hydroalkylation via iron photocatalysis
Source: Nat Catal. 2026 Sep 15;9(9):976–88. doi: 10.1038/s41929-026-01600-0 (PMC13597299; doi:10.1038/s41929-026-01600-0)
Supplement: Supplementary file 1 [file 41929_2026_1600_MOESM1_ESM.pdf]

nature catalysis

Supplementary information

https://doi.org/10.1038/s41929-026-01600-0

Anti-Markovnikov alkene hydroalkylation via iron photocatalysis

In the format provided by the authors and unedited

Contents

Supplementary Methods 3

General Information **3**

Reaction Screening **5**

Experimental Procedures **12**

*General procedure Ⅰ for the synthesis of unactivated alkenes **A2**, **A9**, **A31 – 32**, and **A35 – 37*** 13

*General procedure Ⅱ for the synthesis of unactivated alkenes **A6** and **A7*** 13

*General procedure Ⅲ for the synthesis of unactivated alkenes **A3**, **A4**, **A11–13**, **A15**, **A24**, **A25**, **A26**, **A43**, **A44**, **A45*** 14

*General procedure Ⅳ for the synthesis of unactivated alkenes **A8**, **A14**, and **A39*** 15

*Synthesis of (1S,2S,4aR,4bR,7S,9aR,10aR)-1-methyl-8-methylene-13-oxo-1,2,5,6,8,9,10,10a-octahydro-4a,1-(epoxymethano)-7,9a-methanobenzo[a]azulene-2,7(4bH)-diyl diacetate **A29**.* 16

*Synthesis of **A30** and **A46*** 16

*Synthesis of N,N-diallyltosylamide **A38*** 17

General Procedure Ⅴ for the Synthesis of Malonic acids 18

*Synthesis of **MA-7*** 19

*Synthesis of **MA-13*** 20

Synthesis of bromide synthon 21

*Synthesis of **MA-17*** 21

Synthesis of bis(2,4,6-triisopropylphenyl)disulfide (TRIP disulfide) 22

General procedure A 23

General procedure B 25

*Synthetic procedure for the hydromethylation of **A9*** 26

*Synthetic procedure for the hydromethylation of **A35, A36**&**A37*** 27

*Synthetic procedure for the hydrofluoromethylation of **A35**, **A36*** 28

*Procedure for the hydroethylation of **A9** on 0.5 gram scale* 30

General procedure C for hydromonofluorocarboxylation of alkenes 32

General procedure D for hydrodifluorocarboxylation of alkenes 33

General procedure E for hydroalkylcarboxylation of alkenes 34

Procedure for decarboxylative deuteration of monofluorocarboxylic acid 35

Procedure for decarboxylative halogenation of monofluorocarboxylic acid 36

Procedure for decarboxylative monofluoroalkylation 38

Procedure for decarboxylative halogenation of difluorocarboxylic acid 39

Procedure for decarboxylative difluoroalkylation 41

1,6-exo-trig radical-clock experiment 42

Deuterium-labeling experiment 44

Kinetic isotope effect (KIE) experiments 45

Kinetic studies to determine reaction rate law 46

Light on-off experiment 50

Facile quantum yield determination via NMR actinometry[5] 52

Quantum yield measurement 54

Full reaction profiles for the invesitigation of free carboxylic acid intermediate and hydroalkylation product formation 56

Interrogation of the formation of Fe(III) photoactive species through UV-vis spectroscopy 60

Supplementary scope of alkene hydroalkylation 63

NMR characterization of corresponding products **65**

NMR Spectra **122**

Supplementary References 110

Supplementary Methods

General Information

Solvents were obtained from Sigma-Aldrich, Alfa-Aesar, and Acros, and used directly without further purification. Other commercially available reagents were purchased at the highest commercial quality and used without further purification, unless otherwise stated. All the iron catalysts used for reaction were purchased from Strem Chemicals. All reactions were monitored by either **^{1}H NMR**, **^{19}F NMR** or thin layer chromatography (TLC) carried out on 0.25 mm pre-coated silia plates (F-254) purchased from Silicycle, Quebec, Canada, using shortwave UV light as visualizing agent and $KMnO_4$ or phosphomolybdic acid (PMA) as developing agents. Preparative thin layer chromatography was performed using Silica Gel GF silica gel (1000 μm) purchased from MilesScientific, Newark, DE, USA. Flash column chromatography was performed using SiliaFlash-P60 silica gel (40–63 μm) purchased from Silicycle, Quebec, Canada. **^{1}H**, **^{13}C** and **^{19}F** spectra were recorded on a Bruker DRX-600 spectrometers operating at 600 MHz for proton nuclei, 151 MHz for carbon nuclei, and 564 MHz for fluorine nuclei. Chemical shifts were reported in parts per million (ppm) and calibrated using residual undeuterated solvent as an internal reference ($CDCl_3$: 7.26 ppm **^{1}H NMR**, 77.16 ppm **^{13}C NMR**). For reporting NMR peak multiplicities, the following abbreviations were used: s = singlet, d = doublet, t = triplet, q = quartet, quin = quintet, m = multiplet. GC-FID chromatograms were recorded on an Agilent 8860 GC using an Agilent J&W HP-5 (5%-diphenyl)-dimethylpolysiloxane column (30 m x 0.32 mm x 0.25 μm film thickness). ALS injector used a split ratio of 10:1 with an injection volume of 1 μL and a temperature of 300 °C. Flow rate of carrier gas H_2 was maintained at a constant pressure of

1 psi, and the FID temperature was set to 300 °C. Data analysis was completed through OpenLab CDS software. GC-MS chromatograms were recorded on an Agilent 8860 GC and Agilent 5977B GC/MSD, using an Agilent J&W HP-5ms Ultra Inert (5%-phenyl)-methylpolysiloxane column (30 m x 0.25 mm x 0.25 μm film thickness). ALS injector used a split ratio of 10:1 with an injection volume of 1 μL and a temperature of 300 °C. Flow rate of carrier gas H2 was maintained at a constant pressure of 5.5 psi. The mass spectrometer was operated under standard conditions; the instrument was tuned (Agilent EI autotune routine) with a source temperature of 230 °C and a quadrupole temperature of 150 °C. In scan mode, the mass range was m/z 25-550. Data analysis was completed through OpenLab CDS and MassHunter software. High-resolution mass spectra (HRMS) were recorded on an Agilent UHPLC TOF mass spectrometer using electrospray ionization time-of-flight (ESI-TOF), chemical ionization time-of-flight (CITOF) or atmospheric pressure chemical ionization (APCI). All photochemical reactions were performed in 16 mL disposable borosilicate glass tubes with threaded end (Fisherbrand™ catalog Number #14-959-35B) equipped with black unlined phenolic screw cap with open top (Kimble® 73804-15425) and white PTFE/clear silicone septa (VWR Supplier No. B7995-15, VWR Catalog Number #66030-760) on a magnetic stirrer hotplate with temperature probe (Heidolph™ Catalog Number #13-876-206). Wavelength of the Kessil PR160L-390 can be found online: https://kessil.com/products/science_PR160L.php.

Reaction Screening

Reactions were performed at 0.1 mmol scale with the following setup described in general procedure A. Yields were determined by ^{1}H NMR analysis of the crude reaction mixture with CH_2Br_2 as an internal standard.

Supplementary Table 1. (a)Screening of iron catalysts

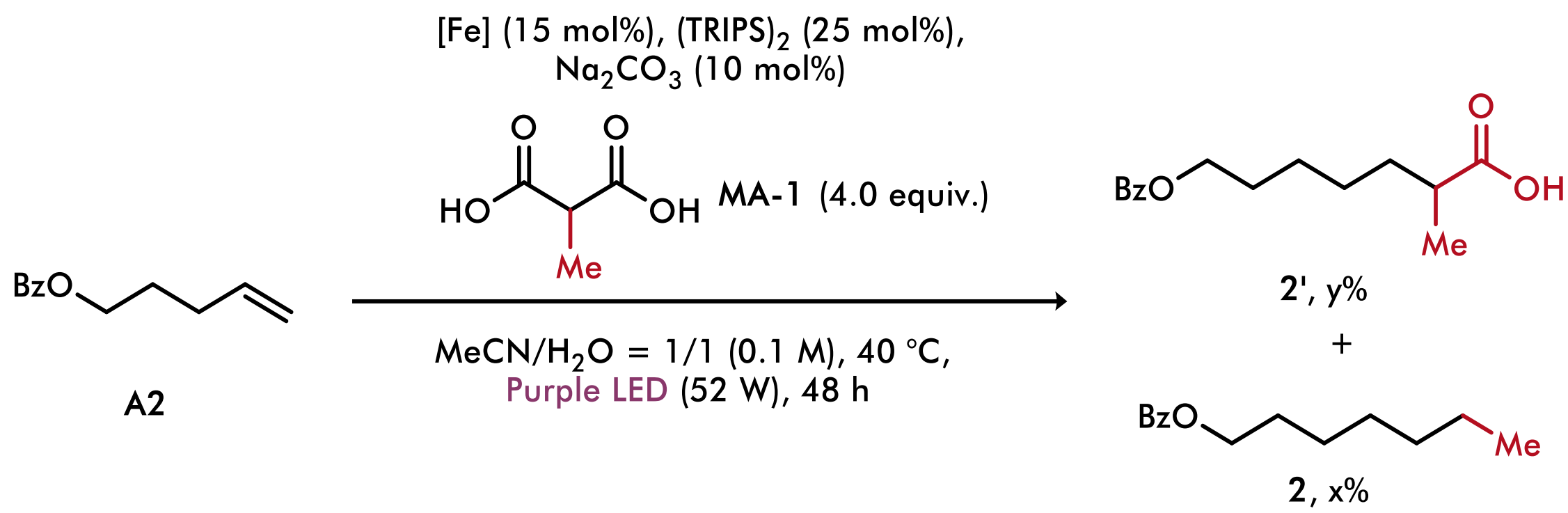

[Fe]	*2*	*2′*
$Fe(NO_3)_3 \cdot 9H_2O$	71%	trace
$Fe(OAc)_2$	35%	trace
$Fe_2(SO4)_3 \cdot 5H_2O$ (7.5 mol%)	62%	trace
$Fe(OTf)_3$	56%	trace
$FeCl_3 \cdot 6H_2O$	70%	trace
$Fe_2(C_2O_4)_3 \cdot 6H_2O$ (7.5 mol%)	trace	trace
$Fe(acac)_3$	7%	trace

Supplementary Table 2. Screening of thiol reagents

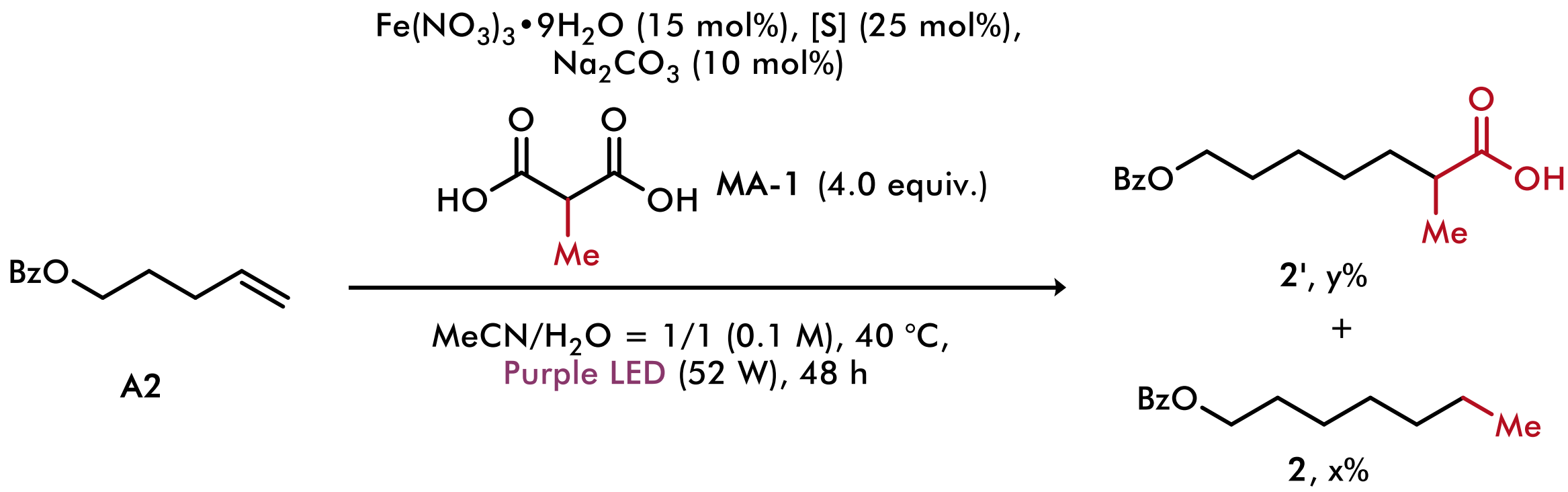

[S]	*2*	*2′*
$(TRIPS)_2$	71%	trace
$(PhS)_2$	4%	40%
TRIPSH	64%	8%
$(4\text{-Cl-PhS})_2$	trace	72%
$(4\text{-F-PhS})_2$	trace	52%
$(4\text{-NO}_2\text{-PhS})_2$	trace	86%
$(4\text{-OMe-PhS})_2$	2%	12%
$(4\text{-OH-PhS})_2$	14%	14%
$HSC_6H_4CO_2H$ (20 mol%)	13%	83%
$HSCH_2CH_2SO_3Na$ (20 mol%)	N.D.	92%

Supplementary Table 3. Base evaluation

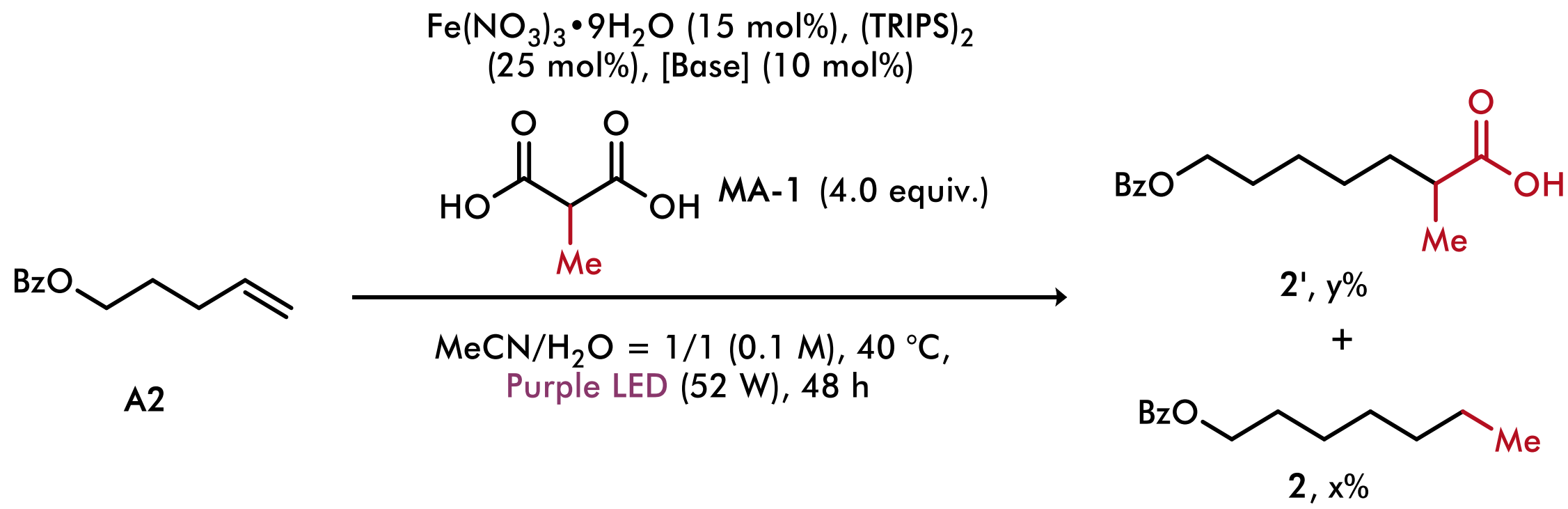

[Base]	*2*	*2′*
Na_2CO_3	71%	trace
Li_2CO_3	63%	trace
K_2CO_3	53%	20%
Cs_2CO_3	71%	trace
Na_2CO_3 (30 mol%)	37%	trace

Supplementary Table 4. Solvent evaluation

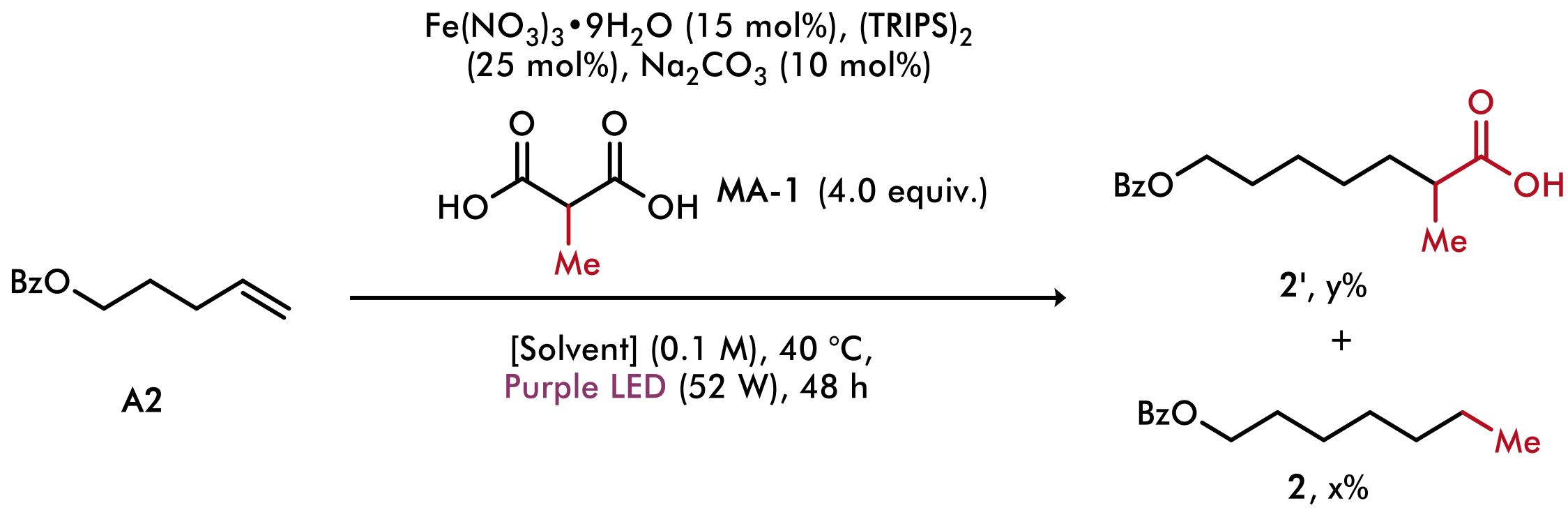

[Solvent]	*2*	*2′*
DCE/H_2O	0%	0%
H_2O	0%	0%
Acetone/H_2O	63%	trace
MeCN/H_2O = 1:1	71%	trace
MeOH/H_2O	51%	13%
THF/H_2O	26%	trace
MeCN/H_2O = 9/1	0%	0%

Supplementary Table 5. Screening the loadings of iron catalyst

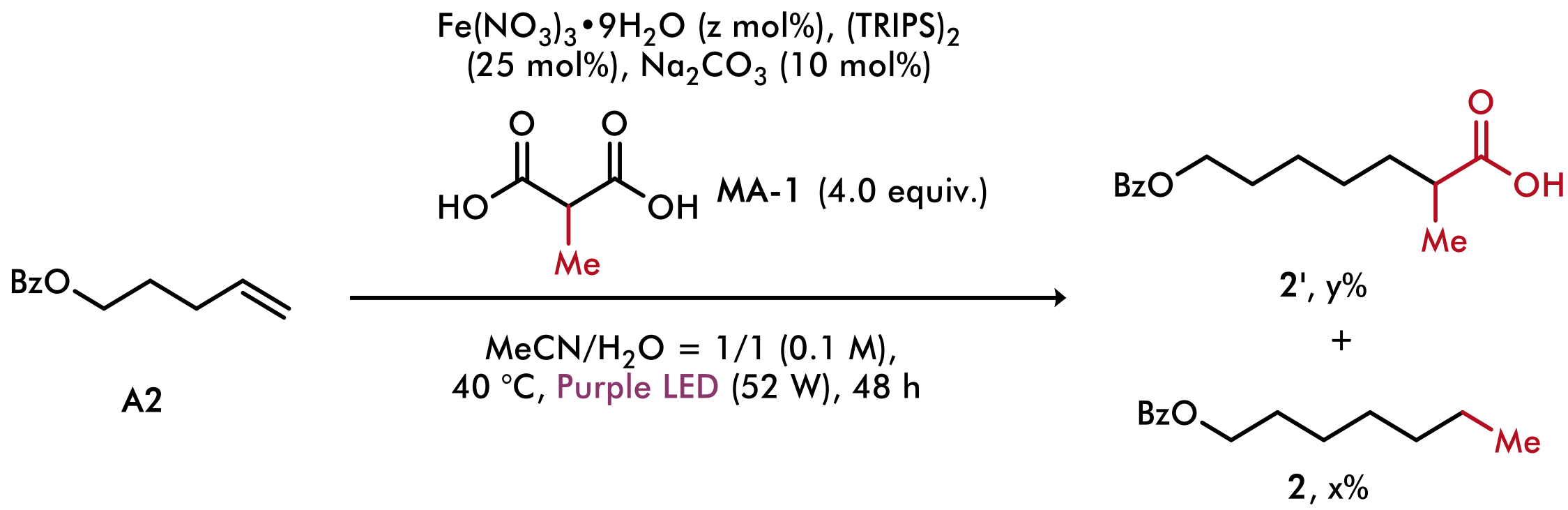

Z mol%	2	2′
5%	60%	trace
10%	61%	trace
15%	71%	trace
20%	69%	trace
30%	70%	trace

Supplementary Table 6. Screening the loadings of TRIP disulfide.

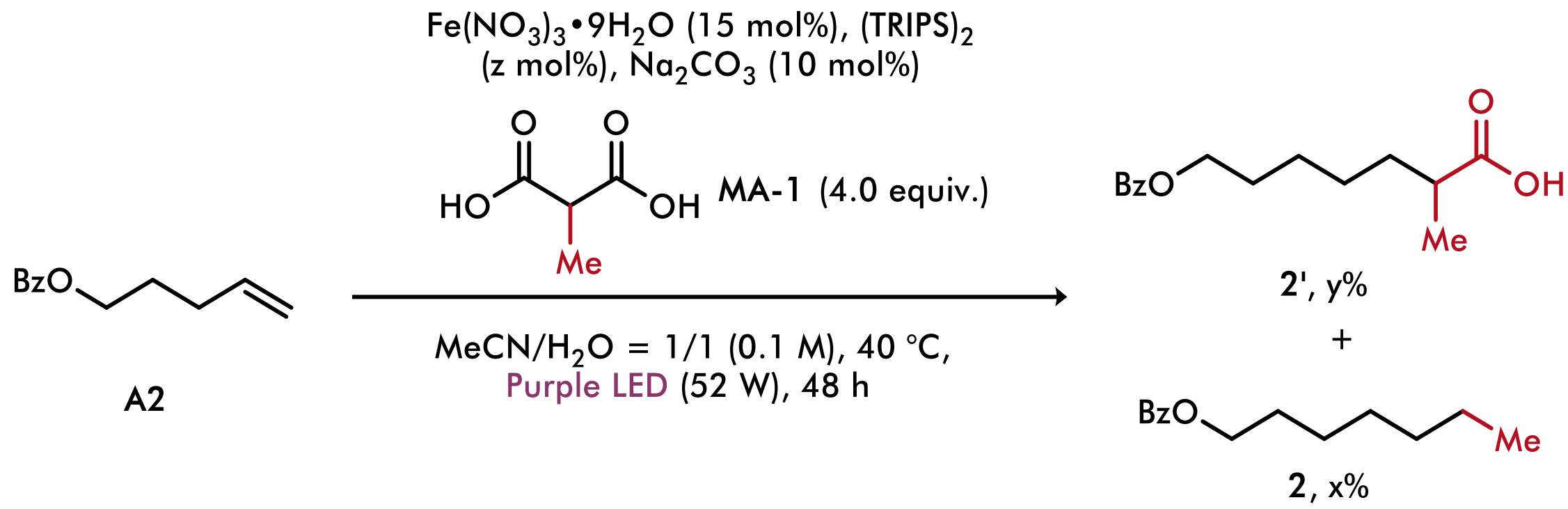

Z mol%	**2**	**2′**
10%	60%	trace
15%	65%	trace
20%	67%	trace
25%	71%	trace
30%	68%	trace

Supplementary Table 7. Effects of reaction temperature on the product yield

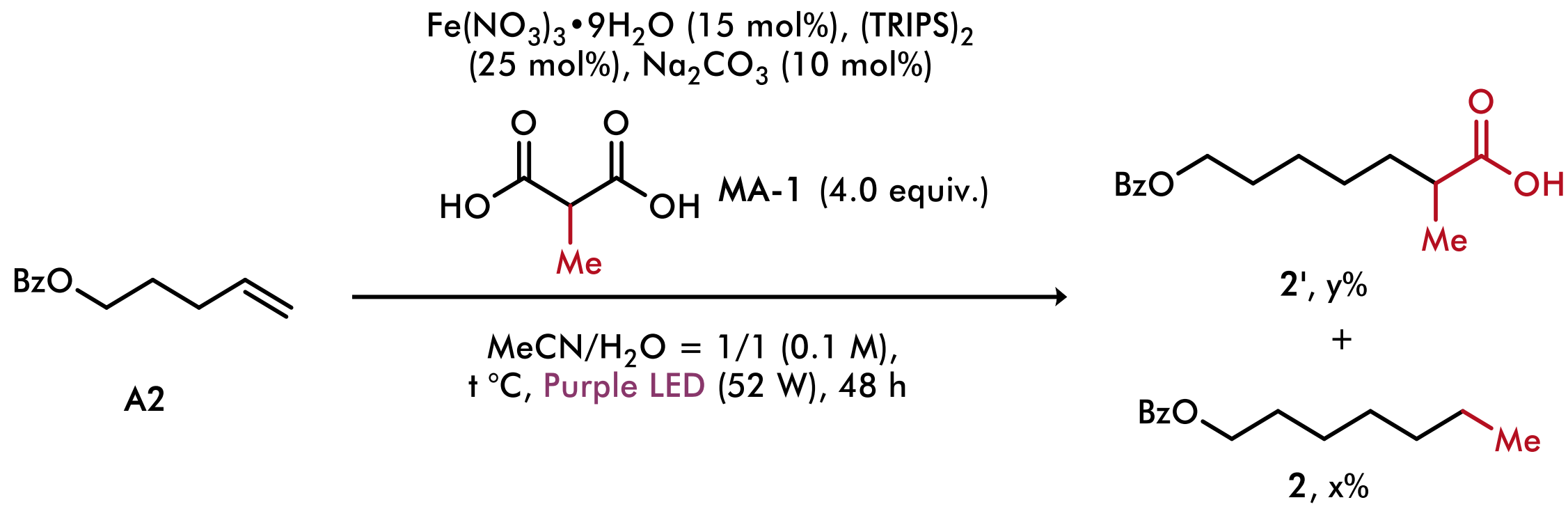

T °C	2	2′
30 °C	41%	31%
40 °C	71%	trace
[a]50 °C	69%	trace
[a]60 °C	73%	trace

a) Reaction was performed in a pre-heated water bath

Experimental Procedures

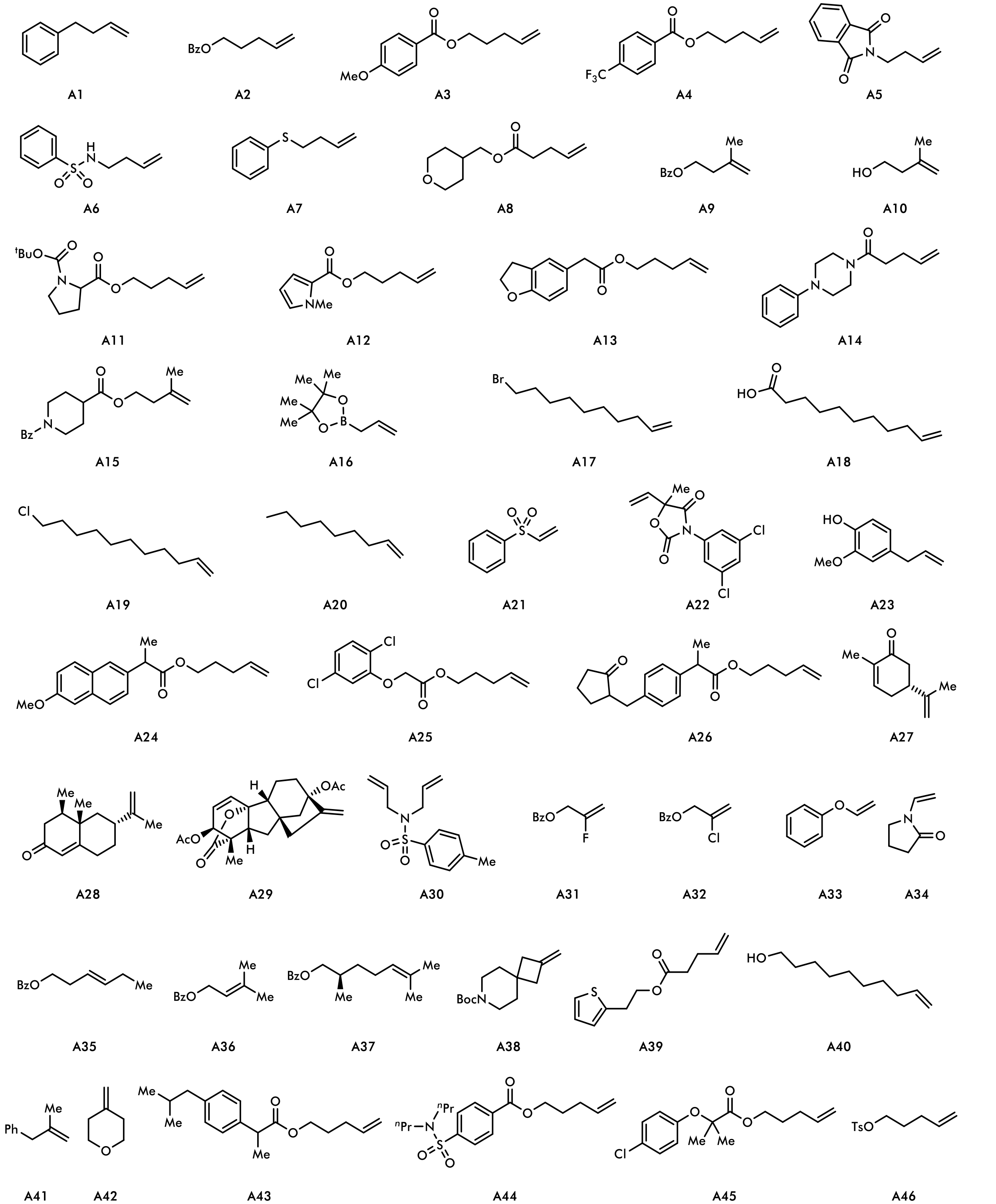

Supplementary Figure 1. List of alkenes tested in hydroalkylation Substrates. **A1**, **A5**, **A10**, **A16–23**, **A27–28**, **A33–34**, and **A40–42** were purchased from commercial vendors. The other Substrates were prepared via the general procedures that follow.

*General procedure I for the synthesis of unactivated alkenes **A2**, **A9**, **A31 – 32**, and **A35 – 37***

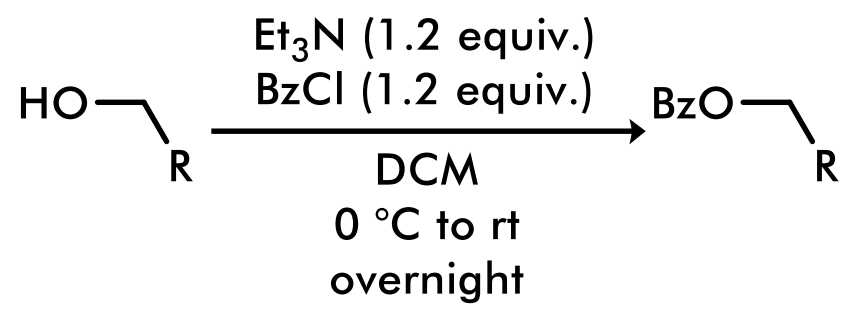

Alcohols (0.86 g, 10 mmol, 1.0 equiv.) and triethylamine (1.7 mL, 12 mmol, 1.2 eq.) were mixed in an RB flask with 20 mL of dichloromethane then cooled to 0 °C. Benzoyl chloride (1.4 mL, 12 mol, 1.2 equiv.) was added to the mixture at 0 °C dropwise under inert atmosphere. After the addition, the mixture was further stirred at 0 °C for 30 min then at rt overnight. The mixture was diluted with 20 mL of DCM then the organic phase was washed with water, 1.0 M NaOH, 1.0 M HCl, brine, respectively. The organic phase was dried over $MgSO_4$, filtered, concentrated, and purified via flash column chromatography using (Hex/EtOAc = 9/1) to give the desired products.

*General procedure II for the synthesis of unactivated alkenes **A6** and **A7***

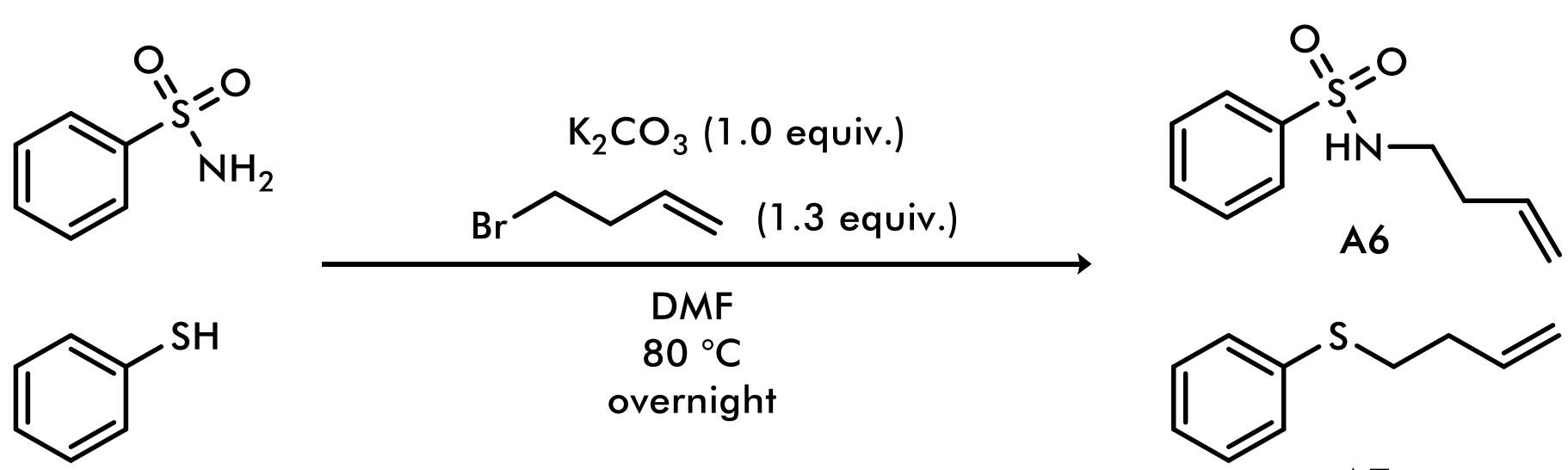

To an RB flask were added benzenesulfonamide (0.94 g, 6.0 mmol, 1.0 equiv.) or thiol phenol (0.66 g, 6.0 mmol, 1.0 equiv.), 4-bromobut-1-ene (0.79 mL, 7.8 mmol, 1.3 equiv.), and dimethylformamide (30 mL). Potassium carbonate (0.83 g, 6.0 mmol, 1.0 equiv.) was added to the reaction mixture and the resulting mixture stirred overnight at 80 °C. Following reaction, the mixture was cooled to room temperature and quenched with water. The reaction mixture was then washed with brine and extracted with diethyl ether. The combined organic phase was concentrated through rotary evaporation. Subsequent flash

column chromatography allowed for isolation of N-(but3-en-1-yl)benzenesulfonamide **A6** (Hex/EtOAc = 3/1) or homoallyl phenyl sulfide **A7** (Hex/EtOAc = 9/1).

General procedure Ⅲ for the synthesis of unactivated alkenes ***A3**, **A4**, **A11–13**, **A15**, **A24**, **A25**, **A26**, **A43**, **A44**, **A45***

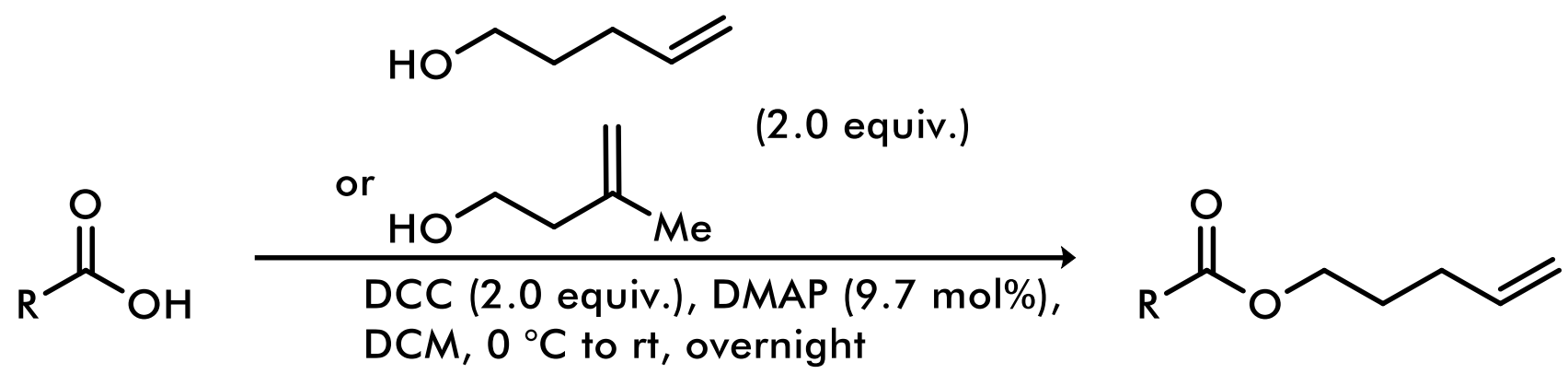

These substrates were prepared according to literature.[1,2] To an RB flask were added 5-pentenol or 2-methyl-1-buten-4-ol (0.43 g, 5.0 mmol, 2.0 equiv.), carboxylic acid (2.5 mmol, 1.0 equiv.), and 4-dimethylamino pyridine (29 mg, 0.24 mmol, 0.097 equiv.). The RB flask was then evacuated and backfilled with nitrogen gas three times. Dry dichloromethane (12 mL) was added via syringe to the RB flask, dissolving the solid components. The RB flask was then placed in an ice bath positioned on top of a stirring plate. Dicyclohexyl carbodiimide (1.0 g, 5.0 mmol, 2.0 equiv.) was added to the mixture via syringe dropwise over a period of 5 minutes. The ice bath was then removed, allowing the reaction to return to room temperature. The reaction was left to stir overnight. Following reaction, the mixture was concentrated through rotary evaporation. Subsequent flash column chromatography (Hex/EtOAc = 3/1) allowed for isolation of the ester.

*General procedure IV for the synthesis of unactivated alkenes **A8**, **A14**, and **A39***

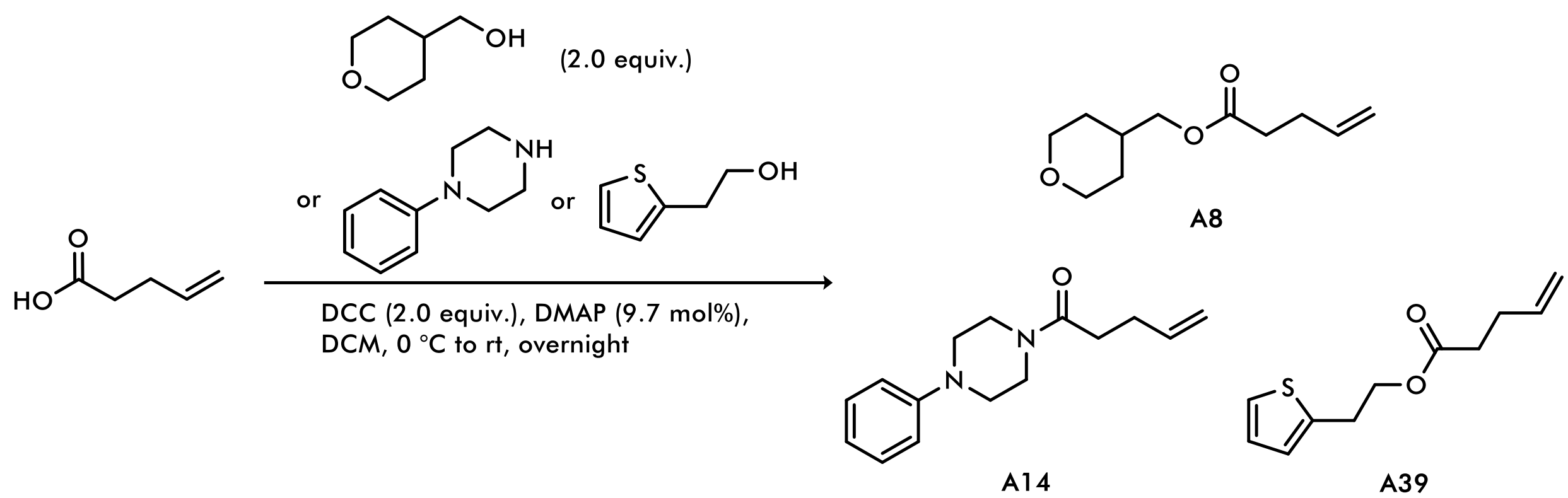

These substrates were prepared according to the literature.[1,2] To an RB flask were added 5-pentenoic acid (0.26 mL, 2.5 mmol, 1.0 equiv.), alcohol or amine (5.0 mmol, 2.0 equiv.), and 4-dimethylamino pyridine (29 mg, 0.24 mmol, 0.097 equiv.). The RB flask was then evacuated and backfilled with nitrogen gas three times. Dry dichloromethane (12 mL) was added via syringe to the RB flask, dissolving the solid components. The RB flask was then placed in an ice bath positioned on top of a stirring plate. Dicyclohexyl carbodiimide (1.0 g, 5.0 mmol, 2.0 equiv.) was added to the mixture via syringe dropwise over a period of 5 minutes. The ice bath was then removed, allowing the reaction to return to room temperature. The reaction was left to stir overnight. Following reaction, the mixture was concentrated through rotary evaporation. Subsequent flash column chromatography (Hex/EtOAc = 3/1) allowed for isolation of the ester.

*Synthesis of (1S,2S,4aR,4bR,7S,9aR,10aR)-1-methyl-8-methylene-13-oxo-1,2,5,6,8,9,10,10a-octahydro-4a,1-(epoxymethano)-7,9a-methanobenzo[a]azulene-2,7(4bH)-diyl diacetate **A29**.*

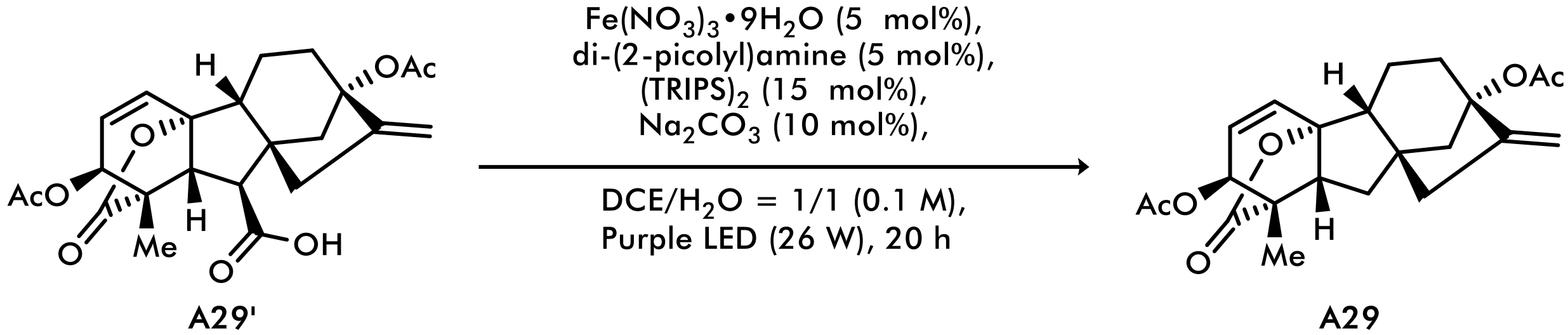

A29 was prepared via the following reported procedures in literature.[3] Subsequent flash column chromatography (Hex/EtOAc = 3/1) allowed for isolation of **A29**.

*Synthesis of **A30** and **A46***

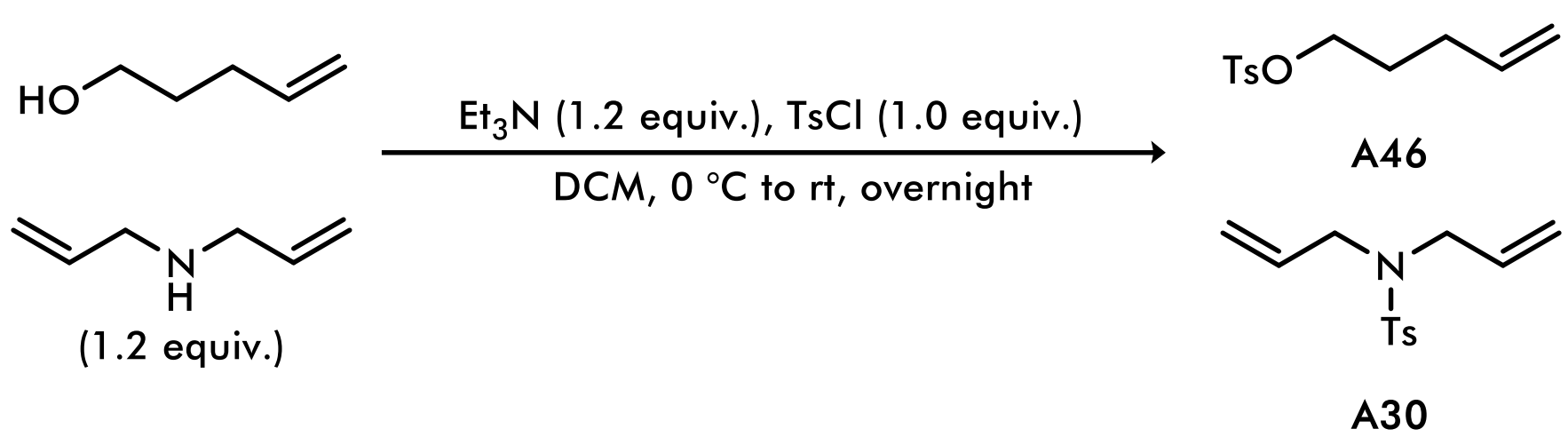

A RB flask was evacuated and backfilled with nitrogen gas three times. To the RB flask were added diallylamine (1.2 mL, 10 mmol, 1.2 equiv.) and dry dichloromethane (30 mL) via syringe. Triethylamine (1.4 mL, 10 mmol, 1.2 equiv.) was then added dropwise to the solution at 0 °C and stirred for 30 mins. Upon slow addition of 4-toluenesulfonyl chloride (1.6 g, 8.3 mmol, 1.0 equiv.), the reaction mixture was then allowed to warm to room temperature and stirred overnight. The organic phase was washed with water and brine, respectively, and dried over $MgSO_4$. The resulting solution was concentrated through rotary evaporation. After evaporation of the solvent, the residue was subjected to flash column chromatography (Hex/EtOAc = 4/1) to give **A30** or **A46**.

*Synthesis of N,N-diallyltosylamide **A38***

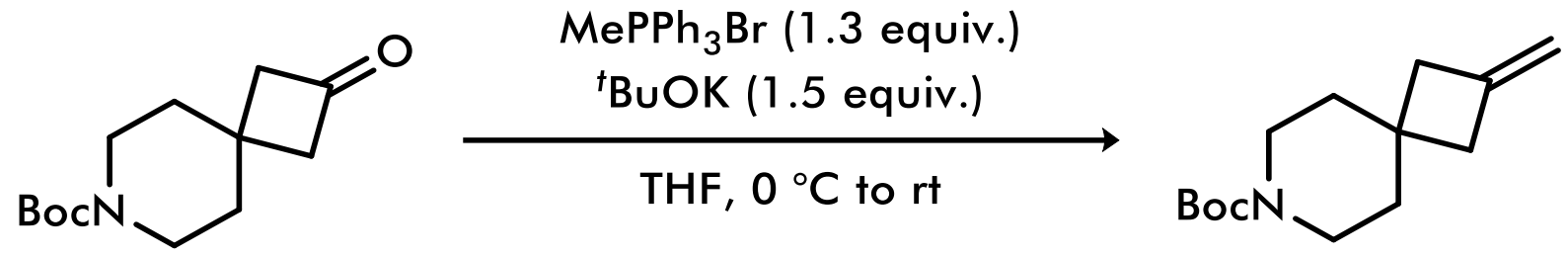

A38

To a stirred solution of methyltriphenylphosphonium bromide (5.7 g, 16.0 mmol, 1.3 equiv.) in dry THF (16 mL) was added a solution of potassium *tert*-butoxide (2.1 g, 18.8 mmol, 1.5 equiv.) in THF (16 mL) dropwise at 0 °C under nitrogen atmosphere and the mixture was stirred for 30 minutes. The mixture was warmed to room temperature and stirred for 30 minutes. To the mixture was added a solution of ketone (tert-butyl 2-oxo-7-azaspiro[3.5]nonane-7-carboxylate) (3.0 g, 12.5 mmol, 1 equiv.) in THF (10 mL) dropwise at 0 °C and the mixture was stirred for 20 hours at room temperature. The reaction was quenched by 30 ml saturated ammonium chloride solution and the organic layer was separated. The aqueous layer was extracted by diethyl ether (3 x 30 mL), and the combined organic layers were dried over anhydrous Na_2SO_4, and concentrated under reduced pressure. The obtained crude product was purified by flash silica-gel column chromatography using Hex/EtOAc (v/v = 9:1) as an eluent to afford alkene **A38** (2.3 g, 78% yield) as a colorless solid. ^{1}H NMR (600 MHz, $CDCl_3$) δ 4.83 (p, *J* = 2.4 Hz, 2H), 3.34 – 3.30 (m, 4H), 2.42 (t, *J* = 2.4 Hz, 4H), 1.55 (t, *J* = 5.8 Hz, 4H), 1.45 (s, 9H) ppm. ^{13}C NMR (151 MHz, $CDCl_3$) δ 155.12, 144.66, 107.82, 79.43, 41.91, 36.76, 33.78, 28.61 ppm.

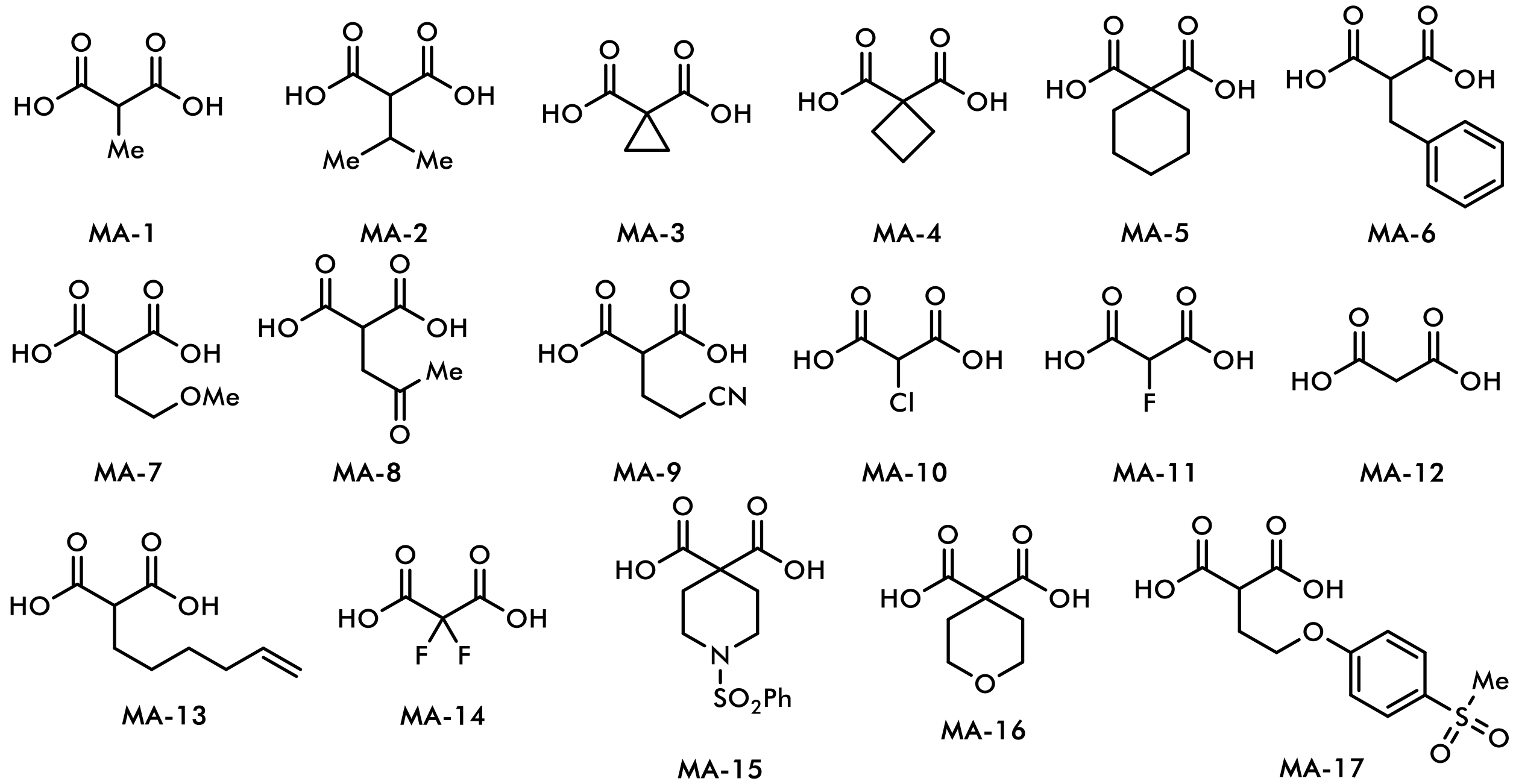

Supplementary Figure 2. List of malonic acids tested in hydroalkylation. **MA-1**, **MA-2**, **MA-3**, **MA-4**, **MA-5** and **MA-12** were purchased from commercial vendors. **MA-6**, **MA-8**, **MA-9** **MA-10**, **MA-11**, **MA-14–16** were prepared via the following general procedure V from the hydrolysis of their corresponding diethyl malonates purchased from commercial vendors. **MA-7**, **MA-13**, and **MA-17** was prepared via the procedures that follow.

General Procedure V for the Synthesis of Malonic acids

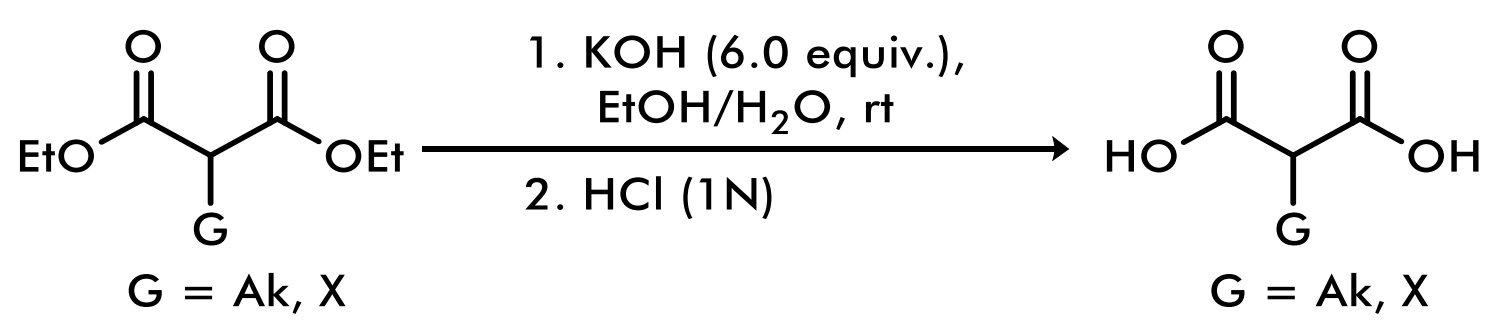

To a solution of potassium hydroxide (1.7 g, 30 mmol, 6.0 equiv.) in EtOH (15 mL) and H_2O (15 mL) was added 2-alkyl or 2-halo diethyl malonate (5.0 mmol, 1.0 equiv.) dropwise. The mixture was stirred at room temperature and monitored with TLC (Hex/EtOAc = 2/0.5). Once the diethyl malonate was totally consumed, the resulting solution was washed with dichloromethane and collected aqueous layer was acidified with 1.0 M HCl at 0 °C until pH

2–3 was reached. The water and EtOH of the aqueous layer was removed with a rotary evaporator under reduced pressure. The white-solid residue was then diluted with EtOAc and dried with Mg_2SO_4. The EtOAc solution was filtered to remove precipatate and concentrated under reduced pressure with a rotary evaporator to give the desired 2-alkyl or 2-halo malonic acid for direct usage without further purification.

*Synthesis of **MA-7***

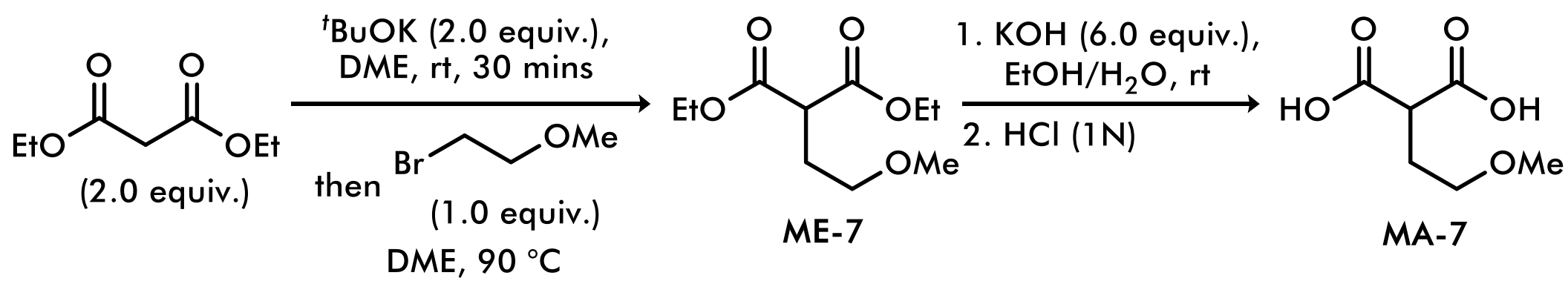

To a stirred solution of diethyl malonate (2.1 mL, 14 mmol, 2.0 equiv.) in dimethoxyethane (30 mL) was portionwise added potassium *tert*-butoxide (1.6 g, 14 mmol, 2.0 equiv.). The resulting mixture was stirred at room temperature for 30 mins. To the resultant solution was added 2-bromoethyl methyl ether (0.66 mL, 7.0 mmol, 1.0 equiv.) in dimethoxyethane (20 mL) through an addition funnel over a period of 30 mins at 90 °C and left to stir until the 2-bromoethyl methyl ether was totally consumed, monitored by thin layer chromatography. Following reaction, the solution was cooled down to room temperature and washed with NH_4Cl (sat.) and brine. The organic phase was dried over $MgSO_4$, filtered, concentrated and purified via flash column chromatography using (Hex/EtOAc = 5:1) to give diethyl 2-(2-methoxyethyl)malonate **ME-7** as a colorless liquid in 85% yield (R_f = 0.2, Hex/EtOAc = 2/0.5). ^{1}H NMR (600 MHz, $CDCl_3$): δ 4.19 (qd, *J* = 7.1 Hz, 1.6 Hz, 4H), 3.52 (t, *J* = 7.3 Hz, 1H), 3.42 (t, *J* = 6.0 Hz, 1H), 3.30 (s, 1H), 2.16 (dt, *J* = 7.3 Hz, 6.0 Hz, 1H), 1.26 (t, *J* = 7.1 Hz, 1H) ppm. ^{13}C NMR (151 MHz, $CDCl_3$): δ 169.4, 69.8, 61.4, 58.6, 49.0, 28.8, 14.1 ppm. Diethyl 2-(2-methoxyethyl)malonate **ME-7** was then hydrolyzed by following the general procedure Ⅴ to obtain the desired malonic acid **MA-7** as a white solid.

Synthesis of ***MA-13***

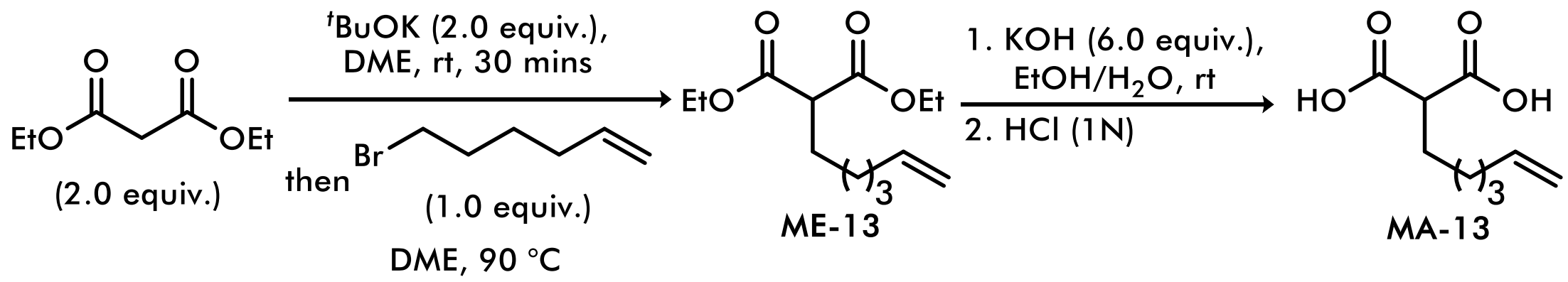

To a stirred solution of diethyl malonate (2.1 mL, 14 mmol, 2.0 equiv.) in dimethoxyethane (30 mL) was portionwise added potassium *tert*-butoxide (1.6 g, 14 mmol, 2.0 equiv.). The resulting mixture was stirred at room temperature for 30 mins. To the resultant solution was then treated with 6-Bromo-1-hexene (0.94 mL, 7.0 mmol, 1.0 equiv.) in dimethoxyethane (20 mL) through a dropping funnel over a period of 30 mins at 90 °C and left to stir until the 6-Bromo-1-hexene was totally consumed, monitored by thin layer chromotography. Following reaction, the solution was cooled down to room temperature and washed with NH_4Cl (sat.) and brine. The organic phase was dried over $MgSO_4$, filtered, concentrated, and purified via flash column chromatography using (Hex/EtOAc = 8:1) to give diethyl 2-(hex-5-en-1-yl)malonate **ME-13** as a colorless liquid in 85% yield (R_f = 0.5, Hex/EtOAc = 2/0.5). ^{1}H NMR (600 MHz, $CDCl_3$): δ 5.78 (ddt, *J* = 17.0, 10.2, 6.7 Hz, 1H), 5.09 – 4.81 (m, 2H), 4.19 (qd, *J* = 7.1, 1.9 Hz, 4H), 3.31 (t, *J* = 7.5 Hz, 1H), 2.20 – 1.96 (m, 2H), 1.94 – 1.80 (m, 2H), 1.48 – 1.39 (m, 2H), 1.38 – 1.31 (m, 2H), 1.26 (t, *J* = 7.1 Hz, 6H) ppm. ^{13}C NMR (151 MHz, $CDCl_3$): δ 169.6, 138.6, 114.6, 61.3, 52.0, 33.4, 28.6, 28.5, 26.8, 14.1 ppm. Diethyl 2-(hex-5-en-1-yl)malonate **ME-13** was then hydrolyzed by following the general procedure V to obtain the desired malonic acid **MA-13** as a white solid.

Synthesis of bromide synthon

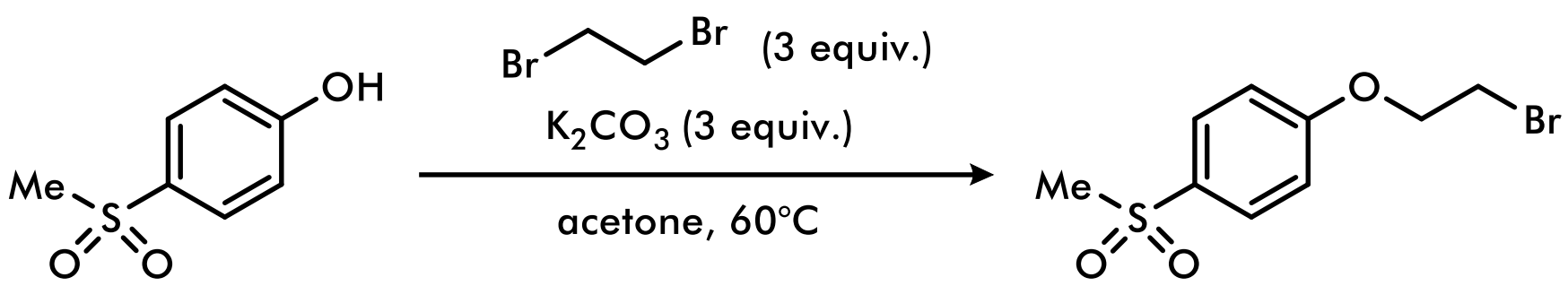

A mixture of 4-(methylsulfonyl)phenol (5.0 g, 29.0 mmol, 1 equiv.), 1,2-dibromoethane (7.5 ml, 87.0 mmol, 3 equiv.), potassium carbonate (12.0 g, 87 mmol, 3 equiv.) in acetone (58 mL) was stirred at 60 °C for 20 h. The reaction solution was cooled down to room temperature and then filtered and the solvent was removed under vacuum to give a crude residue, which was purified by flash silica-gel column chromatography using Hex/EtOAc (v/v = 3:2) as an eluent to afford the product bromide as a white solid (2.9 g, 36%). ^{1}H NMR (600 MHz, $CDCl_3$) δ 7.90 – 7.87 (m, 2H), 7.06 – 7.02 (m, 2H), 4.37 (t, *J* = 6.1 Hz, 2H), 3.67 (t, *J* = 6.1 Hz, 2H), 3.03 (s, 3H) ppm. ^{13}C NMR (151 MHz, $CDCl_3$) δ 162.31, 133.22, 129.83, 115.23, 68.24, 44.97, 28.47 ppm.

Synthesis of ***MA-17***

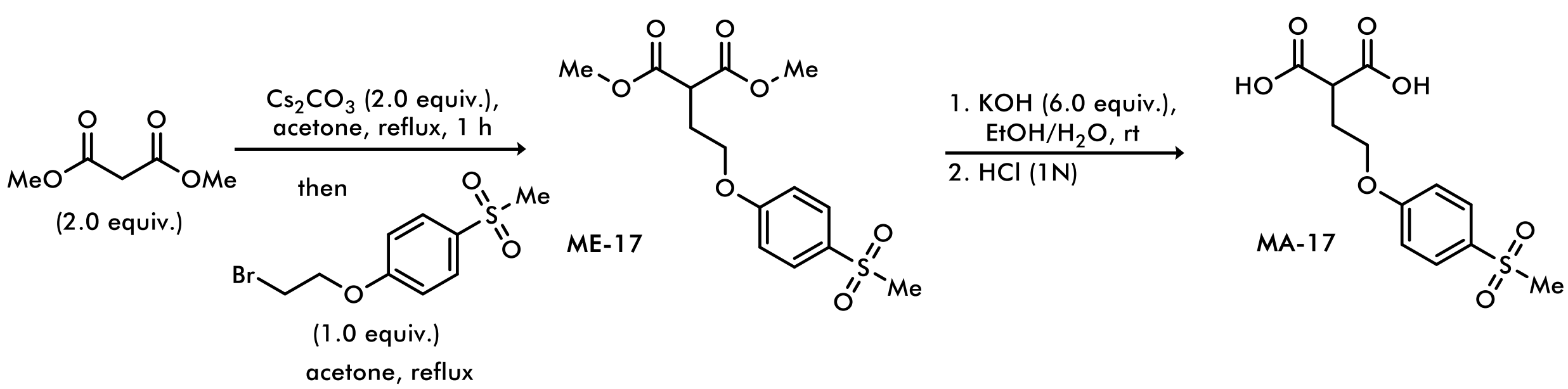

To a stirred solution of dimethyl malonate (0.76 mL, 6.7 mmol, 2.0 equiv.) in acetone (30 mL) was portionwise added Caesium carbonate (2.2 g, 6.7 mmol, 2.0 equiv.). The resulting mixture was brought to reflux and stirred for 1 hour. To the resultant solution was then treated with 1-(2-bromoethoxy)-4-(methylsulfonyl)benzene (0.9 g, 3.3 mmol, 1.0 equiv.) in acetone (20 mL) through a dropping funnel over a period of 30 mins and left to stir until the 1-(2-bromoethoxy)-4-(methylsulfonyl)benzene was totally consumed, monitored by thin

layer chromotography. Following reaction, the solution was cooled down to room temperature and washed with NH_4Cl (sat.) and brine. The organic phase was dried over $MgSO_4$, filtered, concentrated, and purified via flash column chromatography using (Hex/EtOAc = 3:1) to give dimethyl 2-(2-(4-(methylsulfonyl)phenoxy)ethyl)malonate **ME-17** as a white solid in 60% yield. ^1H NMR (600 MHz, $CDCl_3$): δ 7.85 (d, *J* = 8.8 Hz, 2H), 6.98 (d, *J* = 8.8 Hz, 2H), 4.11 (t, *J* = 5.9 Hz, 2H), 3.75 (s, 6H), 3.68 (t, *J* = 7.2 Hz, 1H), 3.01 (s, 3H), 2.42 (q, *J* = 6.3 Hz, 1H). ^{13}C NMR (151 MHz, $CDCl_3$): δ 169.3, 162.6, 132.6, 129.6, 114.9, 65.7, 52.8, 48.3, 44.8, 28.3. ppm. dimethyl 2-(2-(4-(methylsulfonyl)phenoxy)ethyl)malonate **ME-17** was then hydrolyzed by following the general procedure Ⅴ to obtain the desired malonic acid **MA-17** as a white solid.

Synthesis of bis(2,4,6-triisopropylphenyl)disulfide (TRIP disulfide)

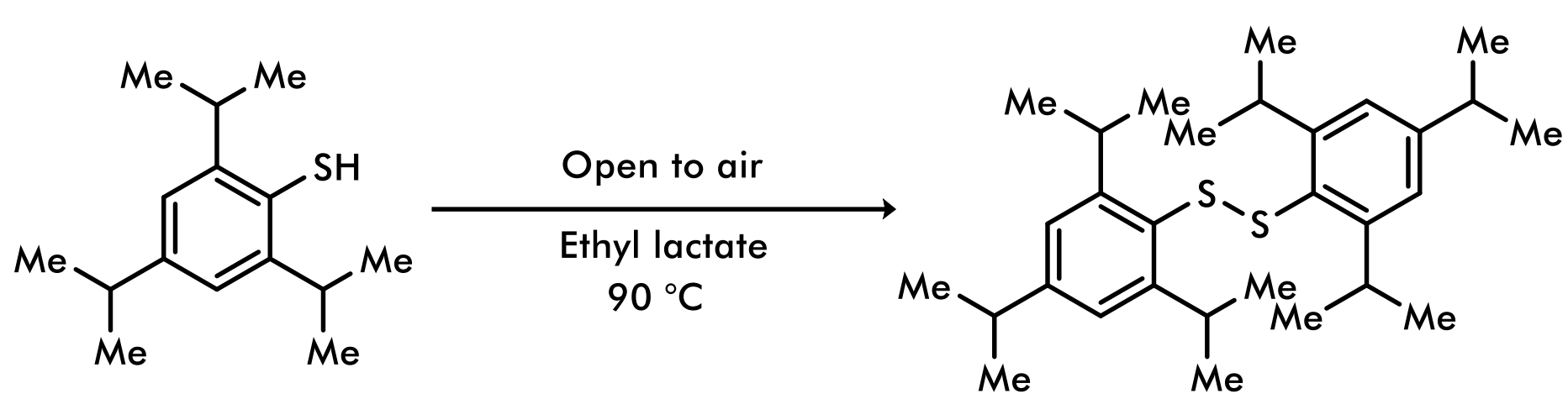

TRIP disulfide is commercially available reagent that could be purchased from Ambeed and Oakwood Chemical. If needed, it is able to be easily prepared via the revised procedure in literature from 2,4,6-triisopropylthiophenol (TRIP thiol) by stirring under air in ethyl lactate at 90 °C.[4] The reaction was monitored with TLC (Hex). Once the starting TRIP thiol was totally consumed, the work-up and purification procedure in the literature were followed to obtain the desired TRIP disulfide in 52–73% as a light-yellow solid. (Note: either vendor-purchased or bench-made TRIP disulfide had been tested on model substrate **A2** in the reaction and lead to equivalent results.)

General procedure A

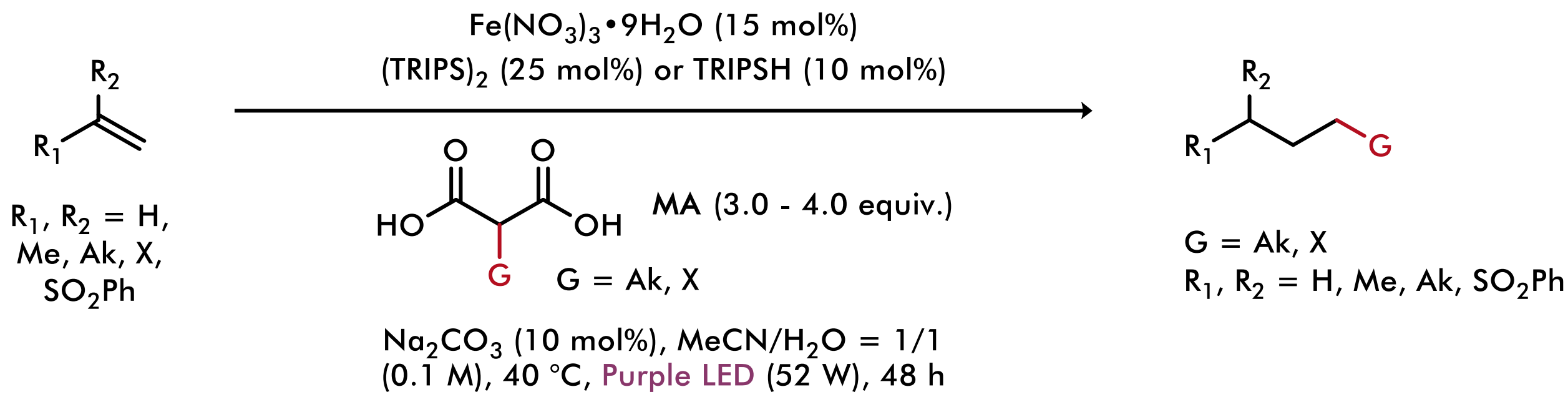

To an oven-dried 16 mL borosilicate glass tube (Fisherbrand™ catalog Number #14-959-35B) containing a Teflon®-coated magnetic stir bar was added $Fe(NO_3)_3 \cdot 9H_2O$ (6.0 mg, 0.015 mmol, 15 mol%), Na_2CO_3 (1.1 mg, 0.01 mmol, 10 mol%), bis(2,4,6-triisopropylphenyl)disulfide (11.4 mg, 0.025 mmol, 25 mol%) or 2,4,6-Triisopropylthiophenol (2.5 μL, 0.01 mmol, 10 mol%), malonic acid (0.3–0.4 mmol, 3.0–4.0 equiv.), and alkene (0.1 mmol, 1.0 equiv.) (added at the beginning if solid). The tube was sealed on the top with a phenolic screw cap (Kimble® 73804-15425) equipped with white PTFE septa (VWR Supplier No. B7995-15, VWR Catalog Number #66030-760) and the vial was evacuated and backfilled with N_2 (repeated for 4 times). MeCN (0.5 mL) was added into the tube, followed by alkene (0.1 mmol, 1.0 equiv.) (added at this stage if liquid), and water (0.5 mL) via syringe under N_2. After addition, the punctured holes of the tube cap was sealed with high-vacuum grease and electric tape/parafilm for better air-tight protection, the reaction mixture was prestirred for 15 mins then placed under 390 nm Kessil® lamp (52 W, 100% intensity) equipped with a cooling fan (or reaction mixture was placed in a pre-heated water bath at 40 °C with temperature probe, Heidolph™ Catalog Number #13-876-206, if necessary) on a magnetic stirrer hotplate (700 rpm) to react for 48 hours. Following this, the solvent was removed with a rotary evaporator under reduced pressure, and the residue was filtered through a microscale (pipette) column (5 cm) which was subsequently rinsed with DCM (note: EtOAc was used on the screening of reaction conditions to obtain the precise yield of carboxylic acid intermediate.) or pentane (note: for volatile product). The filtrate was concentrated, and the residue was then purified by preparative thin layer or flash column chromatography to give the corresponding hydroalkylated product.

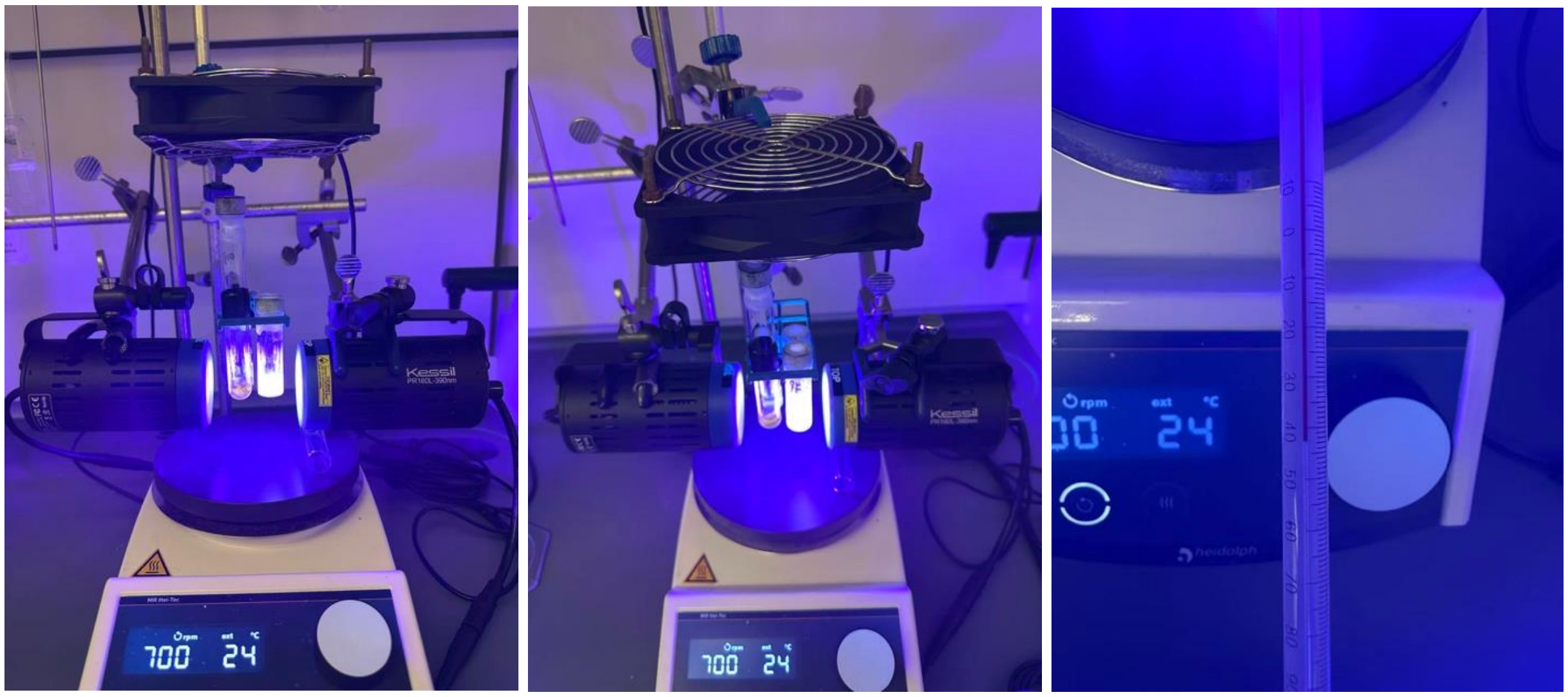

A **B** **C**

Supplementary Figure 3. Experimental setup for general procedure A. (a) The distance between the centers of two Kessil LEDs is 4 cm, while the center of the lamp is 1cm away from the closet side of the reaction vial. Both lamps are set to 52W (100% intensity) for the irradiation. (b) The 4V-12V cooling fan is set 14 cm above the vial holder, which is set to 8V for the constant cooling power. (c) The temperature of the geometric center between the lamps is detected at about 40 °C constantly. The rpm of the stirring plate is set to 700.

General procedure B

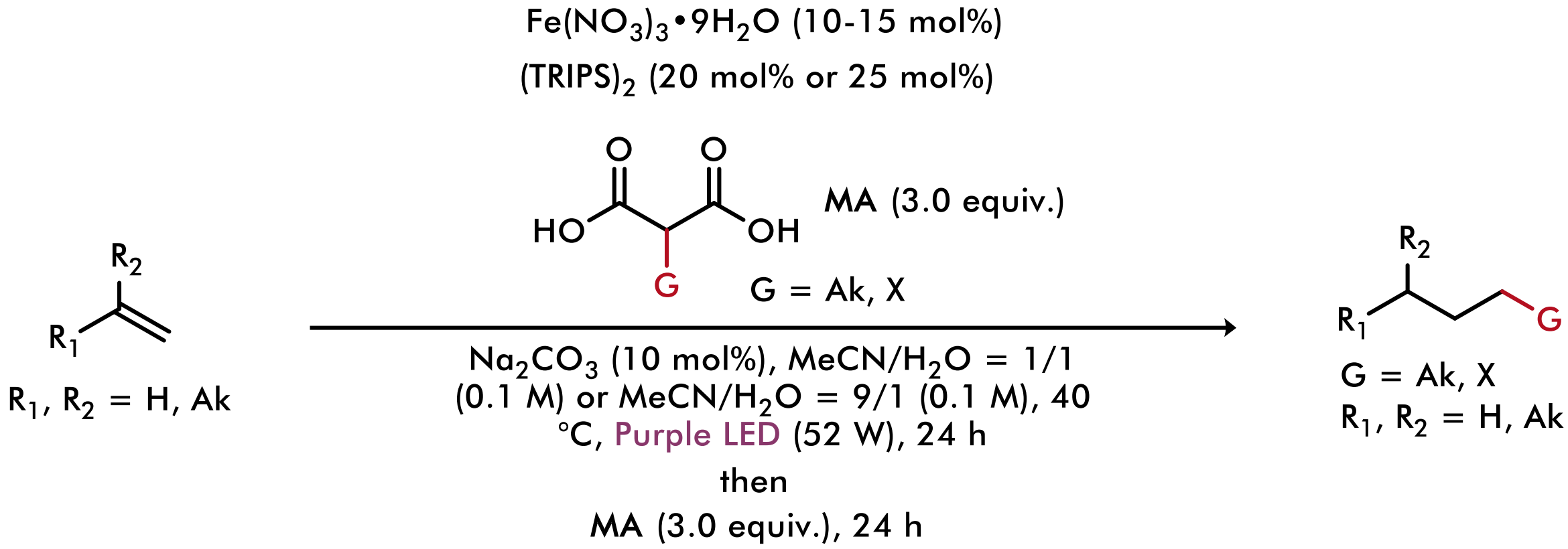

To an oven-dried 16 mL borosilicate glass tube (Fisherbrand™ catalog Number #14-959-35B) containing a Teflon®-coated magnetic stir bar was added $Fe(NO_3)_3 \cdot 9H_2O$ (4.0 mg, 0.01 mmol, 10 mol% or 6.0 mg, 0.015 mmol, 15 mol%), Na_2CO_3 (1.1 mg, 0.01 mmol, 10 mol%), bis(2,4,6-triisopropylphenyl)disulfide (9.4 mg, 0.02 mmol, 20 mol% or 11.4 mg, 0.025 mmol, 25 mol%), malonic acid (0.3 mmol, 3.0 equiv.), and alkene (0.1 mmol, 1.0 equiv.) (added at the beginning if solid). The tube was then sealed on the top with a phenolic screw cap (Kimble® 73804-15425) equipped with white PTFE septa (VWR Supplier No. B7995-15, VWR Catalog Number #66030-760) was evacuated and backfilled with N_2 (repeated for 4 times). MeCN (0.5 or 0.9 mL) was added into the tube, followed by alkene (0.1, 1.0 equiv.) (added at this stage if liquid), and water (0.5 or 0.1 mL) via syringe under N_2. After addition, the punctured holes of the tube cap were sealed with high-vacuum grease and electric tape/parafilm for better air-tight protection and the reaction mixture was prestirred for 15 mins then placed under 390 nm Kessil® lamp (52W, 100% intensity) equipped with a cooling fan (or reaction mixture was placed in a pre-heated water bath at 40 °C with temperature probe, Heidolph™ Catalog Number #13-876-206, if necessary) on a magnetic stirrer hotplate (700 rpm) to react for 24 hours. Following this, another batch of malonic acid (0.3 mmol, 3.0 equiv.) was added to the reaction mixture, followed by purging with nitrogen balloon for one minute. The tube was then left to react for another 24 hours under the setup aforementioned in general procedure A before work up by following the general procedure A.

*Synthetic procedure for the hydromethylation of **A9***

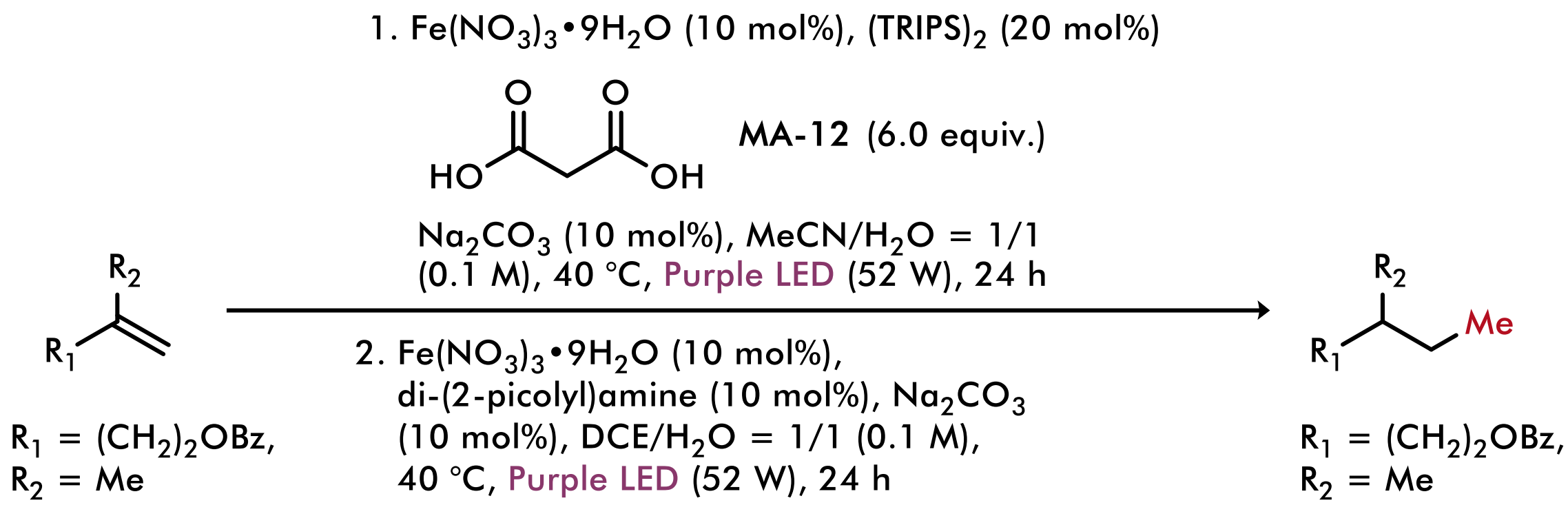

To an oven-dried 16 mL borosilicate glass tube (Fisherbrand™ catalog Number #14-959-35B) containing a Teflon®-coated magnetic stir bar was added $Fe(NO_3)_3 \cdot 9H_2O$ (4.0 mg, 0.01 mmol, 10 mol%), Na_2CO_3 (0.01 mmol, 10 mol%), bis(2,4,6-triisopropylphenyl)disulfide (9.4 mg, 0.02 mmol, 20 mol%), malonic acid **MA-12** (62 mg, 0.6 mmol, 6.0 equiv.). The tube was then sealed on the top with a phenolic screw cap (Kimble® 73804-15425) equipped with white PTFE septa (VWR Supplier No. B7995-15, VWR Catalog Number #66030-760) and evacuated and backfilled with N_2 (repeated for 4 times). MeCN (0.5 mL) was added into the tube, followed by alkene (0.1 mmol, 1.0 equiv.), and then water (0.5 mL) via syringe under N_2. After addition, the punctured holes of the tube cap were sealed with high-vacuum grease and electric tape/parafilm for better air-tight protection and the reaction mixture was prestirred for 15 mins then placed under 390 nm Kessil® lamp (52 W, 100% intensity) equipped with a cooling fan (or reaction mixture was placed in a pre-heated water bath at 40 °C with temperature probe, Heidolph™ Catalog Number #13-876-206, if necessary) on a magnetic stirrer hotplate (700 rpm) to react for 24 hours. Following this, the solvent and the generated acetic acid were removed with a rotary evaporator under reduced pressure, and the residue was filtered through a microscale (pipette) column (5 cm) of celite which was subsequently rinsed with DCM. The filtrate was concentrated and put under high vacuum pump for a short time to remove the residue of acetic acid. This resulting mixture was then dissolved with dichloroethane (0.5 mL) and transferred into a nitrogen-evacuated and backfilled 16 mL borosilicate glass tube containing $Fe(NO_3)_3 \cdot 9H_2O$ (4.0 mg, 0.01 mmol, 10 mol%), Na_2CO_3 (0.01 mmol, 10 mol%). To this tube was added dipicolylamine (1.8 μL, 0.01 mmol, 10 mol%) and water (0.5 mL). The reaction mixture was left to react for another 24 hours under the

setup aforementioned in general procedure A. The product was then obtained by following the workup procedure in general procedure A.

Synthetic procedure for the hydromethylation of ***A35, A36*&*A37***

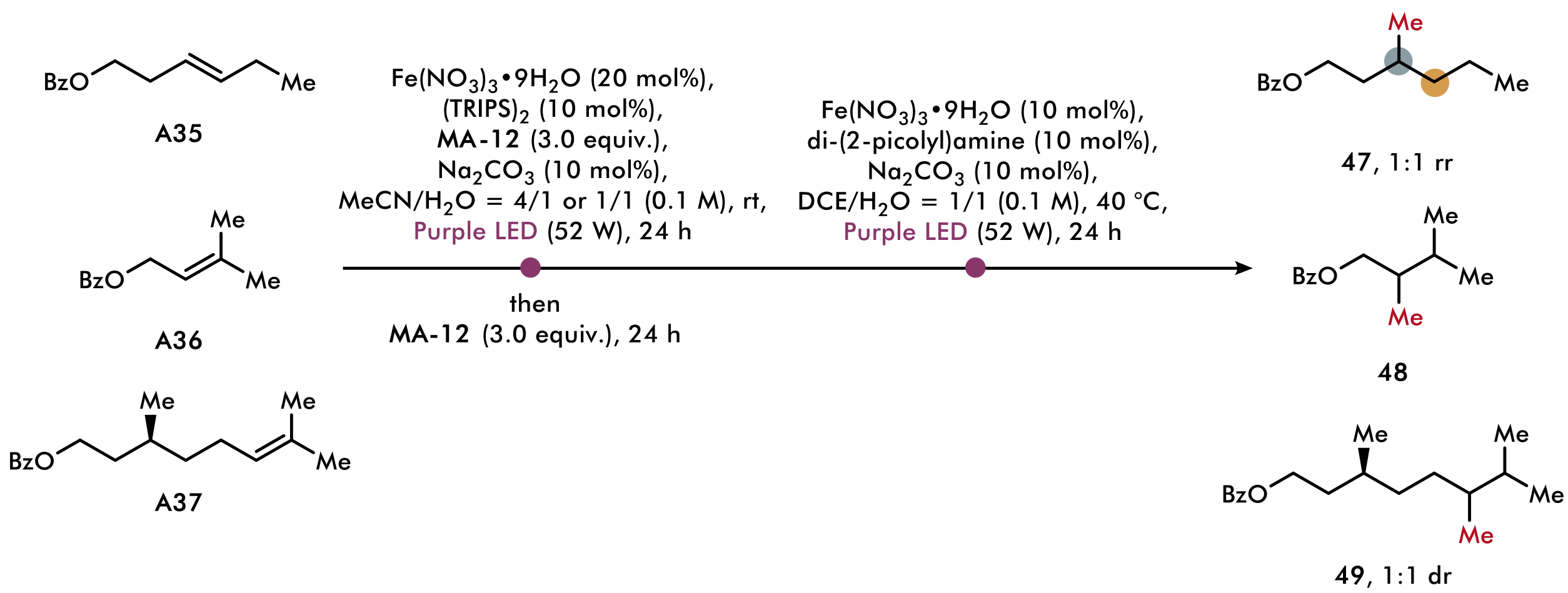

To an oven-dried 16 mL borosilicate glass tube (Fisherbrand™ catalog Number #14-959-35B) containing a Teflon®-coated magnetic stir bar was added $Fe(NO_3)_3 \cdot 9H_2O$ (8.0 mg, 0.02 mmol, 20 mol%), Na_2CO_3 (0.01 mmol, 10 mol%), bis(2,4,6-triisopropylphenyl)disulfide (4.7 mg, 0.01 mmol, 10 mol%), malonic acid **MA-12** (31 mg, 0.3 mmol, 3.0 equiv.). The tube was then sealed on the top with a phenolic screw cap (Kimble® 73804-15425) equipped with white PTFE septa (VWR Supplier No. B7995-15, VWR Catalog Number #66030-760) and evacuated and backfilled with N_2 (repeated for 4 times). MeCN (0.8 mL) or MeCN (0.5 mL) (for disubstituted alkene, **A35**) was added into the tube, followed by alkene (0.1 mmol, 1.0 equiv.), and then water (0.2 mL) or water (0.5 mL) via syringe under N_2. After addition, the punctured holes of the tube cap were sealed with high-vacuum grease and electric tape/parafilm for better air-tight protection and the reaction mixture was prestirred for 15 mins then placed under 390 nm Kessil® lamp (2*52 W, 100% intensity) equipped with a cooling fan on a magnetic stirrer hotplate (700 rpm) to react for 24 hours at room temparature. Following this, another batch of malonic acid (31 mg, 0.3 mmol, 3.0 equiv.) was added to the reaction mixture, followed by purging with nitrogen balloon for one minute. The tube was then left to react for another 24 hours. The solvent and the generated acetic

acid were removed with a rotary evaporator under reduced pressure, and the residue was filtered through a microscale (pipette) column (5 cm) of celite which was subsequently rinsed with DCM. The filtrate was concentrated and put under high vacuum pump for a short time to remove the residue of acetic acid. This resulting mixture was then dissolved with dichloroethane (0.5 mL) and transferred into a nitrogen-evacuated and backfilled 16 mL borosilicate glass tube containing $Fe(NO_3)_3 \cdot 9H_2O$ (4.0 mg, 0.01 mmol, 10 mol%), Na_2CO_3 (0.01 mmol, 10 mol%). To this tube was added dipicolylamine (1.8 μL, 0.01 mmol, 10 mol%) and water (0.5 mL). The reaction mixture was left to react for another 24 hours under the setup aforementioned in general procedure A. The product was then obtained by following the workup procedure in general procedure A.

Synthetic procedure for the hydrofluoromethylation of ***A35, A36***

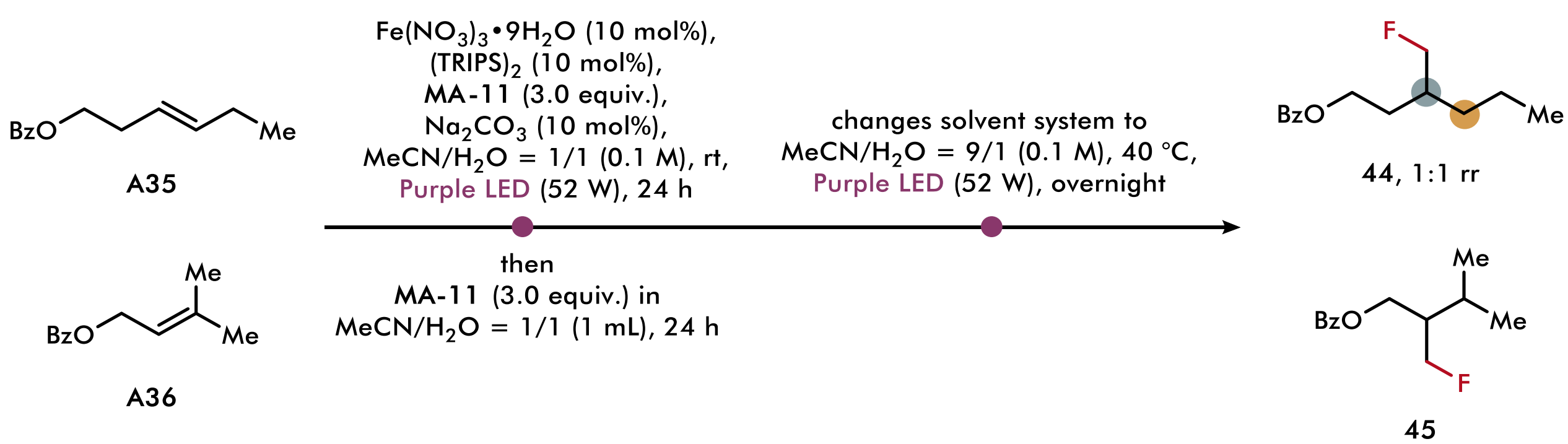

To an oven-dried 16 mL borosilicate glass tube (Fisherbrand™ catalog Number #14-959-35B) containing a Teflon®-coated magnetic stir bar was added $Fe(NO_3)_3 \cdot 9H_2O$ (4.0 mg, 0.01 mmol, 10 mol%), Na_2CO_3 (0.01 mmol, 10 mol%), bis(2,4,6-triisopropylphenyl)disulfide (4.7 mg, 0.01 mmol, 10 mol%), fluoromalonic acid **MA-11** (36 mg, 0.3 mmol, 3.0 equiv.). The tube was then sealed on the top with a phenolic screw cap (Kimble® 73804-15425) equipped with white PTFE septa (VWR Supplier No. B7995-15, VWR Catalog Number #66030-760) and evacuated and backfilled with N_2 (repeated for 4 times). MeCN (0.5 mL) was added into the tube, followed by alkene (0.1 mmol, 1.0 equiv.), and then water (0.5 mL) via syringe under N_2. After addition, the punctured holes of the tube cap were sealed with high-vacuum grease and electric tape/parafilm for better air-tight protection and the reaction mixture was

prestirred for 15 mins then placed under 390 nm Kessil® lamp (52 W, 100% intensity) equipped with a cooling fan on a magnetic stirrer hotplate (700 rpm) to react for 24 hours at room temparature. Following this, another batch of fluoromalonic acid **MA-11** (36 mg, 0.3 mmol, 3.0 equiv.) in MeCN/H_2O (0.5 mL/0.5 mL) was added to the reaction mixture under N_2. The tube was then left to react for another 24 hours. The solvent and the generated acid were removed with a rotary evaporator under reduced pressure. The resulting residue was redissolved with fresh MeCN/H_2O = 9/1 (0.1 M) as solvent in the same tube. The reaction mixture was then left to react overnight under the setup aforementioned in general procedure A. The product was then obtained by following the workup procedure in general procedure A.

*Procedure for the hydroethylation of **A9** on 0.5 gram scale*

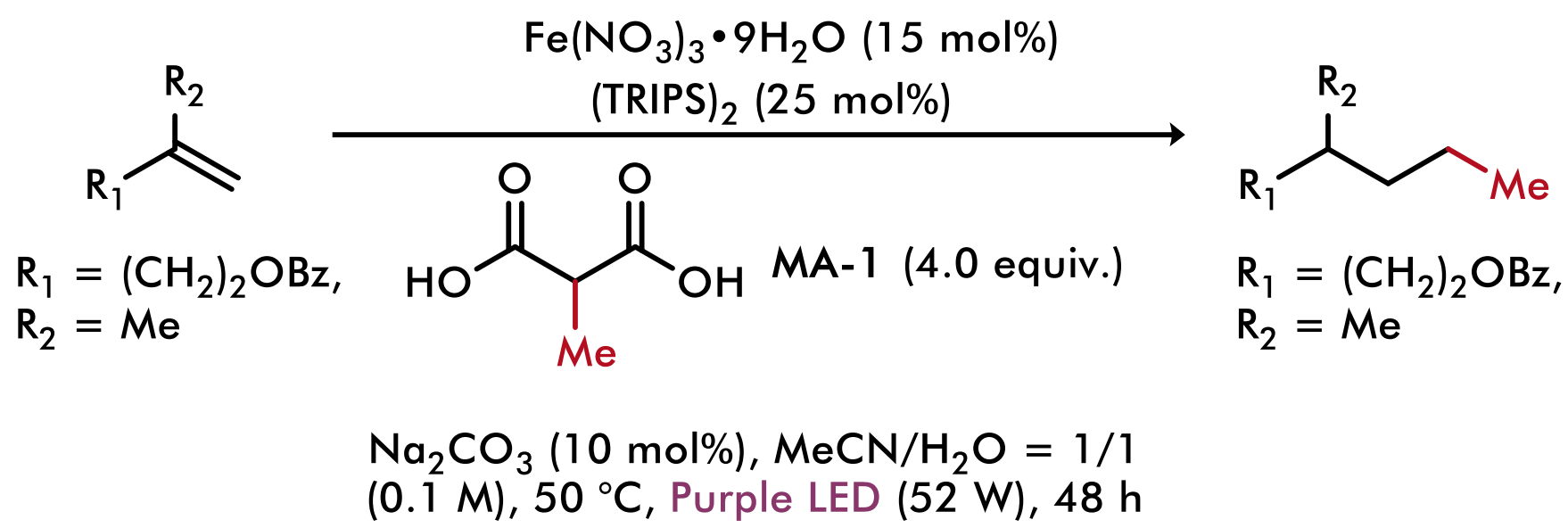

To an oven-dried 100 mL round-bottom flask containing a Teflon®-coated magnetic stir bar was added $Fe(NO_3)_3 \cdot 9H_2O$ (181 mg, 0.45 mmol, 15 mol%), Na_2CO_3 (33 mg, 0.3 mmol, 10 mol%), bis(2,4,6-triisopropylphenyl)disulfide (342 mg, 0.75 mmol, 25 mol%), methylmalonic acid **MA-1** (1.4 g, 12 mmol, 4.0 equiv.). The flask was then sealed on the top with a septum and was evacuated and backfilled with N_2 (repeated for 4 times). MeCN (15 mL) was added into the flask, followed by alkene **A9** (570 mg, 3.0 mmol, 1.0 equiv.), and water (15 mL) via syringe under N_2. After the reaction mixture was prestirred for 15 mins, the flask was removed from the schlenk line and placed under a nitrogen balloon then irradiated using a 390 nm Kessil® lamp (2*52 W, 100% intensity) with 1–2 cm distance between each lamp and the flask, and a cooling fan was used to keep the reaction temperature 50 °C at the outside environment and 60 °C at the inside solution directly measured using a temperature probe. The reaction was performed at such conditions for 48 hours. Following this, the reaction mixture was dilute with EtOAc and Na_2CO_3 (sat.) and separated in a separatory funnel. The organic layer was washed with Na_2CO_3 (sat.) and brine. The collected organic layer was dried over Na_2SO_4, filtered, concentrated with a rotary evaporator under reduced pressure, and the residue was then purified by flash column chromatography to give the corresponding hydroalkylated product (83%, 548.1 mg).

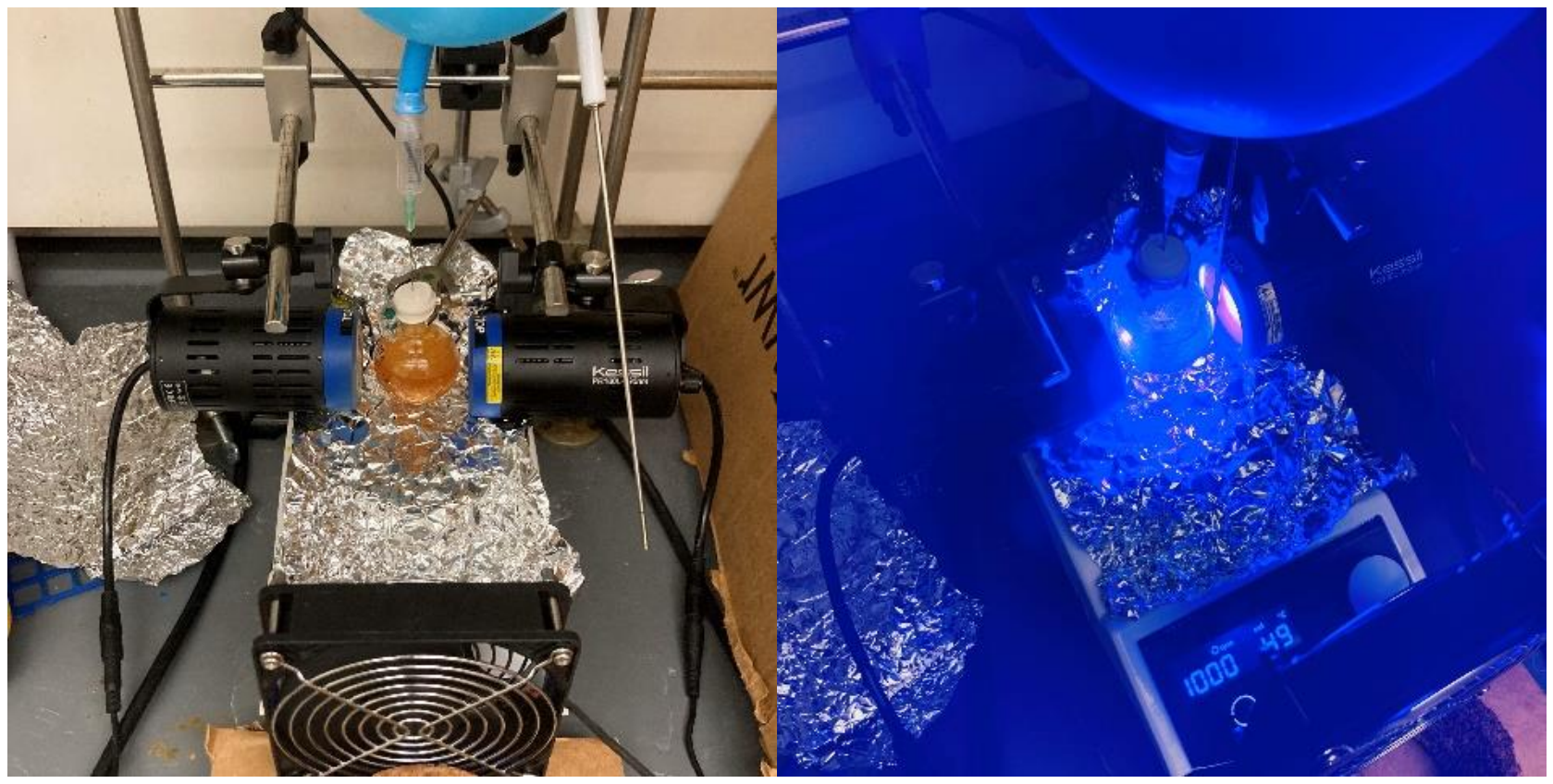

A B

Supplementary Figure 4. Experimental setup for 0.5 gram scale. (a) A mini fan was placed at the front to maintain the reaction temperature. (b) An aluminum foil was placed at the bottom and cover from behind to acquire higher irradiation efficacy.

General procedure C for hydromonofluorocarboxylation of alkenes

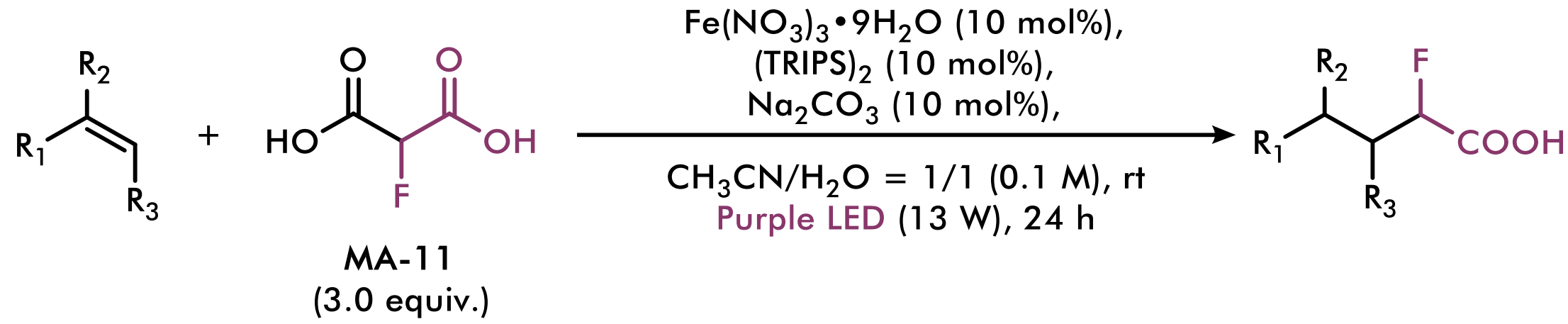

$Fe(NO_3)_3 \cdot 9H_2O$ (4.0 mg, 0.01 mmol, 10 mol%), Na_2CO_3 (1.1 mg, 0.01 mmol, 10 mol%), bis(2,4,6-triisopropylphenyl)disulfide (4.7 mg, 0.01 mmol, 10 mol%), and fluoromalonic acid **MA-11** (36 mg, 0.3 mmol, 3.0 equiv.) were added in an oven-dried 8-mL test vial containing a Teflon®-coated magnetic stir bar. The vial was evacuated and backfilled with N_2 (repeated for 4 times), followed by addition of alkenes (0.1 mmol, 1.0 equiv.), in MeCN/H_2O (1/1, 0.1 M in regard to alkenes) via syringe under N_2. The reaction mixture was placed under 390 nm Kessil® light (13 W) after sealing the punctured holes of the vial cap with vacuum grease and electric tape/parafilm for better air-tight protection and allowed to react at room temperature for 24 h. Following this, the reaction mixture was filtered through a pad of celite which was subsequently rinsed with EA. The filtrate was concentrated, and the residue was then purified by flash column chromatography or preparatory thin-layer chromatography to give the corresponding hydromonofluorocarboxylation products.

General procedure D for hydrodifluorocarboxylation of alkenes

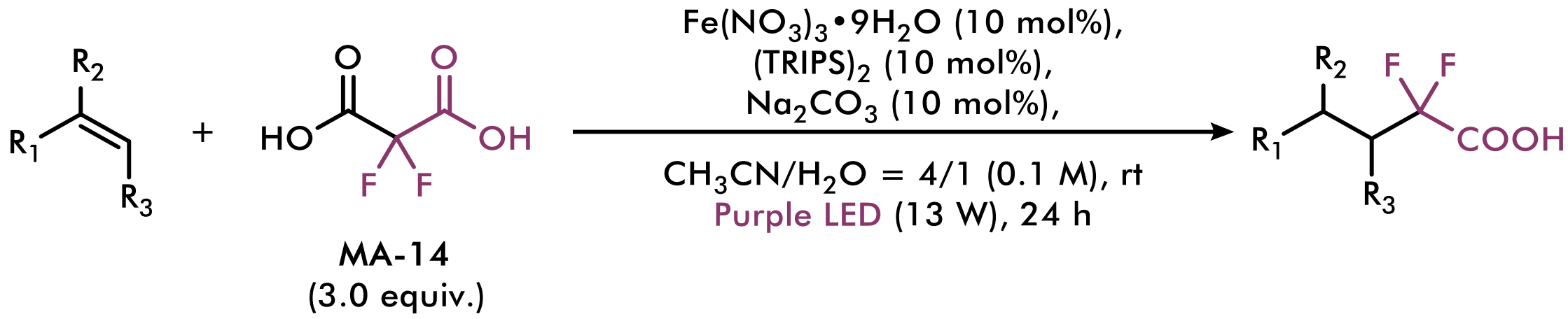

$Fe(NO_3)_3 \cdot 9H_2O$ (4.0 mg, 0.01 mmol, 10 mol%), Na_2CO_3 (1.1 mg, 0.01 mmol, 10 mol%), bis(2,4,6-triisopropylphenyl)disulfide (4.7 mg, 0.01 mmol, 10 mol%), and fluoromalonic acid **MA-11** (36 mg, 0.3 mmol, 3.0 equiv.) were added in an oven-dried 8-mL test vial containing a Teflon®-coated magnetic stir bar. The vial was evacuated and backfilled with N_2 (repeated for 4 times), followed by addition of alkenes (0.1 mmol, 1.0 equiv.), in MeCN/H_2O (4/1, 0.1 M in regard to alkenes) via syringe under N_2. The reaction mixture was placed under 390 nm Kessil® light (13 W) after sealing the punctured holes of the vial cap with vacuum grease and electric tape/parafilm for better air-tight protection and allowed to react at room temperature for 24 h. Following this, the reaction mixture was filtered through a pad of celite which was subsequently rinsed with EA. The filtrate was concentrated, and the residue was then purified by flash column chromatography or preparatory thin-layer chromatography to give the corresponding hydromonofluorocarboxylation products.

General procedure E for hydroalkylcarboxylation of alkenes

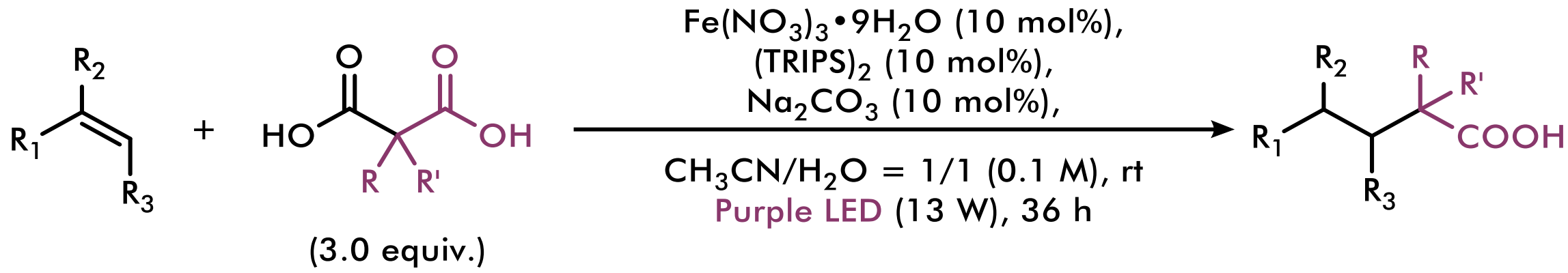

$Fe(NO_3)_3 \cdot 9H_2O$ (4.0 mg, 0.01 mmol, 10 mol%), Na_2CO_3 (1.1 mg, 0.01 mmol, 10 mol%), bis(2,4,6-triisopropylphenyl)disulfide (4.7 mg, 0.01 mmol, 10 mol%), and fluoromalonic acid **MA-11** (36 mg, 0.3 mmol, 3.0 equiv.) were added in an oven-dried 8-mL test vial containing a Teflon®-coated magnetic stir bar. The vial was evacuated and backfilled with N_2 (repeated for 4 times), followed by addition of alkenes (0.1 mmol, 1.0 equiv.), in MeCN/H_2O (1/1, 0.1 M in regard to alkenes) via syringe under N_2. The reaction mixture was placed under 390 nm Kessil® light (13 W) after sealing the punctured holes of the vial cap with vacuum grease and electric tape/parafilm for better air-tight protection and allowed to react at room temperature for 36 h. Following this, the reaction mixture was filtered through a pad of celite which was subsequently rinsed with EA. The filtrate was concentrated, and the residue was then purified by flash column chromatography or preparatory thin-layer chromatography to give the corresponding hydromonofluorocarboxylation products.

Procedure for decarboxylative deuteration of monofluorocarboxylic acid

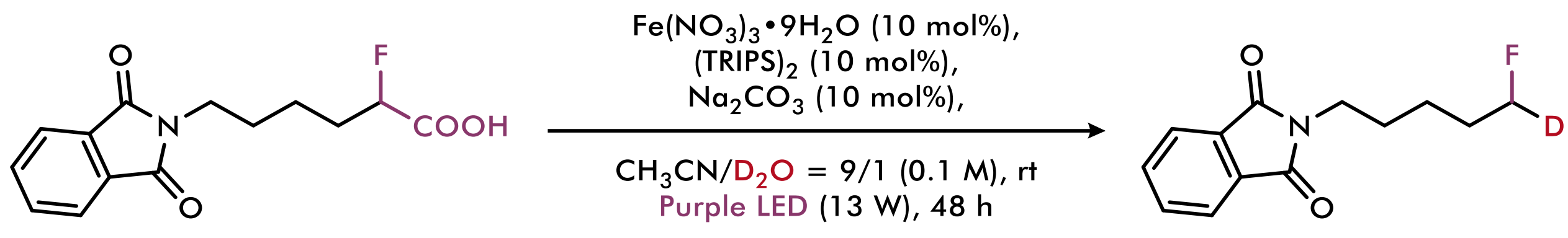

57 **77**

$Fe(NO_3)_3 \cdot 9H_2O$ (4.0 mg, 0.01 mmol, 10 mol%), bis(2,4,6-triisopropylphenyl)disulfide (4.7 mg, 0.01 mmol, 10 mol%), carboxylic acid **57** (27.9 mg, 0.1 mmol, 1.0 equiv), and Na_2CO_3 (1.1 mg, 0.01 mmol, 10 mol%) were added in an oven-dried 8-mL test vial containing a Teflon®-coated magnetic stir bar. The vial was evacuated and backfilled with N_2 (repeated for 4 times), followed by addition of MeCN/D_2O (9/1, 0.1 M in regard to carboxylic acid) via syringe under N_2. The reaction mixture was placed under 390nm Kessil® light (13 W) after sealing the punctured holes of the vial cap with vacuum grease and electric tape/parafilm for better air-tight protection and allowed to react at room temperature for 48 h. Following this, the reaction mixture was filtered through a pad of celite which was subsequently rinsed with DCM. The filtrate was concentrated, and the residue was then purified by preparatory thin-layer chromatography to give the corresponding decarboxylative deuteration product **77**.

Procedure for decarboxylative halogenation of monofluorocarboxylic acid

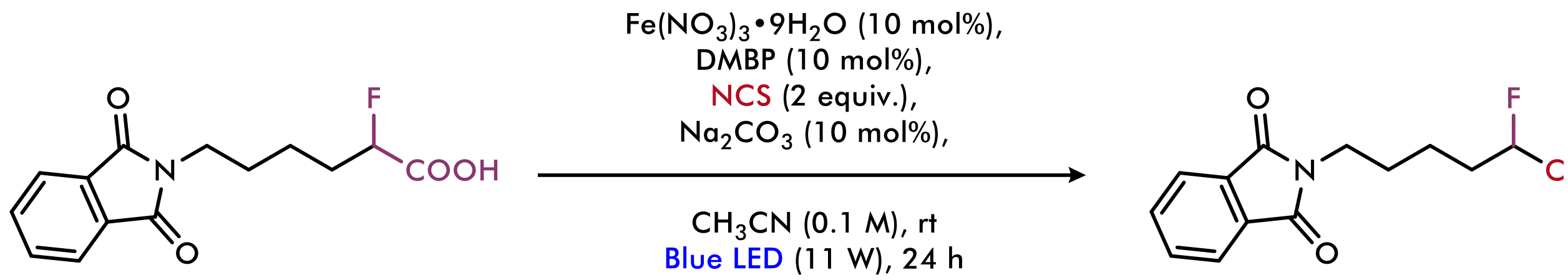

57 **78**, X = Cl

$Fe(NO_3)_3 \cdot 9H_2O$ (4.0 mg, 0.01 mmol, 10 mol%), 4,4'-Dimethoxy-2,2'-bipyridine (DMBP) (2.2 mg, 0.01 mmol, 10 mol%), carboxylic acid **57** (27.9 mg, 0.1 mmol, 1.0 equiv), Na_2CO_3 (1.1 mg, 0.01 mmol, 10 mol%), and N-chlorosuccinimide (NCS) (26.6 mg, 0.2 mmol, 2 equiv.) were added in an oven-dried 8-mL test vial containing a Teflon®-coated magnetic stir bar. The vial was evacuated and backfilled with N_2 (repeated for 4 times), followed by addition of MeCN (0.1 M in regard to carboxylic acid) via syringe under N_2. The reaction mixture was placed under 427nm Kessil® light (11 W) after sealing the punctured holes of the vial cap with vacuum grease and electric tape/parafilm for better air-tight protection and allowed to react at room temperature for 24 hours. Following this, the reaction mixture was filtered through a pad of celite which was subsequently rinsed with DCM. The filtrate was concentrated, and the residue was then purified by preparatory thin-layer chromatography to give the corresponding decarboxylative chlorination product.

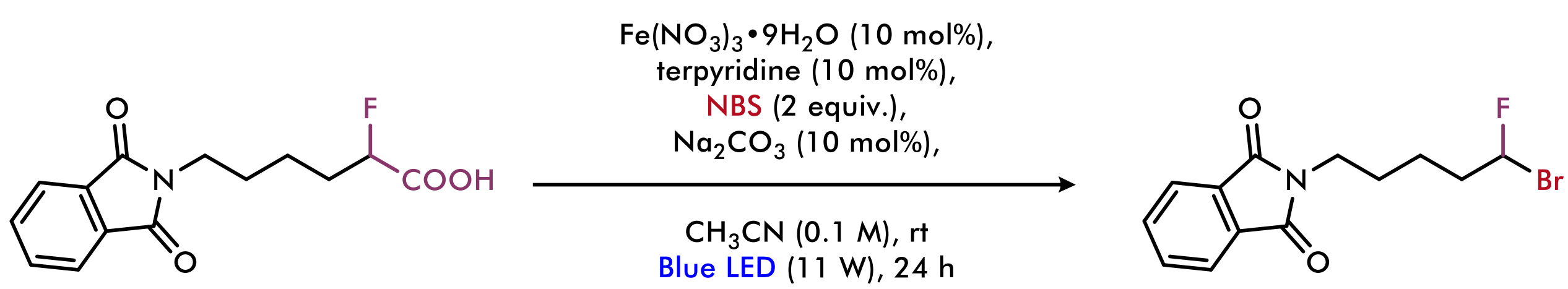

57 **78'**, X = Br

$Fe(NO_3)_3 \cdot 9H_2O$ (4.0 mg, 0.01 mmol, 10 mol%), terpyridine (2.3 mg, 0.01 mmol, 10 mol%), carboxylic acid **57** (27.9 mg, 0.1 mmol, 1.0 equiv), Na_2CO_3 (1.1 mg, 0.01 mmol, 10 mol%), and N-bromosuccinimide (NBS) (35.4 mg, 0.2 mmol, 2 equiv.) were added in an oven-dried 8-mL test vial containing a Teflon®-coated magnetic stir bar. The vial was evacuated and backfilled with N_2 (repeated for 4 times), followed by addition of MeCN (0.1 M in regard to carboxylic acid) via syringe under N_2. The reaction mixture was placed under 427nm Kessil® light (11

W) after sealing the punctured holes of the vial cap with vacuum grease and electric tape/parafilm for better air-tight protection and allowed to react at room temperature for 24 hours. Following this, the reaction mixture was filtered through a pad of celite which was subsequently rinsed with DCM. The filtrate was concentrated, and the residue was then purified by preparatory thin-layer chromatography to give the corresponding decarboxylative bromination product.

Procedure for decarboxylative monofluoroalkylation

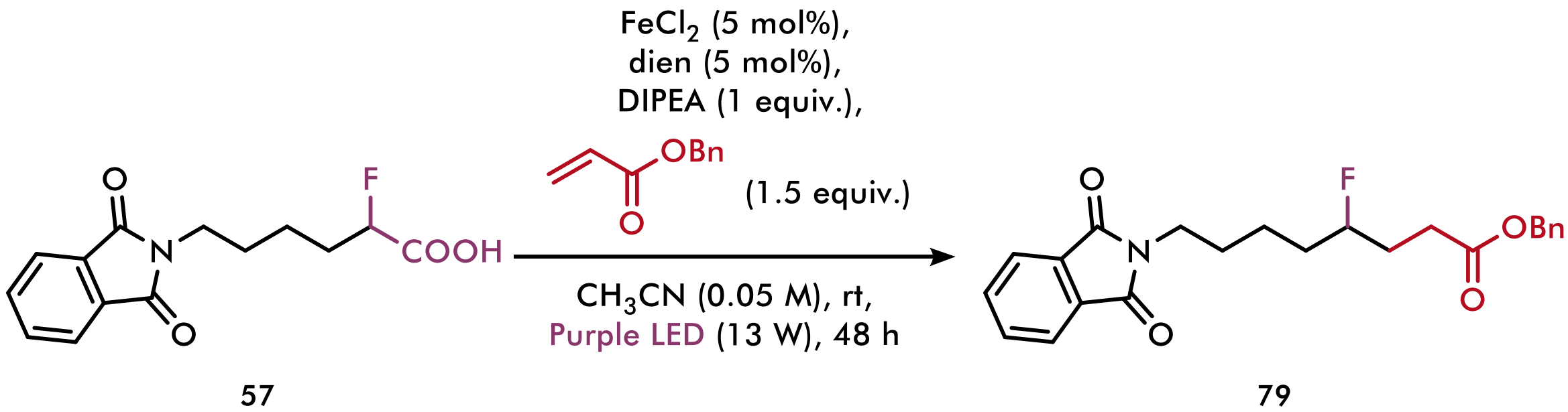

$FeCl_2$ (0.65 mg, 0.005 mmol, 5 mol%), diethylenetriamine (dien) (0.5 µL, 0.005 mmol, 5 mol%), carboxylic acid **57** (27.9 mg, 0.1 mmol, 1.0 equiv), N,N-Diisopropylethylamine (DIPEA) (17.4 µL, 0.1 mmol, 1.0 equiv.), and Benzyl acrylate (23 µL, 0.15 mmol, 1.5 equiv.) were added in an oven-dried 8-mL test vial containing a Teflon®-coated magnetic stir bar. The vial was evacuated and backfilled with N_2 (repeated for 4 times), followed by addition of MeCN (0.05 M in regard to carboxylic acid) via syringe under N_2. The reaction mixture was placed under 390nm Kessil® light (13 W) after sealing the punctured holes of the vial cap with vacuum grease and electric tape/parafilm for better air-tight protection and allowed to react at room temperature for 48 h. Following this, the reaction mixture was filtered through a pad of celite which was subsequently rinsed with DCM. The filtrate was concentrated, and the residue was then purified by preparatory thin-layer chromatography to give the corresponding decarboxylative monofluoroalkylation product.

Procedure for decarboxylative halogenation of difluorocarboxylic acid

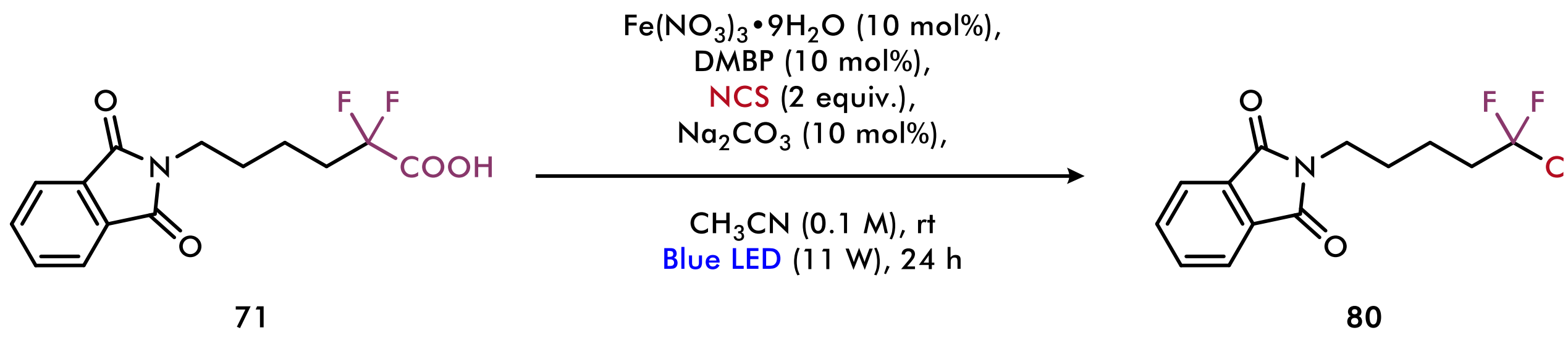

$Fe(NO_3)_3 \cdot 9H_2O$ (4.0 mg, 0.01 mmol, 10 mol%), 4,4'-Dimethoxy-2,2'-bipyridine (DMBP) (2.2 mg, 0.01 mmol, 10 mol%), carboxylic acid **71** (28.3 mg, 0.1 mmol, 1.0 equiv), Na_2CO_3 (1.1 mg, 0.01 mmol, 10 mol%), and N-chlorosuccinimide (NCS) (26.6 mg, 0.2 mmol, 2 equiv.) were added in an oven-dried 8-mL test vial containing a Teflon®-coated magnetic stir bar. The vial was evacuated and backfilled with N_2 (repeated for 4 times), followed by addition of MeCN (0.1 M in regard to carboxylic acid) via syringe under N_2. The reaction mixture was placed under 427nm Kessil® light (11 W) after sealing the punctured holes of the vial cap with vacuum grease and electric tape/parafilm for better air-tight protection and allowed to react at room temperature for 24 hours. Following this, the reaction mixture was filtered through a pad of celite which was subsequently rinsed with DCM. The filtrate was concentrated, and the residue was then purified by preparatory thin-layer chromatography to give the corresponding decarboxylative chlorination product.

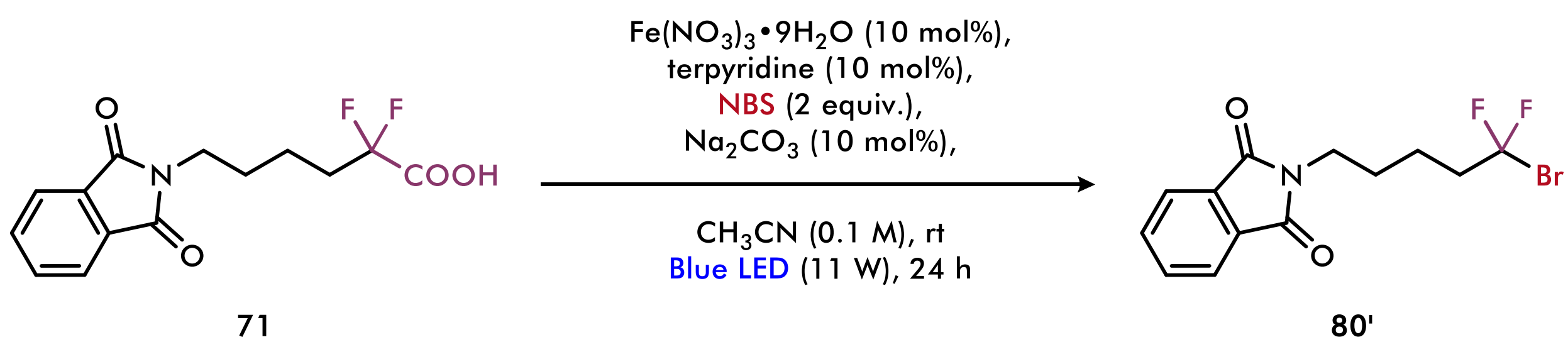

$Fe(NO_3)_3 \cdot 9H_2O$ (4.0 mg, 0.01 mmol, 10 mol%), terpyridine (2.3 mg, 0.01 mmol, 10 mol%), carboxylic acid **71** (28.3 mg, 0.1 mmol, 1.0 equiv), Na_2CO_3 (1.1 mg, 0.01 mmol, 10 mol%), and N-bromosuccinimide (NBS) (35.4 mg, 0.2 mmol, 2 equiv.) were added in an oven-dried 8-mL test vial containing a Teflon®-coated magnetic stir bar. The vial was evacuated and backfilled with N_2 (repeated for 4 times), followed by addition of MeCN (0.1 M in regard to carboxylic

acid) via syringe under N_2. The reaction mixture was placed under 427nm Kessil® light (11 W) after sealing the punctured holes of the vial cap with vacuum grease and electric tape/parafilm for better air-tight protection and allowed to react at room temperature for 24 hours. Following this, the reaction mixture was filtered through a pad of celite which was subsequently rinsed with DCM. The filtrate was concentrated, and the residue was then purified by preparatory thin-layer chromatography to give the corresponding decarboxylative bromination product.

Procedure for decarboxylative difluoroalkylation

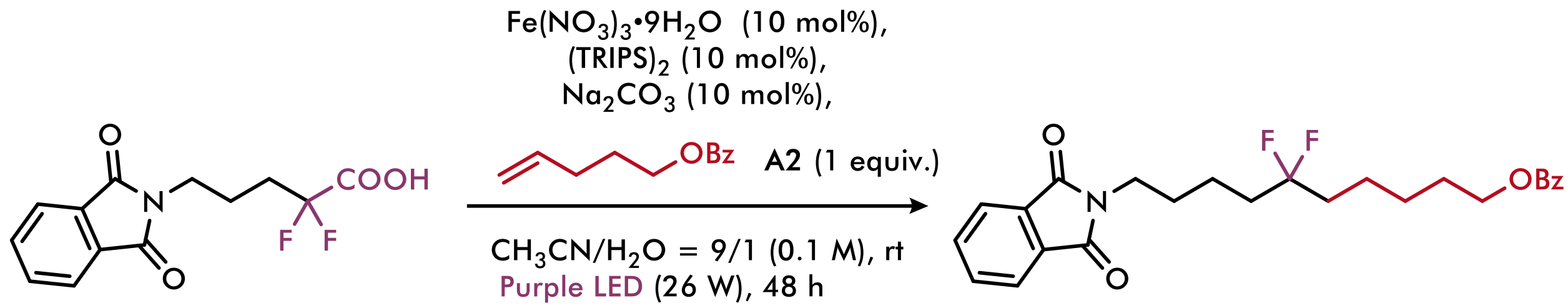

71
(3 equiv.)

81

$Fe(NO_3)_3 \cdot 9H_2O$ (4.0 mg, 0.01 mmol, 10 mol%), bis(2,4,6-triisopropylphenyl)disulfide (4.7 mg, 0.01 mmol, 10 mol%), carboxylic acid **71** (84.9 mg, 0.3 mmol, 3.0 equiv), and Na_2CO_3 (1.1 mg, 0.01 mmol, 10 mol%) were added in an oven-dried 8-mL test vial containing a Teflon®-coated magnetic stir bar. The vial was evacuated and backfilled with N_2 (repeated for 4 times), followed by addition of alkene **A2** (19 mg, 0.1 mmol, 1.0 equiv.) in MeCN/H_2O (9/1, 0.1 M in regard to alkene) via syringe under N_2. The reaction mixture was placed under 390nm Kessil® light (26 W) after sealing the punctured holes of the vial cap with vacuum grease and electric tape/parafilm for better air-tight protection and allowed to react at room temperature for 48 h. Following this, the reaction mixture was filtered through a pad of celite which was subsequently rinsed with DCM. The filtrate was concentrated, and the residue was then purified by preparatory thin-layer chromatography to give the corresponding decarboxylative difluoroalkylation product.

1,6-exo-trig radical-clock experiment

Reaction was performed at 0.1 mmol scale with the setup described in general procedure A. Yield was determined by GC-FID analysis of the crude reaction mixture with cyclododecane as an internal standard.

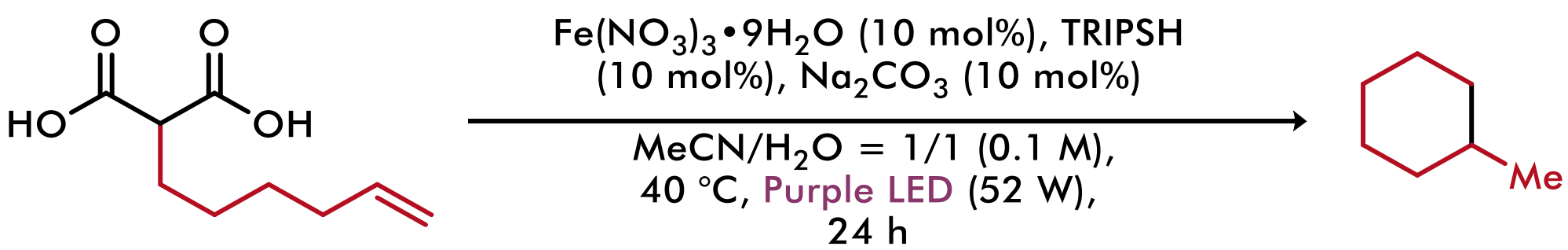

MA-13 (1.0 equiv.) **83**, 29%

Sample Chromatograms

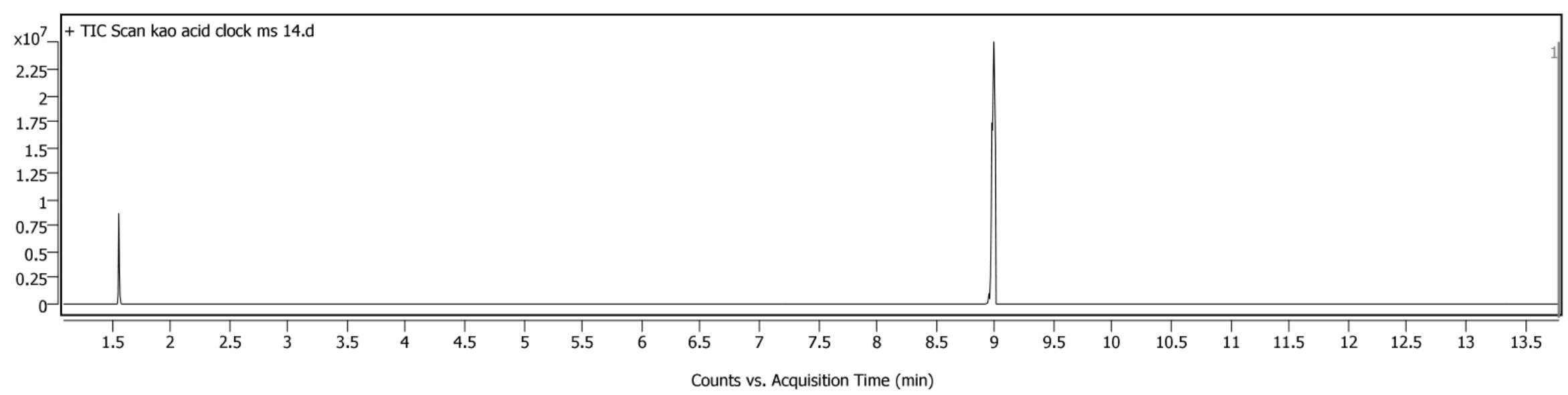

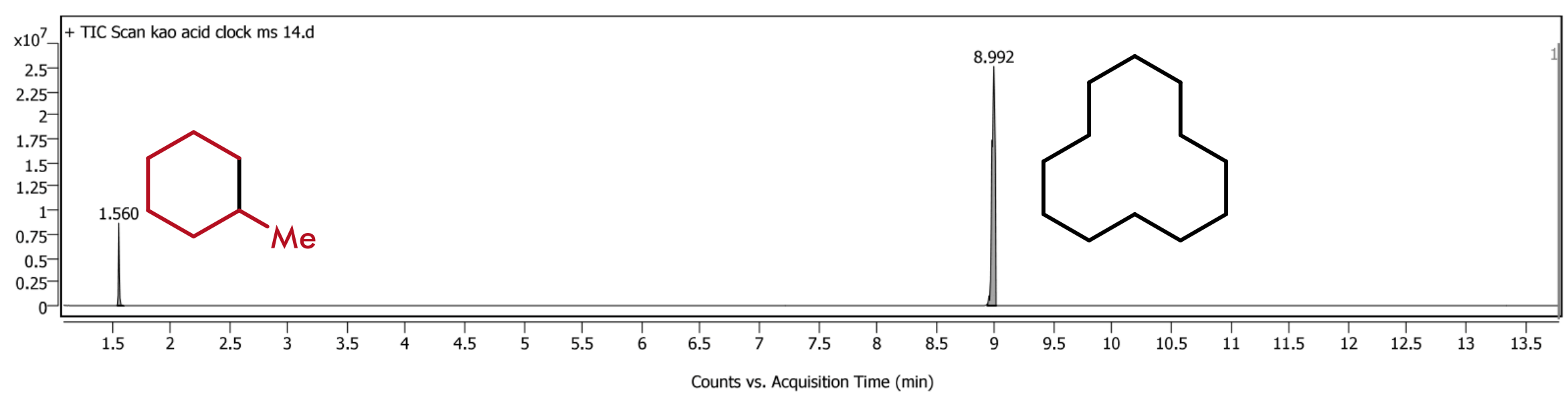

Chromatogram Peaks

Peak	Start	RT	End	Height	Area	Area %	SNR
1	1.542	1.560	1.602	8673349	5026620	10.71	
2	8.938	8.992	9.016	25112034	46938735	100.00	

Sample Spectra

+ Scan (rt: 1.55-1.57 min) **Peak 1 from + TIC Scan**

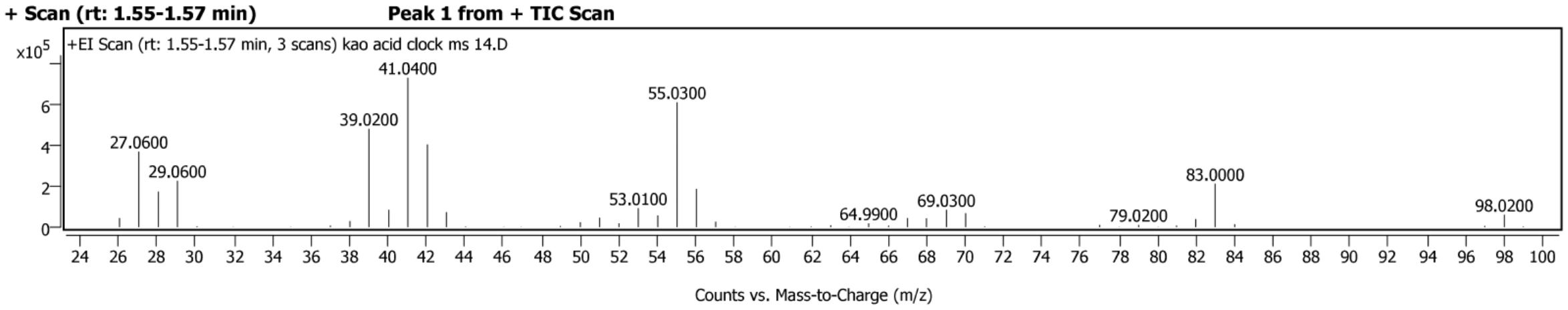

B

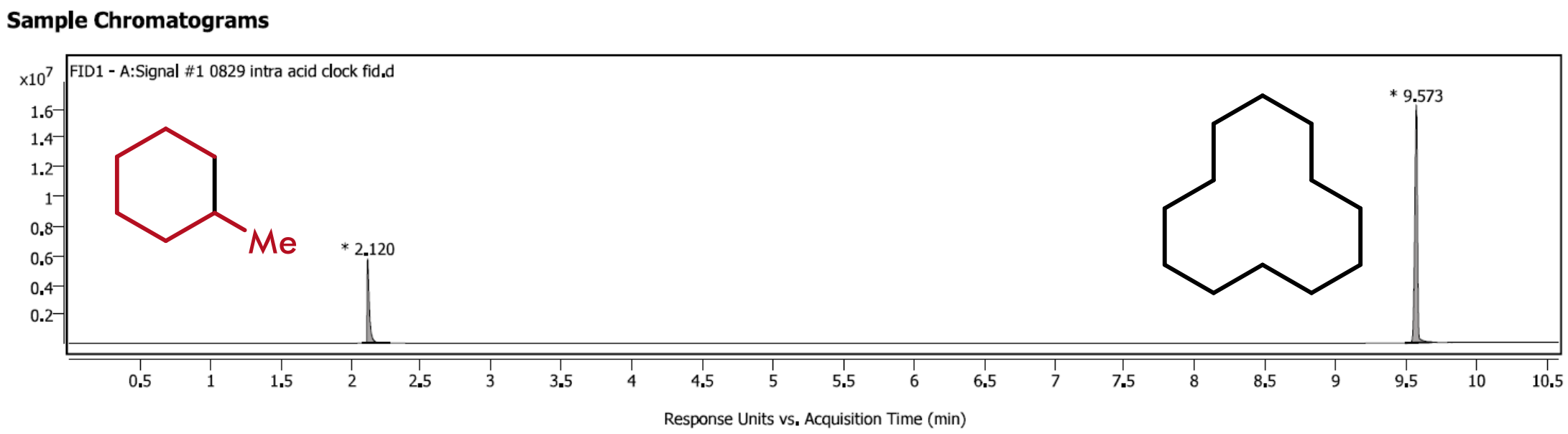

Chromatogram Peaks

Peak	Start	RT	End	Height	Area	Area %	SNR
1	2.084	2.120	2.277	5743703	6407084	29.74	
2	9.491	9.573	9.684	16161187	21543214	100.00	

Supplementary Figure 5. Analyzing the formation of **83** through Gas Chromotography. (a) Tracking the formation of **83** via GC-MS spectroscopy. (b) Yield determination via GC-FID spectroscopy.

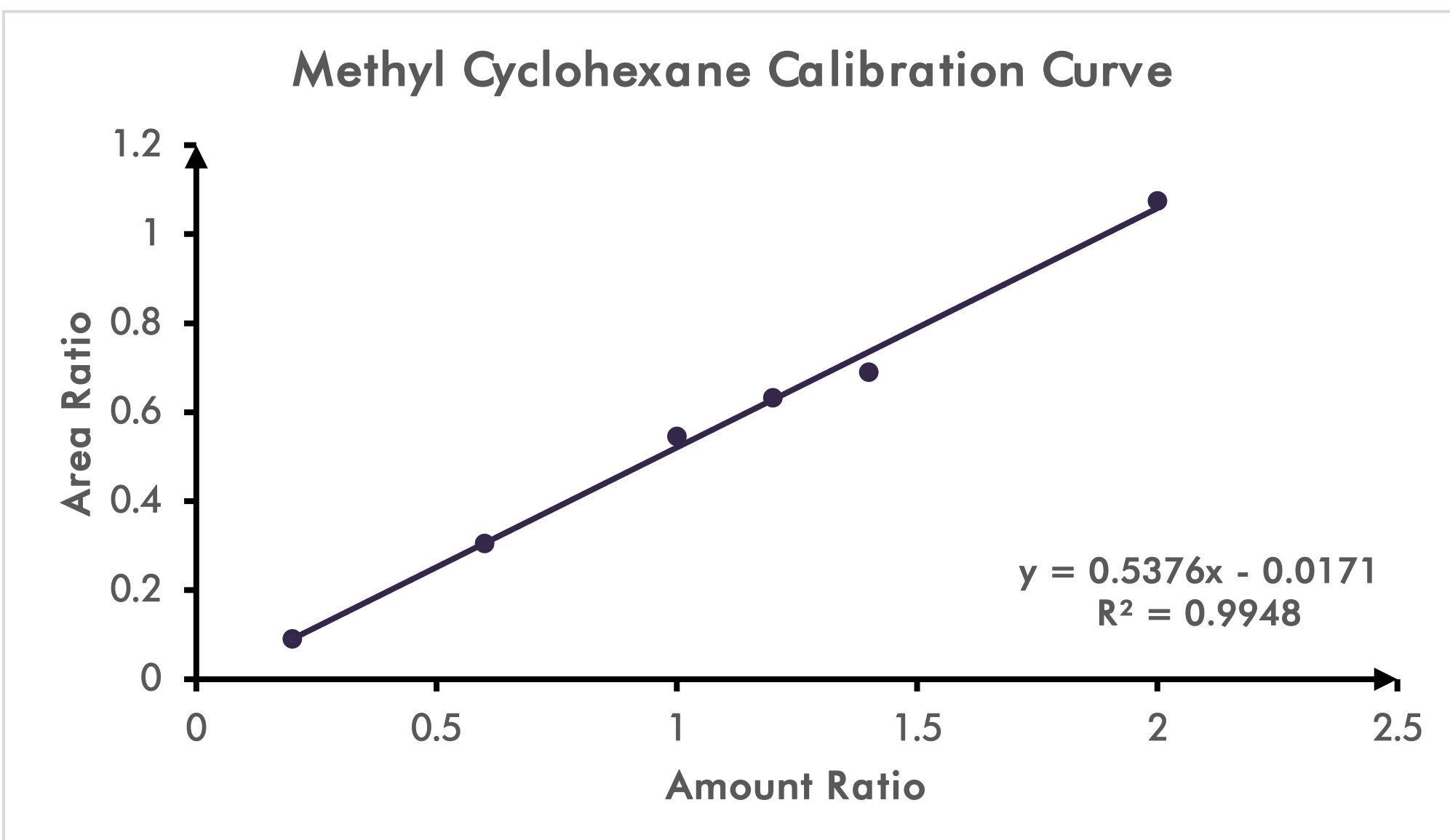

Supplementary Figure 6. GC calibration curve for methyl cyclohexane.

Deuterium-labeling experiment

Reaction was performed at 0.1 mmol scale with the setup described in general procedure A. Yield was isolated yield and the percentage labelings were determined by ^1H NMR analysis of pure spectrum of compound **86**.

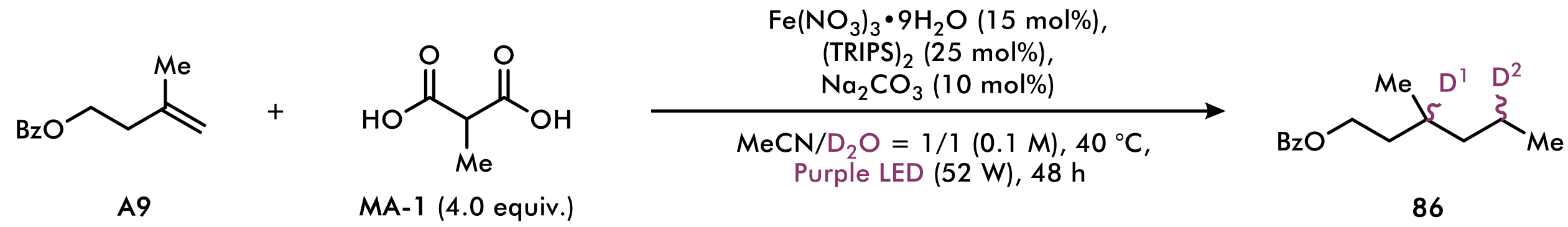

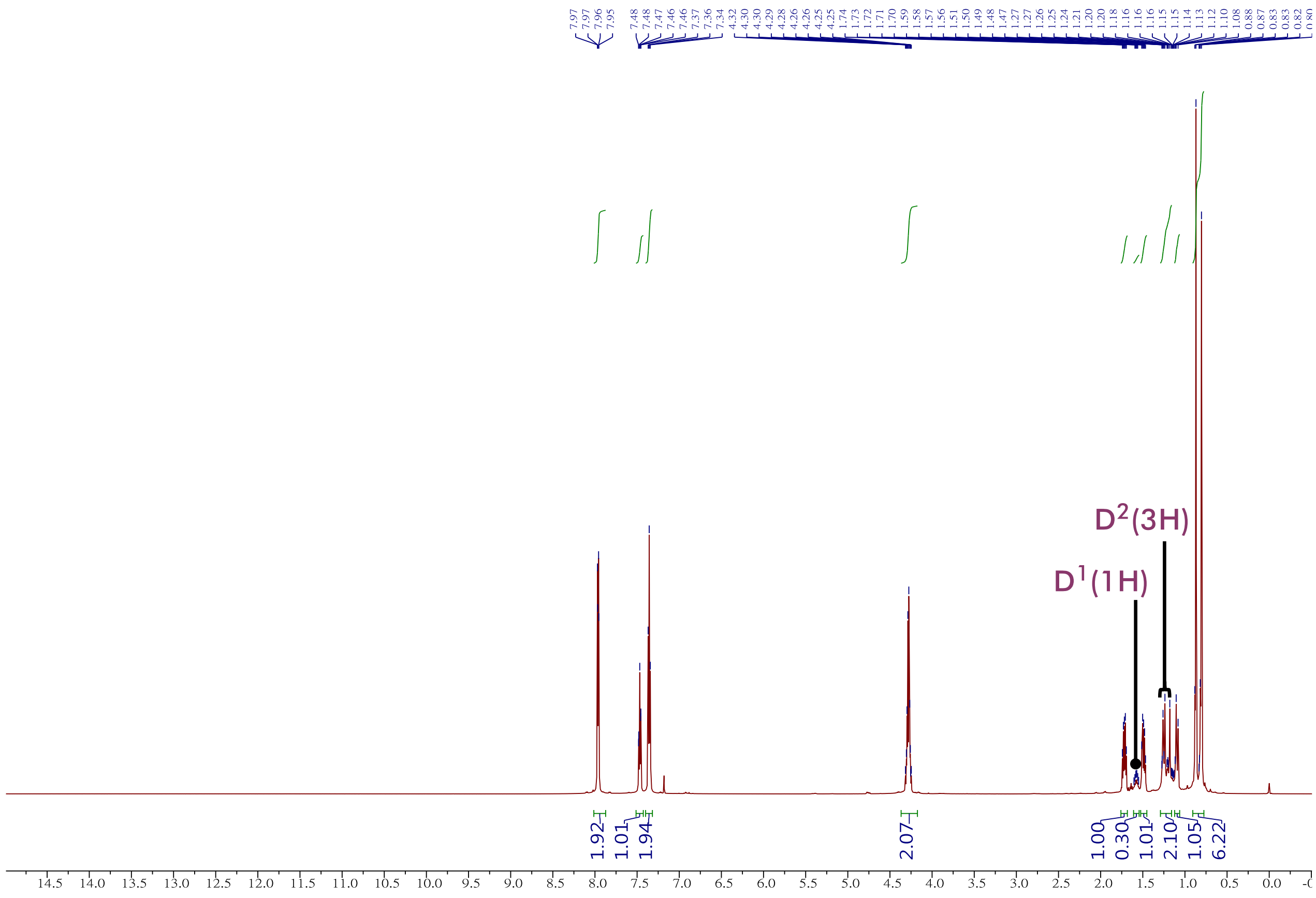

Supplementary Figure 7. ^1H NMR spectrum of pure compound **86**.

Kinetic isotope effect (KIE) experiments

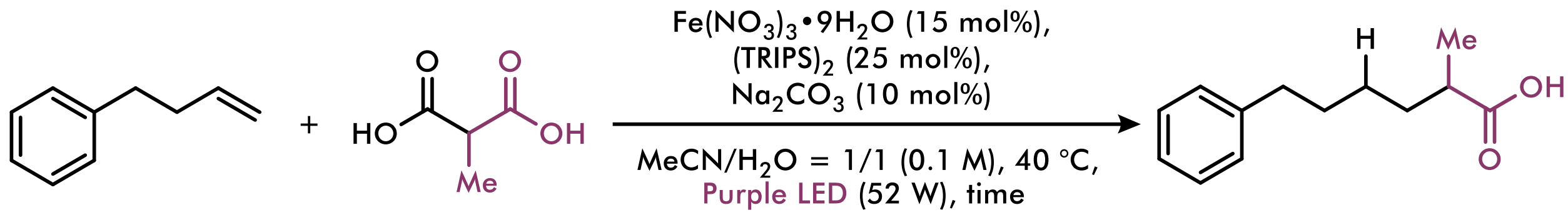

A1 MA-1 (4.0 equiv.) 87

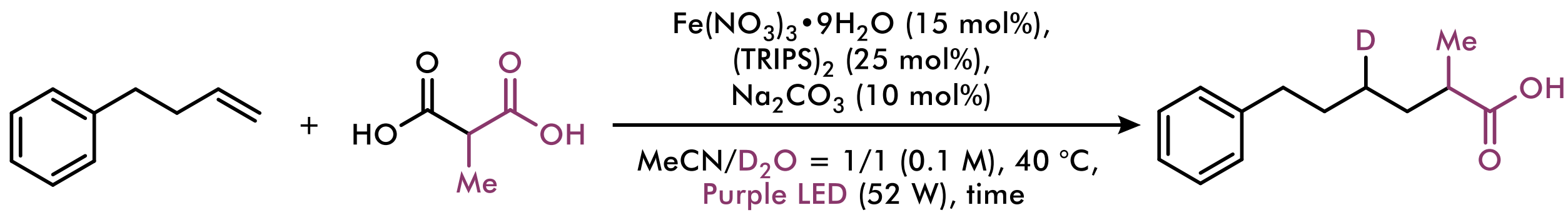

A1 MA-1 (4.0 equiv.) 88

$Fe(NO_3)_3 \cdot 9H_2O$ (12 mg, 0.03 mmol, 15 mol%), bis(2,4,6-triisopropylphenyl)disulfide (22.8 mg, 0.05 mmol, 25 mol%), methylmalonic acid **MA-1** (93 mg, 0.8 mmol, 4.0 equiv.), and Na_2CO_3 (2.2 mg, 0.02 mmol, 10 mol%) were added in an oven-dried 8-mL test vial containing a Teflon®-coated magnetic stir bar. The vial was evacuated and backfilled with N_2 (repeated for 4 times), followed by addition of alkene **A1** (30 μL, 0.2 mmol, 1.0 equiv.) and CH_2Br_2 (internal standard) in $MeCN/H_2O$ or $MeCN/D_2O$ (1/1, 0.1 M in regard to alkene) via syringe under N_2. Following this, the vial cap was attached a nitrogen balloon and sealed with vacuum grease and electric tape/parafilm for better air-tight protection. The reaction mixture was placed under 390nm Kessil® light (52 W) and allowed to react at 40 °C. At different time points, the aliquot of reaction mixture was directly separeated in benzene-d_6 and brine. The organic layer was transferred to NMR tube and reaction progress was monitored by ^{1}H NMR.

Supplementary Table 8. KIE experiments of decarboxylative carboxylethylation

Time (s)		*Product **87** (M) (H_2O)*	*Time (s)*		*Product **88** (M) (D_2O)*
900		0.005	900		0.004
1800		0.007	1800		0.006
2700		0.009	2700		0.008
3600		0.012	3600		0.009
4500		0.013	4500		0.011
5400		0.015	5400		0.012
Entry	*A1 (mmol)*	*Acid (mmol)*	*Disulfide (mmol)*	*[Fe] (mmol)*	*Initial rate ([M]/s)*
In H_2O	0.2	0.8	0.05	0.03	2.25E-06
In D_2O	0.2	0.8	0.05	0.03	1.78E-06

KIE: k_H/k_D = 1.3

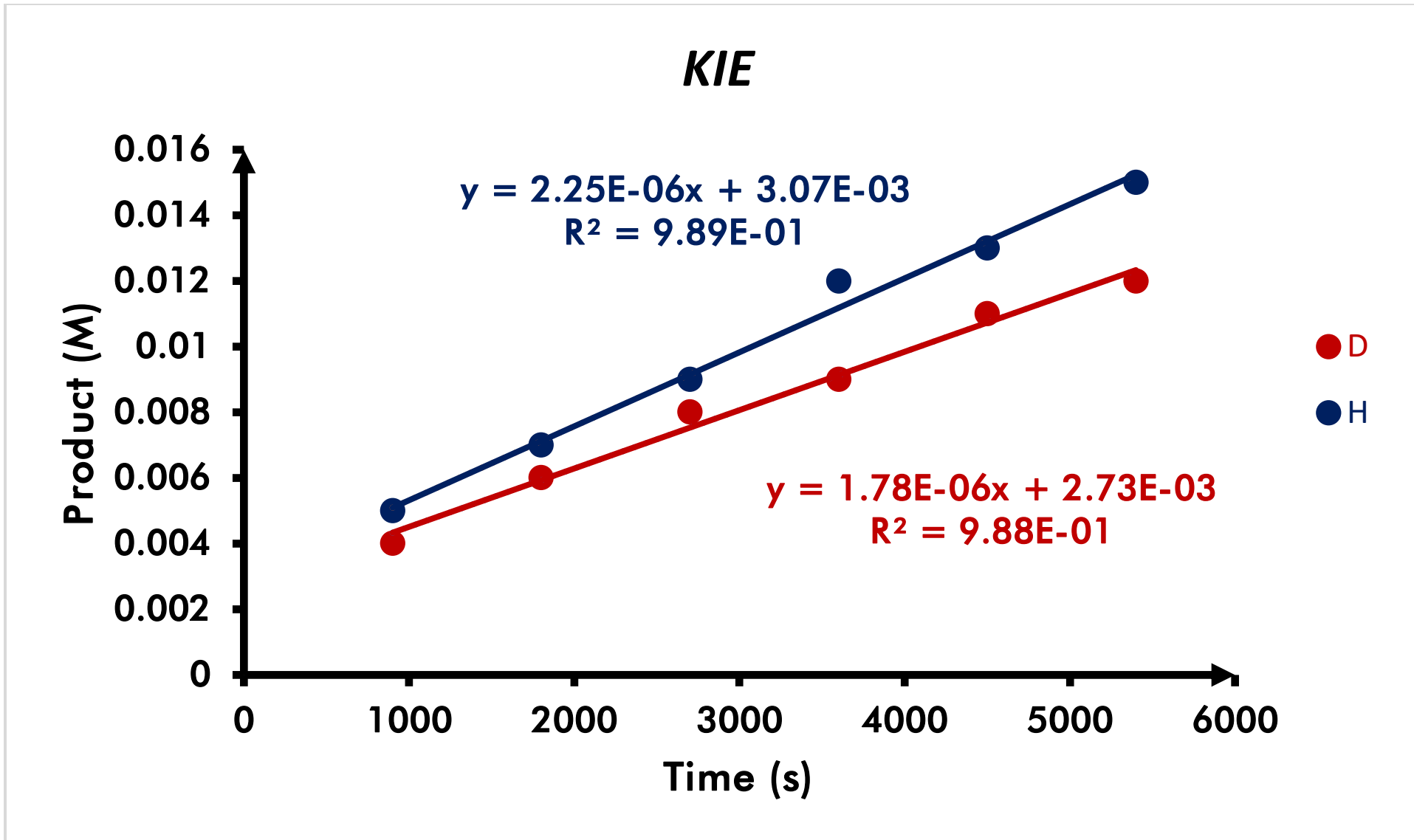

Supplementary Figure 8. Determination of k_H/k_D

Kinetic studies to determine reaction rate law

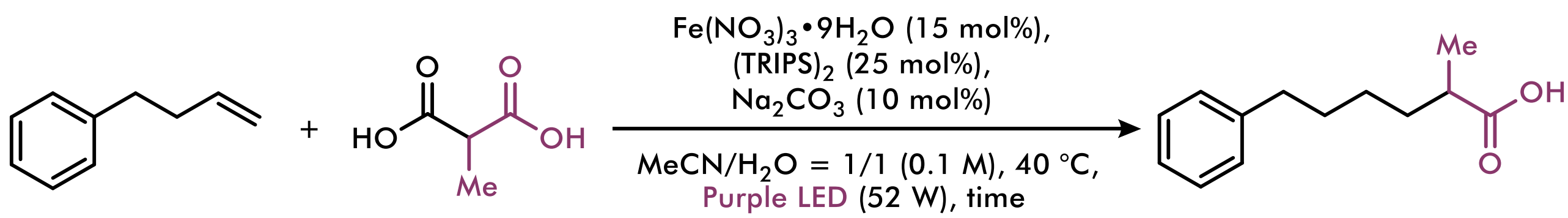

$Fe(NO_3)_3 \cdot 9H_2O$ (12 mg, 0.03 mmol, 15 mol%), bis(2,4,6-triisopropylphenyl)disulfide (22.8 mg, 0.05 mmol, 25 mol%), methylmalonic acid **MA-1** (93 mg, 0.8 mmol, 4.0 equiv.), and Na_2CO_3 (2.2 mg, 0.02 mmol, 10 mol%) were added in an oven-dried 8-mL test vial containing a Teflon®-coated magnetic stir bar. The vial was evacuated and backfilled with N_2 (repeated for 4 times), followed by addition of alkene **A1** (30 µL, 0.2 mmol, 1.0 equiv.) and CH_2Br_2 (internal standard) in $MeCN/H_2O$ (1/1, 0.1 M in regard to alkene) via syringe under N_2. Following this, the vial cap was attached a nitrogen balloon and sealed with vacuum grease and electric tape/parafilm for better air-tight protection. The reaction mixture was placed under 390nm Kessil® light (52 W) and allowed to react at 40 °C. At different time points, the aliquot of reaction mixture was directly separated in benzene-d_6 and brine. The organic layer was transferred to NMR tube and reaction progress was monitored by ^1H NMR.

Supplementary Table 9. Kinetic studies of decarboxylative carboxylethylation

Entry	***86*** *(mmol)*	*Acid (mmol)*	*Disulfide (mmol)*	*[Fe] (mmol)*	*Initial rate ([M]/s)*
1	0.2	0.8	0.05	0.03	2.25E-06
2	0.2	0.8	0.05	**0.015**	1.62E-06
3	0.2	0.8	0.05	**0.0075**	1.40E-06
4	0.2	0.8	**0.025**	0.03	2.19E-06
5	0.2	0.8	**0.0175**	0.03	2.22E-06
6	0.2	**0.4**	0.05	0.03	2.08E-06
7	0.2	**0.2**	0.05	0.03	2.14E-06
8	**0.1**	0.8	0.05	0.03	1.95E-06
9	**0.05**	0.8	0.05	0.03	2.22E-06

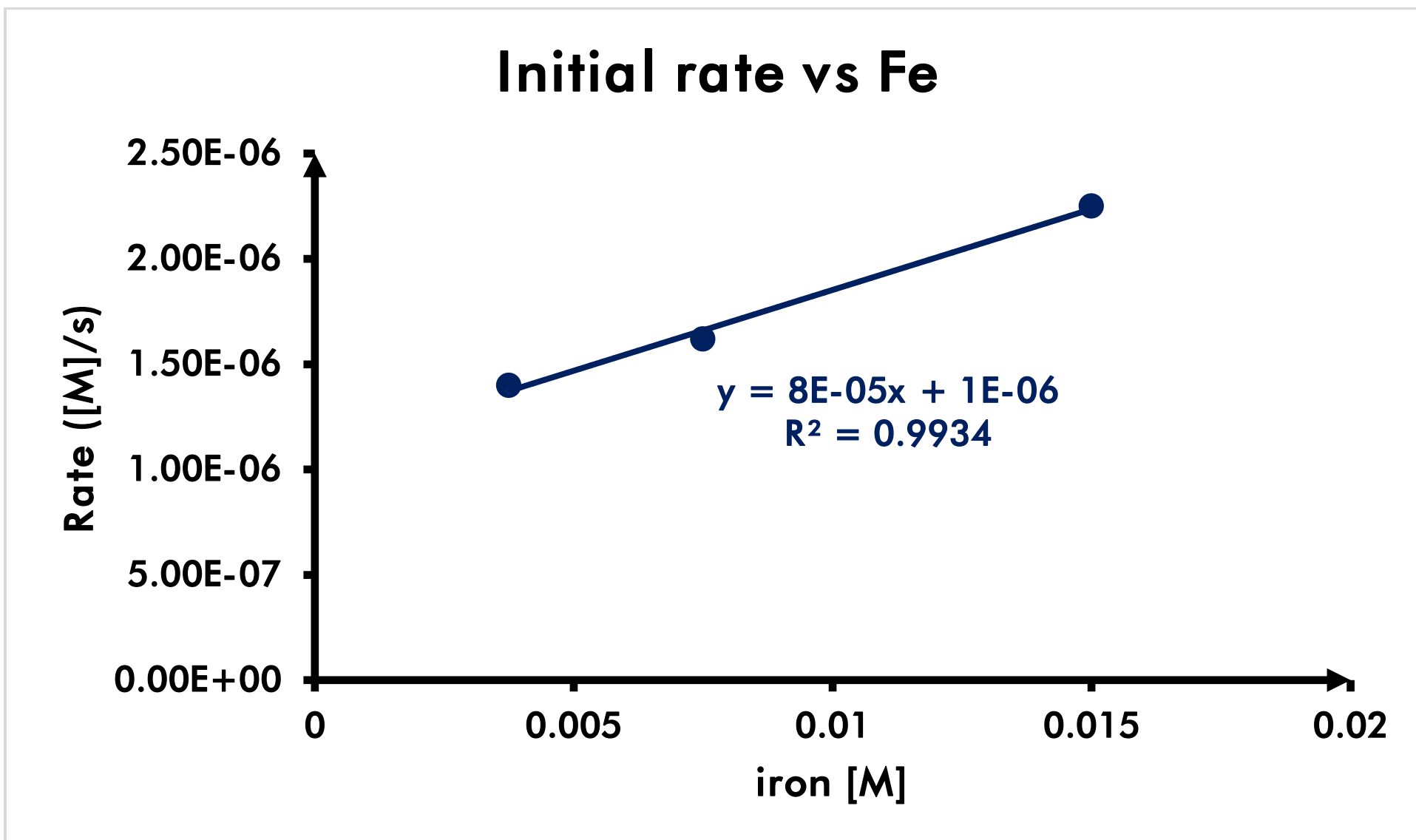

Supplementary Figure 9. Determinating reaction order of iron component.

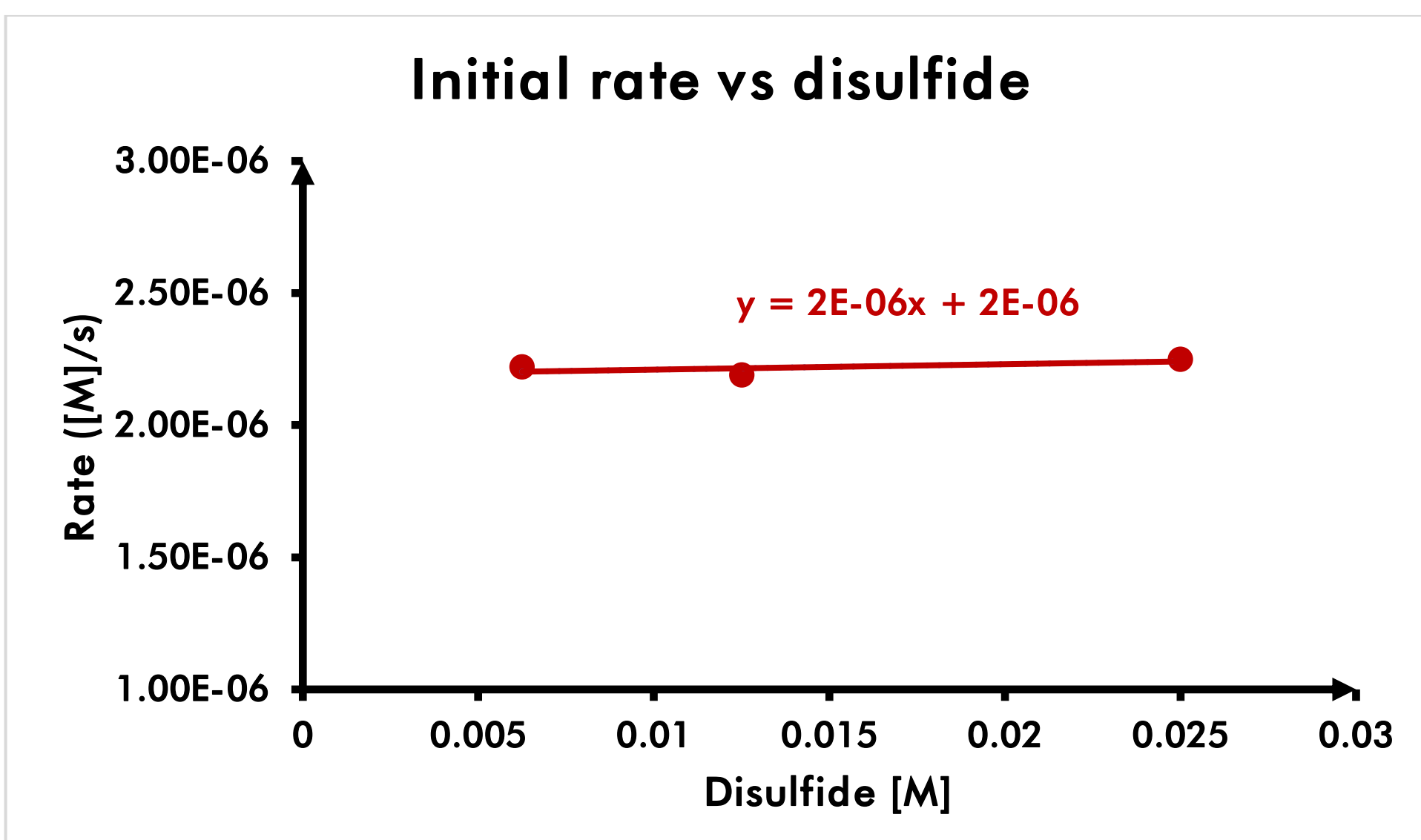

Supplementary Figure 10. Determinating reaction order of disulfide component.

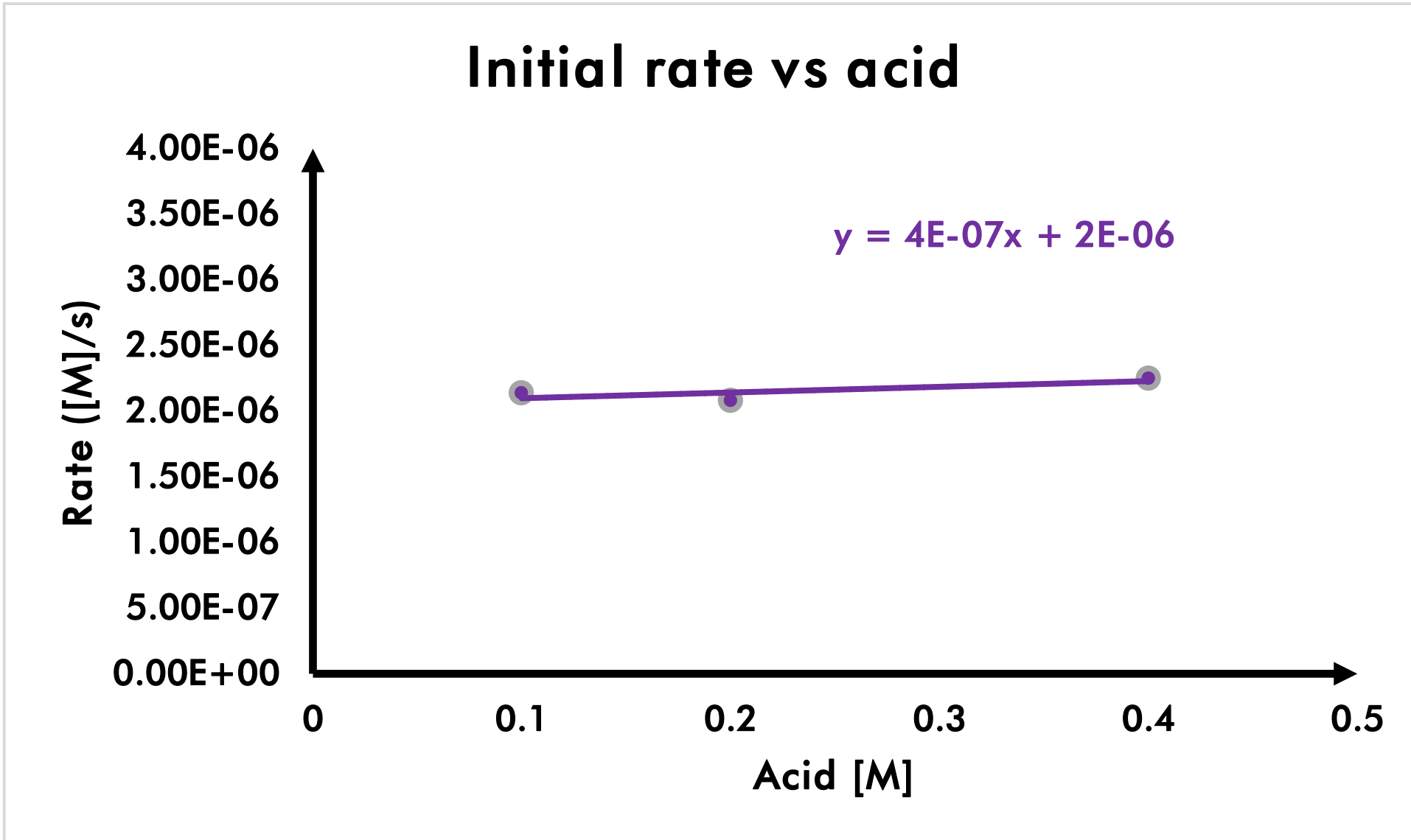

Supplementary Figure 11. Determinating reaction order of acid component.

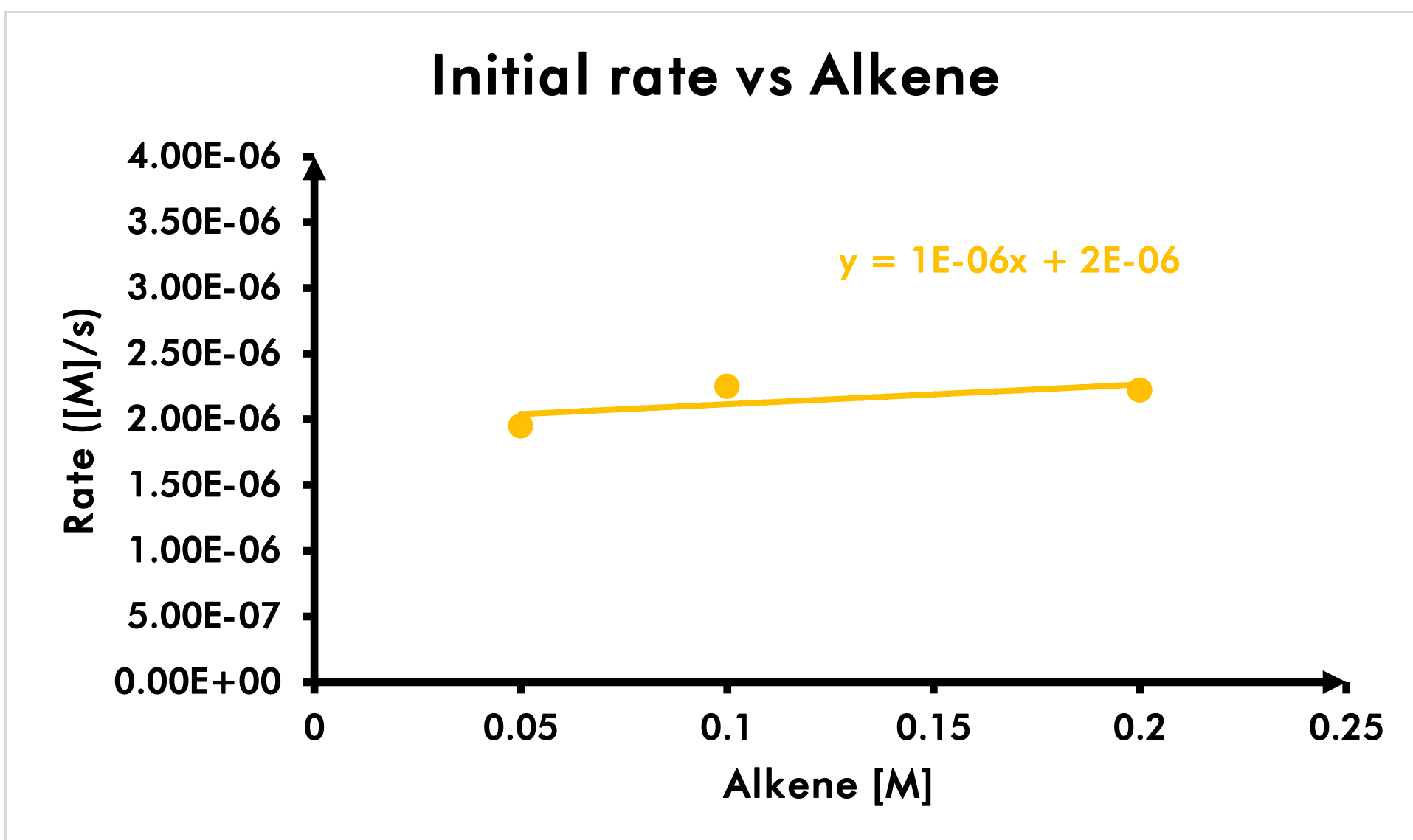

Supplementary Figure 12. Determinating reaction order of alkene component.

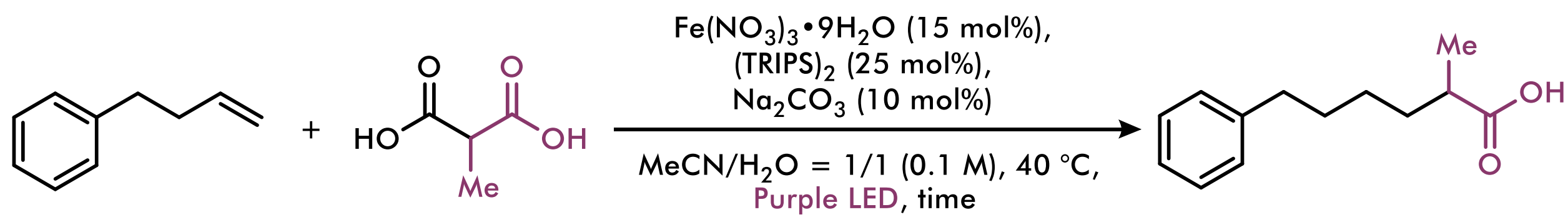

$Fe(NO_3)_3 \cdot 9H_2O$ (12 mg, 0.03 mmol, 15 mol%), bis(2,4,6-triisopropylphenyl)disulfide (22.8 mg, 0.05 mmol, 25 mol%), methylmalonic acid **MA-1** (93 mg, 0.8 mmol, 4.0 equiv.), and Na_2CO_3 (2.2 mg, 0.02 mmol, 10 mol%) were added in an oven-dried 8-mL test vial containing a Teflon®-coated magnetic stir bar. The vial was evacuated and backfilled with N_2 (repeated for 4 times), followed by addition of alkene **A1** (30 μL, 0.2 mmol, 1.0 equiv.) and CH_2Br_2 (internal standard) in MeCN/H_2O (1/1, 0.1 M in regard to alkene) via syringe under N_2. Following this, the vial cap was attached a nitrogen balloon and sealed with vacuum grease and electric tape/parafilm for better air-tight protection. The reaction mixture was placed under 390nm Kessil® light (52 W) and allowed to react at 40 °C. At different time points, the aliquot of reaction mixture was directly sepaeated in benzene-d_6 and brine. The organic layer was transferred to NMR tube and reaction progress was monitored by ^1H NMR.

Supplementary Table 10. Kinetic studies of decarboxylative carboxylethylation

Entry	***86*** *(mmol)*	*Acid (mmol)*	*Disulfide (mmol)*	*[Fe] (mmol)*	*Initial rate ([M]/s)*
13 W	0.2	0.8	0.05	0.03	1.11E-06
26 W	0.2	0.8	0.05	0.03	1.78E-06
52 W	0.2	0.8	0.05	0.03	2.25E-06

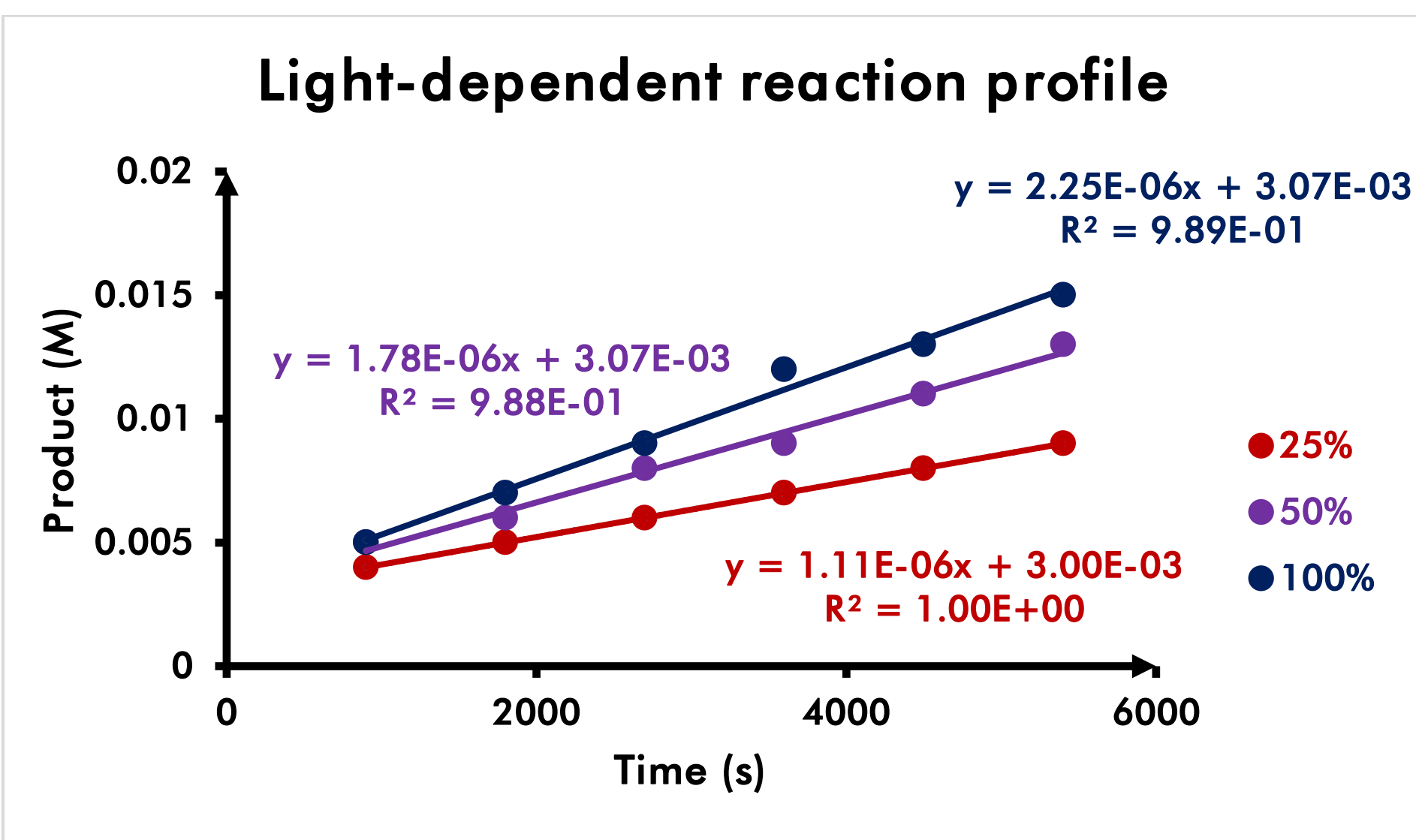

Supplementary Figure 13. Light intensity impact on reaction rate.

Light on-off experiment

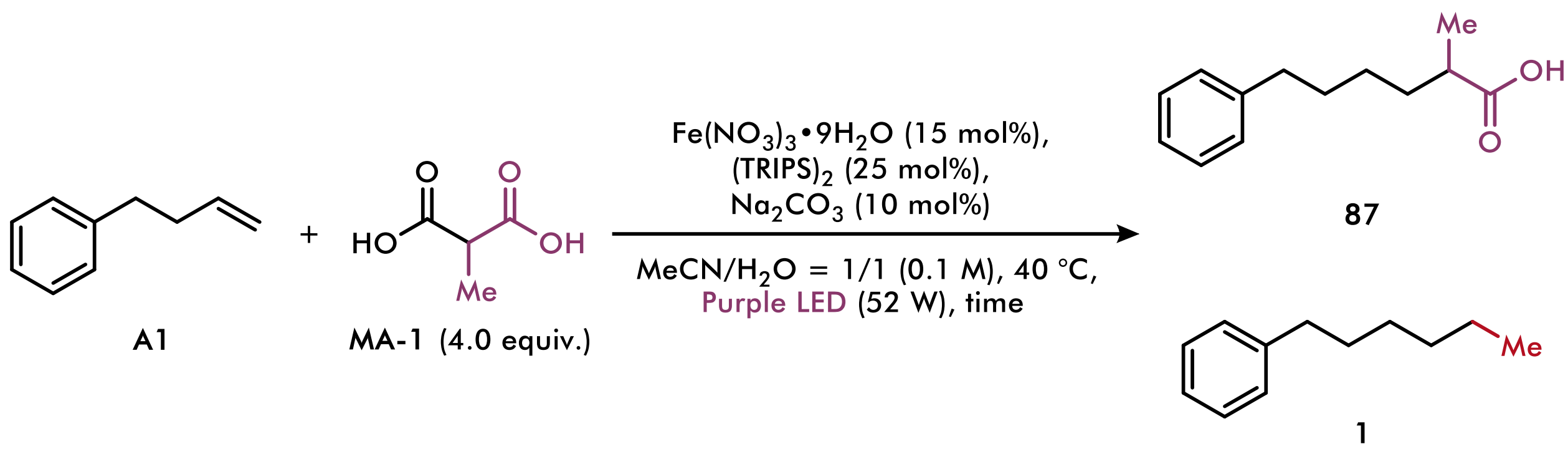

$Fe(NO_3)_3 \cdot 9H_2O$ (12 mg, 0.03 mmol, 15 mol%), bis(2,4,6-triisopropylphenyl)disulfide (22.8 mg, 0.05 mmol, 25 mol%), methylmalonic acid **MA-1** (93 mg, 0.8 mmol, 4.0 equiv.), and Na_2CO_3 (2.2 mg, 0.02 mmol, 10 mol%) were added in an oven-dried 8-mL test vial containing a Teflon®-coated magnetic stir bar. The vial was evacuated and backfilled with N_2 (repeated for 4 times), followed by addition of alkene **A1** (30 μL, 0.2 mmol, 1.0 equiv.) in MeCN/H_2O (1/1, 0.1 M in regard to alkene) via syringe under N_2. Following this, the vial cap was attached a nitrogen balloon and sealed with vacuum grease and electric tape/parafilm for better air-tight protection. The reaction mixture was placed under 390nm Kessil® light (52 W) and allowed to react at 40 °C. At different time points, the aliquot of reaction mixture was directly separeated in benzene-d_6 and brine, followed by addition of 1,1,2,2-tetrachloroethane (internal standard). The organic layer was transferred to NMR tube and reaction progress was monitored by ^1H NMR.

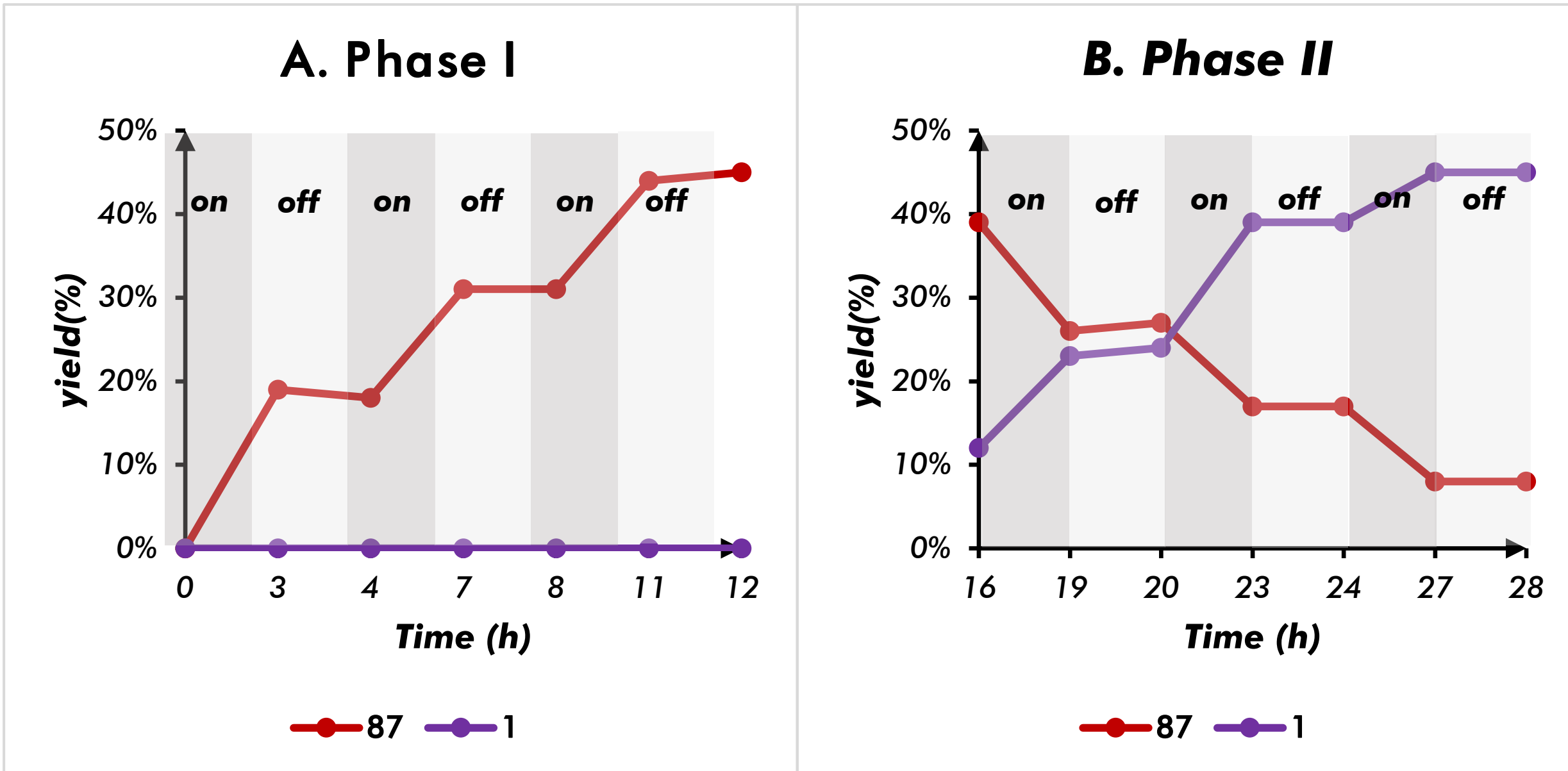

Supplementary Figure 14. Investigating the potential radical chain process through light on-off experiment. (a) Phase I: hydroalkylcarboxylation (b) Phase II: protodecarboxylation.

Facile quantum yield determination via NMR actinometry[5]

The photon flux of a standardized reaction apparatus was first measured using o-nitrobenzaldehyde as a chemical actinometer via the following procedure:

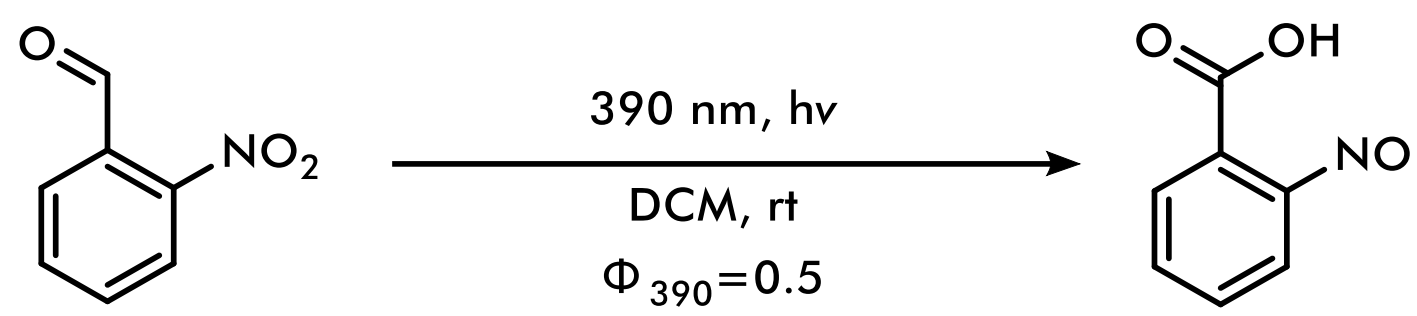

o-nitrobenzaldehyde were added in an oven-dried 8-mL test vial containing a Teflon®-coated magnetic stir bar. The vial was evacuated and backfilled with N_2 (repeated for 4 times), followed by addition of DCM and CH_2Br_2 (internal standard) via syringe under N_2. Following this, the vial cap was attached a nitrogen balloon and sealed with vacuum grease and electric tape/parafilm for better air-tight protection. The reaction mixture was placed under 390nm Kessil® light (52 W) and allowed to react at 40 °C using a water bath. At different time points, an aliquot of reaction mixture was removed and directly mixed with chloroform-d_3 and transferred to NMR tube and reaction progress was monitored by ^1H NMR. The initial rates were obtained for each of these concentrations (supplementary table 11) extracted from the linear region of the *o*-nitrobenzaldehyde consumption data obtained in the first 4 minutes of reaction (linear region).

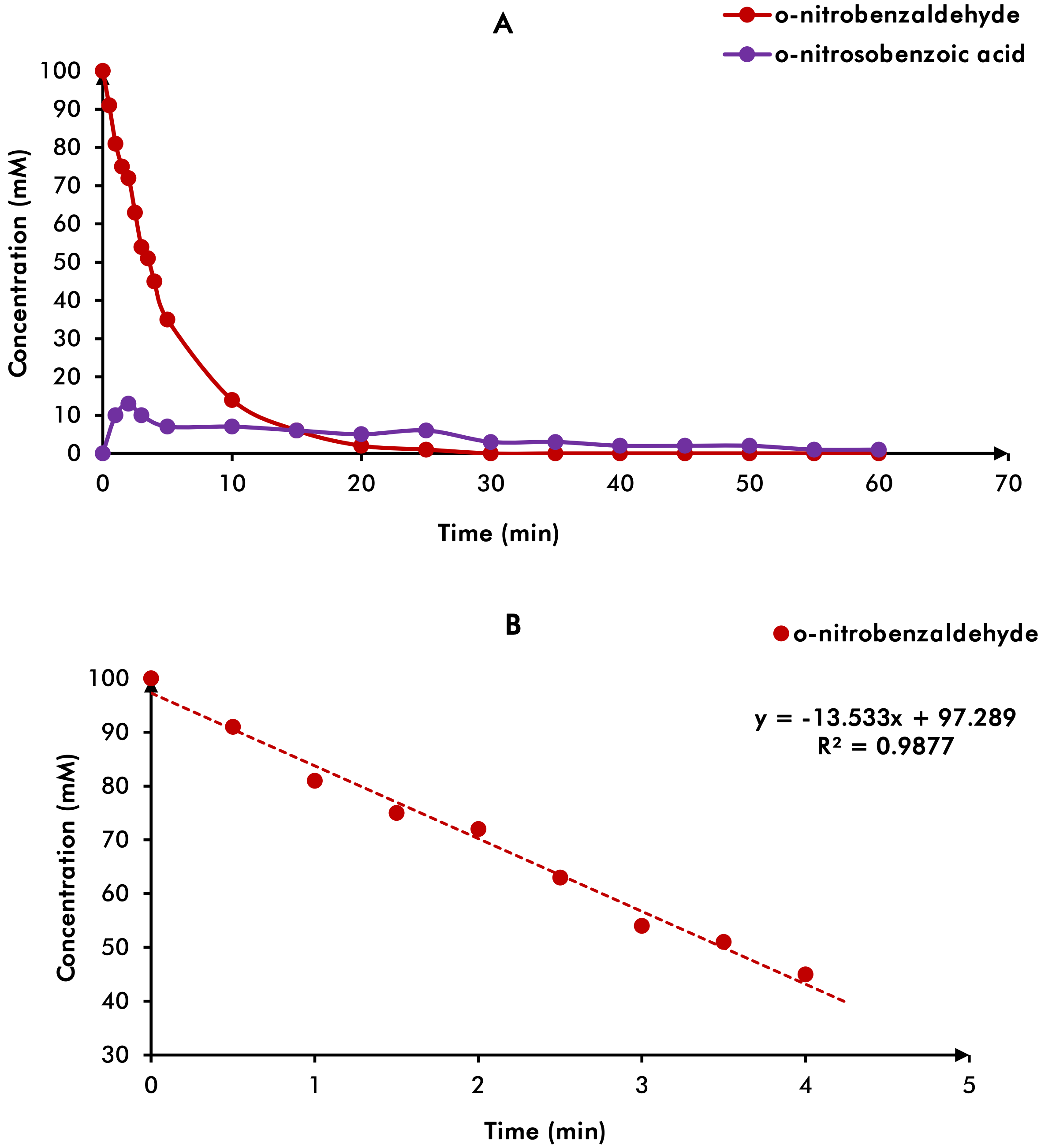

Supplementary Figure 15. Photochemistry of o-nitrobenzaldehyde. (A) Temporal concentration profiles monitored by ^{1}H NMR spectroscopy for the transformation of o-nitrobenzaldehyde (100 mM) to o-nitrosobenzoic acid under visible light light (390 nm) irradiation. (B) Extraction of the initial rate using the data of the first 4 minutes.

Supplementary Table 11. Initial rates obtained at different initial concentrations of o-NBA.

Entry	*50 mM*	*100 mM*	*200 mM*
Rate, mM/min	13.1	13.5	12.8

The 0th order behavior of 0-NBA decomposition was confirmed across relevant concentration ranges using the standardized reaction apparatus. The rate data (k_0) at 100 mM were used to calculate the incident light intensity (I_0) received by the reaction mixture using the standardized reaction apparatus using the following procedure:

(1) Convert k_0 to M/s: 13.5 mM/min = 2.26E^{-04} M/s

(2) Calculate I_0 using k_0 and the known ϕ (0.5) of o-nitrobenzene photodecomposition via $I_0 = \frac{k_0}{\phi} = \frac{2.26\text{E}-04\ \text{M/s}}{0.5} = 4.52\text{E-04 einstein/L*s}$

Quantum yield measurement

Following the *o*-nitrobenzene measurement of the standardized reaction apparatus, standard reactions were performed using the exact same apparatus, light intensity settings, and water bath temperature using the following procedures:

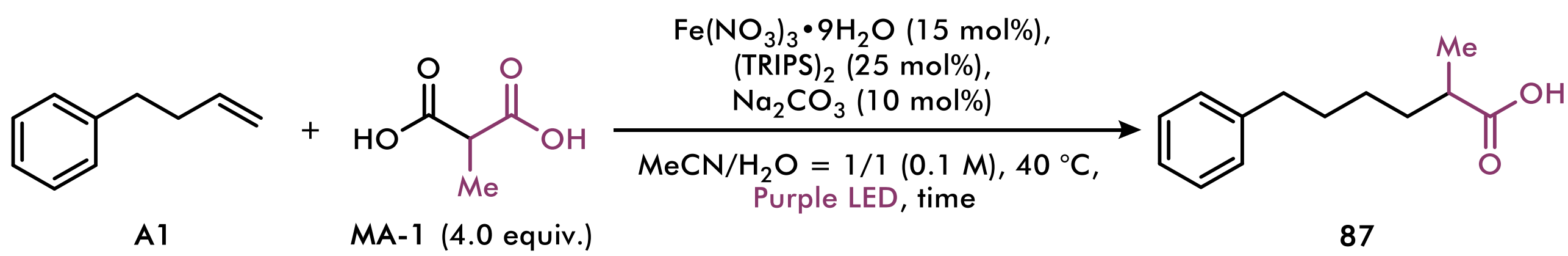

$Fe(NO_3)_3 \cdot 9H_2O$ (12 mg, 0.03 mmol, 15 mol%), bis(2,4,6-triisopropylphenyl)disulfide (22.8 mg, 0.05 mmol, 25 mol%), methylmalonic acid **MA-1** (93 mg, 0.8 mmol, 4.0 equiv.), and Na_2CO_3 (2.2 mg, 0.02 mmol, 10 mol%) were added in an oven-dried 8-mL test vial containing a Teflon®-coated magnetic stir bar. The vial was evacuated and backfilled with N_2 (repeated for 4 times), followed by addition of alkene **A1** (30 µL, 0.2 mmol, 1.0 equiv.) and CH_2Br_2 (internal standard) in $MeCN/H_2O$ (1/1, 0.1 M in regard to alkene) via syringe under N_2. Following this, the vial cap was attached a nitrogen balloon and sealed with vacuum grease and electric tape/parafilm for better air-tight protection. The reaction mixture was placed in the standardized reaction apparatus and irradiated under 390nm Kessil® light (52 W) at 40 °C using a water bath. At different time points, an aliquot of reaction mixture was directly separated in benzene-d_6 and brine. The organic layer was transferred to NMR tube and

reaction progress was monitored by ^{1}H NMR. This procedure provided an initial rate (k_0) of alkene formation of 2.25E^{-06} M/s.

The quantum yield was then calculated using the incident flux determined from prior actinometry and the following relation:

$$\phi = \frac{k_0}{I_0} = \frac{2.25E{-}06\ M/s}{4.52\text{E}{-}04\ \text{einstein/L}{*}\text{s}} = 0.50\ \%$$

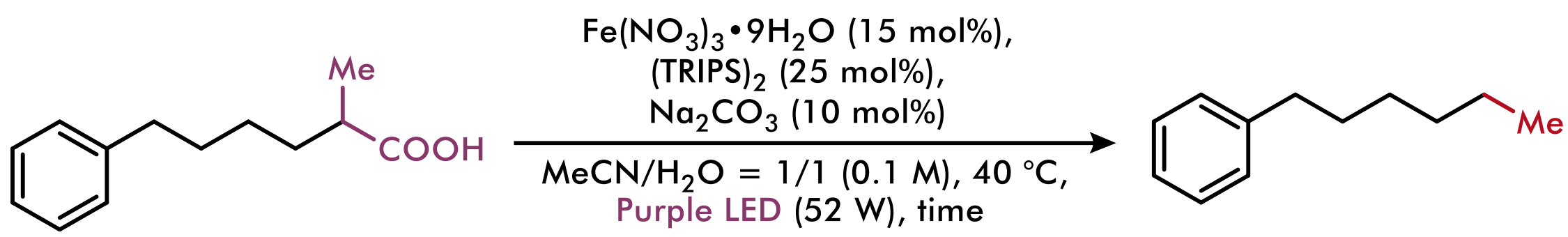

87 1

$Fe(NO_3)_3 \cdot 9H_2O$ (12 mg, 0.03 mmol, 15 mol%), bis(2,4,6-triisopropylphenyl)disulfide (22.8 mg, 0.05 mmol, 25 mol%), carboxylic acid **87** (41 mg, 0.2 mmol, 1.0 equiv.), and Na_2CO_3 (2.2 mg, 0.02 mmol, 10 mol%) were added in an oven-dried 8-mL test vial containing a Teflon®-coated magnetic stir bar. The vial was evacuated and backfilled with N_2 (repeated for 4 times), followed by addition of CH_2Br_2 (internal standard) in $MeCN/H_2O$ (1/1, 0.1 M in regard to alkene) via syringe under N_2. Following this, the vial cap was attached a nitrogen balloon and sealed with vacuum grease and electric tape/parafilm for better air-tight protection. The reaction mixture was placed in the standardized reaction apparatus and irradiated under 390nm Kessil® light (52 W) at 40 °C using a water bath. At different time points, an aliquot of reaction mixture was directly separated in benzene-d_6 and brine. The organic layer was transferred to NMR tube and reaction progress was monitored by ^{1}H NMR. This procedure provided an initial rate (k_0) of hydroalkylation product **1** formation of 1.98E^{-06} M/s.

The quantum yield was then calculated using the incident flux determined from prior actinometry and the following relation:

$$\phi = \frac{k_0}{I_0} = \frac{1.98E{-}06\ M/s}{4.52\text{E}{-}04\ \text{einstein/L}{*}\text{s}} = 0.44\ \%$$

Full reaction profiles for the invesitigation of free carboxylic acid intermediate and hydroalkylation product formation

General procedure $Fe(NO_3)_3 \cdot 9H_2O$ (12 mg, 0.03 mmol, 15 mol%), bis(2,4,6-triisopropylphenyl)disulfide (22.8 mg, 0.05 mmol, 25 mol%), methylmalonic acid **MA-1** (93 mg, 0.8 mmol, 4.0 equiv.) or **MA-4** (115 mg, 0.8 mmol, 4.0 equiv.) or **MA-11** (98 mg, 0.8 mmol, 4.0 equiv.), and Na_2CO_3 (2.2 mg, 0.02 mmol, 10 mol%) were added in an oven-dried 8-mL test vial containing a Teflon®-coated magnetic stir bar. The vial was evacuated and backfilled with N_2 (repeated for 4 times), followed by addition of alkene **A1** (30 μL, 0.2 mmol, 1.0 equiv.) or **A9** (38 mg, 0.2 mmol, 1.0 equiv.) in MeCN/H_2O (1/1, 0.1 M in regard to alkene) via syringe under N_2. Following this, the vial cap was attached a nitrogen balloon and sealed with vacuum grease and electric tape/parafilm for better air-tight protection. The reaction mixture was placed under 390nm Kessil® light (52 W) and allowed to react at 40 °C. At different time points, the aliquot of reaction mixture was directly separeated in benzene-d_6 and brine, followed by addition of 1,1,2,2-tetrachloroethane (internal standard). The organic layer was transferred to NMR tube and reaction progress was monitored by ^1H NMR.

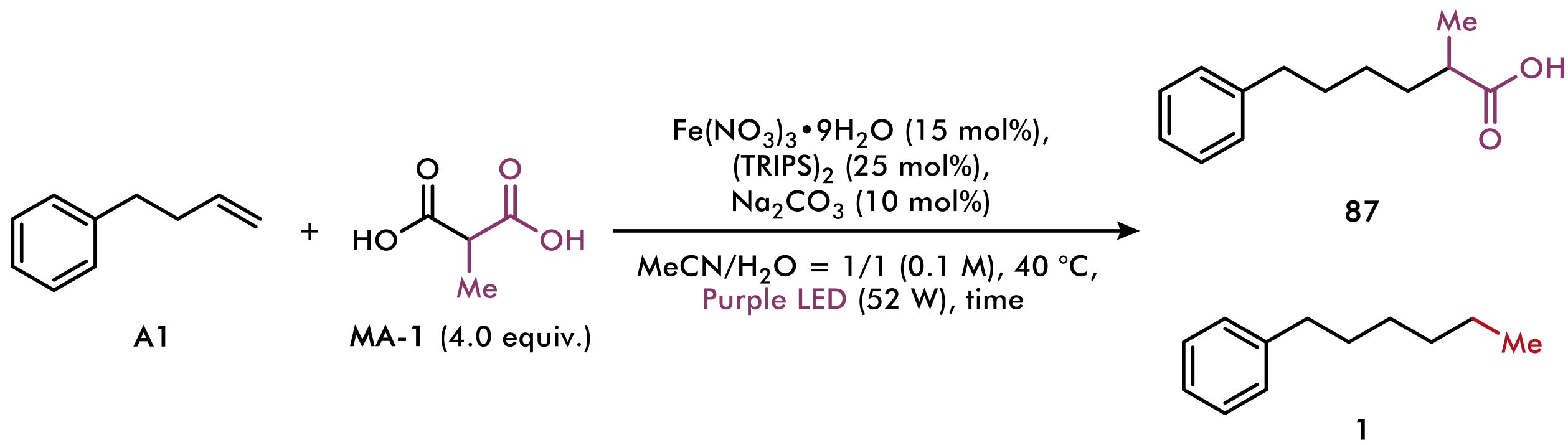

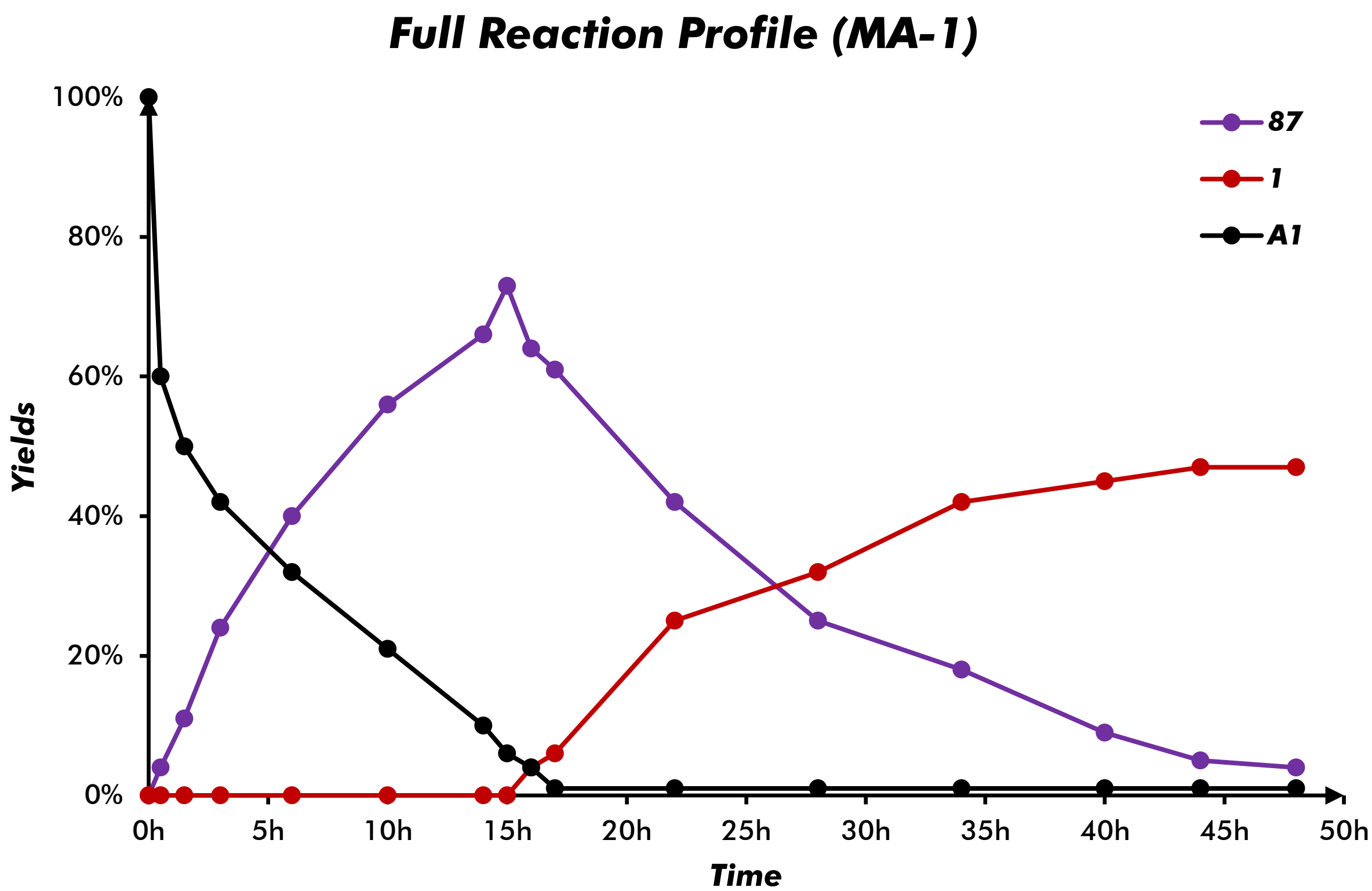

Supplementary Figure 16. Tracking full reaction progression with mono-alkylated malonic acid (**MA-1**).

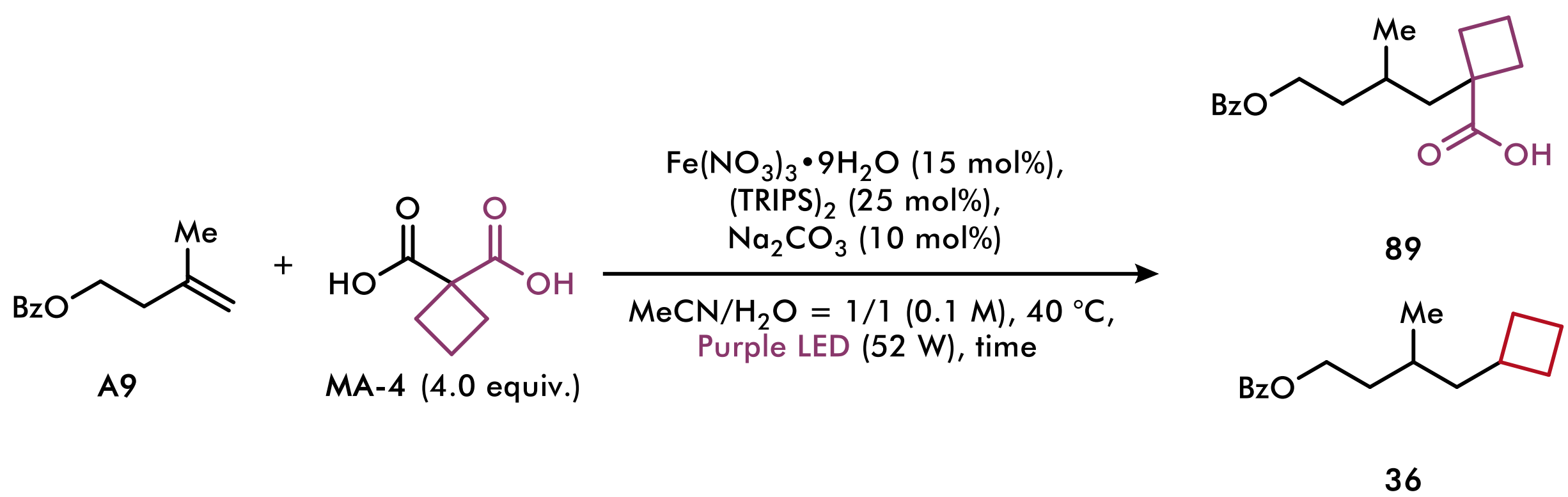

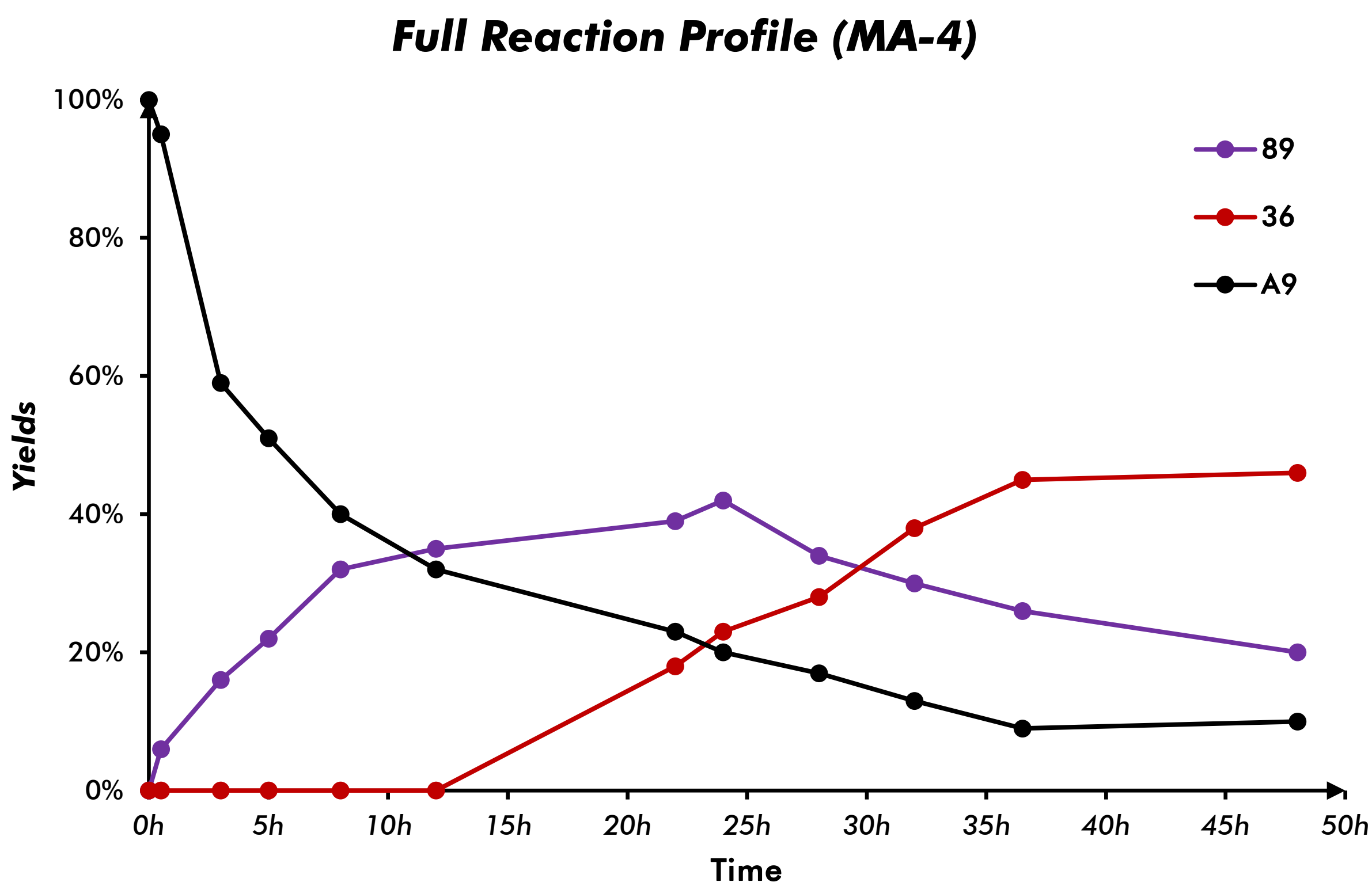

Supplementary Figure 17. Tracking full reaction progression with dialkylated malonic acid (**MA-4**).

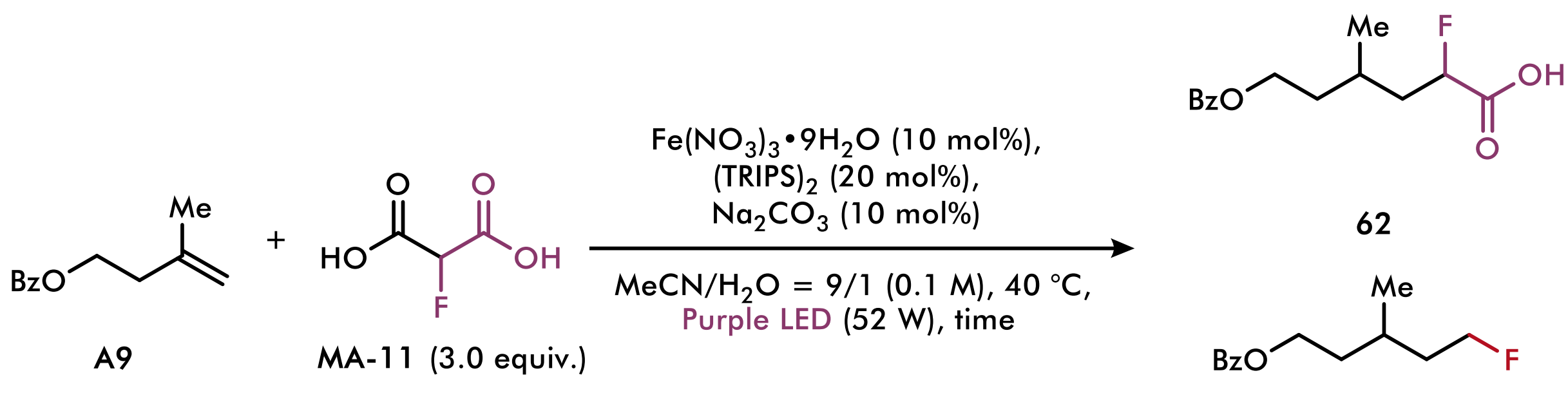

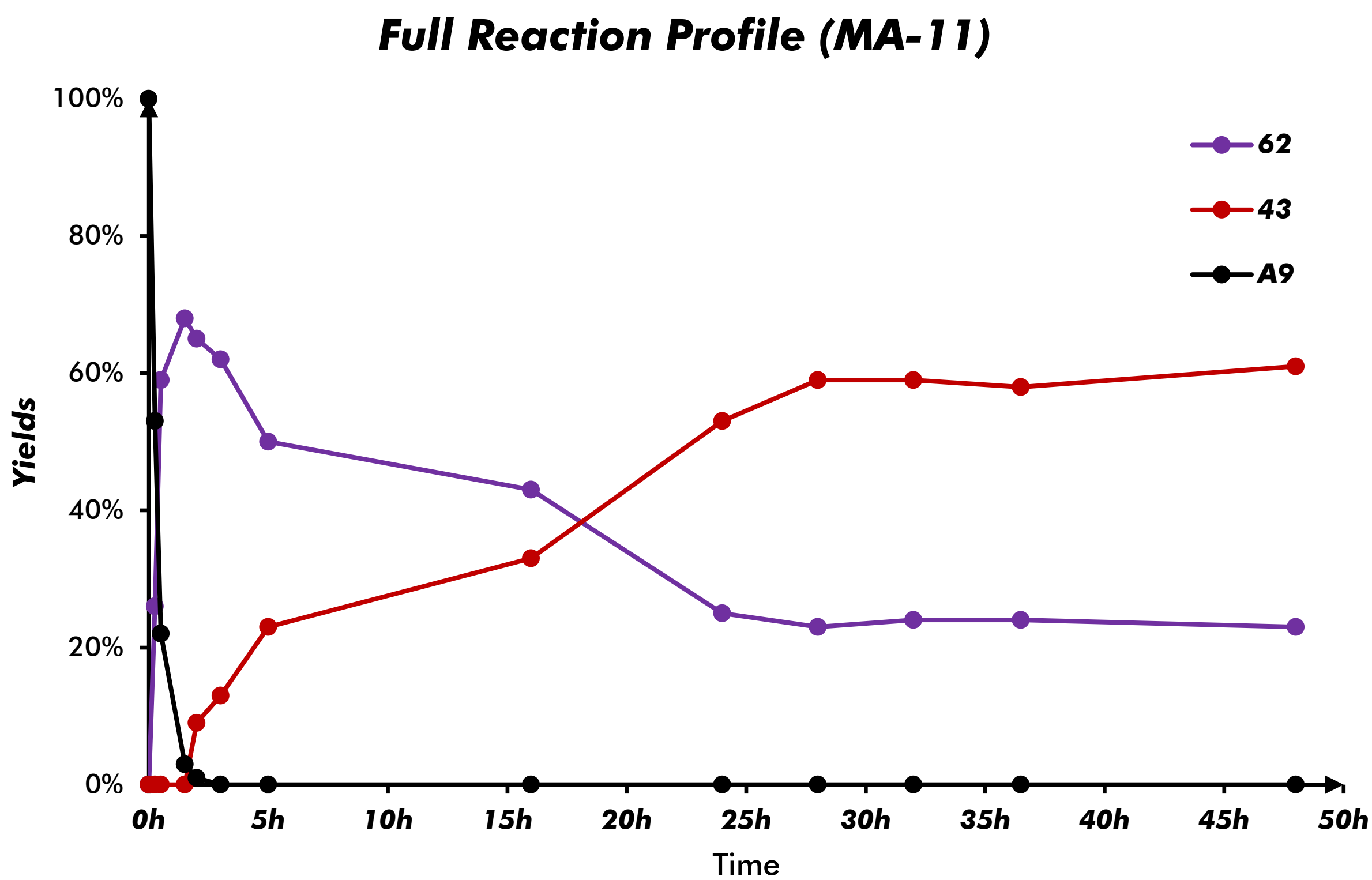

Supplementary Figure 18. Tracking full reaction progression with EWG-substituted malonic acid (**MA-11**).

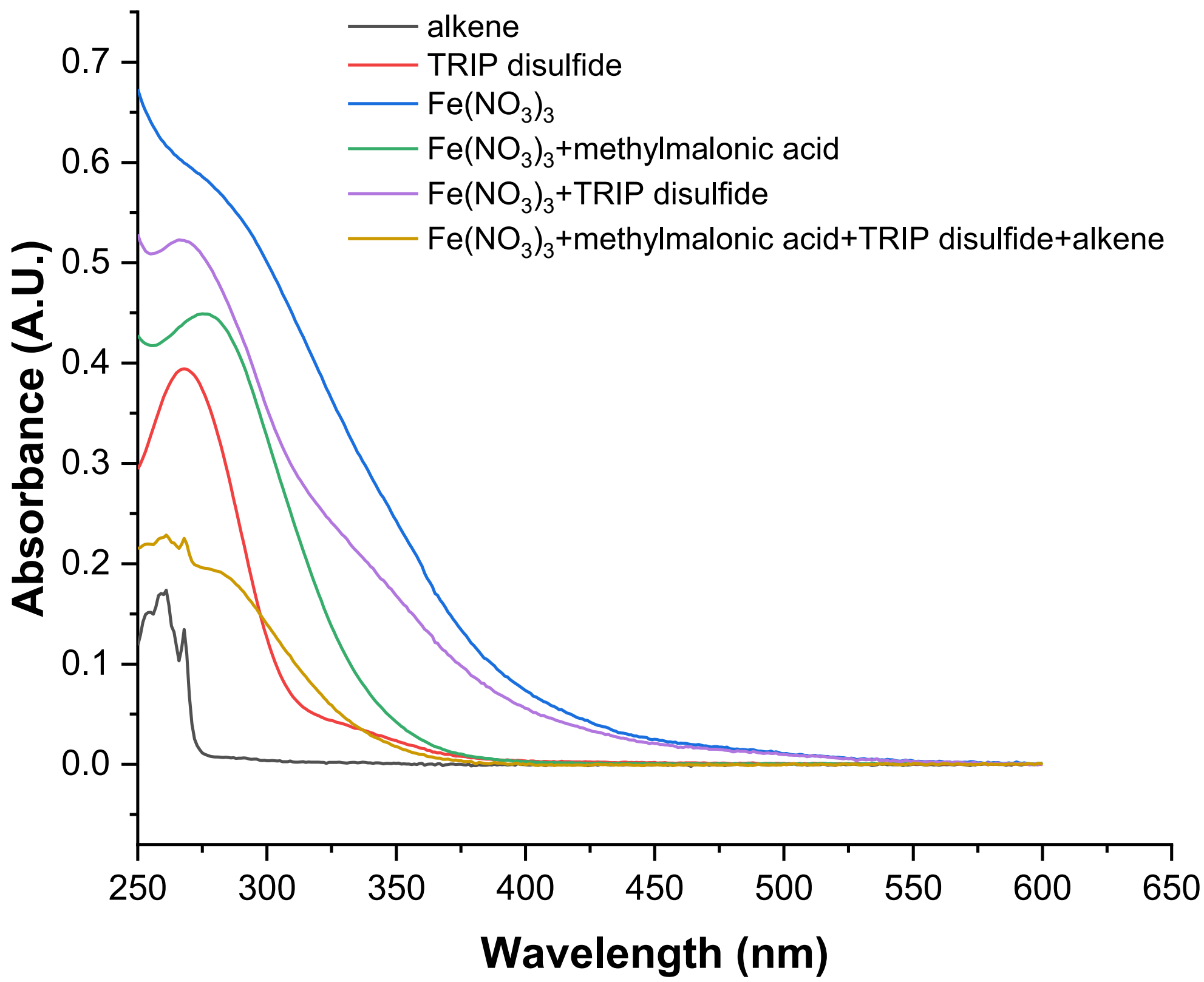

Supplementary Figure 19. UV-Vis analysis of phtoactive iron species.

Experimental procedure $Fe(NO_3)_3 \cdot 9H_2O$ (1 mg, 2.5 µmol, 15 mol%), bis(2,4,6-triisopropylphenyl)disulfide (1.9 mg, 4.2 µmol, 25 mol%), alkene **A1** (2.5 µL, 16.7 µmol, 1 equiv.)]. To prepare the sample solution for each trial, the specified quantities of the required compounds above were dissolved in 200 µL of MeCN/H_2O (1/1) under N_2 to create a stock solution. Subsequently, a 50 µL aliquot of this stock solution was diluted with 3 mL of MeCN/H_2O (1/1) under N_2 for UV-Vis measurement. The UV–Vis absorption spectra (Supplementary Figure 19) reveal that the addition of methylmalonic acid to the iron(III) nitrate in MeCN/H_2O solution induces significant alterations in the absorbance spectrum (blue to green), suggesting the formation of iron(III) malonate species.[6,7] In contrast, the addition of disulfide exhibited no detectable change in the absorption patterns. Next, we investigated the visible light-induced homolysis capability of the iron(III) malonate species

for the decarboxylation of methylmalonic acid using time-resolved UV-VIS analysis (Supplementary Figure 20). [Note: The sample solution was prepared according to the previously described procedure in a fluorometer cell under N_2; $FeCl_3 \cdot 6H_2O$ (0.68 mg, 2.5 µmol, 15 mol%) was used instead of iron(III) nitrate to prevent the intrinsic oxidation ability of the nitrate counteranion obstructing the formation and examination of iron(II) species. The resulting absorption spectrum (0 s) suggests that iron(III) malonate species might be forming in the reaction (Supplementary Figure 20, Panel A, grey). We then exposed the solution to a 390 nm Kessil® lamp (26 W) for a total of 3 minutes and collected absorption spectra at different time intervals. During the first minute, there was minimal difference from the preliminary spectrum. However, after one and a half minutes of light exposure, we observed a decrease in the intensity of the absorption spectrum, with patterns increasingly resembling the spectrum of potential iron(II) malonate species (Supplementary Figure 20, Panel B), indicating an oxidation/reduction process between the iron(III) center and malonate through inner sphere electron transfer (ISET). This suggests the potential photolysis of iron(III) malonate species and supports the hypothesis that our reaction is photochemical in nature. Finally, iron(II) and iron(III) species possibly degraded after 5 minutes of light exposure, leaving a weak absorption around 275 nm. From these results and previous studies showing the photo-redox process between malonic acid and iron(III) species, as well as kinetic studies indicating that the rate-limiting step of our reaction might be the decarboxylation of iron-malonate reactive species (~1st order dependence on iron(III) nitrate) and that the reaction exhibited dependence on light intensity (~1st order dependence on light intensity), we postulate that iron(III) species can oxidize malonic acid under our conditions to generate α-carboxyalkyl radicals through a visible light-induced homolysis process of the iron-malonate reactive species.

A

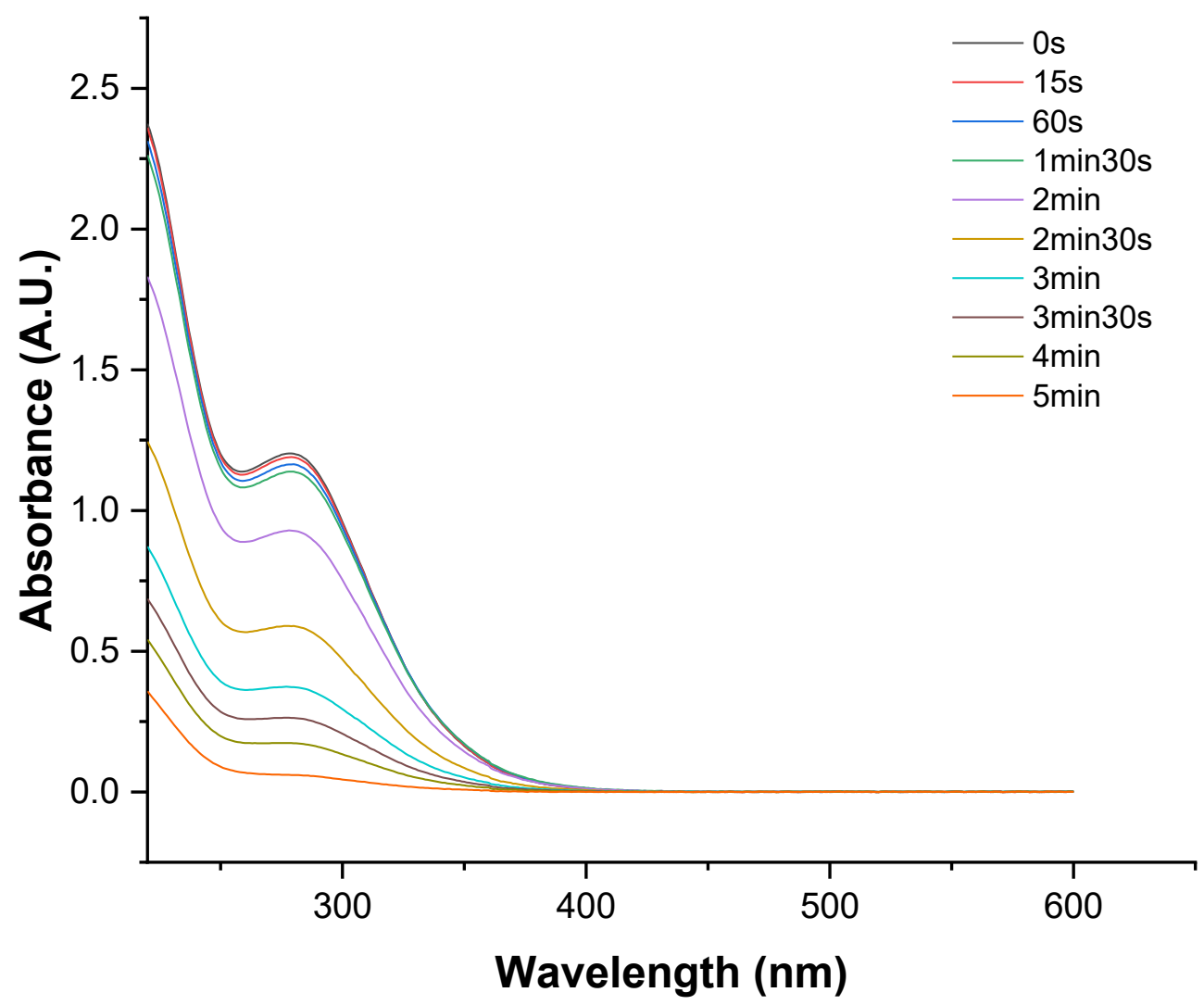

B

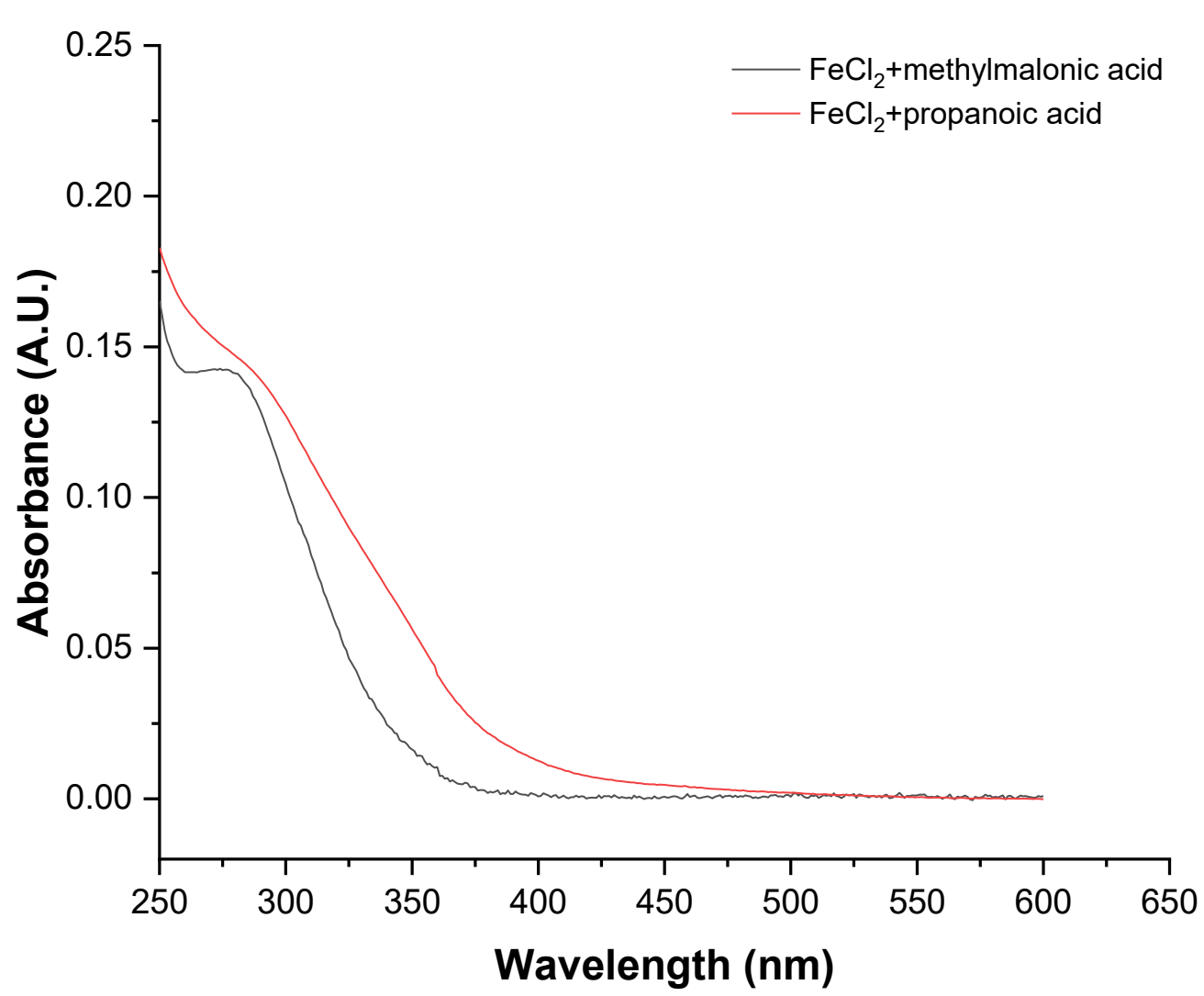

Supplementary Figure 20. Time-resolved UV-Vis analysis. (A) Investigating the visible light-induced homolysis capability of the iron(III) malonate species for the decarboxylation of methylmalonic acid (B) Detecting the formation of iron(II) malonate species through UV-Vis analysis.

Supplementary scope of alkene hydroalkylation

Realizing the low product yields obtained in several cases potentially due to the inhibited reactivity of carboxylic acid intermediates by the excessive amount of propionic acid generated as a side product through the reaction progress, and the less effective radical addition leading to the unreacted starting materials remaining, we managed to apply the revised procedure from the hydromethylation of **A9** abovementioned to these unproductive substrates to solve the low yielding issue. Through the vigorous removement of propionic acid and extension of reaction time plus the assistance from water-soluble thiols, we were able to eventually optimize the reaction with higher yields in most cases.

Supplementary Table 12. Revised procedures for optimizing unproductive substrates in hydroethlyation.

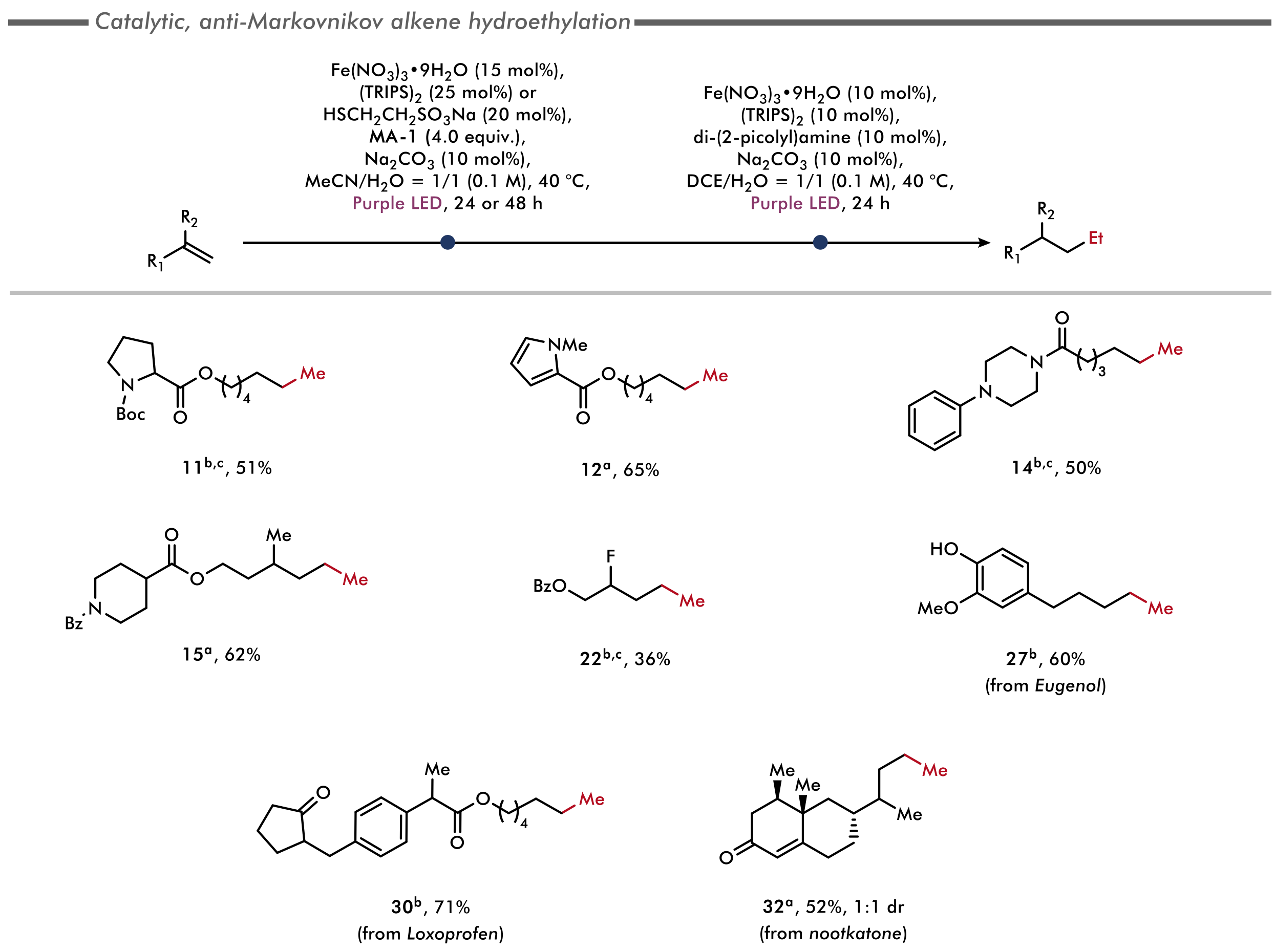

a) 24h at the first step b) 48h at the first step c) $HSCH_2CH_2SO_3Na$ was used at the first step

Revised general procedure for the alkene hydroethylation To an oven-dried 8-mL test vial containing a Teflon®-coated magnetic stir bar was added $Fe(NO_3)_3 \cdot 9H_2O$ (6 mg, 0.015 mmol, 15 mol%), Na_2CO_3 (1.1 mg, 0.01 mmol, 10 mol%), bis(2,4,6-triisopropylphenyl)disulfide (11.4 mg, 0.025 mmol, 25 mol%) or sodium 2-mercaptoethanesulfonate (3.3 mg, 0.02 mmol, 20 mol%) (for **11**, **14**, **22**), **MA-1** (46 mg, 0.8 mmol, 4.0 equiv.). The vial was evacuated and backfilled with N_2 (repeated for 4 times), followed by addition of alkenes (1.0 equiv.) and MeCN/H_2O (1/1, 0.1 M in regard to alkene) via syringe under N_2. Following this, the vial cap was attached a nitrogen balloon and sealed with vacuum grease and electric tape/parafilm for better air-tight protection. The reaction mixture was placed under 390nm Kessil® light (52 W) and allowed to react at 40 °C for 24 hours (for **12**, **15**, **32**) or 48 hours (for **11**, **14**, **22**, **27**, **30**). Following this, the reaction mixture was cooled to room temperature and then washed with brine and extracted with EtOAc. The combined organic phase was concentrated through rotary evaporation under reduced pressure for removing the solvent and the generated propionic acid, and the residue was filtered through a microscale (pipette) column (5 cm) of silica gel which was subsequently rinsed with EtOAc. The filtrate was concentrated and put under high vacuum pump for a short time to remove the residue of propionic acid. This resulting mixture was then dissolved with dichloroethane (0.5 mL) and transferred into a nitrogen-evacuated and backfilled 8-mL test vial containing $Fe(NO_3)_3 \cdot 9H_2O$ (4.0 mg, 0.01 mmol, 10 mol%), bis(2,4,6-triisopropylphenyl)disulfide (4.6 mg, 0.01 mmol, 10 mol%), and Na_2CO_3 (0.01 mmol, 10 mol%). To this tube was added dipicolylamine (1.8 μL, 0.01 mmol, 10 mol%) and water (0.5 mL). The reaction mixture was left to react for another 24 hours under the setup aforementioned in general procedure A. The product was then obtained by following the workup procedure.

NMR characterization of corresponding products

Hexylbenzene (1)

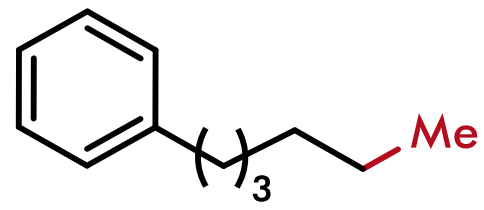

Prepared according to the general procedure A using $Fe(NO_3)_3 \cdot 9H_2O$ (6.0 mg, 0.015 mmol, 15 mol%), Na_2CO_3 (1.1 mg, 0.01 mmol, 10 mol%), bis(2,4,6-triisopropylphenyl)disulfide (11.4 mg, 0.025 mmol, 25 mol%), malonic acid **MA-1** (47 mg, 0.4 mmol, 4.0 equiv.), and alkene **A1** (15 μL, 0.1 mmol, 1.0 equiv.). Yield was determined by ^1H NMR analysis of the crude reaction mixture with 1,3,5-trimethoxybenzene as an internal standard (76% yield). The crude was purified by preparative thin layer chromatography, providing the title compound as a colorless oil.

$\mathbf{R_f}$ = 0.67 (Hex)

^{1}H NMR (600 MHz, CDCl$_3$) δ 7.29 – 7.26 (m, 2H), 7.19 – 7.16 (m, 3H), 2.61 (t, *J* = 7.8 Hz, 2H), 1.64 – 1.59 (m, 2H), 1.36 – 1.28 (m, 6H), 0.89 (t, *J* = 7.2 Hz, 3H).

^{13}C NMR (151 MHz, CDCl$_3$) δ 143.0, 128.4, 128.2, 125.6, 36.0, 31.8, 31.5, 29.0, 22.6, 14.1.

HRMS (APCI) m/z calcd. for $C_{12}H_{18}$ $[M+H]^+$ **163.1481** found: **163.1479**.

Heptyl benzoate (2)

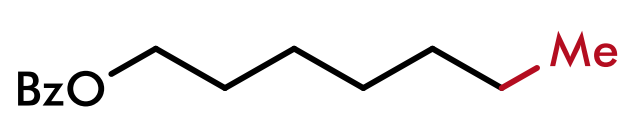

2

Prepared according to the general procedure A using $Fe(NO_3)_3 \cdot 9H_2O$ (6.0 mg, 0.015 mmol, 15 mol%), Na_2CO_3 (1.1 mg, 0.01 mmol, 10 mol%), bis(2,4,6-triisopropylphenyl)disulfide (11.4 mg, 0.025 mmol, 25 mol%), malonic acid **MA-1** (47 mg, 0.4 mmol, 4.0 equiv.), and alkene **A2** (19 mg, 0.1 mmol, 1.0 equiv.). The crude was purified by preparative thin layer chromatography, providing the title compound as a colorless oil (15.4 mg, 70% yield).

$\mathbf{R_f}$ = 0.65 (Hex/EtOAc = 2/0.5)

^{1}H NMR (600 MHz, CDCl$_3$) δ 8.05 – 8.04 (m, 2H), 7.56 – 7.54 (m, 1H), 7.44 (t, *J* = 7.8 Hz, 2H), 4.32 (t, *J* = 6.7 Hz, 2H), 1.78 – 1.74 (m, 2H), 1.45 – 1.42 (m, 2H), 1.39 – 1.26 (m, 6H), 0.89 (t, *J*

= 6.8 Hz, 3H).

^{13}C NMR (151 MHz, $CDCl_3$) δ 166.7, 132.8, 130.6, 129.5, 128.3, 65.2, 31.8, 29.0, 28.8, 26.0, 22.6, 14.1.

HRMS (APCI) m/z calcd. for $C_{14}H_{20}O_2$ $[M+H]^+$ **221.1536** found: **221.1531**.

Heptyl 4-methoxybenzoate (3)

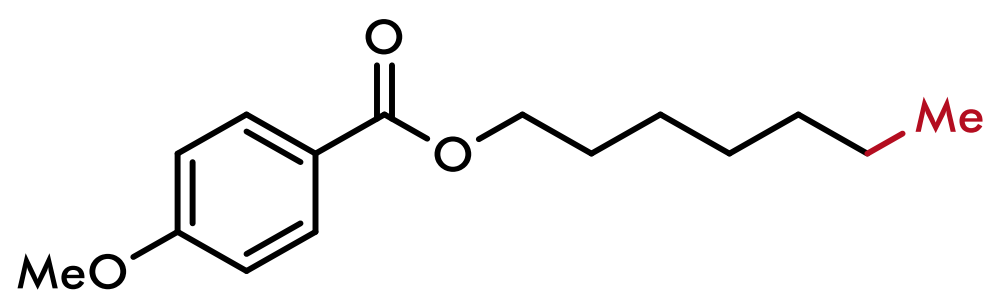

3

Prepared according to the general procedure A using $Fe(NO_3)_3 \cdot 9H_2O$ (6.0 mg, 0.015 mmol, 15 mol%), Na_2CO_3 (1.1 mg, 0.01 mmol, 10 mol%), 2,4,6-Triisopropylthiophenol (2.5 µL, 0.01 mmol, 10 mol%), methylmalonic acid **MA-1** (47 mg, 0.4 mmol, 4.0 equiv.), and alkene **A3** (22 mg, 0.1 mmol, 1.0 equiv.). The crude was purified by preparative thin layer chromatography, providing the title compound as a pale-yellow oil (15.0 mg, 60% yield).

R_f = 0.51 (Hex/EtOAc = 2/0.5)

^{1}H NMR (600 MHz, $CDCl_3$) δ 7.92 (d, *J* = 8.9 Hz, 2H), 6.84 (d, *J* = 8.9 Hz, 2H), 4.21 (t, *J* = 6.7 Hz, 2H), 3.79 (s, 3H), 1.70 – 1.65 (m, 2H), 1.38 – 1.15 (m, 8H), 0.81 (t, *J* = 7.0 Hz, 3H).

^{13}C NMR (151 MHz, $CDCl_3$) δ 166.5, 163.3, 131.6, 123.0, 113.6, 64.9, 55.4, 31.8, 29.0, 28.8, 26.1, 22.6, 14.1.

HRMS (APCI) m/z calcd. for $C_{15}H_{22}O_3$ $[M+H]^+$ **251.1642** found: **251.1634**.

Heptyl 4-(trifluoromethyl)benzoate (4)

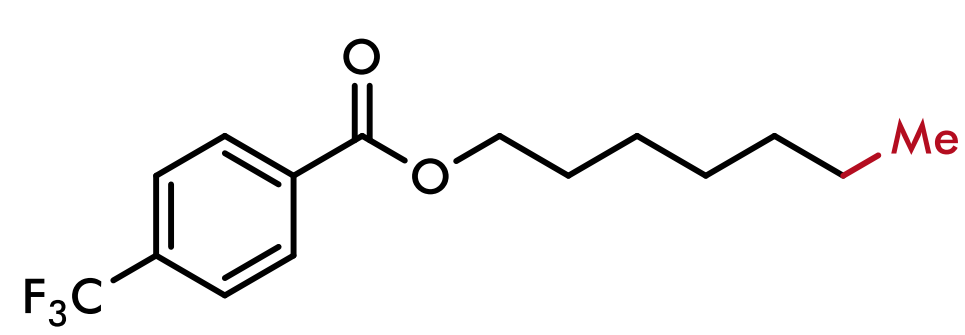

4

Prepared according to the general procedure A using $Fe(NO_3)_3 \cdot 9H_2O$ (6.0 mg, 0.015 mmol, 15 mol%), Na_2CO_3 (1.1 mg, 0.01 mmol, 10 mol%), bis(2,4,6-triisopropylphenyl)disulfide

(11.4 mg, 0.025 mmol, 25 mol%), methylmalonic acid **MA-1** (47 mg, 0.4 mmol, 4.0 equiv.), and alkene **A4** (25.8 mg, 0.1 mmol, 1.0 equiv.). The crude was purified by preparative thin layer chromatography, providing the title compound as a pale-yellow oil (14.1 mg, 49% yield).

R_f = 0.72 (Hex/EtOAc = 2/0.5)

^{1}H NMR (600 MHz, $CDCl_3$) δ 8.15 (d, *J* = 8.0 Hz, 2H), 7.70 (d, *J* = 8.0 Hz, 2H), 4.35 (t, *J* = 6.7 Hz, 2H), 1.80 – 1.76 (m, 2H), 1.46 – 1.41 (m, 2H), 1.40 – 1.27 (m, 6H), 0.89 (t, *J* = 6.9 Hz, 3H).

^{13}C NMR (151 MHz, $CDCl_3$) δ 165.5, (134.7, 134.4, 134.2, 134.0) (q, *J* = 32.6 Hz), 133.8, 129.9, (125.4, 125.4, 125.4, 125.3) (q, *J* = 3.8 Hz), (126.4, 124.6, 122.8, 121.0) (q, *J* = 272.9 Hz), 65.7, 31.7, 28.9, 28.7, 26.0, 22.6, 14.1.

^{19}F NMR (564 MHz, $CDCl_3$) δ –63.1

HRMS (APCI) m/z calcd. for $C_{15}H_{19}F_3O_2$ $[M+H]^+$ **289.1410** found: **289.0494**.

2-Hexylisoindoline-1,3-dione (5)

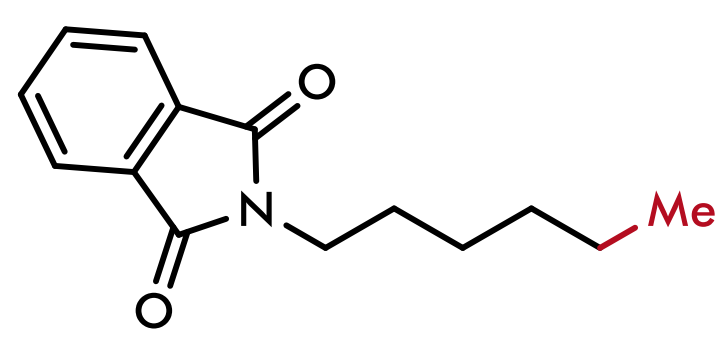

5

Prepared according to the general procedure A using $Fe(NO_3)_3 \cdot 9H_2O$ (6.0 mg, 0.015 mmol, 15 mol%), Na_2CO_3 (1.1 mg, 0.01 mmol, 10 mol%), bis(2,4,6-triisopropylphenyl)disulfide (11.4 mg, 0.025 mmol, 25 mol%), methylmalonic acid **MA-1** (47 mg, 0.4 mmol, 4.0 equiv.), and alkene **A5** (20.1 mg, 0.1 mmol, 1.0 equiv.). The crude was purified by preparative thin layer chromatography, providing the title compound as a white solid (13.0 mg, 56% yield).

R_f = 0.46 (Hex/EtOAc = 2/0.5)

^{1}H NMR (600 MHz, $CDCl_3$) δ 7.83 (dd, *J* = 5.4, 3.0 Hz, 2H), 7.70 (dd, *J* = 5.5, 3.0 Hz, 2H), 3.67 (t, *J* = 7.3 Hz, 2H), 1.70 – 1.63 (m, 2H), 1.36 – 1.26 (m, 6H), 0.87 (t, *J* = 7.2 Hz, 3H).

^{13}C NMR (151 MHz, $CDCl_3$) δ 168.5, 133.8, 132.2, 123.2, 123.1, 38.1, 31.4, 28.6, 26.5, 22.5, 14.0.

Mp = 34.2 – 34.9.

HRMS (APCI) m/z calcd. for $C_{14}H_{17}NO_2$ $[M+H]^+$ **232.1332** found: **232.1327**.

N-Hexylbenzenesulfonamide (6)

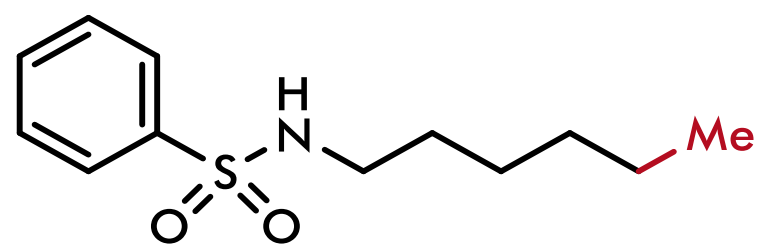

6

Prepared according to the general procedure A using $Fe(NO_3)_3 \cdot 9H_2O$ (6.0 mg, 0.015 mmol, 15 mol%), Na_2CO_3 (1.1 mg, 0.01 mmol, 10 mol%), bis(2,4,6-triisopropylphenyl)disulfide (11.4 mg, 0.025 mmol, 25 mol%), methylmalonic acid **MA-1** (47 mg, 0.4 mmol, 4.0 equiv.), and alkene **A6** (21.1 mg, 0.1 mmol, 1.0 equiv.). The crude was purified by preparative thin layer chromatography, providing the title compound as a colorless oil (10.7 mg, 50% yield).
$\mathbf{R_f}$ = 0.23 (Hex/EtOAc = 2/0.5)
^{1}H NMR (600 MHz, $CDCl_3$) δ 7.81 – 7.80 (m, 2H), 7.52 – 7.50 (m, 1H), 7.45 – 7.43 (m, 2H), 4.44 (t, *J* = 6.1 Hz, N–H), 2.88 (td, *J* = 7.1, 6.1 Hz, 2H), 1.40 – 1.35 (m, 2H), 1.20 – 1.09 (m, 6H), 0.77 (t, *J* = 7.1 Hz, 3H).
^{13}C NMR (151 MHz, $CDCl_3$) δ 140.0, 132.6, 129.1, 127.1, 43.3, 31.2, 29.6, 26.2, 22.5, 14.0.
HRMS (APCI) m/z calcd. for $C_{12}H_{19}NO_2S$ $[M+H]^+$ **242.1209** found: **242.1202**.

Hexyl(phenyl)sulfane (7)

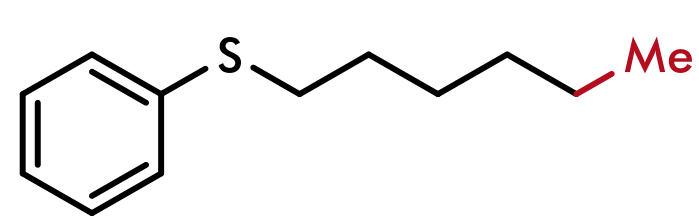

7

Prepared according to the general procedure A using $Fe(NO_3)_3 \cdot 9H_2O$ (6.0 mg, 0.015 mmol, 15 mol%), Na_2CO_3 (1.1 mg, 0.01 mmol, 10 mol%), bis(2,4,6-triisopropylphenyl)disulfide (11.4 mg, 0.025 mmol, 25 mol%), methylmalonic acid **MA-1** (47 mg, 0.4 mmol, 4.0 equiv.), and alkene **A7** (19.3 mg, 0.1 mmol, 1.0 equiv.). The crude was purified by preparative thin layer chromatography, providing the title compound as a colorless oil (9.7 mg, 50% yield).
$\mathbf{R_f}$ = 0.79 (Hex/EtOAc = 2/0.5)

^{1}H NMR (600 MHz, CDCl$_3$) δ 7.33 – 7.31 (m, 2H), 7.29 – 7.26 (m, 2H), 7.17 – 7.14 (m, 1H), 2.92 (t, *J* = 7.2 Hz, 2H), 1.67 – 1.62 (m, 2H), 1.45 – 1.40 (m, 2H), 1.34 – 1.24 (m, 4H), 0.88 (t, *J* = 7.2 Hz, 3H).
^{13}C NMR (151 MHz, CDCl$_3$) δ 137.1, 128.9, 128.8, 125.6, 33.6, 31.4, 29.1, 28.6, 22.6, 14.0.
HRMS (APCI) m/z calcd. for $C_{12}H_{18}S$ [M+H]$^+$ **195.1202** found: **195.1197**.

(Tetrahydro-2H-pyran-4-yl)methyl heptanoate (8)

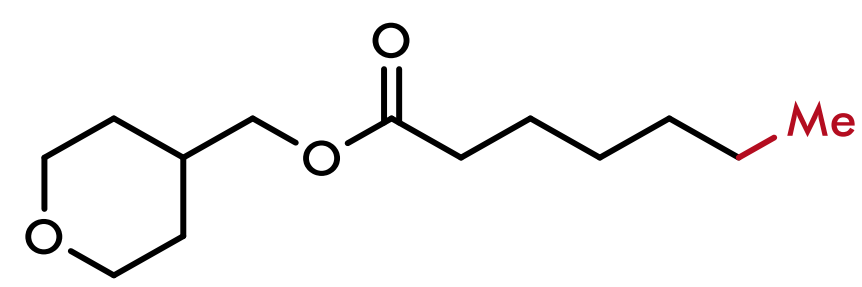

8

Prepared according to the general procedure A using $Fe(NO_3)_3 \cdot 9H_2O$ (6.0 mg, 0.015 mmol, 15 mol%), Na_2CO_3 (1.1 mg, 0.01 mmol, 10 mol%), bis(2,4,6-triisopropylphenyl)disulfide (11.4 mg, 0.025 mmol, 25 mol%), methylmalonic acid **MA-1** (47 mg, 0.4 mmol, 4.0 equiv.), and alkene **A8** (19.8 mg, 0.1 mmol, 1.0 equiv.). The crude was purified by preparative thin layer chromatography, providing the title compound as a pale-yellow oil (17.6 mg, 77% yield).
R$_f$ = 0.36 (Hex/EtOAc = 2/0.5)
^{1}H NMR (600 MHz, CDCl$_3$) δ 3.99 – 3.96 (m, 2H), 3.93 (d, *J* = 6.5 Hz, 2H), 3.38 (td, *J* = 11.8, 2.1 Hz, 2H), 2.30 (t, *J* = 7.5 Hz, 2H), 1.93 – 1.85 (m, 1H), 1.63 – 1.59 (m, 4H), 1.40 – 1.26 (m, 8H), 0.87 (t, *J* = 7.2 Hz, 3H).
^{13}C NMR (151 MHz, CDCl$_3$) δ 173.9, 68.5, 67.5, 34.6, 34.3, 31.4, 29.5, 28.8, 25.0, 22.5, 14.0.
HRMS (APCI) m/z calcd. for $C_{13}H_{24}O_3$ [M+H]$^+$ **229.1798** found: **229.1793**.

3-Methylhexyl benzoate (9)

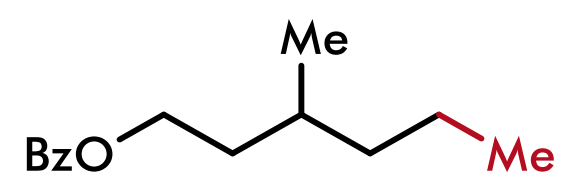

9

Prepared according to the general procedure A using $Fe(NO_3)_3 \cdot 9H_2O$ (6.0 mg, 0.015 mmol, 15 mol%), Na_2CO_3 (1.1 mg, 0.01 mmol, 10 mol%), bis(2,4,6-triisopropylphenyl)disulfide (11.4 mg, 0.025 mmol, 25 mol%), methylmalonic acid **MA-1** (47 mg, 0.4 mmol, 4.0 equiv.), and alkene **A9** (19 mg, 0.1 mmol, 1.0 equiv.). The crude was purified by preparative thin layer chromatography, providing the title compound as a pale-yellow oil (20.3 mg, 92% yield).

$\mathbf{R_f}$ = 0.65 (Hex/EtOAc = 2/0.5)

^{1}H NMR (600 MHz, CDCl$_3$) δ 8.05 – 8.03 (m, 2H), 7.56 – 7.53 (m, 1H), 7.45 – 7.42 (m, 2H), 4.40 – 4.33 (m, 2H), 1.83 – 1.78 (m, 1H), 1.68 – 1.63 (m, 1H), 1.60 – 1.55 (m, 1H), 1.41 – 1.27 (m, 3H), 1.23 – 1.17 (m, 1H), 0.96 (d, *J* = 6.6 Hz, 3H), 0.90 (t, *J* = 7.0 Hz, 3H).

^{13}C NMR (151 MHz, CDCl$_3$) δ 166.5, 132.5, 130.3, 129.3, 129.1, 128.2, 128.1, 63.3, 39.0, 35.3, 29.2, 19.8, 19.3, 14.0.

HRMS (APCI) m/z calcd. for $C_{14}H_{20}O_2$ $[M+H]^+$ **221.1536** found: **221.1532**.

3-Methylhexan-1-ol (10)

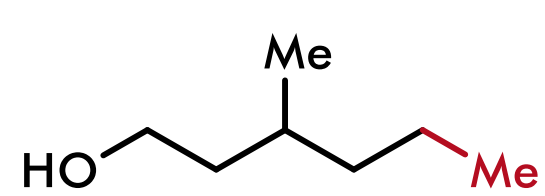

10

Prepared according to the general procedure A using $Fe(NO_3)_3 \cdot 9H_2O$ (6.0 mg, 0.015 mmol, 15 mol%), Na_2CO_3 (1.1 mg, 0.01 mmol, 10 mol%), bis(2,4,6-triisopropylphenyl)disulfide (11.4 mg, 0.025 mmol, 25 mol%), methylmalonic acid **MA-1** (47 mg, 0.4 mmol, 4.0 equiv.), and alkene **A10** (10 μL, 0.1 mmol, 1.0 equiv.). Yield was determined by ^{1}H NMR analysis of the crude reaction mixture with CH_2Br_2 as an internal standard (92% yield). The crude was purified by flash column chromatography, providing the title compound as a colorless oil.

$\mathbf{R_f}$ = 0.29 (Hex/EtOAc = 2/0.5)

^{1}H NMR (600 MHz, $CDCl_3$) δ 3.72 – 3.64 (m, 2H), 1.63 – 1.53 (m, 2H), 1.45 – 1.19 (m, 4H), 1.16 – 1.11 (m, 1H), 0.89 – 0.87 (m, 6H).

^{13}C NMR (151 MHz, $CDCl_3$) δ 61.3, 40.0, 39.4, 29.2, 20.0, 19.6, 14.3.

HRMS (APCI) m/z calcd. for $C_7H_{16}O$ $[M–H_2O+H]^+$ **99.1168** found: **99.0808**.

1-(*tert*-Butyl) 2-heptyl pyrrolidine-1,2-dicarboxylate (11)

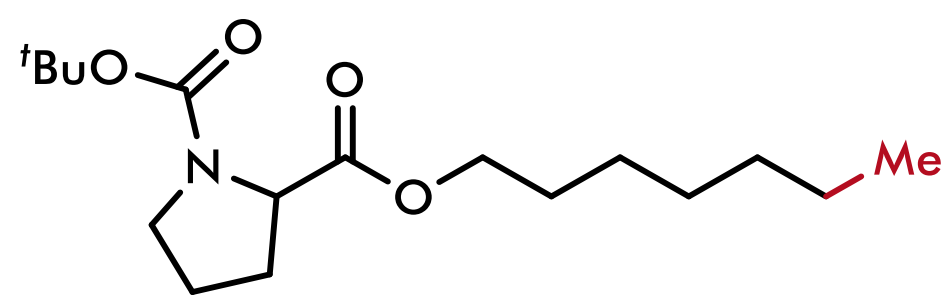

11

Prepared according to the general procedure A using $Fe(NO_3)_3 \cdot 9H_2O$ (6.0 mg, 0.015 mmol, 15 mol%), Na_2CO_3 (1.1 mg, 0.01 mmol, 10 mol%), 2,4,6-Triisopropylthiophenol (2.5 µL, 0.01 mmol, 10 mol%), methylmalonic acid **MA-1** (47 mg, 0.4 mmol, 4.0 equiv.), and alkene **A11** (28.3 mg, 0.1 mmol, 1.0 equiv.). The crude was purified by preparative thin layer chromatography, providing the title compound as a colorless oil (10 mg, 32% yield). On the other hand, if prepared according to the revised procedure (Supplementary Table 12), **11** was obtained in 51% yield, 16.8 mg).

R_f = 0.39 (Hex/EtOAc = 2/0.5)

^{1}H NMR (600 MHz, $CDCl_3$) mixture of rotamers: δ 4.32 – 4.06 (m, 3H), 3.57 – 3.35 (m, 2H), 2.24 – 2.15 (m, 1H), 1.98 – 1.82 (m, 3H), 1.65 – 1.59 (m, 2H), 1.45 (s, 3H), 1.41 (s, 6H), 1.35 – 1.21 (m, 8H), 0.88 (t, *J* = 6.8 Hz, 3H).

^{13}C NMR (151 MHz, $CDCl_3$) mixture of rotamers: δ 173.4, 173.1, 154.4, 153.9, 79.8, 79.7, 65.1, 65.1, 59.2, 59.0, 46.4, 46.3, 31.7, 31.0, 30.0, 29.7, 28.9, 28.9, 28.7, 28.6, 28.5, 28.3, 25.9, 25.8, 24.3, 23.6, 22.6, 14.1.

HRMS (APCI) m/z calcd. for $C_{17}H_{31}NO_4$ $[M+H]^+$ **214.1802** found: **214.1794**.

Heptyl 1-methyl-1H-pyrrole-2-carboxylate (12)

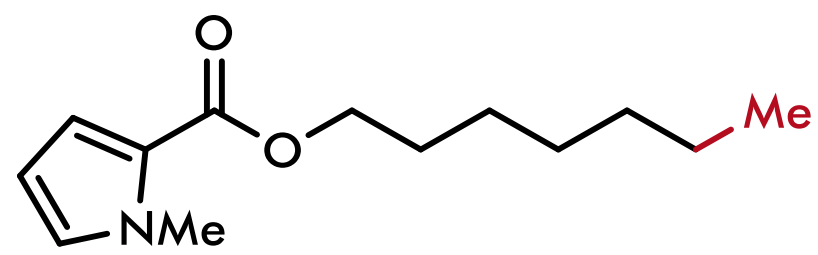

12

Prepared according to the general procedure A using $Fe(NO_3)_3 \cdot 9H_2O$ (6.0 mg, 0.015 mmol, 15 mol%), Na_2CO_3 (1.1 mg, 0.01 mmol, 10 mol%), bis(2,4,6-triisopropylphenyl)disulfide (11.4 mg, 0.025 mmol, 25 mol%), methylmalonic acid **MA-1** (35.4 mg, 0.3 mmol, 3.0 equiv.), and alkene **A12** (19.3 mg, 0.1 mmol, 1.0 equiv.). The crude was purified by preparative thin layer chromatography, providing the title compound as a pale-yellow oil (8.9 mg, 40% yield). On the other hand, if prepared according to the revised procedure (Supplementary Table 12), **12** was obtained in 65% yield, 15.6 mg).

R_f = 0.57 (Hex/EtOAc = 2/0.5)

^{1}H NMR (600 MHz, CDCl$_3$) δ 6.94 (dd, *J* = 4.0, 1.8 Hz, 1H), 6.77 (t, *J* = 2.2 Hz, 1H), 6.11 (dd, *J* = 3.9, 2.5 Hz, 1H), 4.21 (t, *J* = 6.7 Hz, 2H), 3.92 (s, 3H), 1.74 – 1.69 (m, 2H), 1.43 – 1.25 (m, 8H), 0.89 (t, *J* = 6.9 Hz, 3H).

^{13}C NMR (151 MHz, CDCl$_3$) δ 161.5, 129.3, 122.8, 117.6, 107.8, 64.0, 36.8, 31.8, 29.0, 28.9, 26.0, 22.6, 14.1.

HRMS (APCI) m/z calcd. for $C_{13}H_{21}NO_2$ $[M+H]^+$ **224.1645** found: **224.1640**.

Heptyl 2-(2,3-dihydrobenzofuran-5-yl)acetate (13)

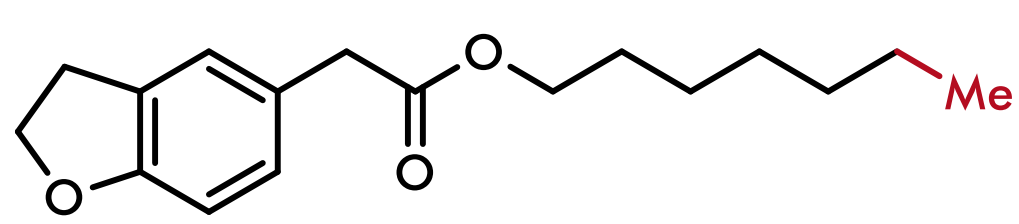

13

Prepared according to the general procedure A using $Fe(NO_3)_3 \cdot 9H_2O$ (6.0 mg, 0.015 mmol, 15 mol%), Na_2CO_3 (1.1 mg, 0.01 mmol, 10 mol%), bis(2,4,6-triisopropylphenyl)disulfide (11.4 mg, 0.025 mmol, 25 mol%), methylmalonic acid **MA-1** (47 mg, 0.4 mmol, 4.0 equiv.), and alkene **A13** (24.6 mg, 0.1 mmol, 1.0 equiv.). The crude was purified by preparative thin layer chromatography, providing the title compound as a pale-yellow oil (12.7 mg, 46%

yield).

R$_f$ = 0.47 (Hex/EtOAc = 2/0.5)

^{1}H NMR (600 MHz, CDCl$_3$) δ 7.12 (d, *J* = 2.0 Hz, 1H), 7.00 (dd, *J* = 8.2, 1.9 Hz, 1H), 6.72 (d, *J* = 8.1 Hz, 1H), 4.55 (t, *J* = 8.7 Hz, 2H), 4.07 (t, *J* = 6.7 Hz, 2H), 3.53 (s, 2H), 3.19 (t, *J* = 8.7 Hz, 2H), 1.63 – 1.59 (m, 2H), 1.33 – 1.23 (m, 8H), 0.88 (t, *J* = 7.0 Hz, 3H).

^{13}C NMR (151 MHz, CDCl$_3$) δ 172.2, 159.2, 128.9, 127.3, 126.1, 125.8, 109.2, 71.3, 65.0, 40.8, 31.7, 29.7, 28.9, 28.6, 25.8, 22.6, 14.1.

HRMS (APCI) m/z calcd. for $C_{17}H_{24}O_3$ $[M+H]^+$ **277.1798** found: **277.1791**.

1-(4-Phenylpiperazin-1-yl)heptan-1-one (14)

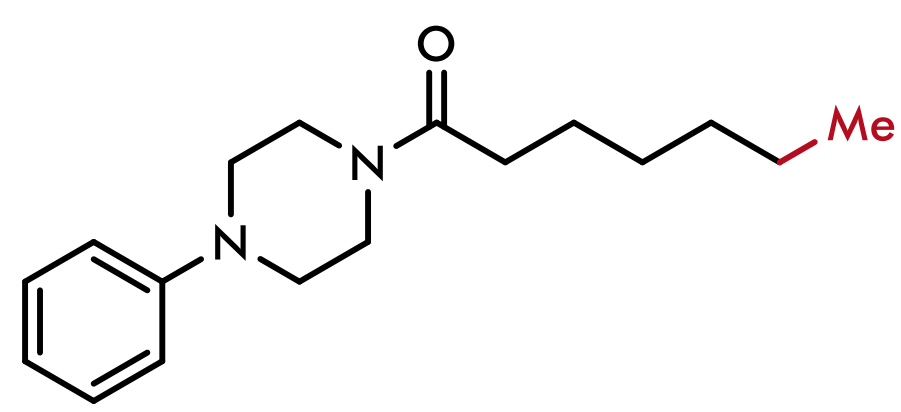

14

Prepared according to the general procedure A using $Fe(NO_3)_3 \cdot 9H_2O$ (6.0 mg, 0.015 mmol, 15 mol%), Na_2CO_3 (1.1 mg, 0.01 mmol, 10 mol%), bis(2,4,6-triisopropylphenyl)disulfide (11.4 mg, 0.025 mmol, 25 mol%), methylmalonic acid **MA-1** (47 mg, 0.4 mmol, 4.0 equiv.), and alkene **A14** (24.4 mg, 0.1 mmol, 1.0 equiv.). The crude was purified by preparative thin layer chromatography, providing the title compound as a colorless crystal forming under low temperature (9.9 mg, 36% yield). On the other hand, if prepared according to the revised procedure (Supplementary Table 12), **14** was obtained in 50% yield, 14.5 mg).

R$_f$ = 0.16 (Hex/EtOAc = 2/0.5)

^{1}H NMR (600 MHz, CDCl$_3$) δ 7.30 – 7.25 (m, 2H), 6.95 – 6.89 (m, 3H), 3.78 (d, *J* = 6.1 Hz, 2H), 3.63 (d, *J* = 6.2 Hz, 2H), 3.18 – 3.13 (m, 4H), 2.39 – 2.33 (m, 2H), 1.68 – 1.59 (m, 2H), 1.39 – 1.22 (m, 6H), 0.91 – 0.87 (m, 3H).

^{13}C NMR (151 MHz, CDCl$_3$) δ 171.8, 151.0, 129.3, 120.5, 116.7, 49.9, 49.5, 45.6, 41.4, 33.4, 31.7, 29.2, 25.4, 22.6, 14.1.

Mp = 54.7 – 55.3.

HRMS (APCI) m/z calcd. for $C_{17}H_{26}N_2O$ $[M+H]^+$ **275.2118** found: **275.2110**.

3-Methylhexyl 1-benzoylpiperidine-4-carboxylate (15)

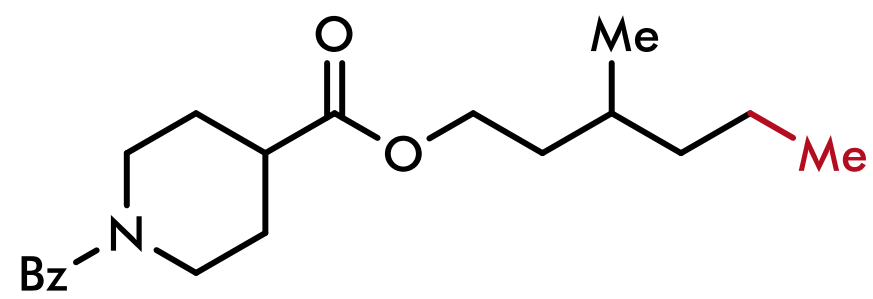

15

Prepared according to the general procedure A using $Fe(NO_3)_3 \cdot 9H_2O$ (6.0 mg, 0.015 mmol, 15 mol%), Na_2CO_3 (1.1 mg, 0.01 mmol, 10 mol%), bis(2,4,6-triisopropylphenyl)disulfide (11.4 mg, 0.025 mmol, 25 mol%), methylmalonic acid **MA-1** (47 mg, 0.4 mmol, 4.0 equiv.), and alkene **A15** (30.1 mg, 0.1 mmol, 1.0 equiv.). The crude was purified by preparative thin layer chromatography, providing the title compound as a yellow oil (12.6 mg, 38% yield). On the other hand, if prepared according to the revised procedure (Supplementary Table 12), **15** was obtained in 62% yield, 20.6 mg).

R_f = 0.12 (Hex/EtOAc = 2/0.5)

^{1}H NMR (600 MHz, $CDCl_3$) δ 7.41 – 7.38 (m, 5H), 4.53 (br s, 1H), 4.17 – 4.09 (m, 2H), 3.74 (br s, 1H), 3.03 (br s, 2H), 2.59 – 2.54 (m, 1H), 2.12 – 1.93 (br s, 1H), 1.92 – 1.20 (m, 9H), 1.16 – 1.11 (m, 1H), 0.91 – 0.86 (m, 6H).

^{13}C NMR (151 MHz, $CDCl_3$) δ 174.3, 170.5, 136.0, 129.6, 128.5, 126.8, 63.4, 63.3, 47.0, 41.5, 41.2, 39.1, 35.5, 29.7, 29.6, 28.6, 28.0, 20.0, 19.7, 19.5, 14.4, 14.3.

HRMS (APCI) m/z calcd. for $C_{20}H_{29}NO_3$ $[M+H]^+$ **332.2220** found: **332.2211**.

4,4,5,5-Tetramethyl-2-pentyl-1,3,2-dioxaborolane (16)

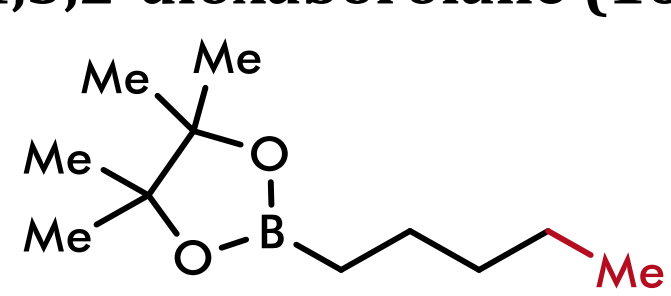

16

Prepared according to the general procedure A using $Fe(NO_3)_3 \cdot 9H_2O$ (6.0 mg, 0.015 mmol, 15 mol%), Na_2CO_3 (1.1 mg, 0.01 mmol, 10 mol%), 2,4,6-Triisopropylthiophenol (2.5 μL, 0.01 mmol, 10 mol%), methylmalonic acid **MA-1** (47 mg, 0.4 mmol, 4.0 equiv.), and alkene **A16** (18.8 μL, 0.1 mmol, 1.0 equiv.). The crude was purified by flash column chromatography, providing the title compound as a colorless oil (11.5 mg, 58% yield).

R_f = 0.65 (Hex/EtOAc = 2/0.5)

^{1}H NMR (600 MHz, CDCl$_3$) δ 1.43 – 1.37 (m, 2H), 1.31 – 1.23 (m, 16H), 0.87 (t, *J* = 7.0 Hz, 3H), 0.75 (t, *J* = 7.9 Hz, 2H).

^{13}C NMR (151 MHz, CDCl$_3$) δ 82.8, 34.7, 24.8, 23.7, 22.5, 14.1.

HRMS (APCI) m/z calcd. for $C_{11}H_{23}BO_2$ [M+H]$^+$ **199.1864** found: **199.1859**.

1-Bromododecane (17)

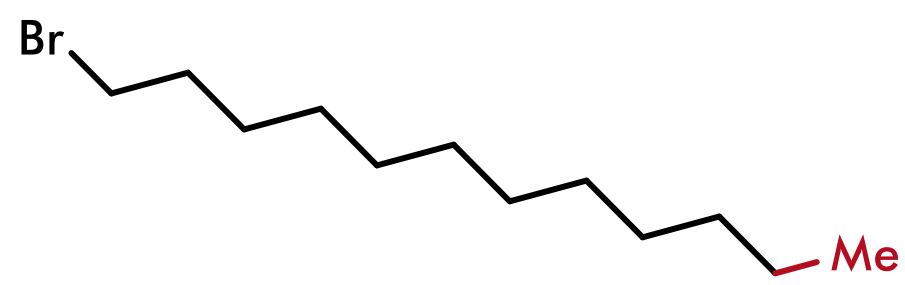

17

Prepared according to the general procedure A using $Fe(NO_3)_3 \cdot 9H_2O$ (6.0 mg, 0.015 mmol, 15 mol%), Na_2CO_3 (1.1 mg, 0.01 mmol, 10 mol%), bis(2,4,6-triisopropylphenyl)disulfide (11.4 mg, 0.025 mmol, 25 mol%), methylmalonic acid **MA-1** (47 mg, 0.4 mmol, 4.0 equiv.), and alkene **A17** (20 μL, 0.1 mmol, 1.0 equiv.) in MeCN/H_2O = 1/1 (0.05 M). Yield was determined by GC-FID analysis of the crude reaction mixture with cyclododecane as an internal standard (35% yield). The crude was purified by flash column chromatography, providing the title compound as a colorless oil.

R_f = 0.77 (Hex)

^{1}H NMR (600 MHz, CDCl$_3$) δ 3.41 (t, *J* = 6.9 Hz, 2H), 1.88 – 1.82 (m, 2H), 1.45 – 1.39 (m, 2H), 1.33 – 1.23 (m, 16H), 0.88 (t, *J* = 7.0 Hz, 3H).

^{13}C NMR (151 MHz, CDCl$_3$) δ 34.1, 32.9, 31.9, 29.6, 29.6, 29.6, 29.5, 29.4, 28.8, 28.2, 22.7, 14.1.

GCMS m/z calcd. for $C_{12}H_{25}Br$ [M]$^+$ **248.1140** found: **248**.

Dodecane (18)

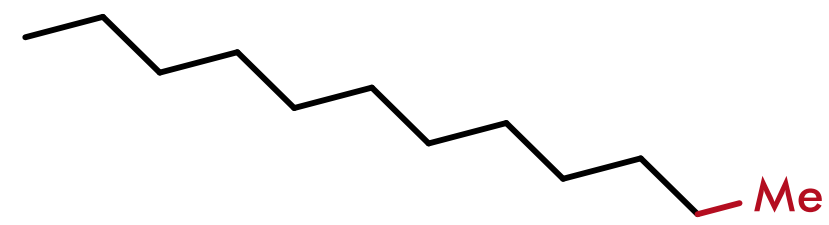

18

Prepared according to the general procedure B using $Fe(NO_3)_3 \cdot 9H_2O$ (6.0 mg, 0.015 mmol, 15 mol%), Na_2CO_3 (1.1 mg, 0.01 mmol, 10 mol%), bis(2,4,6-triisopropylphenyl)disulfide (11.4 mg, 0.025 mmol, 25 mol%), methylmalonic acid **MA-1** (35.4 mg, 0.3 mmol, 3.0 equiv.), and alkene **A18** (18.4 mg, 0.1 mmol, 1.0 equiv.). Yield was determined by GC-FID analysis of the crude reaction mixture with cyclododecane as an internal standard (49% yield). The crude was purified by flash column chromatography, providing the title compound as a colorless oil.

R$_f$ = 0.8 (Hex)

^{1}H NMR (600 MHz, CDCl$_3$) δ 1.33 – 1.19 (m, 20H), 0.88 (t, J = 7.0 Hz, 6H).

^{13}C NMR (151 MHz, CDCl$_3$) δ 32.0, 29.7, 29.7, 29.4, 29.4, 22.7, 14.1.

GCMS m/z calcd. for $C_{12}H_{26}$ $[M]^+$ **170.2035** found: **170**.

1-Chlorotridecane (19)

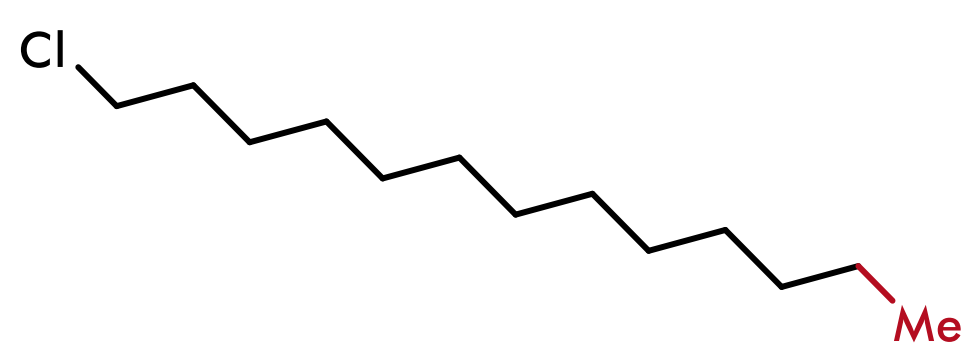

19

Prepared according to the general procedure A using $Fe(NO_3)_3 \cdot 9H_2O$ (6.0 mg, 0.015 mmol, 15 mol%), Na_2CO_3 (1.1 mg, 0.01 mmol, 10 mol%), bis(2,4,6-triisopropylphenyl)disulfide (11.4 mg, 0.025 mmol, 25 mol%), methylmalonic acid **MA-1** (47 mg, 0.4 mmol, 4.0 equiv.), and alkene **A17** (20 μL, 0.1 mmol, 1.0 equiv.) in $MeCN/H_2O$ = 1/1 (0.05 M). Yield was determined by GC-FID analysis of the crude reaction mixture with cyclododecane as an internal standard (31% yield). The crude was purified by flash column chromatography, providing the title compound as a colorless oil.

R$_f$ = 0.73 (Hex)

^{1}H NMR (600 MHz, $CDCl_3$) δ 3.53 (t, *J* = 6.8 Hz, 2H), 1.77 (p, *J* = 7.0 Hz, 2H), 1.41 (q, *J* = 7.3 Hz, 2H), 1.31 – 1.22 (m, 18H), 0.88 (t, *J* = 6.9 Hz, 3H).
^{13}C NMR (151 MHz, $CDCl_3$) δ 45.2, 32.7, 31.9, 29.7, 29.7, 29.7, 29.6, 29.6, 29.6, 29.5, 29.5, 29.4, 28.9, 26.9, 22.7, 14.1.
GCMS m/z calcd. for $C_{13}H_{27}Cl$ $[M]^+$ **218.1801** found: **218**.

Undecane (20)

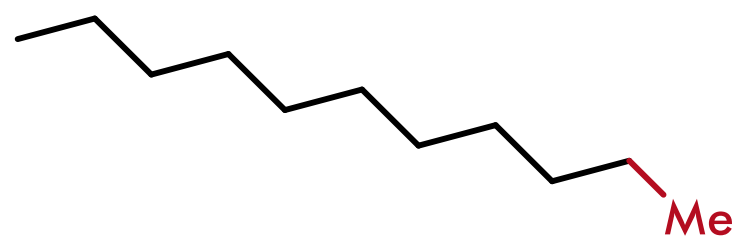

20

Prepared according to the general procedure A using $Fe(NO_3)_3 \cdot 9H_2O$ (10 mg, 0.025 mmol, 25 mol%), Na_2CO_3 (1.1 mg, 0.01 mmol, 10 mol%), bis(2,4,6-triisopropylphenyl)disulfide (11.4 mg, 0.025 mmol, 25 mol%), methylmalonic acid **MA-1** (70.8 mg, 0.6 mmol, 6.0 equiv.), and alkene **A20** (17.3 μL, 0.1 mmol, 1.0 equiv.). The reaction was stirred under irradiation for 48 hours. Yield was determined by GC-FID analysis of the crude reaction mixture with cyclododecane as an internal standard (18% yield).
GCMS m/z calcd. for $C_{11}H_{24}$ $[M]^+$ **156.1878** found: **156**.

(Butylsulfonyl)benzene (21)

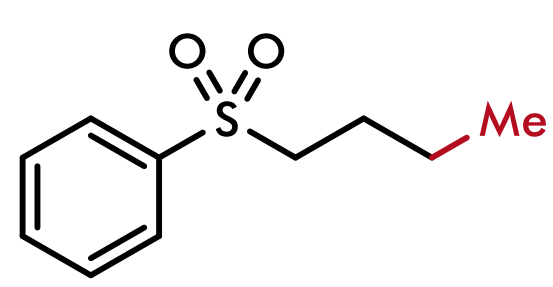

21

Prepared according to the general procedure A using $Fe(NO_3)_3 \cdot 9H_2O$ (6.0 mg, 0.015 mmol, 15 mol%), Na_2CO_3 (1.1 mg, 0.01 mmol, 10 mol%), 2,4,6-Triisopropylthiophenol (2.5 μL, 0.01 mmol, 10 mol%), methylmalonic acid **MA-1** (47 mg, 0.4 mmol, 4.0 equiv.), and alkene **A21** (16.8 mg, 0.1 mmol, 1.0 equiv.). The crude was purified by preparative thin layer chromatography, providing the title compound as a yellow oil (13.1 mg, 66% yield).

R_f = 0.27 (Hex/EtOAc = 2/0.5)

^{1}H NMR (600 MHz, $CDCl_3$) δ 7.91 (dd, *J* = 8.4, 1.3 Hz, 2H), 7.67 – 7.64 (m, 1H), 7.59 – 7.56 (m, 2H), 3.10 – 3.07 (m, 2H), 1.72 – 1.66 (m, 2H), 1.39 (h, *J* = 7.4 Hz, 2H), 0.89 (t, *J* = 7.4 Hz, 3H).

^{13}C NMR (151 MHz, $CDCl_3$) δ 139.3, 133.6, 129.3, 128.1, 56.1, 24.6, 21.6, 13.5.

HRMS (APCI) m/z calcd. for $C_{10}H_{14}O_2S$ $[M+H]^+$ **199.0787** found: **199.0782**.

2-Fluoropentyl benzoate (22)

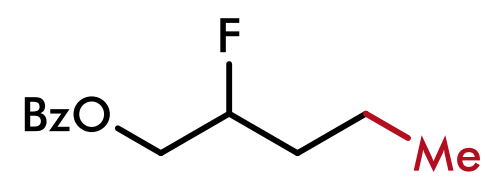

22

Prepared according to the general procedure A using $Fe(NO_3)_3 \cdot 9H_2O$ (6.0 mg, 0.015 mmol, 15 mol%), Na_2CO_3 (1.1 mg, 0.01 mmol, 10 mol%), 2,4,6-Triisopropylthiophenol (2.5 µL, 0.01 mmol, 10 mol%), methylmalonic acid **MA-1** (47 mg, 0.4 mmol, 4.0 equiv.), and alkene **A31** (18 mg, 0.1 mmol, 1.0 equiv.). The crude was purified by preparative thin layer chromatography, providing the title compound as a colorless oil (6 mg, 29% yield). On the other hand, if prepared according to the revised procedure (Supplementary Table 12), **22** was obtained in 36% yield, 7.6 mg).

R_f = 0.28 (Hex/EtOAc = 2/0.1)

^{1}H NMR (600 MHz, $CDCl_3$) δ 8.08 – 8.07 (m, 2H), 7.59 – 7.56 (m, 1H), 7.47 – 7.44 (m, 2H), 4.88 – 4.76 (m, 1H), 4.51 – 4.35 (m, 2H), 1.83 – 1.74 (m, 1H), 1.69 – 1.45 (m, 3H), 0.99 (t, *J* = 7.4 Hz, 3H).

^{13}C NMR (151 MHz, $CDCl_3$) δ 166.4, 133.2, 129.8, 129.7, 128.4, 91.8, 90.6, 66.3 (d, *J* = 22.4 Hz), 33.5 (d, *J* = 20.4 Hz), 18.2 (d, *J* = 4.6 Hz), 13.8.

^{19}F NMR (564 MHz, $CDCl_3$) δ –187.20 (m).

HRMS (ESI) m/z calcd. for $C_{12}H_{15}FO_2$ $[M+H]^+$ **211.1129** found: **211.1127**.

2-Chloropentyl benzoate (23)

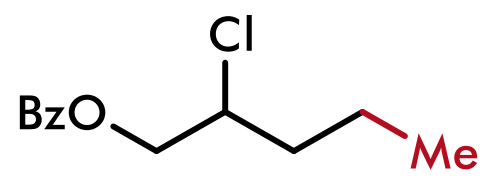

23

Prepared according to the general procedure A using $Fe(NO_3)_3 \cdot 9H_2O$ (6.0 mg, 0.015 mmol, 15 mol%), Na_2CO_3 (1.1 mg, 0.01 mmol, 10 mol%), 2,4,6-Triisopropylthiophenol (2.5 µL, 0.01 mmol, 10 mol%), methylmalonic acid **MA-1** (47 mg, 0.4 mmol, 4.0 equiv.), and alkene **A32** (19.7 mg, 0.1 mmol, 1.0 equiv.). The crude was purified by preparative thin layer chromatography, providing the title compound as a colorless oil (11.5 mg, 51% yield).

$\mathbf{R_f}$ = 0.61 (Hex/EtOAc = 2/0.1)

^{1}H NMR (600 MHz, $CDCl_3$) δ 8.19 – 7.82 (m, 2H), 7.73 – 7.52 (m, 1H), 7.46 (t, *J* = 7.8 Hz, 2H), 4.68 – 4.40 (m, 2H), 4.21 (ddt, *J* = 9.3, 6.6, 4.8 Hz, 1H), 1.96 – 1.71 (m, 2H), 1.73 – 1.38 (m, 3H), 0.97 (t, *J* = 7.4 Hz, 3H).

^{13}C NMR (151 MHz, $CDCl_3$) δ 166.2, 133.3, 129.8, 129.7, 128.5, 68.1, 59.1, 36.8, 19.4, 13.5.

HRMS (ESI) m/z calcd. for $C_{12}H_{15}ClO_2$ $[M+H]^+$ **227.0833** found: **227.0830**.

Butoxybenzene (24)

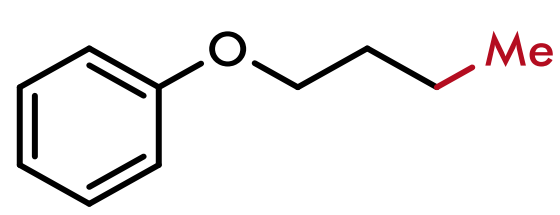

24

Prepared according to the general procedure A using $Fe(NO_3)_3 \cdot 9H_2O$ (6.0 mg, 0.015 mmol, 15 mol%), Na_2CO_3 (1.1 mg, 0.01 mmol, 10 mol%), 2,4,6-Triisopropylthiophenol (2.5 µL, 0.01 mmol, 10 mol%), methylmalonic acid **MA-1** (35 mg, 0.3 mmol, 3.0 equiv.), and alkene **A33** (12 mg, 0.1 mmol, 1.0 equiv.). The crude was purified by preparative thin layer chromatography, providing the title compound as a colorless oil (72% NMR yield). The spectroscopic data of **24** is in accordance with literature.[8]

$\mathbf{R_f}$ = 0.89 (Hex/EtOAc = 2/0.1)

^{1}H NMR (600 MHz, $CDCl_3$) δ 7.44 – 7.11 (m, 2H), 7.02 – 6.67 (m, 3H), 3.97 (t, *J* = 6.5 Hz, 2H), 2.03 – 1.68 (m, 2H), 1.63 – 1.39 (m, 2H), 0.98 (t, *J* = 7.4 Hz, 3H).

^{13}C NMR (151 MHz, $CDCl_3$) δ 159.1, 129.4, 120.4, 114.5, 67.6, 31.4, 19.3, 13.9.

1-Butylpyrrolidin-2-one (25)

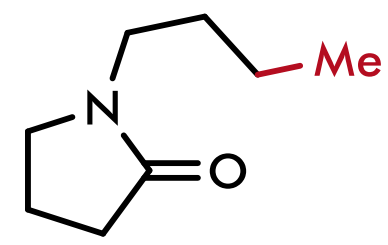

25

Prepared according to the general procedure A using $Fe(OAc)_2$ (0.87 mg, 0.005 mmol, 5 mol%), Na_2CO_3 (1.1 mg, 0.01 mmol, 10 mol%), 2,4,6-Triisopropylthiophenol (5.0 μL, 0.02 mmol, 20 mol%), methylmalonic acid **MA-1** (47 mg, 0.4 mmol, 3.0 equiv.), and alkene **A34** (11 mg, 0.1 mmol, 1.0 equiv.). The crude was purified by preparative thin layer chromatography, providing the title compound as a colorless oil (40% NMR yield).

R_f = 0.26 (Hex/EtOAc = 1/1)

^{1}H NMR (600 MHz, $CDCl_3$) δ 3.48 – 3.33 (m, 2H), 3.27 (t, *J* = 7.4 Hz, 2H), 2.38 (t, *J* = 8.1 Hz, 2H), 2.11 – 1.85 (m, 2H), 1.58 – 1.42 (m, 2H), 1.32 (dq, *J* = 14.8, 7.4 Hz, 2H), 0.93 (t, *J* = 7.4 Hz, 3H).

^{13}C NMR (151 MHz, $CDCl_3$) δ 174.9, 47.1, 42.3, 31.1, 29.4, 20.1, 18.0, 13.8.

HRMS (ESI) m/z calcd. for $C_8H_{15}NO$ $[M+H]^+$ **142.1226** found: **142.1225**.

5-Butyl-3-(3,5-dichlorophenyl)-5-methyloxazolidine-2,4-dione (26)

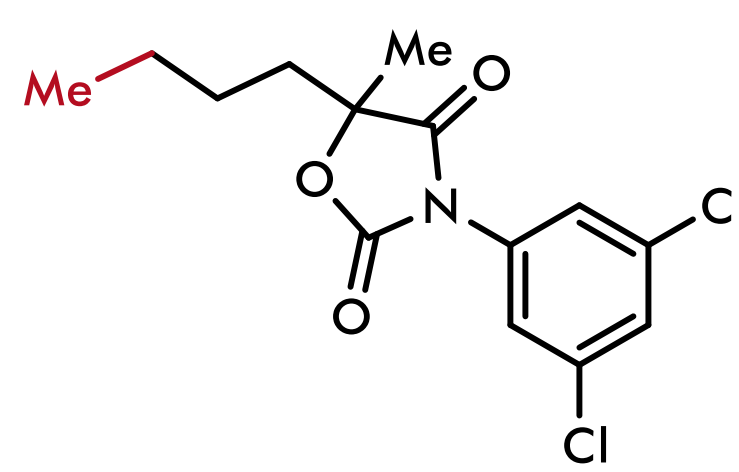

26

Prepared according to the general procedure A using $Fe(NO_3)_3 \cdot 9H_2O$ (6.0 mg, 0.015 mmol, 15 mol%), Na_2CO_3 (1.1 mg, 0.01 mmol, 10 mol%), bis(2,4,6-triisopropylphenyl)disulfide (11.4 mg, 0.025 mmol, 25 mol%), methylmalonic acid **MA-1** (47 mg, 0.4 mmol, 4.0 equiv.), and alkene **A22** (28.6 mg, 0.1 mmol, 1.0 equiv.) in $MeCN/H_2O/EtOAc$ (0.5 mL/0.5 mL/0.05 mL). The crude was purified by preparative thin layer chromatography, providing the title compound as a colorless oil (13.6 mg, 43% yield).

R_f = 0.53 (Hex/EtOAc = 2/0.5)

^{1}H NMR (600 MHz, CDCl$_3$) δ 7.45 (s, 2H), 7.41 (s, 1H), 1.95 (tt, *J* = 14.4, 7.3 Hz, 2H), 1.65 (s, 3H), 1.50 – 1.42 (m, 1H), 1.37 (h, *J* = 7.2 Hz, 2H), 1.30 – 1.19 (m, 1H), 0.92 (t, *J* = 7.3 Hz, 3H).
^{13}C NMR (151 MHz, CDCl$_3$) δ 173.9, 152.7, 135.7, 132.8, 129.1, 123.9, 86.5, 36.9, 25.1, 22.7, 22.5, 13.9.
HRMS (APCI) m/z calcd. for $C_{14}H_{15}C_{12}NO_3$ $[M+H]^+$ **316.0502** found: **316.0491**.

2-Methoxy-4-pentylphenol (27)

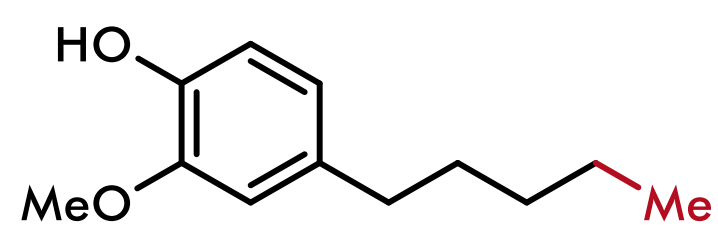

27

Prepared according to the general procedure A using $Fe(NO_3)_3 \cdot 9H_2O$ (6.0 mg, 0.015 mmol, 15 mol%), Na_2CO_3 (1.1 mg, 0.01 mmol, 10 mol%), 2,4,6-Triisopropylthiophenol (2.5 μL, 0.01 mmol, 10 mol%), methylmalonic acid **MA-1** (47 mg, 0.4 mmol, 4.0 equiv.), and alkene **A23** (16.4 mg, 0.1 mmol, 1.0 equiv.). The crude was purified by preparative thin layer chromatography, providing the title compound as a pale-yellow oil (7.2 mg, 37% yield). On the other hand, if prepared according to the revised procedure (Supplementary Table 12), **27** was obtained in 60% yield, 11.7 mg).
R_f = 0.37 (Hex/EtOAc = 2/0.5)
^{1}H NMR (600 MHz, CDCl$_3$) δ 6.83 (d, *J* = 7.7 Hz, 1H), 6.68 – 6.67 (m, 2H), 5.45 (s, 1H), 3.88 (s, 3H), 2.53 (t, *J* = 7.8 Hz, 2H), 1.61 – 1.56 (m, 2H), 1.38 – 1.28 (m, 4H), 0.90 (t, *J* = 6.9 Hz, 3H).
^{13}C NMR (151 MHz, CDCl$_3$) δ 146.3, 143.5, 135.0, 120.9, 114.1, 111.0, 55.9, 35.7, 31.5, 22.6, 14.1.
HRMS (APCI) m/z calcd. for $C_{12}H_{18}O_2$ $[M+H]^+$ **195.1380** found: **195.1375**.

Heptyl 2-(6-methoxynaphthalen-2-yl)propanoate (28)

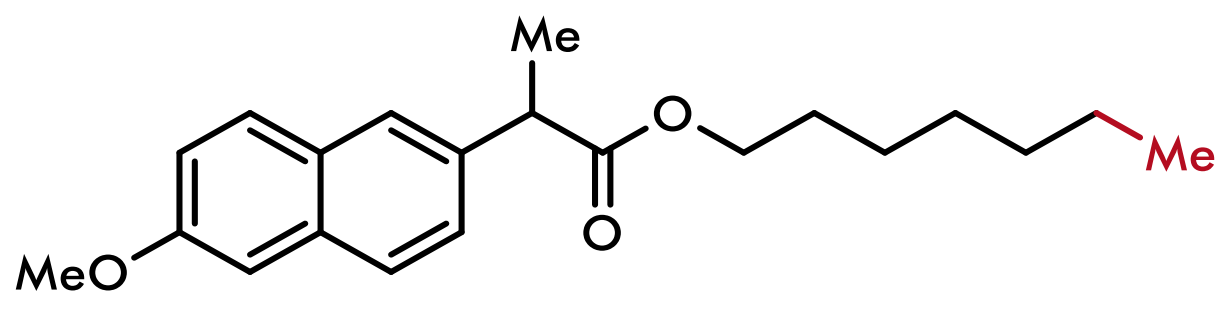

28

Prepared according to the general procedure A using $Fe(NO_3)_3 \cdot 9H_2O$ (6.0 mg, 0.015 mmol, 15 mol%), Na_2CO_3 (1.1 mg, 0.01 mmol, 10 mol%), bis(2,4,6-triisopropylphenyl)disulfide (11.4 mg, 0.025 mmol, 25 mol%), methylmalonic acid **MA-1** (47 mg, 0.4 mmol, 4.0 equiv.), and alkene **A24** (29.8 mg, 0.1 mmol, 1.0 equiv.) in MeCN/H_2O/EtOAc (0.5 mL/0.5 mL/0.05 mL). The crude was purified by preparative thin layer chromatography, providing the title compound as a pale-yellow oil (13.4 mg, 41% yield).

$\mathbf{R_f}$ = 0.56 (Hex/EtOAc = 2/0.5)

^{1}H NMR (600 MHz, $CDCl_3$) δ 7.71 – 7.66 (m, 3H), 7.41 (dd, *J* = 8.5, 1.9 Hz, 1H), 7.15 – 7.10 (m, 2H), 4.06 (t, *J* = 6.7 Hz, 2H), 3.91 (s, 3H), 3.84 (q, *J* = 7.1 Hz, 1H), 1.58 – 1.53 (m, 3H), 1.28 – 1.13 (m, 10H), 0.84 (t, *J* = 7.1 Hz, 3H).

^{13}C NMR (151 MHz, $CDCl_3$) δ 174.8, 157.6, 135.9, 133.8, 129.3, 129.0, 127.1, 126.3, 125.9, 118.9, 105.6, 64.9, 55.3, 45.6, 31.7, 28.8, 28.6, 25.8, 22.5, 18.5, 14.1.

HRMS (APCI) m/z calcd. for $C_{21}H_{28}O_3$ $[M+H]^+$ **329.2111** found: **329.2101**.

Heptyl 2-(2,5-dichlorophenoxy)acetate (29)

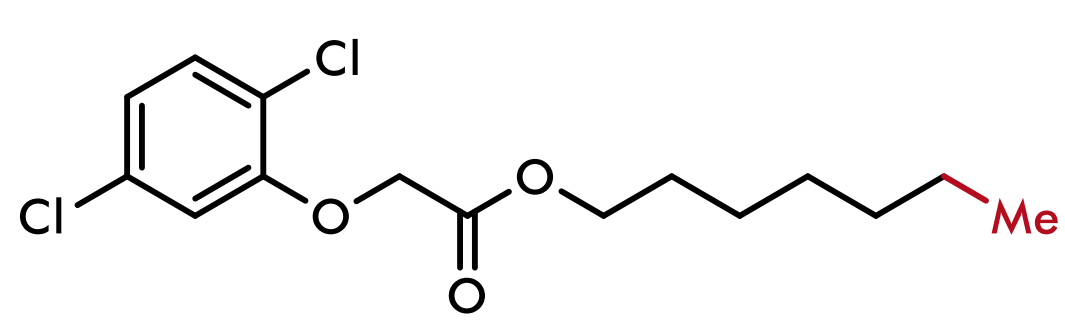

29

Prepared according to the general procedure A using $Fe(NO_3)_3 \cdot 9H_2O$ (6.0 mg, 0.015 mmol, 15 mol%), Na_2CO_3 (1.1 mg, 0.01 mmol, 10 mol%), bis(2,4,6-triisopropylphenyl)disulfide (11.4 mg, 0.025 mmol, 25 mol%), methylmalonic acid **MA-1** (47 mg, 0.4 mmol, 4.0 equiv.), and alkene **A25** (28.9 mg, 0.1 mmol, 1.0 equiv.). The crude was purified by preparative thin layer chromatography, providing the title compound as a yellow oil (19.2 mg, 60% yield).

R$_f$ = 0.54 (Hex/EtOAc = 2/0.5)

^{1}H NMR (600 MHz, CDCl$_3$) δ 7.39 (d, *J* = 2.5 Hz, 1H), 7.16 (dd, *J* = 8.8, 2.6 Hz, 1H), 6.77 (d, *J* = 8.8 Hz, 1H), 4.68 (s, 2H), 4.19 (t, *J* = 6.7 Hz, 2H), 1.67 – 1.60 (m, 2H), 1.32 – 1.20 (m, 8H), 0.88 (t, *J* = 7.1 Hz, 3H).

^{13}C NMR (151 MHz, CDCl$_3$) δ 168.2, 152.5, 130.4, 127.5, 127.1, 124.3, 114.7, 66.4, 65.7, 31.7, 28.8, 28.5, 25.7, 22.6, 14.1.

HRMS (APCI) m/z calcd. for $C_{15}H_{20}C_{12}O_3$ $[M+H]^+$ **319.0862** found: **319.0854**.

Heptyl 2-(4-((2-oxocyclopentyl)methyl)phenyl)propanoate (30)

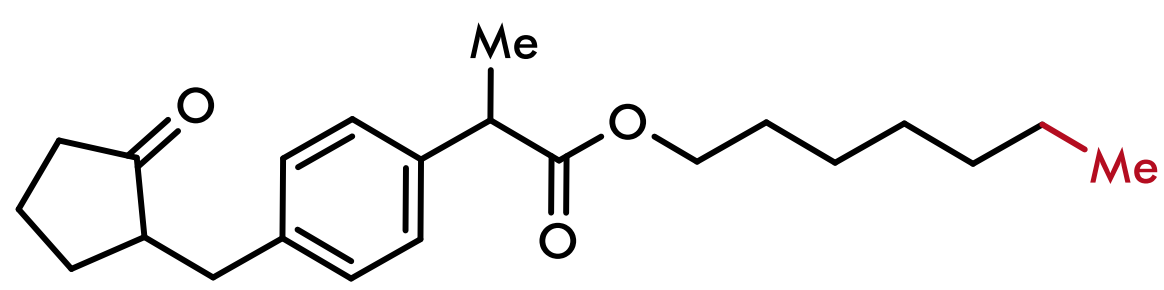

30

Prepared according to the general procedure A using $Fe(NO_3)_3 \cdot 9H_2O$ (6.0 mg, 0.015 mmol, 15 mol%), Na_2CO_3 (1.1 mg, 0.01 mmol, 10 mol%), 2,4,6-Triisopropylthiophenol (2.5 µL, 0.01 mmol, 10 mol%), methylmalonic acid **MA-1** (47 mg, 0.4 mmol, 4.0 equiv.), and alkene **A26** (31.4 mg, 0.1 mmol, 1.0 equiv.). The crude was purified by preparative thin layer chromatography, providing the title compound as a colorless oil (11 mg, 32% yield). On the other hand, if prepared according to the revised procedure (Supplementary Table 12), **30** was obtained in 71% yield, 24.5 mg).

R$_f$ = 0.52 (Hex/EtOAc = 2/0.5)

^{1}H NMR (600 MHz, CDCl$_3$) δ 7.21 (d, *J* = 8.1 Hz, 2H), 7.11 (d, *J* = 8.1 Hz, 2H), 4.05 (t, *J* = 6.7 Hz, 2H), 3.68 (q, *J* = 7.2 Hz, 1H), 3.12 (dd, *J* = 14.0, 4.1 Hz, 1H), 2.50 (dd, *J* = 13.9, 9.6 Hz, 1H), 2.39 – 2.29 (m, 2H), 2.14 – 2.03 (m, 2H), 1.98 – 1.92 (m, 1H), 1.77 – 1.68 (m, 1H), 1.6 – 1.47 (m, 3H), 1.48 (d, *J* = 7.2 Hz, 3H), 1.30 – 1.18 (m, 8H), 0.87 (t, *J* = 7.1 Hz, 3H).

^{13}C NMR (151 MHz, CDCl$_3$) δ 220.2, 174.7, 138.8, 138.5, 129.1, 127.5, 64.9, 51.0, 45.2, 38.2, 35.2, 31.7, 29.2, 28.8, 28.5, 25.7, 22.6, 20.6, 18.5, 18.5, 14.1.

HRMS (APCI) m/z calcd. for $C_{22}H_{32}O_3$ $[M+H]^+$ **345.2424** found: **345.2417**.

(5R)-3-ethyl-2-methyl-5-((R)-pentan-2-yl)cyclohexan-1-one (31)

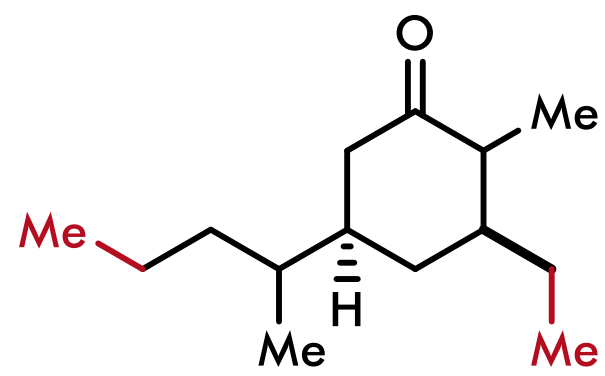

31

Prepared according to the general procedure A using $Fe(NO_3)_3 \cdot 9H_2O$ (6.0 mg, 0.015 mmol, 15 mol%), Na_2CO_3 (1.1 mg, 0.01 mmol, 10 mol%), 2,4,6-Triisopropylthiophenol (2.5 μL, 0.01 mmol, 10 mol%), methylmalonic acid **MA-1** (47 mg, 0.4 mmol, 4.0 equiv.), and alkene **A27** (15.6 μL, 0.1 mmol, 1.0 equiv.). The crude was purified by preparative thin layer chromatography, providing the title compound as a pale-yellow oil (9.7 mg, 46% yield).

R_f = 0.62 (Hex/EtOAc = 2/0.5)

^{1}H NMR (600 MHz, $CDCl_3$) mixture of four diastereomers: δ 2.39 – 2.03 (m, 3H), 1.86 – 1.16 (m, 11H), 1.15 – 0.83 (m, 12H).

^{13}C NMR (151 MHz, $CDCl_3$) δ 214.2, 214.1, 213.8, 213.6, 50.1, 50.1, 49.0, 48.9, 46.5, 45.7, 45.6, 45.6, 45.3, 45.1, 44.8, 44.6, 43.8, 43.1, 42.8, 37.3, 37.3, 37.2, 37.2, 36.3, 36.2, 36.2, 35.9, 34.6, 34.2, 33.0, 31.9, 31.1, 29.7, 29.6, 29.4, 27.7, 26.3, 23.9, 23.8, 22.8, 22.7, 20.5, 20.4, 20.4, 20.3, 15.9, 15.9, 15.7, 15.5, 14.4, 14.3, 14.3, 14.3, 11.4, 10.1, 10.0.

HRMS (APCI) m/z calcd. for $C_{14}H_{26}O$ $[M+H]^+$ **211.2056** found: **211.2051**.

(4R,4aS,6R)-4,4a-dimethyl-6-((R)-pentan-2-yl)-4,4a,5,6,7,8-hexahydronaphthalen-2(3H)-one (32)

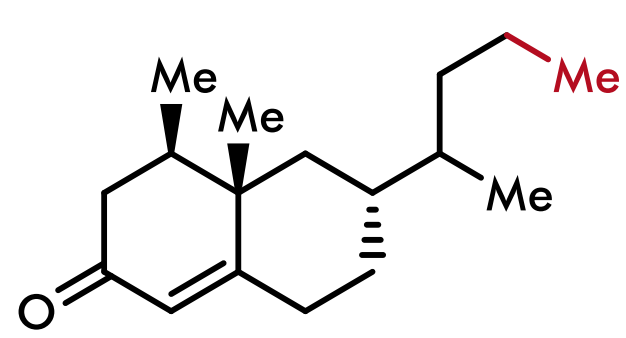

32

Prepared according to the general procedure A using $Fe(NO_3)_3 \cdot 9H_2O$ (6.0 mg, 0.015 mmol, 15 mol%), Na_2CO_3 (1.1 mg, 0.01 mmol, 10 mol%), bis(2,4,6-triisopropylphenyl)disulfide (11.4 mg, 0.025 mmol, 25 mol%), methylmalonic acid **MA-1** (47 mg, 0.4 mmol, 4.0 equiv.),

and alkene **A28** (22.6 µL, 0.1 mmol, 1.0 equiv.). The crude was purified by preparative thin layer chromatography, providing the title compound as a yellow oil (9.9 mg, 40% yield, 1:1 dr). On the other hand, if prepared according to the revised procedure (Supplementary Table 12), **32** was obtained in 52% yield, 12.9 mg, 1:1 dr).

R_f = 0.33 (Hex/EtOAc = 2/0.5)

^{1}H NMR (600 MHz, $CDCl_3$) mixture of two diastereomers: δ 5.74 (s, 1H), 2.48 – 2.40 (m, 1H), 2.36 – 2.16 (m, 3H), 2.01 – 1.95 (m, 1H), 1.86 – 1.75 (m, 2H), 1.66 – 1.57 (m, 1H), 1.42 – 1.28 (m, 3H), 1.28 – 1.07 (m, 4H), 1.07 (s, 1.5H), 1.07 (s, 1.5H), 0.96 (d, *J* = 6.8 Hz, 3H), 0.90 (t, *J* = 7.2 Hz, 3H), 0.84 – 0.84 (m, 3H).

^{13}C NMR (151 MHz, $CDCl_3$) mixture of two diastereomers: δ 199.8, 171.7, 124.4, 124.4, 43.0, 42.1, 42.1, 41.1, 40.6, 40.6, 39.4, 39.2, 37.5, 37.2, 37.1, 36.7, 36.2, 33.4, 33.3, 30.4, 28.6, 20.6, 17.0, 17.0, 16.1, 15.8, 15.0, 15.0, 14.4.

HRMS (APCI) m/z calcd. for $C_{17}H_{28}O$ $[M+H]^+$ **249.2213** found: **249.2206**.

(1S,2S,4aR,4bR,7S,9aS,10aR)-1-methyl-13-oxo-8-propyl-1,2,5,6,8,9,10,10a-octahydro-4a,1-(epoxymethano)-7,9a-methanobenzo[a]azulene-2,7(4bH)-diyl diacetate (33)

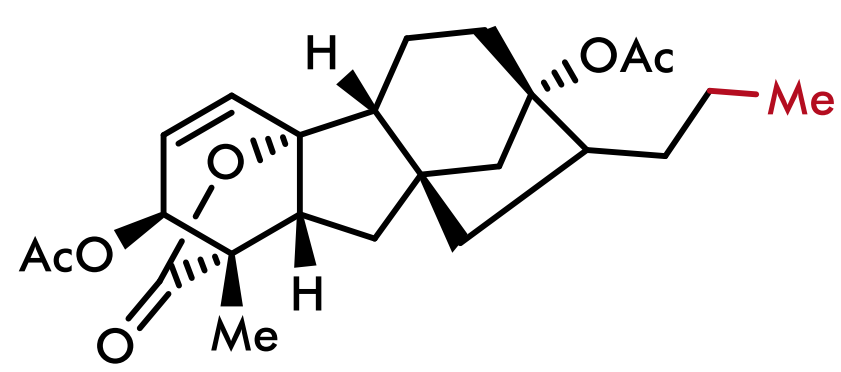

33

Prepared according to the general procedure A using $Fe(NO_3)_3 \cdot 9H_2O$ (6.0 mg, 0.015 mmol, 15 mol%), Na_2CO_3 (1.1 mg, 0.01 mmol, 10 mol%), bis(2,4,6-triisopropylphenyl)disulfide (11.4 mg, 0.025 mmol, 25 mol%), methylmalonic acid **MA-1** (47 mg, 0.4 mmol, 4.0 equiv.), and alkene **A29** (39 mg, 0.1 mmol, 1.0 equiv.) in $MeCN/H_2O/PhCF_3$ (0.5 mL/0.5 mL/0.05 mL). The crude was purified by flash column chromatography, providing the title compound as a pale-yellow frothy oil (14.2 mg, 34% yield, >95:5 dr).

R_f = 0.59 (Hex/EtOAc = 1/1)

^{1}H NMR (600 MHz, CDCl$_3$) δ 6.39 (d, *J* = 9.3 Hz, 1H), 5.83 (dd, *J* = 9.3, 3.8 Hz, 1H), 5.30 (d, *J* = 3.6 Hz, 1H), 2.79 (dd, *J* = 11.2, 8.3 Hz, 1H), 2.54 (dd, *J* = 11.0, 2.2 Hz, 1H), 2.09 (s, 3H), 2.21 – 1.84 (m, 6H), 1.97 (s, 3H), 1.73 (dd, *J* = 11.8, 6.0 Hz, 1H), 1.67 (dd, *J* = 13.8, 11.1 Hz, 1H), 1.62 – 1.52 (m, 2H), 1.49 (d, *J* = 11.0 Hz, 1H), 1.37 – 1.18 (m, 4H), 1.17 (s, 3H), 0.92 (t, *J* = 7.3 Hz, 3H).

^{13}C NMR (151 MHz, CDCl$_3$) δ 177.7, 170.2, 169.9, 134.9, 128.8, 91.8, 86.6, 70.6, 54.4, 52.4, 50.3, 48.8, 46.6, 46.2, 42.0, 37.1, 33.6, 26.6, 22.2, 21.7, 20.8, 16.6, 14.6, 14.4.

HRMS (APCI) m/z calcd. for $C_{24}H_{32}O_6$ [M+H]$^+$ **417.2272** found: **417.2272**.

3,6-Dimethylheptyl benzoate (34)

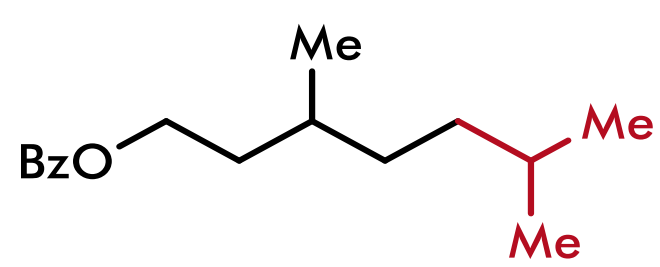

34

Prepared according to the general procedure A using $Fe(NO_3)_3 \cdot 9H_2O$ (6.0 mg, 0.015 mmol, 15 mol%), Na_2CO_3 (1.1 mg, 0.01 mmol, 10 mol%), bis(2,4,6-triisopropylphenyl)disulfide (11.4 mg, 0.025 mmol, 25 mol%), malonic acid **MA-2** (58.4 mg, 0.4 mmol, 4.0 equiv.), and alkene **A9** (19 mg, 0.1 mmol, 1.0 equiv.). The crude was purified by preparative thin layer chromatography, providing the title compound as a colorless oil (18.6 mg, 75% yield).

R_f = 0.66 (Hex/EtOAc = 2/0.5)

^{1}H NMR (600 MHz, CDCl$_3$) δ 8.04 (dd, *J* = 8.2, 1.5 Hz, 2H), 7.55 (t, *J* = 7.4 Hz, 1H), 7.44 (t, *J* = 7.8 Hz, 2H), 4.40 – 4.32 (m, 2H), 1.86 – 1.77 (m, 1H), 1.65 – 1.46 (m, 3H), 1.41 – 1.32 (m, 1H), 1.24 – 1.15 (m, 3H), 0.96 (d, *J* = 6.3 Hz, 3H), 0.88 (dd, *J* = 6.6, 2.6 Hz, 6H).

^{13}C NMR (151 MHz, CDCl$_3$) δ 166.7, 132.8, 130.6, 129.5, 128.3, 63.6, 36.2, 35.6, 34.7, 30.3, 28.3, 22.8, 22.6, 19.7.

HRMS (APCI) m/z calcd. for $C_{16}H_{24}O_2$ [M+H]$^+$ **249.1849** found: **249.1842**.

1-(4-(Benzoyloxy)-2-methylbutyl)cyclopropane-1-carboxylic acid (35)

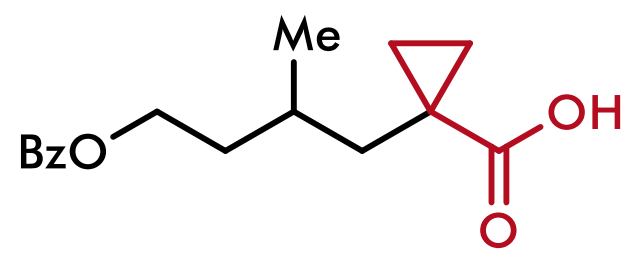

35

Prepared according to the general procedure A using $Fe(NO_3)_3 \cdot 9H_2O$ (6.0 mg, 0.015 mmol, 15 mol%), Na_2CO_3 (1.1 mg, 0.01 mmol, 10 mol%), 2,4,6-Triisopropylthiophenol (2.5 µL, 0.01 mmol, 10 mol%), malonic acid **MA-3** (52 mg, 0.4 mmol, 4.0 equiv.), and alkene **A9** (19 mg, 0.1 mmol, 1.0 equiv.). The crude was purified by preparative thin layer chromatography, providing the title compound as a colorless oil (21.6 mg, 78% yield).

R_f = 0.48 (Hex/EtOAc = 1/1)

^{1}H NMR (600 MHz, $CDCl_3$) δ 8.03 (d, J = 7.0 Hz, 2H), 7.54 (t, *J* = 7.4 Hz, 1H), 7.43 (t, *J* = 7.8 Hz, 2H), 4.42 – 4.31 (m, 2H), 2.18 – 2.07 (m, 1H), 1.90 – 1.78 (m, 2H), 1.62 – 1.53 (m, 1H), 1.40 – 1.24 (m, 3H), 1.00 (d, *J* = 6.7 Hz, 3H), 0.85 – 0.75 (m, 2H).

^{13}C NMR (151 MHz, $CDCl_3$) δ 182.2, 166.7, 132.8, 130.5, 129.6, 128.3, 63.3, 40.6, 35.8, 29.1, 21.7, 19.8, 17.0, 16.5.

HRMS (APCI) m/z calcd. for $C_{16}H_{20}O_4$ $[M+H]^+$ **277.1434** found: **277.1427**.

4-Cyclobutyl-3-methylbutyl benzoate (36)

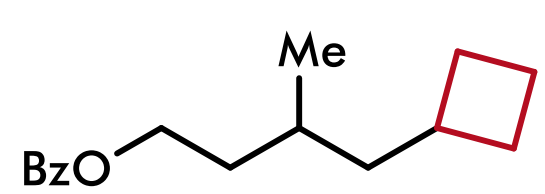

36

Prepared according to the general procedure A using $Fe(NO_3)_3 \cdot 9H_2O$ (6.0 mg, 0.015 mmol, 15 mol%), Na_2CO_3 (1.1 mg, 0.01 mmol, 10 mol%), bis(2,4,6-triisopropylphenyl)disulfide (11.4 mg, 0.025 mmol, 25 mol%), malonic acid **MA-4** (57.6 mg, 0.4 mmol, 4.0 equiv.), and alkene **A9** (19 mg, 0.1 mmol, 1.0 equiv.). The crude was purified by preparative thin layer chromatography, providing the title compound as a colorless oil (15.3 mg, 62% yield).

R_f = 0.57 (Hex/EtOAc = 2/0.5)

^{1}H NMR (600 MHz, $CDCl_3$) δ 8.04 (dd, *J* = 8.3, 1.4 Hz, 2H), 7.57 – 7.54 (m, 1H), 7.46 – 7.42 (m, 2H), 4.39 – 4.29 (m, 2H), 2.44 – 2.35 (m, 1H), 2.08 – 1.98 (m, 2H), 1.89 – 1.74 (m, 3H),

1.66 – 1.53 (m, 4H), 1.49 – 1.43 (m, 1H), 1.33 – 1.28 (m, 1H), 0.92 (d, *J* = 6.5 Hz, 3H).

^{13}C NMR (151 MHz, $CDCl_3$) δ 166.7, 132.8, 130.6, 129.6, 129.5, 128.3, 128.3, 63.5, 44.5, 35.7, 34.1, 29.2, 28.8, 28.5, 19.8, 18.7.

HRMS (APCI) m/z calcd. for $C_{16}H_{22}O_2$ $[M+H]^+$ **247.1693** found: **247.1686**.

4-Cyclohexyl-3-methylbutyl benzoate (37)

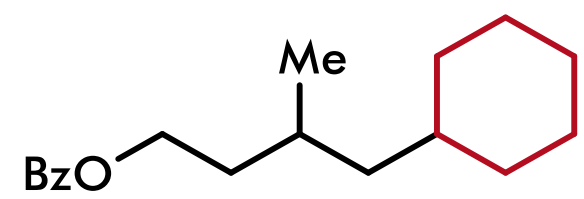

37

Prepared according to the general procedure A using $Fe(NO_3)_3 \cdot 9H_2O$ (6.0 mg, 0.015 mmol, 15 mol%), Na_2CO_3 (1.1 mg, 0.01 mmol, 10 mol%), bis(2,4,6-triisopropylphenyl)disulfide (11.4 mg, 0.025 mmol, 25 mol%), malonic acid **MA-5** (68.8 mg, 0.4 mmol, 4.0 equiv.), and alkene **A9** (19 mg, 0.1 mmol, 1.0 equiv.). The crude was purified by preparative thin layer chromatography, providing the title compound as a colorless oil (7.1 mg, 26% yield).

R_f = 0.63 (Hex/EtOAc = 2/0.5)

^{1}H NMR (600 MHz, $CDCl_3$) δ 8.04 (dd, *J* = 8.3, 1.4 Hz, 2H), 7.57 – 7.54 (m, 1H), 7.44 (t, *J* = 7.8 Hz, 2H), 4.41 – 4.30 (m, 2H), δ 1.81 – 1.73 (m, 2H), 1.73 – 1.62 (m, 5H), 1.59 – 1.52 (m, 1H), 1.38 – 1.31 (m, 1H), 1.28 – 1.12 (m, 5H), 1.09 – 1.04 (m, 1H), 0.94 (d, *J* = 6.5 Hz, 3H), 0.92 – 0.78 (m, 2H).

^{13}C NMR (151 MHz, $CDCl_3$) δ 166.8, 132.9, 130.6, 129.7, 128.5, 63.7, 45.2, 36.0, 34.9, 34.2, 33.3, 26.9, 26.9, 26.6, 26.5, 20.0.

HRMS (APCI) m/z calcd. for $C_{18}H_{26}O_2$ $[M+H]^+$ **275.2006** found: **275.1999**.

3-Methyl-6-phenylhexyl benzoate (38)

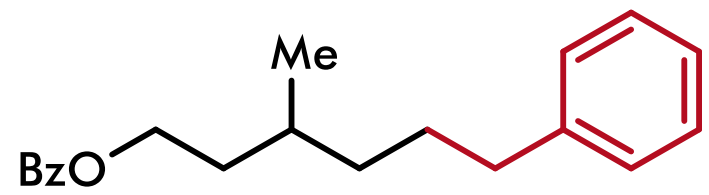

38

Prepared according to the general procedure A using $Fe(NO_3)_3 \cdot 9H_2O$ (6.0 mg, 0.015 mmol, 15 mol%), Na_2CO_3 (1.1 mg, 0.01 mmol, 10 mol%), 2,4,6-Triisopropylthiophenol (2.5 µL, 0.01 mmol, 10 mol%), malonic acid **MA-6** (77.6 mg, 0.4 mmol, 4.0 equiv.), and alkene **A9** (19 mg, 0.1 mmol, 1.0 equiv.). The crude was purified by preparative thin layer chromatography, providing the title compound as a colorless oil (18 mg, 61% yield).

$\mathbf{R_f}$ = 0.54 (Hex/EtOAc = 2/0.5)

^{1}H NMR (600 MHz, $CDCl_3$) δ 8.05 (d, *J* = 7.0 Hz, 2H), 7.58 – 7.54 (m, 1H), 7.45 (t, *J* = 7.8 Hz, 2H), 7.30 – 7.26 (m, 2H), 7.20 – 7.17 (m, 3H), 4.41 – 4.32 (m, 2H), 2.67 – 2.57 (m, 2H), 1.85 – 1.80 (m, 1H), 1.73 – 1.55 (m, 4H), 1.47 – 1.41 (m, 1H), 1.31 – 1.26 (m, 1H), 0.98 (d, *J* = 6.6 Hz, 3H).

^{13}C NMR (151 MHz, $CDCl_3$) δ 166.7, 142.7, 132.8, 132.8, 130.5, 129.6, 129.6, 129.5, 128.4, 128.4, 128.3, 128.3, 128.3, 125.7, 63.5, 36.6, 36.2, 35.6, 29.9, 28.9, 19.6.

HRMS (APCI) m/z calcd. for $C_{20}H_{24}O_2$ $[M+H]^+$ **297.1849** found: **297.1842**.

7-Methoxy-3-methylheptyl benzoate (39)

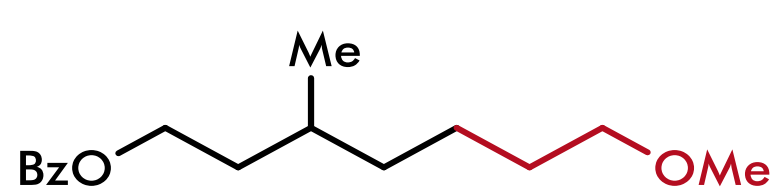

39

Prepared according to the general procedure A using $Fe(NO_3)_3 \cdot 9H_2O$ (6.0 mg, 0.015 mmol, 15 mol%), Na_2CO_3 (1.1 mg, 0.01 mmol, 10 mol%), bis(2,4,6-triisopropylphenyl)disulfide (11.4 mg, 0.025 mmol, 25 mol%), malonic acid **MA-7** (64.8 mg, 0.4 mmol, 4.0 equiv.), and alkene **A9** (19 mg, 0.1 mmol, 1.0 equiv.). The crude was purified by preparative thin layer chromatography, providing the title compound as a colorless oil (15.3 mg, 58% yield).

$\mathbf{R_f}$ = 0.4 (Hex/EtOAc = 2/0.5)

^{1}H NMR (600 MHz, $CDCl_3$) δ 8.03 (d, *J* = 6.9 Hz, 2H), 7.56 – 7.53 (m, 1H), 7.43 (t, *J* = 7.8 Hz,

2H), 4.40 – 4.31 (m, 2H), 3.37 (t, *J* = 6.5 Hz, 2H), 3.32 (s, 3H), 1.84 – 1.78 (m, 1H), 1.69 – 1.62 (m, 1H), 1.61 – 1.53 (m, 3H), 1.44 – 1.31 (m, 3H), 1.26 – 1.20 (m, 1H), 0.96 (d, *J* = 6.6 Hz, 3H).

^{13}C NMR (151 MHz, $CDCl_3$) δ 166.7, 132.8, 130.5, 129.6, 129.5, 128.3, 128.3, 72.9, 63.5, 58.6, 36.8, 35.5, 30.0, 29.9, 23.5, 19.5.

HRMS (APCI) m/z calcd. for $C_{16}H_{24}O_3$ $[M+H]^+$ **265.1798** found: **265.1792**.

3-Methyl-7-oxooctyl benzoate (40)

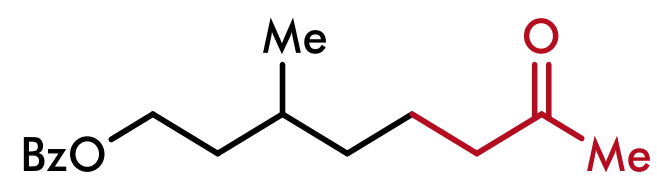

40

Prepared according to the general procedure A using $Fe(NO_3)_3 \cdot 9H_2O$ (6.0 mg, 0.015 mmol, 15 mol%), Na_2CO_3 (1.1 mg, 0.01 mmol, 10 mol%), bis(2,4,6-triisopropylphenyl)disulfide (11.4 mg, 0.025 mmol, 25 mol%), malonic acid **MA-8** (64 mg, 0.4 mmol, 4.0 equiv.), and alkene **A9** (19 mg, 0.1 mmol, 1.0 equiv.) in MeCN/H_2O/DCE (0.5 mL/0.5 mL/0.05 mL). The crude was purified by preparative thin layer chromatography, providing the title compound as a yellow oil (8.4 mg, 32% yield).

R_f = 0.29 (Hex/EtOAc = 2/0.5)

^{1}H NMR (600 MHz, $CDCl_3$) δ 8.05 – 8.02 (m, 2H), 7.55 (tt, *J* = 7.7, 1.2 Hz, 1H), 7.44 (tt, *J* = 7.3, 0.9 Hz, 2H), 4.40 – 4.31 (m, 2H), 2.42 (td, *J* = 7.4, 1.7 Hz, 2H), 2.13 (s, 3H), 1.85 – 1.77 (m, 1H), 1.69 – 1.54 (m, 4H), 1.39 – 1.33 (m, 1H), 1.23 – 1.17 (m, 1H), 0.97 (d, *J* = 6.6 Hz, 3H).

^{13}C NMR (151 MHz, $CDCl_3$) δ 209.0, 166.7, 132.9, 130.5, 129.5, 128.4, 63.4, 43.9, 36.3, 35.4, 29.9, 29.9, 21.2, 19.4.

HRMS (APCI) m/z calcd. for $C_{16}H_{22}O_3$ $[M+H]^+$ **263.1642** found: **263.1635**.

7-Cyano-3-methylheptyl benzoate (41)

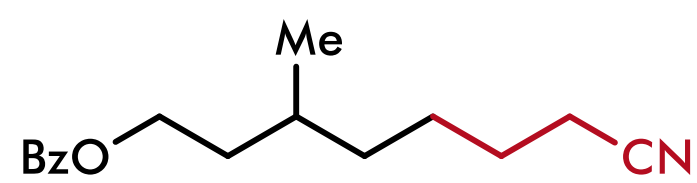

41

Prepared according to the general procedure A using $Fe(NO_3)_3 \cdot 9H_2O$ (6.0 mg, 0.015 mmol, 15 mol%), Na_2CO_3 (1.1 mg, 0.01 mmol, 10 mol%), bis(2,4,6-triisopropylphenyl)disulfide (11.4 mg, 0.025 mmol, 25 mol%), malonic acid **MA-9** (62.8 mg, 0.4 mmol, 4.0 equiv.), and alkene **A9** (19 mg, 0.1 mmol, 1.0 equiv.) in $MeCN/H_2O$ (0.25 mL/0.25 mL). The crude was purified by preparative thin layer chromatography, providing the title compound as a colorless oil (10.9 mg, 42% yield).

R_f = 0.23 (Hex/EtOAc = 2/0.5)

^{1}H NMR (600 MHz, $CDCl_3$) δ 8.03 (dd, *J* = 8.4, 1.4 Hz, 2H), 7.58 – 7.53 (m, 1H), 7.46 – 7.43 (m, 2H), 4.41 – 4.31 (m, 2H), 2.35 (t, *J* = 7.1 Hz, 2H), 1.84 – 1.79 (m, 1H), 1.70 – 1.38 (m, 7H), 1.31 – 1.19 (m, 1H), 0.98 (d, *J* = 6.6 Hz, 3H).

^{13}C NMR (151 MHz, $CDCl_3$) δ 166.7, 132.9, 130.4, 129.6, 129.5, 128.4, 128.4, 128.4, 119.7, 63.3, 36.0, 35.5, 29.8, 26.1, 25.6, 19.4, 17.2.

HRMS (APCI) m/z calcd. for $C_{16}H_{21}NO_2$ $[M+H]^+$ **297.1849** found: **297.1842**.

2-(1-Methyl-2-oxocyclohexyl)ethyl benzoate (41')

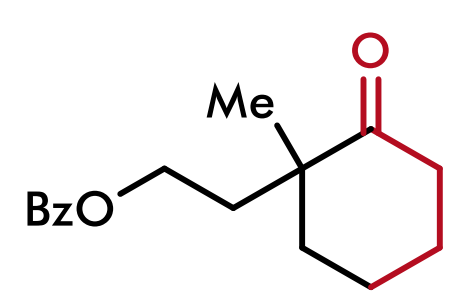

41'

Byproduct of previous reaction. 37' was isolated from the crude in the previous reaction by preparative thin layer chromatography, providing the title compound as a colorless oil (3.1 mg, 12% yield).

R_f = 0.29 (Hex/EtOAc = 2/0.5)

^{1}H NMR (600 MHz, $CDCl_3$) δ 8.00 (d, *J* = 7.0 Hz, 2H), 7.57 – 7.53 (m, 1H), 7.43 (t, *J* = 7.8 Hz, 2H), 4.45 – 4.27 (m, 2H), 2.53 – 2.38 (m, 2H), 2.23 (dt, *J* = 14.4, 7.3 Hz, 1H), 1.95 – 1.67 (m,

7H), 1.20 (s, 3H).

^{13}C NMR (151 MHz, $CDCl_3$) δ 214.9, 166.5, 132.9, 130.2, 129.6, 128.4, 61.5, 47.5, 39.9, 38.7, 36.4, 27.6, 22.7, 21.0.

HRMS (APCI) m/z calcd. for $C_{16}H_{20}O_3$ $[M+H]^+$ **261.1485** found: **261.1478**.

5-Chloro-3-methylpentyl benzoate (42)

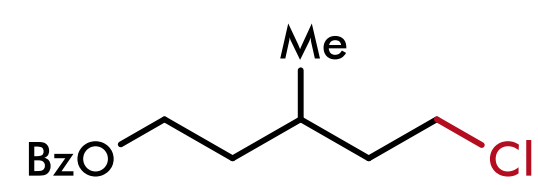

42

Prepared according to the general procedure A using $Fe(NO_3)_3 \cdot 9H_2O$ (6.0 mg, 0.015 mmol, 15 mol%), Na_2CO_3 (1.1 mg, 0.01 mmol, 10 mol%), bis(2,4,6-triisopropylphenyl)disulfide (11.4 mg, 0.025 mmol, 25 mol%), malonic acid **MA-10** (55.2 mg, 0.4 mmol, 4.0 equiv.), and alkene **A9** (19 mg, 0.1 mmol, 1.0 equiv.). The crude was purified by preparative thin layer chromatography, providing the title compound as a colorless oil (15.6 mg, 65% yield).

R_f = 0.51 (Hex/EtOAc = 2/0.5)

^{1}H NMR (600 MHz, $CDCl_3$) δ 8.04 (dd, *J* = 8.1, 1.5 Hz, 2H), 7.58 – 7.54 (m, 1H), 7.44 (t, *J* = 7.8 Hz, 2H), 4.42 – 4.34 (m, 2H), 3.67 – 3.54 (m, 2H), 1.98 – 1.80 (m, 3H), 1.73 – 1.60 (m, 2H), 1.01 (d, *J* = 6.6 Hz, 3H).

^{13}C NMR (151 MHz, $CDCl_3$) δ 166.8, 133.1, 130.5, 129.7, 128.5, 128.5, 63.1, 43.0, 39.6, 35.3, 27.7, 19.1.

HRMS (APCI) m/z calcd. for $C_{13}H_{17}ClO_2$ $[M+H]^+$ **241.0990** found: **241.0983**.

5-Fluoro-3-methylpentyl benzoate (43)

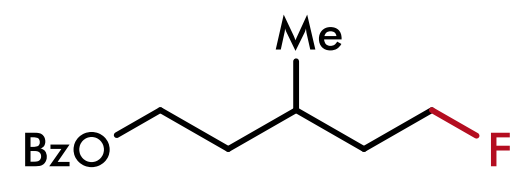

43

Prepared according to the general procedure B using $Fe(NO_3)_3 \cdot 9H_2O$ (4.0 mg, 0.01 mmol, 10 mol%), Na_2CO_3 (1.1 mg, 0.01 mmol, 10 mol%), bis(2,4,6-triisopropylphenyl)disulfide (9.4 mg, 0.02 mmol, 20 mol%), malonic acid **MA-11** (36.6 mg, 0.3 mmol, 3.0 equiv.), and alkene **A9** (19 mg, 0.1 mmol, 1.0 equiv.) in $MeCN/H_2O$ (0.9 mL/0.1 mL). The crude was purified by preparative thin layer chromatography, providing the title compound as a colorless oil (16.1 mg, 72% yield).

$\mathbf{R_f}$ = 0.43 (Hex/EtOAc = 2/0.5)

^{1}H NMR (600 MHz, $CDCl_3$) δ 8.04 (dd, *J* = 8.3, 1.4 Hz, 2H), 7.57 – 7.54 (m, 1H), 7.44 (t, *J* = 7.8 Hz, 2H), 4.61 – 4.54 (m, 1H), 4.52 – 4.46 (m, 1H), 4.41 – 4.35 (m, 2H), 1.95 – 1.77 (m, 3H), 1.70 – 1.53 (m, 2H), 1.03 (d, *J* = 6.5 Hz, 3H).

^{13}C NMR (151 MHz, $CDCl_3$) δ 166.6, 132.9, 130.4, 129.6, 129.5, 129.5, 128.4, 128.3, 82.9, 81.8, 63.1, 37.3, 37.1, 35.5, 26.7, 26.7, 19.3.

^{19}F NMR (564 MHz, $CDCl_3$) δ –218.60 (tt, *J* = 49.3, 26.1 Hz).

HRMS (APCI) m/z calcd. for $C_{16}H_{21}NO_2$ $[M+H]^+$ **225.1285** found: **225.1279**.

Mixture of 4-(fluoromethyl)hexyl benzoate and 3-(fluoromethyl)hexyl benzoate (44)

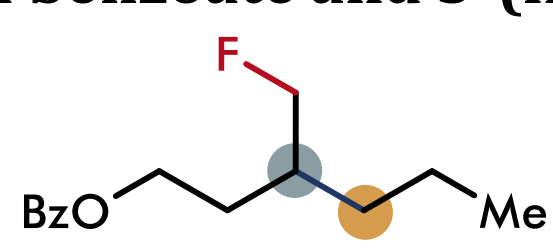

44

Prepared according to the synthetic procedure for the hydrofluoromethylation of **A35** using $Fe(NO_3)_3 \cdot 9H_2O$ (4.0 mg, 0.01 mmol, 10 mol%), Na_2CO_3 (1.1 mg, 0.01 mmol, 10 mol%), bis(2,4,6-triisopropylphenyl)disulfide (4.7 mg, 0.01 mmol, 10 mol%), malonic acid **MA-11** (73.2 mg, 0.6 mmol, 6.0 equiv.) in total, and alkene **A35** (20 mg, 0.1 mmol, 1.0 equiv.). The crude was purified by preparative thin layer chromatography, providing the title compound as a colorless oil (10 mg, 42% yield, 1:1 rr).

R_f = 0.56 (Hex/EtOAc = 2/0.1)

^{1}H NMR (600 MHz, $CDCl_3$) mixture of two regioisomers: δ 8.13 – 7.91 (m, 2H), 7.67 – 7.52 (m, 1H), 7.49 – 7.40 (m, 2H), 4.64 – 4.24 (m, 4H), 1.98 – 1.78 (m, 2H), 1.74 – 1.48 (m, 2H), 1.46 – 1.34 (m, 3H), 1.02 – 0.82 (m, 3H).

^{13}C NMR (151 MHz, $CDCl_3$) mixture of two regioisomers: δ 166.8, 166.7, 133.1, 133.0, 130.5, 130.4, 129.7, 128.5, 128.5, 86.8, 86.6, 85.7, 85.5, 65.3, 63.2, 40.3, 40.2, 36.3, 36.2, 32.7, 32.6, 30.0, 29.9, 29.8, 26.8, 26.7, 26.2, 23.2, 23.1, 20.1, 14.4, 11.3.

^{19}F NMR (564 MHz, $CDCl_3$) mixture of two regioisomers: δ –225.12 (dt, *J* = 47.9, 23.7 Hz), -225.72 (dt, *J* = 48.3, 24.2 Hz).

HRMS (ESI) m/z calcd. for $C_{14}H_{19}FO_2$ $[M+H]^+$ **239.1442** found: **239.1439**.

2-(Fluoromethyl)-3-methylbutyl benzoate (45)

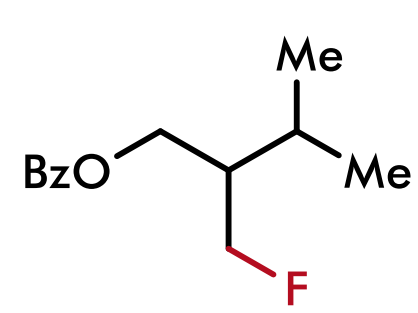

45

Prepared according to the synthetic procedure for the hydrofluoromethylation of **A36** using $Fe(NO_3)_3 \cdot 9H_2O$ (4.0 mg, 0.01 mmol, 10 mol%), Na_2CO_3 (1.1 mg, 0.01 mmol, 10 mol%), bis(2,4,6-triisopropylphenyl)disulfide (4.7 mg, 0.01 mmol, 10 mol%), malonic acid **MA-11** (73.2 mg, 0.6 mmol, 6.0 equiv.) in total, and alkene **A36** (19 mg, 0.1 mmol, 1.0 equiv.). The crude was purified by preparative thin layer chromatography, providing the title compound as a colorless oil (16.8 mg, 75% yield).

R_f = 0.58 (Hex/EtOAc = 2/0.1)

^{1}H NMR (600 MHz, $CDCl_3$) δ 8.24 – 7.82 (m, 2H), 7.64 – 7.51 (m, 1H), 7.45 (t, *J* = 7.9 Hz, 2H), 4.70 – 4.62 (m, 1H), 4.61 – 4.53 (m, 1H), 4.49 (ddd, *J* = 11.2, 4.6, 1.3 Hz, 1H), 4.36 (dd, *J* = 11.2, 6.8 Hz, 1H), 2.06 – 1.88 (m, 2H), 1.04 (d, *J* = 6.5 Hz, 6H).

^{13}C NMR (151 MHz, CDCl$_3$) δ 166.5, 133.0, 130.2, 129.6, 128.4, 83.2, 82.1, 62.9, 62.9, 44.9, 44.8, 26.5, 26.5, 20.2, 20.2.

^{19}F NMR (564 MHz, CDCl$_3$) δ –227.90 (td, *J* = 46.9, 25.1 Hz).
HRMS (ESI) m/z calcd. for $C_{13}H_{17}FO_2$ $[M+H]^+$ **225.1285** found: **225.1283**.

3-Methylpentyl benzoate (46)

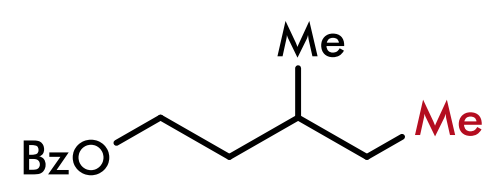

46

Prepared according to the hydromethylation procedure aforementioned for alkene **A9**. The crude was purified by preparative thin layer chromatography, providing the title compound as a pale-yellow oil (13.6 mg, 66% yield).
R$_f$ = 0.6 (Hex/EtOAc = 2/0.5)
^{1}H NMR (600 MHz, CDCl$_3$) δ 8.06 – 8.03 (m, 2H), 7.57 – 7.54 (m, 1H), 7.45 – 7.42 (m, 2H), 4.41 – 4.32 (m, 2H), 1.86 – 1.77 (m, 1H), 1.63 – 1.55 (m, 2H), 1.46 – 1.38 (m, 1H), 1.28 – 1.21 (m, 1H), 0.96 (d, *J* = 6.4 Hz, 3H), 0.91 (t, *J* = 7.4 Hz, 3H).
^{13}C NMR (151 MHz, CDCl$_3$) δ 166.4, 132.5, 130.3, 129.2, 128.0, 63.3, 34.9, 31.3, 29.1, 18.8, 11.0.
HRMS (APCI) m/z calcd. for $C_{13}H_{18}O_2$ $[M+H]^+$ **207.1380** found: **207.1375**.

Mixture of 4-methylhexyl benzoate and 3-methylhexyl benzoate (47)

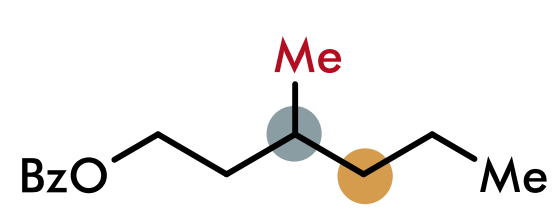

47

Prepared according to the synthetic procedure for the hydromethylation of **A35**. The crude was purified by preparative thin layer chromatography, providing the title compound as a colorless oil (16% NMR yield, 1:1 rr).
R$_f$ = 0.5 (Hex/EtOAc = 2/0.1)

^{1}H NMR (600 MHz, $CDCl_3$) mixture of two regioisomers: δ 8.04 (ddd, *J* = 8.4, 3.6, 1.4 Hz, 2H), 7.55 (td, *J* = 7.4, 1.4 Hz, 1H), 7.44 (td, *J* = 7.9, 1.8 Hz, 2H), 4.74 – 4.00 (m, 2H), 1.81 (dtd, *J* = 14.1, 7.1, 5.3 Hz, 1H), 1.70 – 1.62 (m, 1H), 1.61 – 1.53 (m, 2H), 1.42 – 1.28 (m, 3H), 1.01 – 0.85 (m, 6H).
^{13}C NMR (151 MHz, $CDCl_3$) mixture of two regioisomers: δ 166.7, 132.8, 130.6, 129.5, 128.3, 65.5, 63.6, 39.2, 35.6, 34.1, 32.8, 29.7, 29.4, 26.3, 20.0, 19.6, 19.1, 14.3, 11.4.
HRMS (APCI) m/z calcd. for $C_{14}H_{20}O_2$ $[M+H]^+$ 221.1536 found: 221.1531

2,3-Dimethylbutyl benzoate (48)

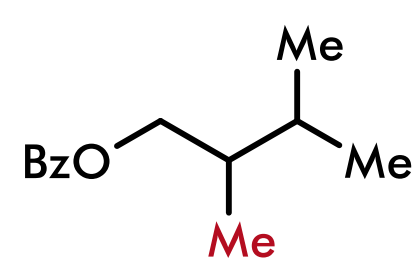

48

Prepared according to the synthetic procedure for the hydromethylation of **A36.** The crude was purified by preparative thin layer chromatography, providing the title compound as a colorless oil (7.6 mg, 37% yield).
R_f = 0.75 (Hex/EtOAc = 2/0.1)
^{1}H NMR (600 MHz, $CDCl_3$) δ 8.12 – 7.97 (m, 2H), 7.62 – 7.51 (m, 1H), 7.51 – 7.40 (m, 2H), 4.50 – 3.77 (m, 2H), 2.16 – 1.68 (m, 2H), 1.11 – 0.84 (m, 9H).
^{13}C NMR (151 MHz, $CDCl_3$) δ 166.7, 132.8, 130.6, 129.5, 128.3, 68.4, 38.3, 29.7, 29.6, 20.4, 18.4, 13.2.
HRMS (ESI) m/z calcd. for $C_{13}H_{18}O_2$ $[M]^+$ **206.1307** found: **206.1375**.

2,5,6-Trimethylheptyl benzoate (49)

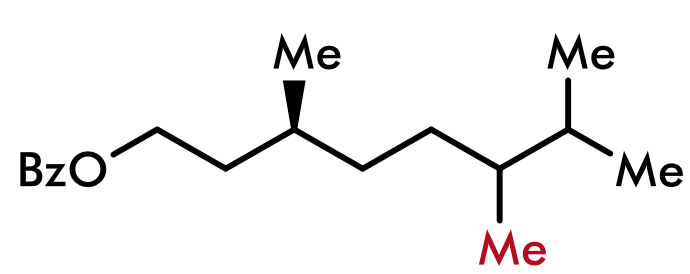

49

Prepared according to the synthetic procedure for the hydromethylation of **A37.** The crude was purified by preparative thin layer chromatography, providing the title compound as a

colorless oil (7.6 mg, 29% yield).

R$_f$ = 0.81 (Hex/EtOAc = 2/0.1)

^{1}H NMR (600 MHz, CDCl$_3$) mixture of two diastereomers: δ 8.23 – 7.82 (m, 2H), 7.63 – 7.53 (m, 1H), 7.44 (t, *J* = 7.8 Hz, 2H), 4.54 – 4.19 (m, 2H), 1.90 – 1.72 (m, 1H), 1.68 – 1.47 (m, 3H), 1.46 – 1.02 (m, 6H), 0.96 (dd, *J* = 6.5, 5.0 Hz, 3H), 0.85 (d, *J* = 6.8 Hz, 3H), 0.84 – 0.76 (m, 5H).

^{13}C NMR (151 MHz, CDCl$_3$) mixture of two diastereomers: δ 166.7, 132.8, 130.6, 129.5, 128.3, 63.6, 38.8, 38.8, 35.8, 35.5, 34.9, 34.8, 32.1, 31.8, 31.4, 31.3, 30.4, 30.3, 20.4, 20.3, 19.8, 19.6, 18.1, 17.8, 15.4, 15.4.

HRMS (ESI) m/z calcd. for $C_{17}H_{26}O_2$ $[M+H]^+$ **277.2162** found: **277.2160**.

2-Fluoro-6-phenylhexanoic acid (50)

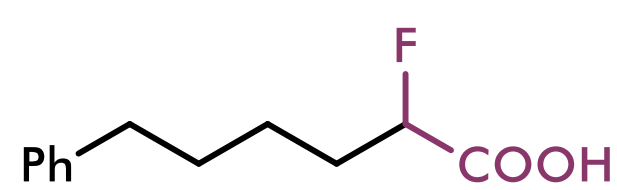

50

Prepared according to the general procedure C. The crude was purified by preparative thin layer chromatography, providing the title compound as a colorless oil (14.9 mg, 71% yield).

R$_f$ = 0.6 (Hex/EtOAc/AcOH = 3/2/0.1)

^{1}H NMR (600 MHz, CDCl$_3$) δ 8.36 – 7.74 (m, 1H), 7.28 – 7.16 (m, 2H), 7.15 – 7.03 (m, 3H), 4.88 (ddd, *J* = 49.1, 7.3, 4.5 Hz, 1H), 2.63 – 2.49 (m, 2H), 2.00 – 1.80 (m, 2H), 1.68 – 1.54 (m, 2H), 1.52 – 1.41 (m, 2H).

^{13}C NMR (151 MHz, CDCl$_3$) δ 175.5 (d, *J* = 24.3 Hz), 142.0, 128.4, 125.86, 88.3 (d, *J* = 184.9 Hz), 35.6, 32.1 (d, *J* = 20.9 Hz), 30.9, 24.1 (d, *J* = 2.8 Hz).

^{19}F NMR (564 MHz, CDCl$_3$) δ –192.17 (dt, *J* = 50.0, 25.5 Hz).

HRMS (ESI) m/z calcd. for $C_{12}H_{15}FO_2$ $[M-H]^-$ **209.0972** found: **209.0977**.

7-(Benzoyloxy)-2-fluoroheptanoic acid (51)

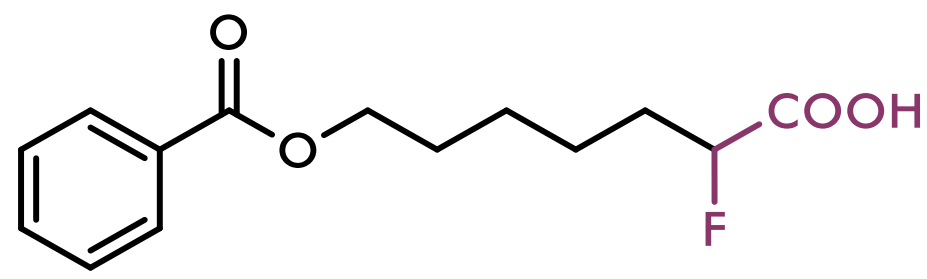

51

Prepared according to the general procedure C. The crude was purified by preparative thin layer chromatography, providing the title compound as a colorless oil (18.0 mg, 67% yield).

^{1}H NMR (600 MHz, $CDCl_3$) δ 8.04 (d, *J* = 7.6 Hz, 2H), 7.56 (t, *J* = 7.4 Hz, 1H), 7.44 (t, *J* = 7.6 Hz, 2H), 4.98 (dt, *J* = 49.1, 5.5 Hz, 1H), 4.33 (t, *J* = 6.6 Hz, 2H), 2.04 – 1.92 (m, 2H), 1.80 (p, *J* = 7.1 Hz, 2H), 1.64 – 1.47 (m, 4H).

^{13}C NMR (151 MHz, $CDCl_3$) δ 175.0, 174.9, 166.8, 133.0, 130.3, 129.6, 128.5, 128.4, 88.9, 87.7, 64.8, 32.2, 32.0, 28.5, 25.6, 24.1, 24.1.

^{19}F NMR (564 MHz, $CDCl_3$) δ –192.19 (dt, *J* = 49.7, 25.3 Hz).

HRMS (APCI) m/z calcd. for $C_{14}H_{17}FO_4$ $[M+H]^+$ **267.1027** found: **267.1033**.

2-Fluoro-7-(tosyloxy)heptanoic acid (52)

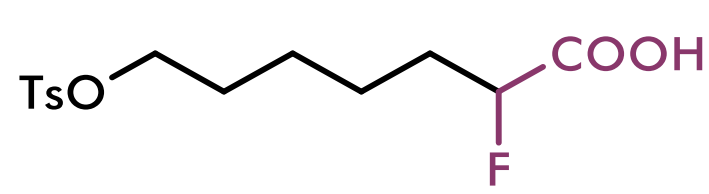

52

Prepared according to the general procedure C. The crude was purified by preparative thin layer chromatography, providing the title compound as a colorless oil (20 mg, 63% yield).

R_f = 0.29 (Hex/EtOAc/AcOH = 3/2/0.1)

^{1}H NMR (600 MHz, $CDCl_3$) δ 7.78 (d, *J* = 8.3 Hz, 2H), 7.67 – 7.40 (m, 1H), 7.34 (d, *J* = 8.0 Hz, 2H), 4.92 (ddd, *J* = 48.9, 7.3, 4.3 Hz, 1H), 4.02 (t, *J* = 6.3 Hz, 2H), 2.44 (s, 3H), 1.97 – 1.83 (m, 2H), 1.73 – 1.56 (m, 2H), 1.52 – 1.32 (m, 4H).

^{13}C NMR (151 MHz, $CDCl_3$) δ 174.8 (d, *J* = 24.2 Hz), 144.9, 133.0, 129.9, 127.9 (d, *J* = 184.8 Hz), 70.3, 31.9 (d, *J* = 20.9 Hz), 28.5, 24.9, 23.7 (d, *J* = 2.8 Hz), 21.6.

^{19}F NMR (564 MHz, $CDCl_3$) δ –191.90 to –192.53 (m).

HRMS (ESI) m/z calcd. for $C_{14}H_{19}FO_5S$ $[M-H]^-$ **317.0853** found: **317.0868**.

2-Fluoro-7-((4-(trifluoromethyl)benzoyl)oxy)heptanoic acid (53)

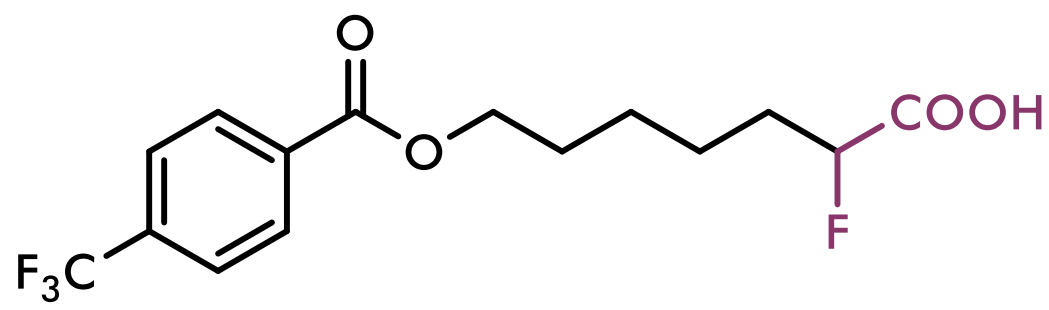

53

Prepared according to the general procedure C. The crude was purified by preparative thin layer chromatography, providing the title compound as a colorless oil (15.1 mg, 45% yield).
R_f = 0.44 (Hex/EtOAc/AcOH = 3/2/0.1)
^{1}H NMR (600 MHz, $CDCl_3$) δ 8.14 (d, *J* = 8.0 Hz, 2H), 7.71 (d, *J* = 8.2 Hz, 2H), 7.25 – 6.78 (m, 1H), 4.98 (ddd, *J* = 49.0, 7.4, 4.4 Hz, 1H), 4.36 (t, *J* = 6.6 Hz, 2H), 2.07 – 1.92 (m, 2H), 1.82 (p, *J* = 7.0 Hz, 2H), 1.64 – 1.49 (m, 4H).
^{13}C NMR (151 MHz, $CDCl_3$) δ 174.9 (d, *J* = 24.3 Hz), 165.5, 134.5 (q, *J* = 32.6 Hz), 133.5, 130.0, 125.4 (q, *J* = 3.8 Hz), 123.6 (q, *J* = 272.8 Hz), 88.3 (d, *J* = 185.0 Hz), 65.4, 32.0 (d, *J* = 20.9 Hz), 28.4, 25.6, 24.1 (d, *J* = 2.8 Hz).
^{19}F NMR (564 MHz, $CDCl_3$) δ –63.12 (s, 3F), –192.08 to –192.38 (m, 1F).
HRMS (ESI) m/z calcd. for $C_{15}H_{16}F_4O_4$ $[M–H]^-$ **335.0901** found: **335.0917**.

2-Fluoro-6-(phenylsulfonamido)hexanoic acid (54)

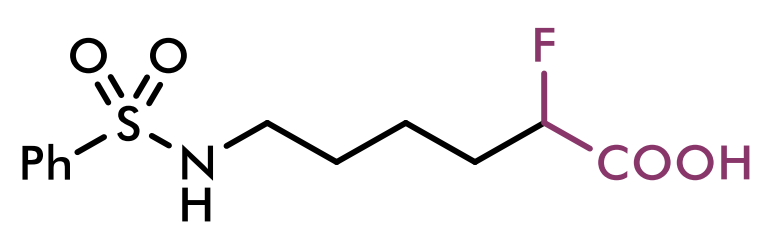

54

Prepared according to the general procedure C. The crude was purified by preparative thin layer chromatography, providing the title compound as a colorless oil (16.5 mg, 57% yield).
R_f = 0.12 (Hex/EtOAc/AcOH = 3/2/0.1)
^{1}H NMR (600 MHz, $CDCl_3$) δ 7.90 – 7.79 (m, 2H), 7.63 – 7.55 (m, 1H), 7.54 – 7.49 (m, 2H), 5.30 – 5.20 (m, 1H), 5.10 – 5.01 (m, 1H), 4.94 (ddd, *J* = 49.0, 6.8, 4.5 Hz, 1H), 3.01 – 2.93 (m, 2H), 1.96 – 1.83 (m, 2H), 1.61 – 1.38 (m, 4H).

^{13}C NMR (151 MHz, CDCl$_3$) δ 173.7 (d, *J* = 24.2 Hz), 139.7, 132.8, 129.2, 127.0, 88.2 (d, *J* = 184.4 Hz), 42.7, 31.5 (d, *J* = 20.9 Hz), 29.0, 21.2 (d, *J* = 2.9 Hz).

^{19}F NMR (564 MHz, CDCl$_3$) δ –191.89 (dt, *J* = 49.9, 25.1 Hz).

HRMS (ESI) m/z calcd. for $C_{12}H_{16}FNO_4S$ $[M–H]^-$ **288.0712** found: **288.0714**.

2-Fluoro-7-oxo-7-(2-(thiophen-2-yl)ethoxy)heptanoic acid (55)

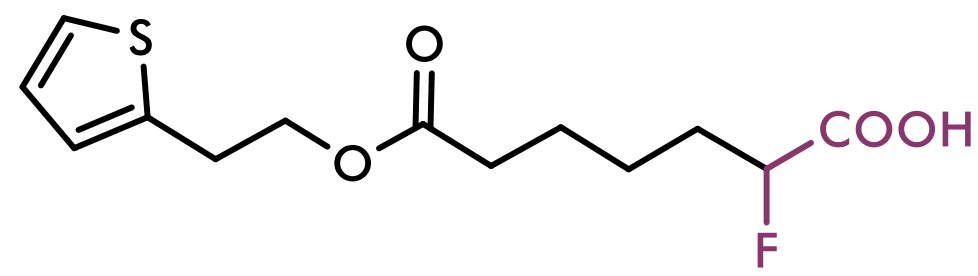

55

Prepared according to the general procedure C. The crude was purified by preparative thin layer chromatography, providing the title compound as a colorless oil (19 mg, 66% yield).

R$_f$ = 0.35 (Hex/EtOAc/AcOH = 3/2/0.1)

^{1}H NMR (600 MHz, CDCl$_3$) δ 7.16 (dd, *J* = 5.1, 1.1 Hz, 1H), 6.94 (dd, *J* = 5.1, 3.4 Hz, 1H), 6.85 (d, *J* = 3.4 Hz, 1H), 6.48 – 6.30 (m, 1H), 4.95 (ddd, *J* = 49.1, 7.5, 4.3 Hz, 1H), 4.31 (t, *J* = 6.7 Hz, 2H), 3.16 (t, *J* = 6.7 Hz, 2H), 2.36 (t, *J* = 7.4 Hz, 2H), 2.06 – 1.89 (m, 2H), 1.79 – 1.63 (m, *J* = 6.5 Hz, 2H), 1.58 – 1.47 (m, 2H).

^{13}C NMR (151 MHz, CDCl$_3$) δ 174.5 (d, *J* = 24.2 Hz), 173.4, 139.9, 126.9, 125.6, 124.1, 88.2 (d, *J* = 184.9 Hz), 64.7, 33.9, 31.9 (d, *J* = 20.9 Hz), 29.3, 24.3, 23.9 (d, *J* = 2.8 Hz).

^{19}F NMR (564 MHz, CDCl$_3$) δ –192.13 (dt, *J* = 50.1, 25.3 Hz).

HRMS (ESI) m/z calcd. for $C_{13}H_{17}FO_4S$ $[M–H]^-$ **287.0748** found: **287.0761**.

7-(2-(2,3-Dihydrobenzofuran-5-yl)acetoxy)-2-fluoroheptanoic acid (56)

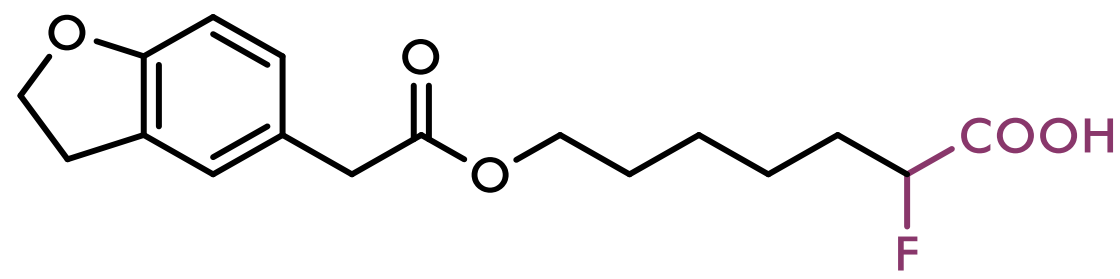

56

Prepared according to the general procedure C. The crude was purified by preparative thin layer chromatography, providing the title compound as a colorless oil (13.6 mg, 42% yield).

R_f = 0.24 (Hex/EtOAc/AcOH = 3/2/0.1)

^{1}H NMR (600 MHz, $CDCl_3$) δ 7.11 (d, *J* = 1.9 Hz, 1H), 6.99 (dd, *J* = 8.1, 1.9 Hz, 1H), 6.72 (d, *J* = 8.1 Hz, 1H), 4.94 (ddd, *J* = 49.1, 7.4, 4.4 Hz, 1H), 4.56 (t, *J* = 8.7 Hz, 2H), 4.52 – 4.24 (m, 1H), 4.09 (t, *J* = 6.6 Hz, 2H), 3.53 (s, 2H), 3.19 (t, *J* = 8.7 Hz, 2H), 2.01 – 1.84 (m, 2H), 1.70 – 1.59 (m, 2H), 1.56 – 1.44 (m, 2H), 1.42 – 1.31 (m, 2H).

^{13}C NMR (151 MHz, $CDCl_3$) δ 173.8 (d, *J* = 24.4 Hz), 172.3, 159.2, 128.9, 127.4, 126.0, 125.8, 109.2, 88.4 (d, *J* = 184.5 Hz), 71.3, 64.6, 40.8, 32.0 (d, *J* = 20.9 Hz), 29.7, 28.3, 25.4, 24.0 (d, *J* = 2.8 Hz).

^{19}F NMR (564 MHz, $CDCl_3$) δ –191.94 (dt, *J* = 53.7, 27.0 Hz).

HRMS (ESI) m/z calcd. for $C_{17}H_{21}FO_5$ $[M–H]^-$ **323.1289** found: **323.1303**.

7-(2-(2,3-Dihydrobenzofuran-5-yl)acetoxy)-2-fluoroheptanoic acid (57)

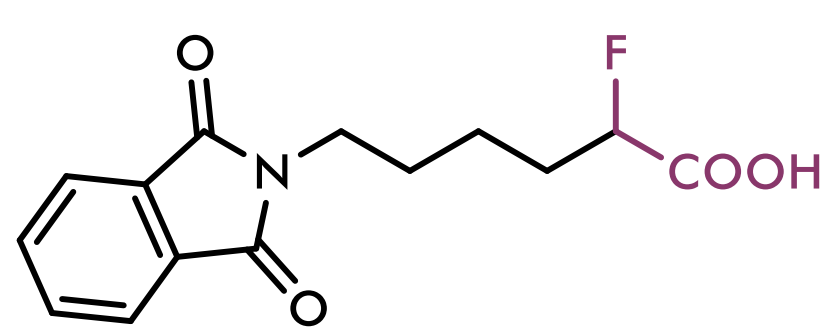

57

Prepared according to the general procedure C. The crude was purified by preparative thin layer chromatography, providing the title compound as a colorless oil (15.6 mg, 56% yield).

R_f = 0.21 (Hex/EtOAc/AcOH = 3/2/0.1)

^{1}H NMR (600 MHz, $CDCl_3$) δ 7.84 (dd, *J* = 5.4, 3.1 Hz, 1H), 7.71 (dd, *J* = 5.5, 3.1 Hz, 1H), 7.52 – 7.27 (m, 1H), 4.96 (ddd, *J* = 49.0, 7.7, 4.1 Hz, 1H), 3.71 (t, *J* = 7.2 Hz, 2H), 2.08 – 1.91 (m, 2H), 1.81-1.69 (m 2H), 1.57 (p, *J* = 7.8 Hz, 2H).

^{13}C NMR (151 MHz, CDCl$_3$) δ 174.5 (d, *J* = 24.2 Hz), 168.5, 134.0, 132.0, 123.3, 88.2 (d, *J* = 184.9 Hz), 37.5, 31.7 (d, *J* = 20.9 Hz), 28.0, 21.7 (d, *J* = 3.1 Hz).

^{19}F NMR (564 MHz, CDCl$_3$) δ –191.98 (dt, *J* = 49.5, 23.8 Hz).

HRMS (ESI) m/z calcd. for $C_{14}H_{14}FNO_4$ [M–H]$^-$ **278.0823** found: **278.0835**.

2-Fluoro-12-hydroxydodecanoic acid (58)

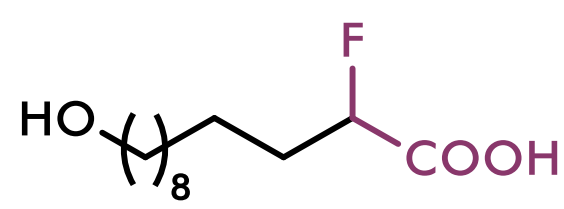

58

Prepared according to the general procedure C. The crude was purified by preparative thin layer chromatography, providing the title compound as a colorless oil (12.0 mg, 51% yield).

R$_f$ = 0.29 (Hex/EtOAc/AcOH = 3/2/0.1)

^{1}H NMR (600 MHz, Acetone-*d$_6$*) δ 4.99 (ddd, *J* = 49.3, 7.4, 4.3 Hz, 1H), 3.52 (t, *J* = 6.6 Hz, 2H), 1.97 – 1.81 (m, 2H), 1.55 – 1.43 (m, 4H), 1.42 – 1.30 (m, 12H).

^{13}C NMR (151 MHz, Acetone-*d$_6$*) δ 171.4, 171.3, 90.1, 88.9, 62.5, 33.8, 33.2, 33.0, 30.3, 30.2, 26.7, 25.2, 25.1.

^{19}F NMR (564 MHz, Acetone-*d$_6$*) δ –191.63 (m).

HRMS (ESI) m/z calcd. for $C_{12}H_{23}FO_3$ [M–H]$^-$ **233.1547** found: **233.1555**.

2-Fluoro-6-(phenylthio)hexanoic acid (59)

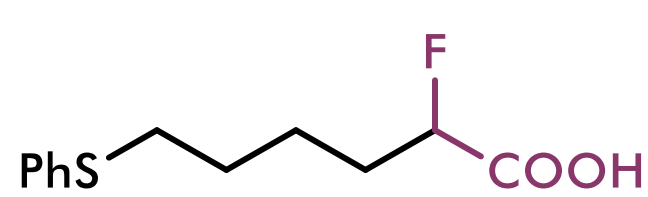

59

Prepared according to the general procedure C. The crude was purified by preparative thin layer chromatography, providing the title compound as a colorless oil (10.0 mg, 48% yield).

R$_f$ = 0.38 (Hex/EtOAc/AcOH = 3/2/0.1)

^{1}H NMR (600 MHz, CDCl$_3$) δ 7.36 – 7.31 (m, 2H), 7.31 – 7.27 (m, 2H), 7.21 – 7.14 (m, 1H), 7.09 – 6.55 (m, 1H), 4.95 (ddd, *J* = 49.0, 7.5, 4.4 Hz, 1H), 2.93 (t, *J* = 7.2 Hz, 2H), 2.04 – 1.87 (m, 2H), 1.76 – 1.56 (m, 4H).

^{13}C NMR (151 MHz, CDCl$_3$) δ 174.8 (d, *J* = 24.4 Hz), 136.4, 129.3, 128.9, 126.0, 88.2 (d, *J* = 185.0 Hz), 33.4, 31.7 (d, *J* = 20.9 Hz), 28.6, 23.6 (d, *J* = 2.9 Hz).
^{19}F NMR (564 MHz, CDCl$_3$) δ –192.23 (dt, *J* = 49.8, 25.3 Hz, 1F).
HRMS (ESI) m/z calcd. for $C_{12}H_{15}FO_2S$ [M–H]$^-$ **241.0693** found: **241.0703**.

12-Bromo-2-fluorododecanoic acid (60)

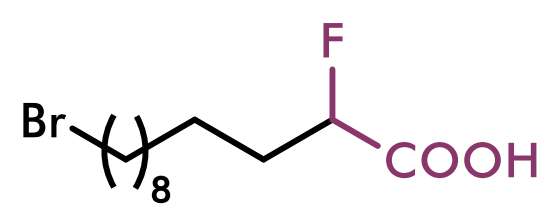

60

Prepared according to the general procedure C. The crude was purified by preparative thin layer chromatography, providing the title compound as a colorless oil (11.9 mg, 40% yield).
R$_f$ = 0.71 (Hex/EtOAc/AcOH = 3/2/0.1)
^{1}H NMR (600 MHz, CDCl$_3$) δ 6.09 (br, 1H), 4.96 (ddd, *J* = 49.2, 7.4, 4.5 Hz, 1H), 3.41 (t, *J* = 6.9 Hz, 2H), 1.99 – 1.88 (m, 2H), 1.85 (p, *J* = 6.9 Hz, 2H), 1.54 – 1.46 (m, 2H), 1.88 – 1.82 (p, *J* = 7.2 Hz, 2H), 1.38 – 1.28 (m, 10H).
^{13}C NMR (151 MHz, CDCl$_3$) δ 175.0 (d, *J* = 24.5 Hz), 88.5 (d, *J* = 184.6 Hz), 34.1, 32.8, 32.2 (d, *J* = 20.9 Hz), 29.4, 29.3, 29.3, 29.0, 28.7, 28.2, 24.3 (d, *J* = 2.7 Hz).
^{19}F NMR (564 MHz, CDCl$_3$) δ –191.88 (m).
HRMS (ESI) m/z calcd. for $C_{12}H_{22}BrFO_2$ [M–H]$^-$ **296.0737** found: **296.0743**.

2-Fluoro-4-methyl-5-phenylpentanoic acid (61)

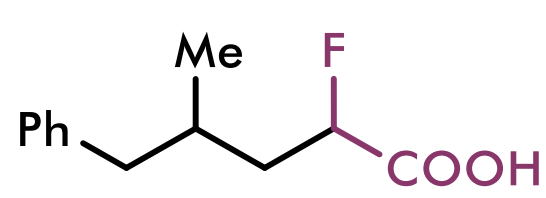

61

Prepared according to the general procedure C. The crude was purified by preparative thin layer chromatography, providing the title compound as a colorless oil (14.1 mg, 67% yield).

R_f = 0.59 (Hex/EtOAc/AcOH = 3/2/0.1)

^{1}H NMR (600 MHz, $CDCl_3$) mixture of four diastereomers: δ 7.56 (br, 1H), 7.30 – 7.28 (m, 2H), 7.22 – 7.20 (m, 1H), 7.17 – 7.14 (m, 2H), 5.03 (ddd, *J* = 19.3, 9.6, 3.4 Hz, 1H), 2.77 (dd, *J* = 13.5, 5.9 Hz, 0.49H), 2.64 (dd, *J* = 13.5, 6.9 Hz, 0.51H), 2.55 (dd, *J* = 13.6, 7.6 Hz, 0.51H), 2.45 (dd, *J* = 13.5, 8.3 Hz, 0.49H), 2.15 – 2.06 (m, 1H), 2.06 – 1.91 (m, 1H), 1.90 – 1.80 (m, 0.48H), 1.75 – 1.62 (m, 0.52H), 1.00 (d, *J* = 6.6 Hz, 1.51H), 0.97 (d, *J* = 6.7 Hz, 1.49H).

^{13}C NMR (151 MHz, $CDCl_3$) mixture of four diastereomers: δ 175.8 (d, *J* = 24.0 Hz), 175.7 (d, *J* = 24.2 Hz), 140.1, 140.0, 129.2, 129.2, 128.3, 126.1, 126.1, 87.5 (d, *J* = 185.0 Hz), 86.9 (d, *J* = 185.0 Hz), 43.7, 42.7, 38.8 (d, *J* = 20.3 Hz), 38.6 (d, *J* = 20.7 Hz), 31.6 (d, *J* = 2.3 Hz), 30.9 (d, *J* = 1.6 Hz), 19.9, 18.6.

^{19}F NMR (564 MHz, $CDCl_3$) mixture of four diastereomers: δ –189.75 to –190.52 (m), –191.82 to –192.50 (m).

HRMS (ESI) m/z calcd. for $C_{12}H_{15}FO_2$ $[M–H]^-$ **209.0972** found: **209.0978**.

6-(Benzoyloxy)-2-fluoro-4-methylhexanoic acid (62)

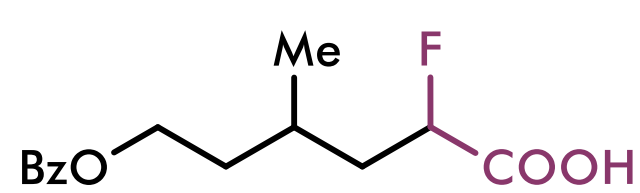

62

Prepared according to the general procedure C. The crude was purified by preparative thin layer chromatography, providing the title compound as a colorless oil (22 mg, 82% yield).

R_f = 0.53 (Hex/EtOAc/AcOH = 3/2/0.1)

^{1}H NMR (600 MHz, $CDCl_3$) mixture of four diastereomers: δ 8.41 – 8.13 (m, 1H), 8.06 – 7.97 (m, 2H), 7.59 – 7.50 (m, 1H), 7.48 – 7.39 (t, *J* = 7.6 Hz, 2H), 5.05 (ddt, *J* = 49.5, 9.9, 3.7 Hz, 1H), 4.45 – 4.33 (m, 2H), 2.11 – 1.79 (m, 4H), 1.79-1.62 (m, 1H), 1.09 (dd, *J* = 6.6, 1.8 Hz, 3H).

^{13}C NMR (151 MHz, $CDCl_3$) mixture of four diastereomers: δ 175.3 (d, *J* = 24.0 Hz), 175.3 (d, *J* = 24.2 Hz), 166.8, 166.8, 133.0, 130.2, 129.6, 128.4, 87.2 (d, *J* = 185.0 Hz), 86.9 (d, *J* = 185.5 Hz), 62.9, 62.8, 39.1 (d, *J* = 20.3 Hz), 39.0 (d, *J* = 20.4 Hz), 35.8, 34.6, 26.9(d, *J* = 1.9 Hz), 26.4 (d, *J* = 1.7 Hz), 19.8, 18.7.

^{19}F NMR (564 MHz, $CDCl_3$) mixture of four diastereomers: δ –190.01 to –190.52 (m), –

191.59 to –192.28 (m).

HRMS (ESI) m/z calcd. for $C_{14}H_{17}FO_4$ $[M–H]^-$ **267.1027** found: **267.1040**.

2-Fluoro-3-(tetrahydro-2H-pyran-4-yl)propanoic acid (63)

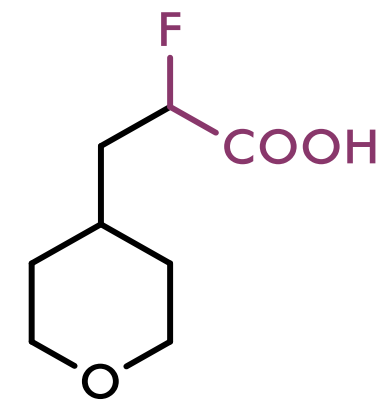

63

Prepared according to the general procedure C. The crude was purified by preparative thin layer chromatography, providing the title compound as a colorless oil (14.1 mg, 80% yield).

$\mathbf{R_f}$ = 0.24 (Hex/EtOAc/AcOH = 3/2/0.1)

^{1}H NMR (600 MHz, $CDCl_3$) δ 8.03 (br, 1H), 5.03 (ddd, *J* = 49.5, 9.1, 2.9 Hz, 1H), 4.06 – 3.97 (m, 2H), 3.50 – 3.38 (m, 2H), 1.98 – 1.78 (m, 3H), 1.77 – 1.71 (m, 1H), 1.68 – 1.62 (m, 1H), 1.49 – 1.35 (m, 2H).

^{13}C NMR (151 MHz, $CDCl_3$) δ 174.6 (d, *J* = 23.9 Hz), 86.8 (d, *J* = 184.9 Hz), 67.8 (d, *J* = 184.9 Hz) , 39.1 (d, *J* = 20.4 Hz), 33.2, 32.1, 31.3 (d, *J* = 1.8 Hz).

^{19}F NMR (564 MHz, $CDCl_3$) δ –191.01 to –191.20 (m).

HRMS (ESI) m/z calcd. for $C_8H_{13}FO_3$ $[M–H]^-$ **175.0765** found: **175.0767**.

3-((Benzoyloxy)methyl)-2-fluoro-4-methylpentanoic acid (64)

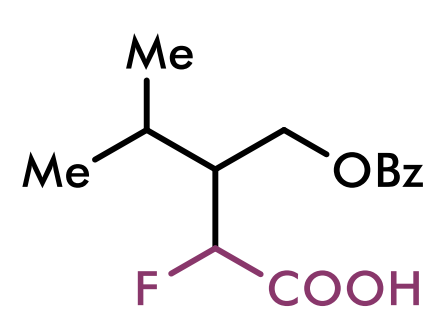

64

Prepared according to the general procedure C. The crude was purified by preparative thin layer chromatography, providing the title compound as a colorless oil (10.7 mg, 40% yield).

$\mathbf{R_f}$ = 0.59 (Hex/EtOAc/AcOH = 3/2/0.1)

^{1}H NMR (600 MHz, CDCl$_3$) mixture of four diastereomers: δ 8.03 – 7.98 (m, 1H), 7.99 – 7.93 (m, 1H), 7.60 – 7.54 (m, 0.49H), 7.54 – 7.50 (m, 0.51H), 7.44 (t, *J* = 7.8 Hz, 1H), 7.39 (t, *J* = 7.8 Hz, 1H), 7.12 – 6.65 (br, 1H), 5.18 – 4.97 (m, 1H), 4.58 – 4.48 (m, 1H), 4.42 – 4.35 (m, 1H), 2.44 – 2.25 (m, 1H), 2.12 – 2.04 (m, 0.49H), 2.01 – 1.93 (m, 0.51H), 1.13 – 1.02 (m, 6H).

^{13}C NMR (151 MHz, CDCl$_3$) mixture of four diastereomers: δ 166.3, 166.3, 133.2, 133.1, 129.8, 129.7, 129.6, 129.6, 128.5, 128.4, 88.3 (d, *J* = 190.3 Hz), 87.2 (d, *J* = 190.3 Hz), 61.6 (d, *J* = 6.3 Hz), 61.3 (d, *J* = 3.3 Hz), 46.5 (d, *J* = 19.6 Hz), 46.4 (d, *J* = 18.1 Hz), 27.8, 26.4, 26.4, 21.5, 20.5, 20.3, 19.3, 19.3.

^{19}F NMR (564 MHz, CDCl$_3$) mixture of four diastereomers: δ –198.14 to –198.57 (m), –202.96 to –203.28 (m).

HRMS (ESI) m/z calcd. for $C_{14}H_{17}FO_4$ $[M–H]^-$ **267.1027** found: **267.1041**.

2-Fluoro-7-((2-(4-isobutylphenyl)propanoyl)oxy)heptanoic acid (65)

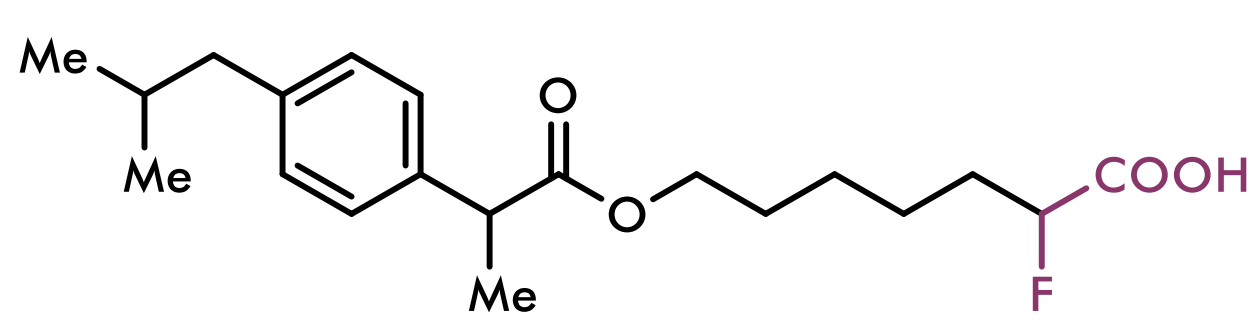

65

Prepared according to the general procedure C using malonic acid **MA-11** (61 mg, 0.5 mmol, 5.0 equiv.). The reaction was performed for 48 hours. The crude was purified by preparative thin layer chromatography, providing the title compound as a colorless oil (15.9 mg, 45% yield).

R$_f$ = 0.59 (Hex/EtOAc/AcOH = 3/2/0.1)

^{1}H NMR (600 MHz, CDCl$_3$) δ 7.95 – 7.28 (m, 1H), 7.19 (d, *J* = 7.7 Hz, 2H), 7.09 (d, *J* = 7.7 Hz, 2H), 4.91 (ddd, *J* = 49.1, 7.3, 4.5 Hz, 1H), 4.15 – 4.00 (m, 2H), 3.68 (q, *J* = 7.1 Hz, 1H), 2.44 (d, *J* = 7.2 Hz, 2H), 1.96 – 1.77 (m, 3H), 1.60 (p, *J* = 7.1 Hz, 2H), 1.51 – 1.42 (m, 5H), 1.35 – 1.26 (m, 2H), 0.89 (d, *J* = 6.6 Hz, 6H).

^{13}C NMR (151 MHz, CDCl$_3$) δ 175.0, 174.8 (d, *J* = 24.3 Hz), 140.5, 137.8, 129.3, 127.2, 88.3 (d,

J = 184.8 Hz), 64.4, 45.2, 45.0, 32.0 (d, *J* = 20.8 Hz), 30.2, 28.2, 25.3, 24.0 (d, *J* = 2.8 Hz), 22.4, 18.4.

^{19}F NMR (564 MHz, CDCl$_3$) δ –191.91 (ddt, *J* = 81.2, 54.0, 25.4 Hz).

HRMS (ESI) m/z calcd. for $C_{20}H_{29}FO_4$ [M–H]$^-$ **351.1966** found: **351.1980**.

7-((4-(N,N-dipropylsulfamoyl)benzoyl)oxy)-2-fluoroheptanoic acid (66)

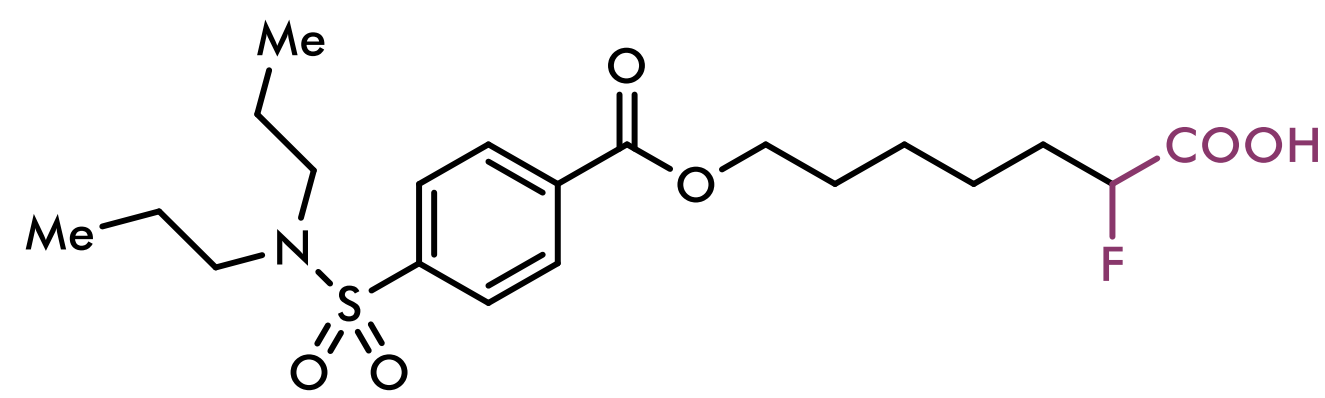

66

Prepared according to the general procedure C using malonic acid **MA-11** (61 mg, 0.5 mmol, 5.0 equiv.). The reaction was performed for 48 hours. The crude was purified by preparative thin layer chromatography, providing the title compound as a colorless oil (28.4 mg, 66% yield).

R$_f$ = 0.35 (Hex/EtOAc/AcOH = 3/2/0.1)

^{1}H NMR (600 MHz, CDCl$_3$) δ 8.14 (d, *J* = 8.5 Hz, 2H), 7.87 (d, *J* = 8.5 Hz, 2H), 7.36 – 6.95 (m, 1H), 4.98 (ddd, *J* = 49.0, 7.4, 4.3 Hz, 1H), 4.36 (t, *J* = 6.6 Hz, 2H), 3.14 – 3.06 (m, 4H), 2.05 – 1.90 (m, 2H), 1.82 (p, *J* = 7.0 Hz, 2H), 1.65 – 1.47 (m, 8H), 0.86 (t, *J* = 7.4 Hz, 6H).

^{13}C NMR (151 MHz, CDCl$_3$) δ 174.9 (d, *J* = 24.4 Hz), 165.5, 144.3, 133.7, 130.3, 127.1, 88.3 (d, *J* = 184.9 Hz), 65.6, 50.0, 32.1 (d, *J* = 20.9 Hz), 28.5, 25.7, 24.2 (d, *J* = 2.8 Hz), 22.0, 11.3.

^{19}F NMR (564 MHz, CDCl$_3$) δ –192.06 (dq, *J* = 58.8, 31.0, 28.4 Hz).

HRMS (ESI) m/z calcd. for $C_{20}H_{30}FNO_6S$ [M–H]$^-$ **430.1694** found: **430.1709**.

7-((2-(4-Chlorophenoxy)-2-methylpropanoyl)oxy)-2-fluoroheptanoic acid (67)

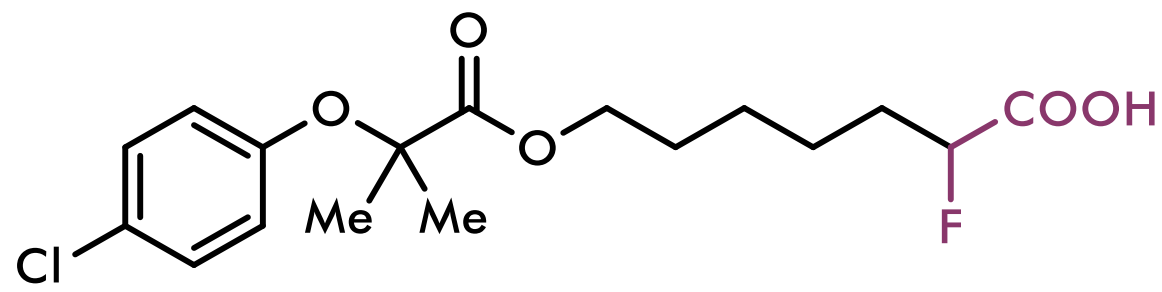

67

Prepared according to the general procedure C. The reaction was performed for 48 hours. The crude was purified by preparative thin layer chromatography, providing the title compound as a colorless oil (22 mg, 61% yield).

R_f = 0.56 (Hex/EtOAc/AcOH = 3/2/0.1)

^{1}H NMR (600 MHz, $CDCl_3$) δ 8.60 – 7.73 (m, 1H), 7.19 (d, *J* = 8.9 Hz, 2H), 6.77 (d, *J* = 8.9 Hz, 2H), 4.92 (ddd, *J* = 49.1, 7.1, 4.7 Hz, 1H), 4.15 (t, *J* = 6.5 Hz, 2H), 1.94 – 1.78 (m, 2H), 1.66 – 1.60 (m, 2H), 1.58 (s, 6H), 1.52 – 1.41 (m, 2H), 1.34 – 1.26 (m, 2H).

^{13}C NMR (151 MHz, $CDCl_3$) δ 175.0 (d, *J* = 24.3 Hz), 174.2, 154.1, 129.2, 127.1, 120.2, 88.2 (d, *J* = 185.0 Hz), 79.5, 65.3, 32.0 (d, *J* = 20.9 Hz), 28.2, 25.3, 25.3, 24.0 (d, *J* = 2.8 Hz).

^{19}F NMR (564 MHz, $CDCl_3$) δ –192.20 (dt, *J* = 51.1, 25.4 Hz).

HRMS (ESI) m/z calcd. for $C_{17}H_{22}ClFO_5$ $[M–H]^-$ **359.1056** found: **359.1073**.

2,2-Difluoro-6-phenylhexanoic acid (68)

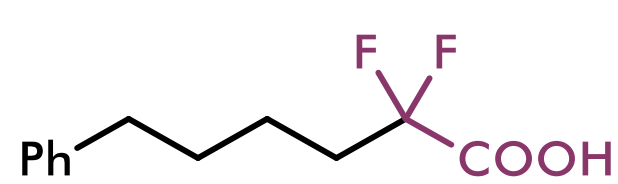

68

Prepared according to the general procedure D. The crude was purified by flash column chromatography, providing the title compound as a colorless oil (18.4 mg, 82% yield).

R_f = 0.5 (Hex/EtOAc/AcOH = 3/2/0.1)

^{1}H NMR (600 MHz, Acetone-*d*$_6$) δ 7.27 (t, *J* = 7.5 Hz, 2H), 7.23 – 7.19 (m, 2H), 7.19 – 7.14 (m, 1H), 2.65 (t, *J* = 7.6 Hz, 2H), 2.21 – 2.10 (m, 2H), 1.75 – 1.67 (m, 2H), 1.56 – 1.48 (m, 2H).

^{13}C NMR (151 MHz, Acetone-*d*$_6$) δ 165.3, 142.1, 128.3, 128.2, 125.7, 116.9 (t, J = 242.0Hz) 35.2, 34.1 (t, *J* = 23.1 Hz), 30.8, 21.2.

^{19}F NMR (564 MHz, Acetone-d_6) δ –105.51 to –107.51 (m).

HRMS (ESI) m/z calcd. for $C_{12}H_{14}F_2O_2$ $[M–H]^-$ **227.0878** found: **227.0886**.

7-(Benzoyloxy)-2,2-difluoroheptanoic acid (69)

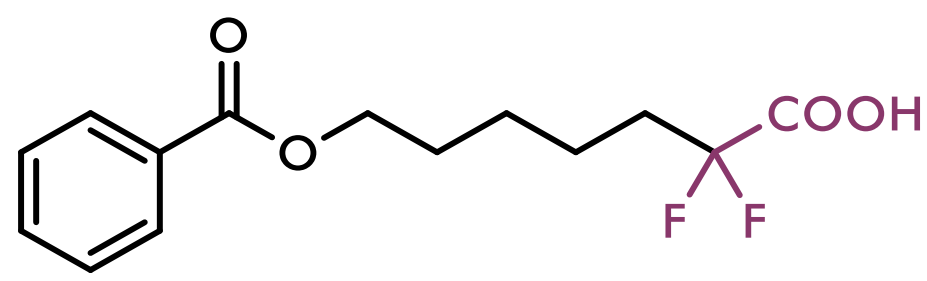

69

Prepared according to the general procedure D. The crude was purified by flash column chromatography, providing the title compound as a colorless oil (17.7 mg, 62% yield).

R$_f$ = 0.24 (Hex/EtOAc/AcOH = 3/2/0.1)

^{1}H NMR (600 MHz, Acetone-d_6) δ 8.07 – 7.99 (m, 2H), 7.66 – 7.59 (m, 1H), 7.51 (t, J = 7.8 Hz, 2H), 5.56 – 4.80 (m, 1H), 4.33 (t, J = 6.5 Hz, 2H), 2.23 – 2.10 (m, 2H), 1.82 (p, J = 6.6 Hz, 2H), 1.62-1.53 (m, 4H).

^{13}C NMR (151 MHz, **Acetone-d_6**) δ 167.7, 134.7, 132.3, 131.1, 130.3, 118.7 (t, J = 247.2 Hz), 66.2, 35.9 (t, J = 23.2 Hz), 30.1, 27.2, 23.1 (t, J = 4.4 Hz).

^{19}F NMR (564 MHz, Acetone-d_6) δ –105.80 to –107.00 (m).

HRMS (ESI) m/z calcd. for $C_{14}H_{16}F_2O_4$ $[M–H]^-$ **285.0933** found: **285.0945**.

2,2-Difluoro-7-(tosyloxy)heptanoic acid (70)

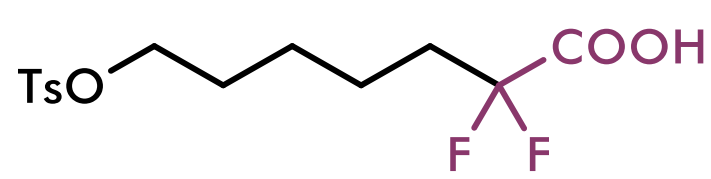

70

Prepared according to the general procedure D. The crude was purified by flash column chromatography, providing the title compound as a colorless oil (13.5 mg, 40% yield).

R$_f$ = 0.15 (Hex/EtOAc/AcOH = 3/2/0.1)

^{1}H NMR (600 MHz, Acetone-d_6) δ 7.94 – 7.74 (m, 2H), 7.49 (d, J = 8.0 Hz, 2H), 5.68 – 5.32 (m, 1H), 4.07 (t, J = 6.3 Hz, 2H), 2.46 (s, 3H), 2.11 – 1.99 (m, 2H), 1.71 – 1.63 (m, 2H), 1.47 –

1.35 (m, 4H).

^{13}C NMR (151 MHz, Acetone-*d*$_6$) δ 166.5 (t, *J* = 32.5 Hz), 146.7, 135.3, 131.8, 129.6, 118.5 (t, *J* = 247.5 Hz), 72.2, 35.7 (t, *J* = 23.1 Hz), 30.1, 26.5, 22.7 (t, *J* = 4.5 Hz), 22.4.

^{19}F NMR (564 MHz, Acetone-*d*$_6$) δ –106.62 (t, *J* = 17.0 Hz).

HRMS (ESI) m/z calcd. for $C_{14}H_{18}F_2O_5S$ [M–H]$^-$ **335.0759** found: **335.0774**.

6-(1,3-Dioxoisoindolin-2-yl)-2,2-difluorohexanoic acid (71)

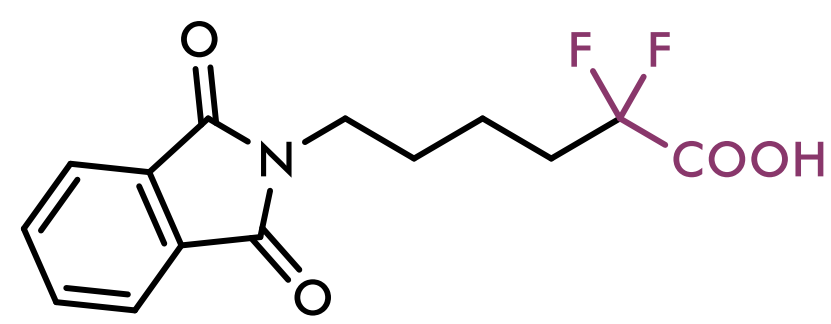

71

Prepared according to the general procedure D. The crude was purified by flash column chromatography, providing the title compound as a colorless oil (12.5 mg, 42% yield).

R$_f$ = 0.26 (Hex/EtOAc/AcOH = 3/2/0.1)

^{1}H NMR (600 MHz, Acetone-*d*$_6$) δ 10.50 – 8.19 (m, 1H), 7.92 – 7.76 (m, 4H), 3.70 (t, *J* = 6.9 Hz, 2H), 2.25 – 2.14 (m, 2H), 1.79 (p, *J* = 7.2 Hz, 2H), 1.61 – 1.51 (m, 2H).

^{13}C NMR (151 MHz, Acetone-*d*$_6$) δ 167.9, 164.6 (t, *J* = 32.5 Hz), 134.1, 132.2, 122.8, 116.6 (t, *J* = 248.2 Hz), 37.1, 33.7 (t, *J* = 23.3 Hz), 27.8, 19.0 (t, *J* = 4.6 Hz).

^{19}F NMR (564 MHz, Acetone-*d*$_6$) δ –106.60 (t, *J* = 16.9 Hz).

HRMS (ESI) m/z calcd. for $C_{14}H_{13}F_2NO_4$ [M–H]$^-$ **296.0729** found: **296.0741**.

2,2-Difluoro-4-methyl-5-phenoxypentanoic acid (72)

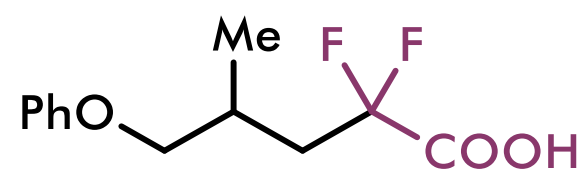

72

Prepared according to the general procedure D. The crude was purified by flash column chromatography, providing the title compound as a colorless oil (16.1 mg, 66% yield).

R$_f$ = 0.35 (Hex/EtOAc/AcOH = 3/2/0.1)

^{1}H NMR (600 MHz, Acetone-*d$_6$*) δ 7.31 – 7.23 (m, 2H), 6.98 – 6.87 (m, 3H), 4.72 – 4.29 (m, 1H), 3.96 – 3.79 (m, 2H), 2.51 – 2.23 (m, 2H), 2.14 – 2.07 (m, 1H), 1.14 (d, *J* = 5.2 Hz, 3H).

^{13}C NMR (151 MHz, Acetone-*d$_6$*) δ 160.9, 131.2, 122.3, 116.3, 73.7, 39.4 (t, d = 22.0 Hz), 25.0 18.7.

^{19}F NMR (564 MHz, Acetone-*d$_6$*) δ –101.96 to –105.00 (m).

HRMS (ESI) m/z calcd. for $C_{12}H_{14}F_2O_3$ $[M–H]^-$ **243.0827** found: **243.0836**.

1-(5-(Benzoyloxy)pentyl)cyclobutane-1-carboxylic acid (73)

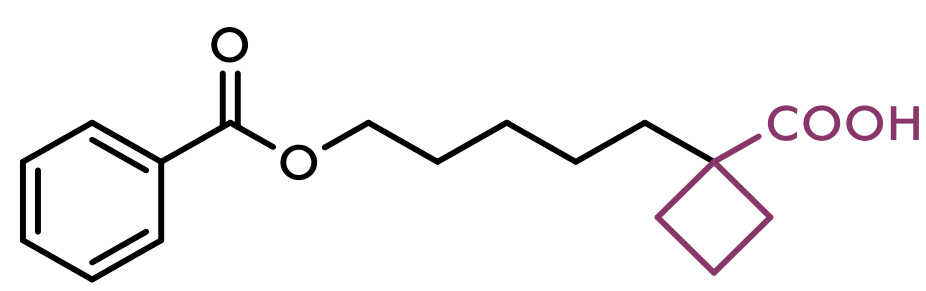

73

Prepared according to the general procedure E. The crude was purified by preparative thin layer chromatography, providing the title compound as a colorless oil (20 mg, 69% yield).

R$_f$ = 0.14 (Hex/EtOAc = 2/0.5)

^{1}H NMR (600 MHz, CDCl$_3$) δ 8.07 – 8.00 (m, 2H), 7.59 – 7.52 (m, 1H), 7.46 – 7.40 (m, 2H), 4.31 (t, *J* = 6.6 Hz, 2H), 2.51 – 2.41 (m, 2H), 1.96 – 1.87 (m, 4H), 1.85 – 1.74 (m, 4H), 1.51 – 1.42 (m, 2H), 1.37 – 1.28 (m, 2H).

^{13}C NMR (151 MHz, CDCl$_3$) δ 183.8, 166.8, 132.9, 130.6, 129.7, 128.5, 65.2, 47.6, 37.9, 30.1, 28.8, 26.5, 24.7, 15.7.

HRMS (ESI) m/z calcd. for $C_{17}H_{22}O_4$ $[M–H]^-$ **289.1434** found: **289.1449**.

1-(5-(Benzoyloxy)pentyl)cyclohexane-1-carboxylic acid (74)

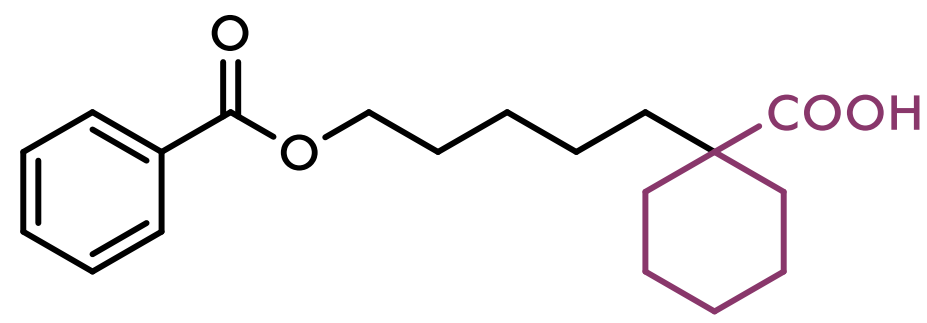

74

Prepared according to the general procedure E. The crude was purified by preparative thin layer chromatography, providing the title compound as a colorless oil (8.9 mg, 35% yield).

R_f = 0.24 (Hex/EtOAc = 2/0.5)

^{1}H NMR (600 MHz, $CDCl_3$) δ 8.03 (d, *J* = 7.6 Hz, 2H), 7.55 (t, *J* = 7.5 Hz, 1H), 7.43 (t, *J* = 7.6 Hz, 2H), 4.30 (t, *J* = 6.6 Hz, 2H), 2.10 – 2.00 (m, 2H), 1.79 – 1.73 (m, 2H), 1.60 – 1.53 (m, 4H), 1.47 – 1.32 (m, 6H), 1.27 – 1.21 (m, 4H).

^{13}C NMR (151 MHz, $CDCl_3$) δ 183.3, 166.8, 132.9, 130.6, 129.7, 128.5, 65.2, 46.9, 34.0, 28.8, 26.7, 26.0, 23.9, 23.3.

HRMS (ESI) m/z calcd. for $C_{19}H_{26}O_4$ $[M-H]^-$ **317.1747** found: **317.1760**.

4-(5-(Benzoyloxy)pentyl)tetrahydro-2H-pyran-4-carboxylic acid (75)

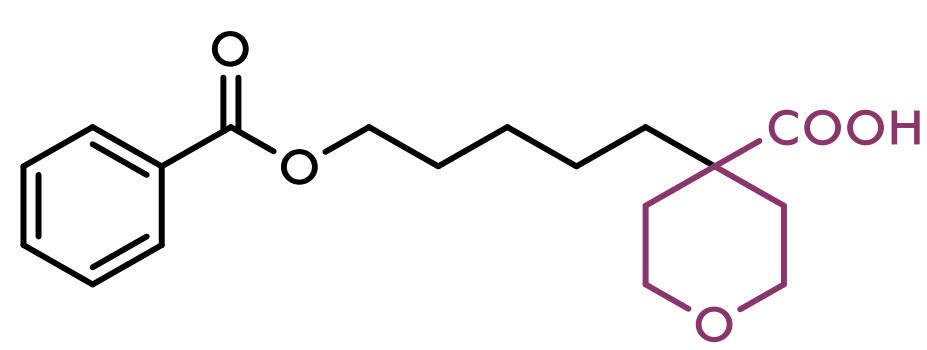

75

Prepared according to the general procedure E. The crude was purified by preparative thin layer chromatography, providing the title compound as a colorless oil (9.9 mg, 31% yield).

R_f = 0.37 (Hex/EtOAc = 2/0.5)

^{1}H NMR (600 MHz, $CDCl_3$) δ 8.03 (d, *J* = 7.7 Hz, 2H), 7.56 (t, *J* = 7.4 Hz, 1H), 7.44 (td, *J* = 7.9, 2.1 Hz, 2H), 4.31 (td, *J* = 6.6, 2.1 Hz, 2H), 3.85 (d, *J* = 11.9 Hz, 2H), 3.51 (t, *J* = 11.7 Hz, 2H), 2.10 – 2.04 (m, 2H), 1.81 – 1.73 (m, 2H), 1.63 – 1.57 (m, 2H), 1.56 – 1.50 (m, 2H), 1.47 – 1.39 (m, 2H), 1.38 – 1.32 (m, 2H).

^{13}C NMR (151 MHz, CDCl$_3$) δ 181.4, 166.8, 133.0, 130.5, 129.7, 128.5, 65.5, 65.0, 44.9, 40.7, 34.1, 28.7, 26.5, 23.5.
HRMS (ESI) m/z calcd. for $C_{18}H_{24}O_5$ [M–H]$^-$ **319.1540** found: **319.1553**.

4-(5-(Benzoyloxy)pentyl)-1-(phenylsulfonyl)piperidine-4-carboxylic acid (76)

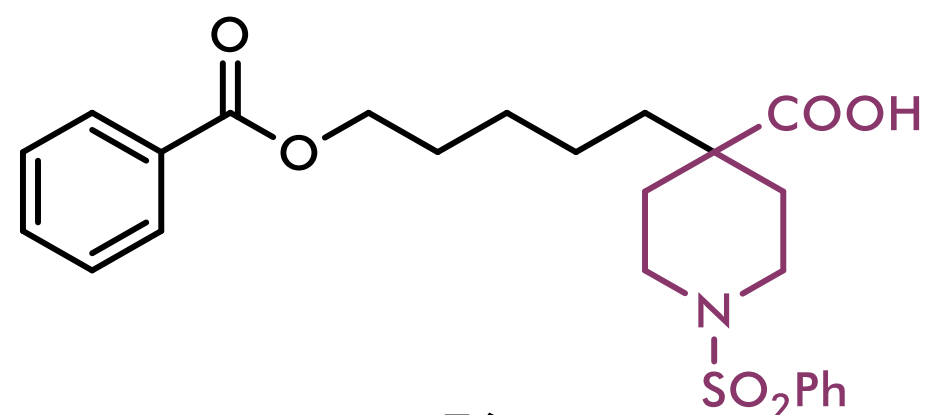

76

Prepared according to the general procedure E. The crude was purified by preparative thin layer chromatography, providing the title compound as a colorless oil (18.4 mg, 40% yield).
R$_f$ = 0.27 (Hex/EtOAc = 1/1)
^{1}H NMR (600 MHz, CDCl$_3$) δ 8.05 – 7.99 (m, 2H), 7.77 – 7.70 (m, 2H), 7.60 – 7.54 (m, 2H), 7.53 – 7.48 (m, 2H), 7.46 – 7.41 (m, 2H), 4.28 (t, *J* = 6.6 Hz, 2H), 3.59 (dt, *J* = 12.9, 3.8 Hz, 2H), 2.49 (td, *J* = 12.1, 2.6 Hz, 2H), 2.22 – 2.11 (m, 2H), 1.78 – 1.67 (m, 2H), 1.59 – 1.46 (m, 4H), 1.42 – 1.34 (m, 2H), 1.29 – 1.25 (m, 2H).
^{13}C NMR (151 MHz, CDCl$_3$) δ 179.4, 166.7, 136.6, 132.9, 132.7, 130.3, 129.5, 129.1, 128.4, 127.6, 64.8, 44.8, 43.7, 39.9, 32.7, 28.5, 26.3, 23.5.
HRMS (ESI) m/z calcd. for $C_{24}H_{29}NO_6S$ [M–H]$^-$ **458.1632** found: **458.1651**.

2-(5-Fluoropentyl-5-d)isoindoline-1,3-dione (77)

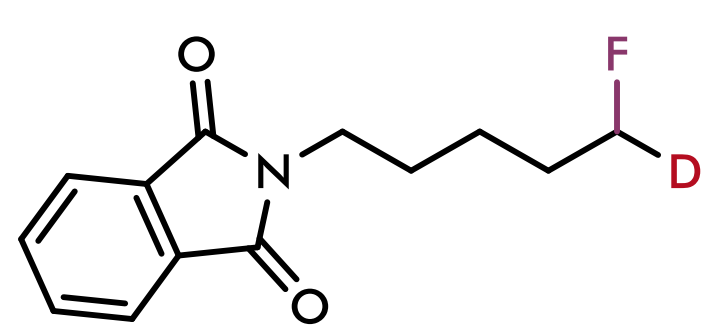

77

Prepared according to the procedure for decarboxylative deuteration. The crude was purified by preparative thin layer chromatography, providing the title compound as a

colorless oil (14.2 mg, 60% yield, 85% D).

R$_f$ = 0.5 (Hex/EtOAc = 2/0.5)

^{1}H NMR (600 MHz, CDCl$_3$) δ 7.83 (dd, *J* = 5.4, 3.1 Hz, 2H), 7.71 (dd, *J* = 5.5, 3.0 Hz, 2H), 4.42 (ddt, *J* = 47.3, 11.9, 6.0 Hz, 1.19H), 3.70 (t, *J* = 7.2 Hz, 2H), 1.78 – 1.68 (m, 4H), 1.52 – 1.41 (m, 2H).

^{13}C NMR (151 MHz, CDCl$_3$) δ 168.4, 133.9, 132.1, 123.2, 83.4 (dt, *J* = 164.0, 23.1 Hz), 37.8, 29.8 (d, *J* = 19.8 Hz), 28.2, 22.6 (d, *J* = 5.0 Hz).

^{19}F NMR (564 MHz, CDCl$_3$) δ –218.11 to –218.73 (m), –218.93 to –219.33 (m).

HRMS (APCI) m/z calcd. for $C_{13}H_{13}DFNO_2$ $[M+H]^+$ **237.1144** found: **237.1137**.

2-(5-Chloro-5-fluoropentyl)isoindoline-1,3-dione (78)

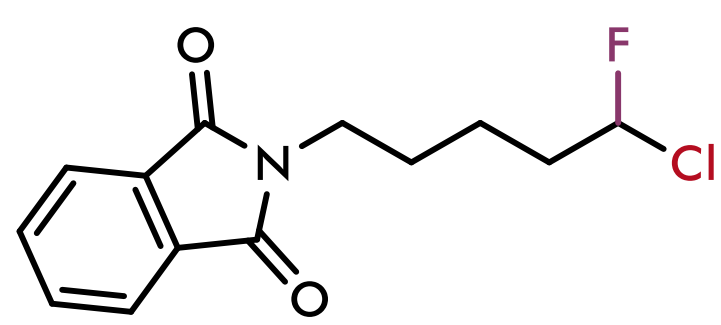

78

Prepared according to the procedure for decarboxylative halogenation of monofluorocarboxylic acid. The crude was purified by preparative thin layer chromatography, providing the title compound as a colorless oil (11.8 mg, 44% yield).

R$_f$ = 0.47 (Hex/EtOAc = 2/0.5)

^{1}H NMR (600 MHz, CDCl$_3$) δ 7.84 (dd, *J* = 5.4, 3.1 Hz, 2H), 7.72 (dd, *J* = 5.5, 3.0 Hz, 2H), 6.15 (dt, *J* = 50.9, 5.3 Hz, 1H), 3.71 (t, *J* = 7.2 Hz, 2H), 2.20 – 2.02 (m, 2H), 1.75 (p, *J* = 7.5 Hz, 2H), 1.60 – 1.52 (m, 2H).

^{13}C NMR (151 MHz, CDCl$_3$) δ 168.4, 134.0, 132.1, 123.3, 102.4 (d, *J* = 241.6 Hz), 38.7 (d, *J* = 20.1 Hz), 37.5, 27.8, 21.4 (d, *J* = 4.3 Hz).

^{19}F NMR (564 MHz, CDCl$_3$) δ –130.15 (dt, *J* = 50.9, 18.2 Hz).

HRMS (APCI) m/z calcd. for $C_{13}H_{13}ClFNO_2$ $[M+H]^+$ **270.0692** found: **270.0683**.

2-(5-Bromo-5-fluoropentyl)isoindoline-1,3-dione (78')

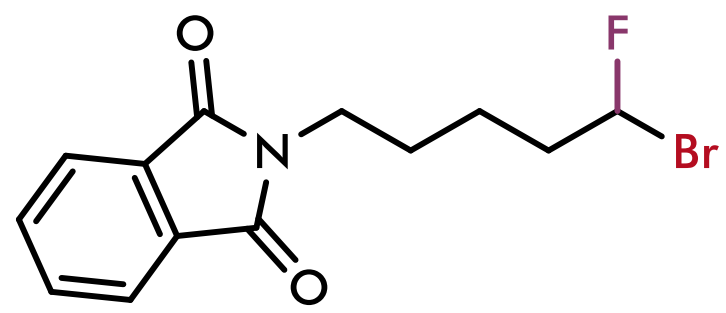

78'

Prepared according to the procedure for decarboxylative halogenation of monofluorocarboxylic acid. The crude was purified by preparative thin layer chromatography, providing the title compound as a colorless oil (27 mg, 86% yield).

R_f = 0.5 (Hex/EtOAc = 2/0.5)

^{1}H NMR (600 MHz, $CDCl_3$) δ 7.84 (dd, *J* = 5.4, 3.0 Hz, 2H), 7.71 (dd, *J* = 5.5, 3.0 Hz, 2H), 6.44 (dt, *J* = 50.3, 5.3 Hz, 1H), 3.70 (t, *J* = 7.2 Hz, 2H), 2.33 – 2.14 (m, 2H), 1.75 (p, *J* = 7.6 Hz, 2H), 1.61 – 1.52 (m, 2H).

^{13}C NMR (151 MHz, $CDCl_3$) δ 168.4, 134.0, 132.1, 123.3, 95.0 (d, *J* = 252.4 Hz), 40.0 (d, *J* = 19.0 Hz), 37.4, 27.7, 22.3 (d, *J* = 4.1 Hz).

^{19}F NMR (564 MHz, $CDCl_3$) δ –130.96 (dt, *J* = 50.2, 19.0 Hz).

HRMS (APCI) m/z calcd. for $C_{13}H_{13}BrFNO_2$ $[M+H]^+$ **315.0220** found: **315.0212**.

Benzyl 8-(1,3-dioxoisoindolin-2-yl)-4-fluorooctanoate (79)

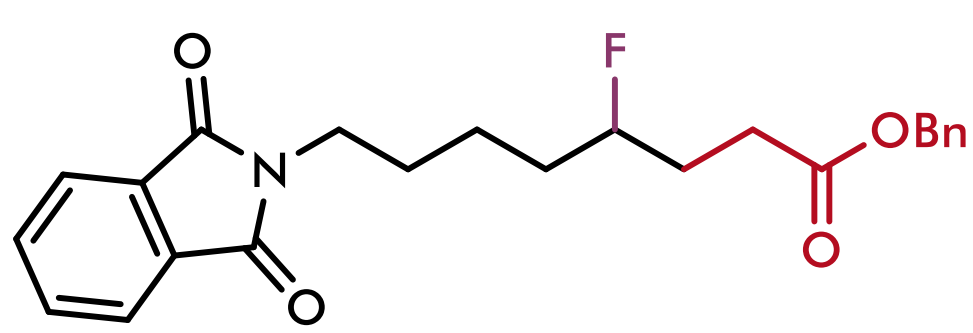

79

Prepared according to the procedure for decarboxylative monofluoroalkylation. The crude was purified by preparative thin layer chromatography, providing the title compound as a colorless oil (25.8 mg, 65% yield).

R_f = 0.3 (Hex/EtOAc = 2/0.5)

^{1}H NMR (600 MHz, $CDCl_3$) δ 7.84 (dd, *J* = 5.4, 3.1 Hz, 2H), 7.71 (dd, *J* = 5.4, 3.0 Hz, 2H), 7.39 – 7.30 (m, 5H), 5.12 (d, *J* = 1.7 Hz, 2H), 4.59 – 4.36 (m, 1H), 3.69 (t, *J* = 7.2 Hz, 2H), 2.59 – 2.41

(m, 2H), 1.98 – 1.86 (m, 2H), 1.75 – 1.65 (m, 3H), 1.63 – 1.58 (m, 1H), 1.55 – 1.48 (m, 1H), 1.45 – 1.37 (m, 1H).

^{13}C NMR (151 MHz, CDCl$_3$) δ 172.9, 168.4, 135.9, 133.9, 132.1, 128.6, 128.3, 128.3, 123.2, 93.0 (d, *J* = 168.7 Hz), 66.4, 37.7, 34.6 (d, *J* = 20.6 Hz), 30.3 (d, *J* = 21.3 Hz), 29.9 (d, *J* = 4.2 Hz), 28.4, 22.4 (d, *J* = 4.4 Hz).

^{19}F NMR (564 MHz, CDCl$_3$) δ –182.97 to –183.55 (m).

HRMS (APCI) m/z calcd. for $C_{23}H_{24}FNO_4$ [M+H]$^+$ **398.1762** found: **398.1751**.

2-(5-Chloro-5,5-difluoropentyl)isoindoline-1,3-dione (80)

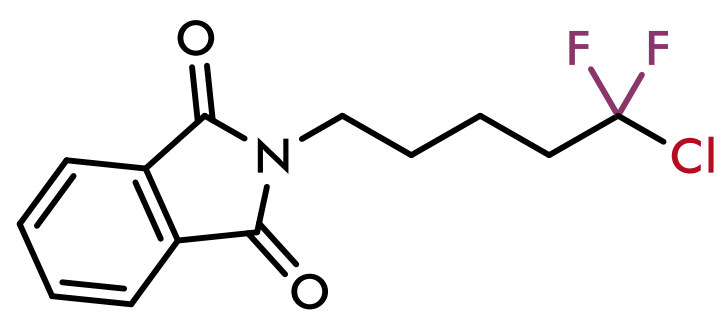

80

Prepared according to the procedure for decarboxylative halogenation of difluorocarboxylic acid. The crude was purified by preparative thin layer chromatography, providing the title compound as a colorless oil (12 mg, 42% yield).

R$_f$ = 0.53 (Hex/EtOAc = 2/0.5)

^{1}H NMR (600 MHz, CDCl$_3$) δ 7.87 – 7.83 (m, 2H), 7.75 – 7.69 (m, 2H), 3.72 (t, *J* = 7.1 Hz, 2H), 2.42 – 2.29 (m, 2H), 1.82 – 1.73 (m, 2H), 1.73 – 1.62 (m, 2H).

^{13}C NMR (151 MHz, CDCl$_3$) δ 168.4, 134.0, 132.0, 129.7 (t, *J* = 291.7 Hz), 123.3, 41.3 (t, *J* = 24.2 Hz), 37.3, 27.5, 20.6 (t, *J* = 3.1 Hz).

^{19}F NMR (564 MHz, CDCl$_3$) δ -50.70 (t, *J* = 12.9 Hz).

HRMS (APCI) m/z calcd. for $C_{13}H_{12}ClF_2NO_2$ [M+H]$^+$ **288.0597** found: **288.0591**.

2-(5-Bromo-5,5-difluoropentyl)isoindoline-1,3-dione (80')

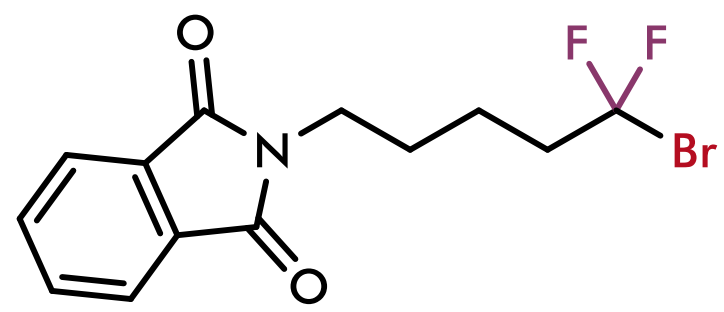

80'

Prepared according to the procedure for decarboxylative halogenation of difluorocarboxylic acid. The crude was purified by preparative thin layer chromatography, providing the title compound as a colorless oil (29.2 mg, 88% yield).

R_f = 0.57 (Hex/EtOAc = 2/0.5)

^{1}H NMR (600 MHz, $CDCl_3$) δ 7.85 (dd, *J* = 5.5, 3.1 Hz, 2H), 7.72 (dd, *J* = 5.6, 3.0 Hz, 2H), 3.71 (t, *J* = 7.1 Hz, 2H), 2.48 – 2.33 (m, 2H), 1.81–1.74 (m, 2H), 1.73 – 1.63 (m, 2H).

^{13}C NMR (151 MHz, $CDCl_3$) δ 168.4, 134.0, 132.0, 123.3, 122.7 (t, *J* = 305.1 Hz), 43.7 (t, *J* = 21.6 Hz), 37.3, 27.4, 21.3 (t, *J* = 3.2 Hz).

^{19}F NMR (564 MHz, $CDCl_3$) δ –43.66 (t, *J* = 13.7 Hz).

HRMS (APCI) m/z calcd. for $C_{13}H_{12}BrF_2NO_2$ $[M+H]^+$ **333.0126** found: **333.0119**.

10-(1,3-Dioxoisoindolin-2-yl)-6,6-difluorodecyl benzoate (81)

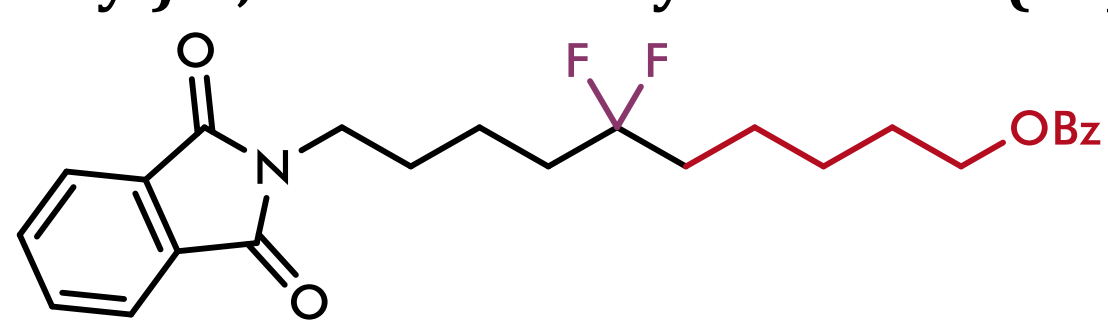

81

Prepared according to the procedure for decarboxylative difluoroalkylation. The crude was purified by preparative thin layer chromatography, providing the title compound as a colorless oil (15.9 mg, 36% yield).

R_f = 0.27 (Hex/EtOAc = 2/0.5)

^{1}H NMR (600 MHz, $CDCl_3$) δ 8.06 – 8.01 (m, 2H), 7.87 – 7.81 (m, 2H), 7.74 – 7.67 (m, 2H), 7.58 – 7.52 (m, 1H), 7.47 – 7.39 (m, 2H), 4.32 (t, *J* = 6.6 Hz, 2H), 3.70 (t, *J* = 7.2 Hz, 2H), 1.92 – 1.76 (m, 6H), 1.76 – 1.70 (m, 2H), 1.58 – 1.43 (m, 6H).

^{13}C NMR (151 MHz, $CDCl_3$) δ 168.4, 166.6, 134.0, 132.9, 132.1, 130.4, 129.5, 128.4, 124.9 (t,

J = 240.6 Hz), 123.2, 64.8, 37.6, 36.3 (t, J = 25.4 Hz), 35.9 (t, J = 25.7 Hz), 28.6, 28.3, 25.9, 35.9 (t, J = 25.7 Hz), 22.0 (t, J = 4.5 Hz), 19.6 (t, J = 4.6 Hz).

^{19}F NMR (564 MHz, CDCl$_3$) δ –98.32 (p, J = 16.6 Hz).

HRMS (APCI) m/z calcd. for $C_{25}H_{27}F_2NO_4$ [M+H]$^+$ **444.1981** found: **444.1967**.

***tert*-Butyl-2-(4-(4-(methylsulfonyl)phenoxy)butyl)-7-azaspiro[3.5]nonane-7-carboxylate (82)**

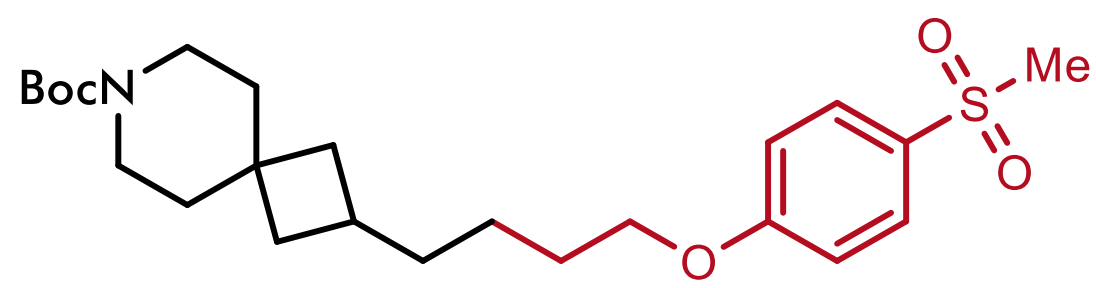

82

Prepared according to the procedure A using using $Fe(NO_3)_3 \cdot 9H_2O$ (60 mg, 0.15 mmol, 15 mol%), Na_2CO_3 (11 mg, 0.1 mmol, 10 mol%), bis(2,4,6-triisopropylphenyl)disulfide (94 mg, 0.2 mmol, 20 mol%), malonic acid **MA-17** (906 mg, 3 mmol, 3.0 equiv.), and alkene **A38** (237 mg, 1 mmol, 1 equiv.) in MeCN/H_2O (5 mL/5 mL). The crude was purified by preparative thin layer chromatography, providing the title compound as a white solid (248.4 mg, 55% yield).

R_f = 0.11 (Hex/EtOAc = 2/0.5)

^{1}H NMR (600 MHz, CDCl$_3$) δ 7.85 (d, J = 8.9 Hz, 2H), 6.99 (d, J = 8.9 Hz, 2H), 4.01 (t, J = 6.5 Hz, 2H), 3.33 (t, J = 5.7 Hz, 2H), 3.25 (t, J = 5.7 Hz, 2H), 3.02 (s, 3H), 2.21 (p, J = 8.0 Hz, 1H), 1.98 – 1.91 (m, 2H), 1.78 (p, J = 6.7 Hz, 2H), 1.57 – 1.50 (m, 2H), 1.49 – 1.32 (m, 17H).

^{13}C NMR (151 MHz, CDCl$_3$) δ 163.3, 155.0, 132.0, 129.5, 114.9, 114.9, 79.2, 68.5, 44.9, 38.0, 37.5, 34.0, 29.7, 29.0, 28.7, 28.5, 23.7.

HRMS (APCI) m/z calcd. for $C_{24}H_{37}NO_5S$ [M+H]$^+$ 452.2465 found: 452.2454.

3-(2-Fluoroethyl)-4-methyl-1-tosylpyrrolidine (84)

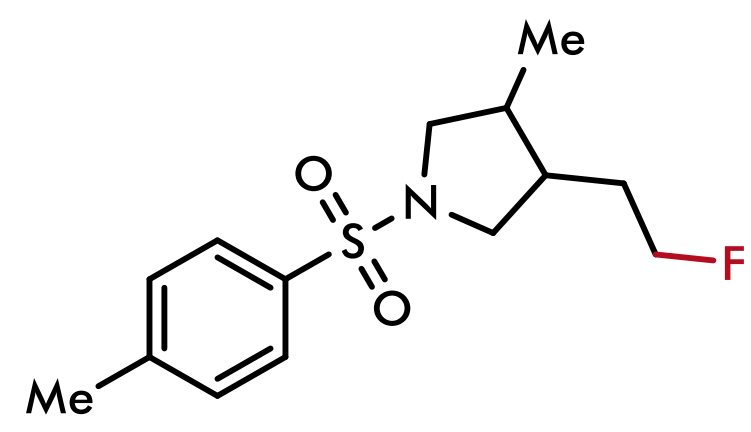

84

Prepared according to the general procedure B using $Fe(NO_3)_3 \cdot 9H_2O$ (4.0 mg, 0.01 mmol, 10 mol%), Na_2CO_3 (1.1 mg, 0.01 mmol, 10 mol%), bis(2,4,6-triisopropylphenyl)disulfide (9.4 mg, 0.02 mmol, 20 mol%), malonic acid **MA-11** (36.6 mg, 0.3 mmol, 3.0 equiv.), and alkene **A30** (25.1 mg, 0.1 mmol, 1.0 equiv.) in $MeCN/H_2O$ (0.9 mL/0.1 mL). The crude was purified by preparative thin layer chromatography, providing the title compound as a colorless oil (17.7 mg, 62% yield, 1:3 dr).

$\mathbf{R_f}$ = 0.16 (Hex/EtOAc = 2/0.5)

^{1}H NMR (600 MHz, $CDCl_3$) mixture of diastereomers, minor diastereomer in brackets [ ]: δ 7.71 (d, *J* = 8.2 Hz, 2H), [7.71], 7.32 (d, *J* = 8.2 Hz, 2H), [7.32], 4.45 – 4.41 (m, 1H), [4.45 – 4.41], 4.38 – 4.33 (m, 1H), [4.38 – 4.33], 3.42 (dd, *J* = 10.1, 7.5 Hz, 1H), [3.56], 3.34 (ddd, *J* = 9.7, 6.3, 3.4 Hz, 1H), [3.50], 3.06 (dd, *J* = 9.8, 3.4 Hz, 1H), [2.77], 3.00 (ddd, *J* = 13.0, 9.0, 4.3 Hz, 1H), [2.90], δ 2.43 (s, 3H), [2.43], δ 2.27 – 2.17 (m, 1H), 2.18 – 2.11 (m, 1H), 1.78 – 1.63 (m, 2H), [δ 1.91 – 1.79 (m, 1H)], [1.53 – 1.41 (m, 3H)], 0.74 (d, *J* = 7.0 Hz, 3H), [0.94].

^{13}C NMR (151 MHz, $CDCl_3$) δ 143.4, [143.4], 134.1, [133.9], [129.7], 129.6, [127.5], 127.4, 83.2, [83.2], 82.1, [82.1], 54.7, [54.5], [53.2], 50.8, [42.8], [42.8], [38.9], 38.6, 38.6, 35.3, [32.6], [32.5], [29.7], 28.6, 28.5, 21.5, [16.1], 13.2.

^{19}F NMR (564 MHz, $CDCl_3$) δ –219.36 (tt, *J* = 47.5, 26.2 Hz), [–218.38].

HRMS (APCI) m/z calcd. for $C_{14}H_{20}FNO_2S$ $[M+H]^+$ **286.1272** found: **286.1263**.

2-Methyl-3-(4-methyl-1-tosylpyrrolidin-3-yl)propanoic acid (85)

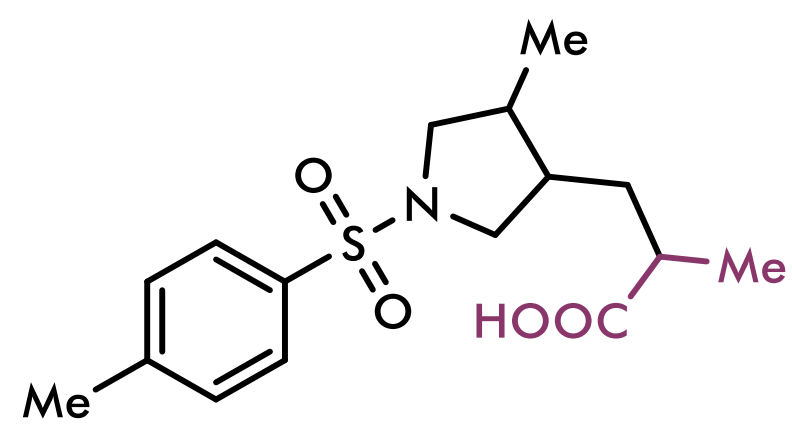

85

Prepared according to the general procedure A using $Fe(NO_3)_3 \cdot 9H_2O$ (6.0 mg, 0.015 mmol, 15 mol%), Na_2CO_3 (1.1 mg, 0.01 mmol, 10 mol%), $HSCH_2CH_2SO_3Na$ (3.3 mg, 0.02 mmol, 20 mol%), malonic acid **MA-1** (47.2 mg, 0.4 mmol, 4.0 equiv.), and alkene **A30** (25.1 mg, 0.1 mmol, 1.0 equiv.) in $MeCN/H_2O$ (0.1 mL/0.1 mL). The crude was purified by preparative thin layer chromatography, providing the title compound as a yellowish oil (11.3 mg, 35% yield).

$\mathbf{R_f}$ = 0.21 (Hex/EtOAc = 1/1)

^{1}H NMR (600 MHz, $CDCl_3$) mixture of diastereomers: δ 7.86 – 7.62 (m, 2H), 7.36 – 7.31 (m, 2H), 3.68 – 3.26 (m, 2H), 3.19 – 2.64 (m, 2H), 2.54 – 0.61 (m, 14H).

^{13}C NMR (151 MHz, $CDCl_3$) mixture of four diastereomers: δ 206.9, 143.4, 134.1, 133.9, 130.1, 129.8, 129.7, 129.7, 127.9, 127.6, 127.5, 127.4, 127.4, 127.3, 54.9, 54.6, 54.3, 54.2, 53.1, 51.7, 50.9, 50.7, 46.1, 43.6, 41.6, 40.7, 39.7, 39.6, 39.1, 38.5, 37.7, 36.8, 35.4, 34.9, 34.8, 31.7, 31.5, 30.2, 29.7, 21.6, 21.5, 17.7, 17.2, 16.2, 13.4, 13.1, 13.0.

HRMS (ESI) m/z calcd. for $C_{16}H_{23}NO_4S$ $[M+H]^+$ **326.1426** found: **326.1417**.

3-Methylhexyl-3,5-*d₂* benzoate (86)

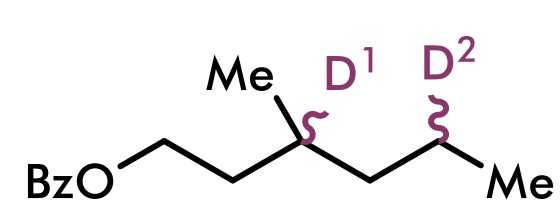

86

Prepared according to the general procedure A using $Fe(NO_3)_3 \cdot 9H_2O$ (6.0 mg, 0.015 mmol, 15 mol%), Na_2CO_3 (1.1 mg, 0.01 mmol, 10 mol%), bis(2,4,6-triisopropylphenyl)disulfide (11.4 mg, 0.025 mmol, 25 mol%), malonic acid **MA-1** (47.2 mg, 0.4 mmol, 4.0 equiv.), and

alkene **A9** (19 mg, 0.1 mmol, 1.0 equiv.) in MeCN/D_2O (0.5 mL/0.5 mL). The crude was purified by preparative thin layer chromatography, providing the title compound as a pale-yellow oil (19.8 mg, 89% yield).

R_f = 0.65 (Hex/EtOAc = 2/0.5)

^{1}H NMR (600 MHz, $CDCl_3$) mixture of diastereomers: δ 8.04 (d, *J* = 7.4 Hz, 2H), 7.57 – 7.52 (m, 1H), 7.44 (t, *J* = 7.6 Hz, 2H), 4.36 (q, *J* = 6.5 Hz, 2H), 1.82 – 1.77 (m, 1H), 1.69 – 1.61 (m, 0.3 H), 1.60 – 1.55 (m, 1H), 1.37 – 1.28 (m, 2.1H), 1.24 – 1.16 (m, 1H), 0.95 (s, 2H), 0.88 (s, 3H).

^{13}C NMR (151 MHz, $CDCl_3$) mixture of diastereomers: δ 166.7, 132.8, 130.6, 129.5, 128.3, 63.6, 39.1, 39.0, 39.0, 38.9, 35.8, 35.7, 35.6, 35.6, 35.5, 29.7, 29.7, 29.7, 29.3, 29.2, 29.1, 19.7, 19.6, 19.6, 19.5, 19.5, 19.3, 19.2, 19.1, 19.1, 19.0, 14.3, 14.3, 14.2, 14.2, 14.1, 14.1.

HRMS (APCI) m/z calcd. for $C_{14}H_{18}D_2O_2$ $[M+H]^+$ **222.1570** found: **222.1535**.

7-(Benzoyloxy)-2-methylheptanoic acid (2')

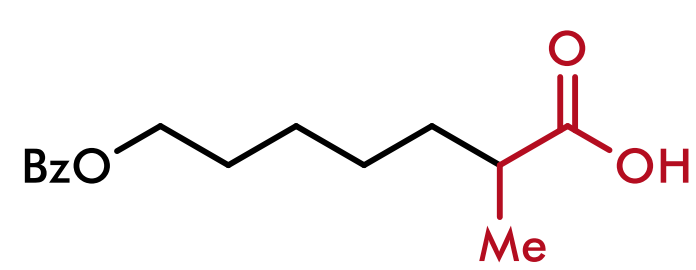

2'

Byproduct of model reaction in optimization section. **2'** was isolated by preparative thin layer chromatography, providing the title compound as a colorless oil.

R_f = 0.15 (Hex/EtOAc = 2/0.5)

^{1}H NMR (600 MHz, $CDCl_3$) δ 8.04 (dd, *J* = 8.3, 1.4 Hz, 2H), 7.55 (td, *J* = 7.3, 1.2 Hz, 1H), 7.44 (t, *J* = 7.7 Hz, 2H), 4.31 (t, *J* = 6.6 Hz, 2H), 2.51 – 2.45 (m, 1H), 1.81 – 1.69 (m, 3H), 1.51 – 1.38 (m, 5H), 1.19 (d, *J* = 7.0 Hz, 3H).

^{13}C NMR (151 MHz, $CDCl_3$) δ 182.9, 166.7, 132.9, 130.4, 129.5, 128.3, 65.0, 39.3, 33.4, 28.6, 26.9, 26.0, 16.9.

HRMS (APCI) m/z calcd. for $C_{15}H_{20}O_4$ $[M-H]^+$ **263.1278** found: **263.1287**.

NMR Spectra

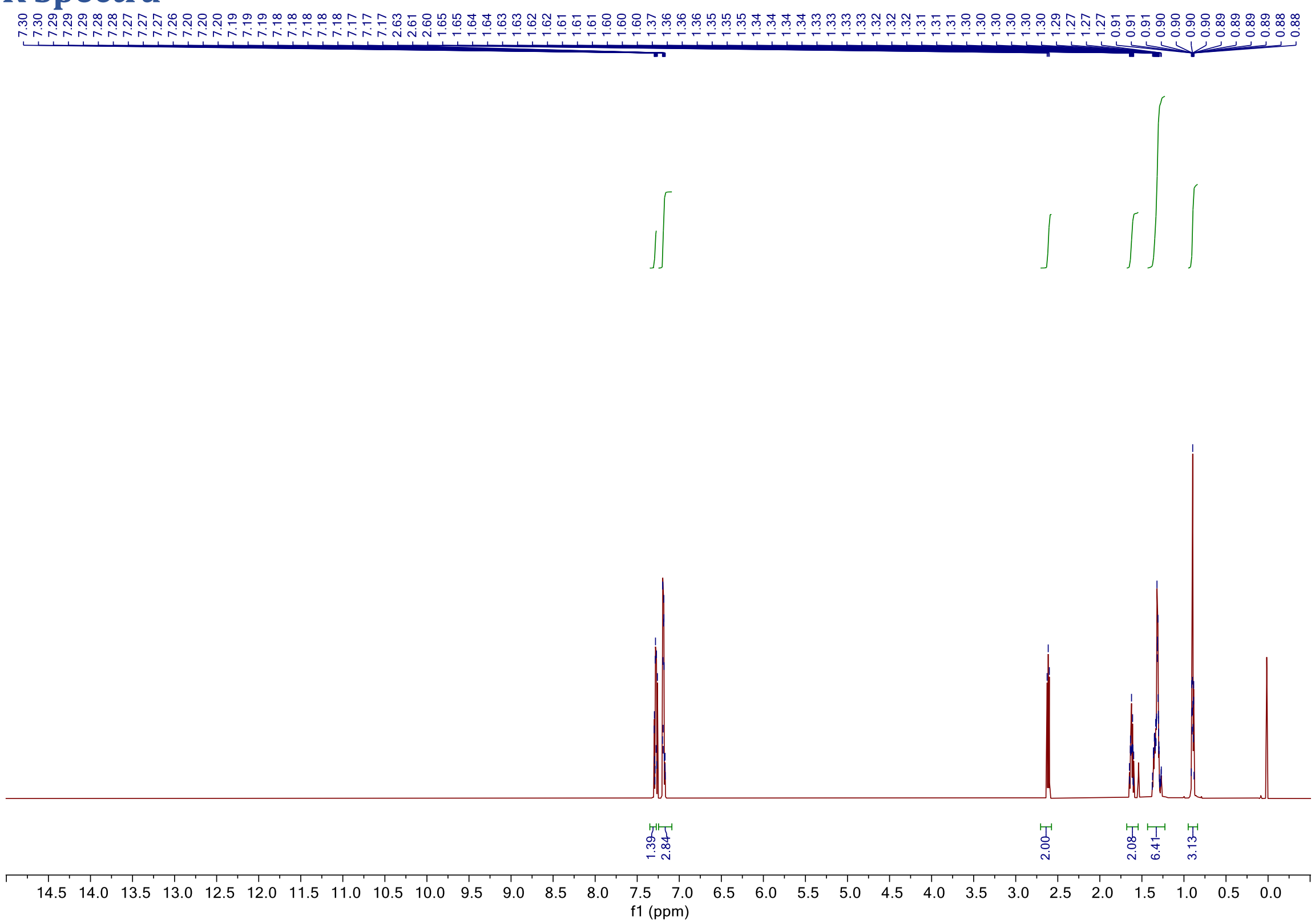

Supplementary Figure 21. ^{1}H NMR (600 MHz) of Compound (1)

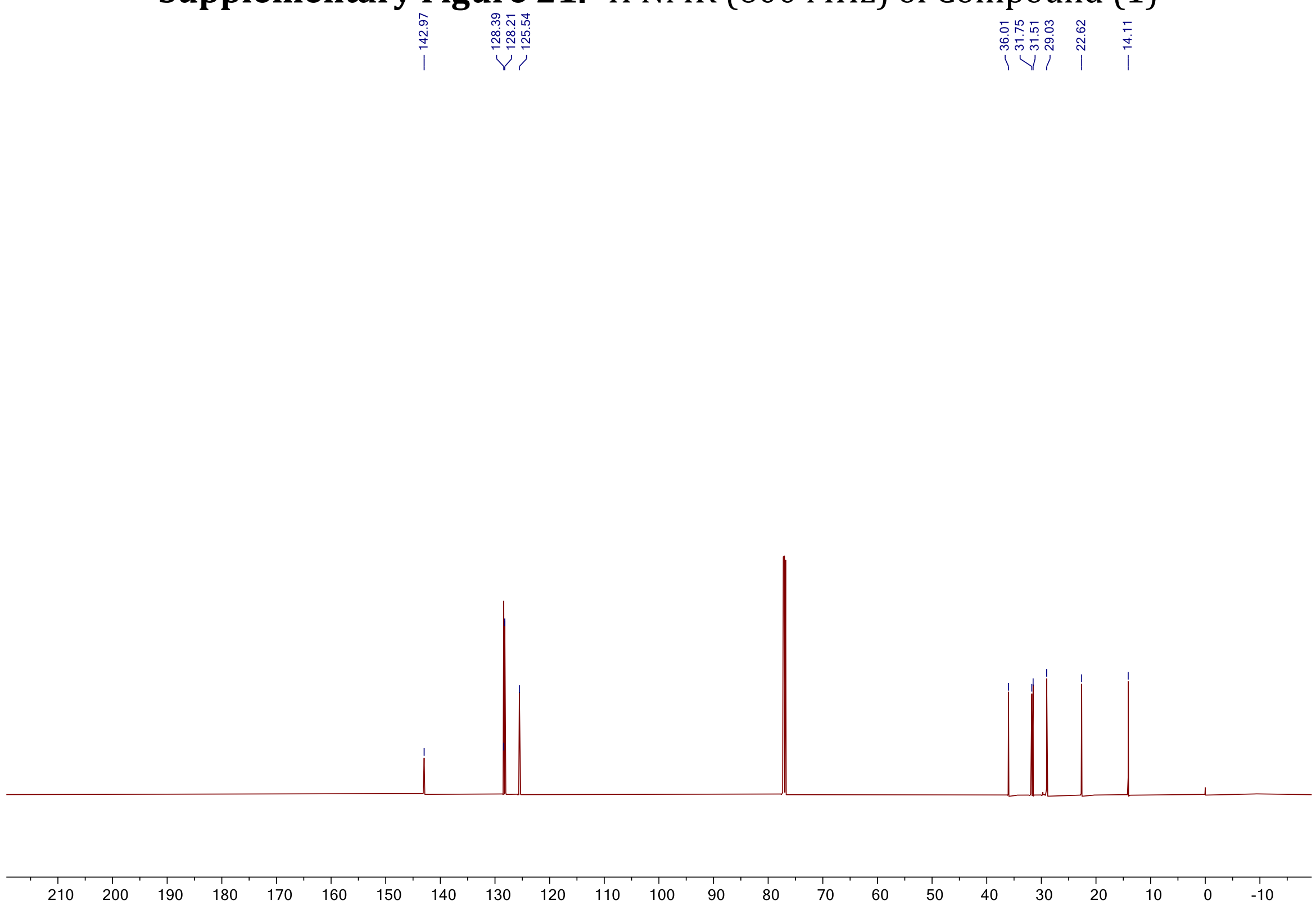

Supplementary Figure 22. ^{13}C NMR (151 MHz) of Compound (1)

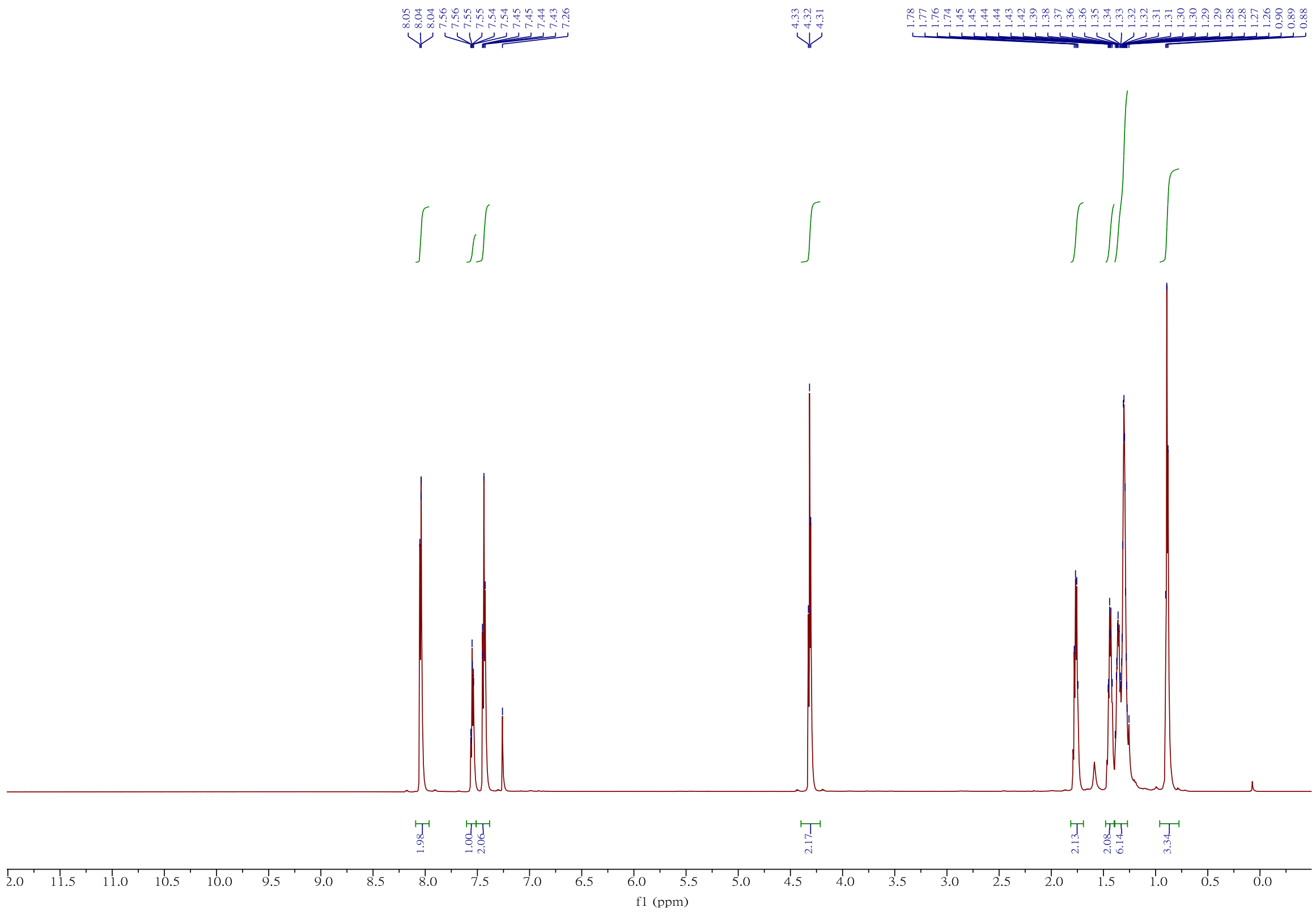

Supplementary Figure 23. ^{1}H NMR (600 MHz) of Compound (2)

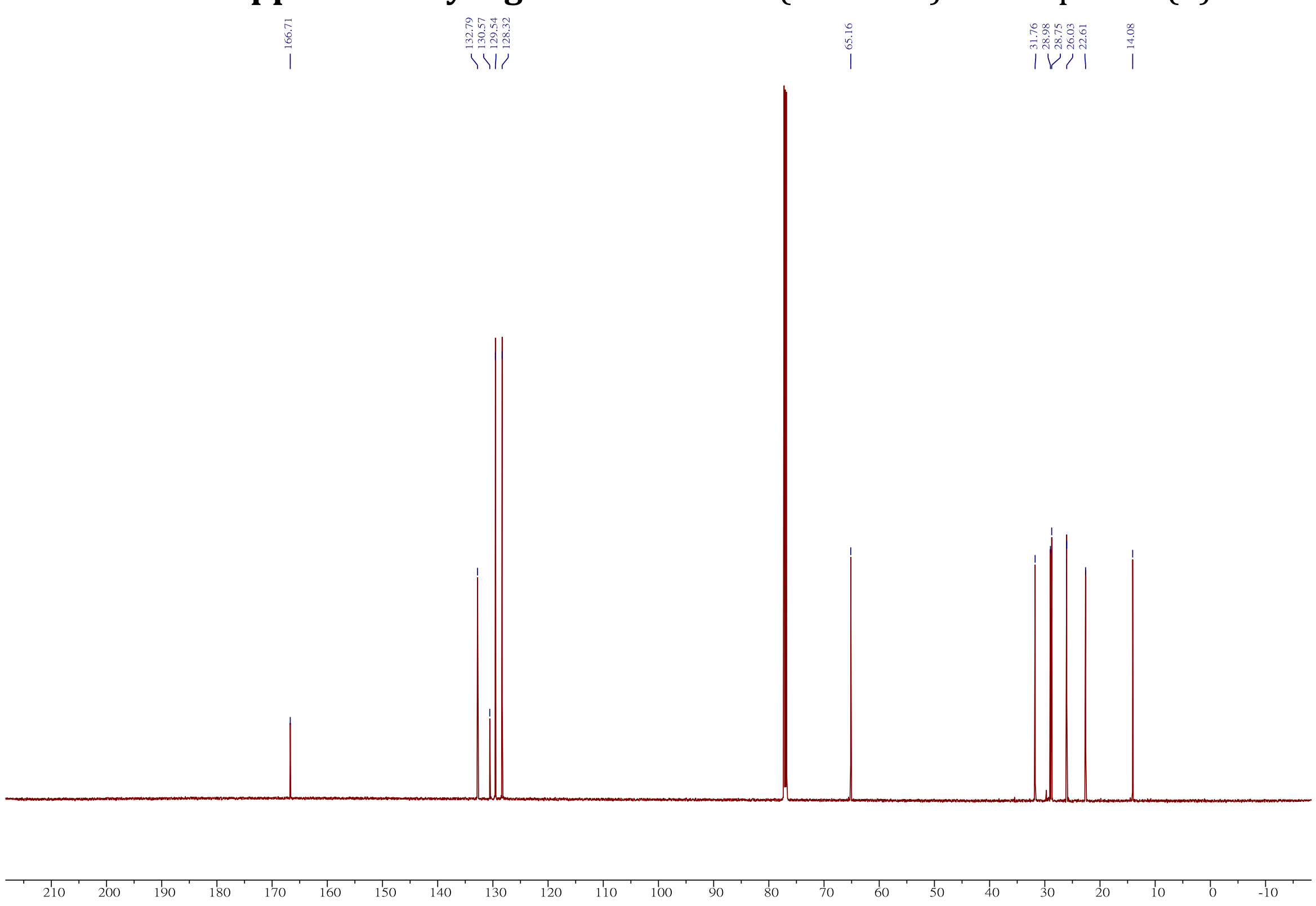

Supplementary Figure 24. ^{13}C NMR (151 MHz) of Compound (2)

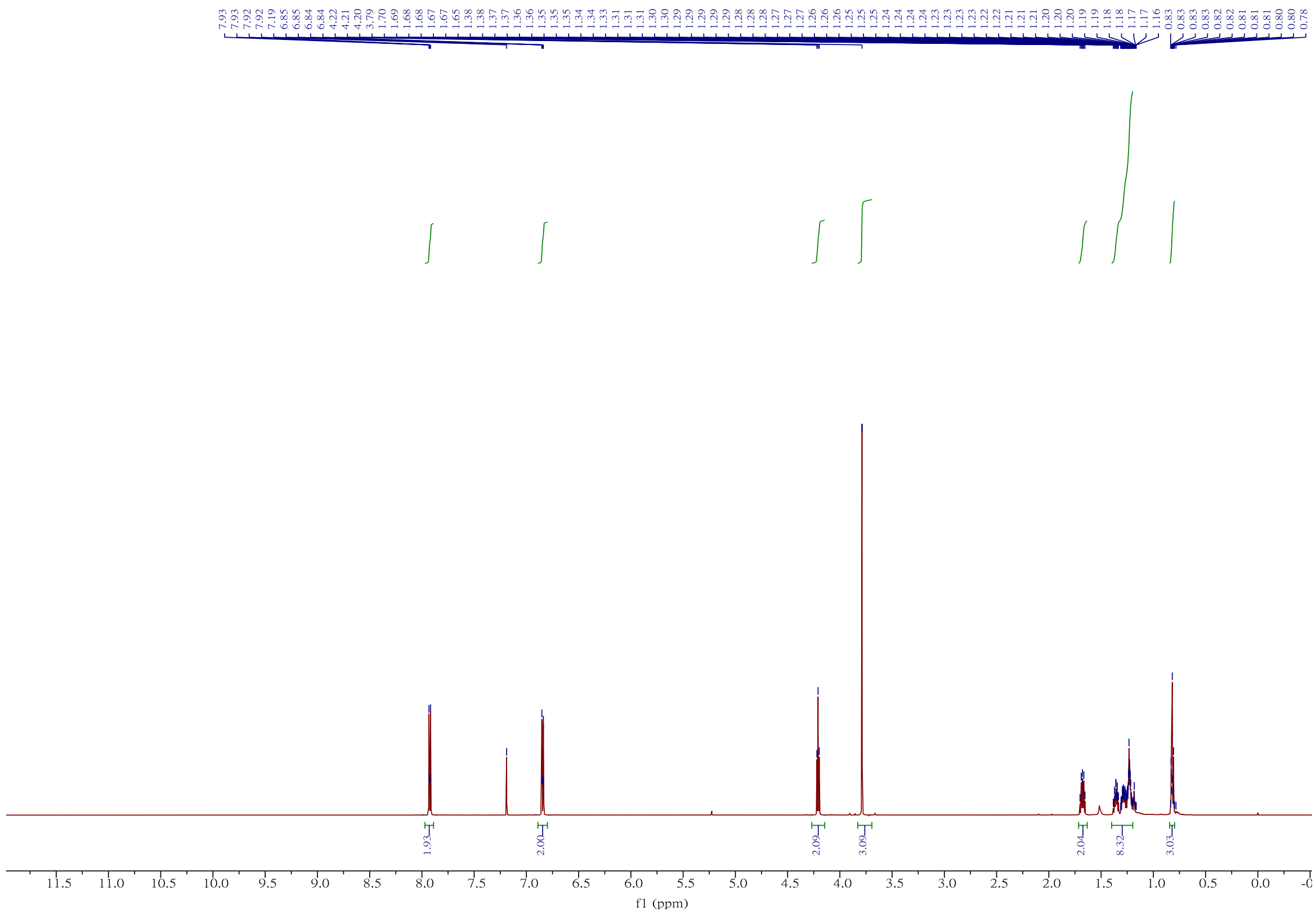

Supplementary Figure 25. ^{1}H NMR (600 MHz) of Compound (3)

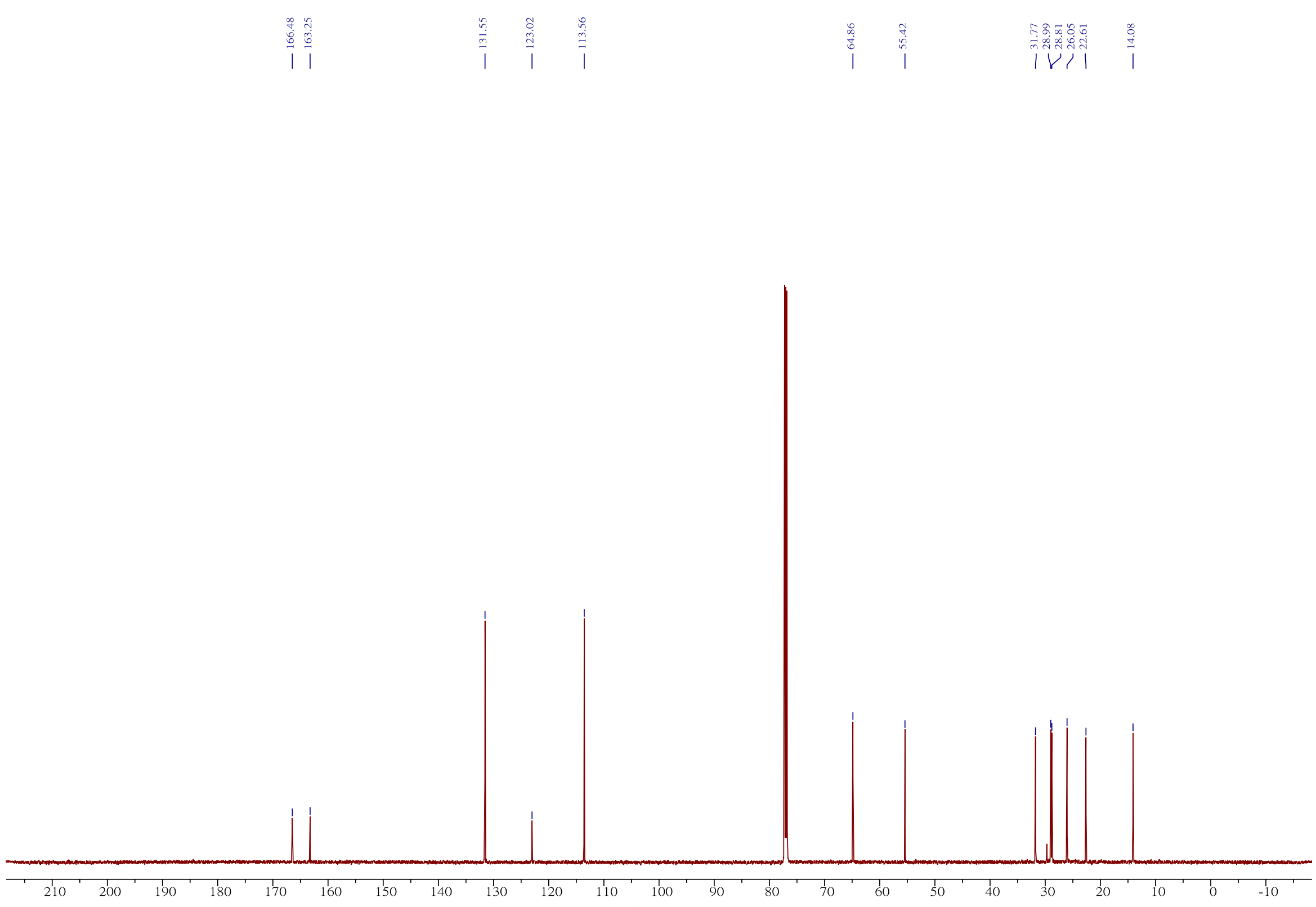

Supplementary Figure 26. ^{13}C NMR (151 MHz) of Compound (3)

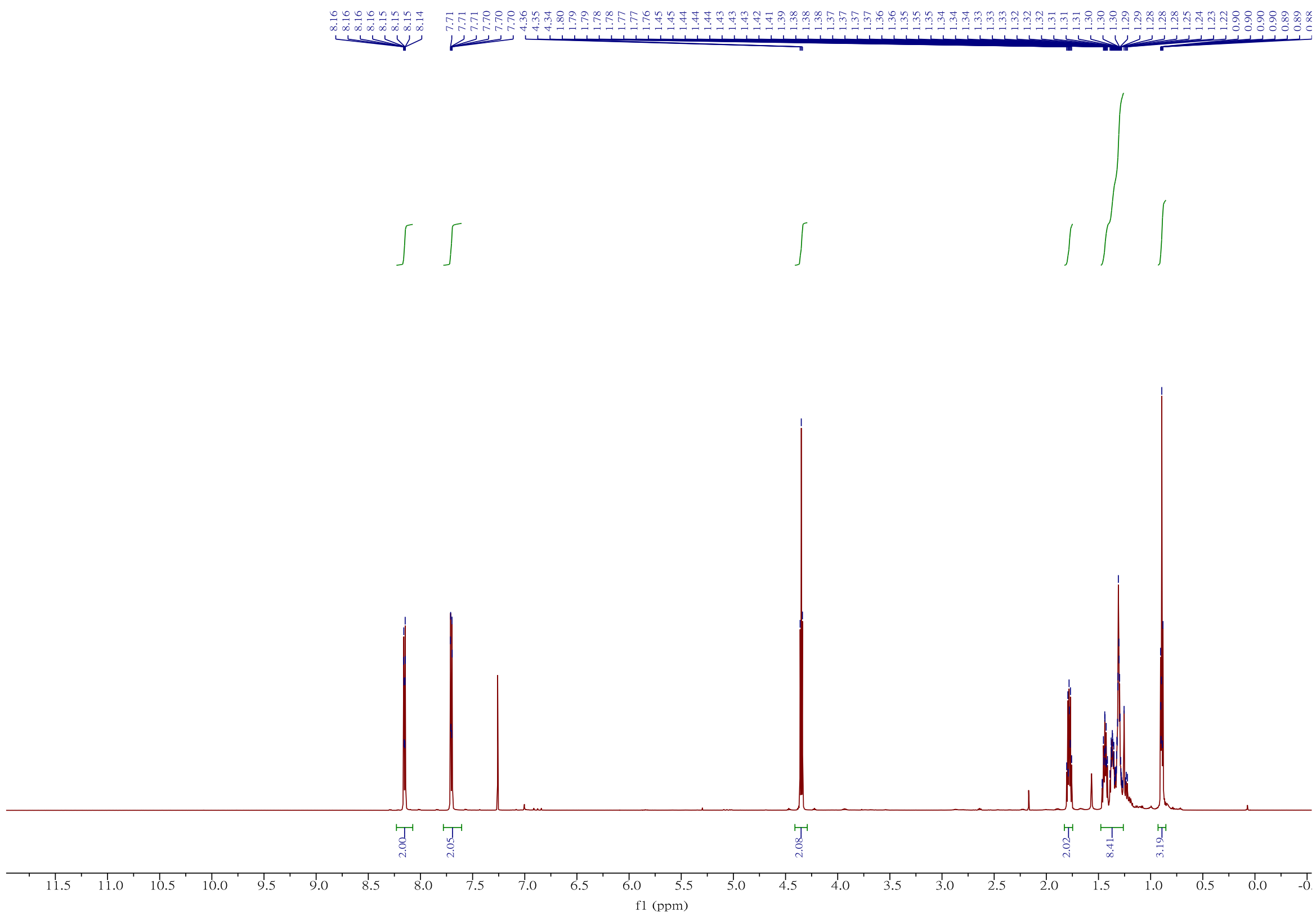

Supplementary Figure 27. ^{1}H NMR (600 MHz) of Compound (4)

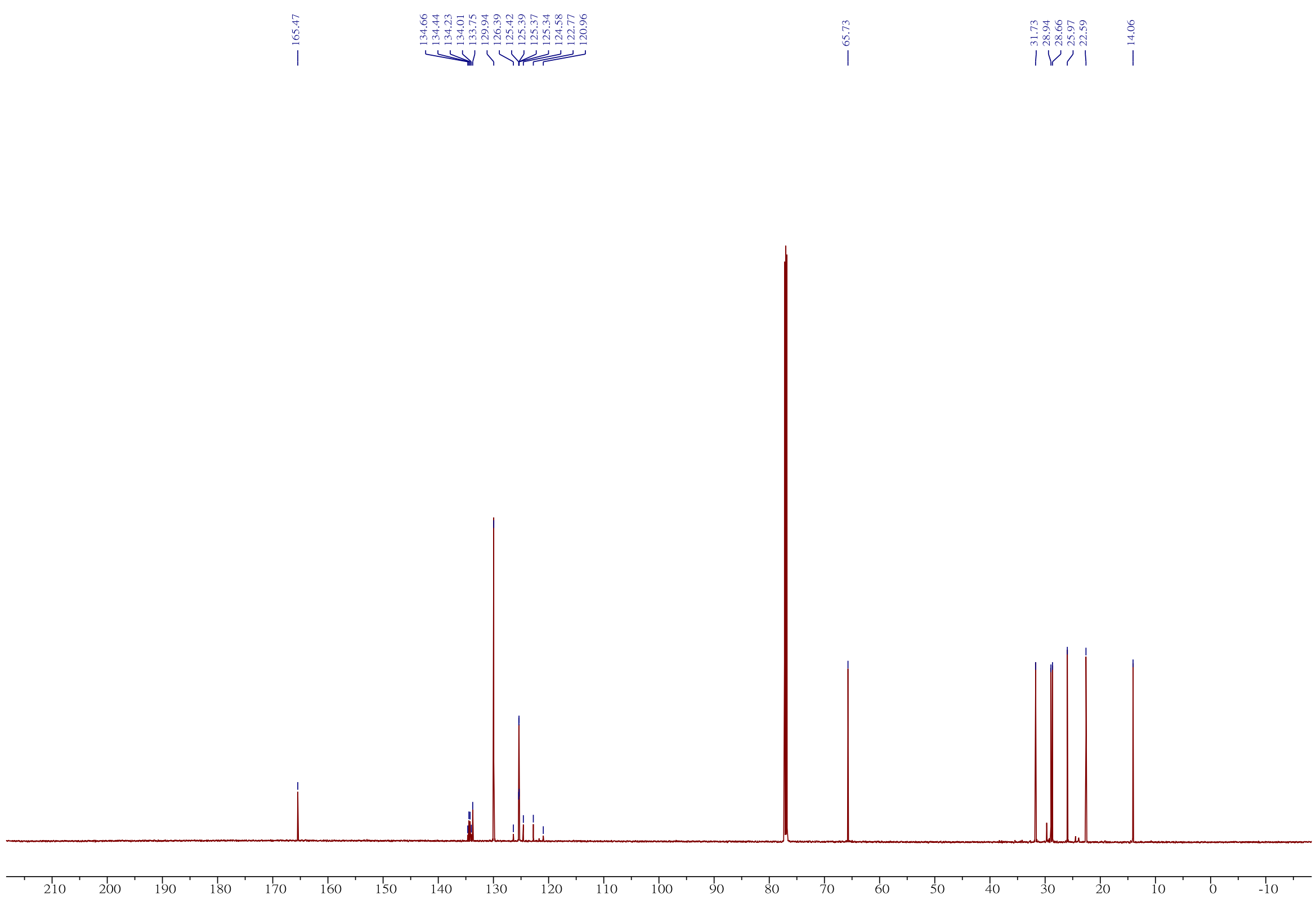

Supplementary Figure 28. ^{13}C NMR (151 MHz) of Compound (4)

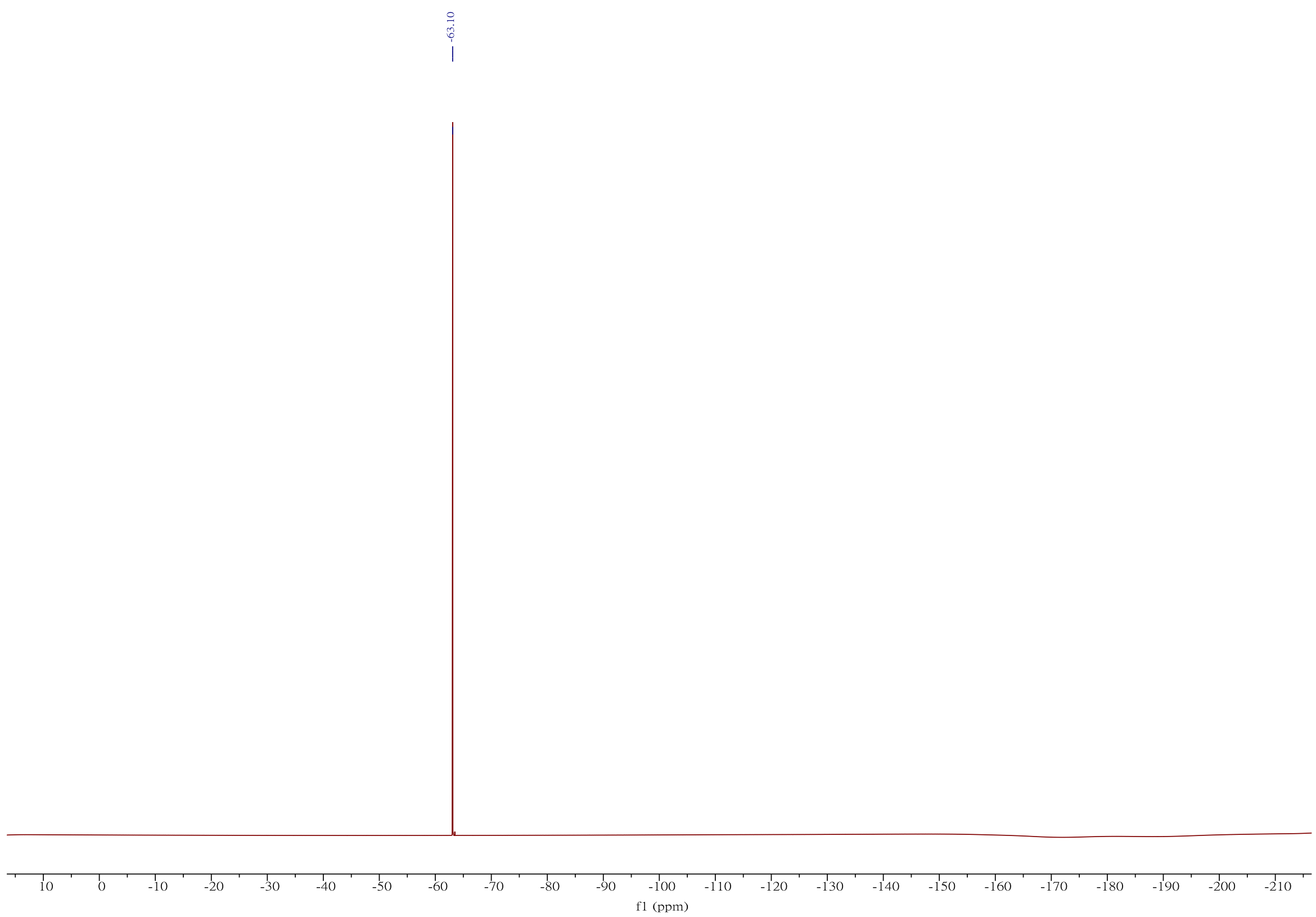

Supplementary Figure 29. ^{19}F NMR (564 MHz) of Compound (4)

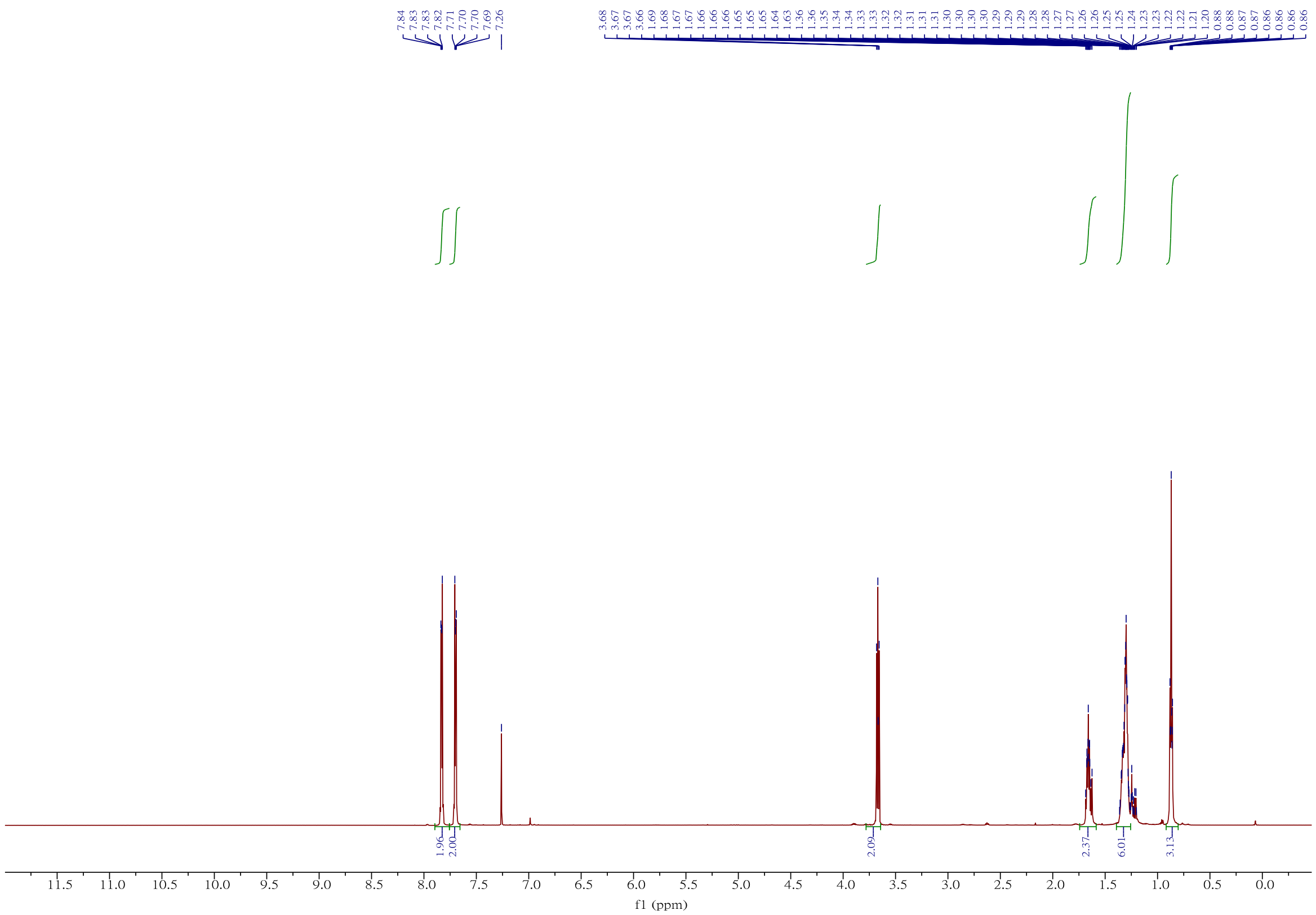

Supplementary Figure 30. ^{1}H NMR (600 MHz) of Compound (5)

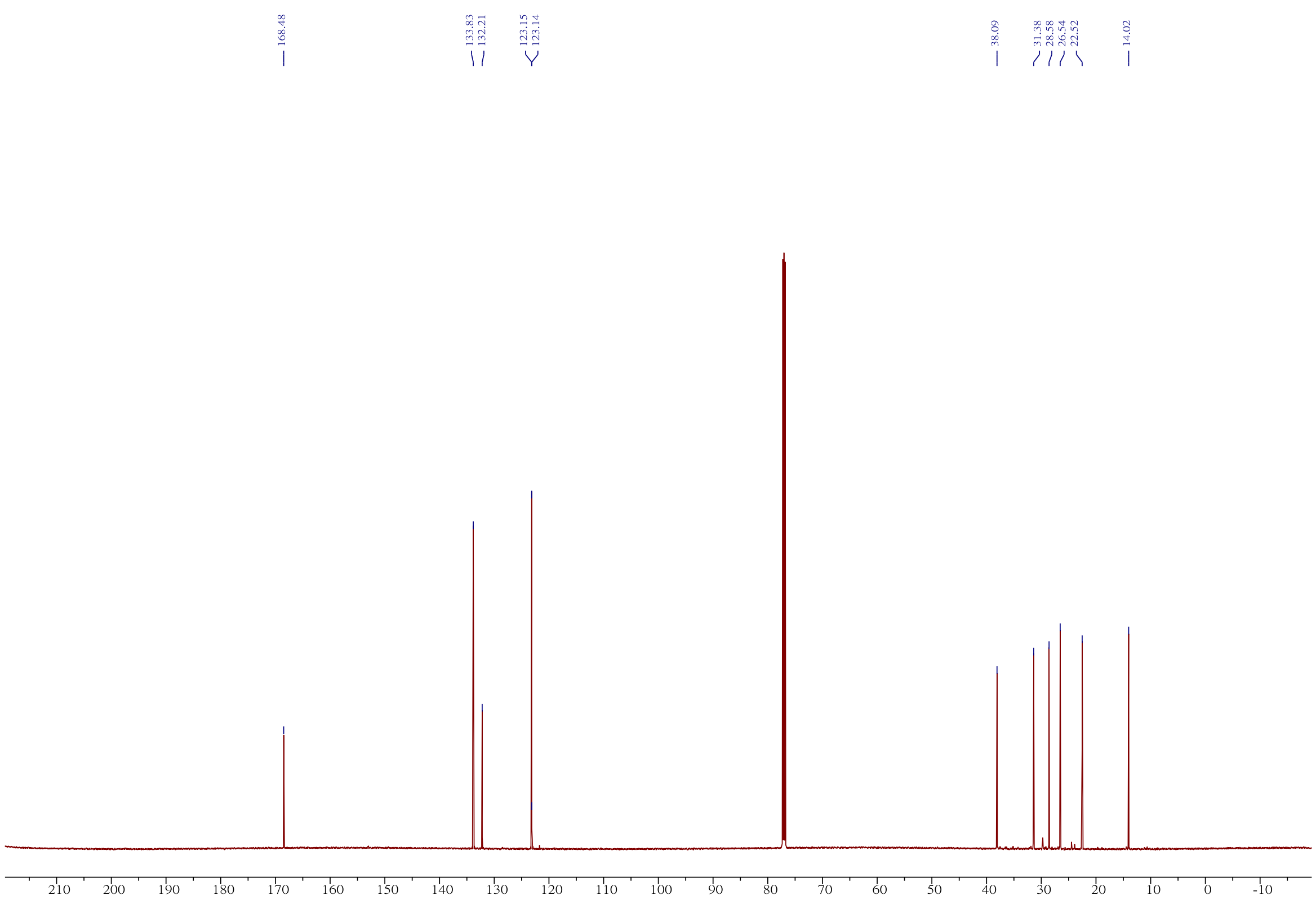

Supplementary Figure 31. ^{13}C NMR (151 MHz) of Compound (5)

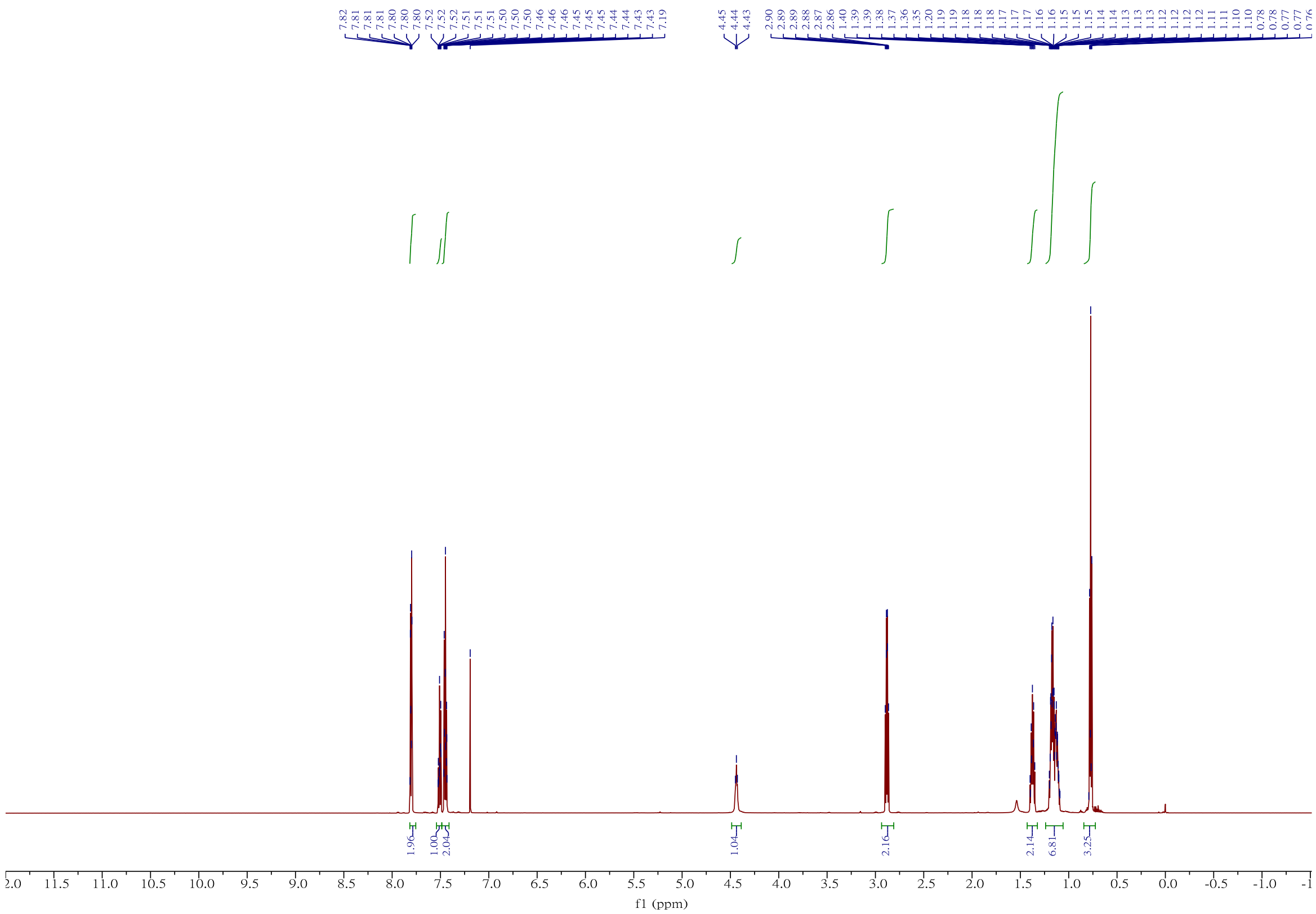

Supplementary Figure 32. ^{1}H NMR (600 MHz) of Compound (6)

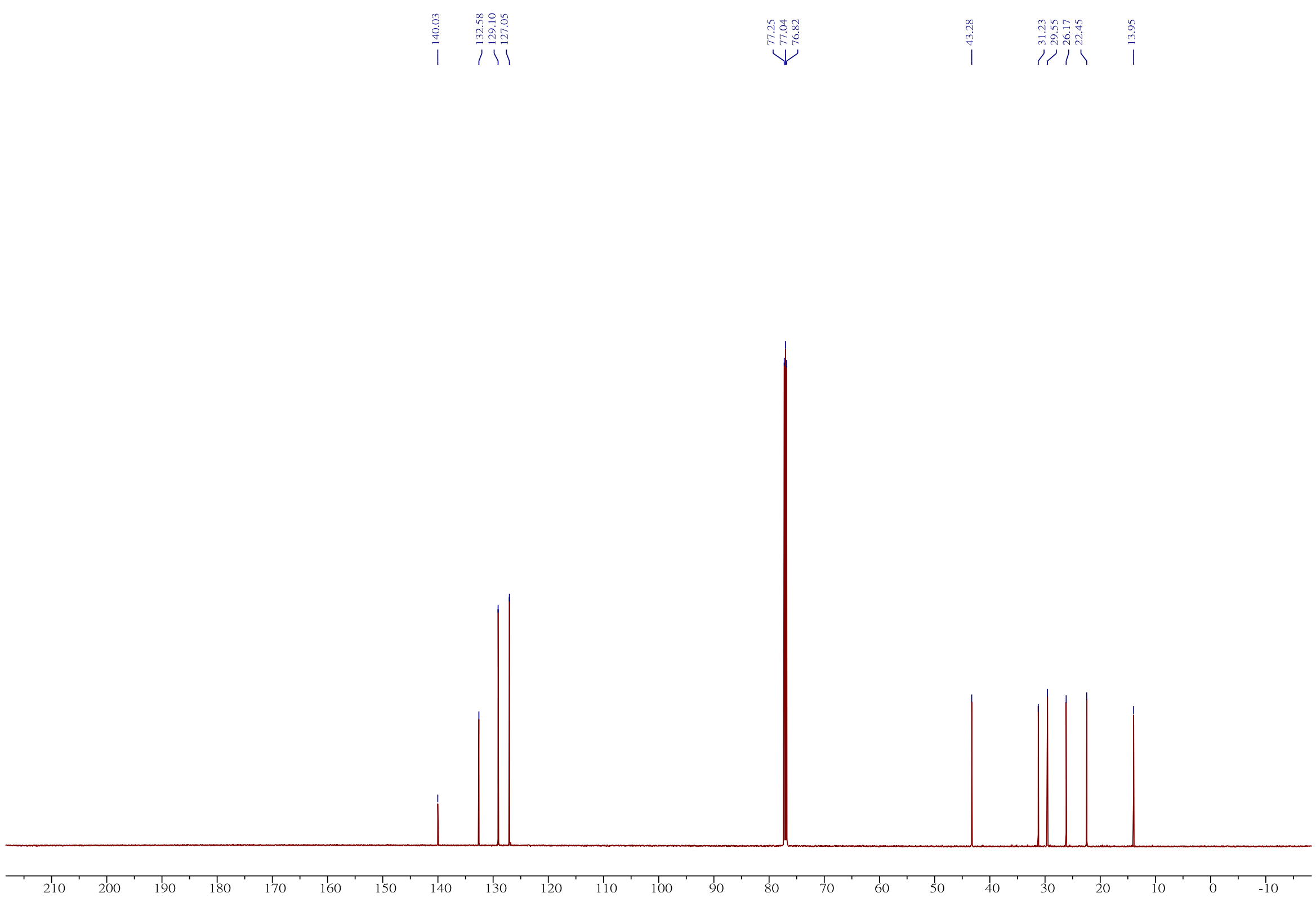

Supplementary Figure 33. ^{13}C NMR (151 MHz) of Compound (6)

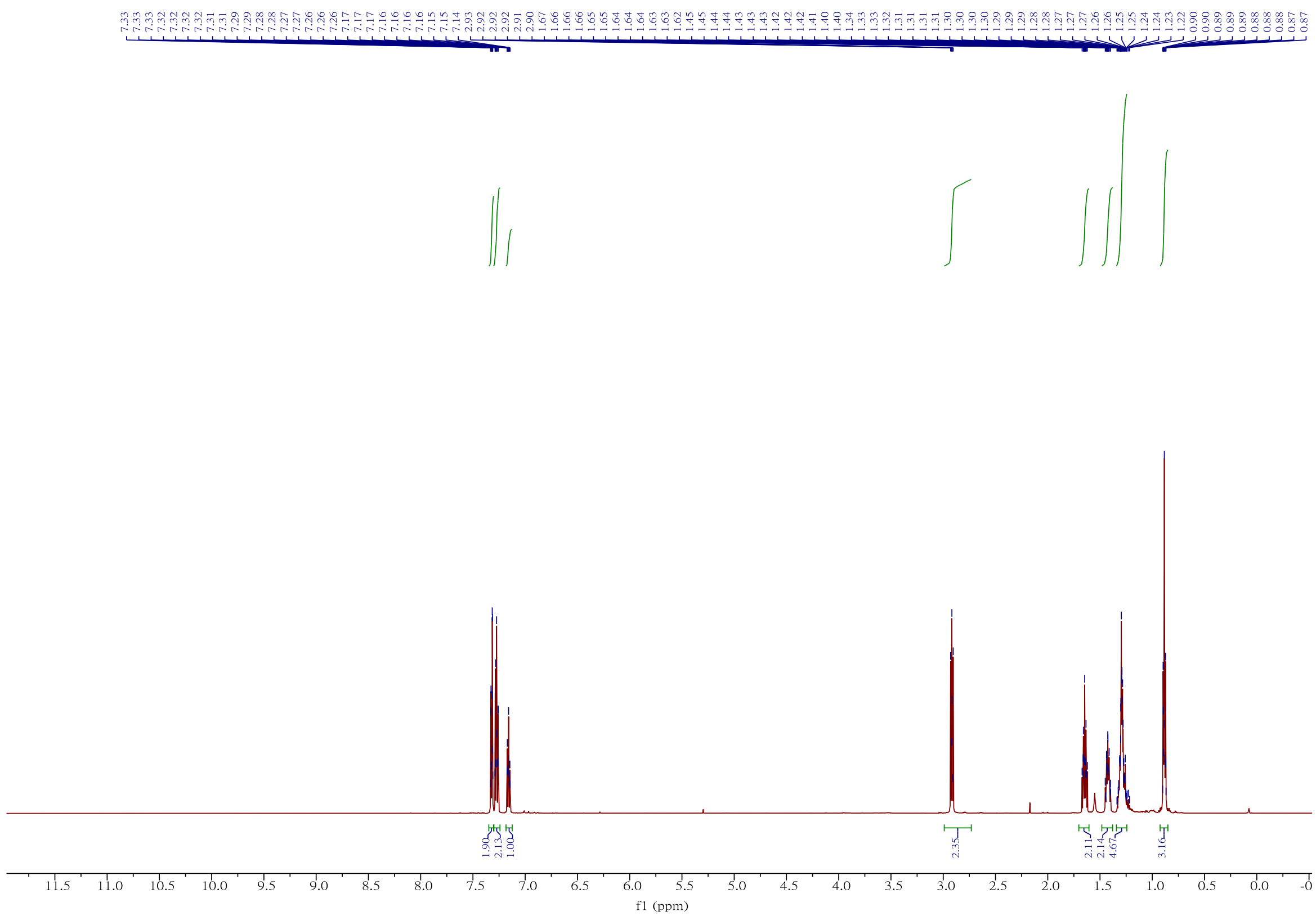

Supplementary Figure 34. ^{1}H NMR (600 MHz) of Compound (7)

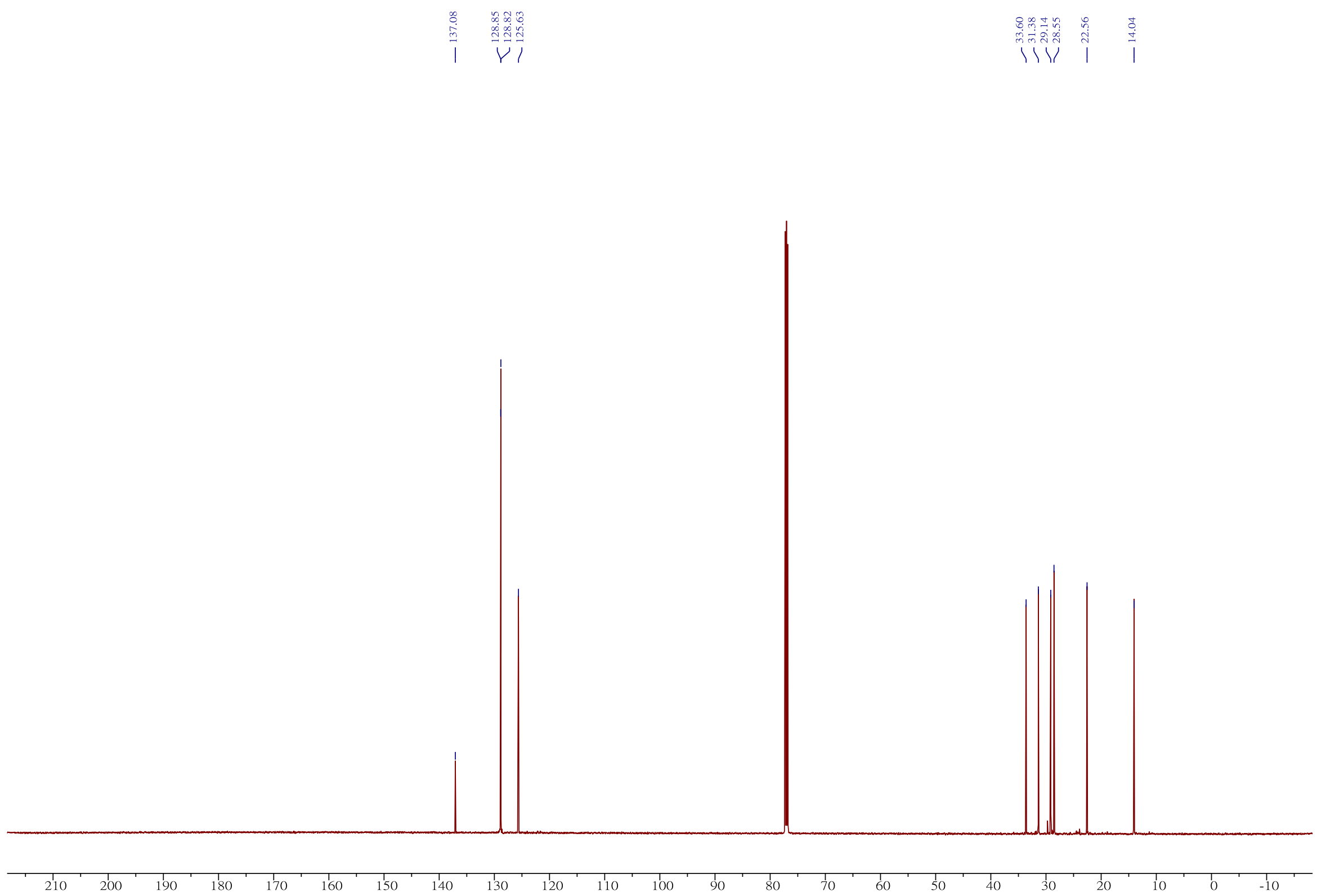

Supplementary Figure 35. ^{13}C NMR (151 MHz) of Compound (7)

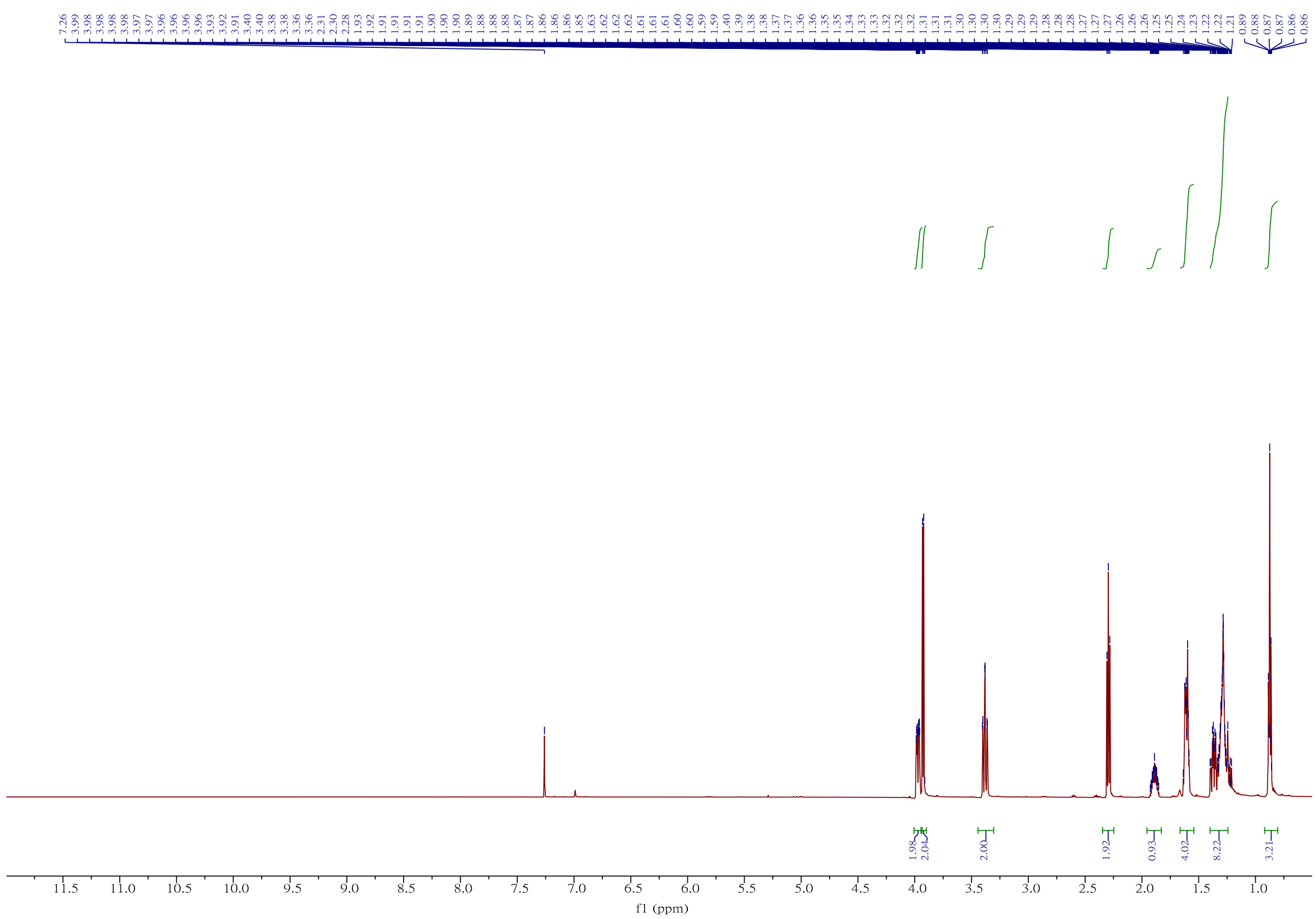

Supplementary Figure 36. ^{1}H NMR (600 MHz) of Compound (8)

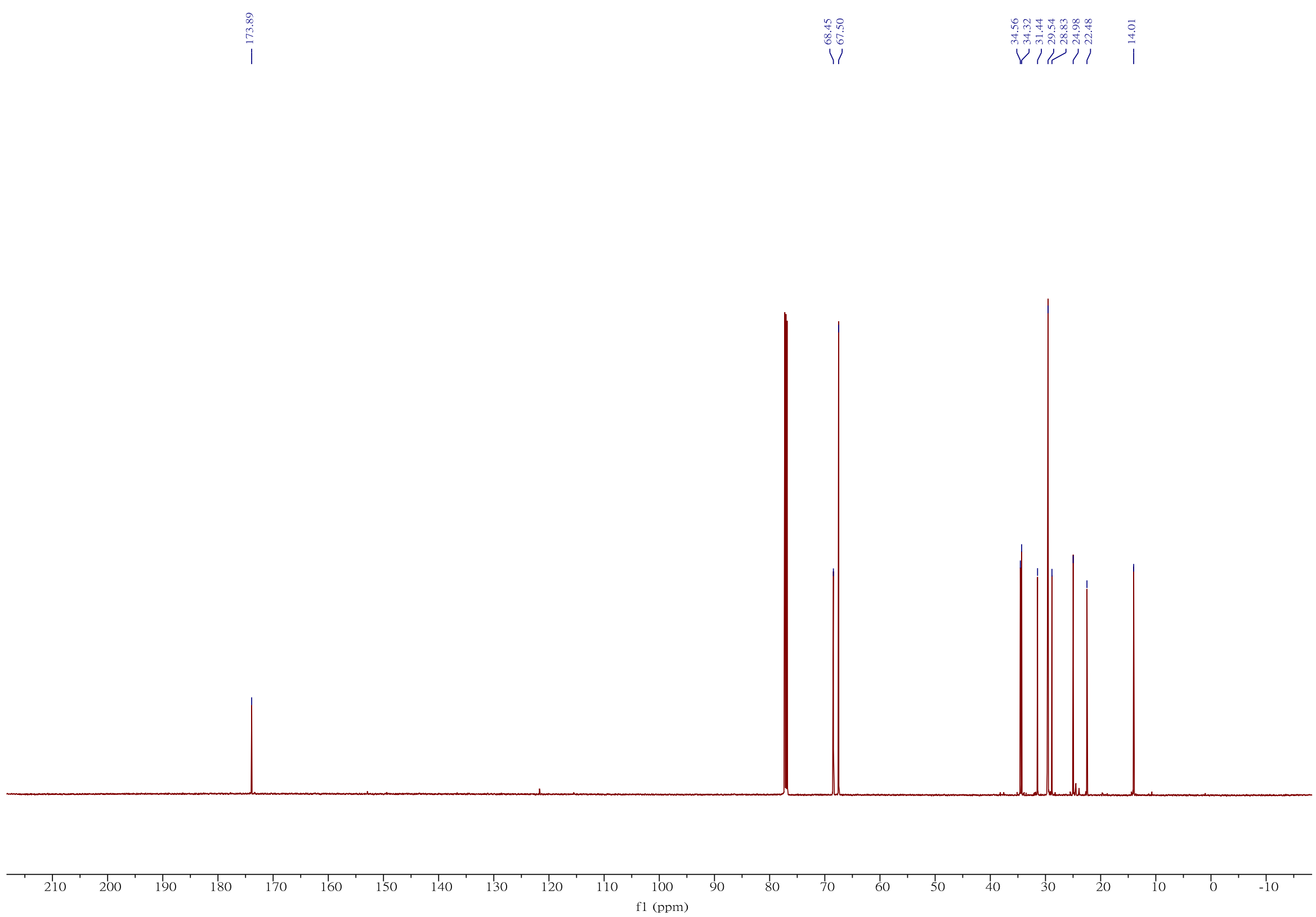

Supplementary Figure 37. ^{13}C NMR (151 MHz) of Compound (8)

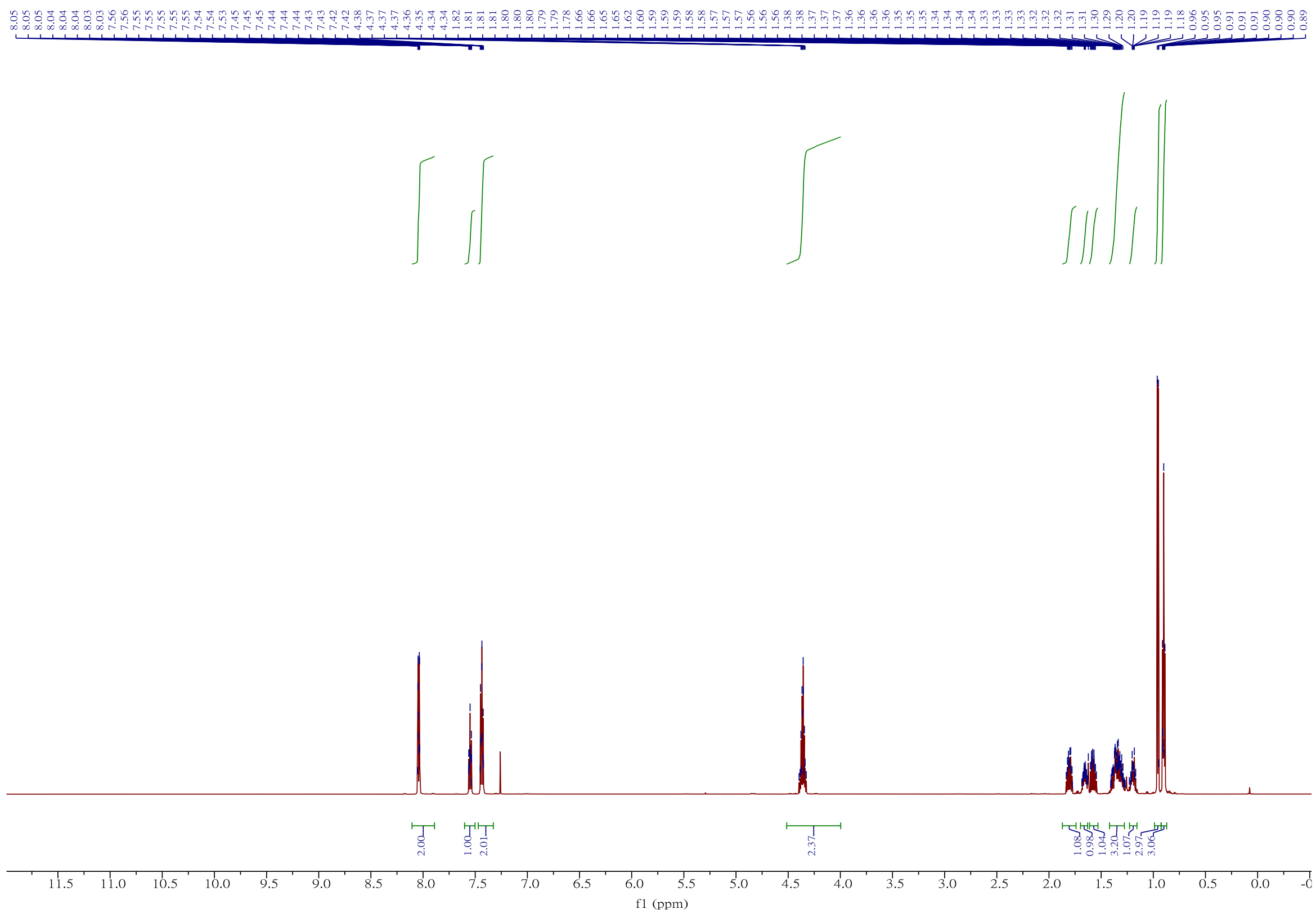

Supplementary Figure 38. ^{1}H NMR (600 MHz) of Compound (9)

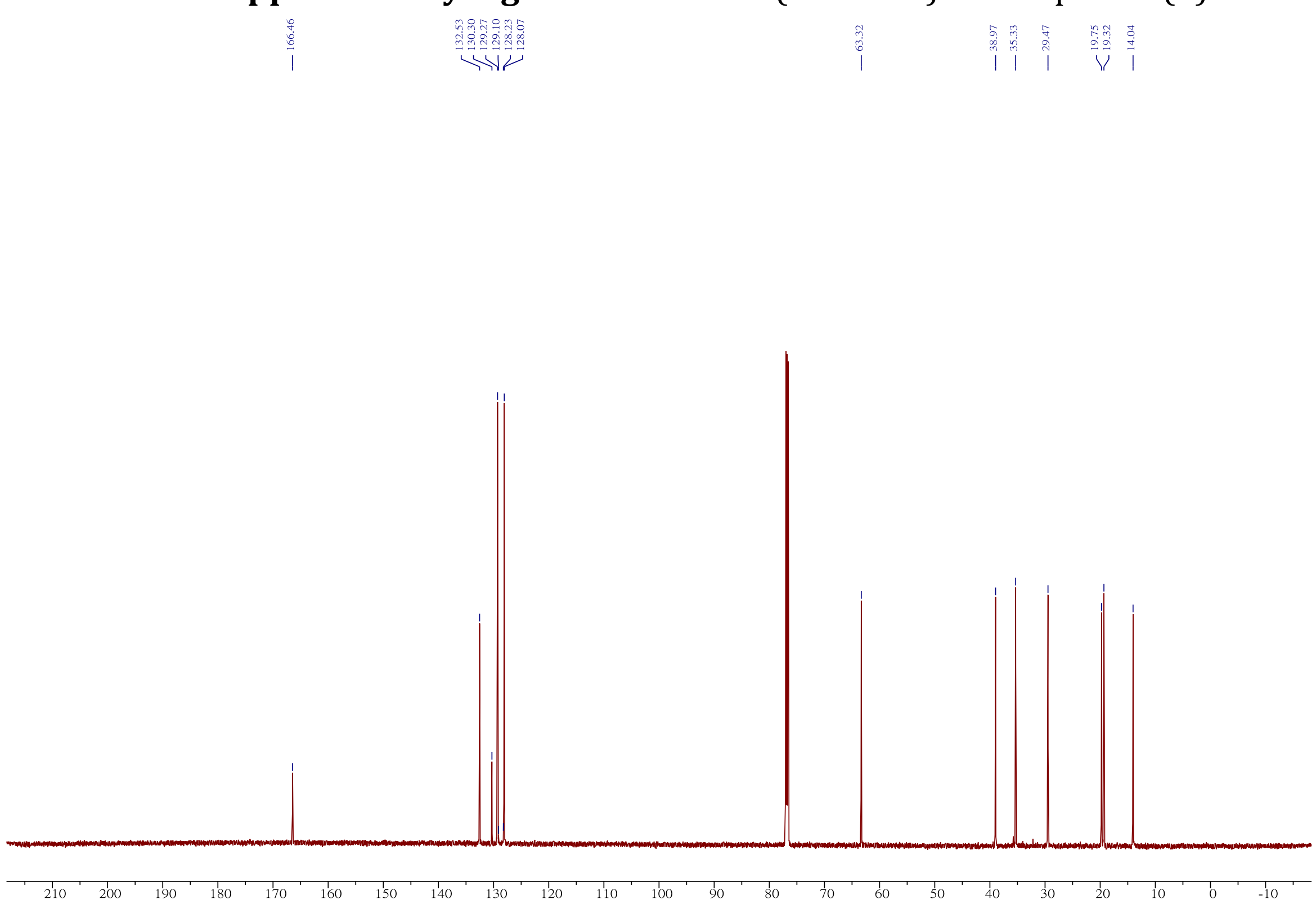

Supplementary Figure 39. ^{13}C NMR (151 MHz) of Compound (9)

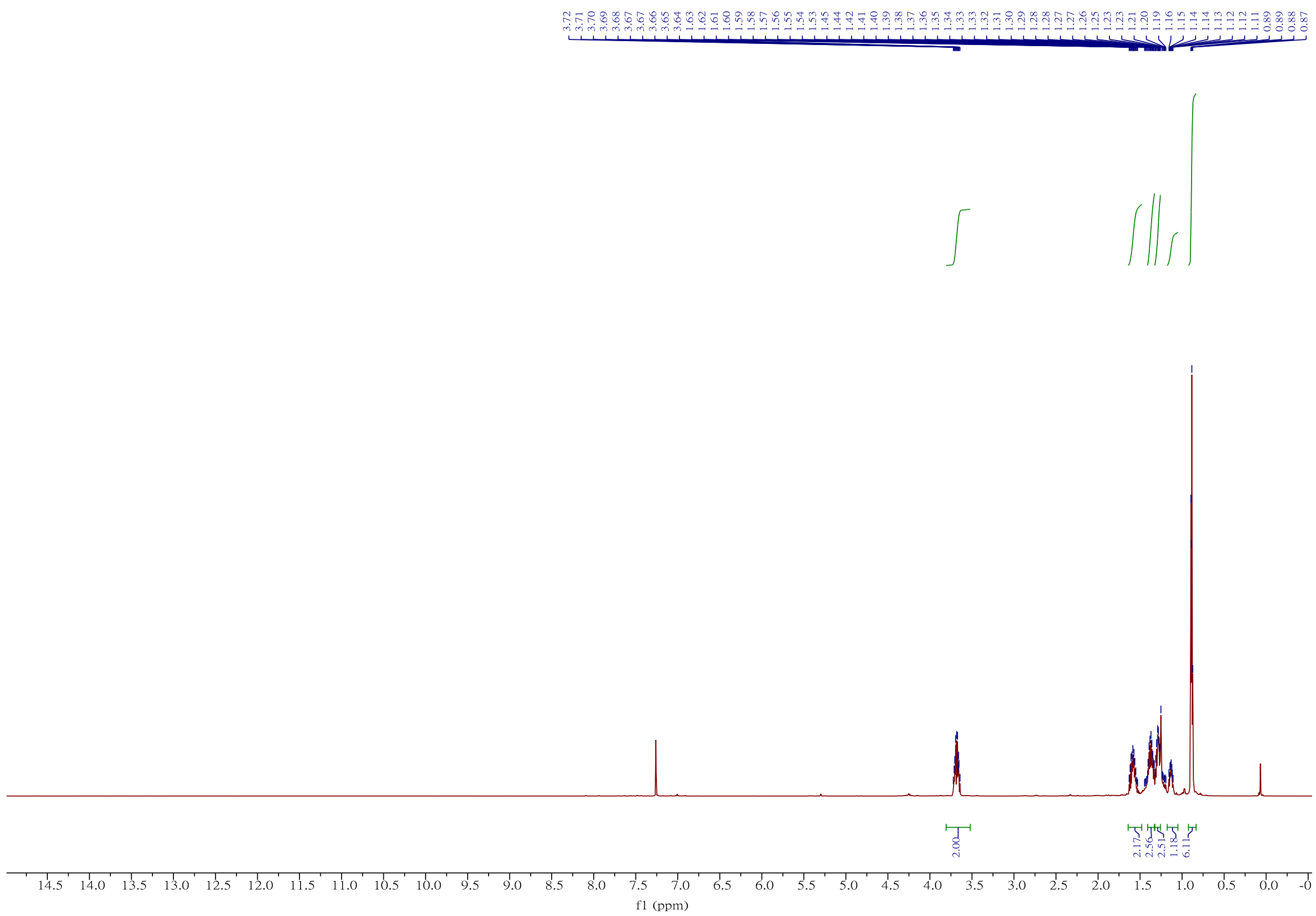

Supplementary Figure 40. ^{1}H NMR (600 MHz) of Compound (10)

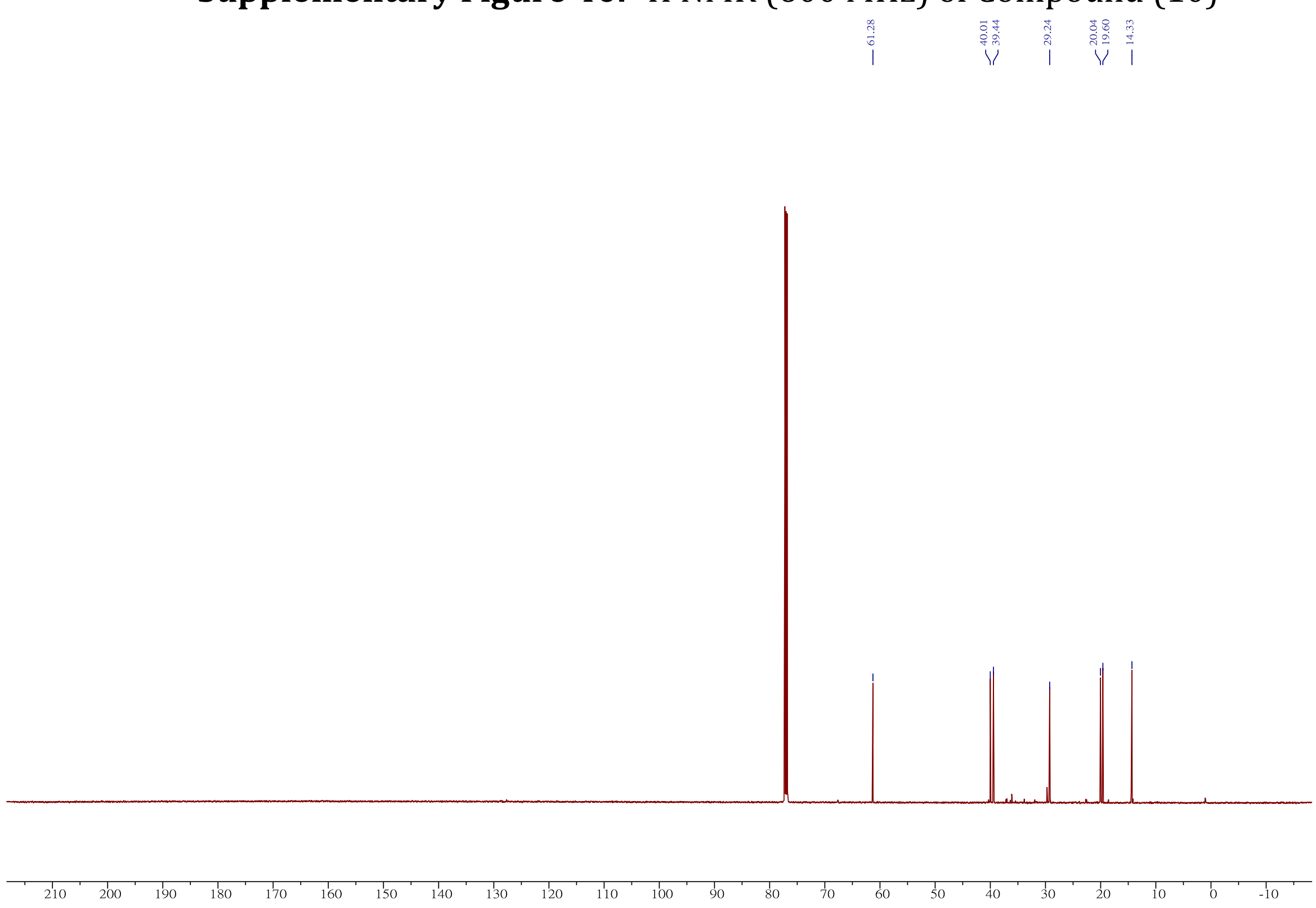

Supplementary Figure 41. ^{13}C NMR (151 MHz) of Compound (10)

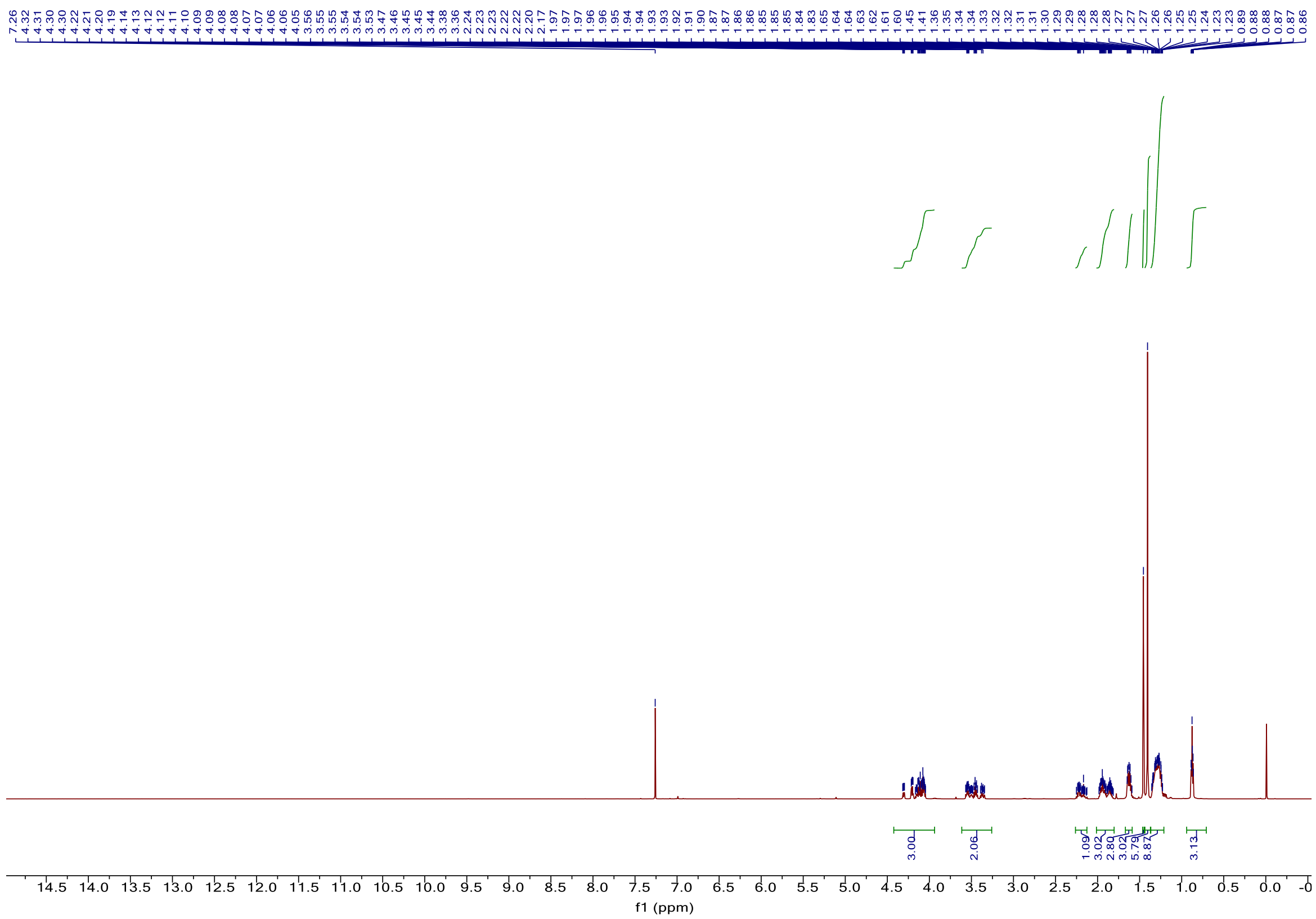

Supplementary Figure 42. ^{1}H NMR (600 MHz) of Compound (11)

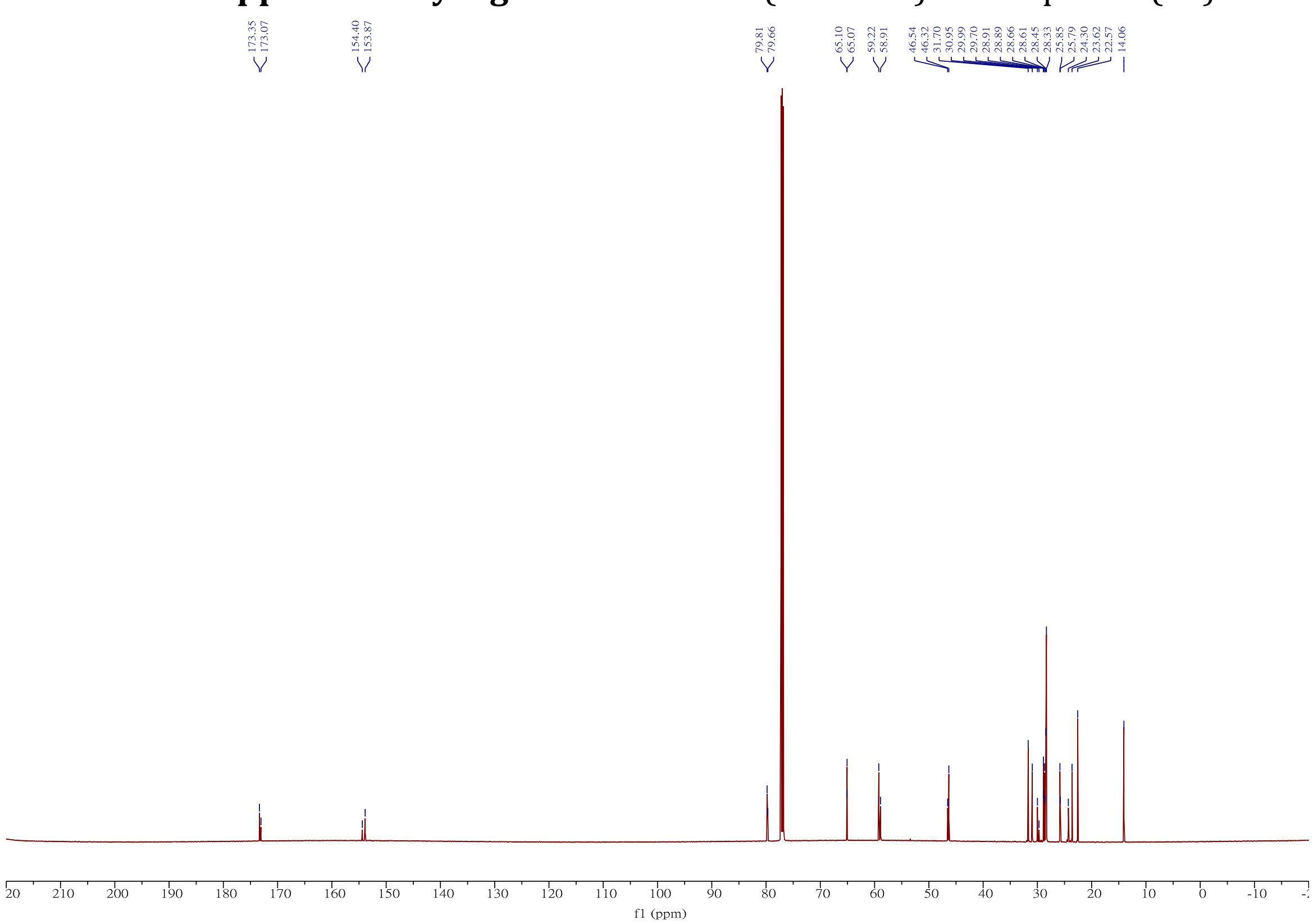

Supplementary Figure 43. ^{13}C NMR (151 MHz) of Compound (11)

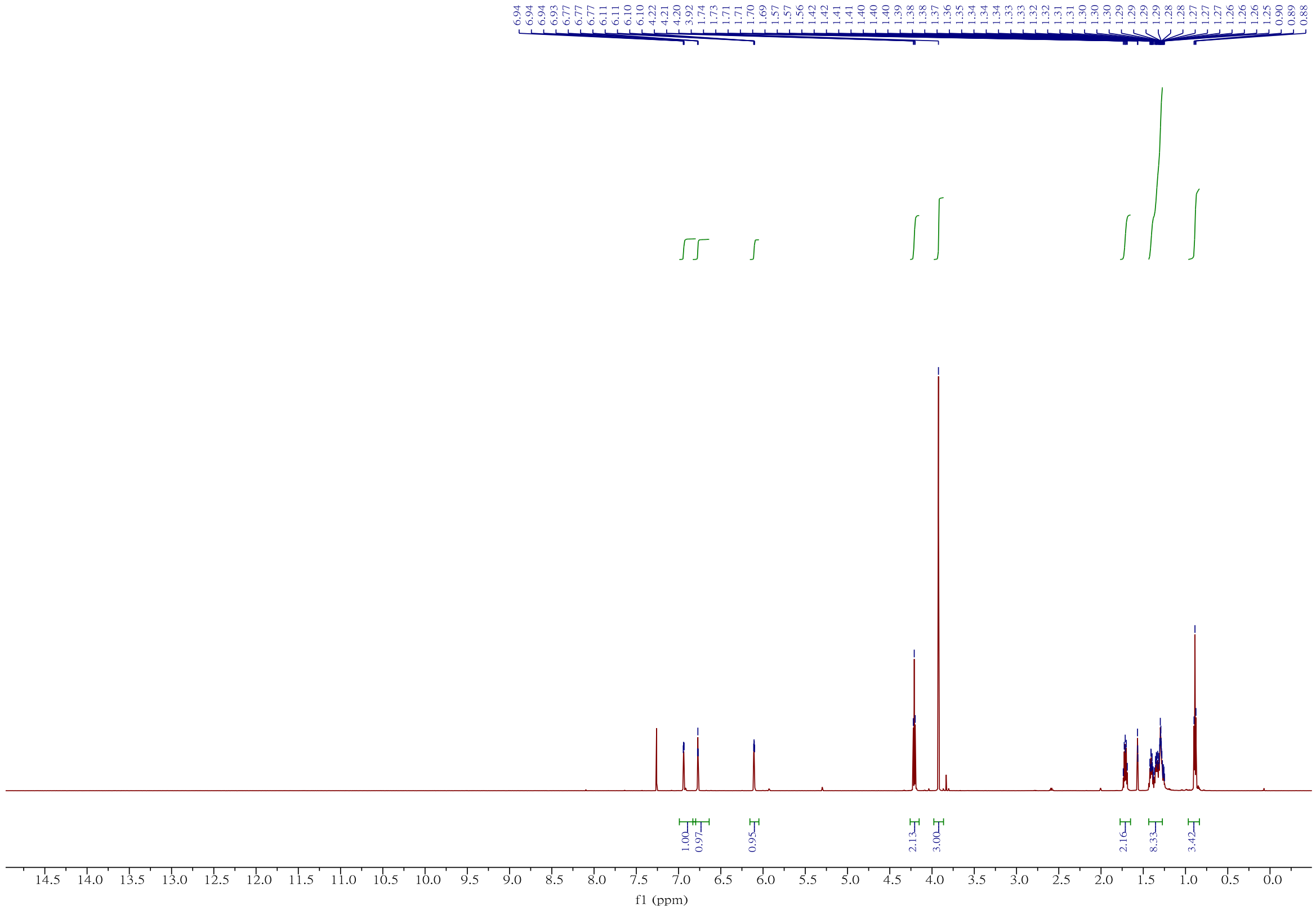

Supplementary Figure 44. ^{1}H NMR (600 MHz) of Compound (12)

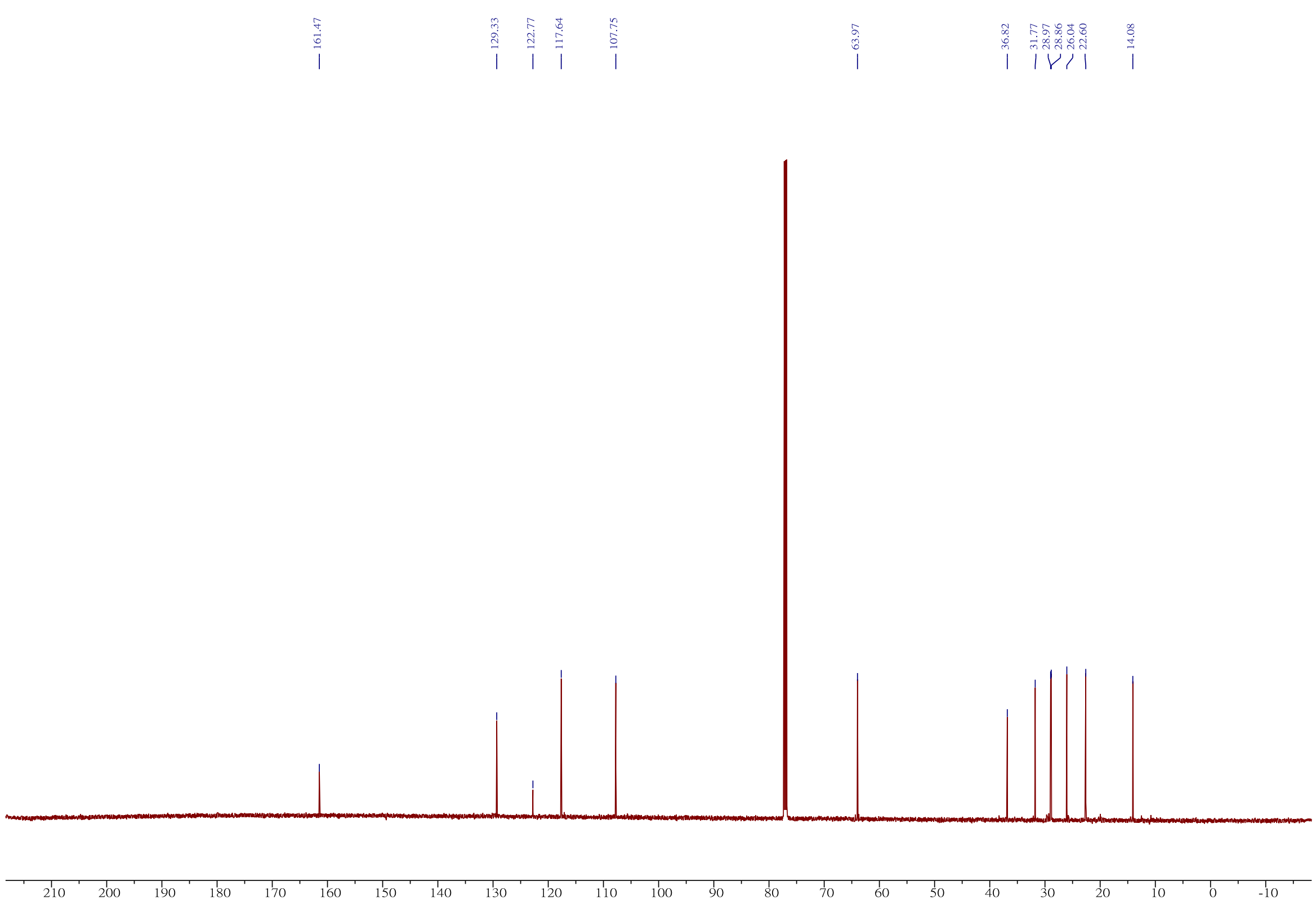

Supplementary Figure 45. ^{13}C NMR (151 MHz) of Compound (12)

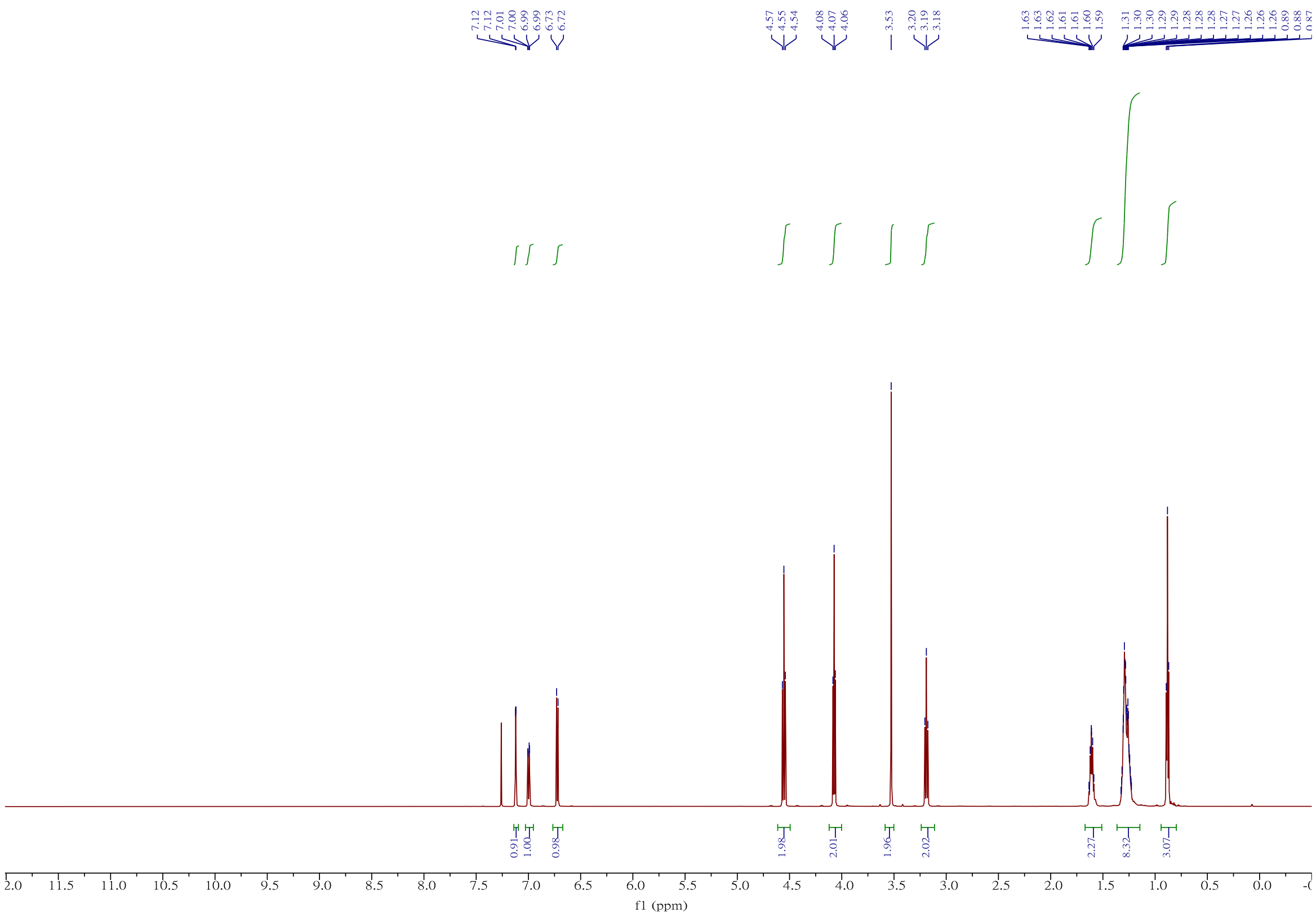

Supplementary Figure 46. ^{1}H NMR (600 MHz) of Compound (13)

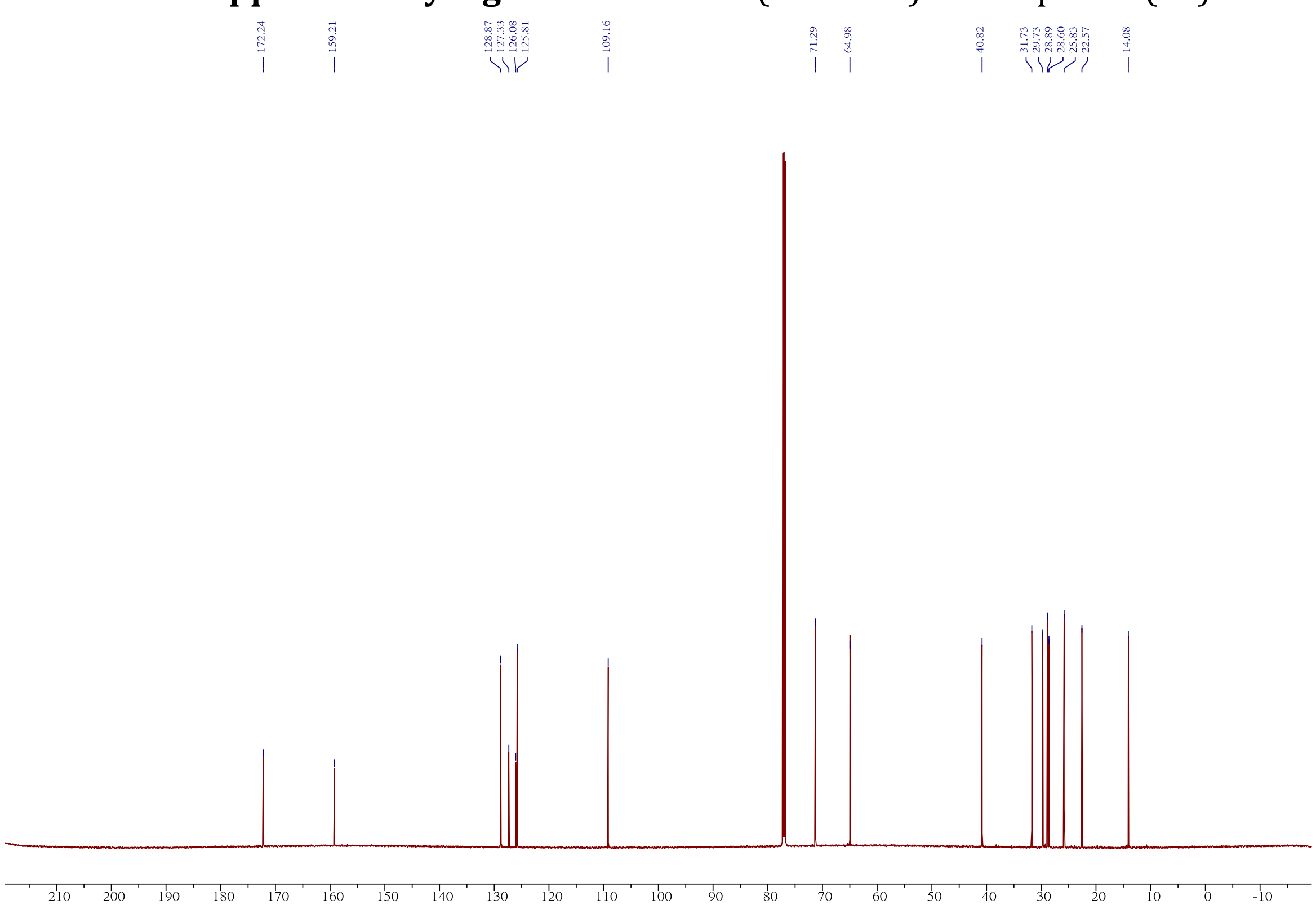

Supplementary Figure 47. ^{13}C NMR (151 MHz) of Compound (13)

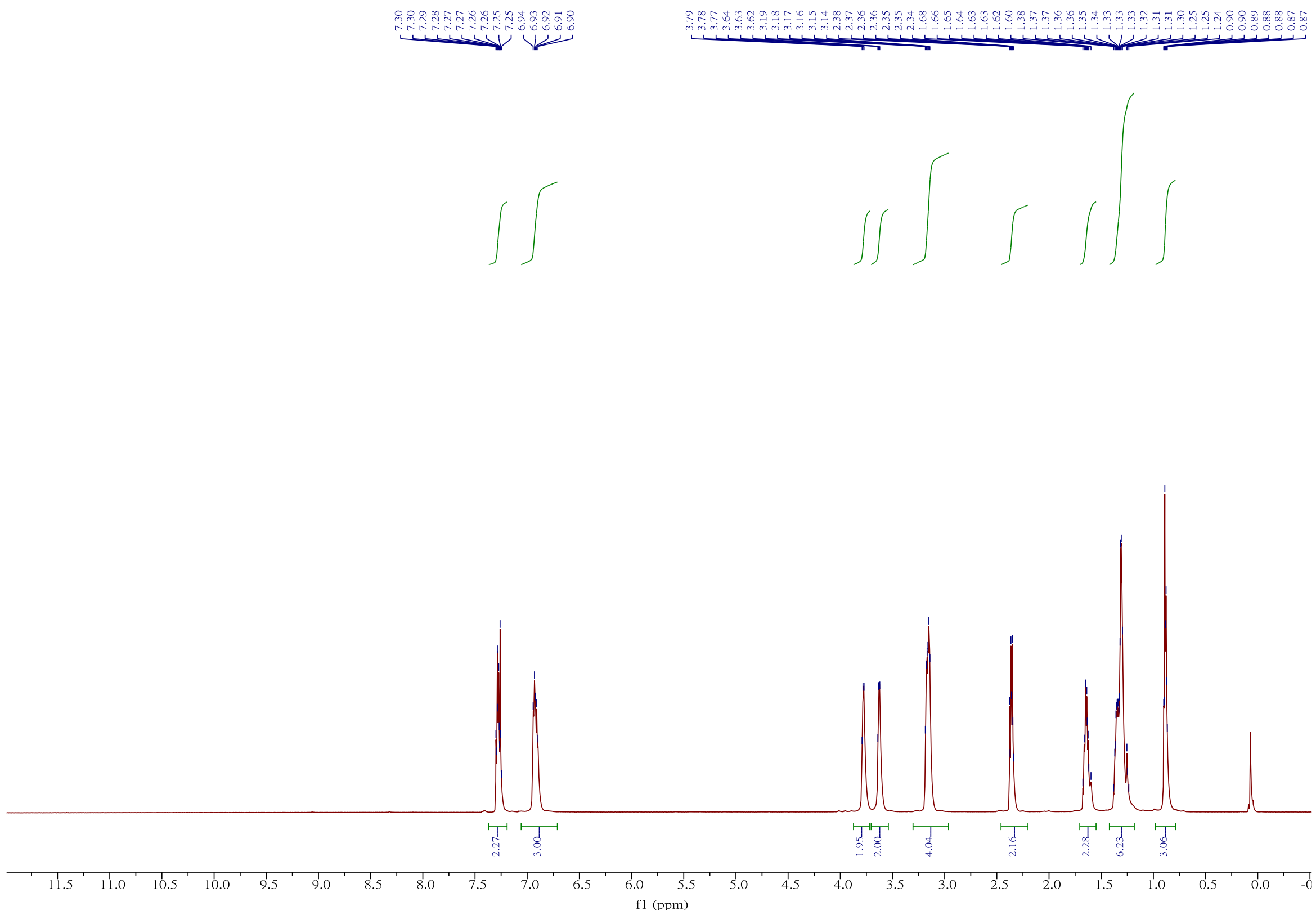

Supplementary Figure 48. ^{1}H NMR (600 MHz) of Compound (14)

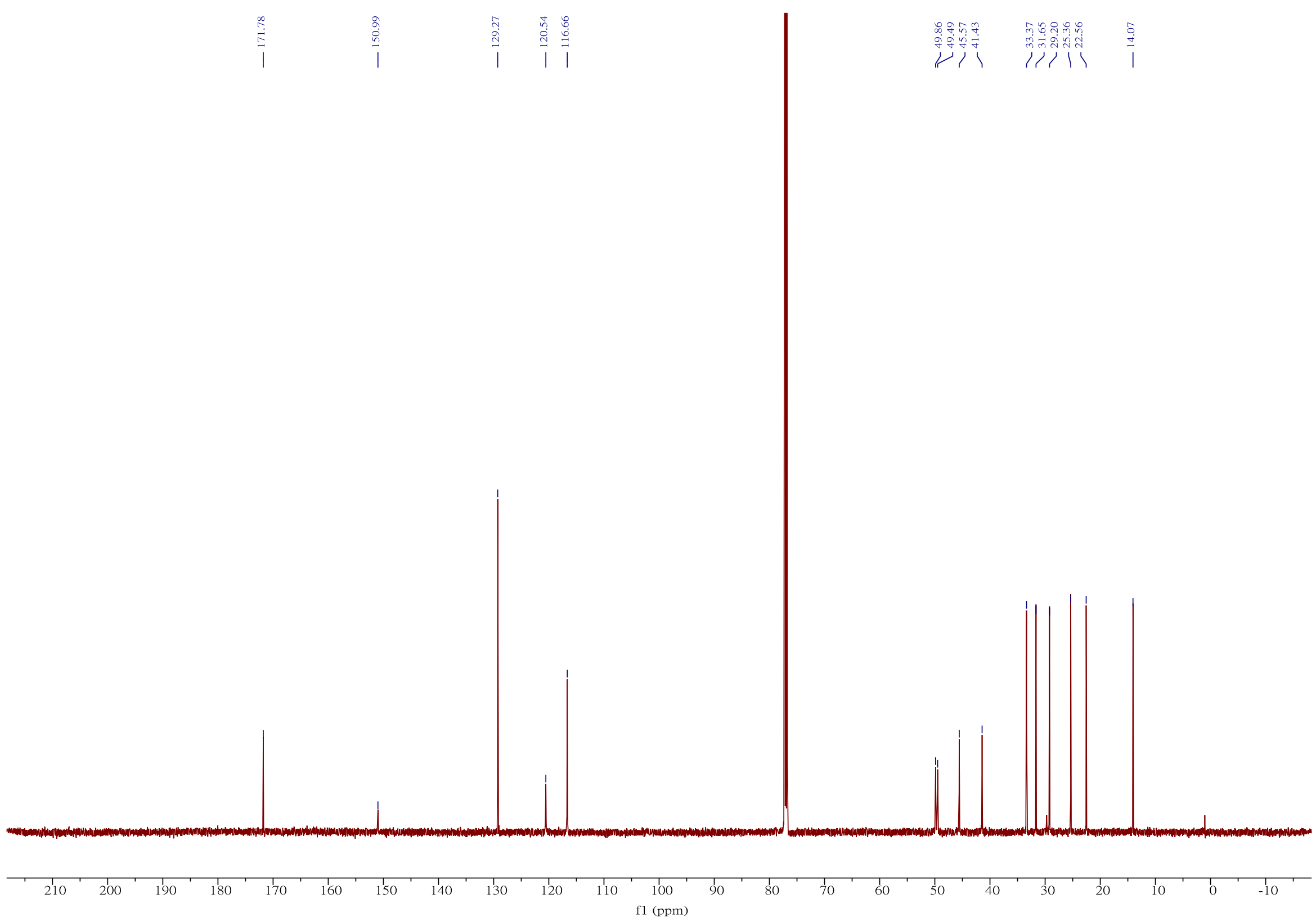

Supplementary Figure 49. ^{13}C NMR (151 MHz) of Compound (14)

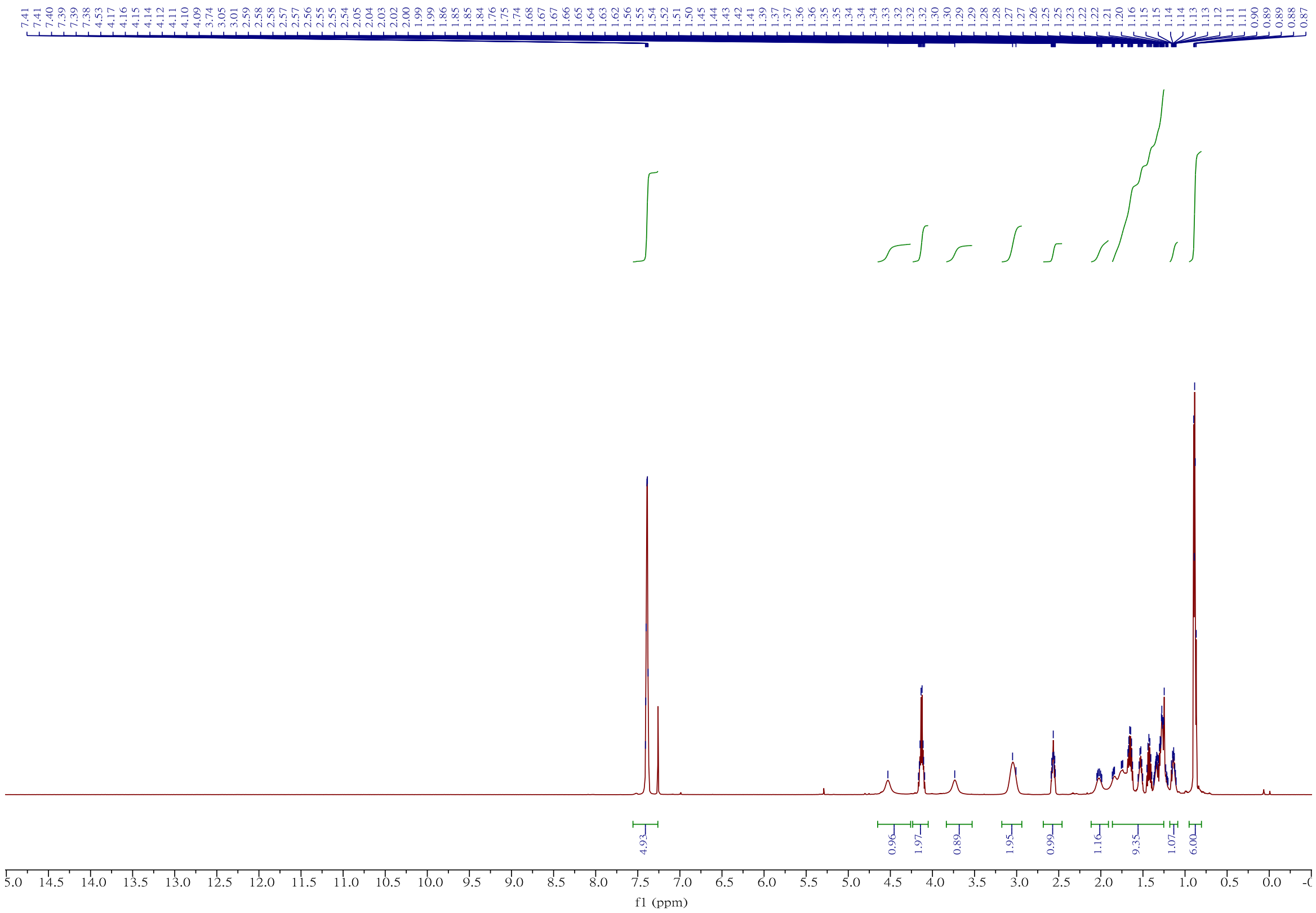

Supplementary Figure 50. ^{1}H NMR (600 MHz) of Compound (15)

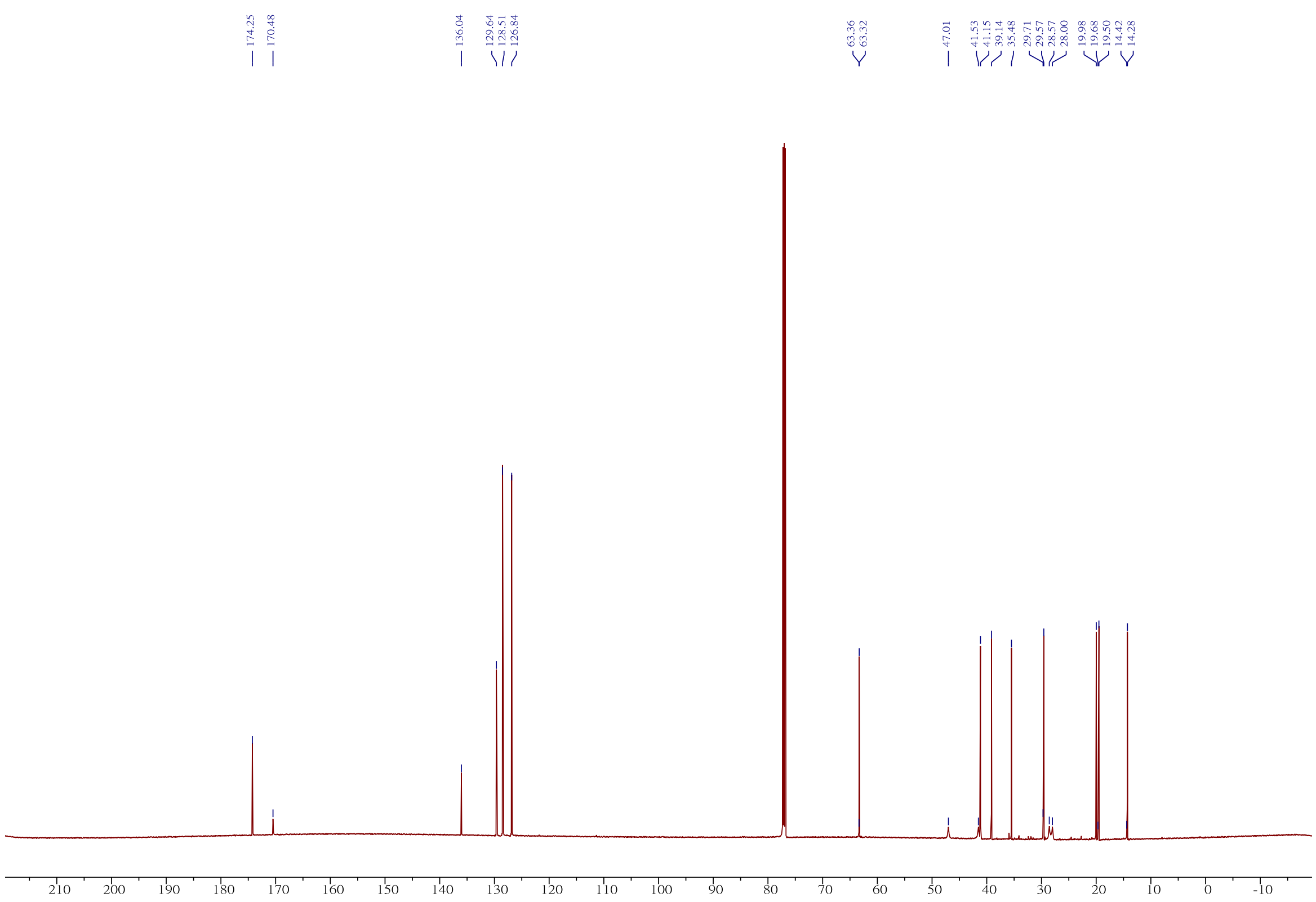

Supplementary Figure 51. ^{13}C NMR (151 MHz) of Compound (15)

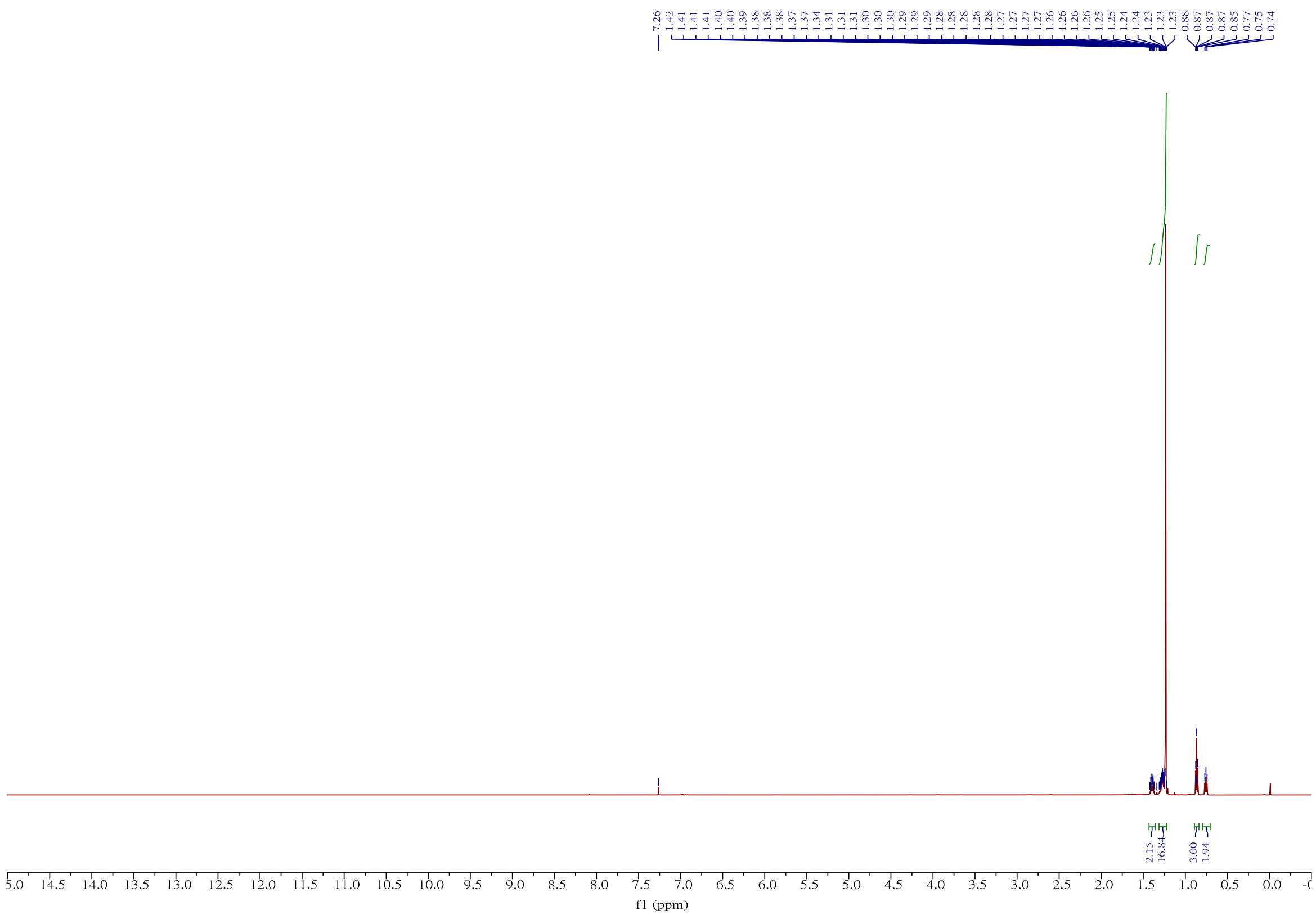

Supplementary Figure 52. ^{1}H NMR (600 MHz) of Compound (16)

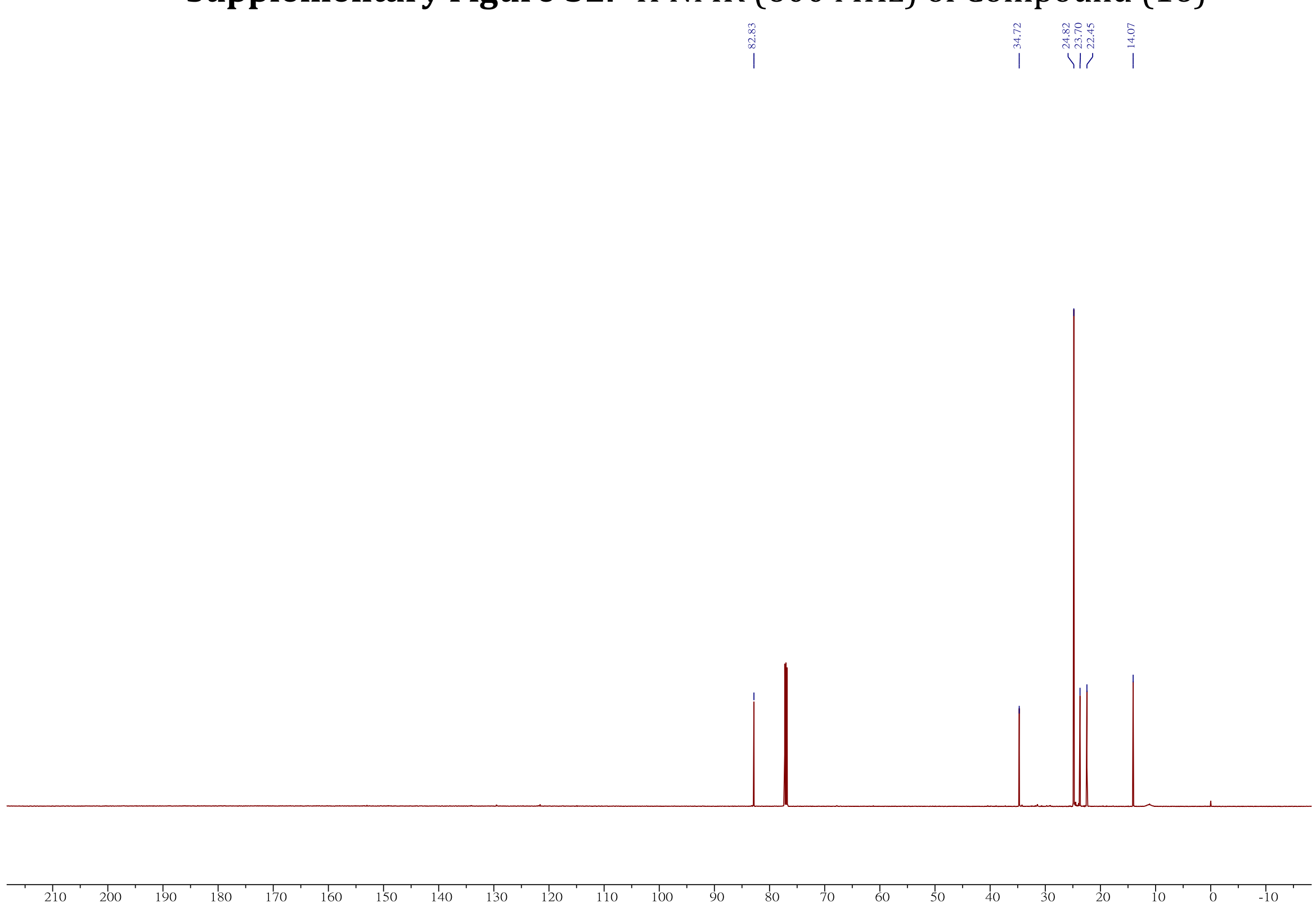

Supplementary Figure 53. ^{13}C NMR (151 MHz) of Compound (16)

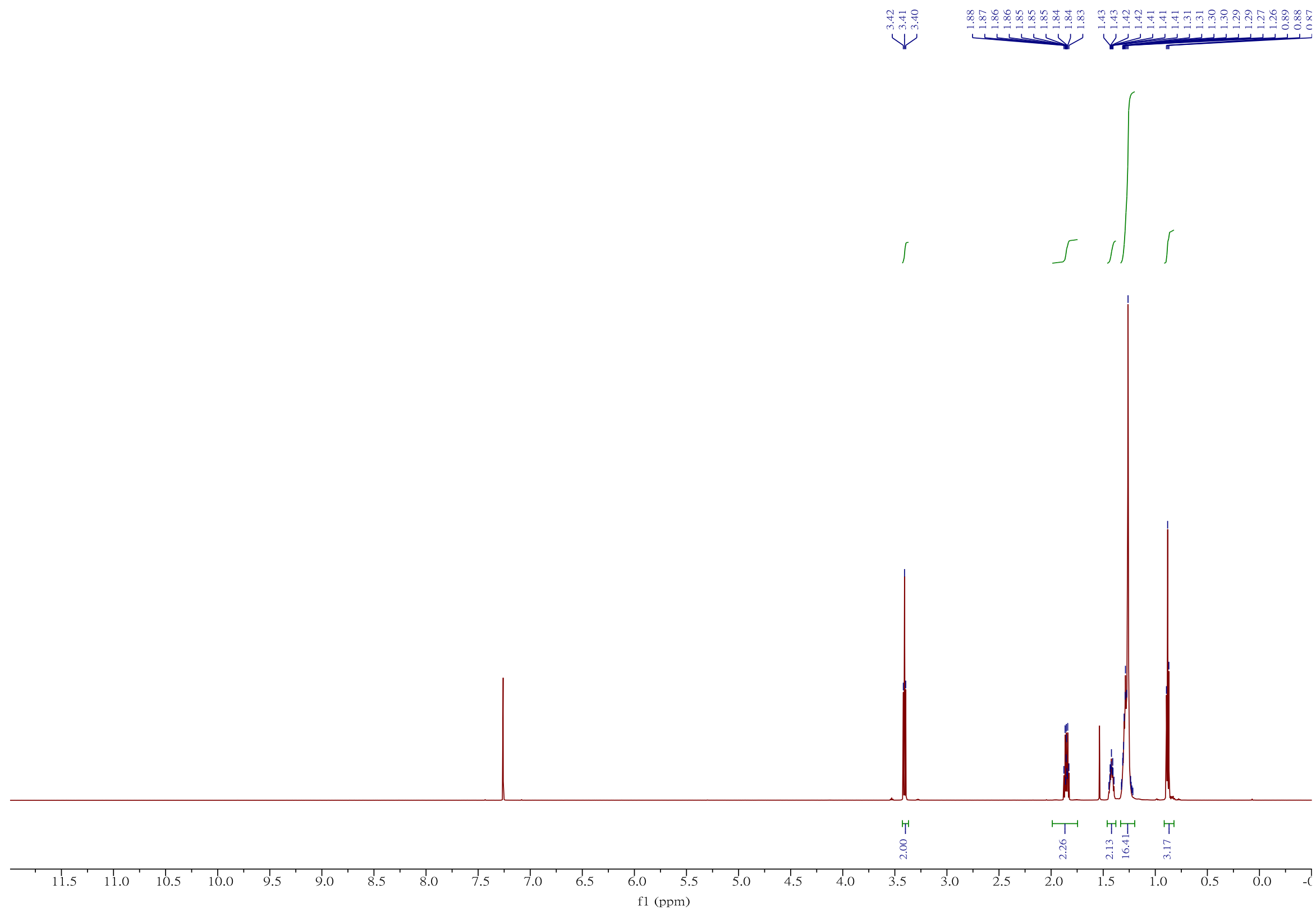

Supplementary Figure 54. ^{1}H NMR (600 MHz) of Compound (17)

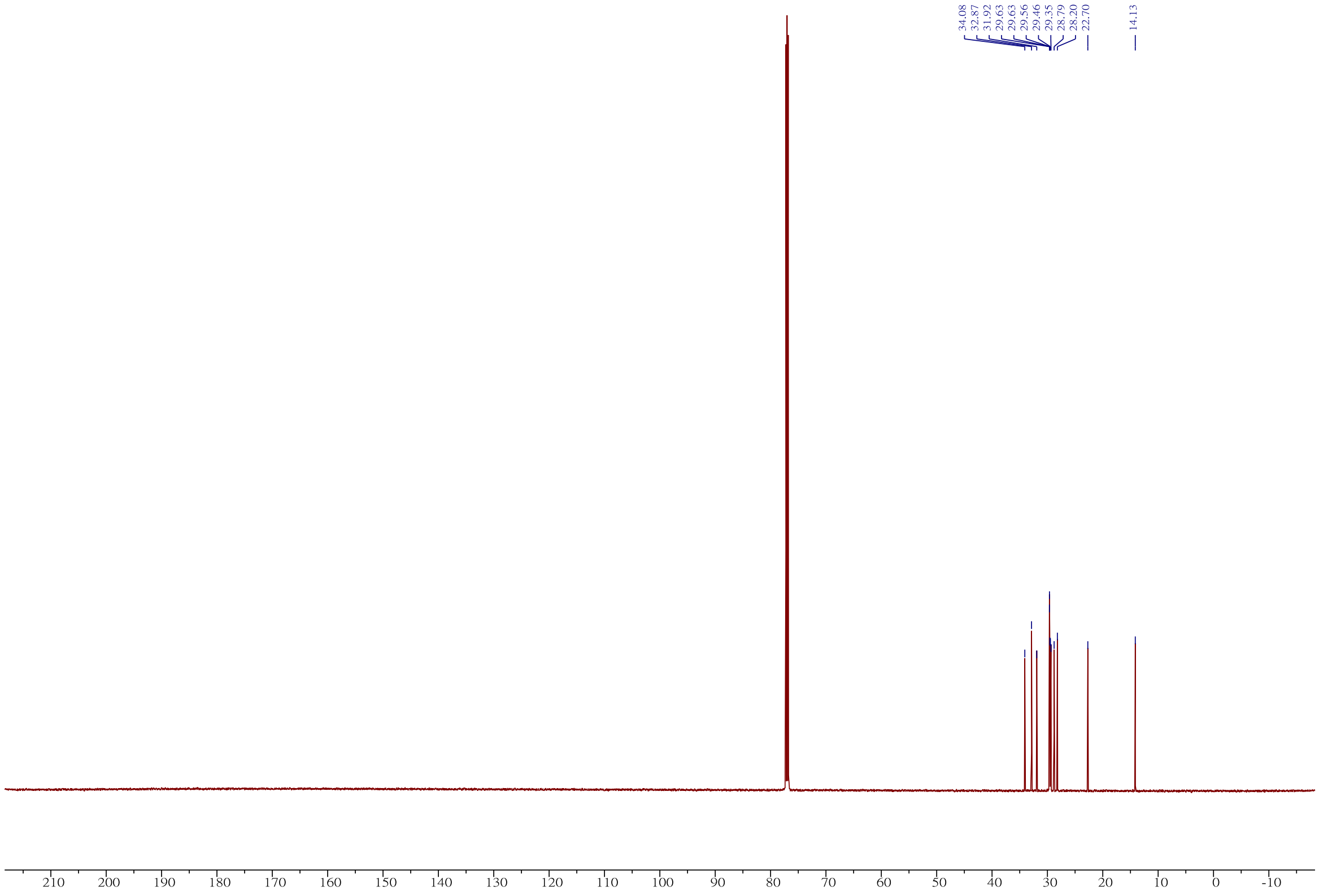

Supplementary Figure 55. ^{13}C NMR (151 MHz) of Compound (17)

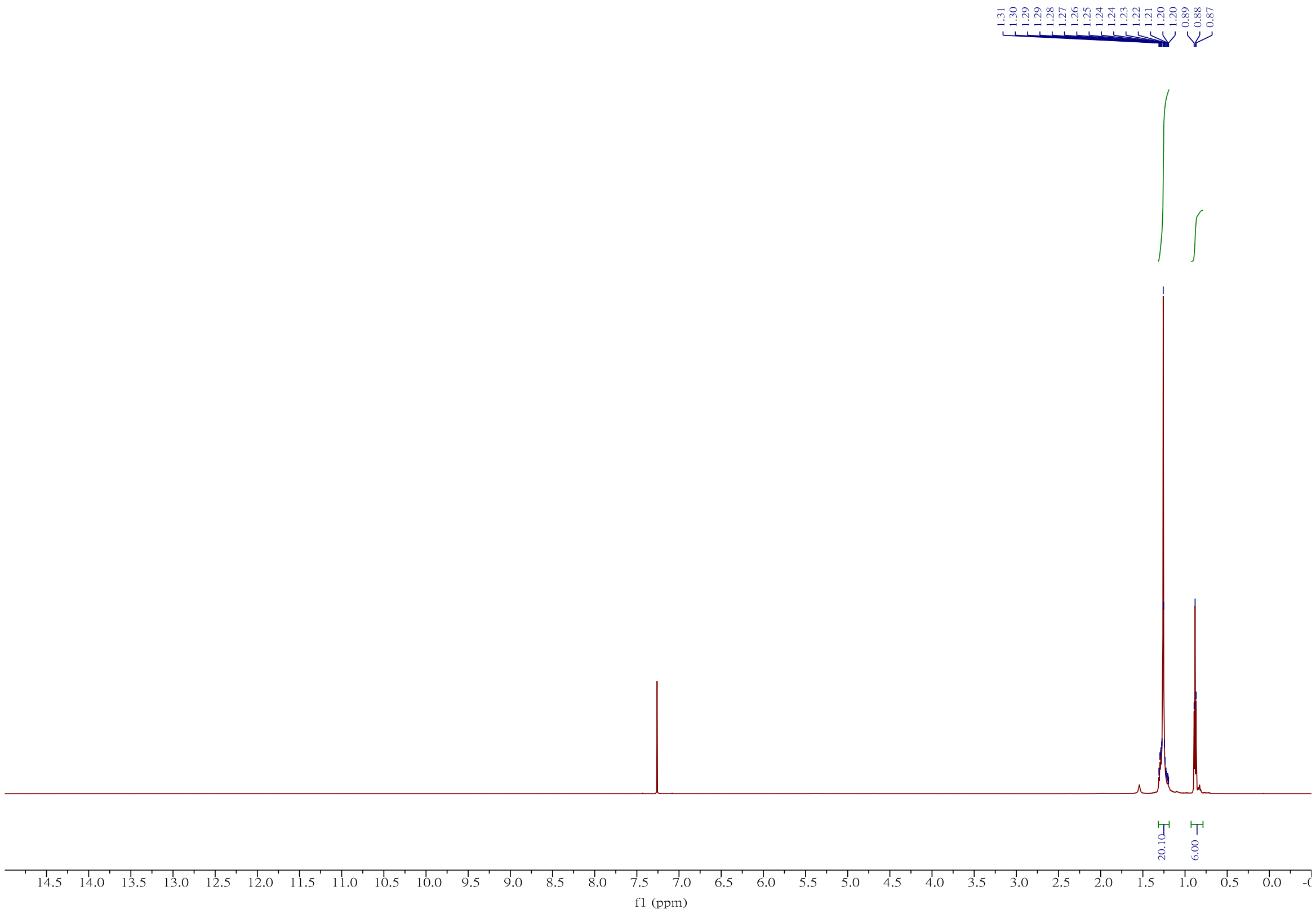

Supplementary Figure 56. ^{1}H NMR (600 MHz) of Compound (18)

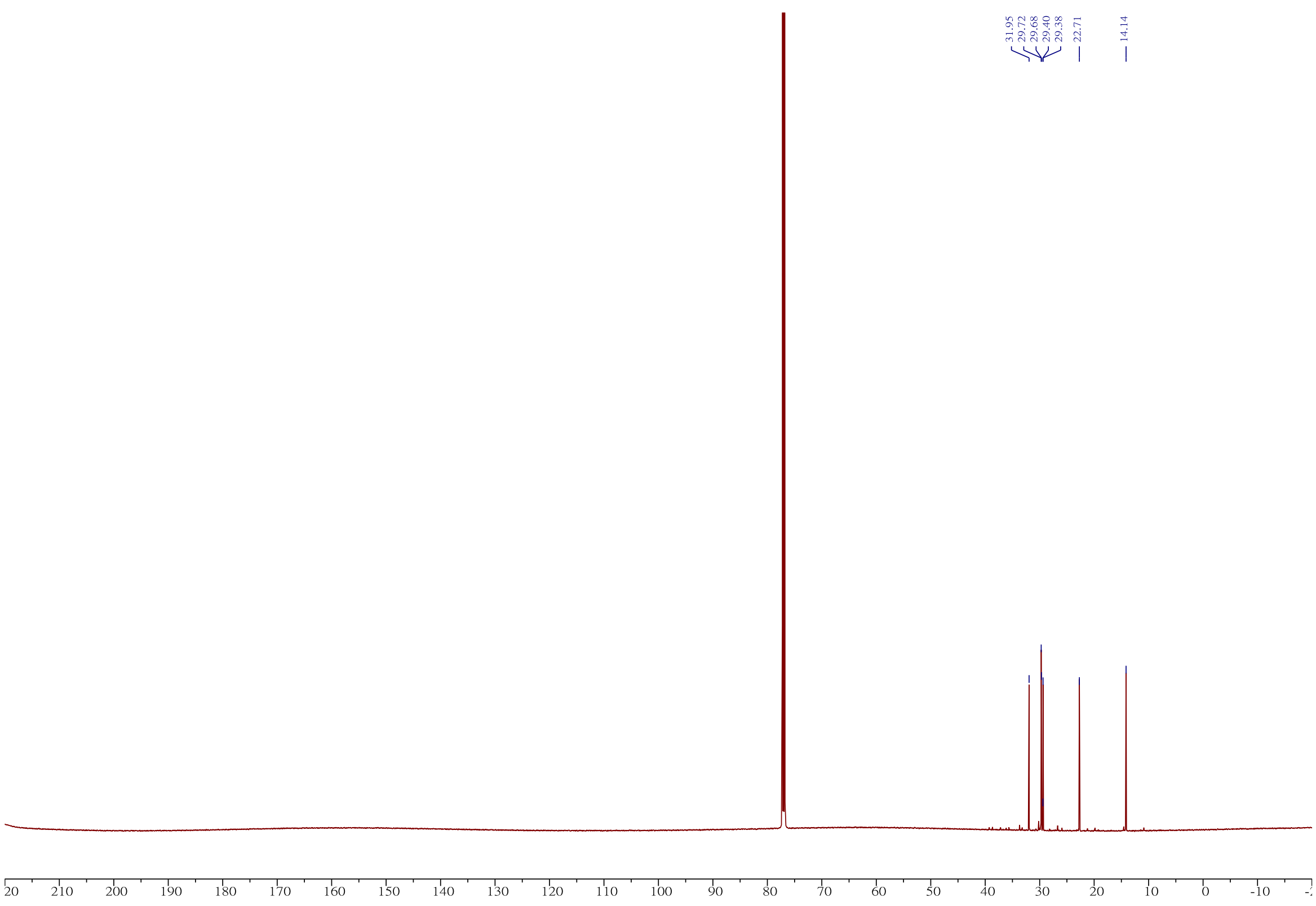

Supplementary Figure 57. ^{13}C NMR (151 MHz) of Compound (18)

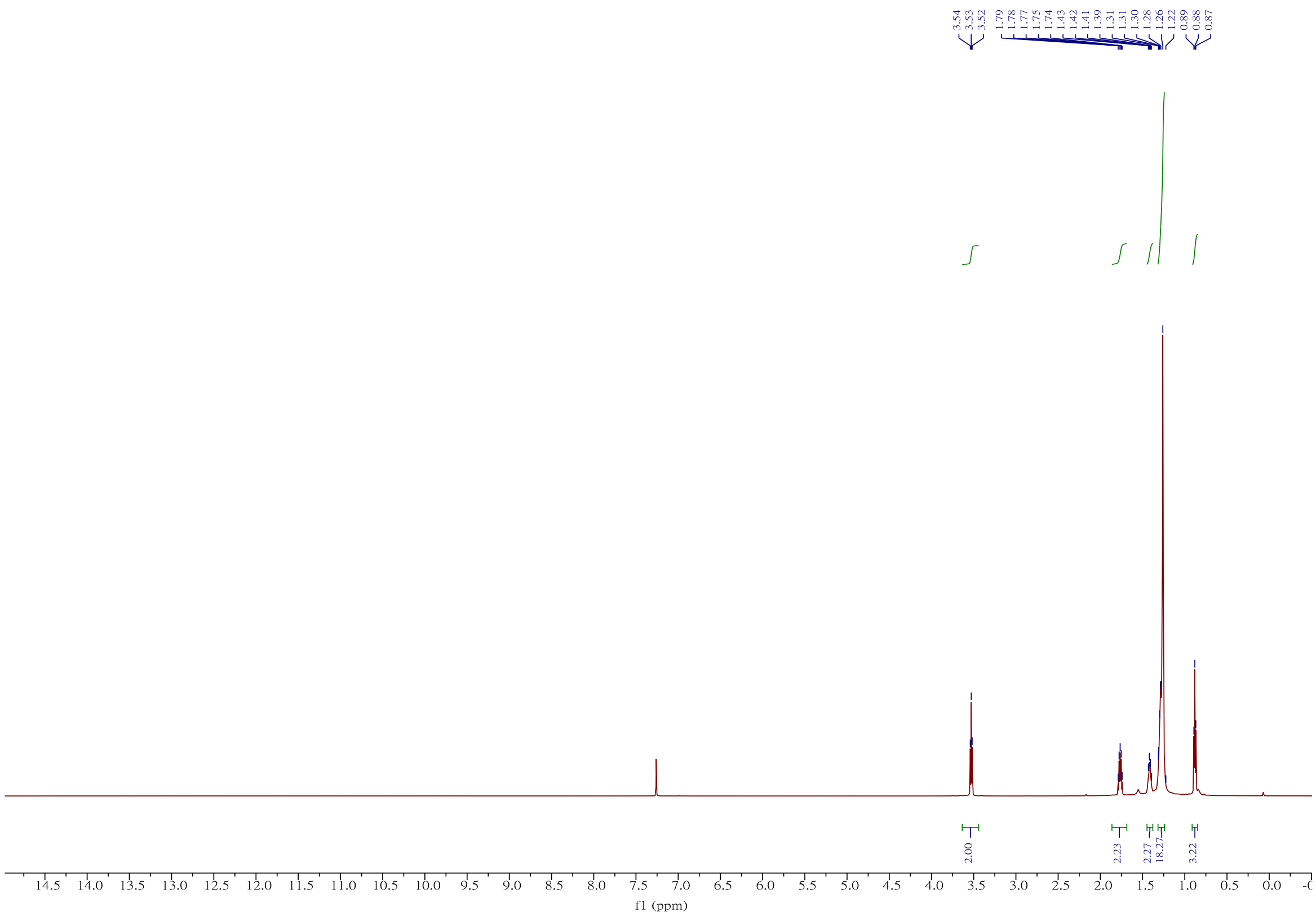

Supplementary Figure 58. ^{1}H NMR (600 MHz) of Compound (19)

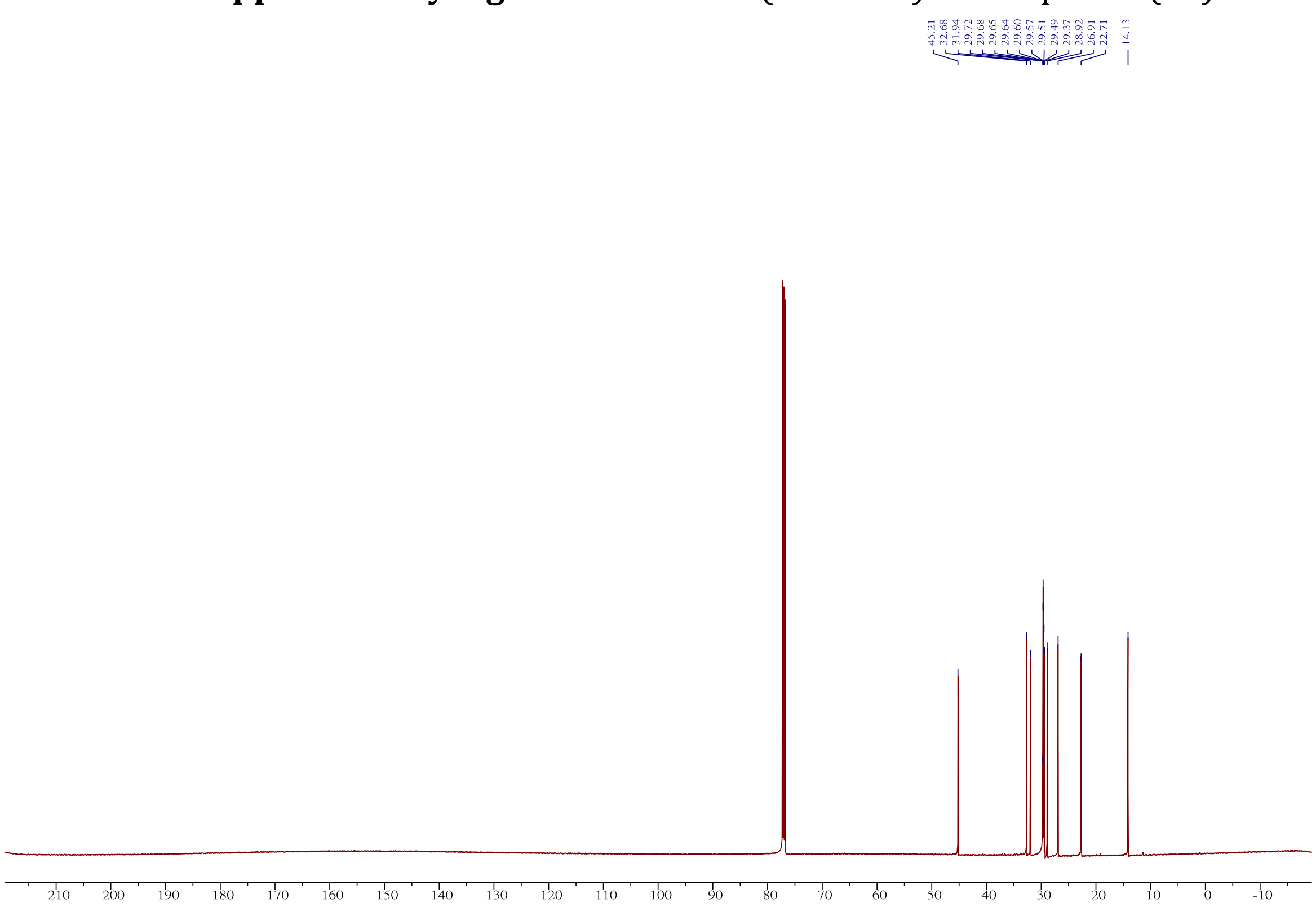

Supplementary Figure 59. ^{13}C NMR (151 MHz) of Compound (19)

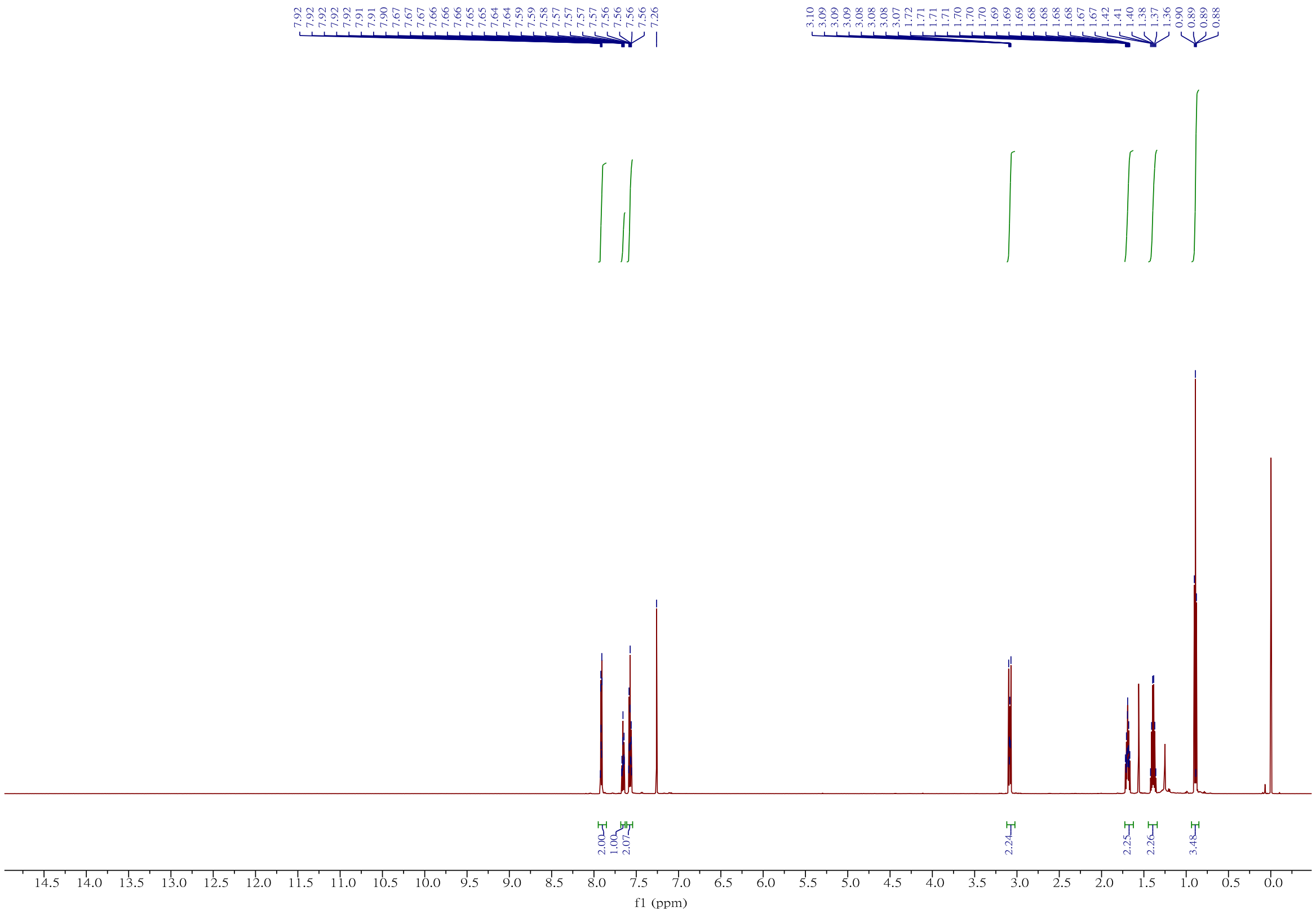

Supplementary Figure 60. ^{1}H NMR (600 MHz) of Compound (21)

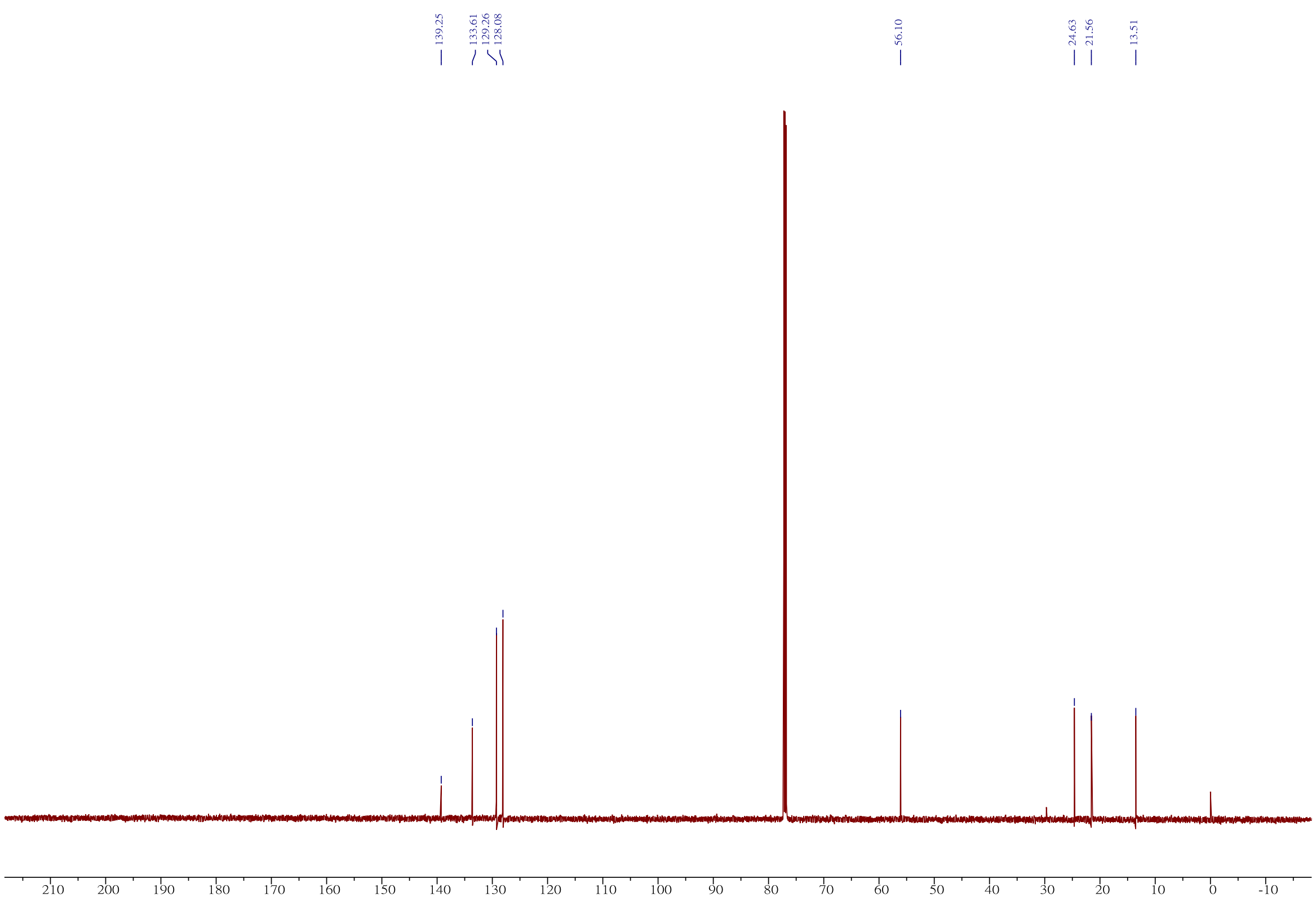

Supplementary Figure 61. ^{13}C NMR (151 MHz) of Compound (21)

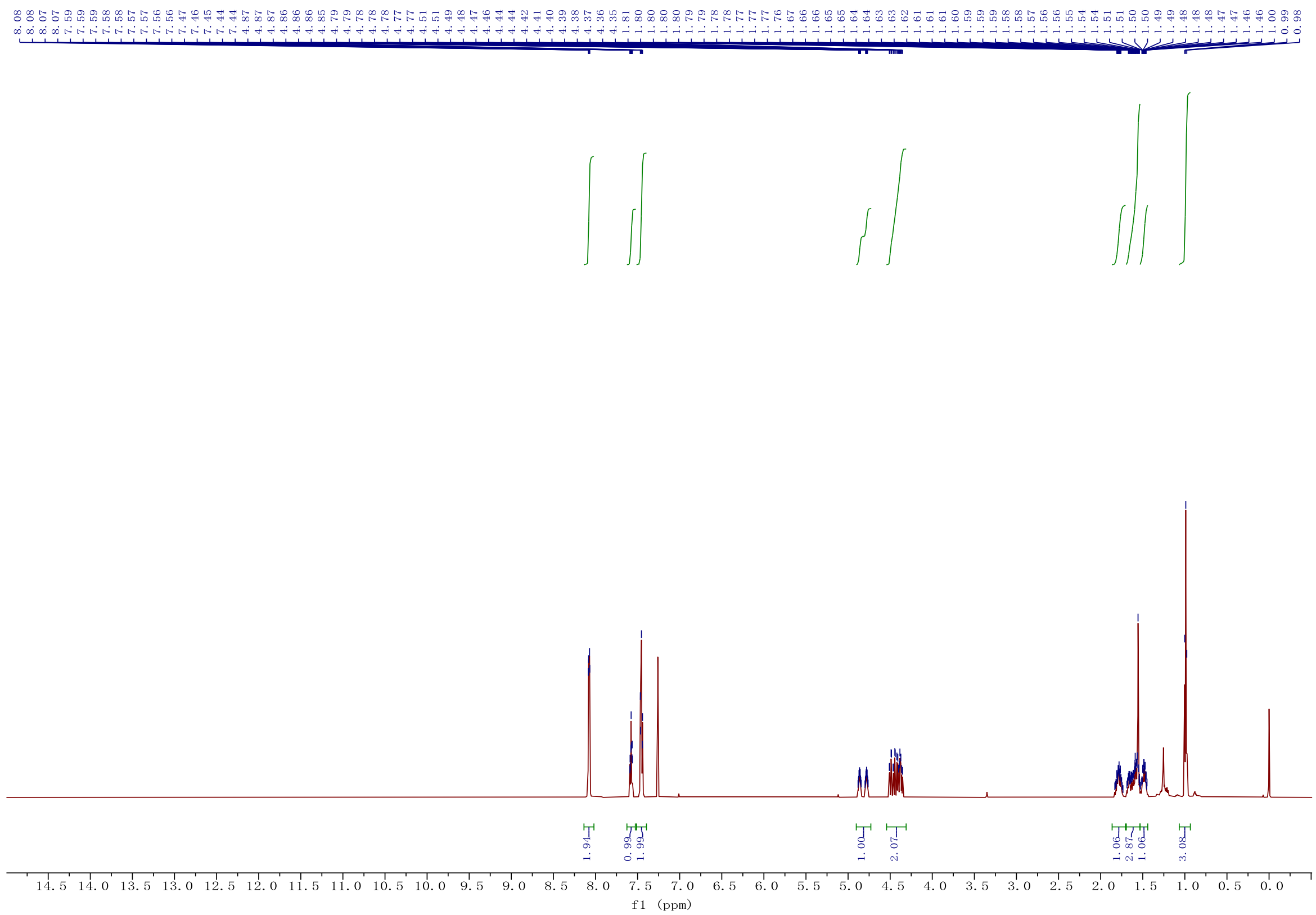

Supplementary Figure 62. ^{1}H NMR (600 MHz) of Compound (22)

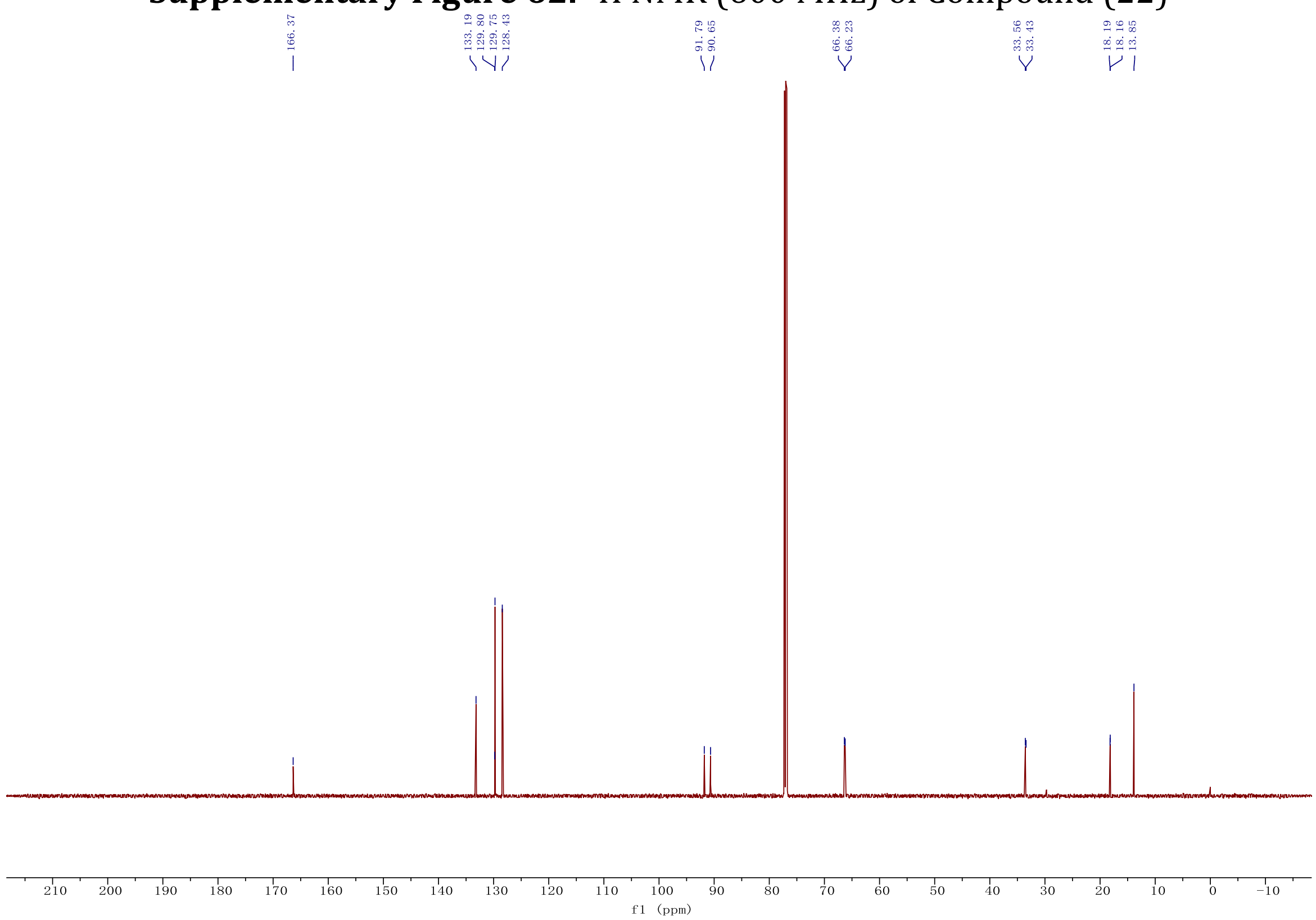

Supplementary Figure 63. ^{13}C NMR (151 MHz) of Compound (22)

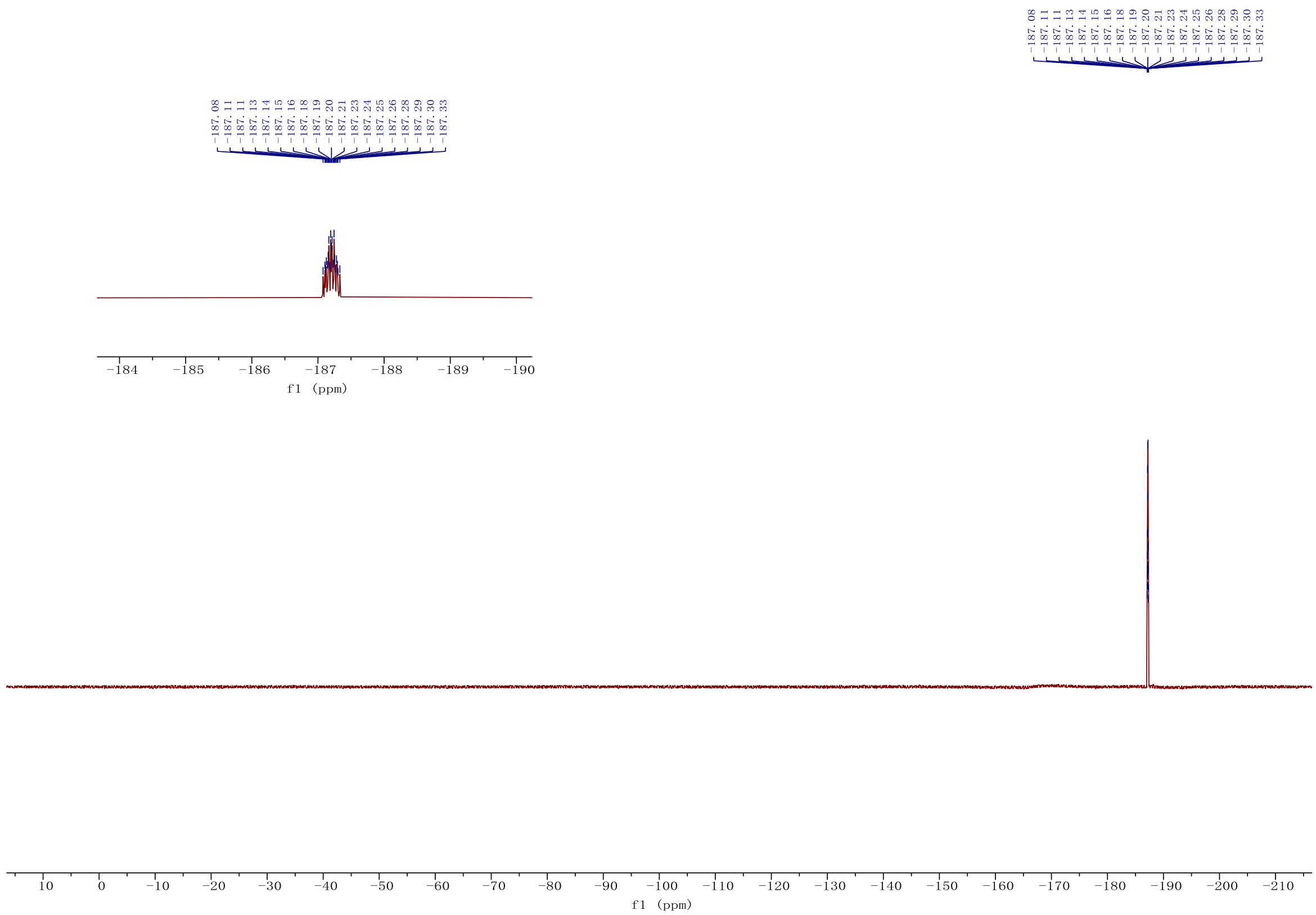

Supplementary Figure 64. ^{19}F NMR (564 MHz) of Compound (22)

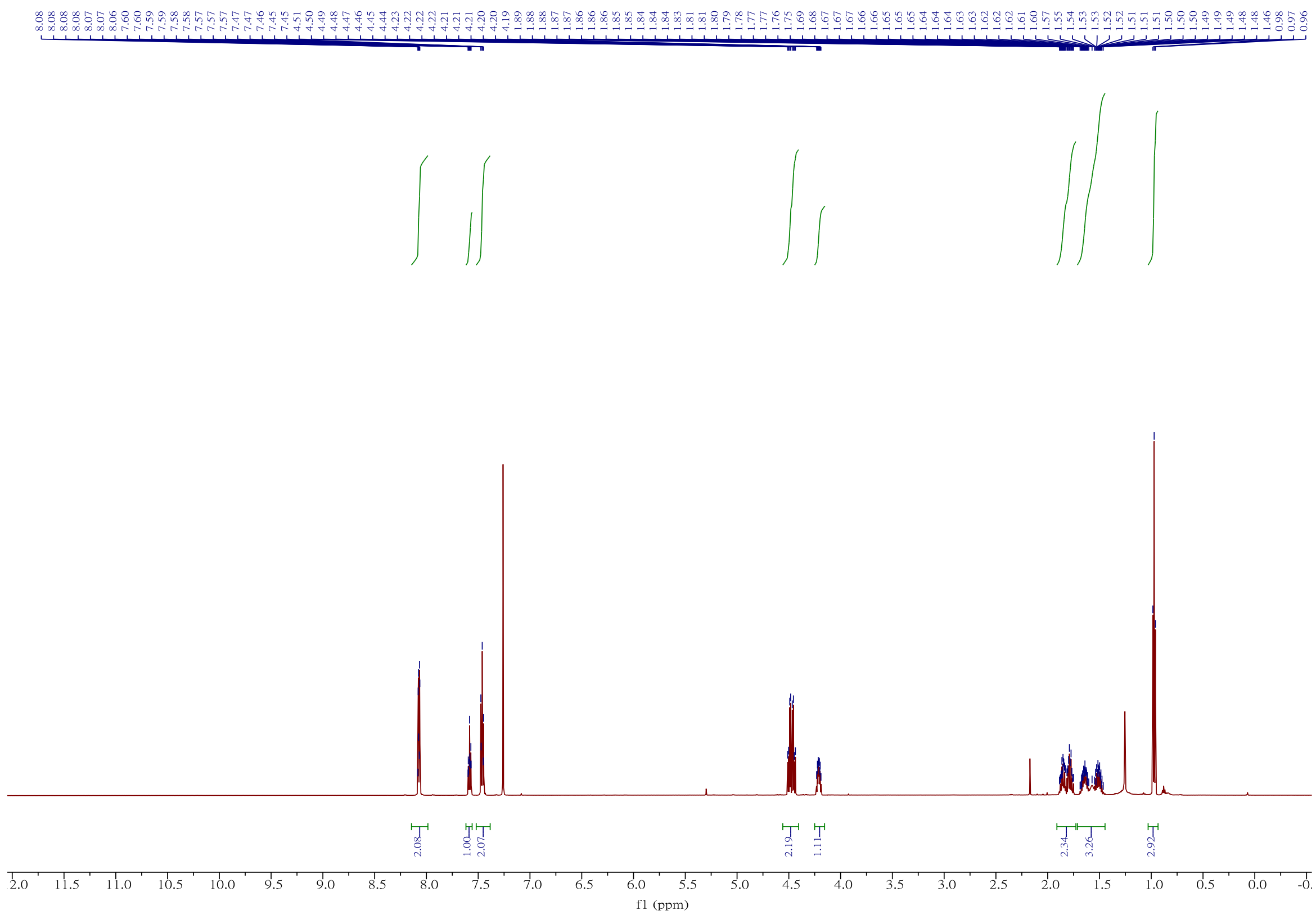

Supplementary Figure 65. ^{1}H NMR (600 MHz) of Compound (23)

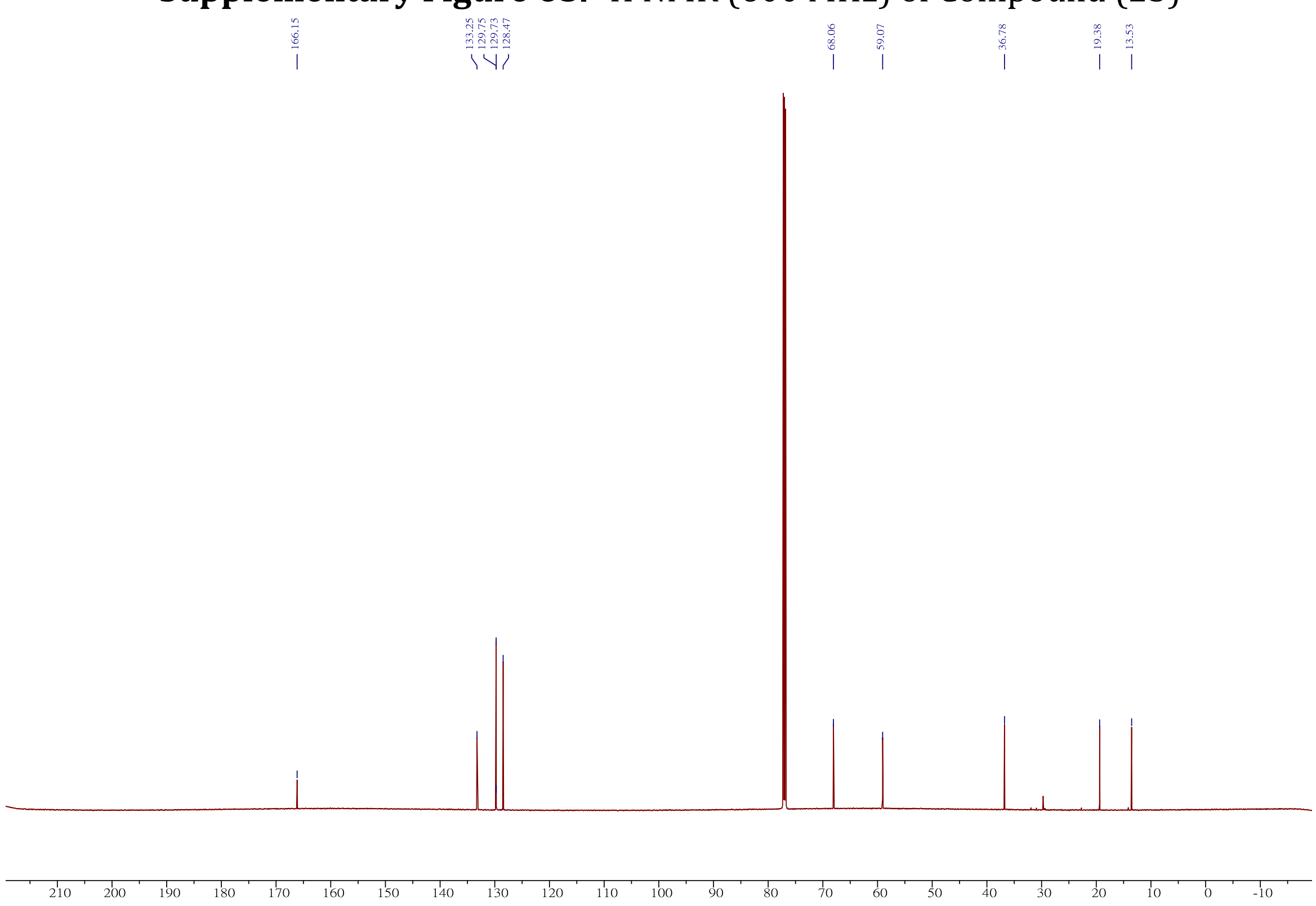

Supplementary Figure 66. ^{13}C NMR (151 MHz) of Compound (23)

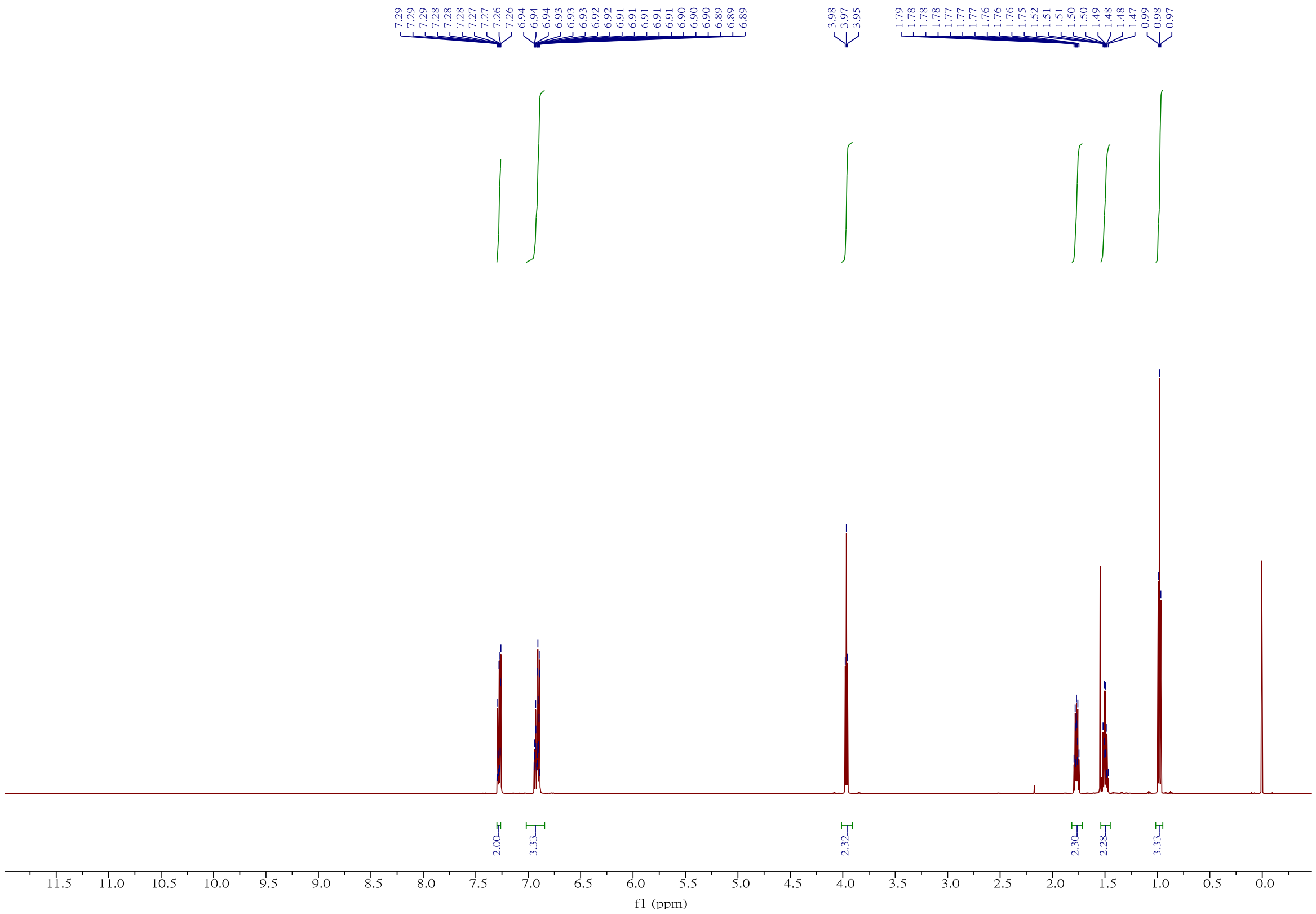

Supplementary Figure 67. ^{1}H NMR (600 MHz) of Compound (24)

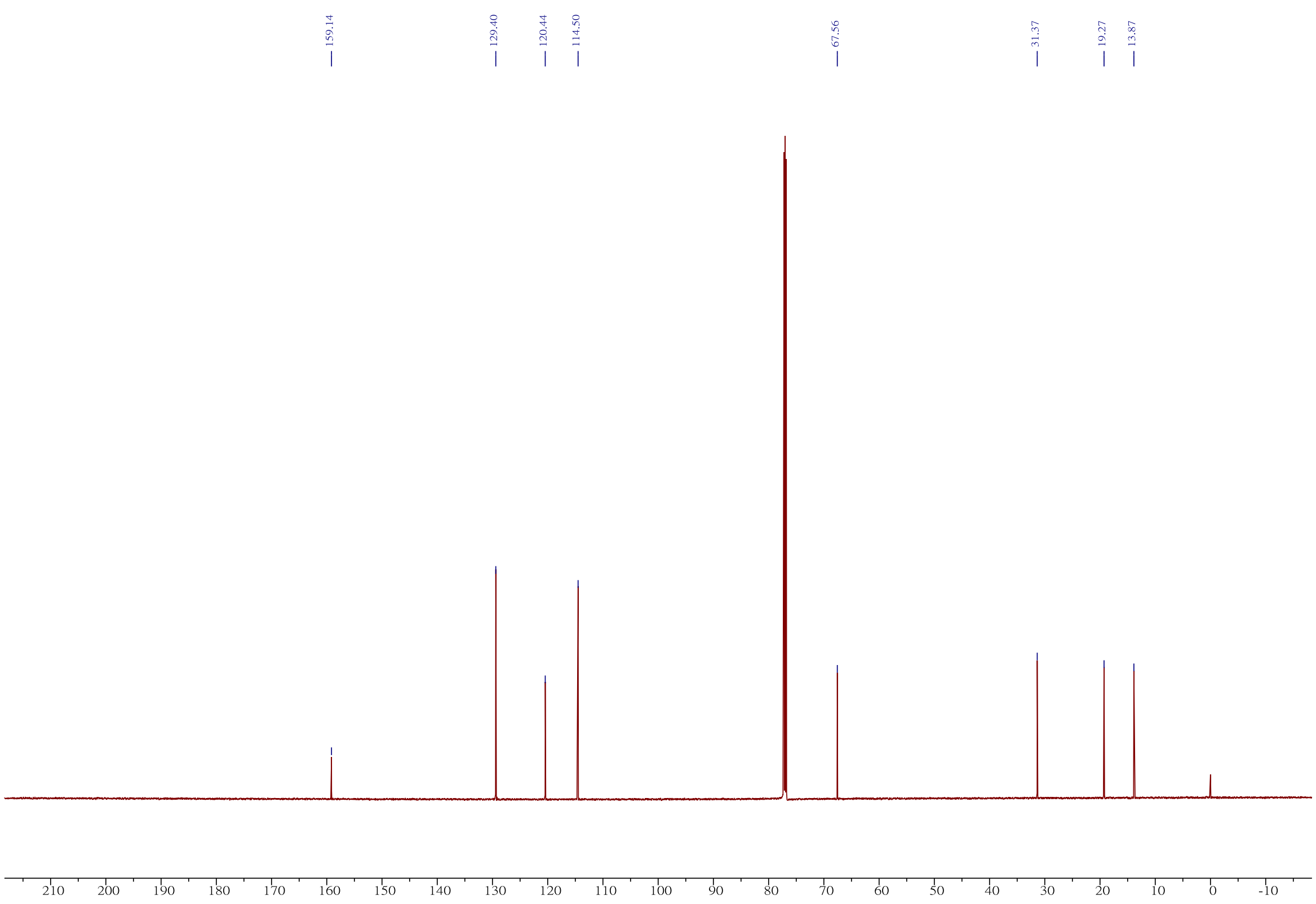

Supplementary Figure 68. ^{13}C NMR (151 MHz) of Compound (24)

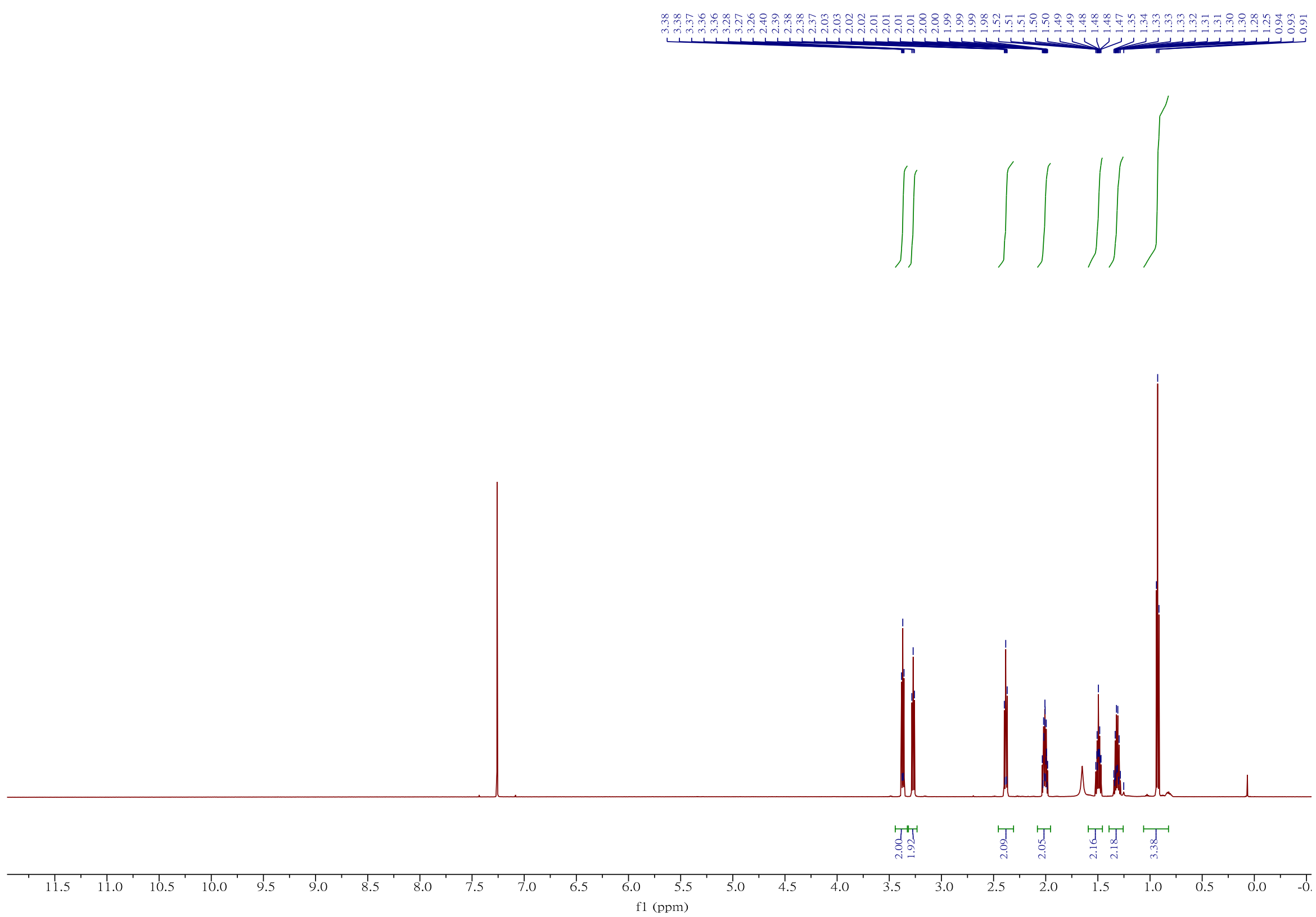

Supplementary Figure 69. ^{1}H NMR (600 MHz) of Compound (25)

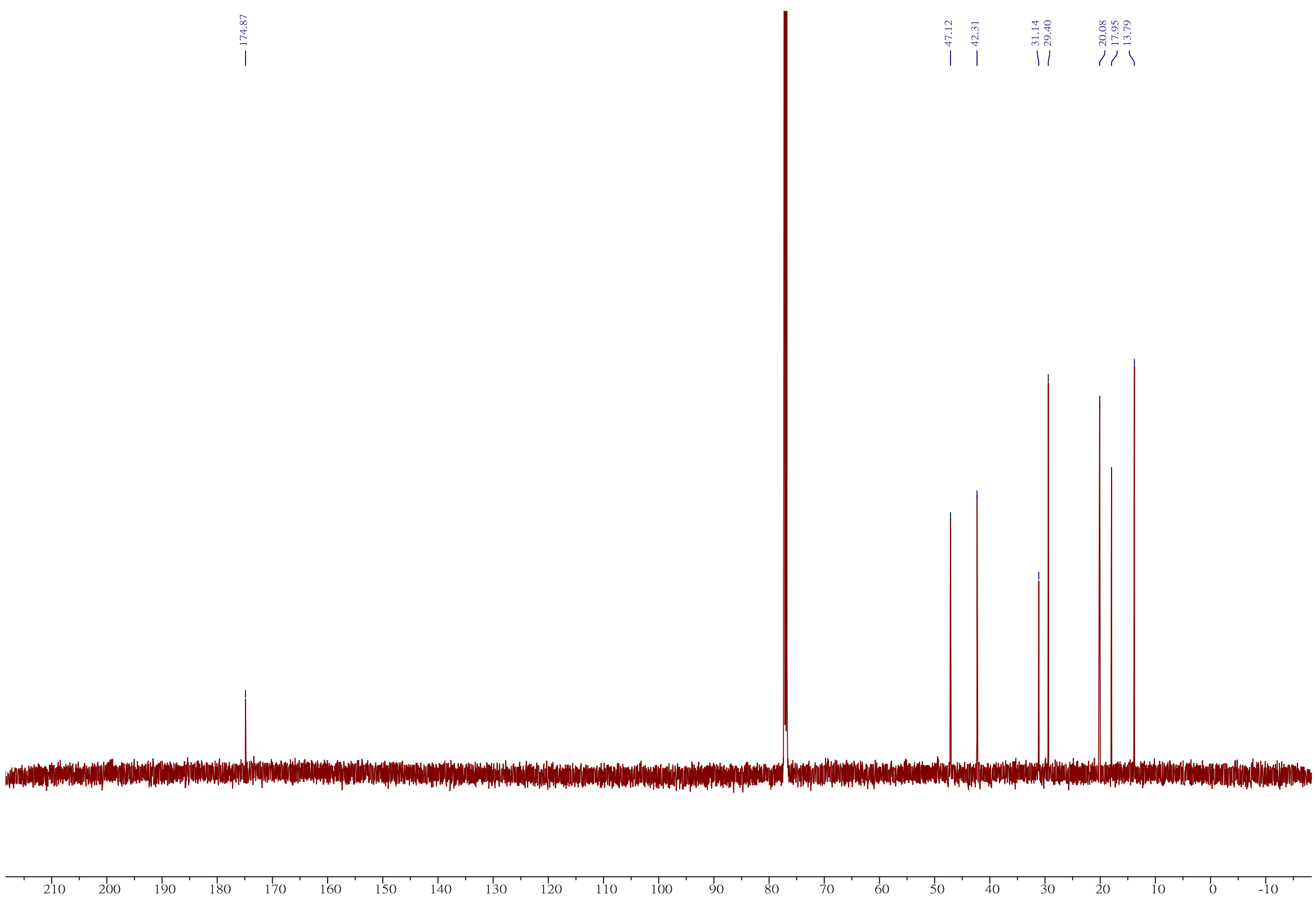

Supplementary Figure 70. ^{13}C NMR (151 MHz) of Compound (25)

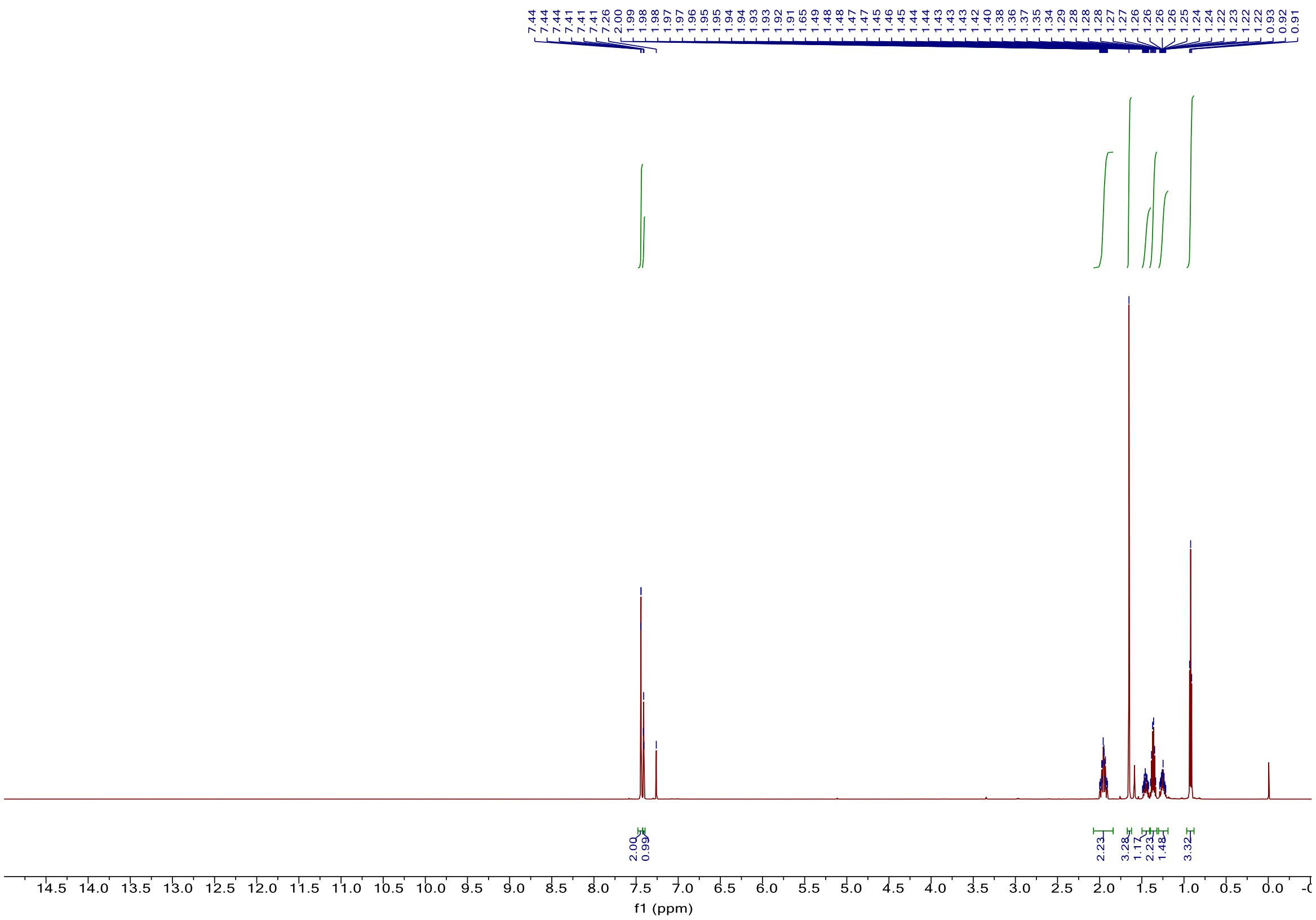

Supplementary Figure 71. ^{1}H NMR (600 MHz) of Compound (26)

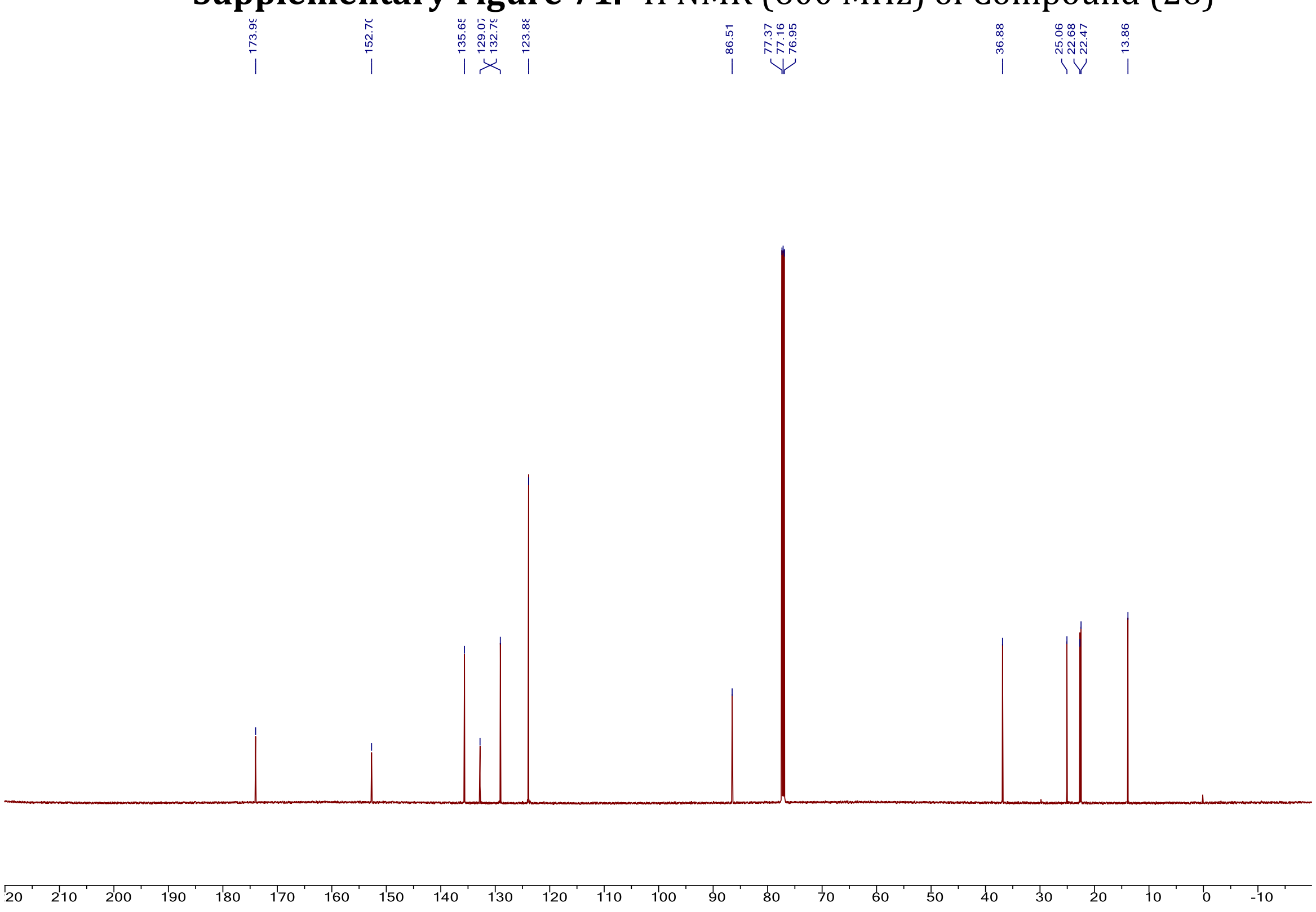

Supplementary Figure 72. ^{13}C NMR (151 MHz) of Compound (26)

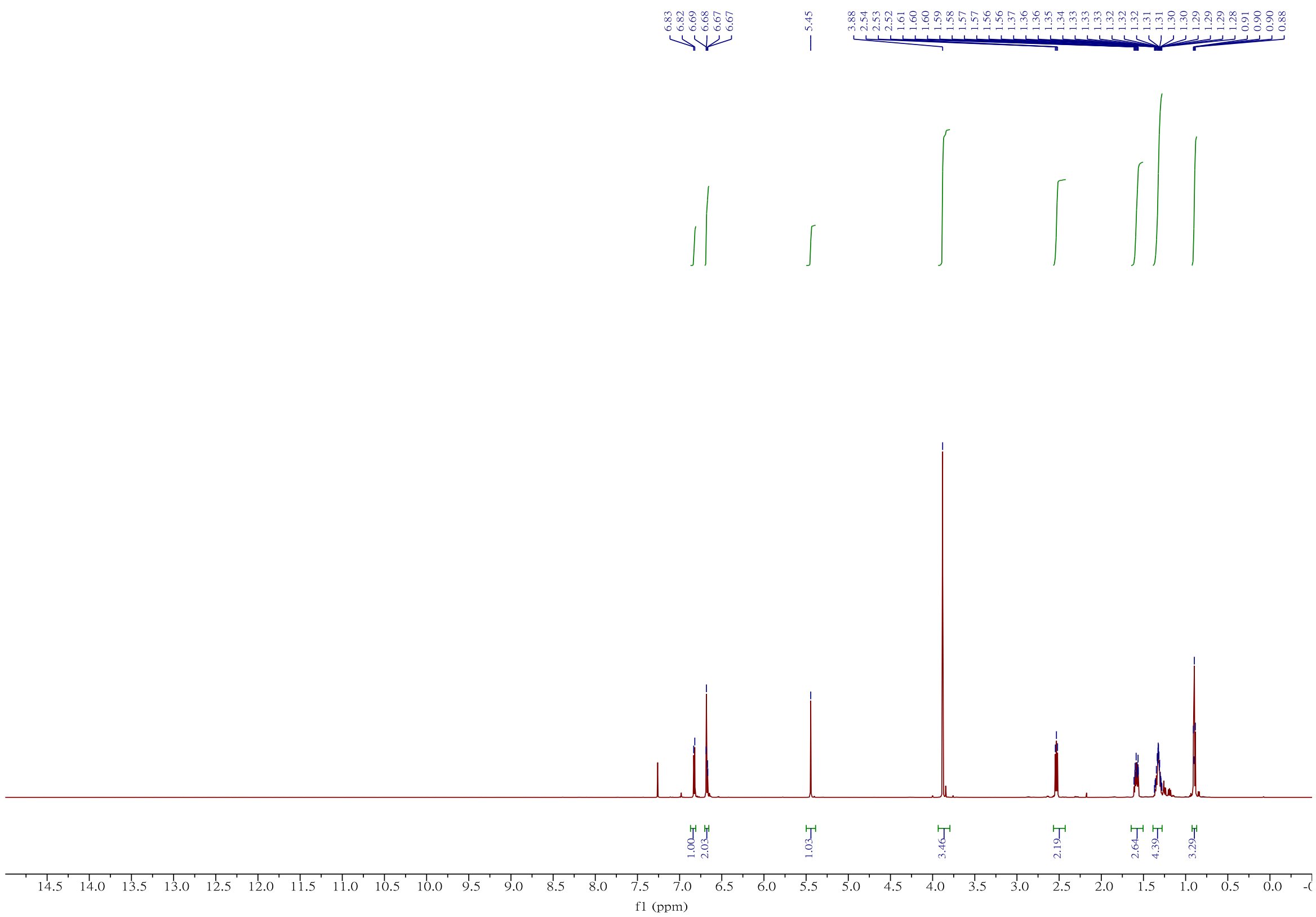

Supplementary Figure 73. ^{1}H NMR (600 MHz) of Compound (27)

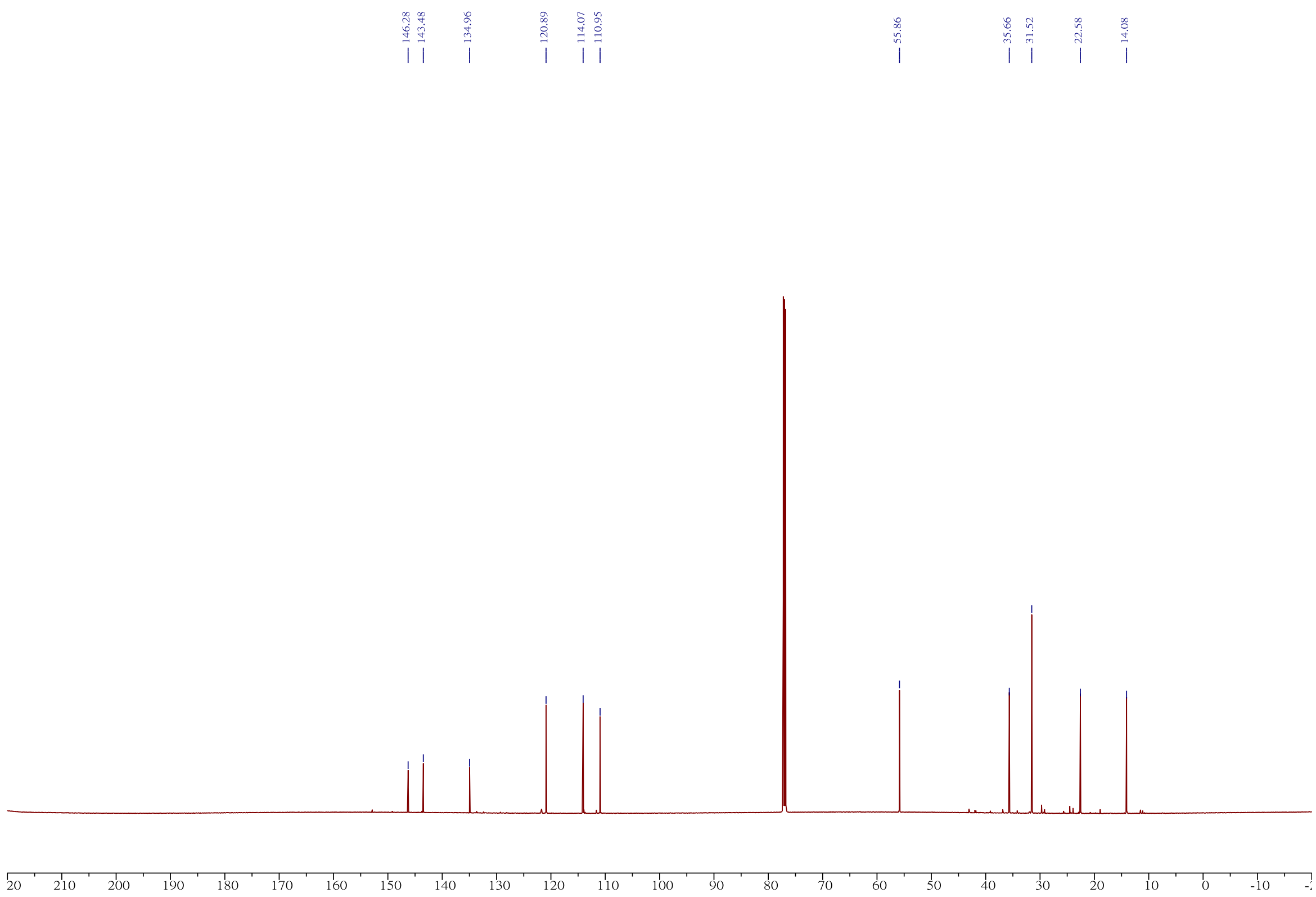

Supplementary Figure 74. ^{13}C NMR (151 MHz) of Compound (27)

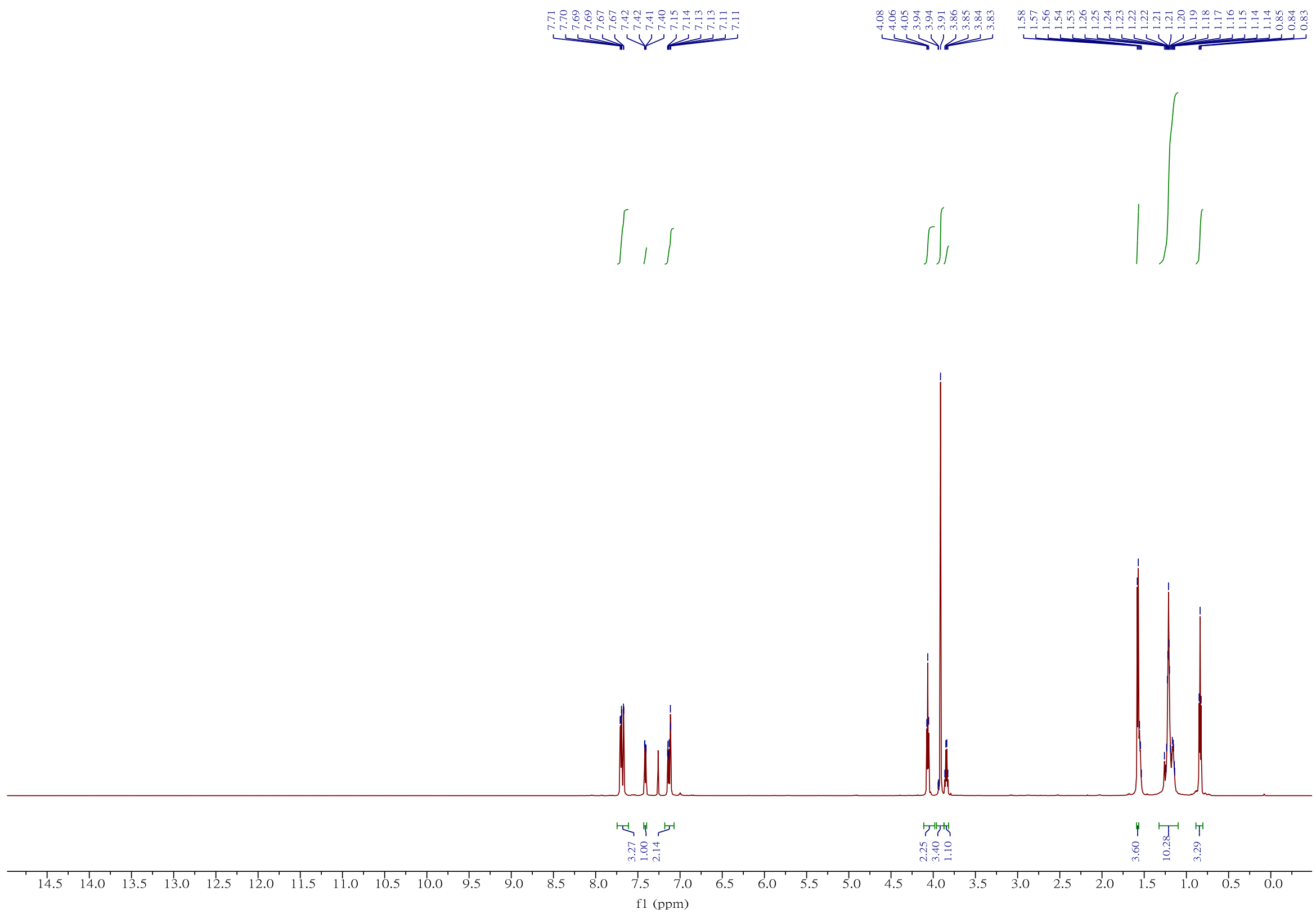

Supplementary Figure 75. ^{1}H NMR (600 MHz) of Compound (28)

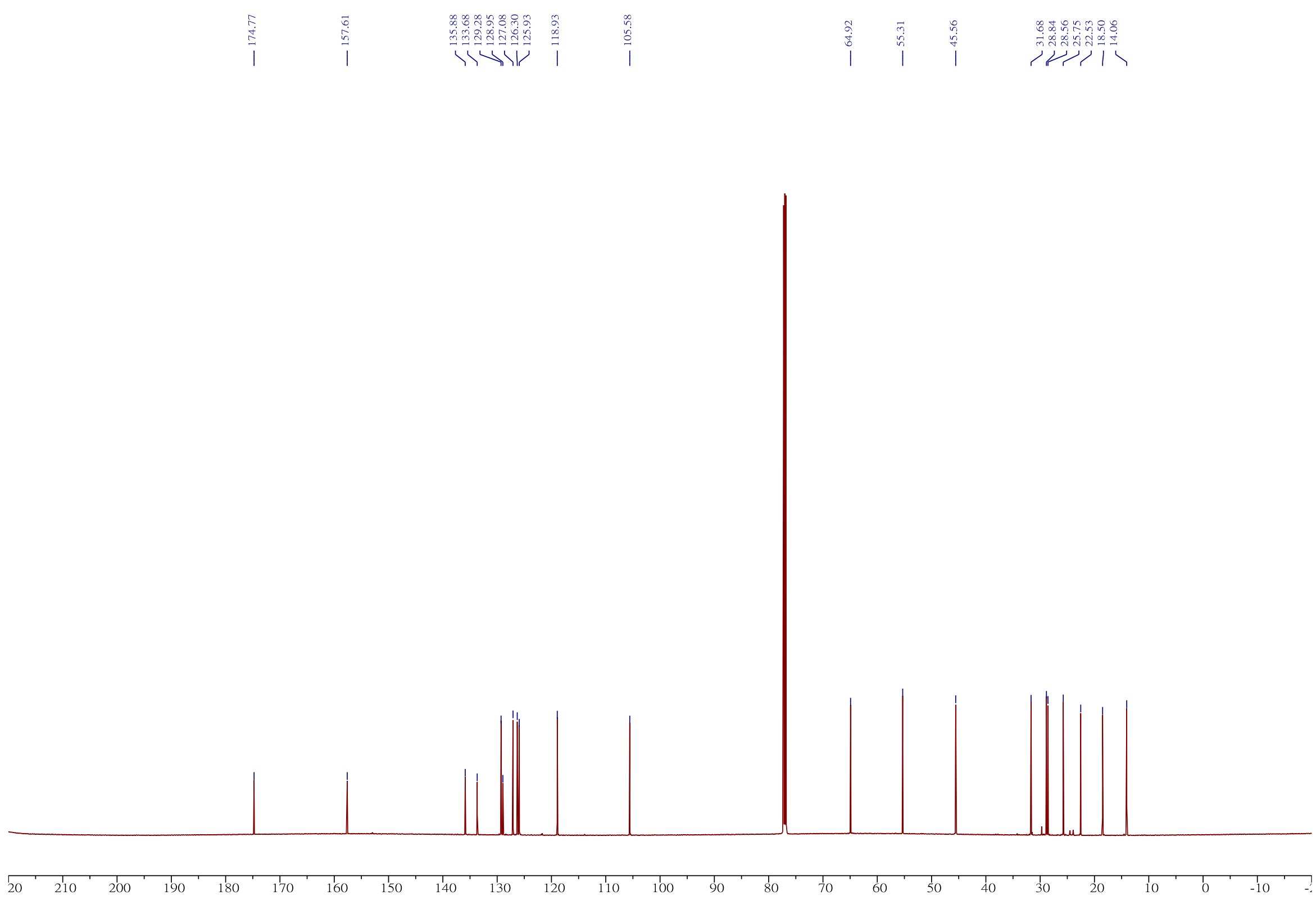

Supplementary Figure 76. ^{13}C NMR (151 MHz) of Compound (28)

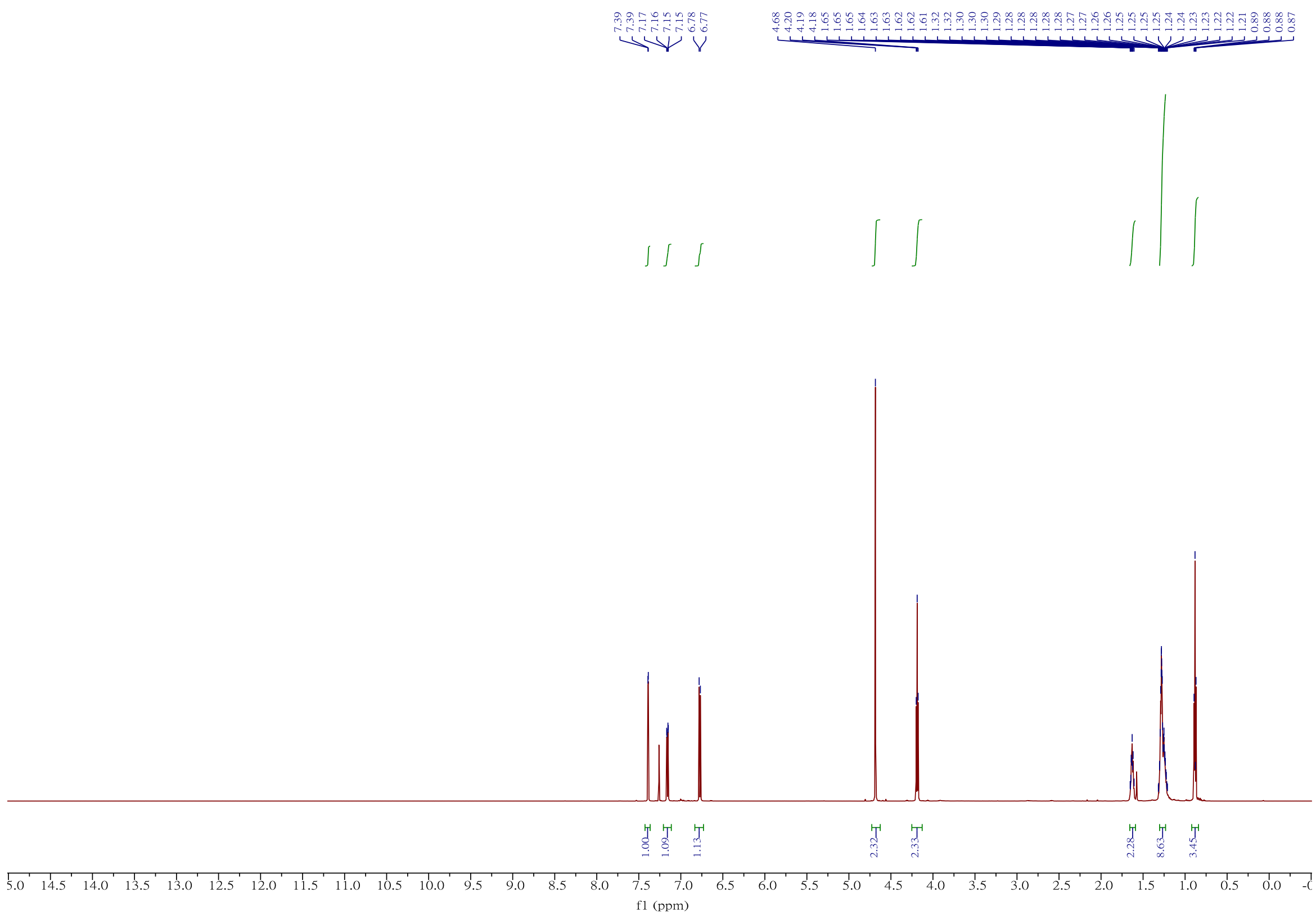

Supplementary Figure 77. ^{1}H NMR (600 MHz) of Compound (29)

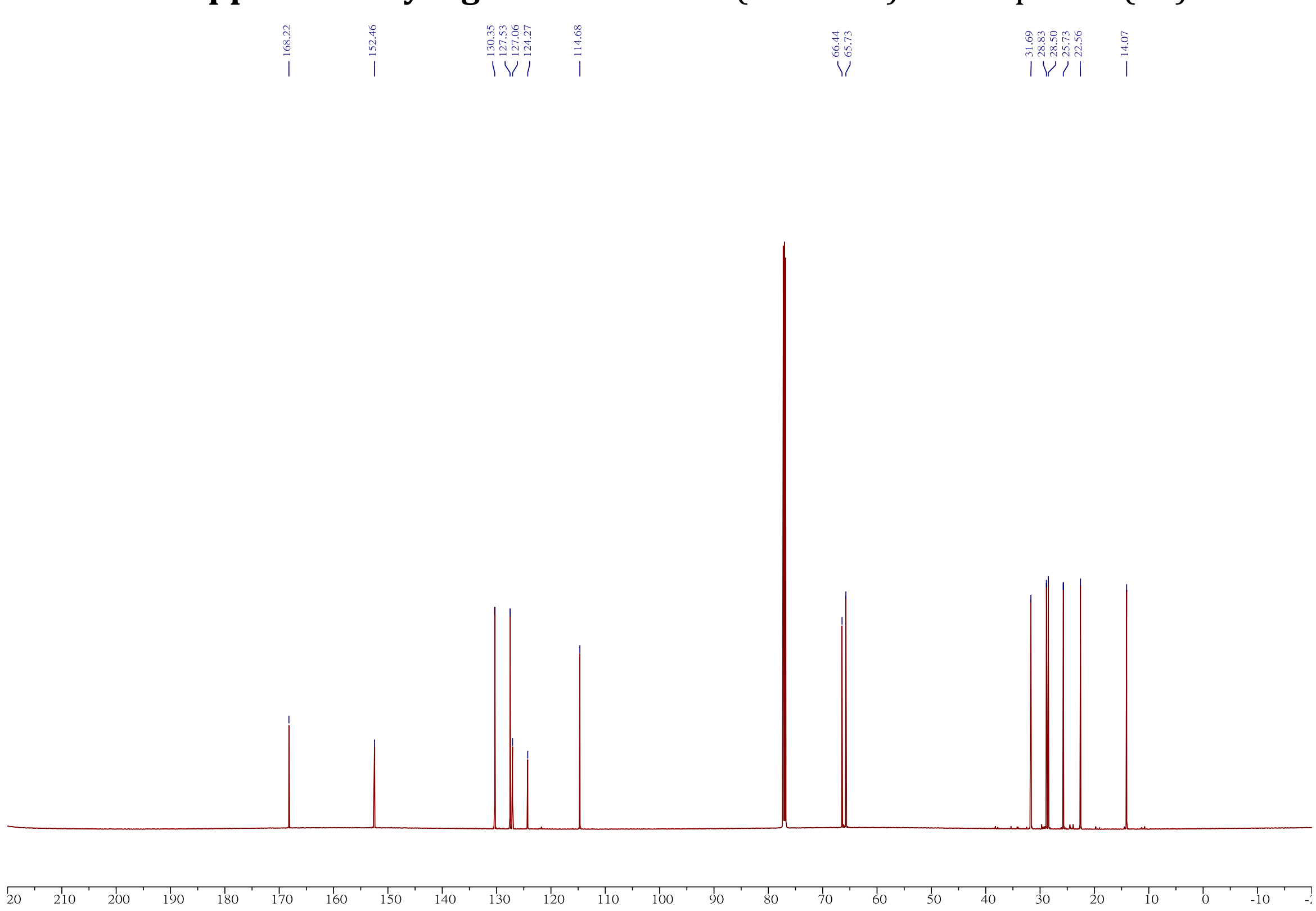

Supplementary Figure 78. ^{13}C NMR (151 MHz) of Compound (29)

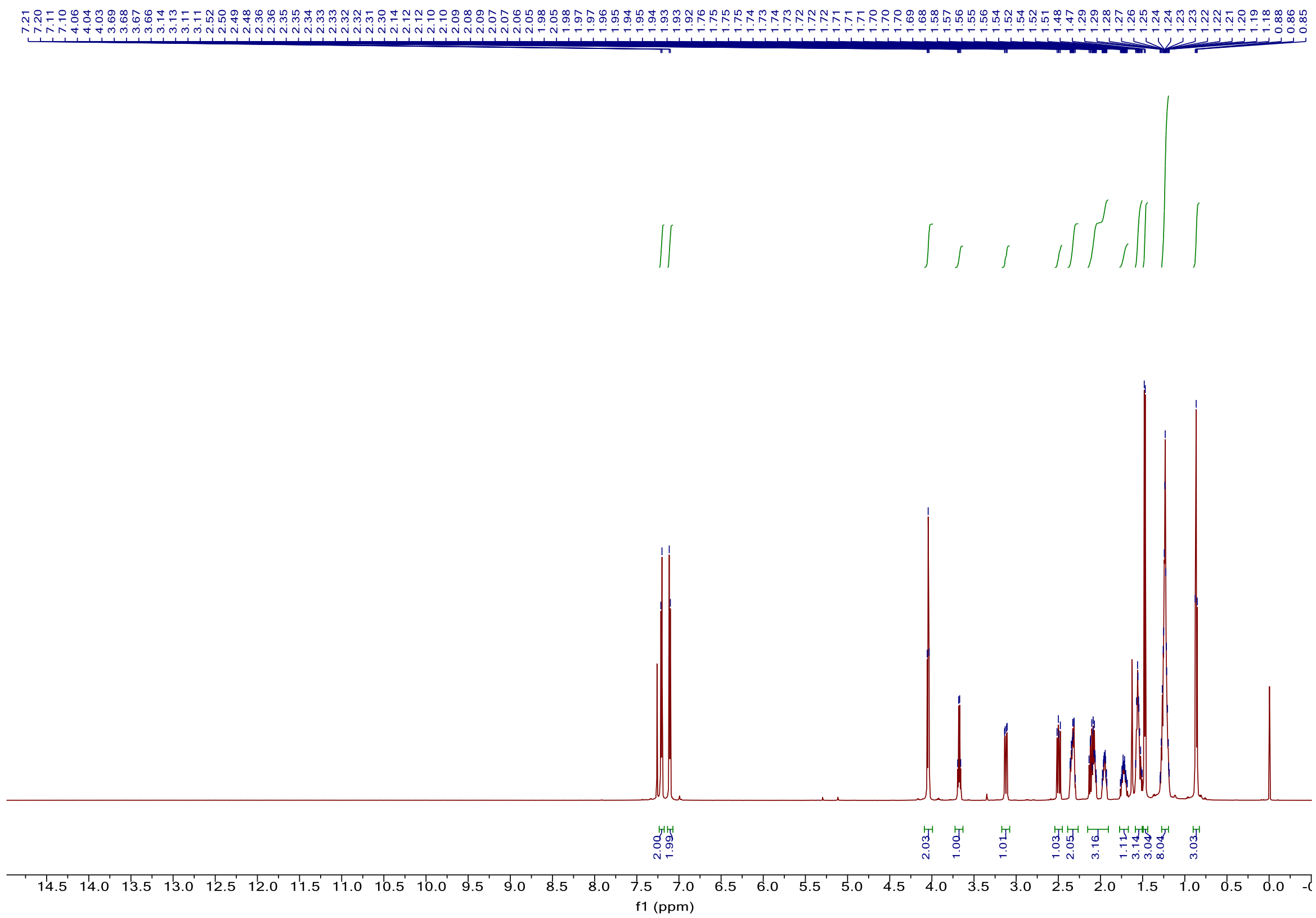

Supplementary Figure 79. ^{1}H NMR (600 MHz) of Compound (30)

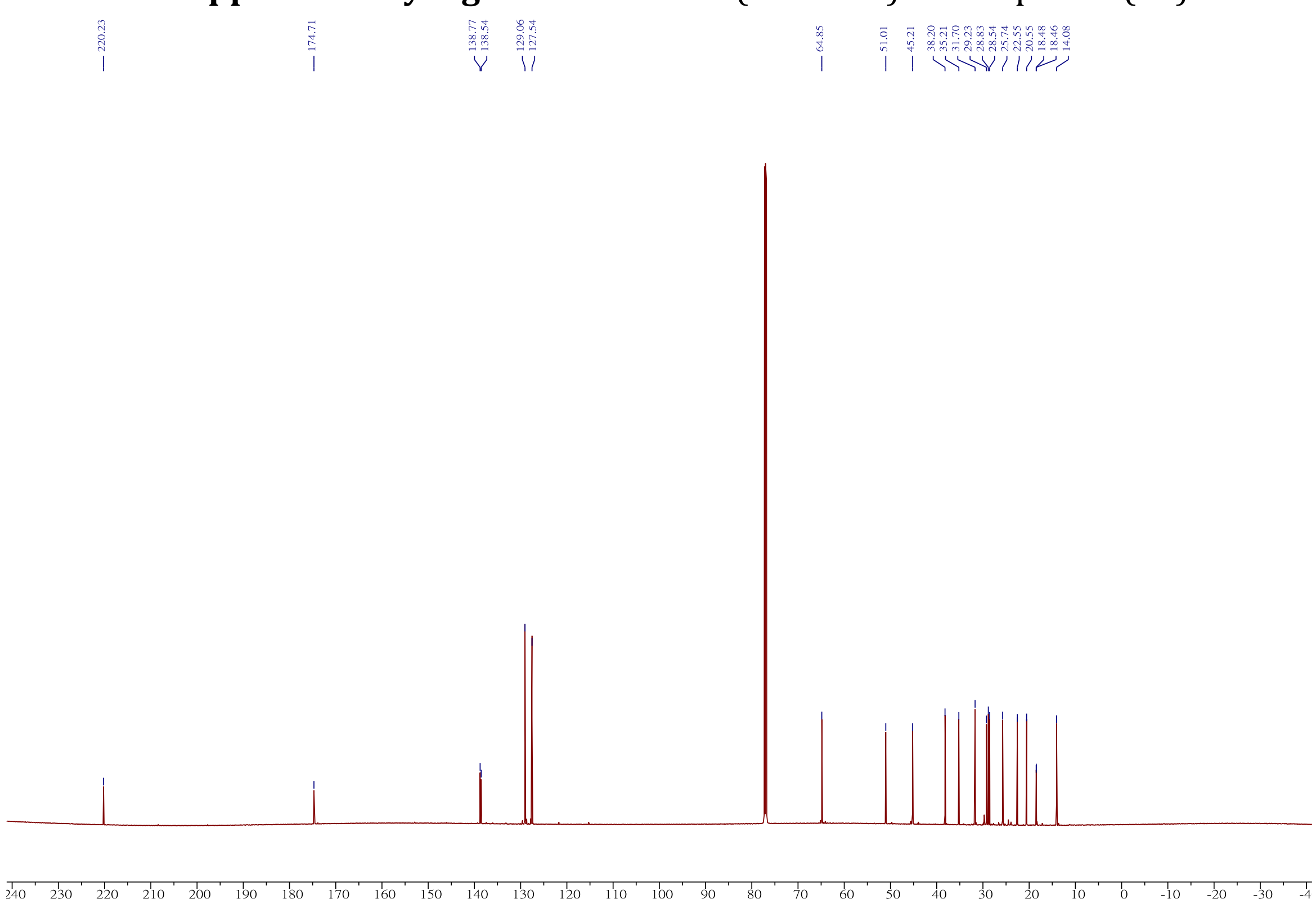

Supplementary Figure 80. ^{13}C NMR (151 MHz) of Compound (30)

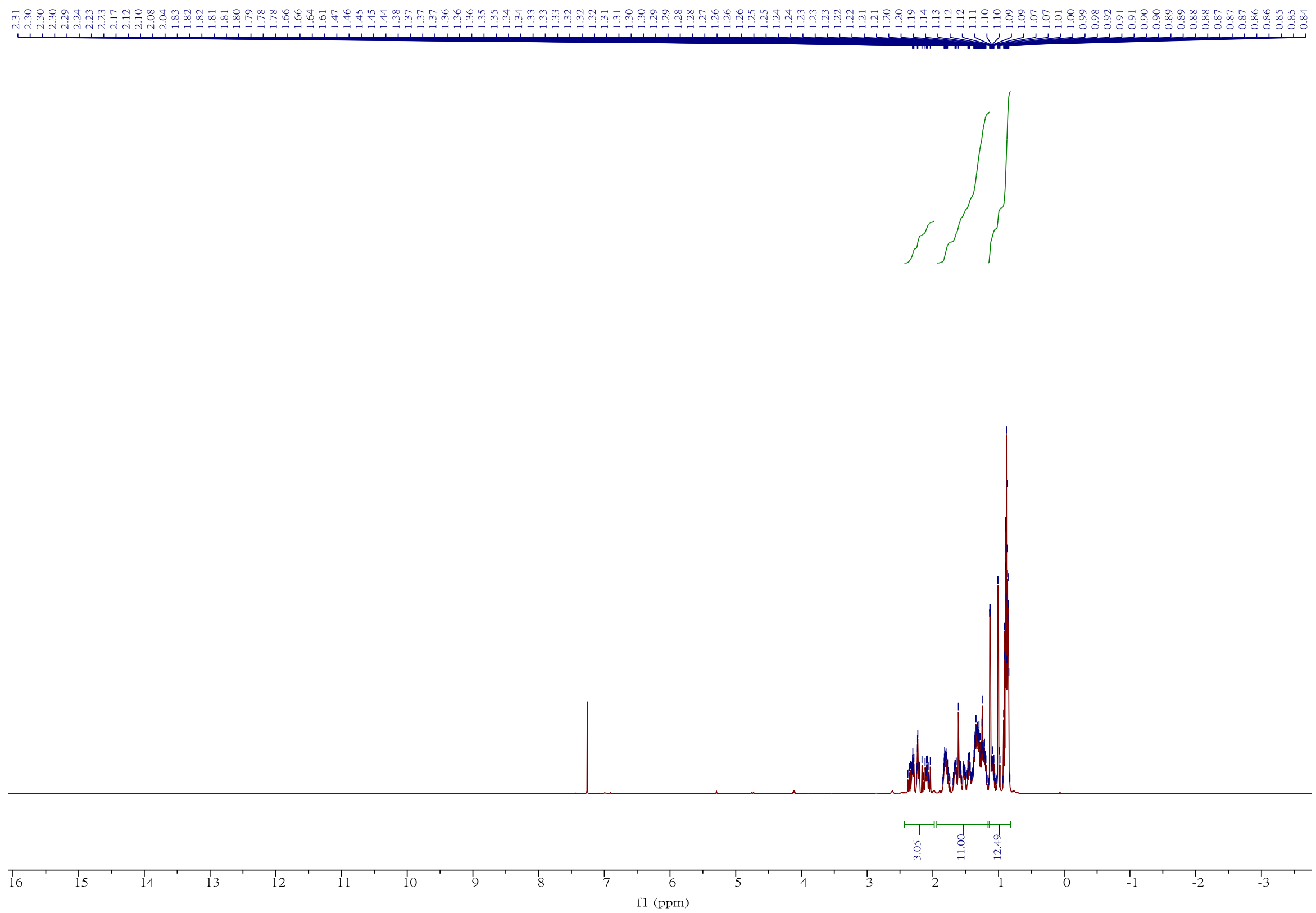

Supplementary Figure 81. ^{1}H NMR (600 MHz) of Compound (31)

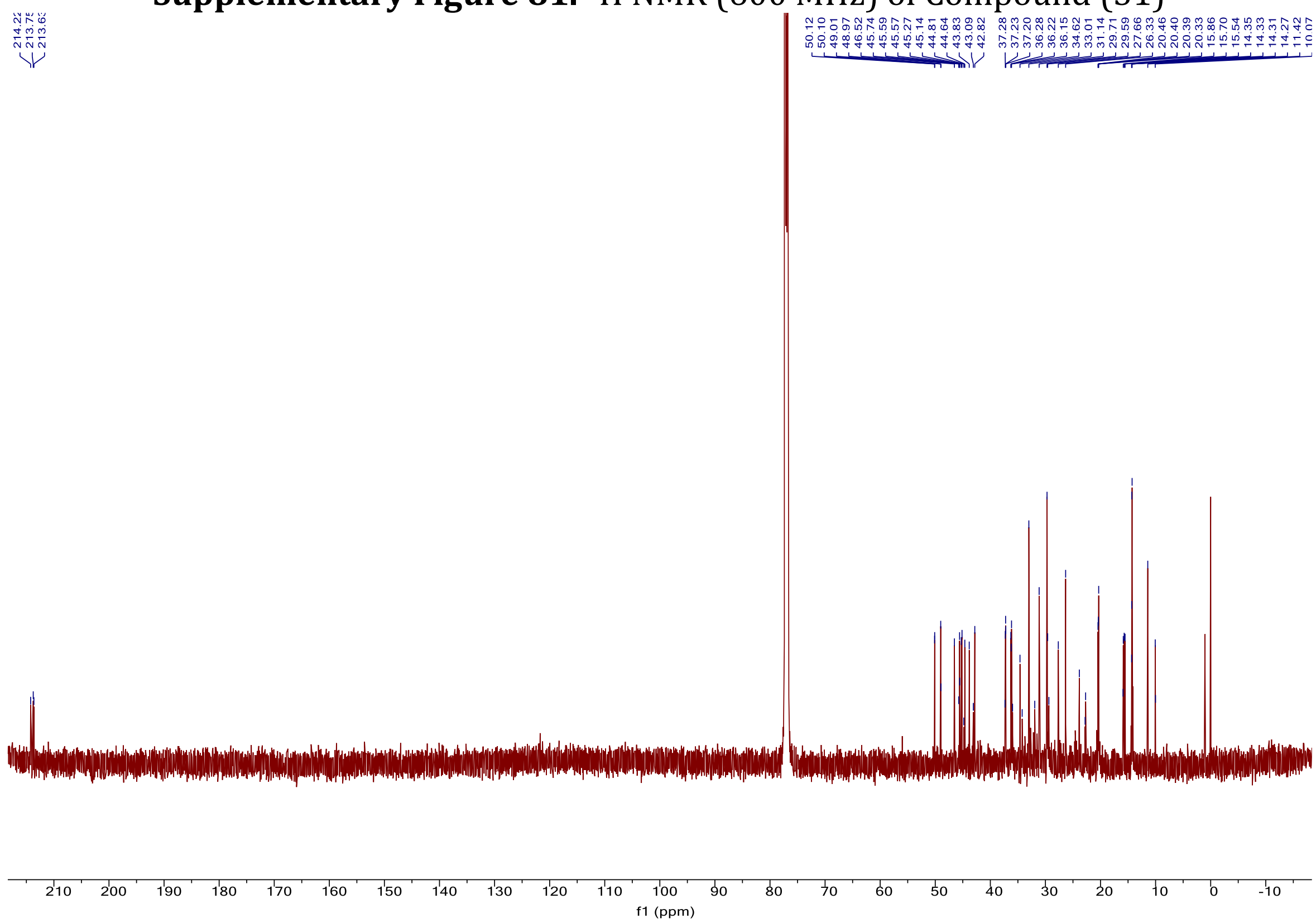

Supplementary Figure 82. ^{13}C NMR (151 MHz) of Compound (31)

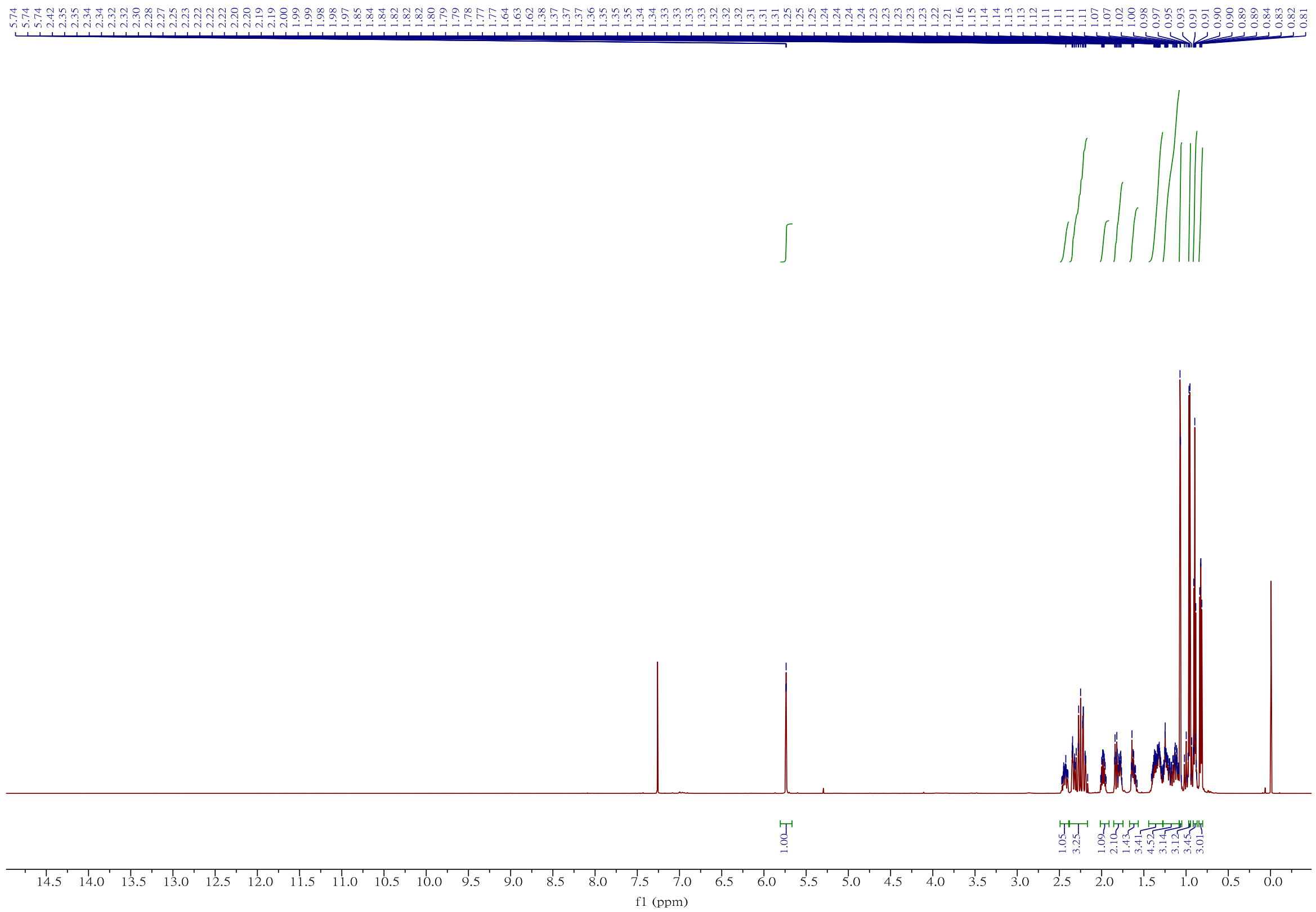

Supplementary Figure 83. ^{1}H NMR (600 MHz) of Compound (32)

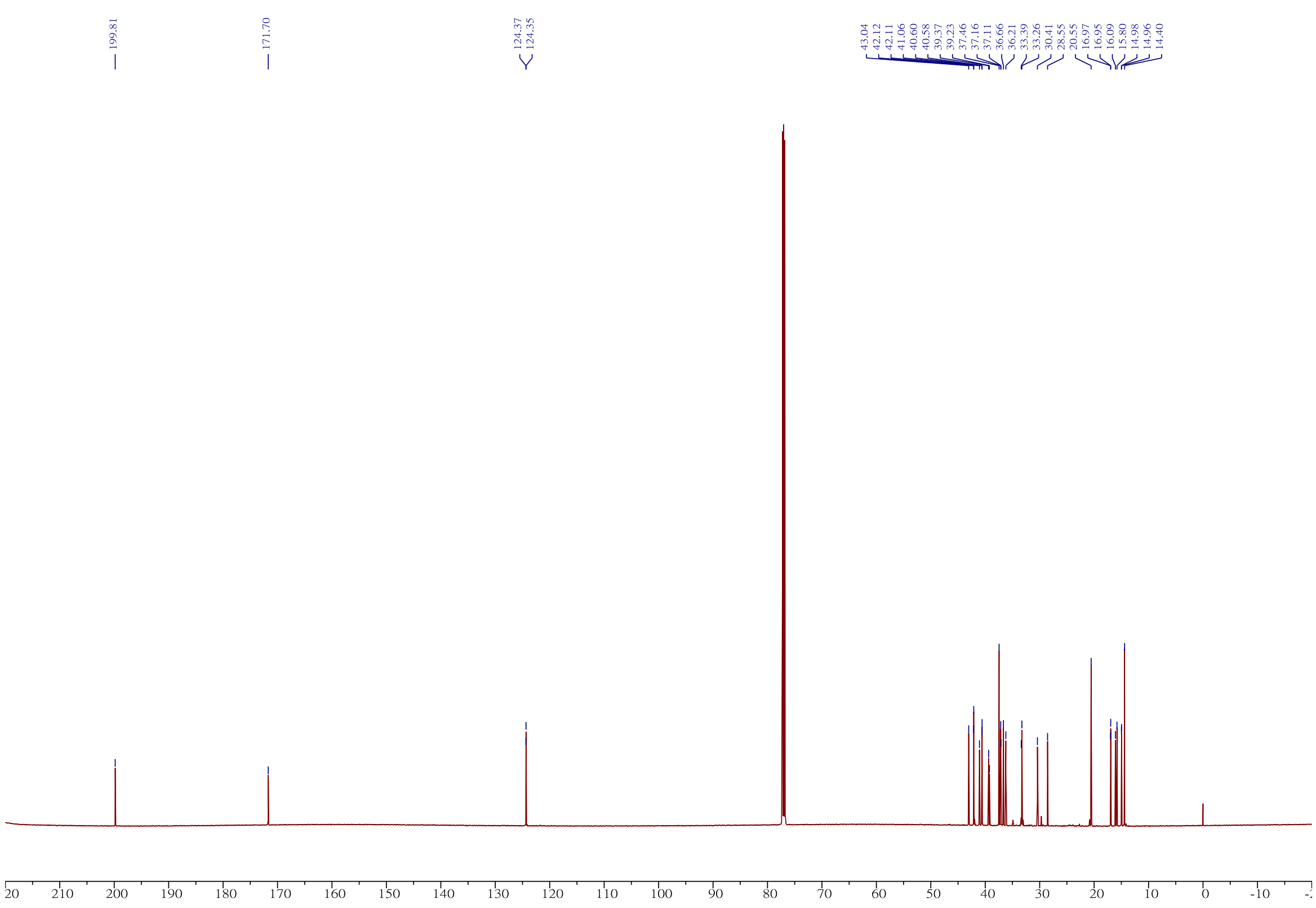

Supplementary Figure 84. ^{13}C NMR (151 MHz) of Compound (32)

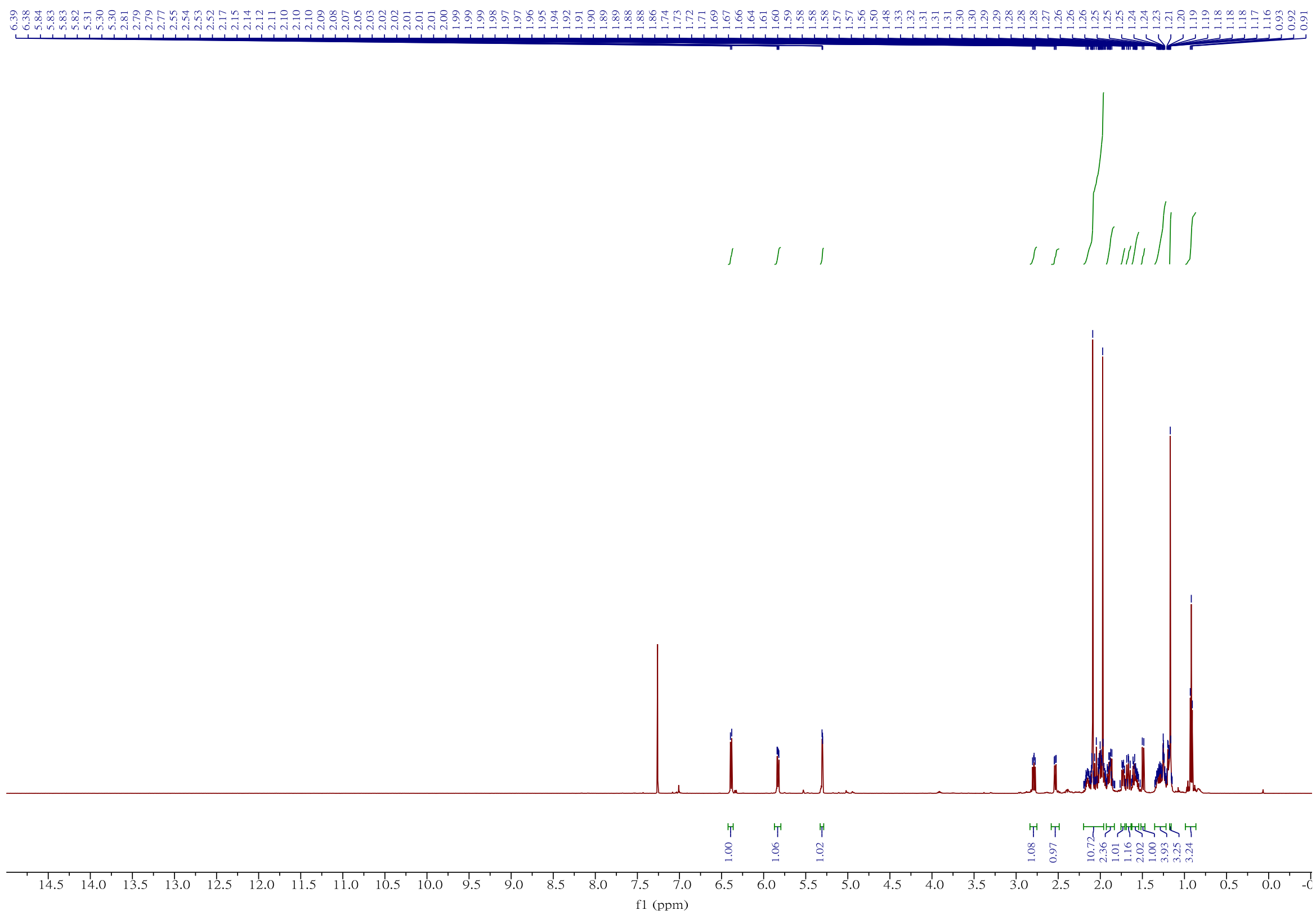

Supplementary Figure 85. ^{1}H NMR (600 MHz) of Compound (33)

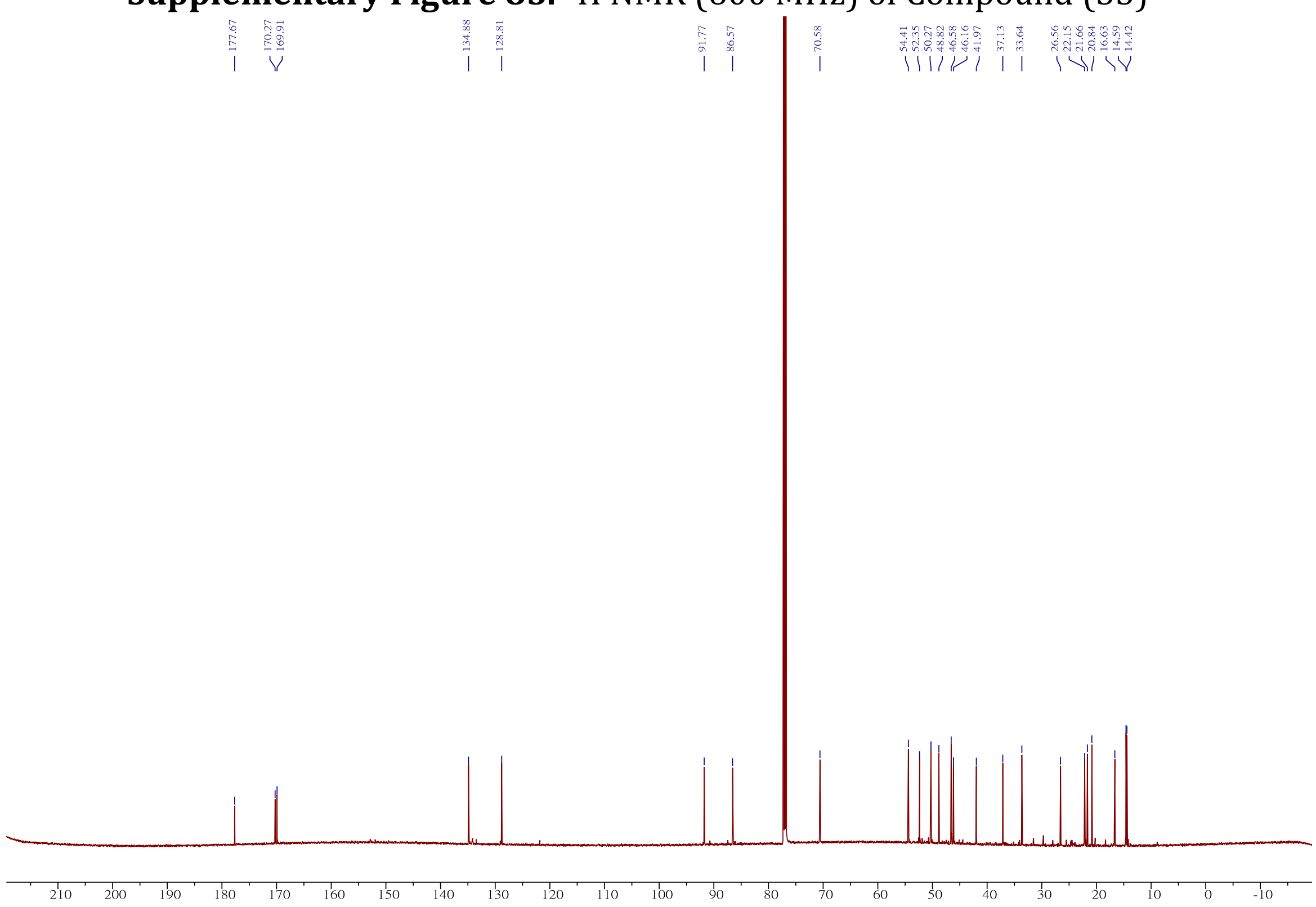

Supplementary Figure 86. ^{13}C NMR (151 MHz) of Compound (33)

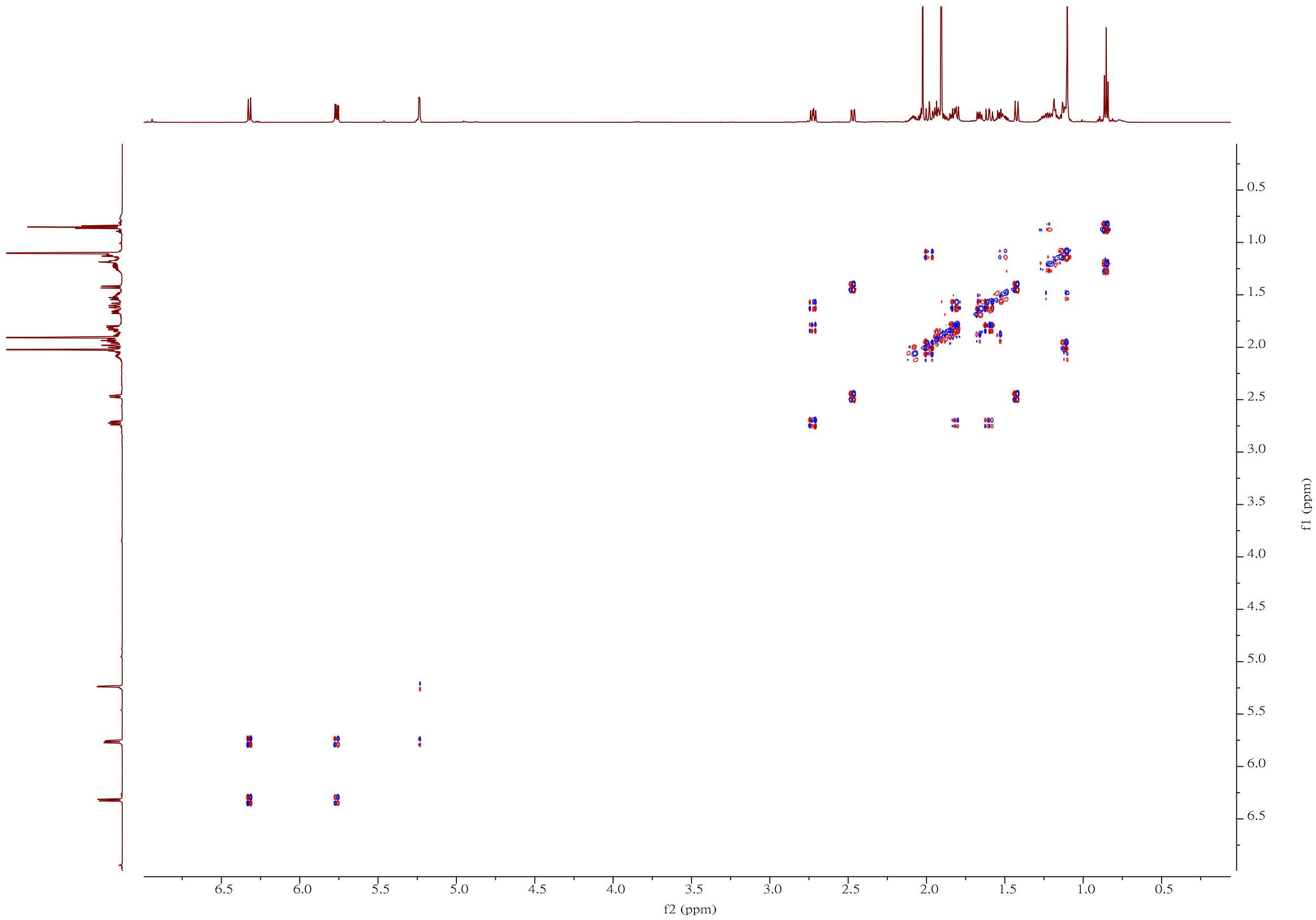

Supplementary Figure 87. ^{1}H–^{1}H NMR (600 MHz: $CDCl_3$) COSY of Compound (33)

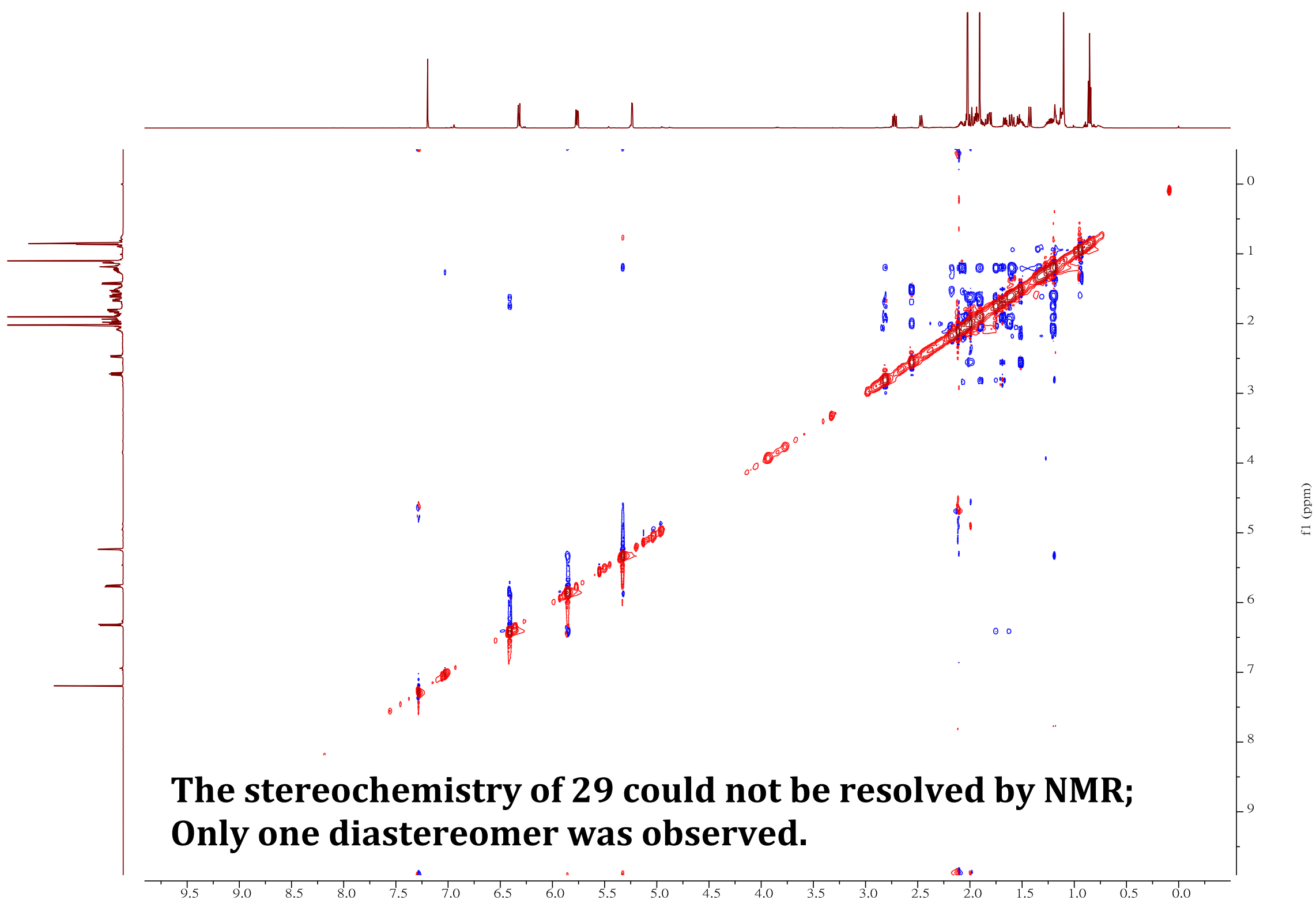

Supplementary Figure 88. ^{1}H–^{1}H NMR (600 MHz: $CDCl_3$) NOESY of Compound (33)

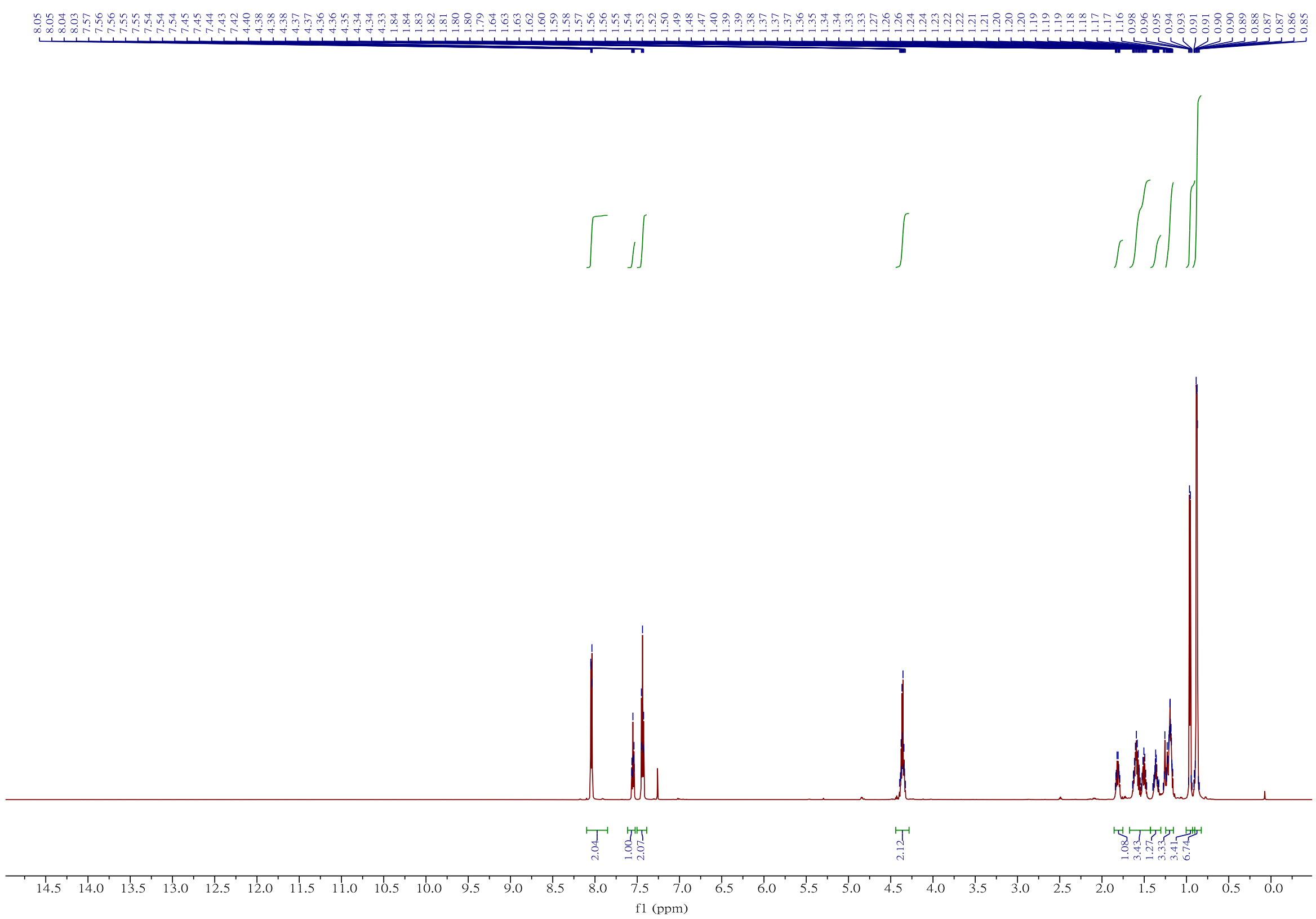

Supplementary Figure 89. ^{1}H NMR (600 MHz) of Compound (34)

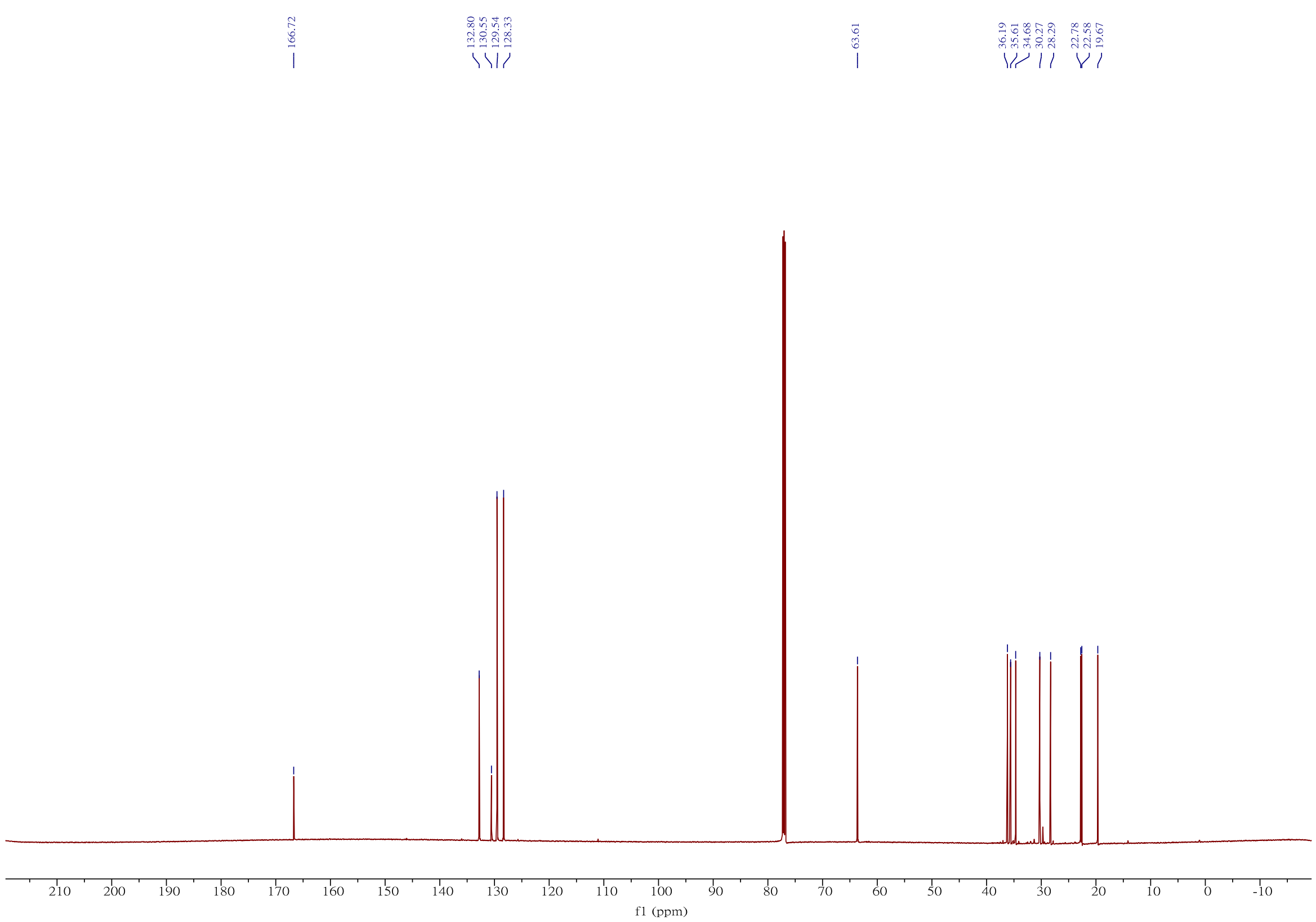

Supplementary Figure 90. ^{13}C NMR (151 MHz) of Compound (34)

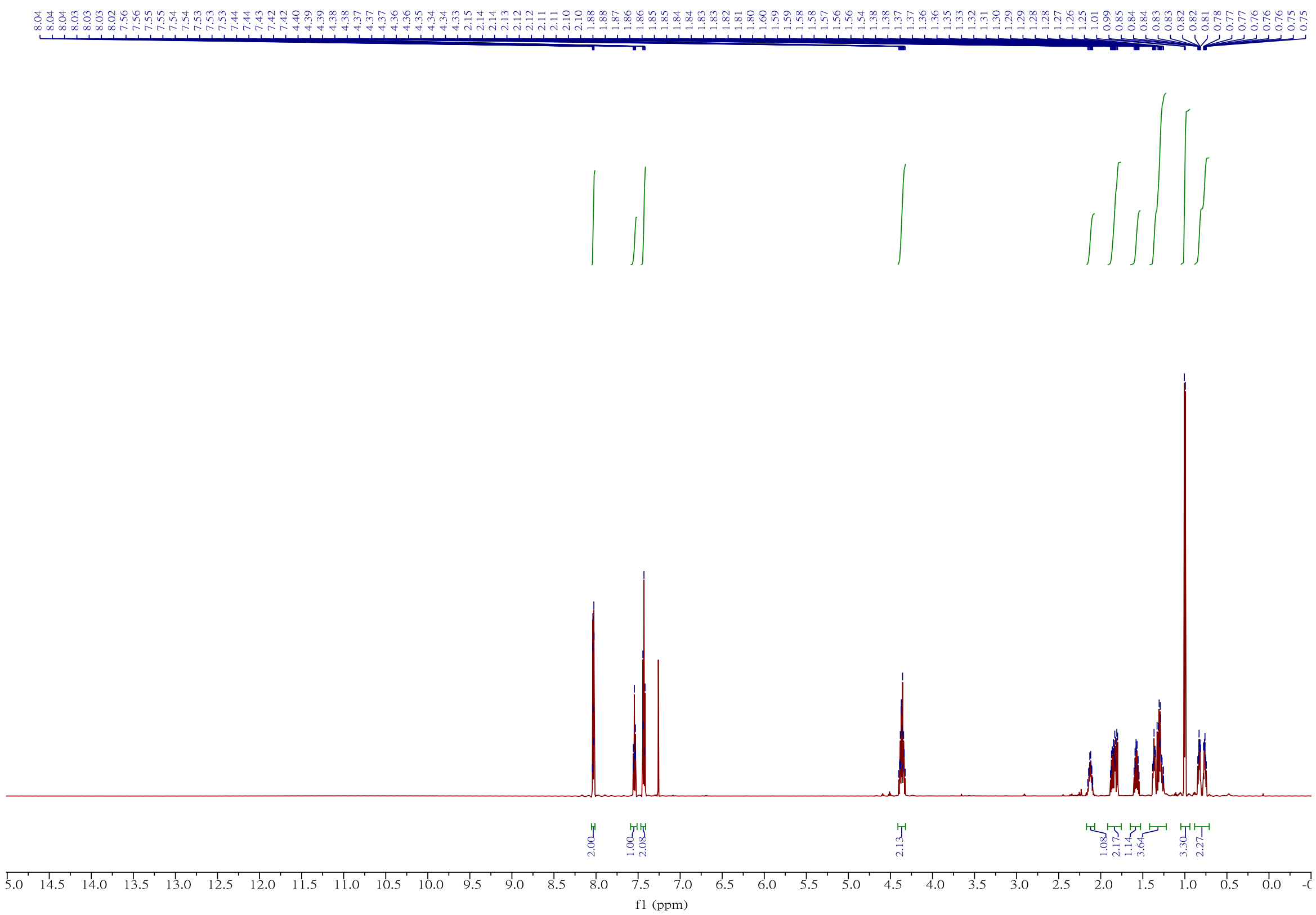

Supplementary Figure 91. ^{1}H NMR (600 MHz) of Compound (35)

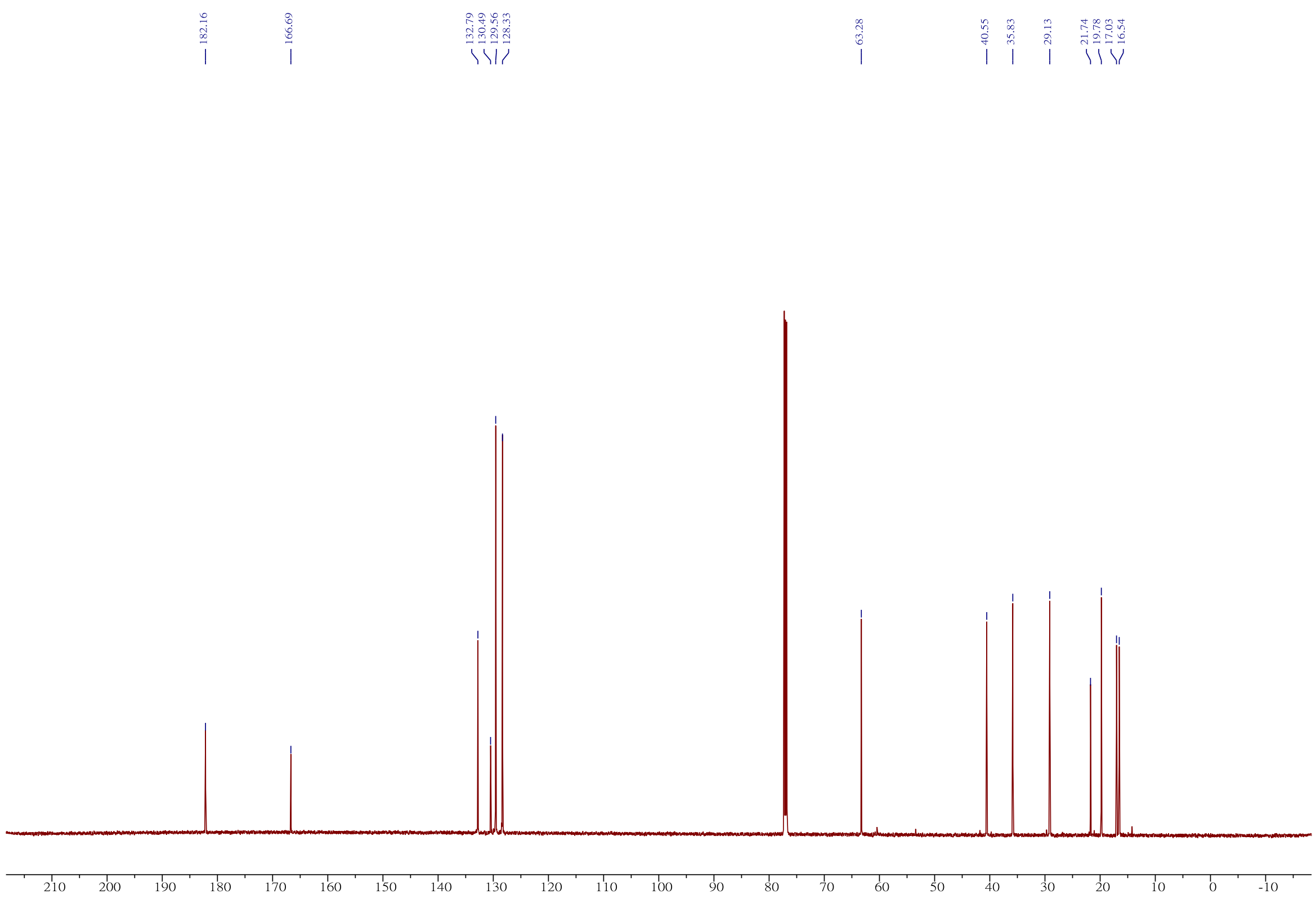

Supplementary Figure 92. ^{13}C NMR (151 MHz) of Compound (35)

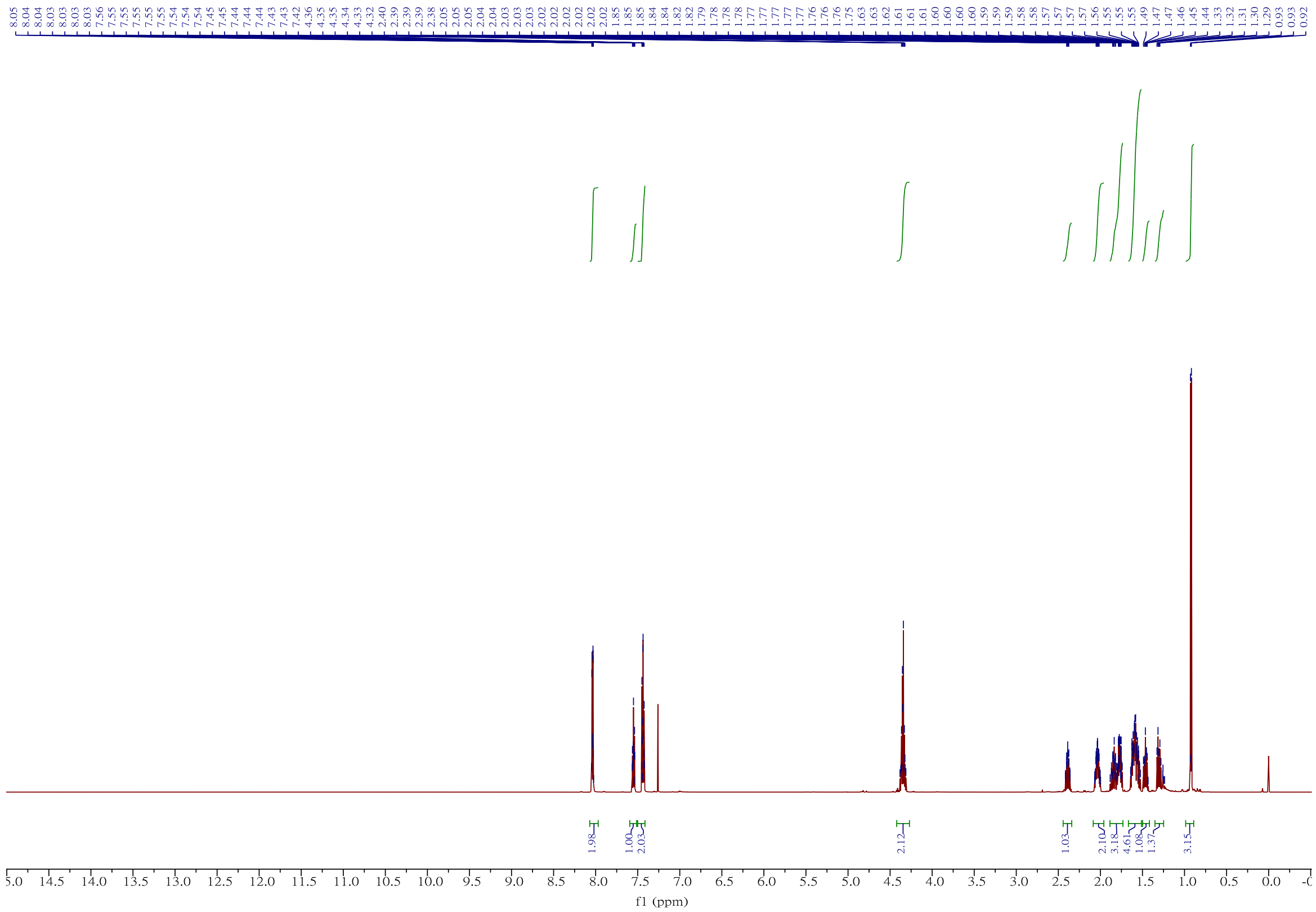

Supplementary Figure 93. ^{1}H NMR (600 MHz) of Compound (36)

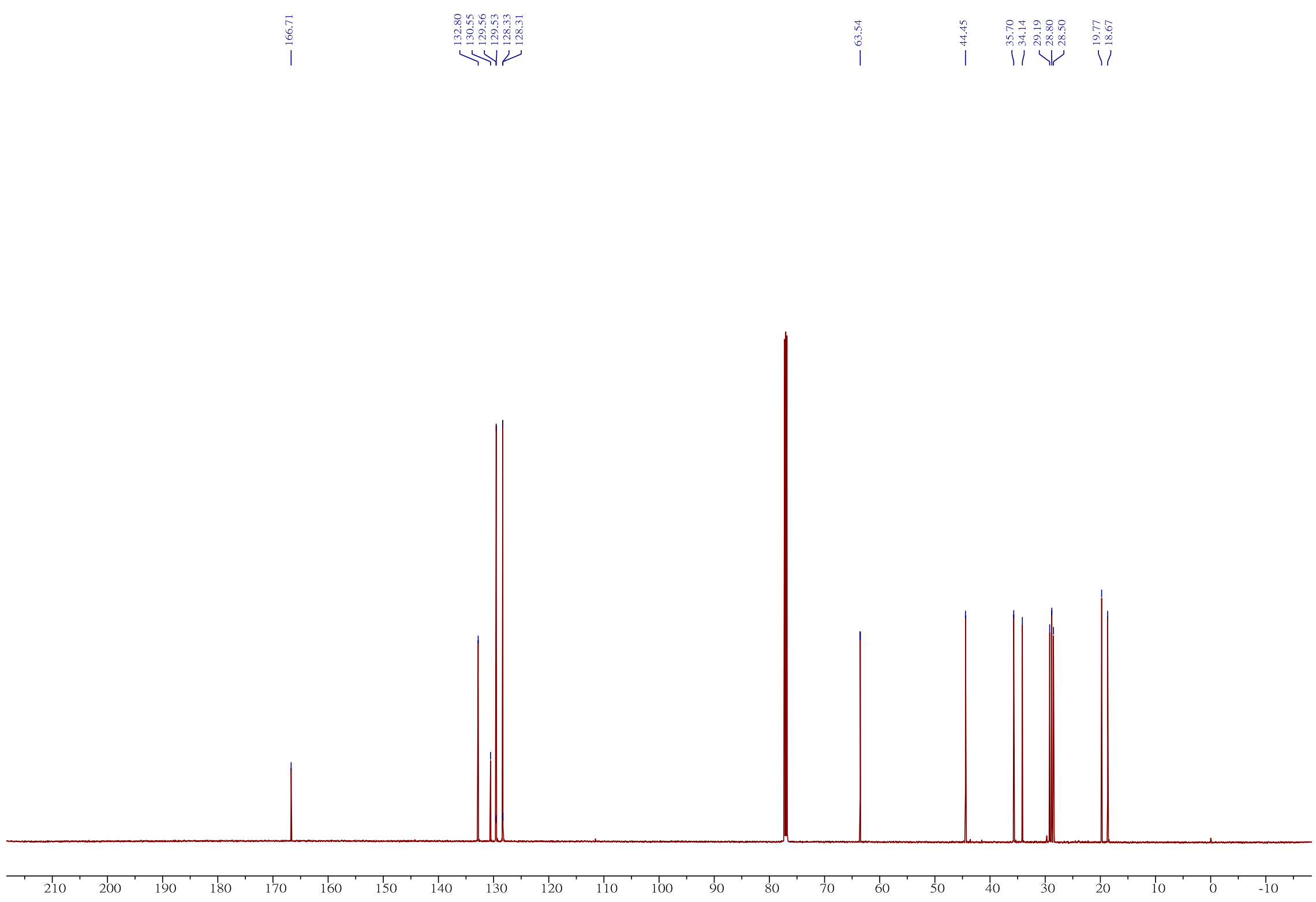

Supplementary Figure 94. ^{13}C NMR (151 MHz) of Compound (36)

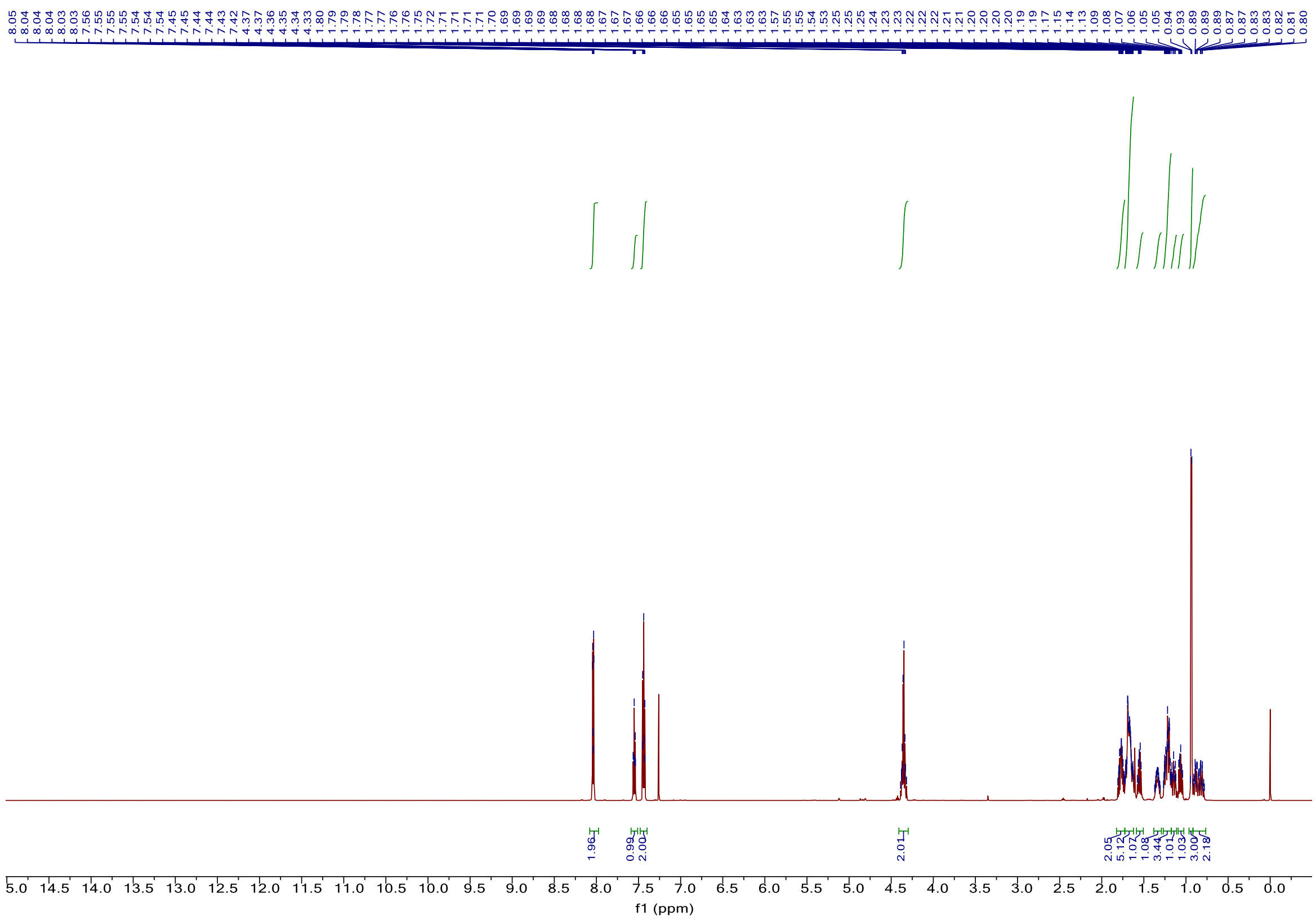

Supplementary Figure 95. ^{1}H NMR (600 MHz) of Compound (37)

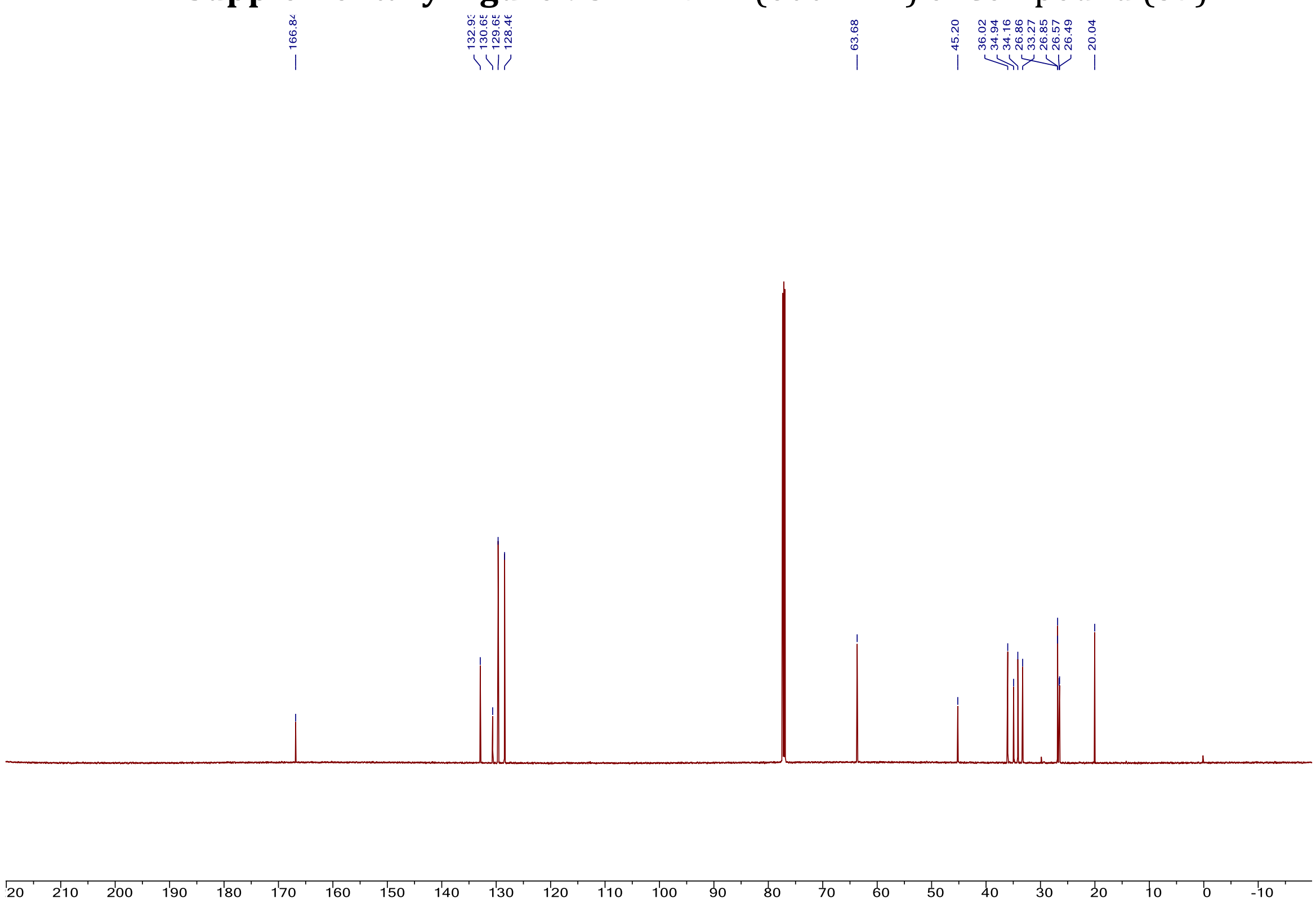

Supplementary Figure 96. ^{13}C NMR (151 MHz) of Compound (37)

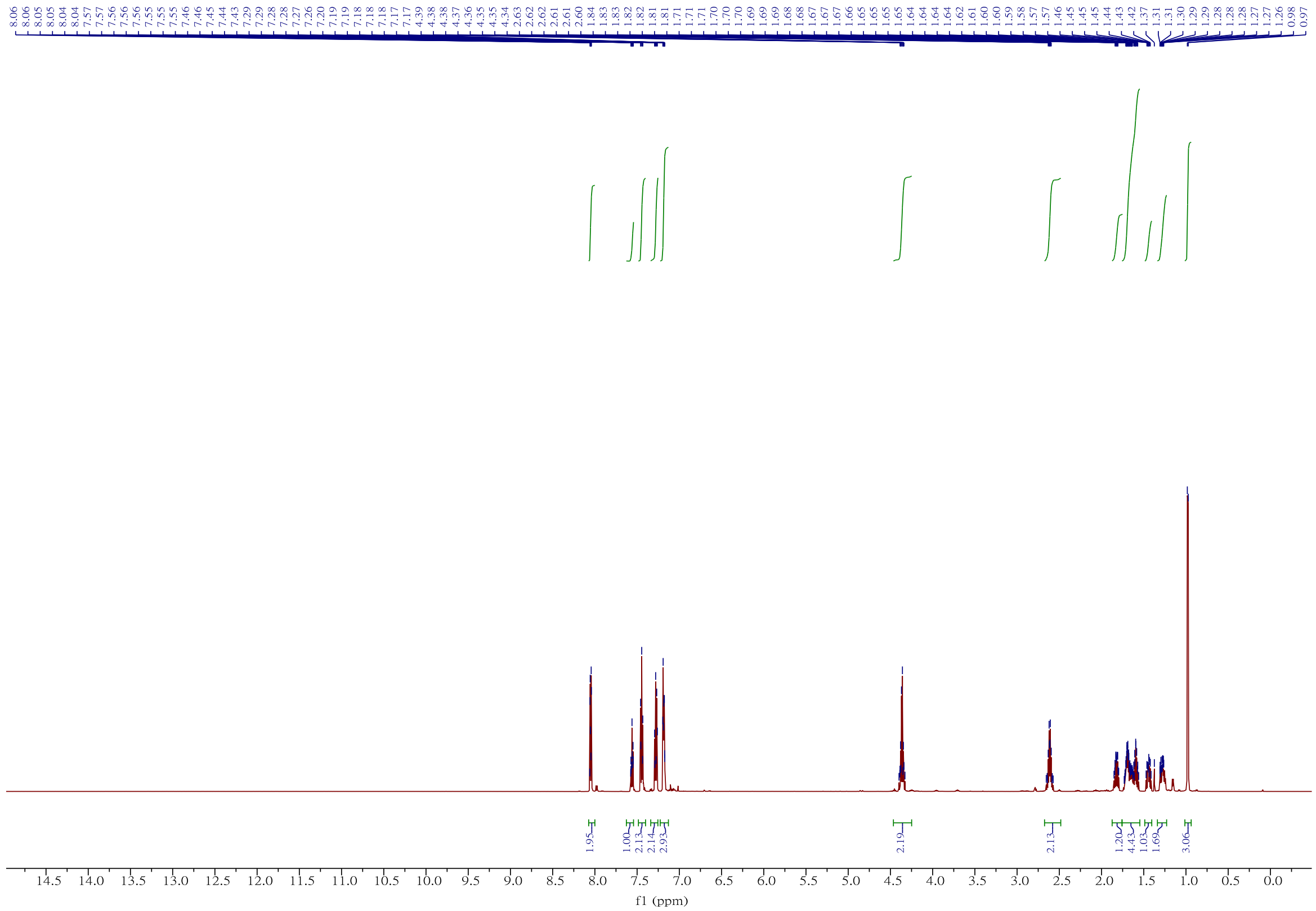

Supplementary Figure 97. ^{1}H NMR (600 MHz) of Compound (38)

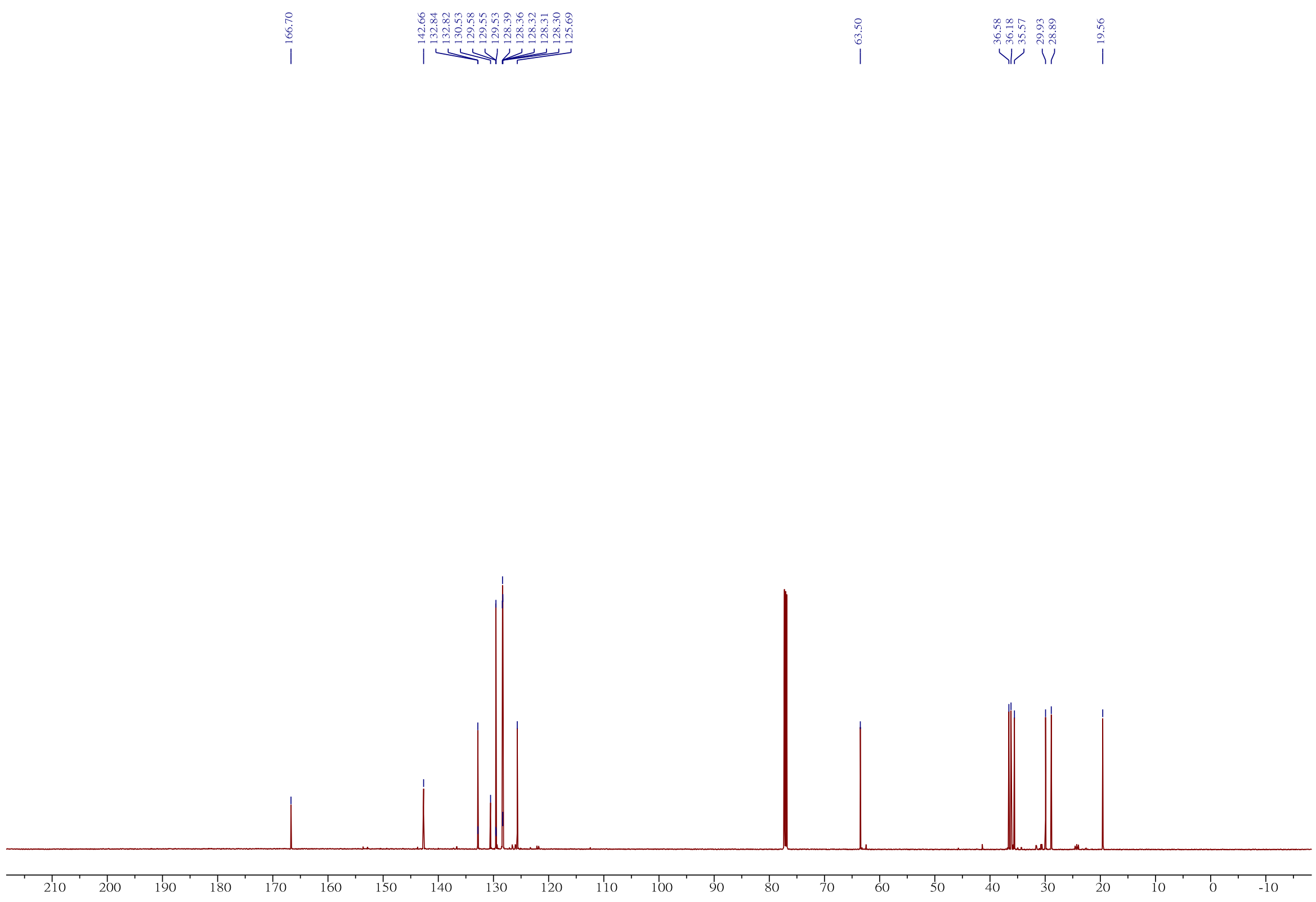

Supplementary Figure 98. ^{13}C NMR (151 MHz) of Compound (38)

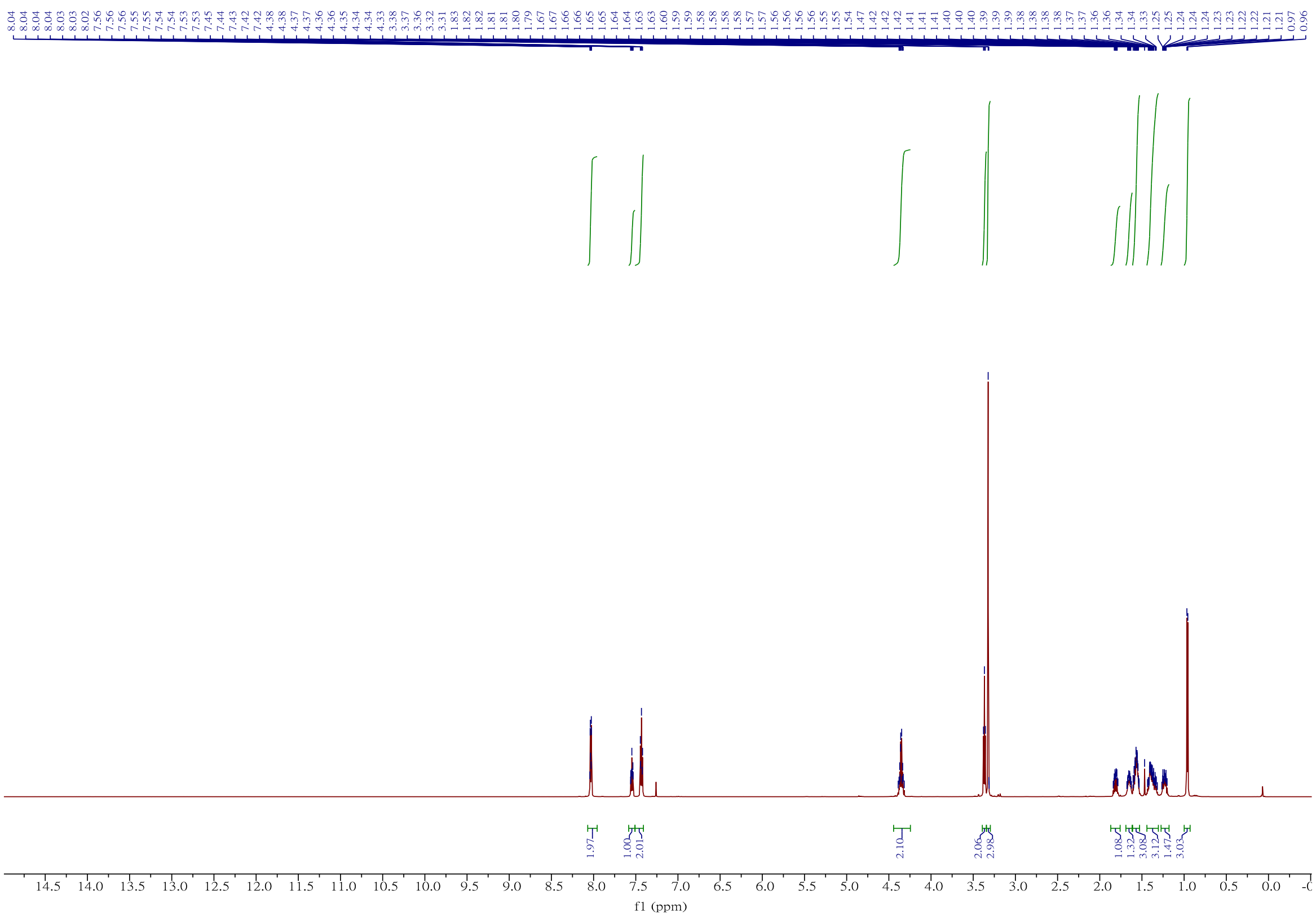

Supplementary Figure 99. ^{1}H NMR (600 MHz) of Compound (39)

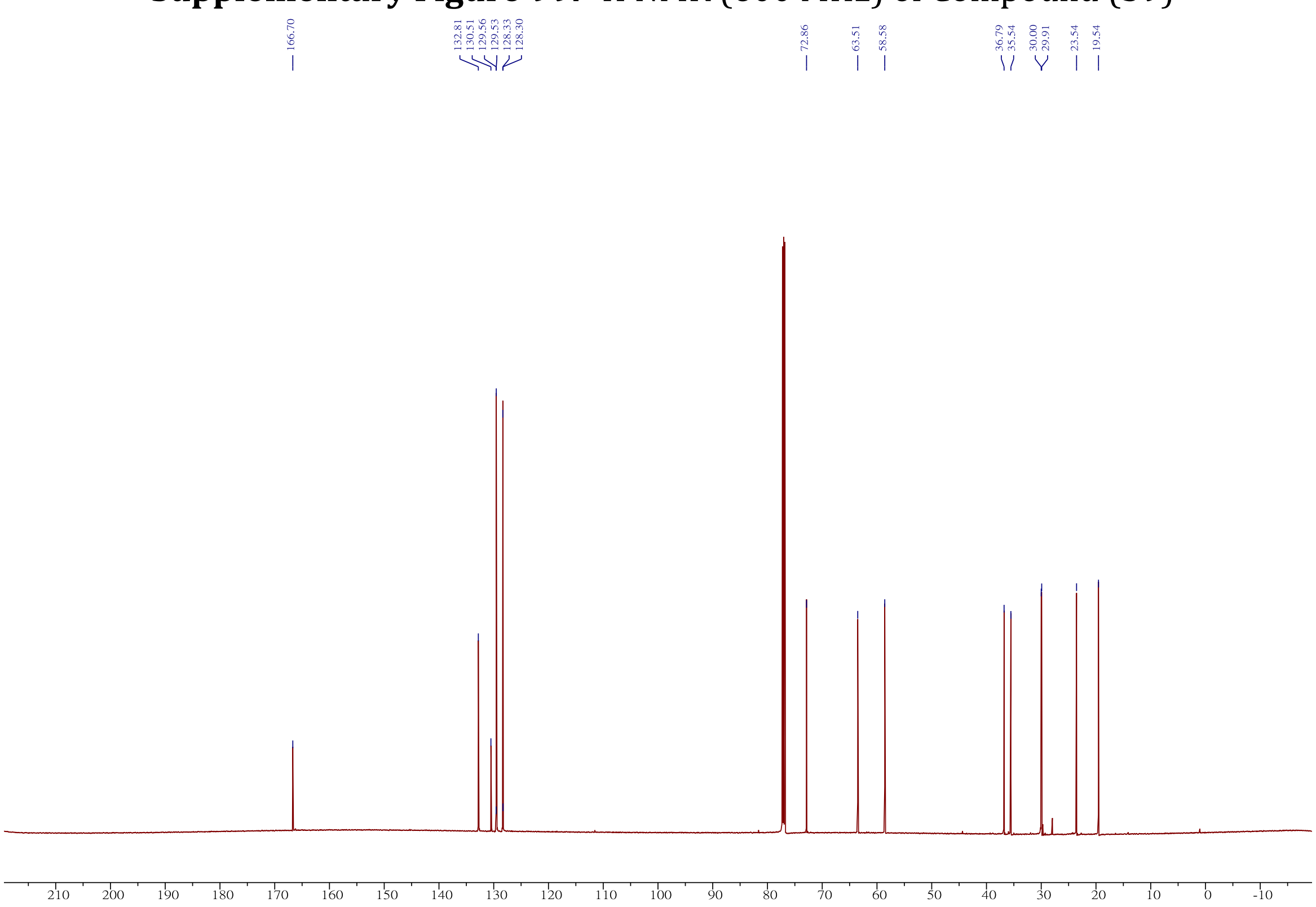

Supplementary Figure 100. ^{13}C NMR (151 MHz) of Compound (39)

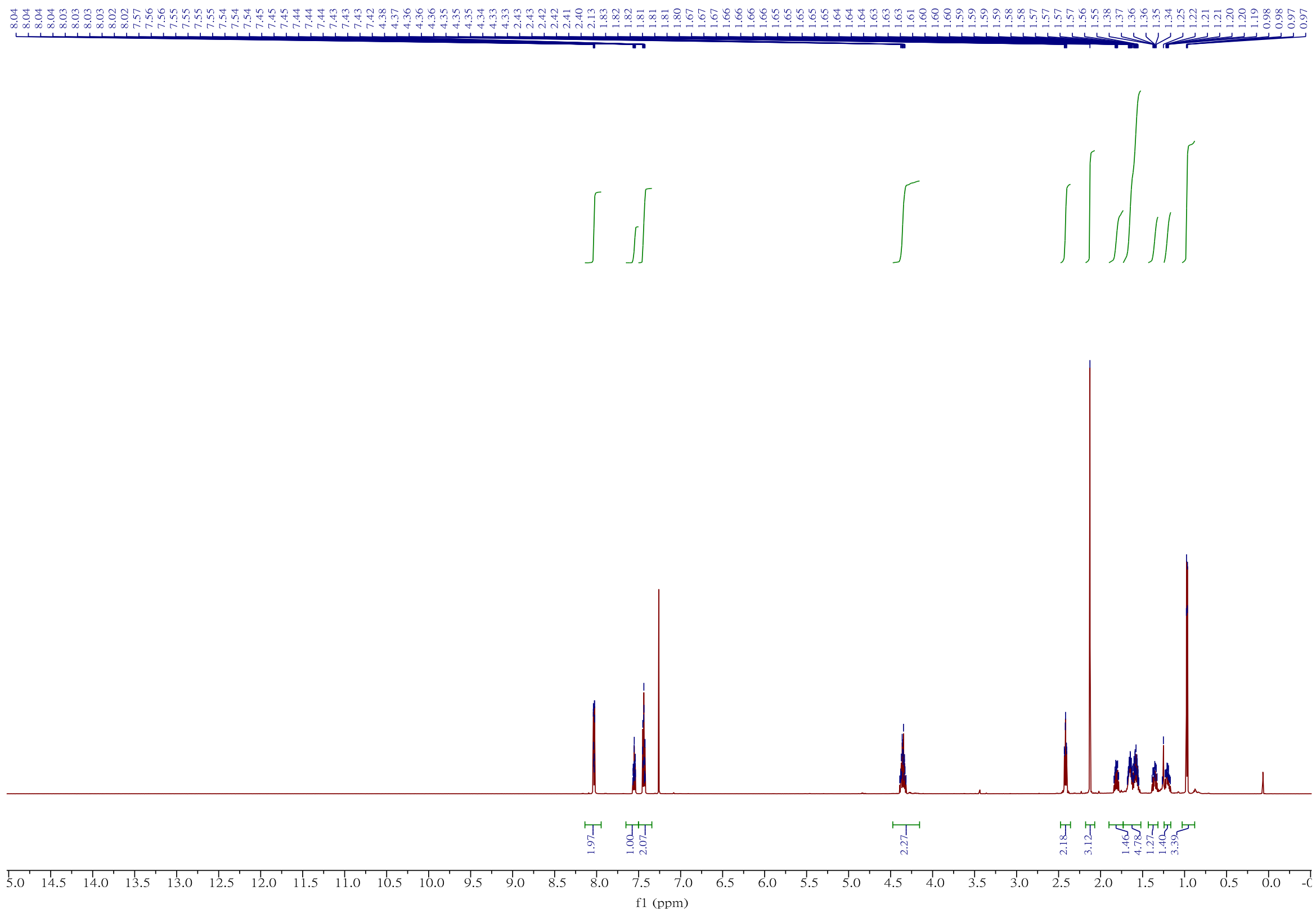

Supplementary Figure 101. ^{1}H NMR (600 MHz) of Compound (40)

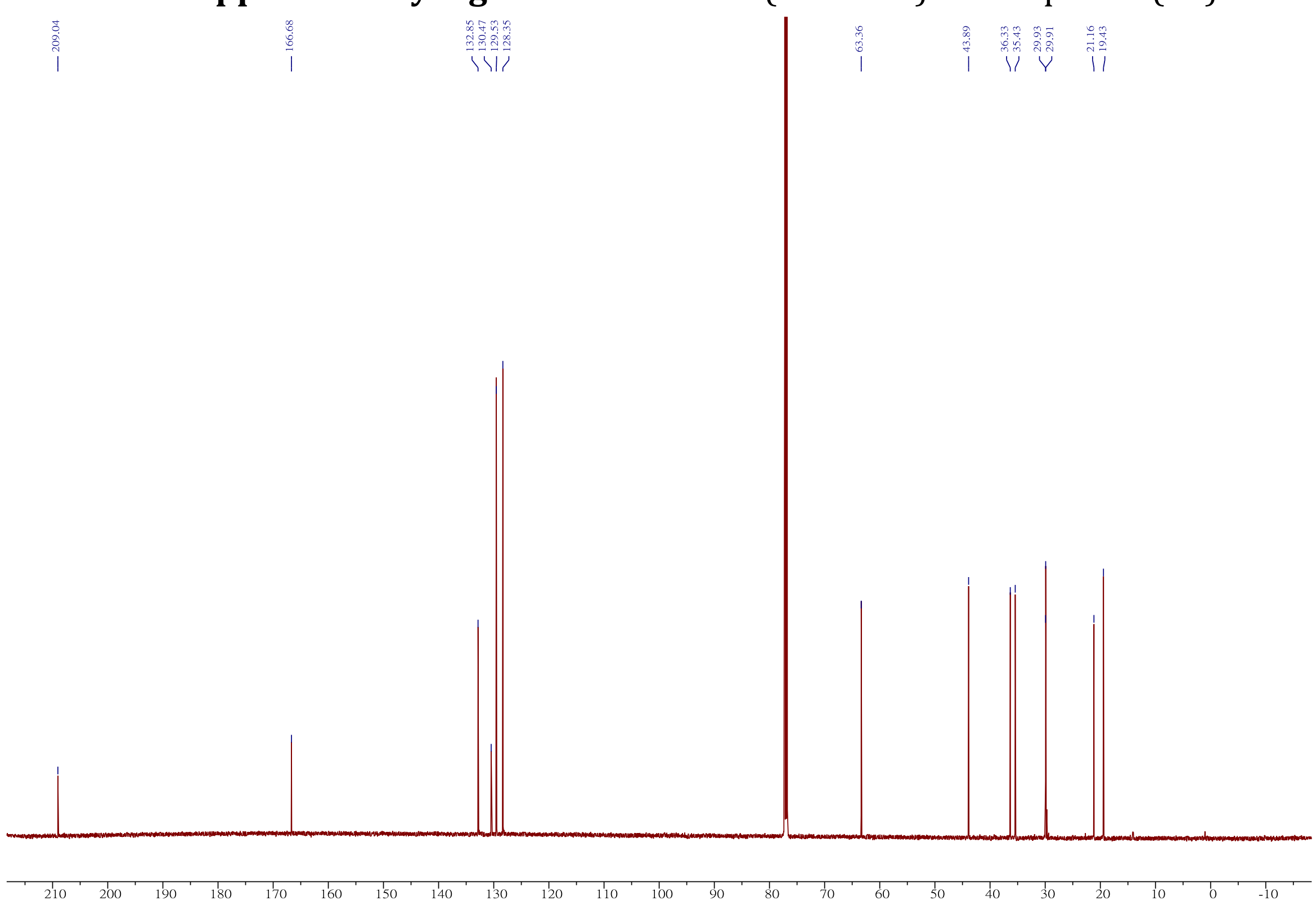

Supplementary Figure 102. ^{13}C NMR (151 MHz) of Compound (40)

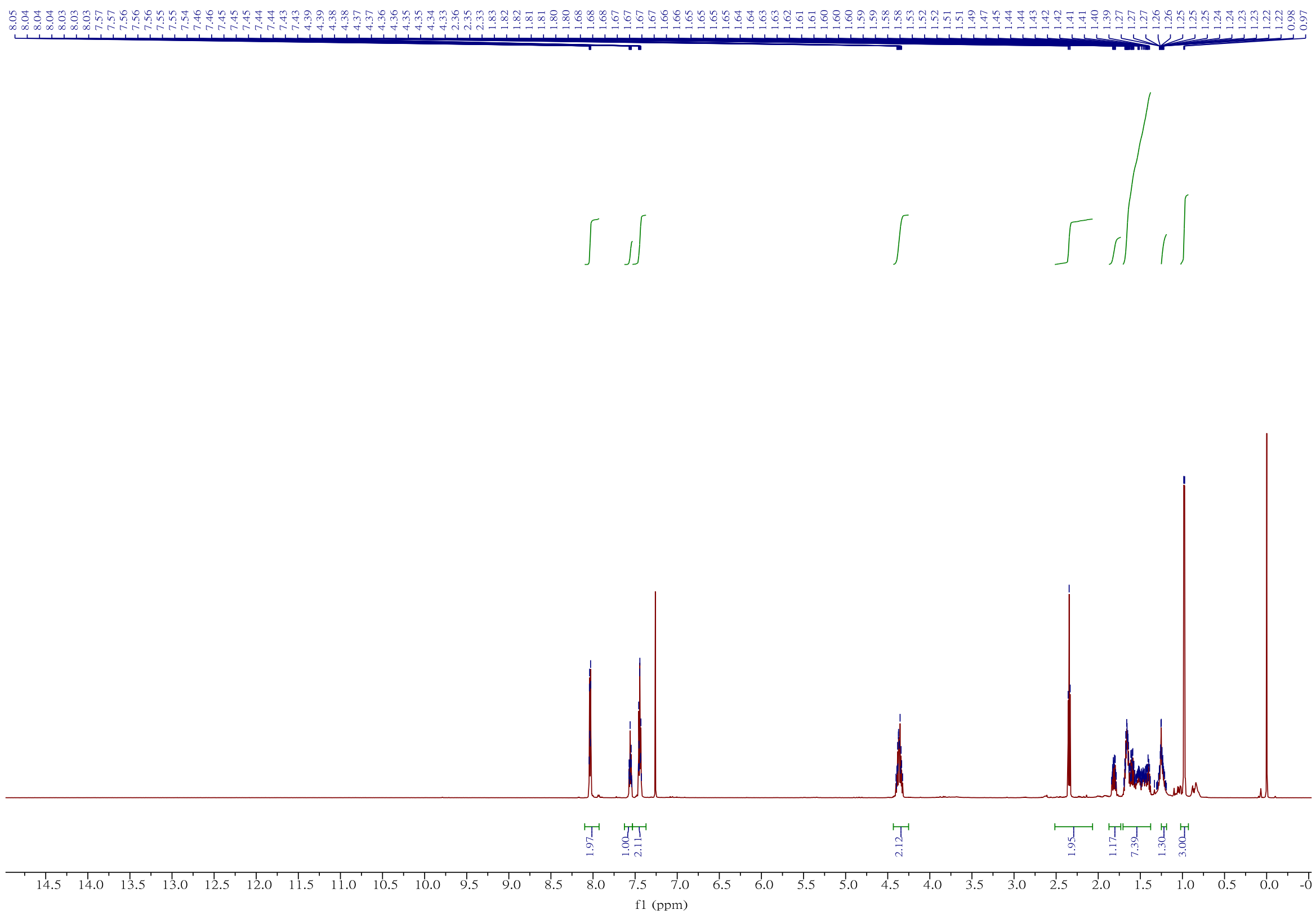

Supplementary Figure 103. ^{1}H NMR (600 MHz) of Compound (41)

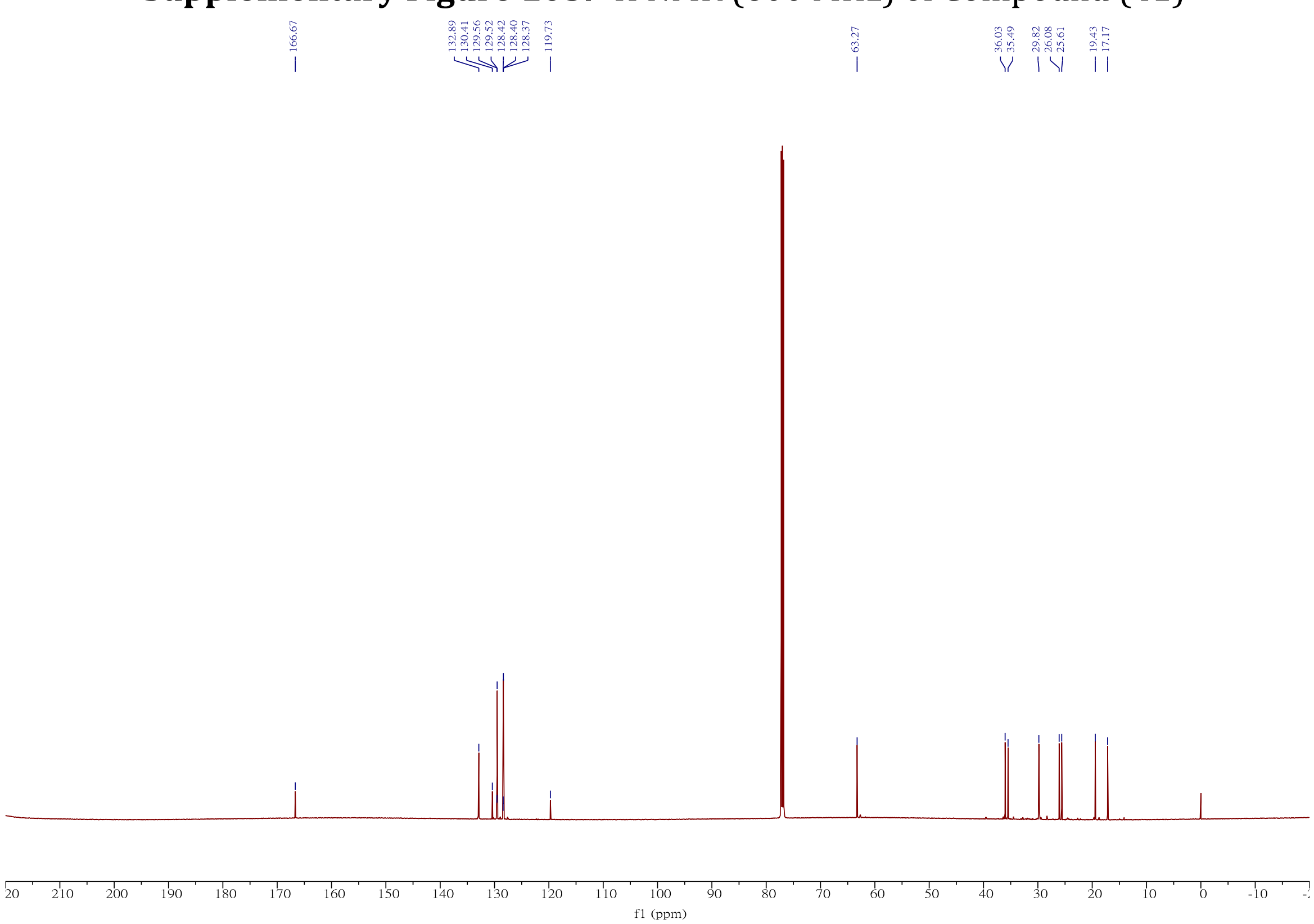

Supplementary Figure 104. ^{13}C NMR (151 MHz) of Compound (41)

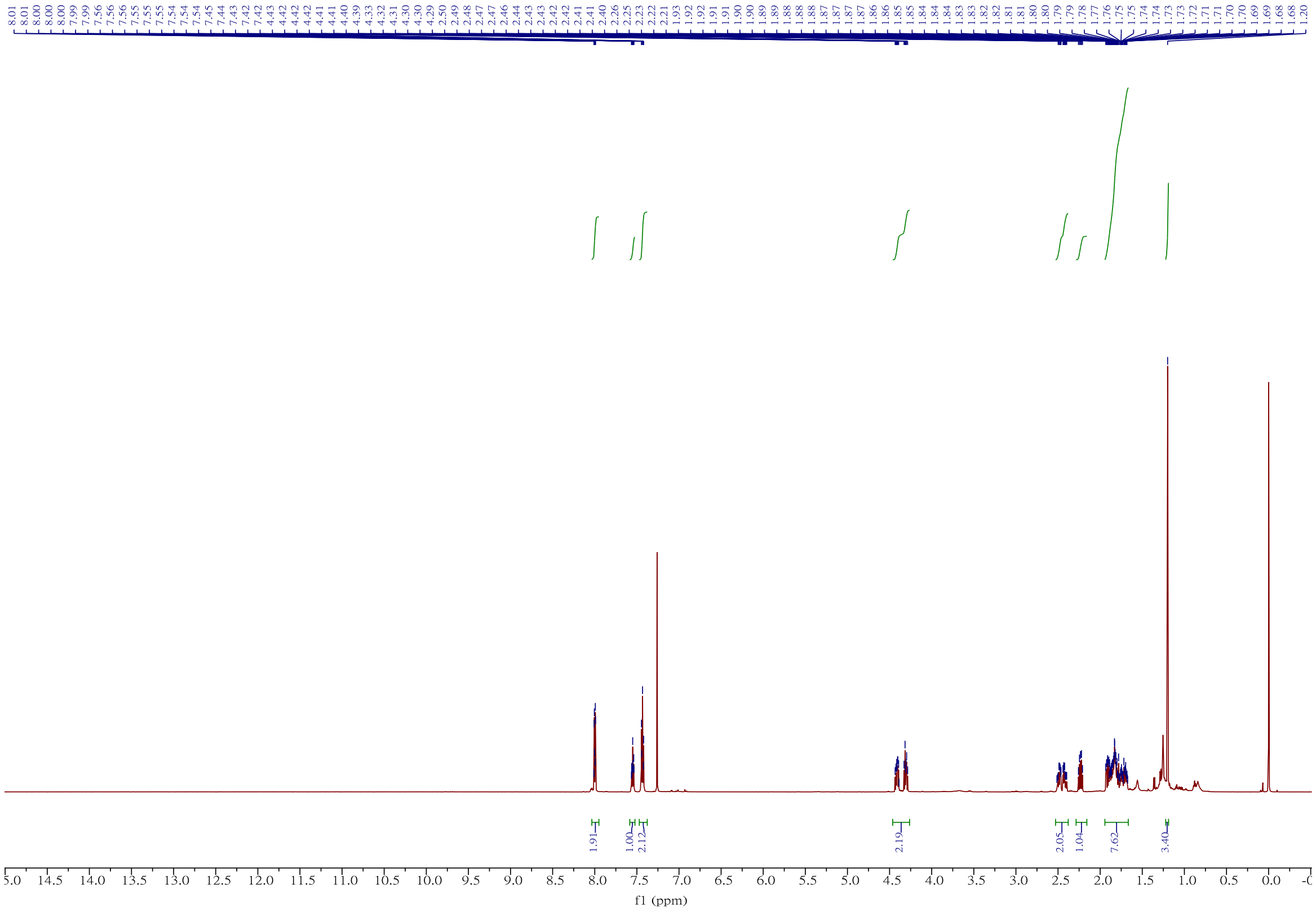

Supplementary Figure 105. ^{1}H NMR (600 MHz) of Compound (41')

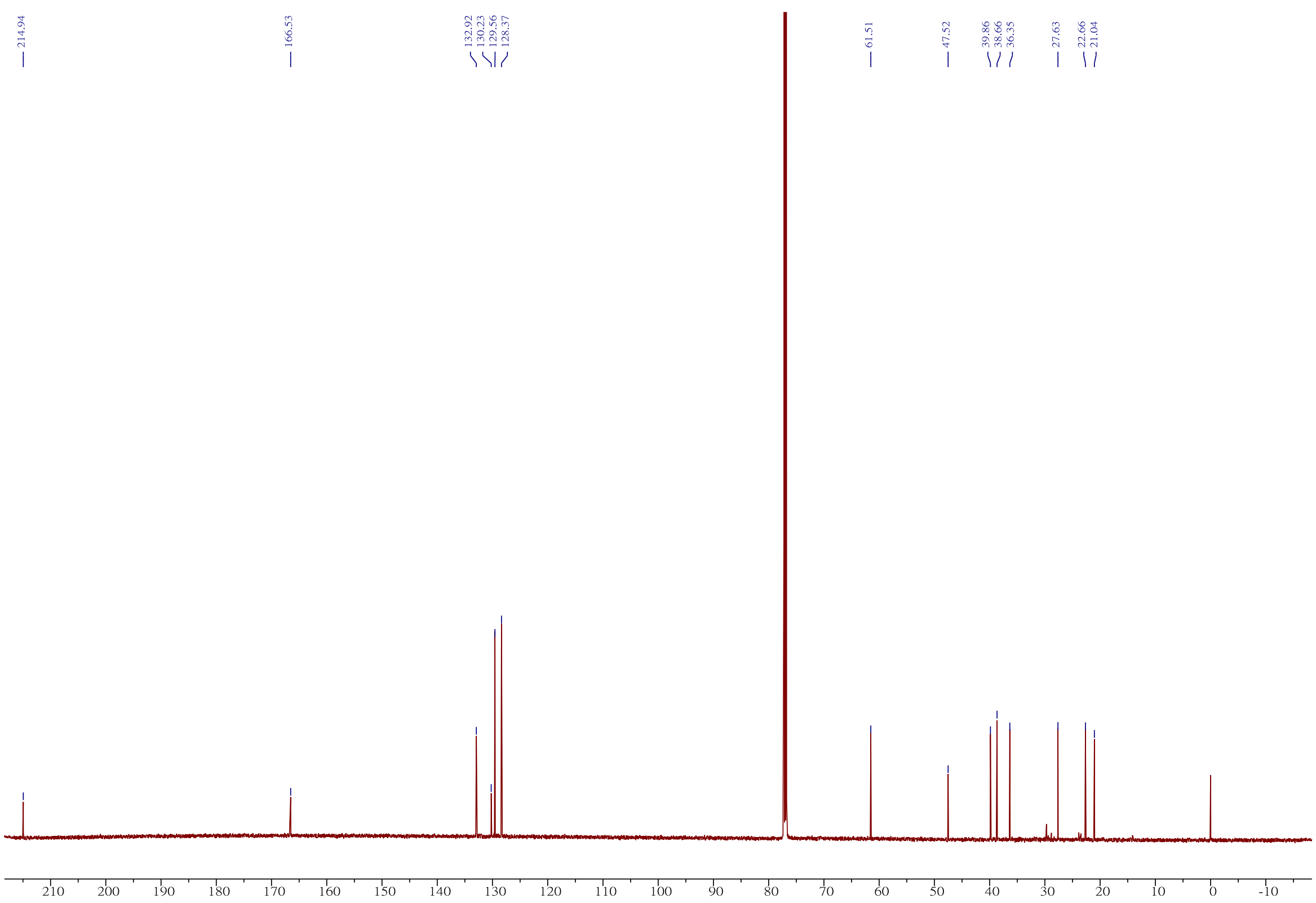

Supplementary Figure 106. ^{13}C NMR (151 MHz) of Compound (41')

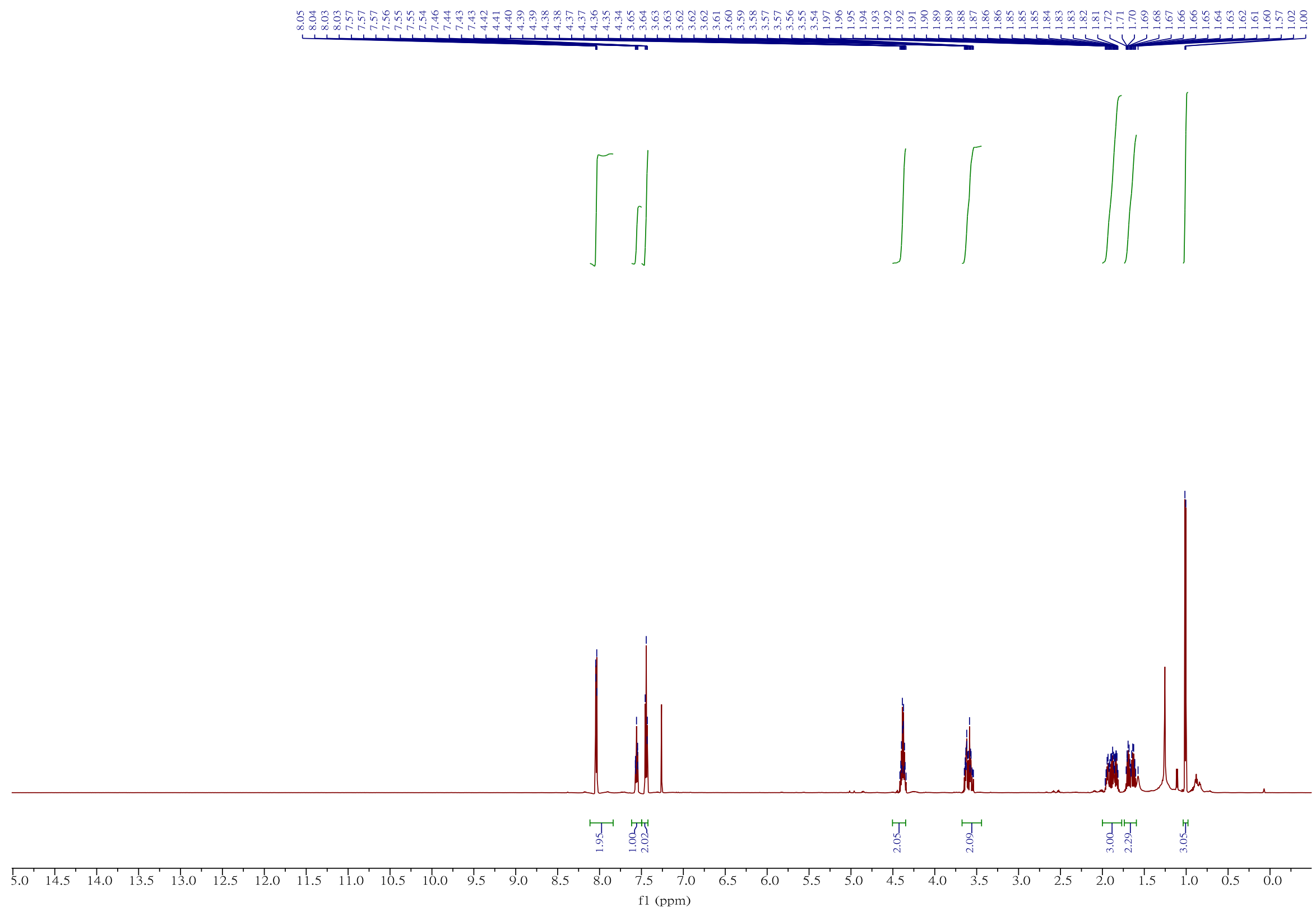

Supplementary Figure 107. ^{1}H NMR (600 MHz) of Compound (42)

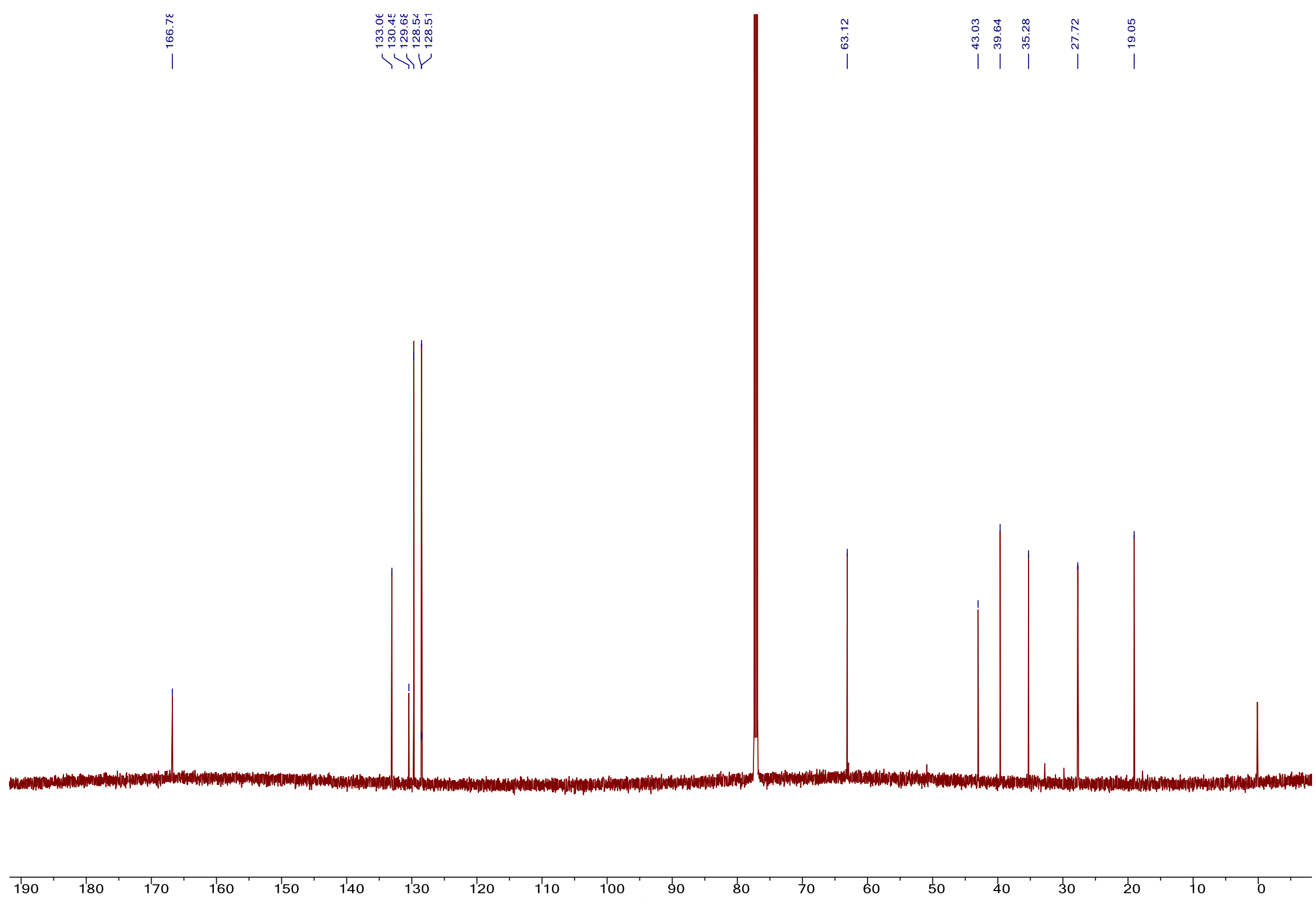

Supplementary Figure 108. ^{13}C NMR (151 MHz) of Compound (42)

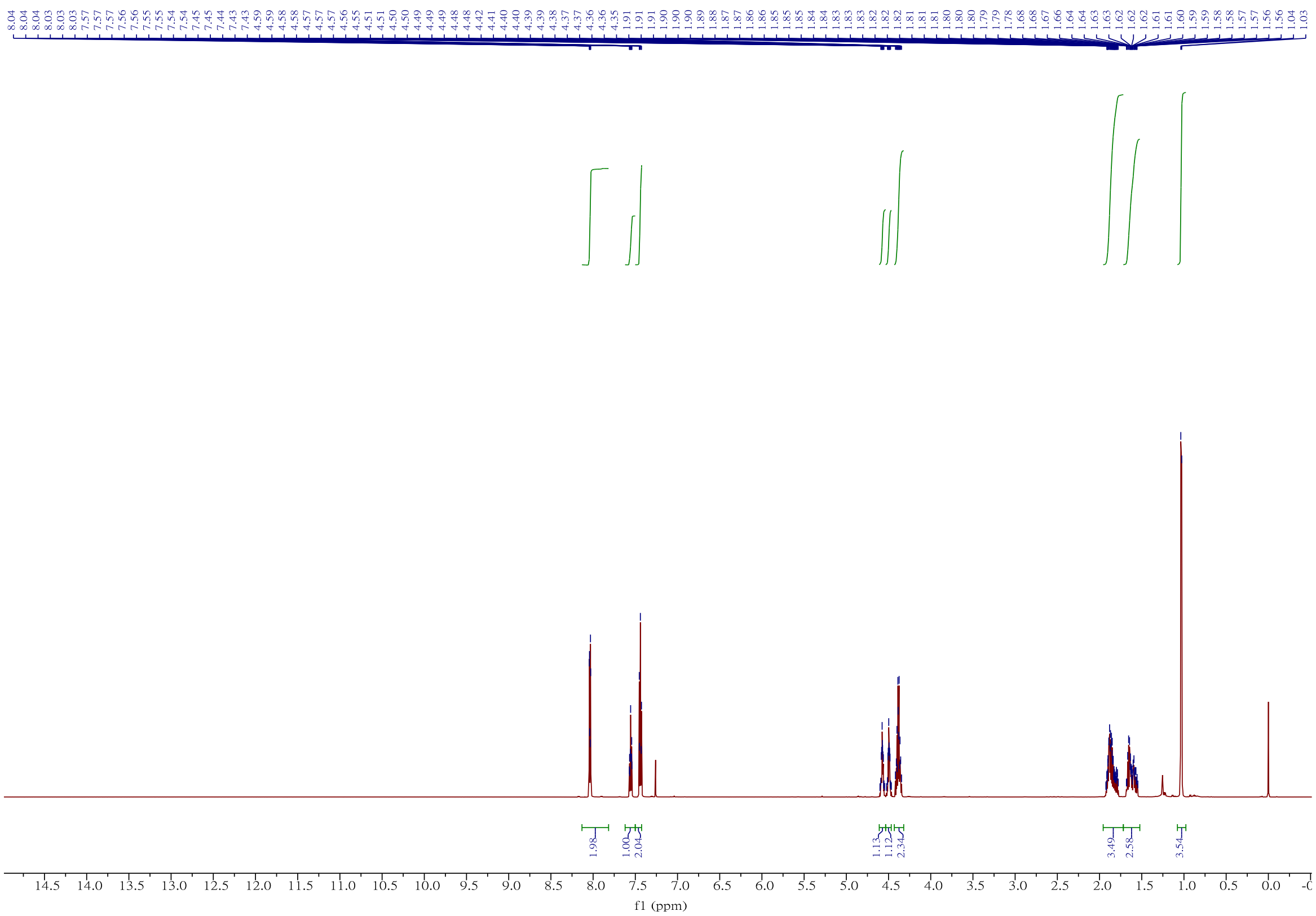

Supplementary Figure 109. ^{1}H NMR (600 MHz) of Compound (43)

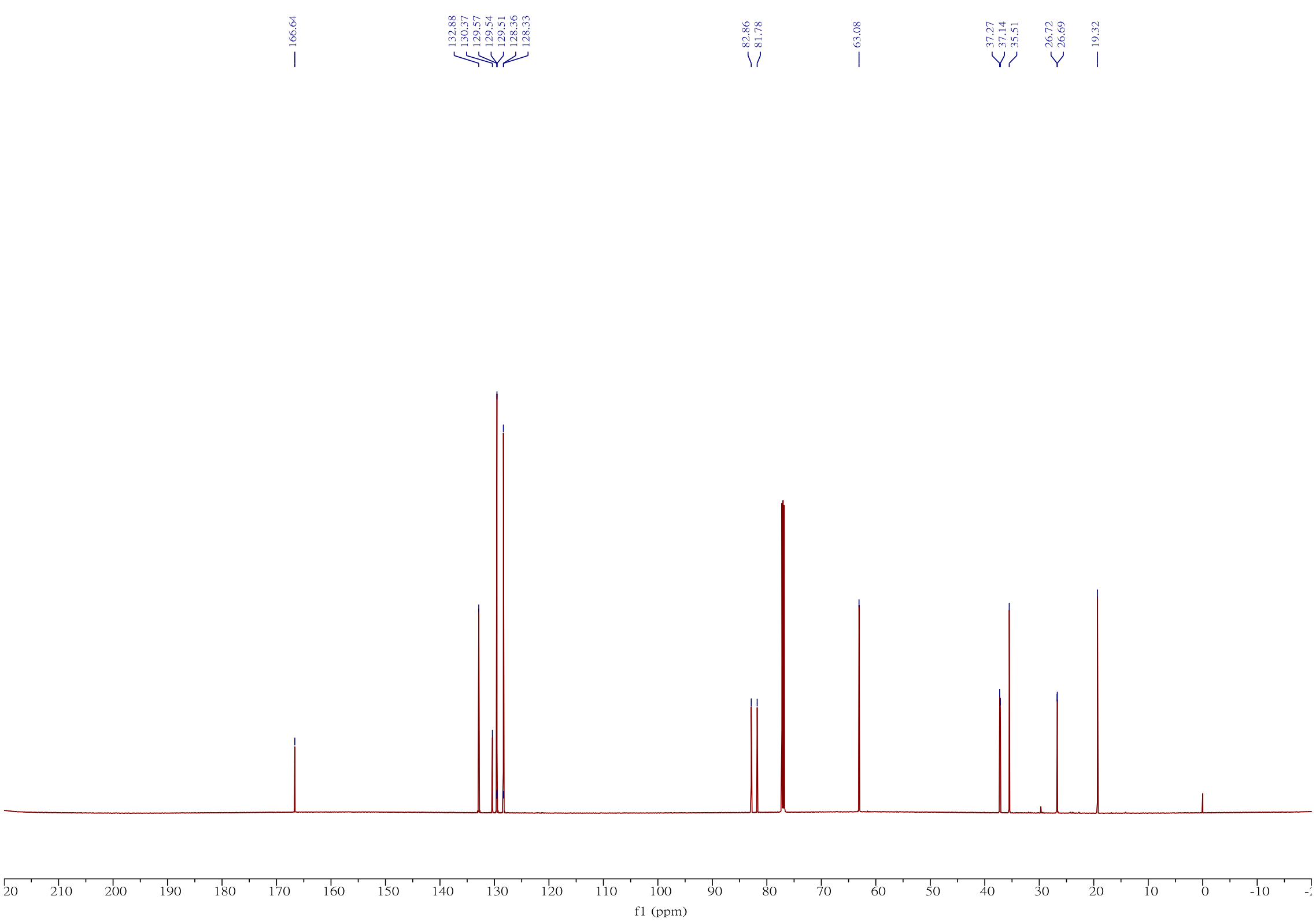

Supplementary Figure 110. ^{13}C NMR (151 MHz) of Compound (43)

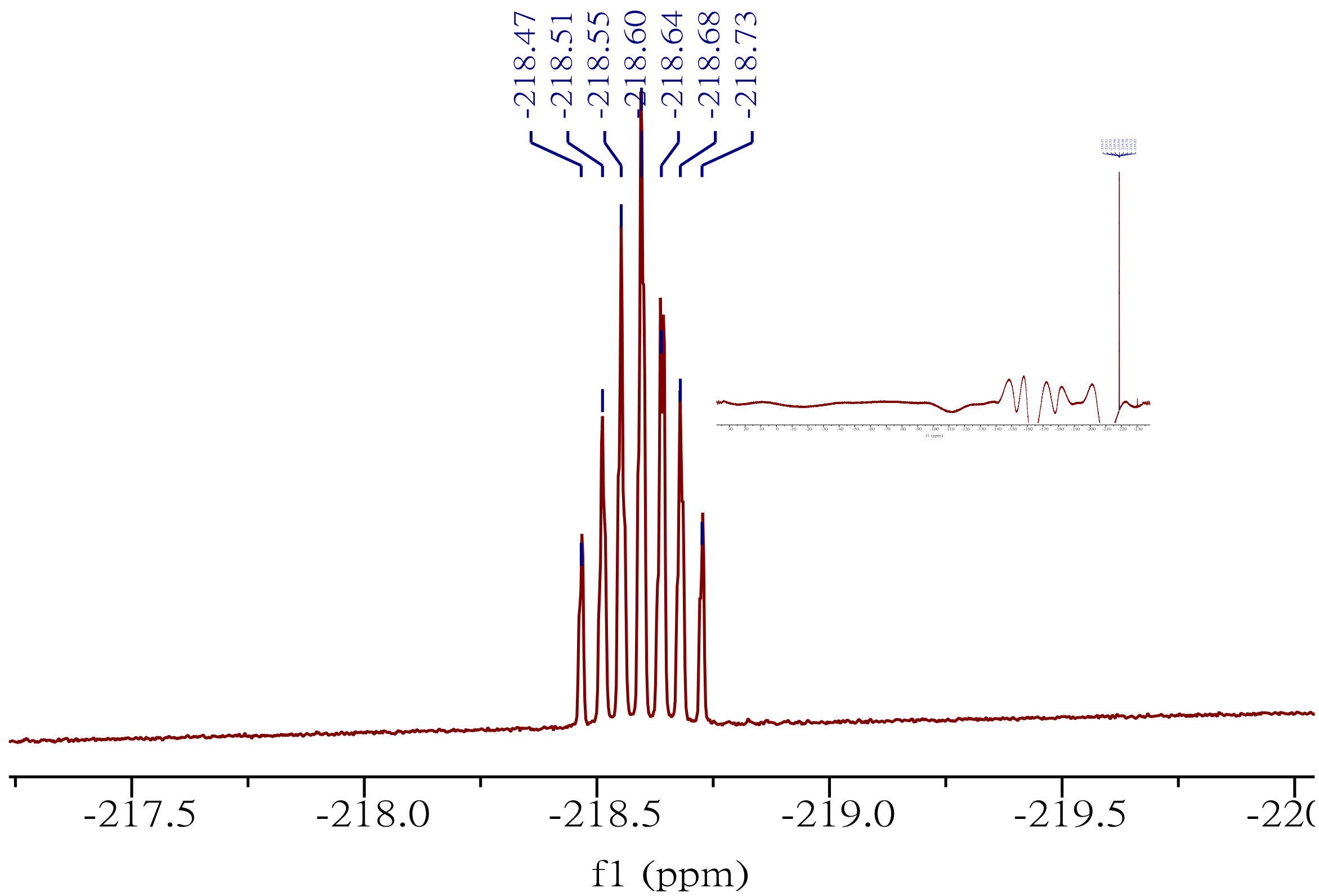

Supplementary Figure 111. ^{19}F NMR (564 MHz) of Compound (43)

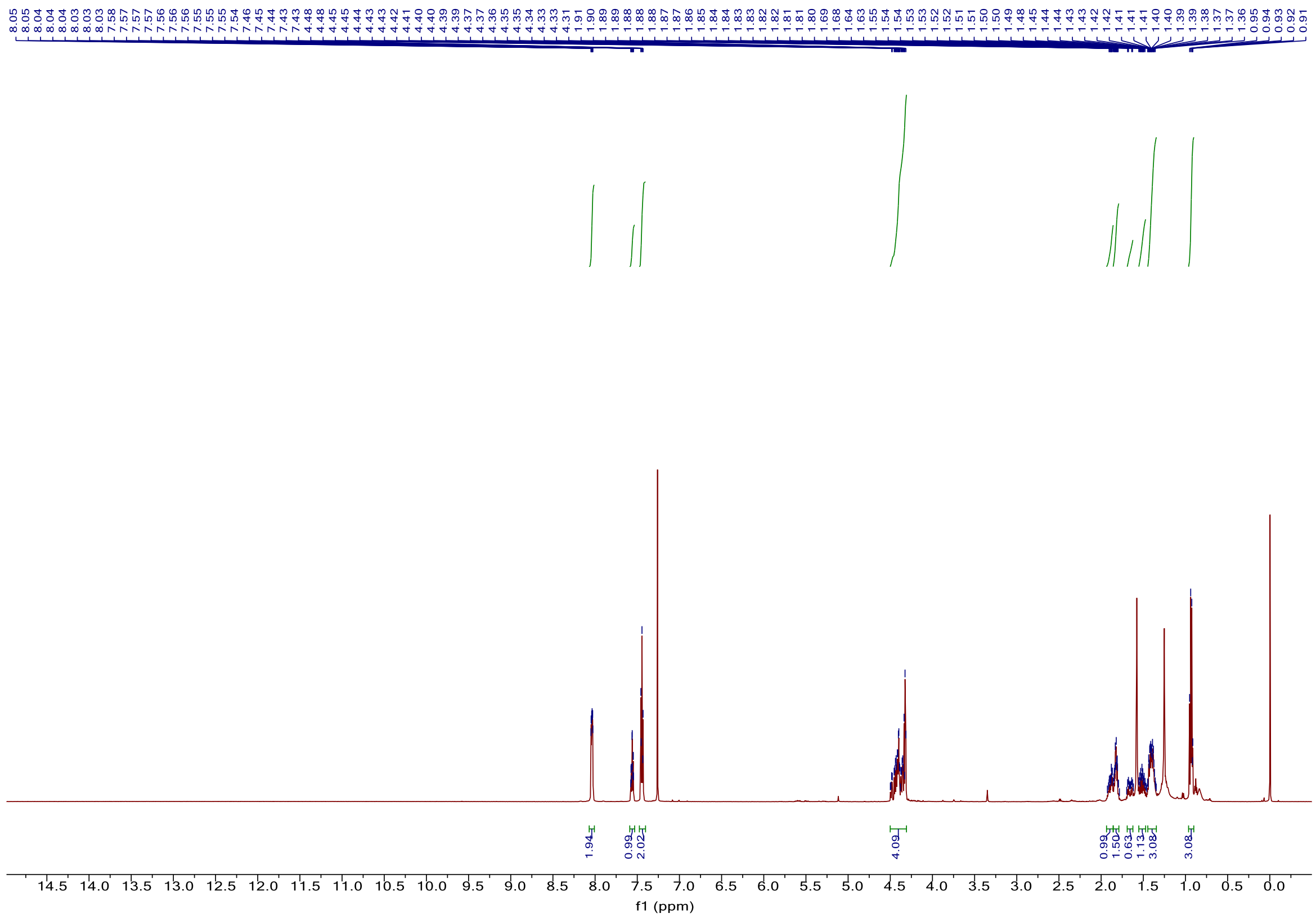

Supplementary Figure 112. ^{1}H NMR (600 MHz) of Compound (44)

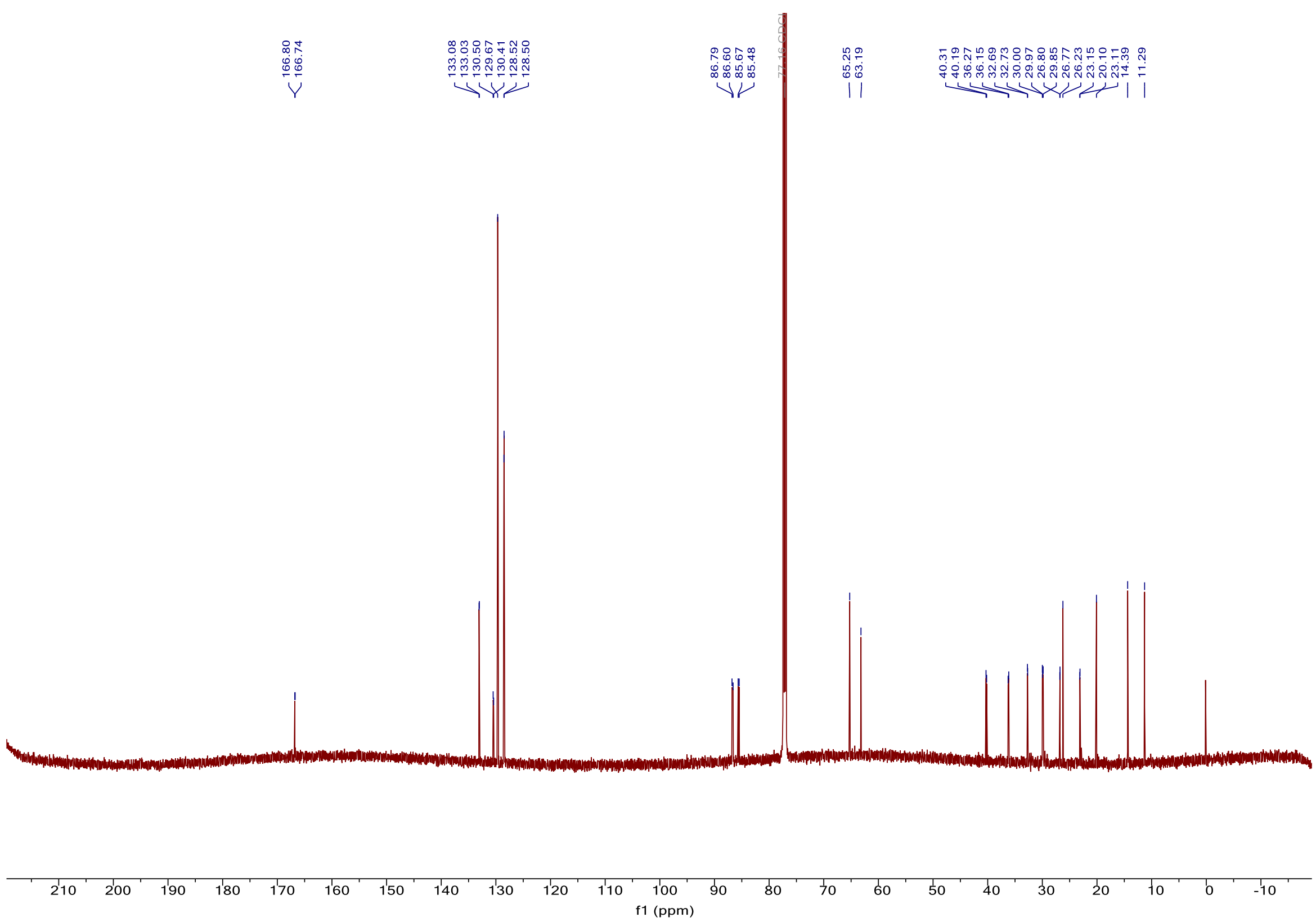

Supplementary Figure 113. ^{13}C NMR (151 MHz) of Compound (44)

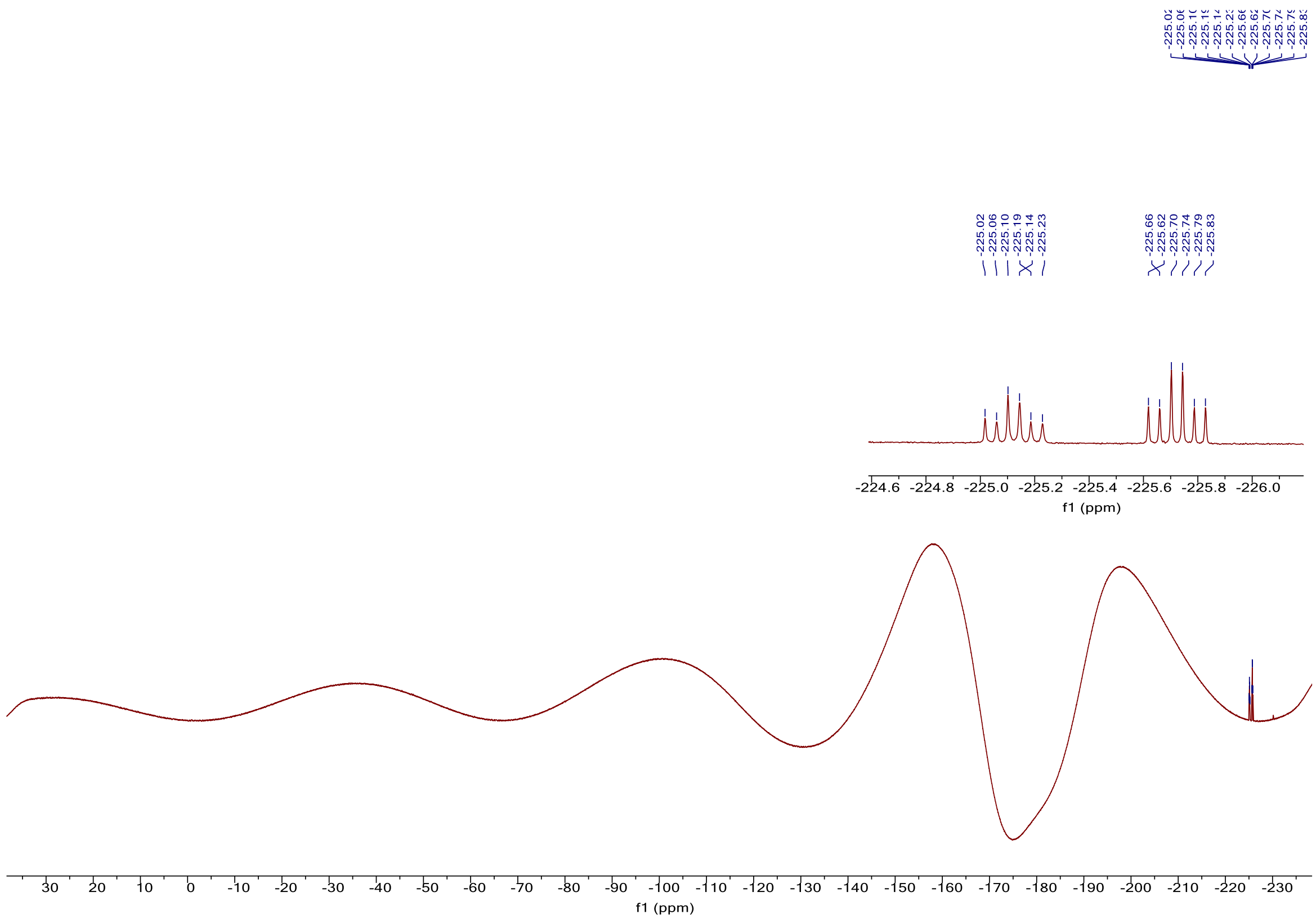

Supplementary Figure 114. ^{19}F NMR (564 MHz) of Compound (44)

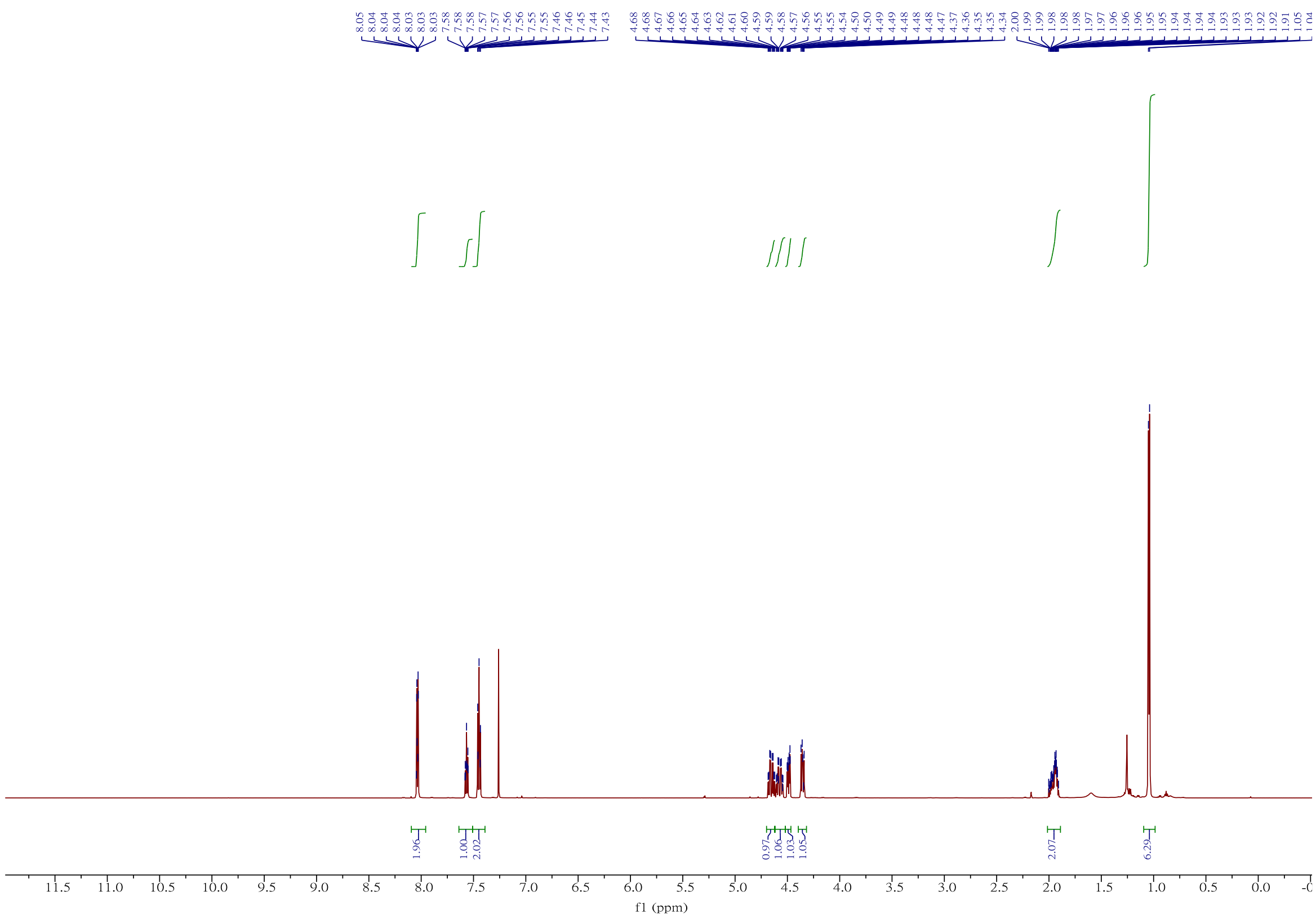

Supplementary Figure 115. ^{1}H NMR (600 MHz) of Compound (45)

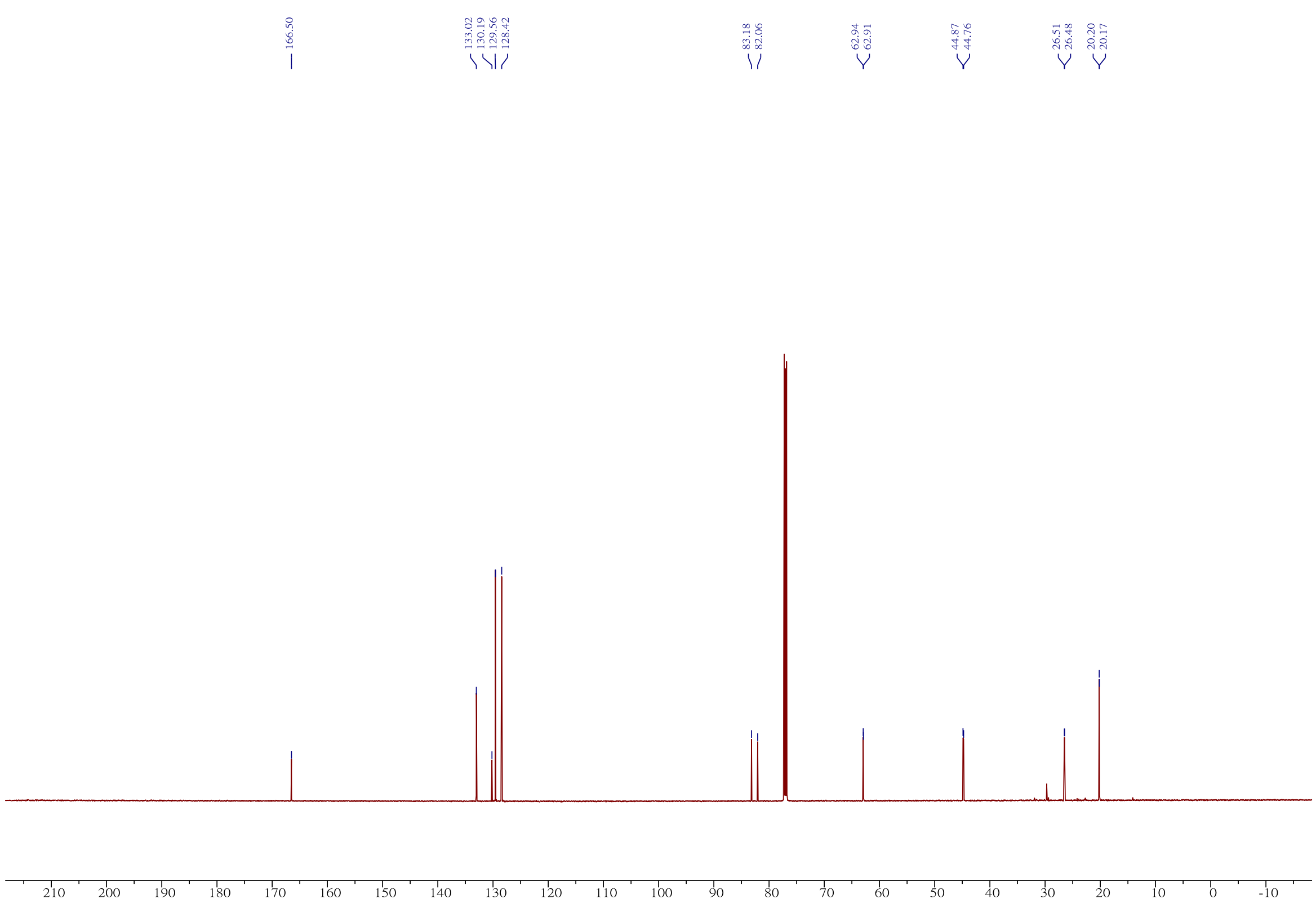

Supplementary Figure 116. ^{13}C NMR (151 MHz) of Compound (45)

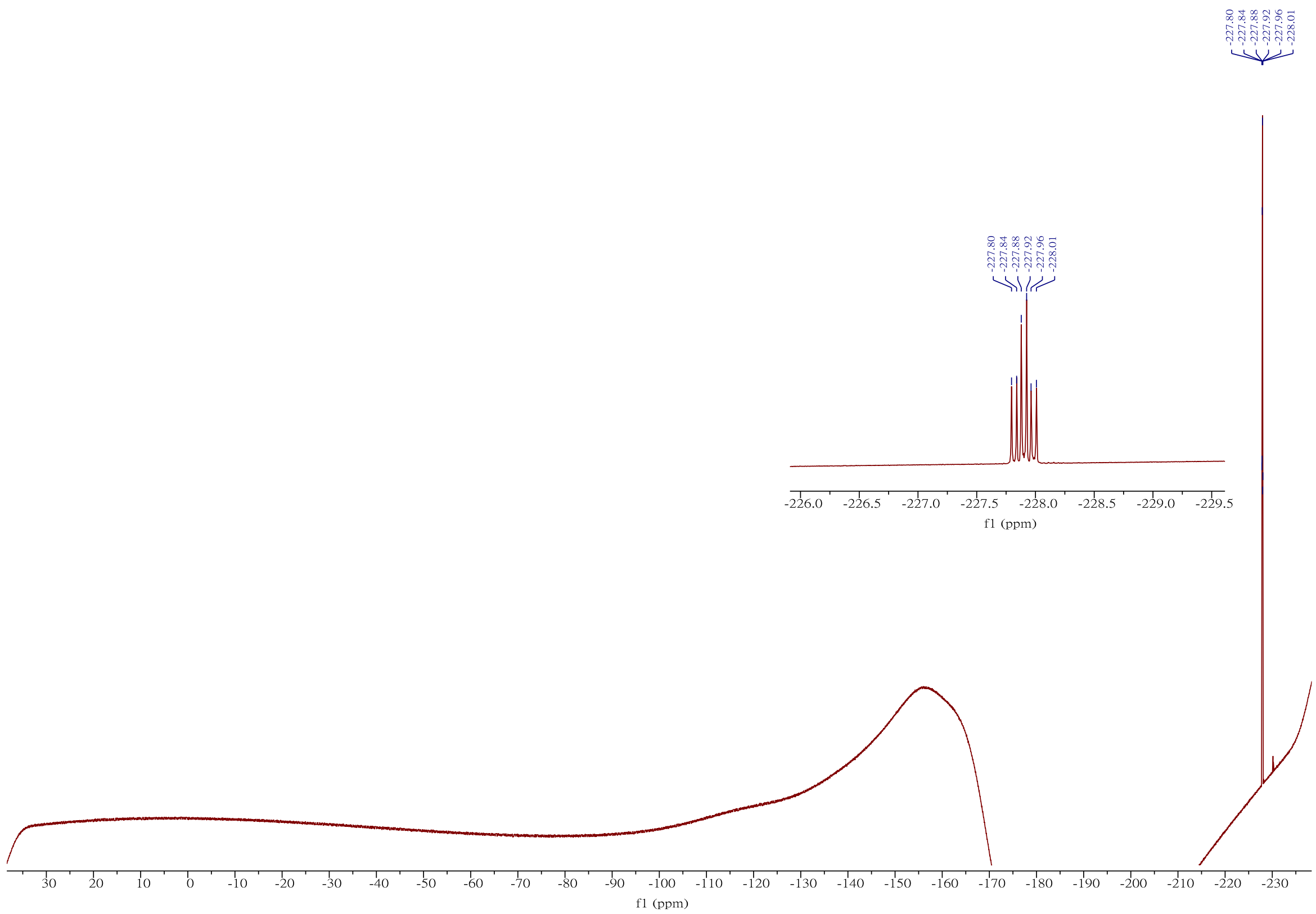

Supplementary Figure 117. ^{19}F NMR (564 MHz) of Compound (45)

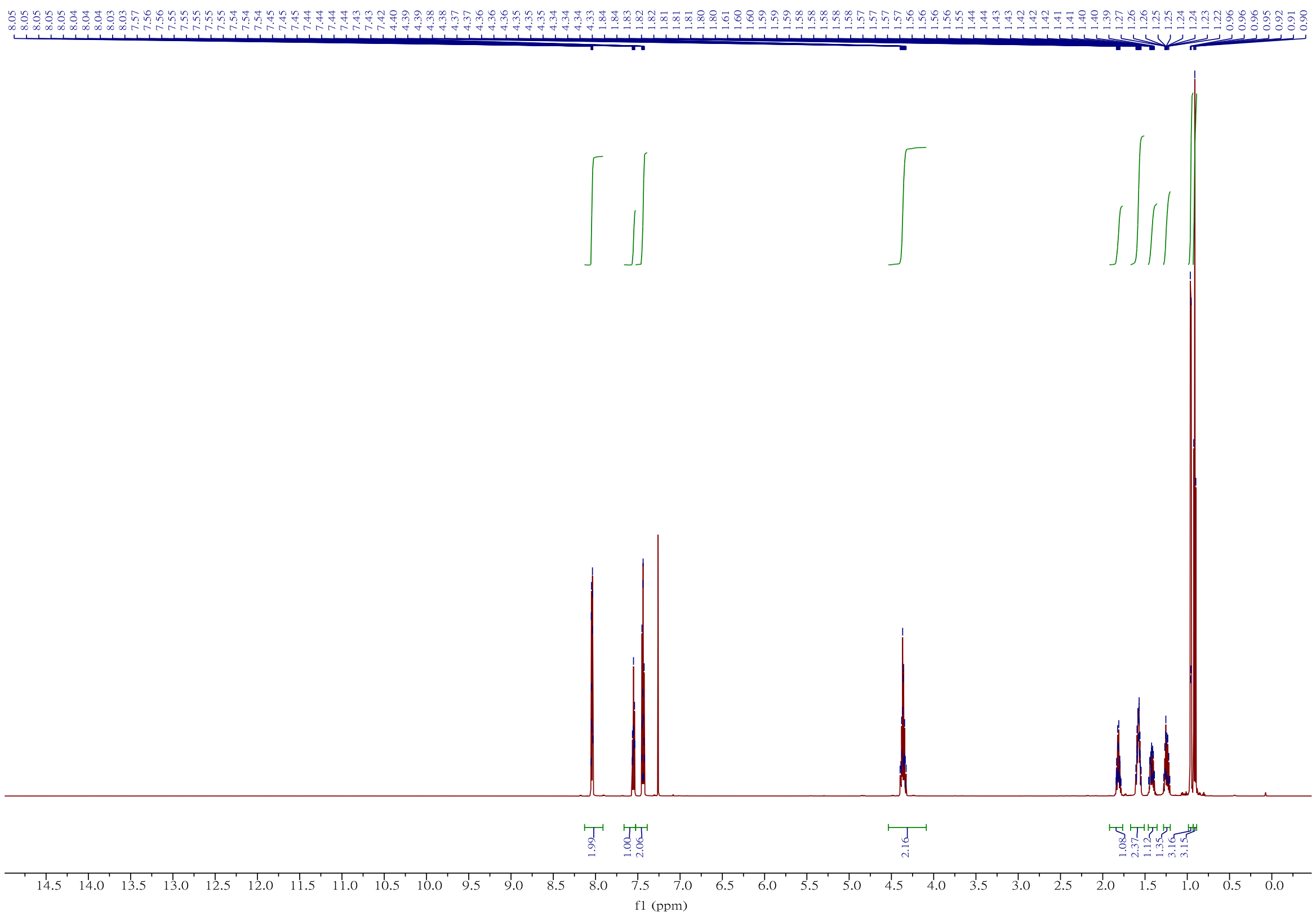

Supplementary Figure 118. ^{1}H NMR (600 MHz) of Compound (46)

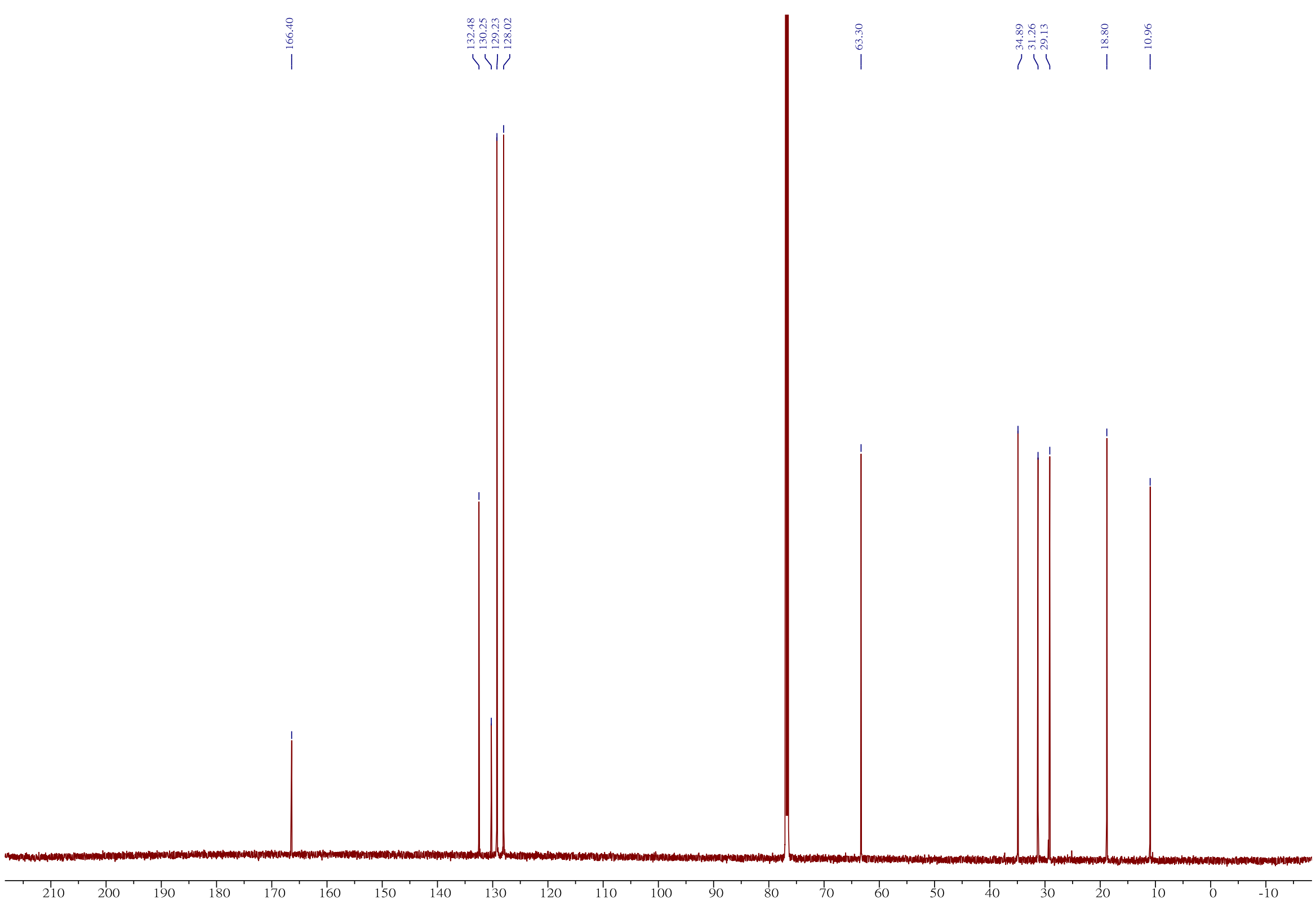

Supplementary Figure 119. ^{13}C NMR (151 MHz) of Compound (46)

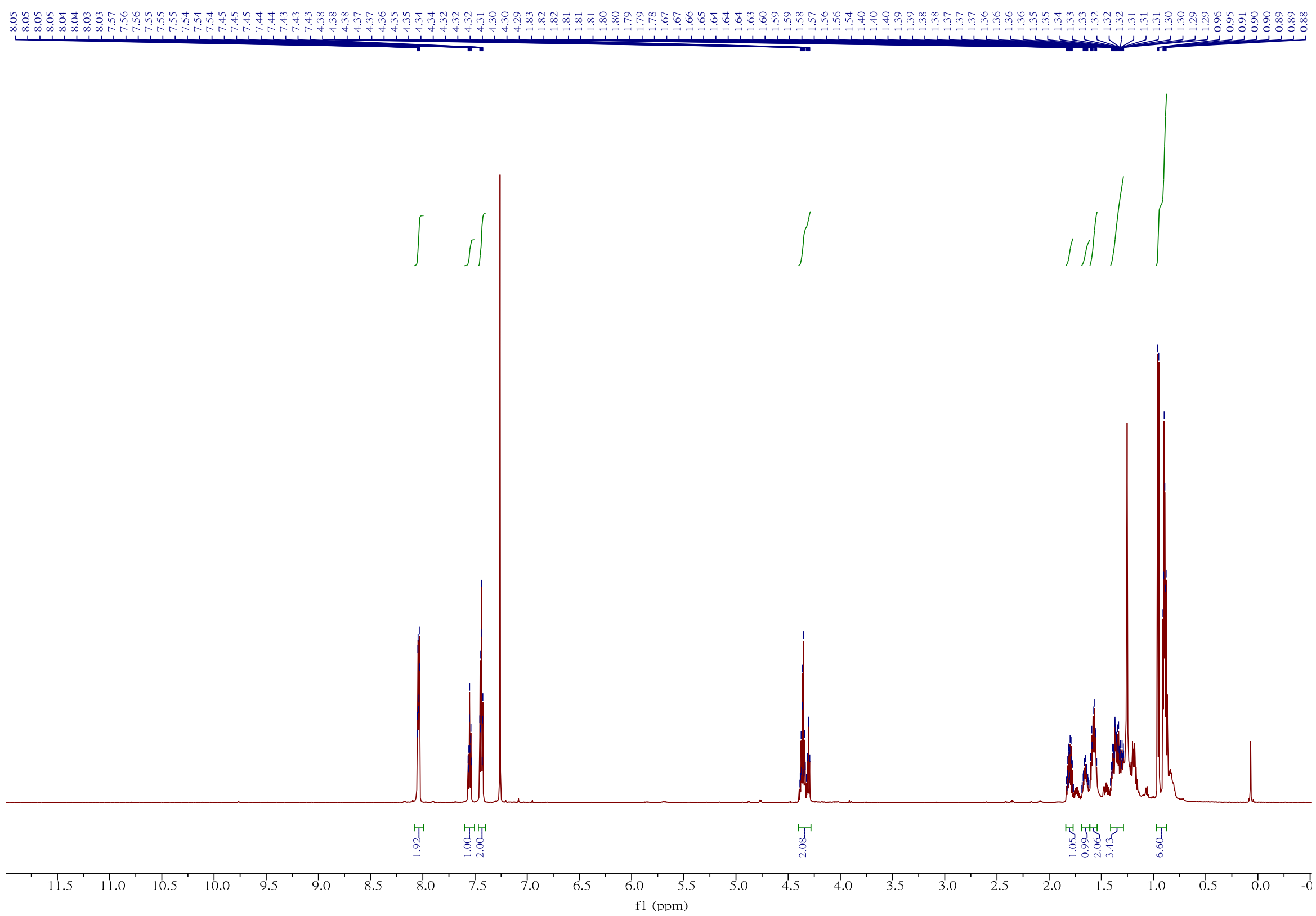

Supplementary Figure 120. ^{1}H NMR (600 MHz) of Compound (47)

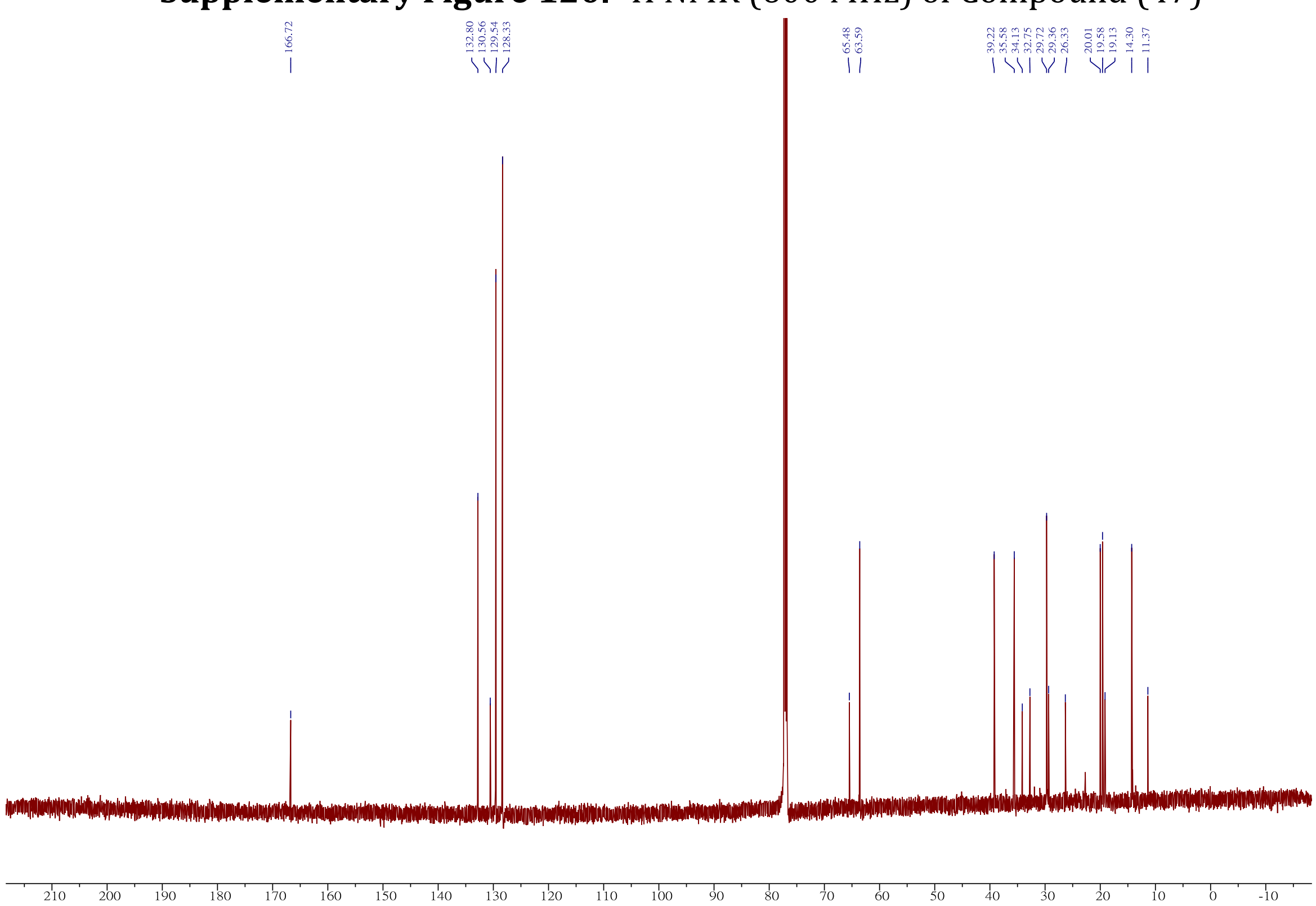

Supplementary Figure 121. ^{13}C NMR (151 MHz) of Compound (47)

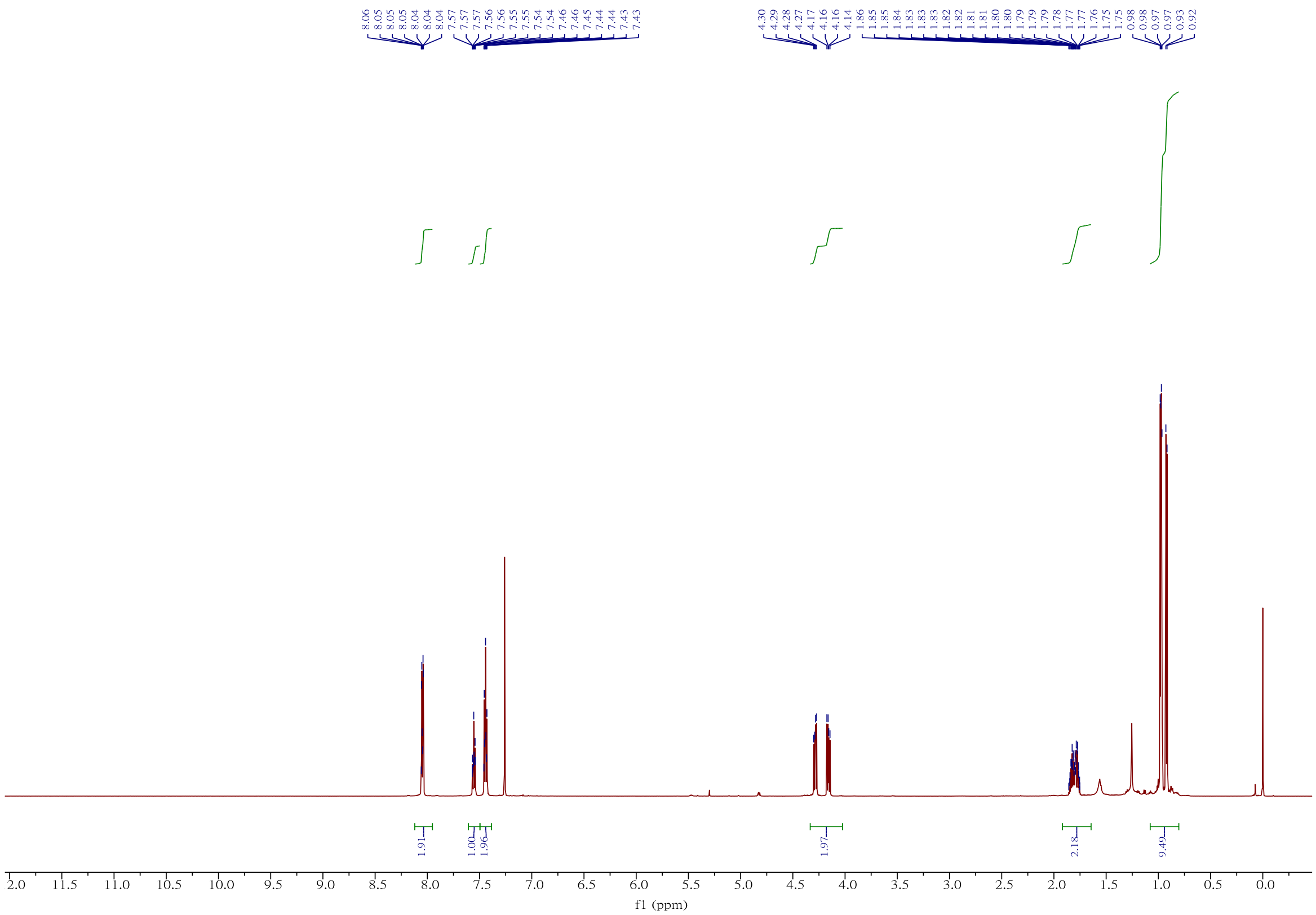

Supplementary Figure 122. ^{1}H NMR (600 MHz) of Compound (48)

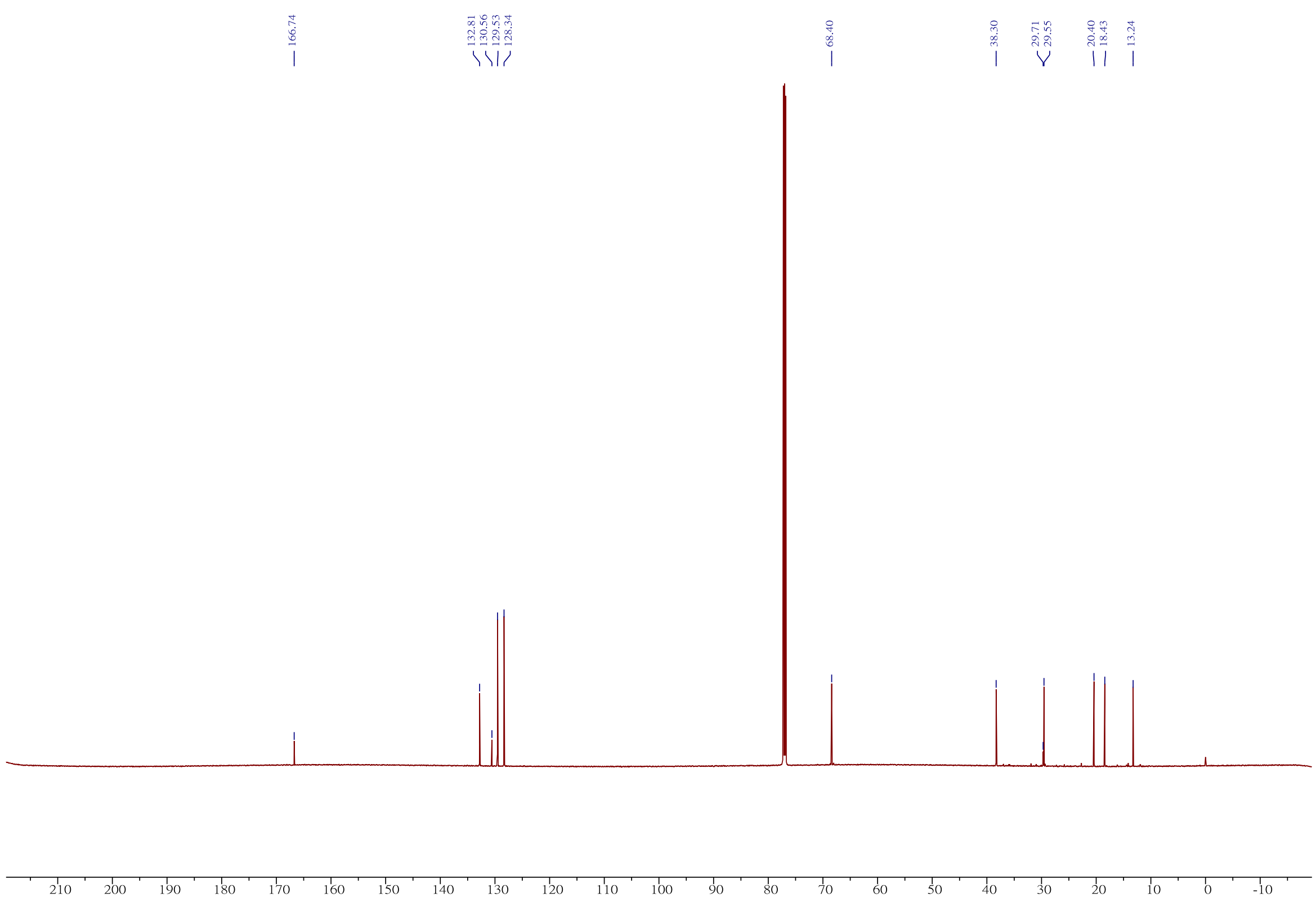

Supplementary Figure 123. ^{13}C NMR (151 MHz) of Compound (48)

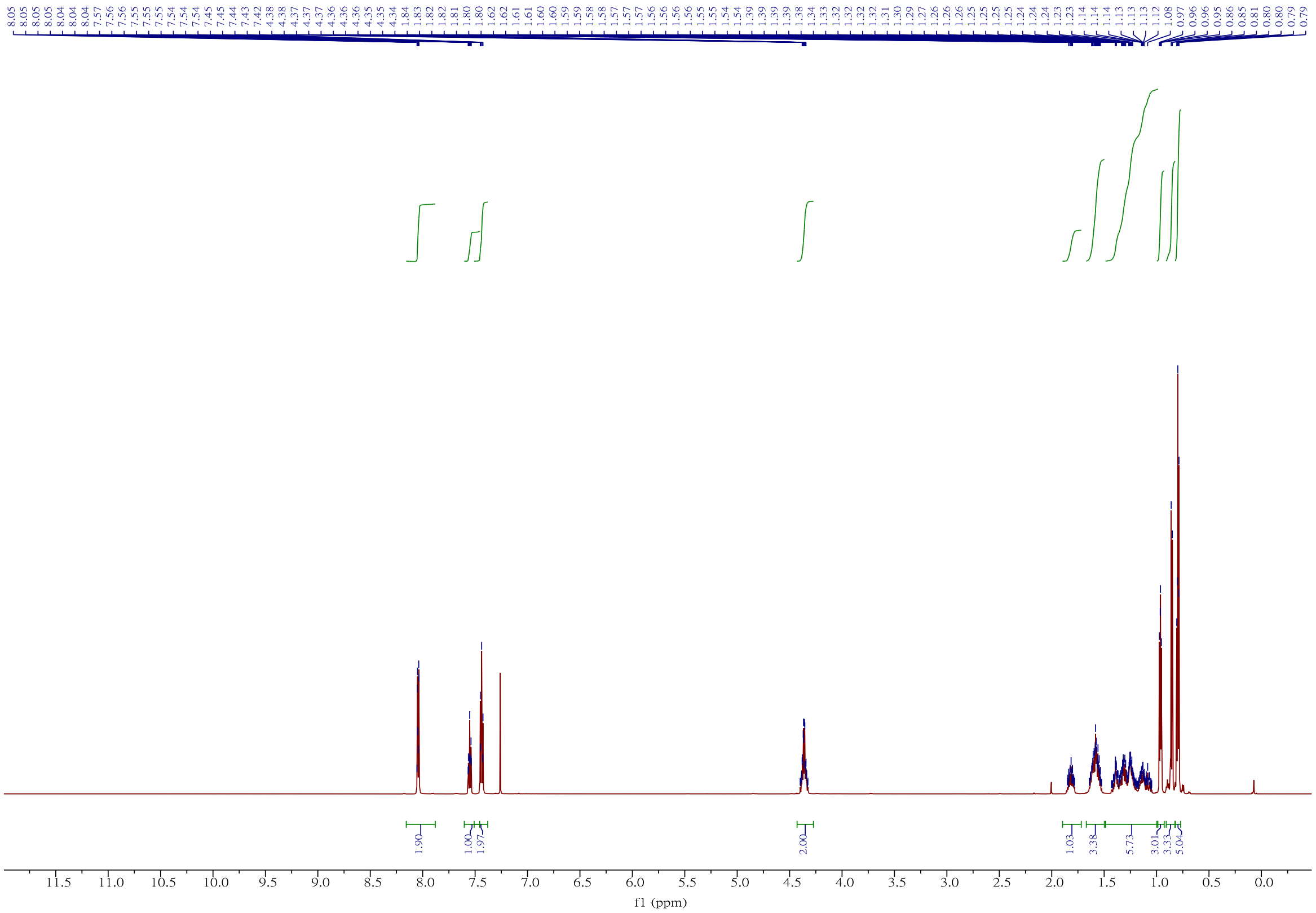

Supplementary Figure 124. ^{1}H NMR (600 MHz) of Compound (49)

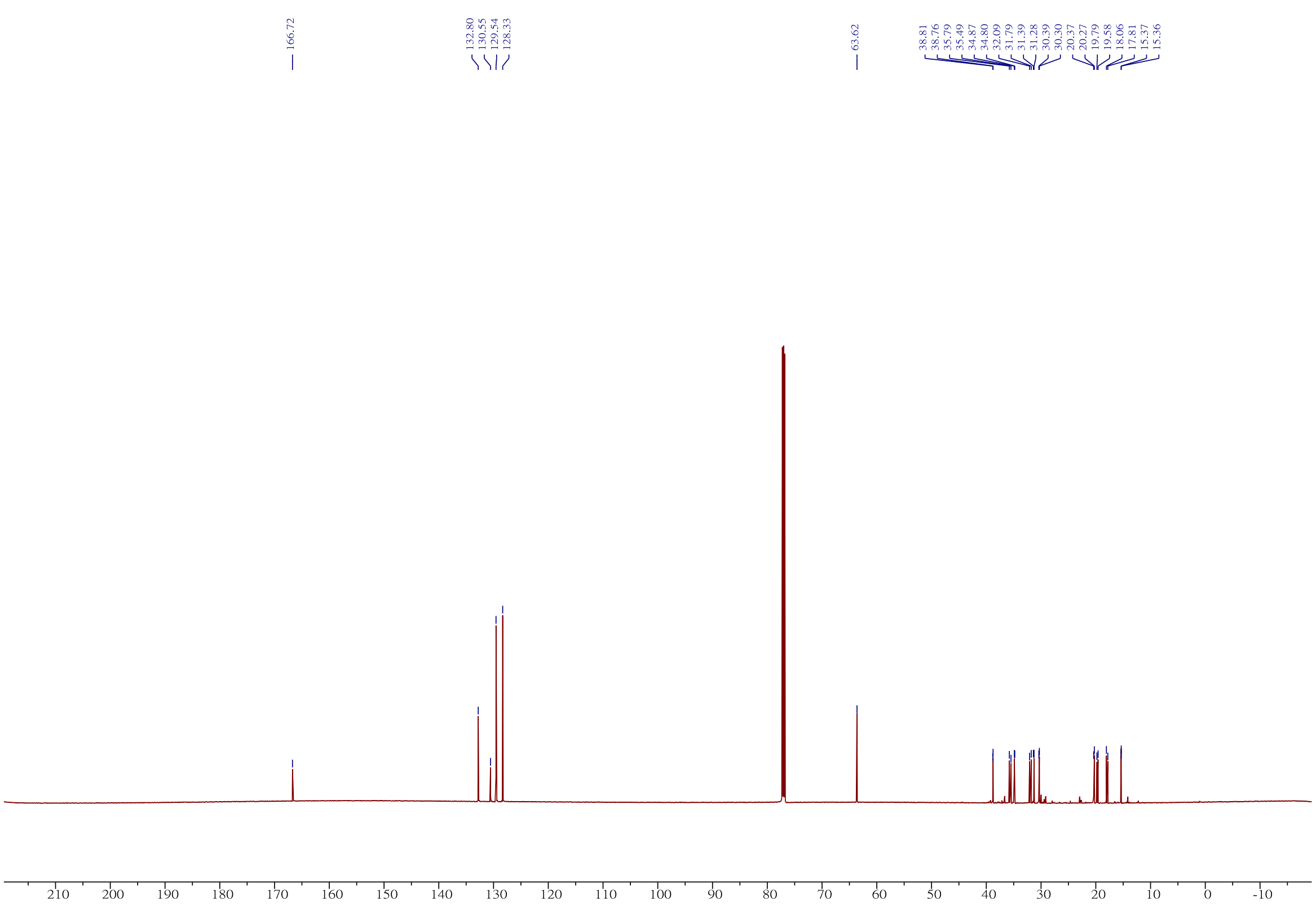

Supplementary Figure 125. ^{13}C NMR (151 MHz) of Compound (49)

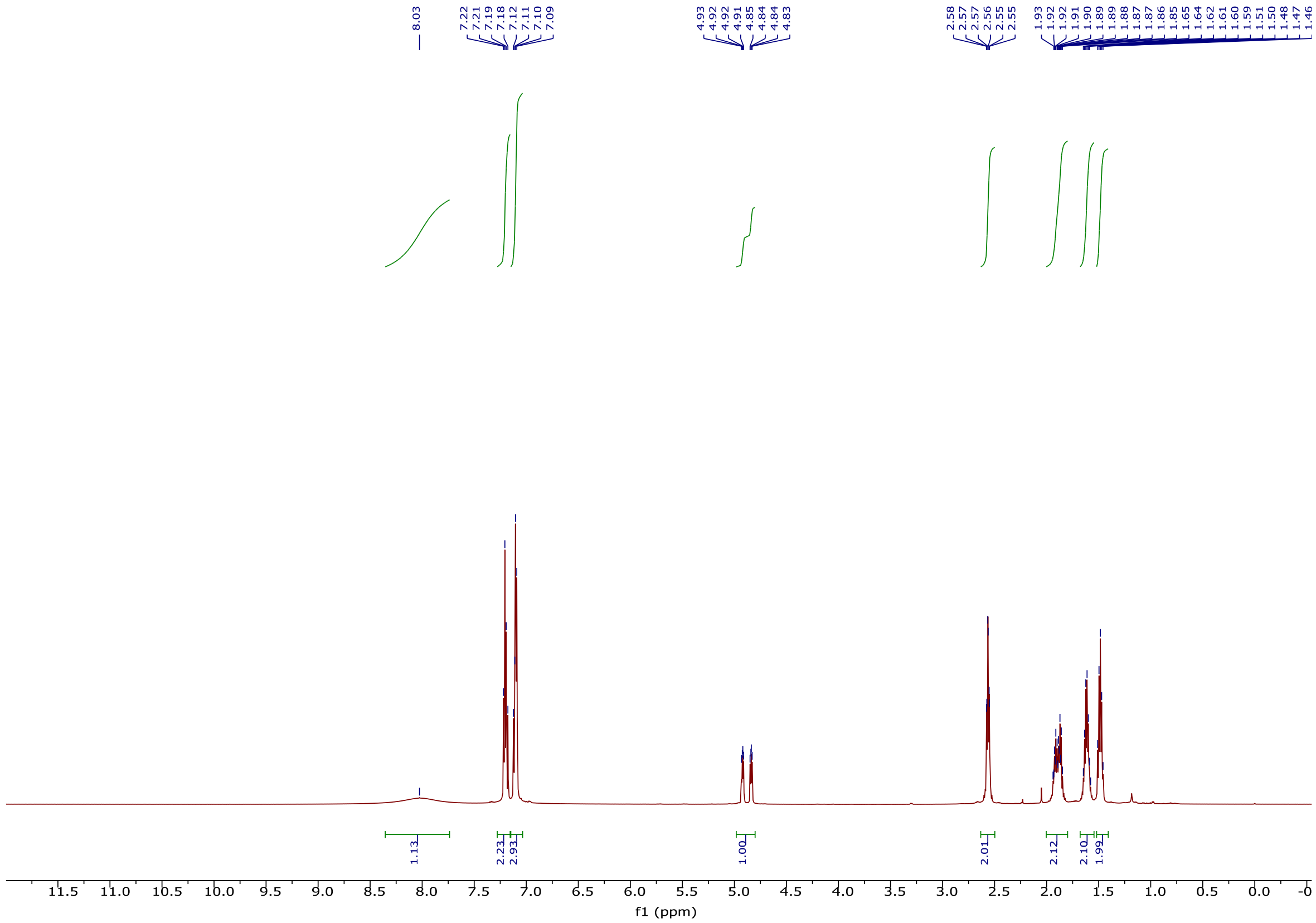

Supplementary Figure 126. ^{1}H NMR (600 MHz) of Compound (50)

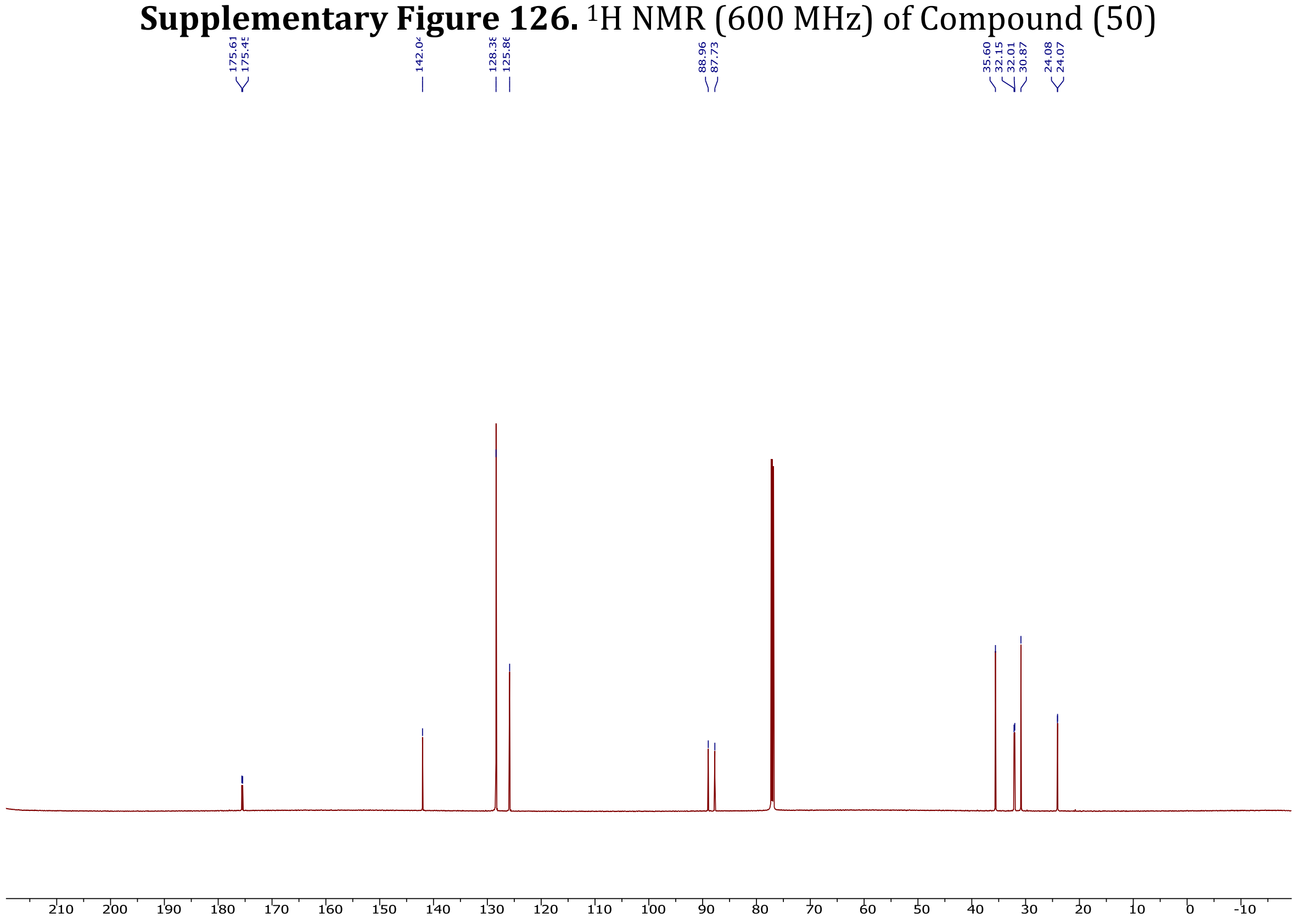

Supplementary Figure 127. ^{13}C NMR (151 MHz) of Compound (50)

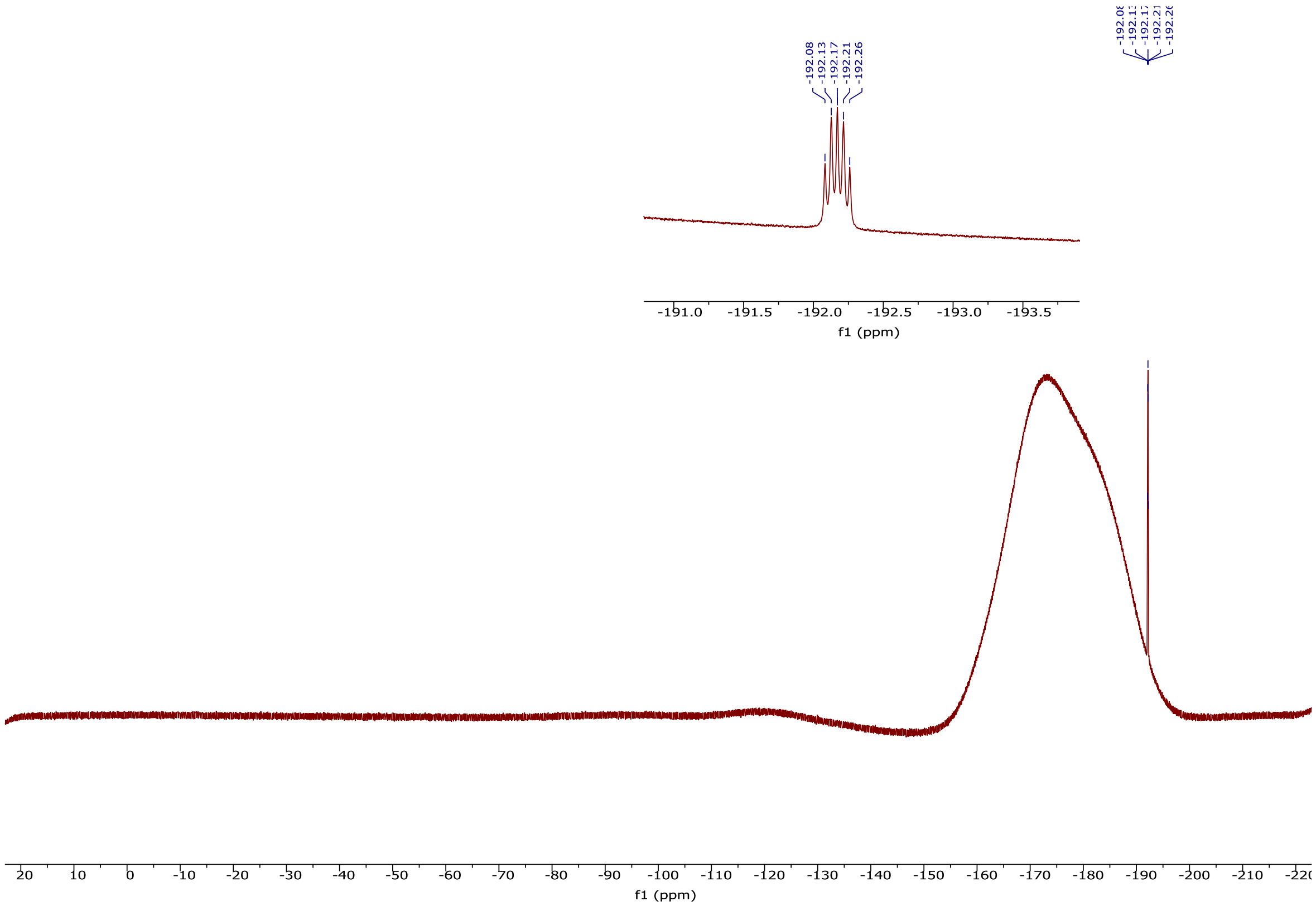

Supplementary Figure 128. ^{19}F NMR (564 MHz) of Compound (50)

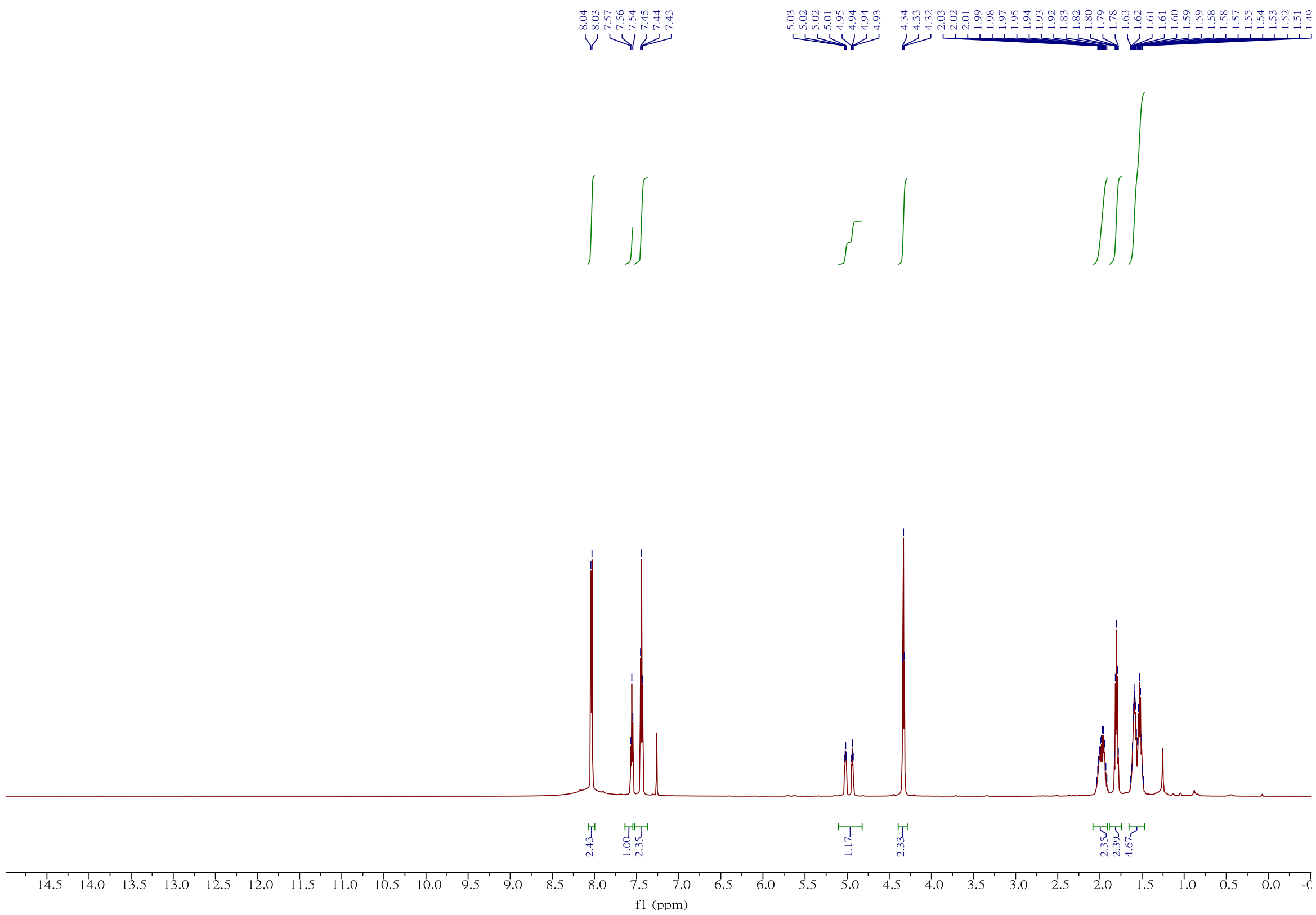

Supplementary Figure 129. ^{1}H NMR (600 MHz) of Compound (51)

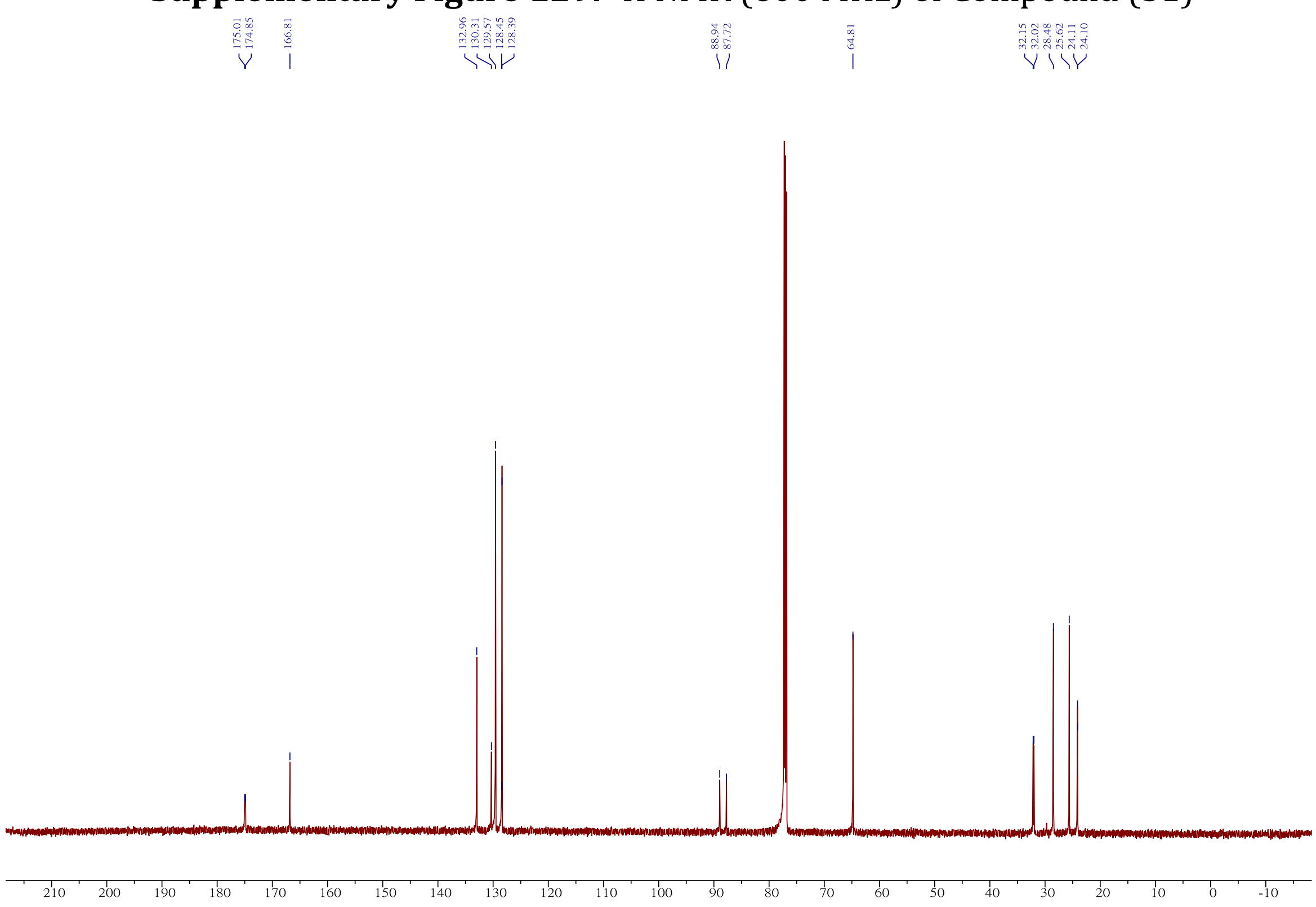

Supplementary Figure 130. ^{13}C NMR (151 MHz) of Compound (51)

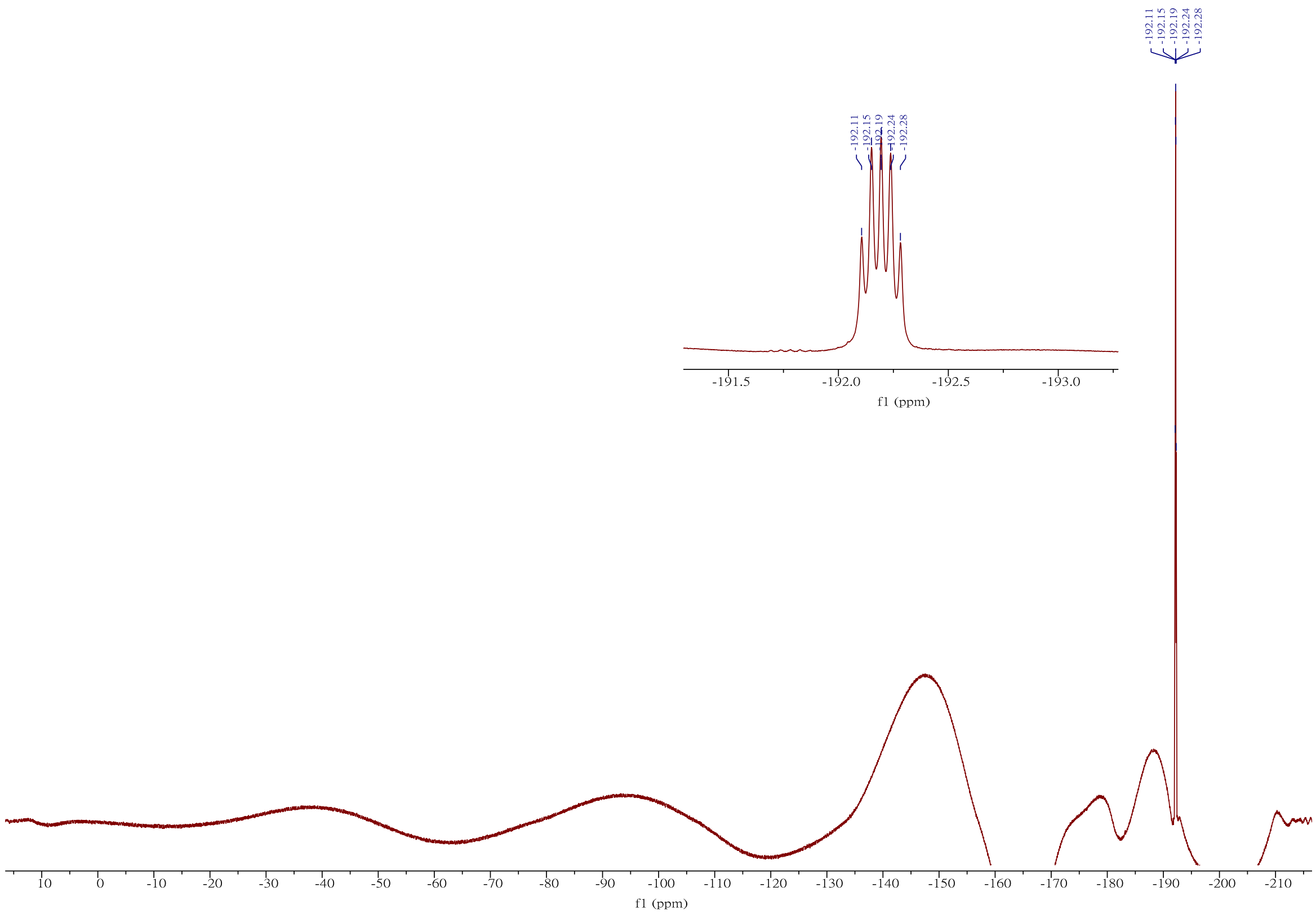

Supplementary Figure 131. ^{19}F NMR (564 MHz) of Compound (51)

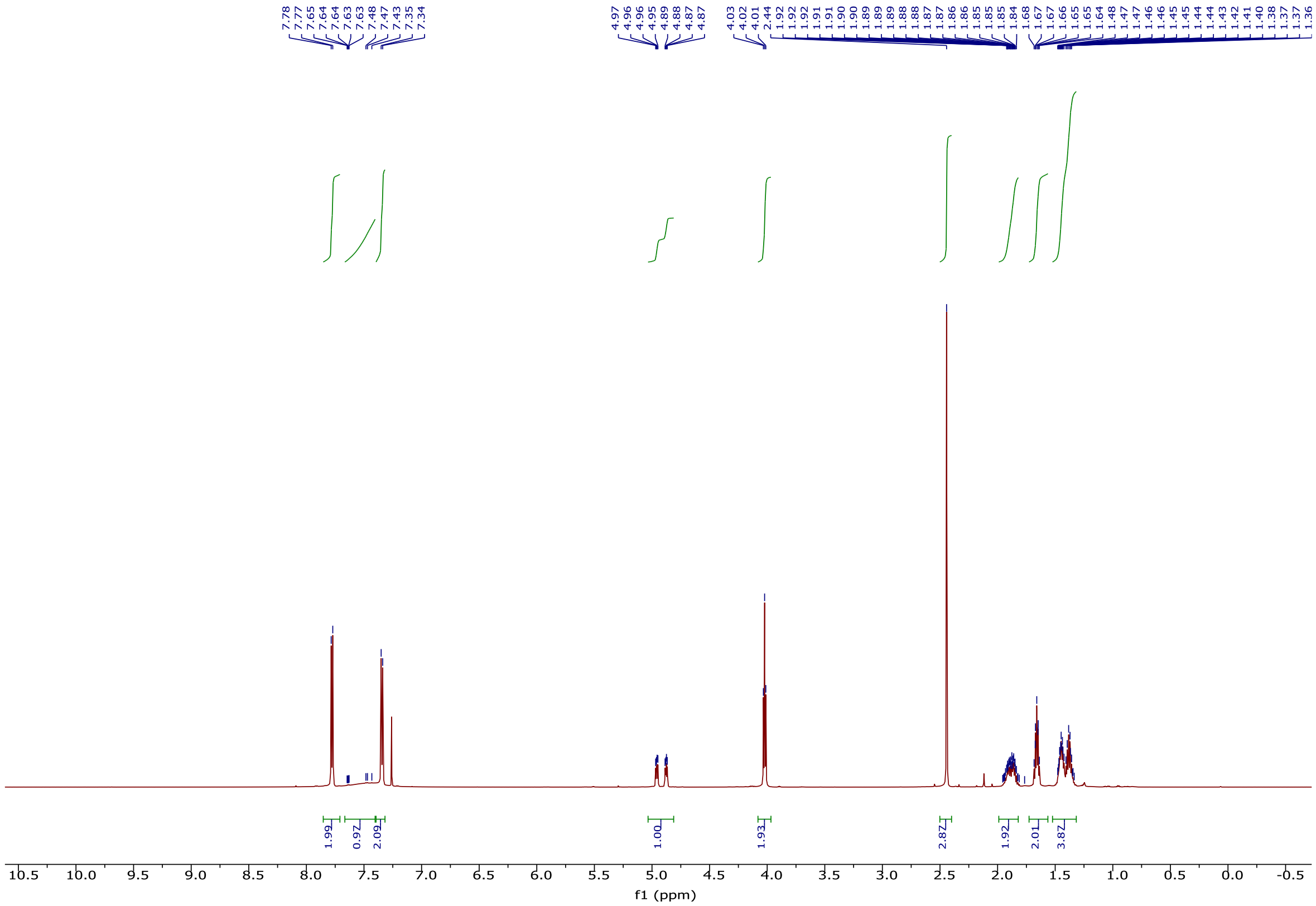

Supplementary Figure 132. ^{1}H NMR (600 MHz) of Compound (52)

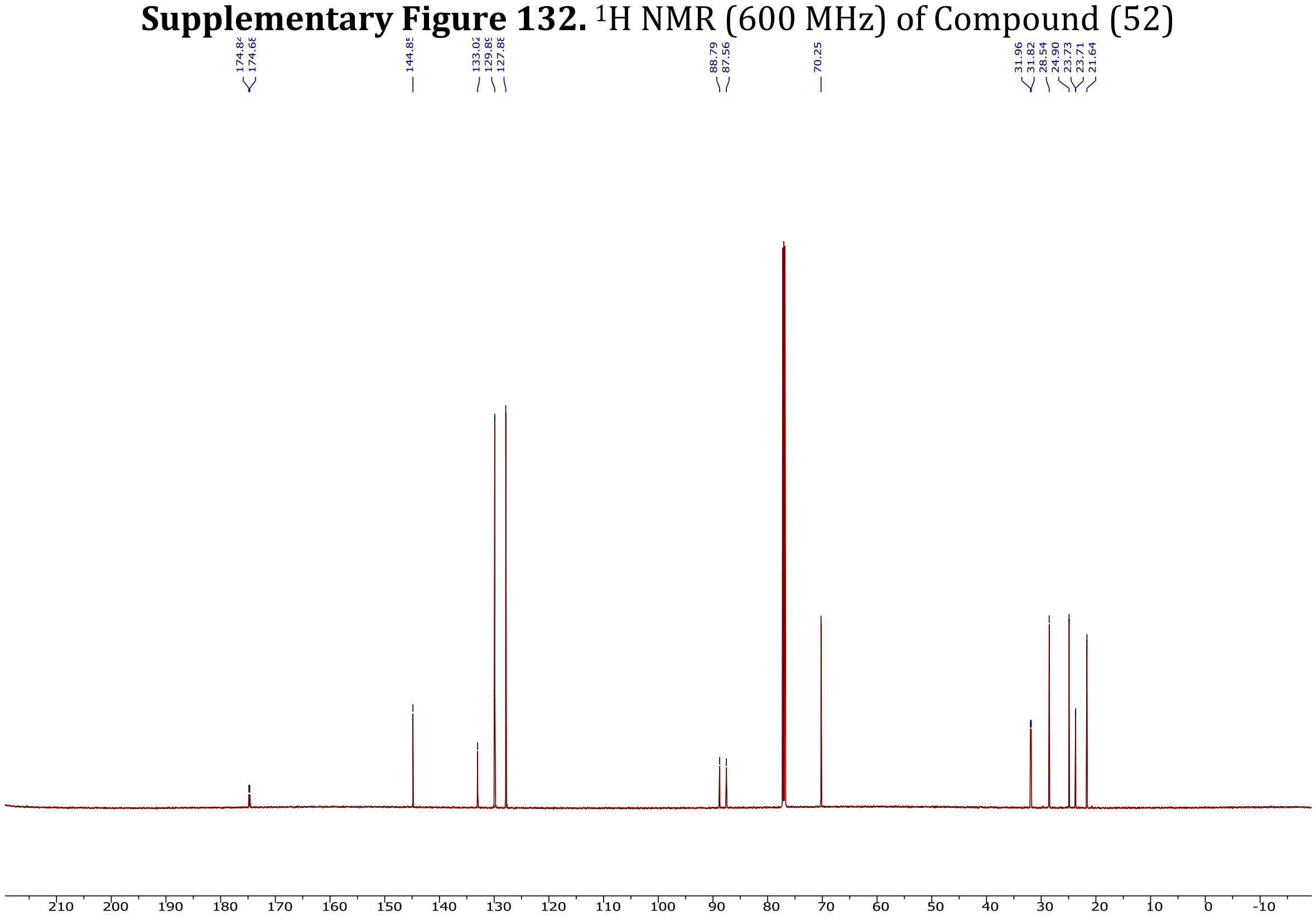

Supplementary Figure 133. ^{13}C NMR (151 MHz) of Compound (52)

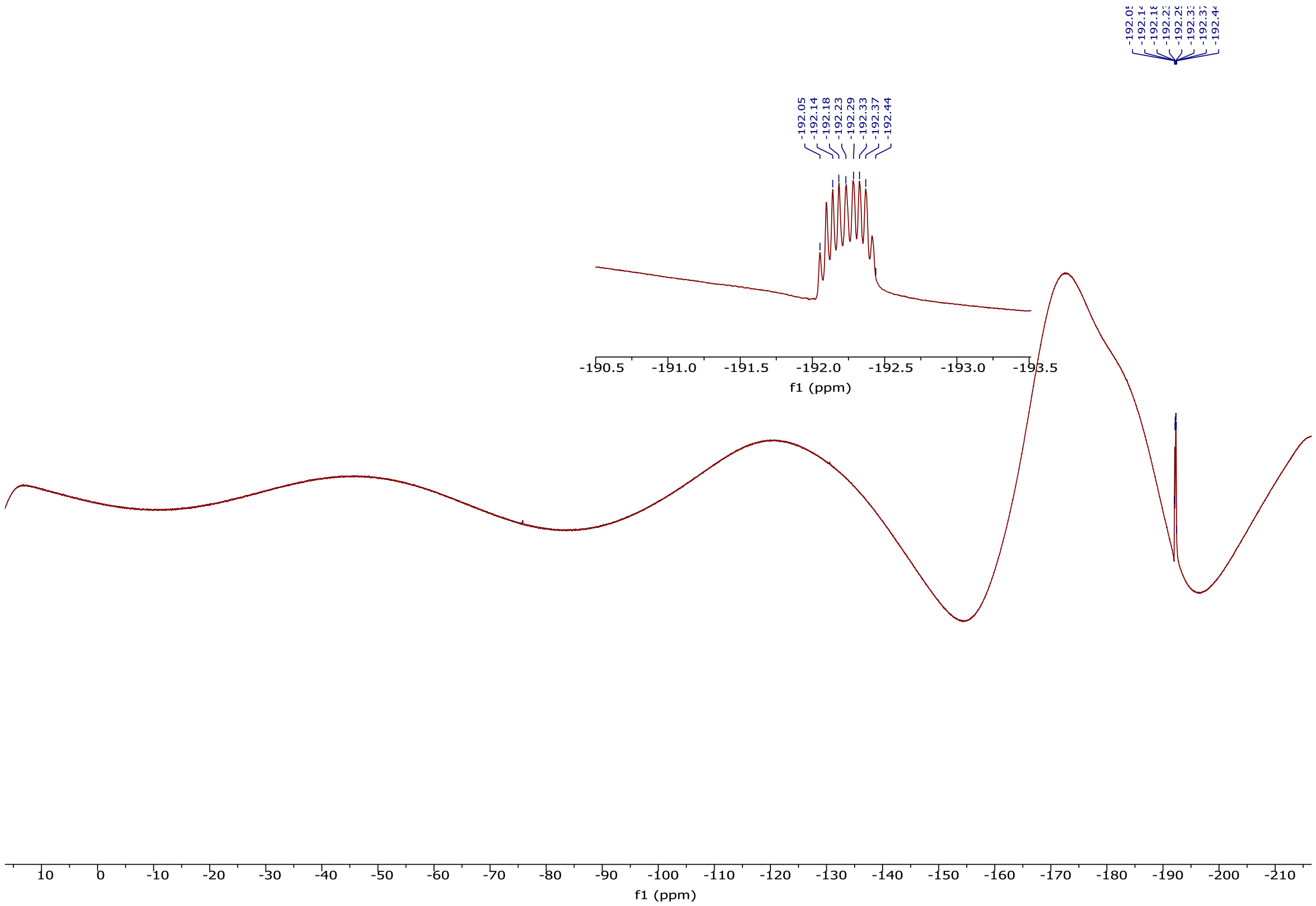

Supplementary Figure 134. ^{19}F NMR (564 MHz) of Compound (52)

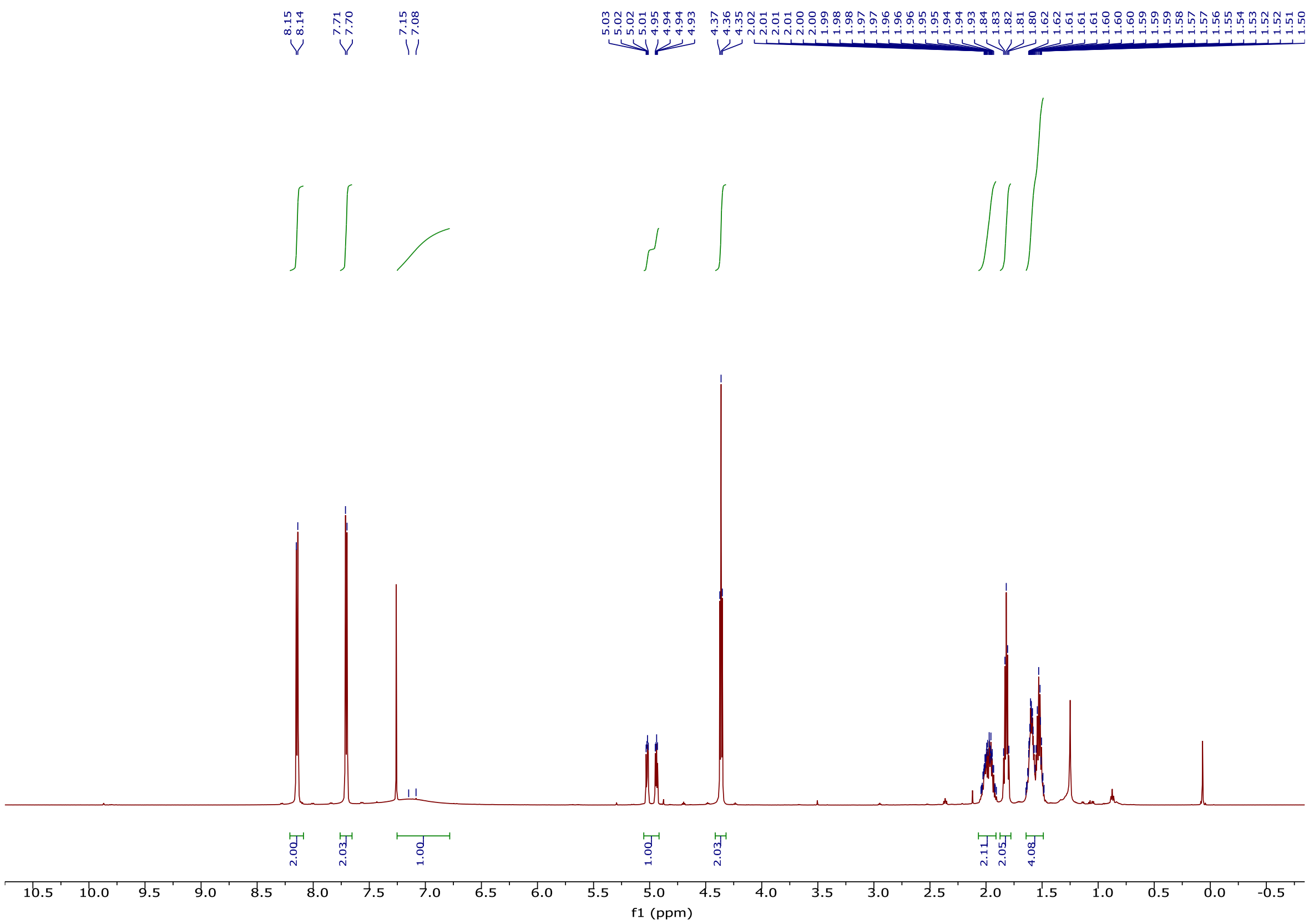

Supplementary Figure 135. ^{1}H NMR (600 MHz) of Compound (53)

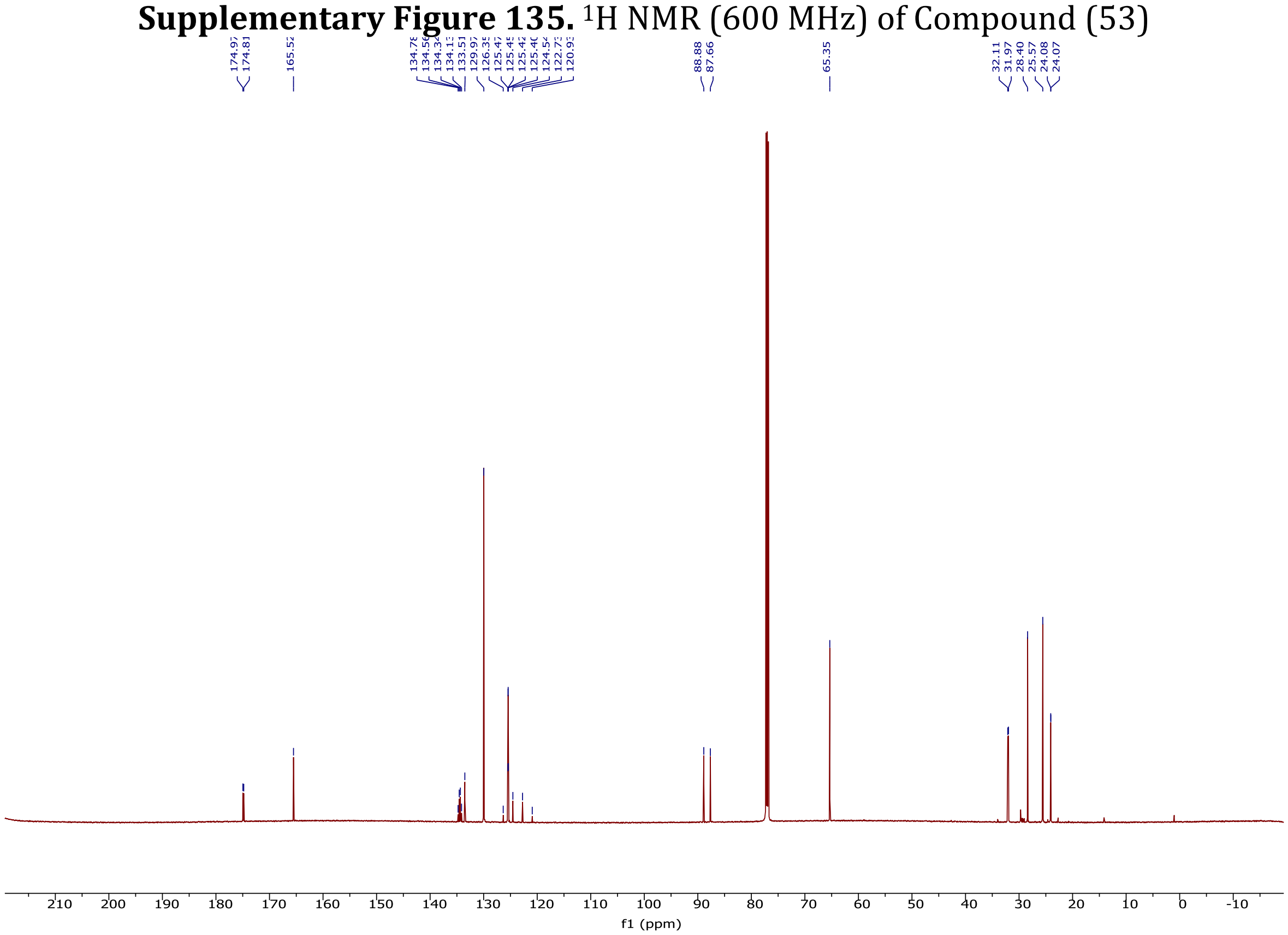

Supplementary Figure 136. ^{13}C NMR (151 MHz) of Compound (53)

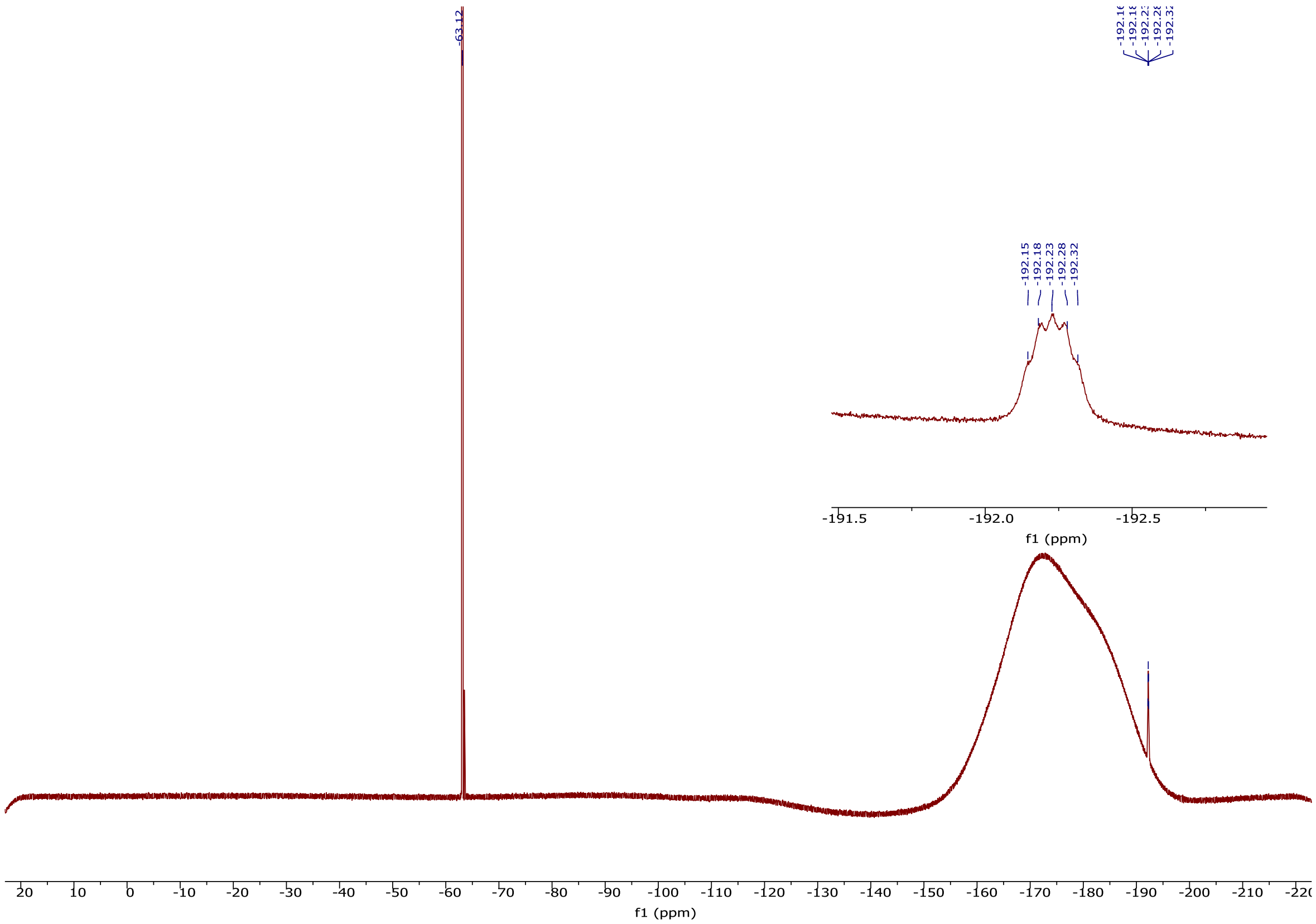

Supplementary Figure 137. ^{19}F NMR (564 MHz) of Compound (53)

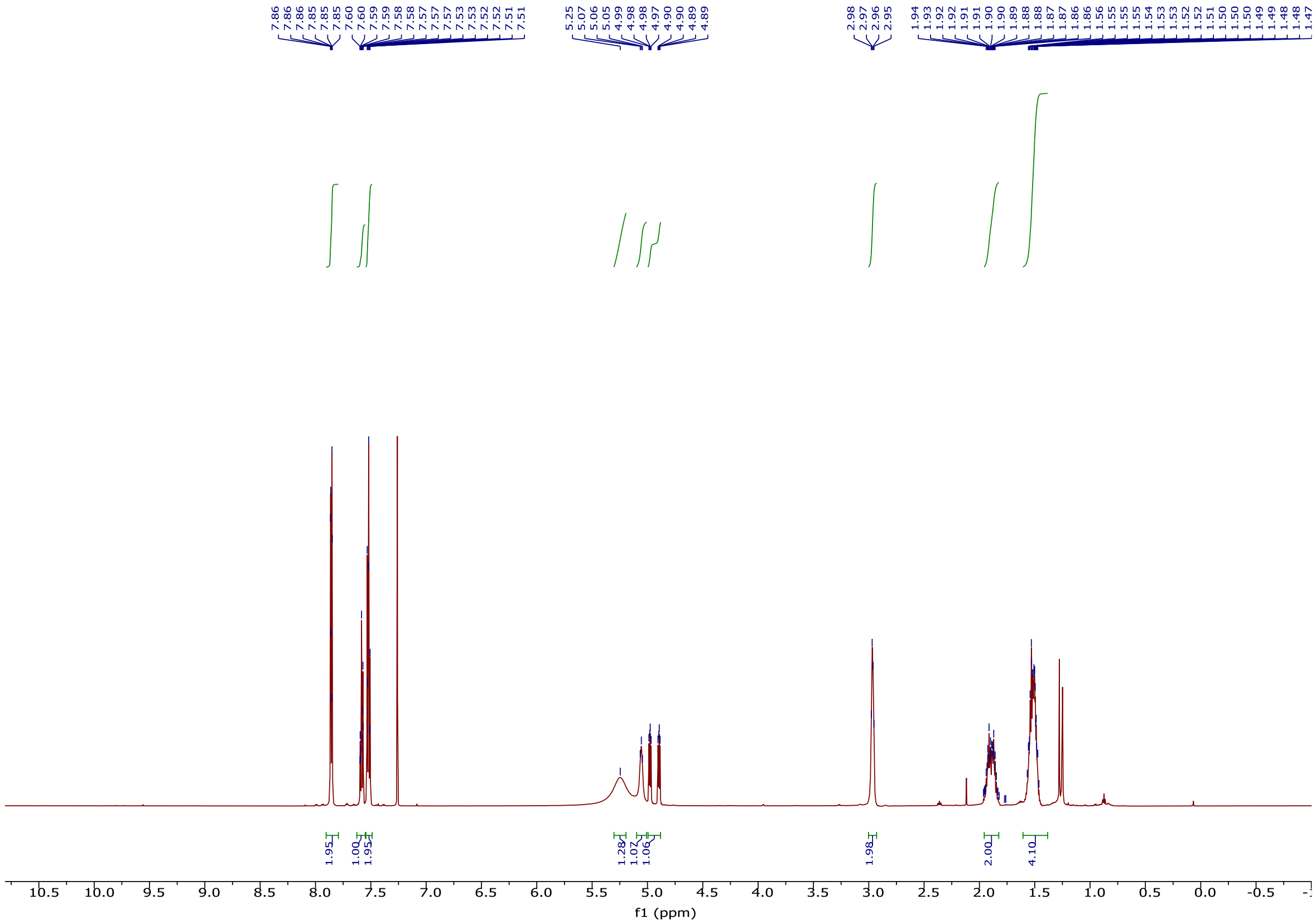

Supplementary Figure 138. ^{1}H NMR (600 MHz) of Compound (54)

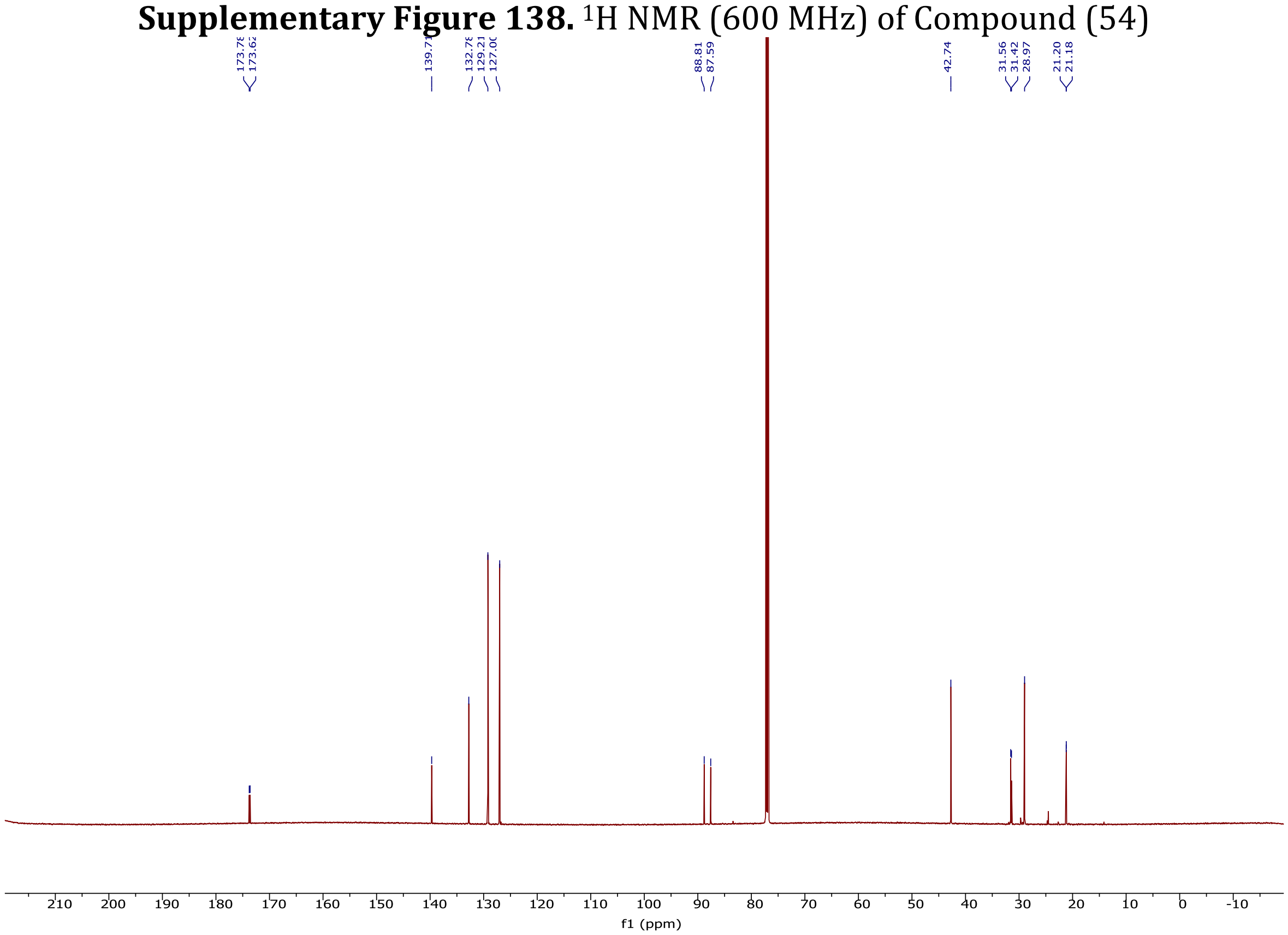

Supplementary Figure 139. ^{13}C NMR (151 MHz) of Compound (54)

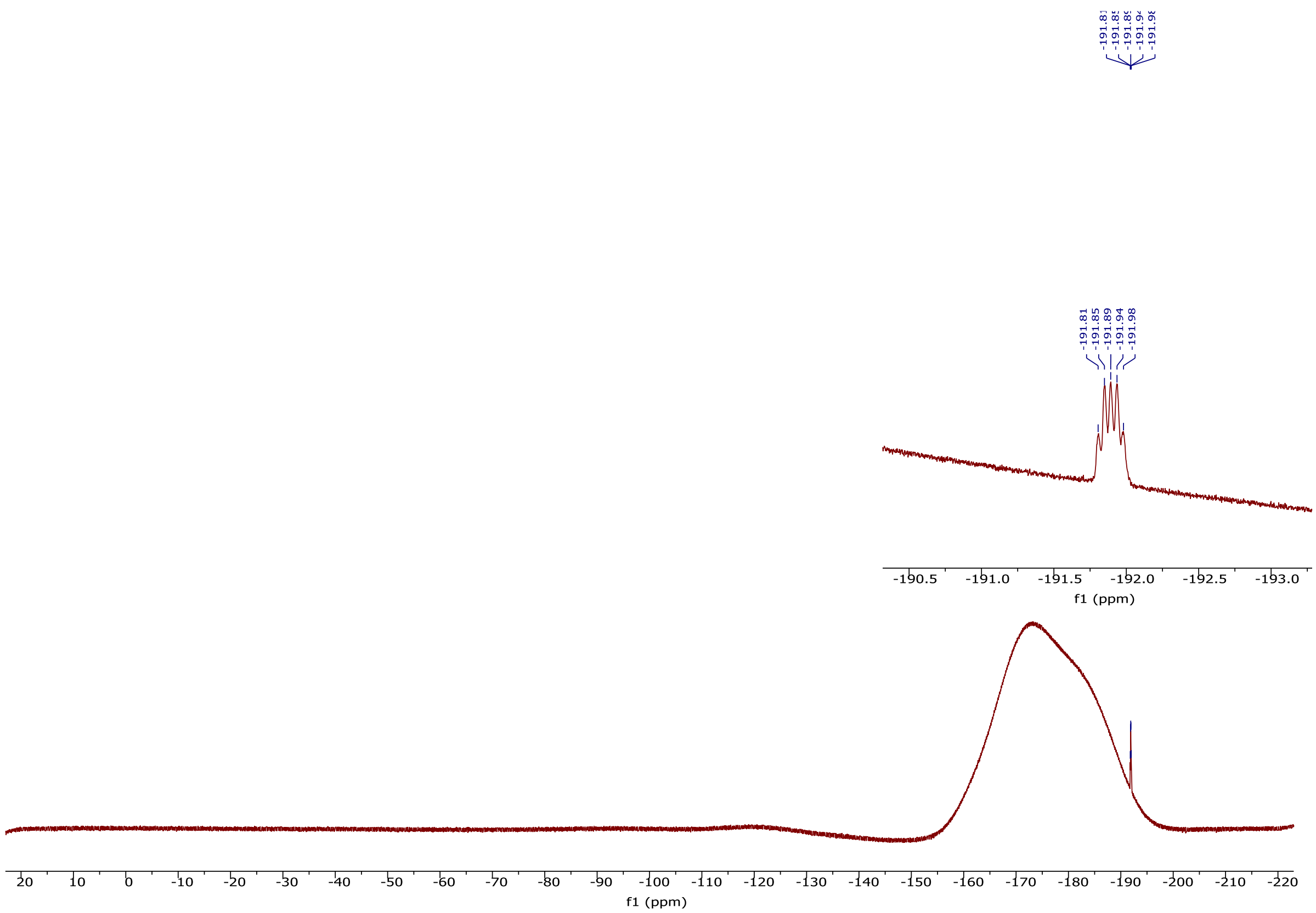

Supplementary Figure 140. ^{19}F NMR (564 MHz) of Compound (54)

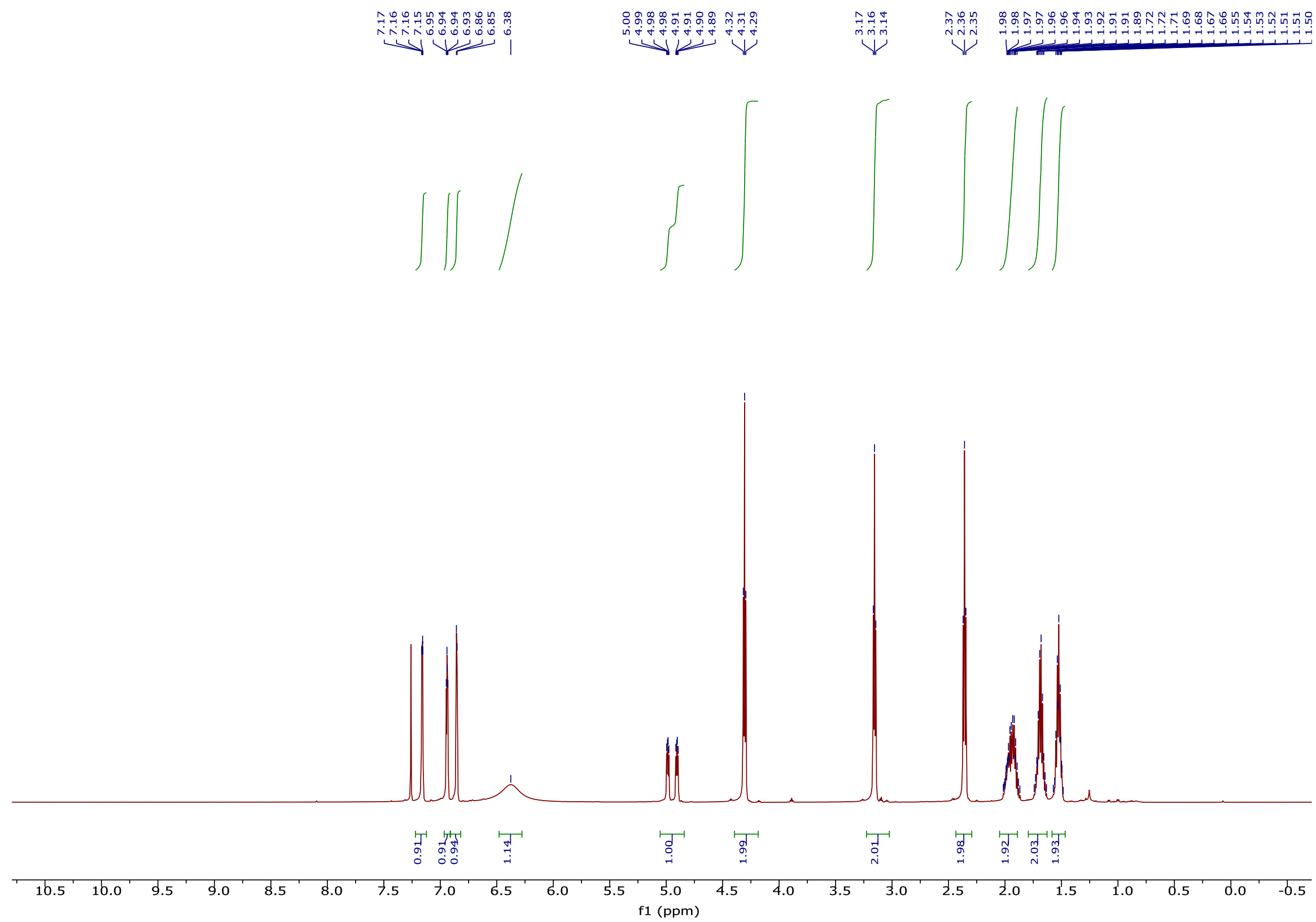

Supplementary Figure 141. ^{1}H NMR (600 MHz) of Compound (55)

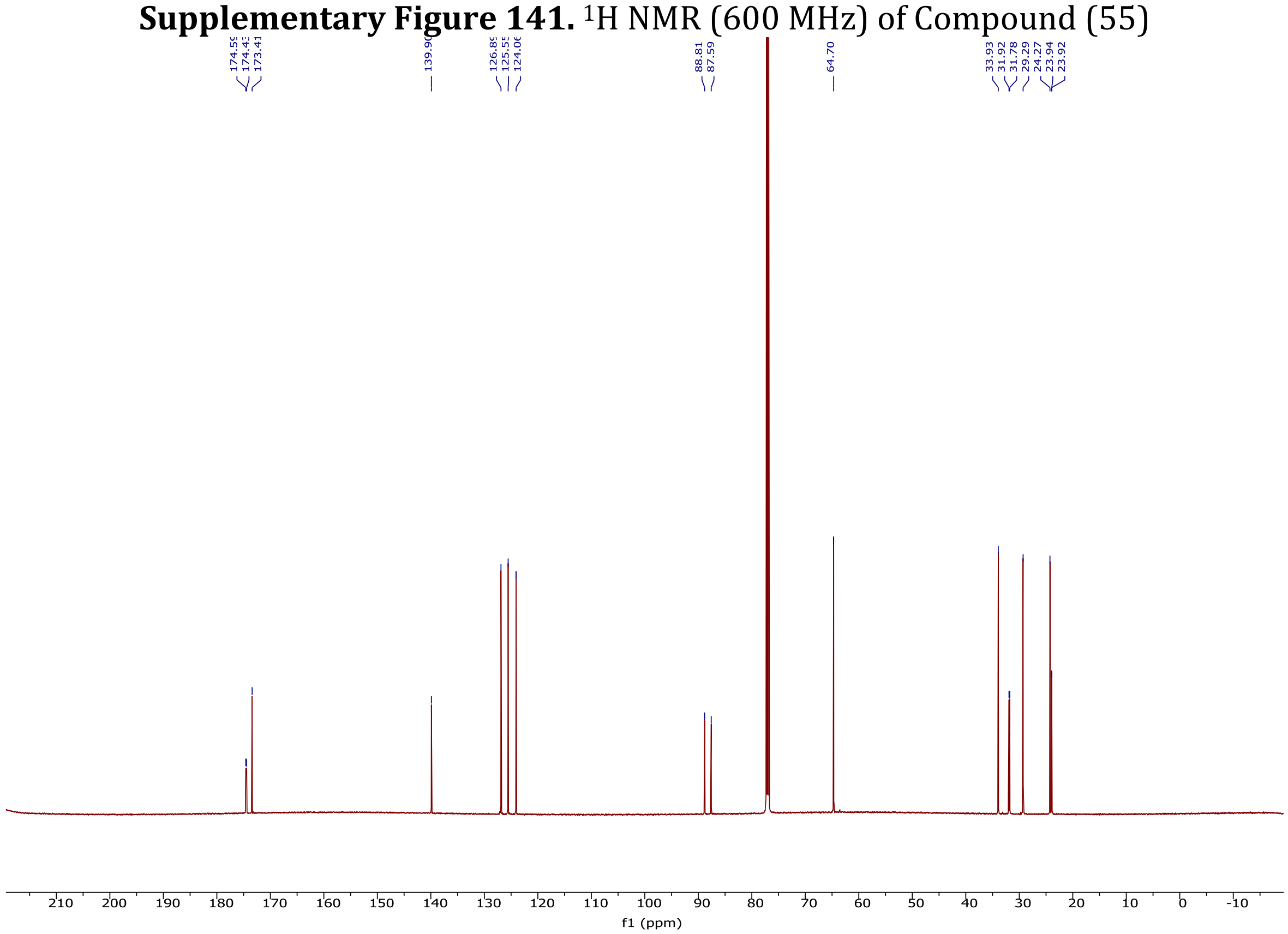

Supplementary Figure 142. ^{13}C NMR (151 MHz) of Compound (55)

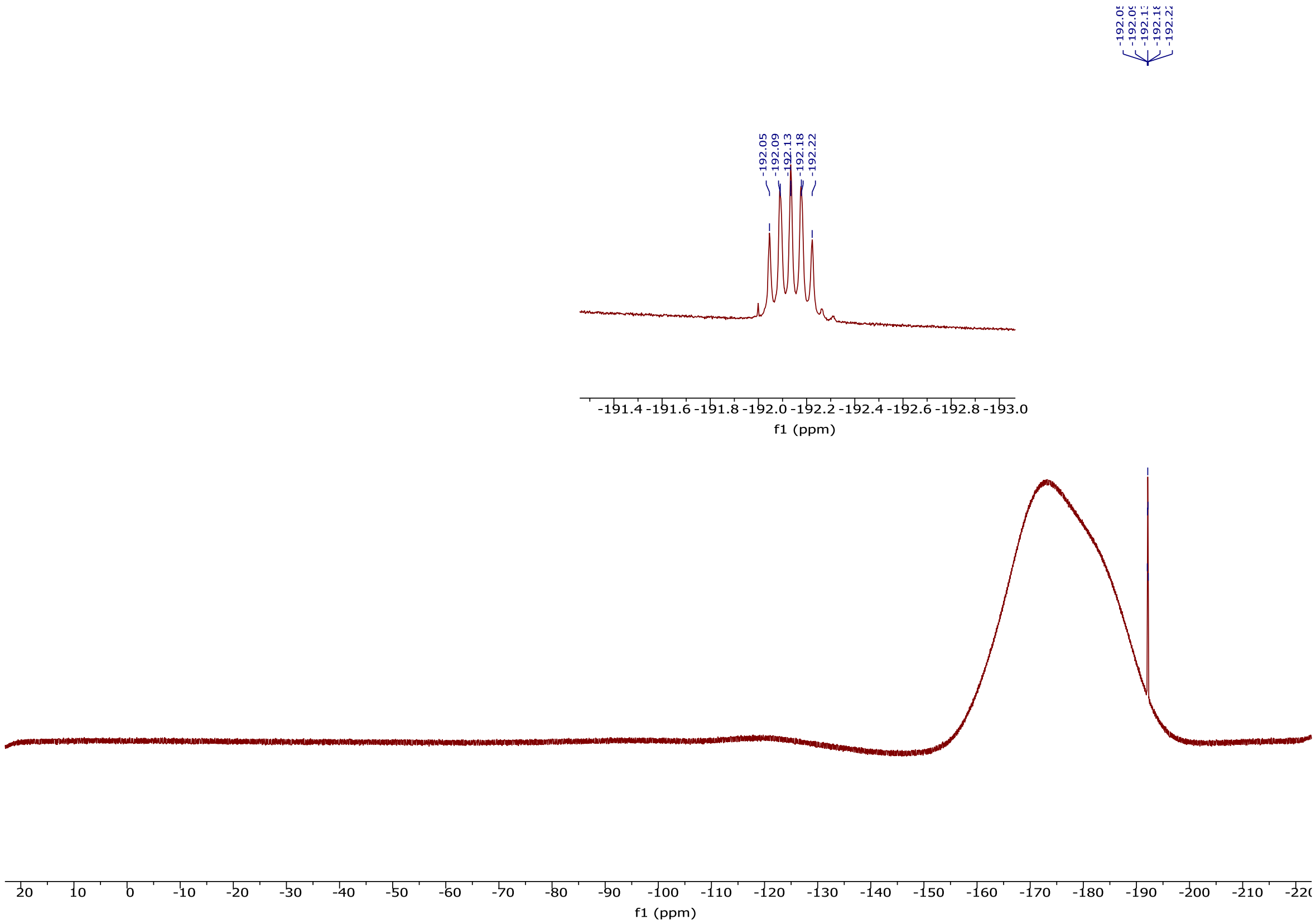

Supplementary Figure 143. ^{19}F NMR (564 MHz) of Compound (55)

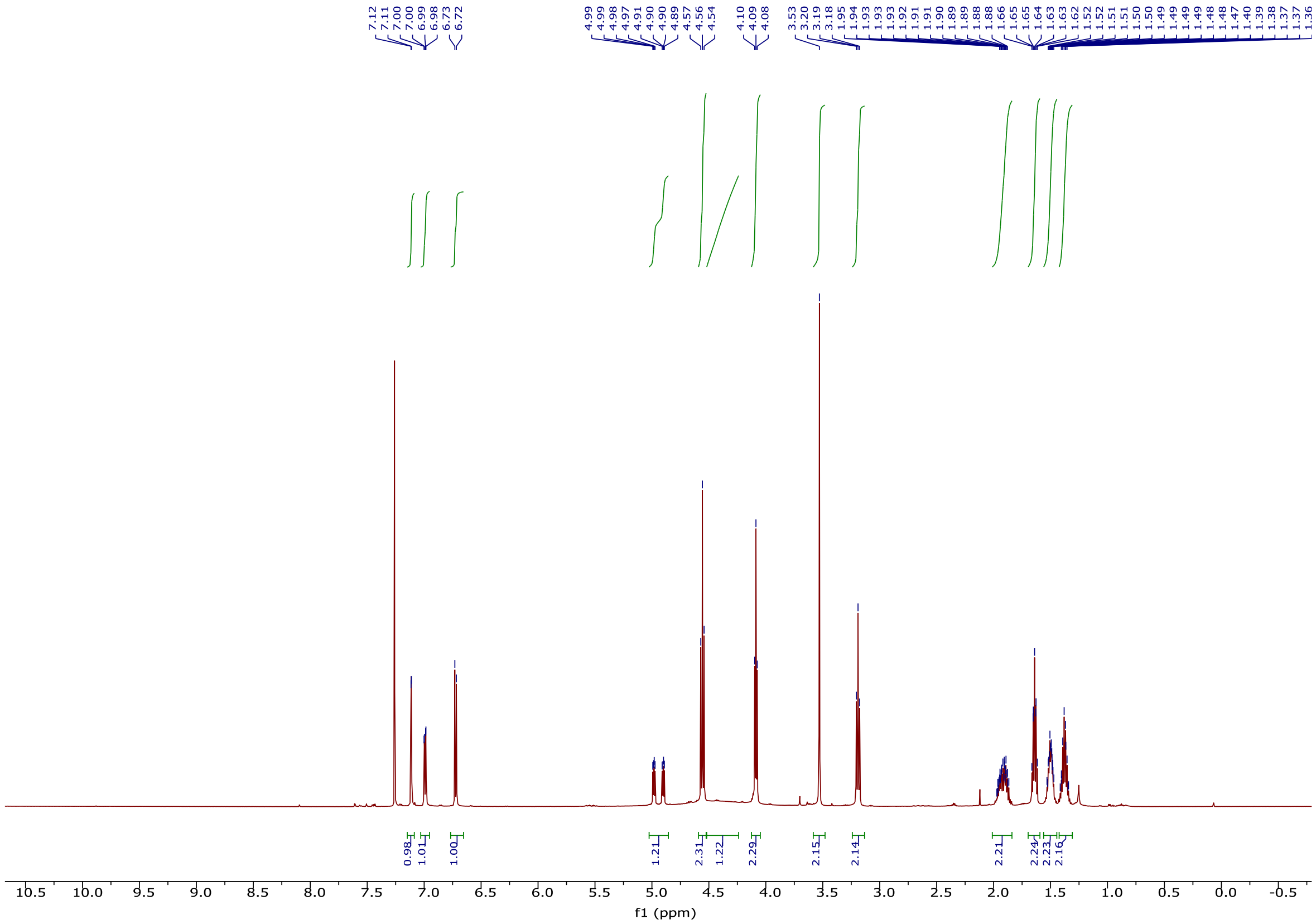

Supplementary Figure 144. ^{1}H NMR (600 MHz) of Compound (56)

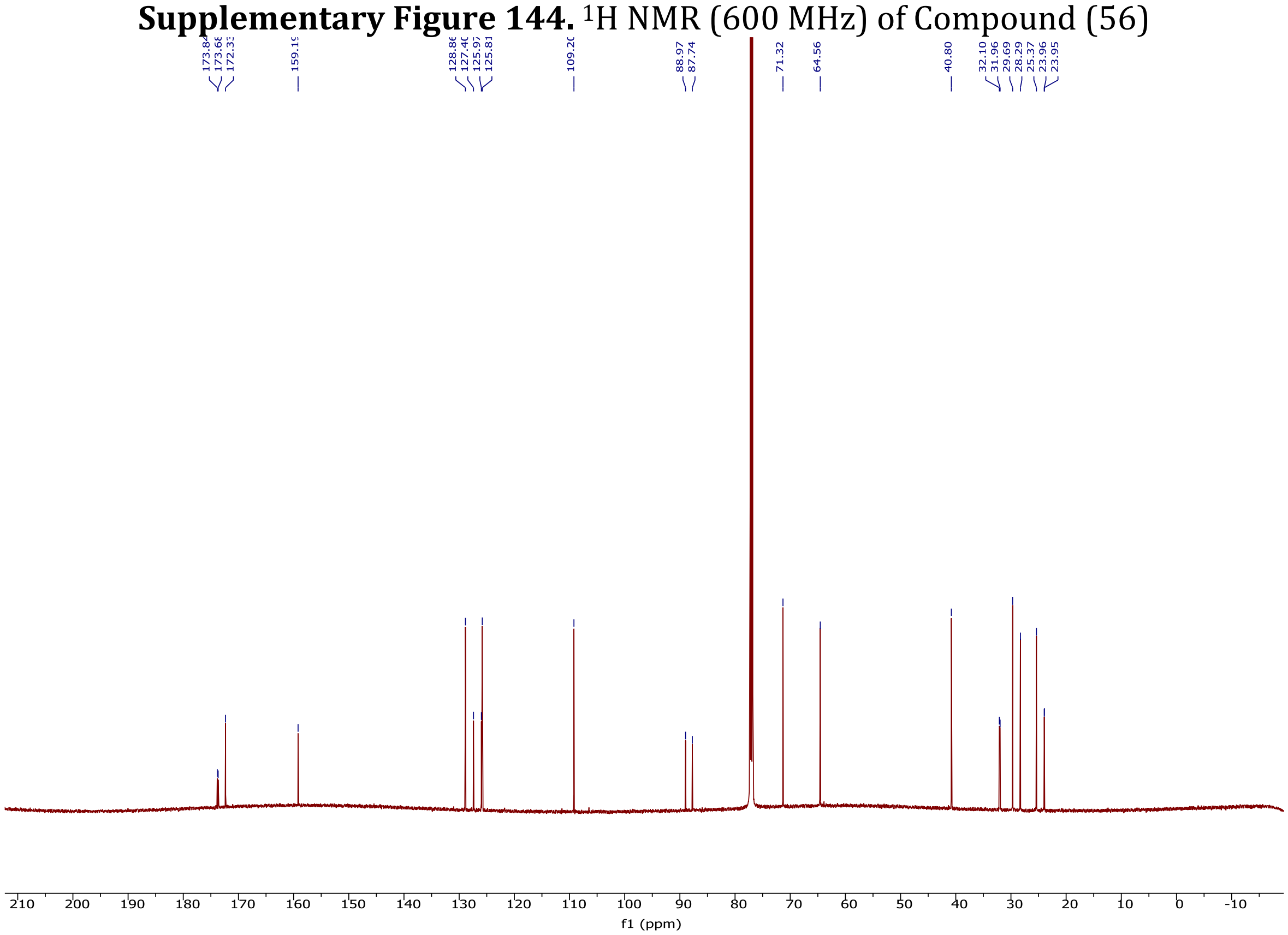

Supplementary Figure 145. ^{13}C NMR (151 MHz) of Compound (56)

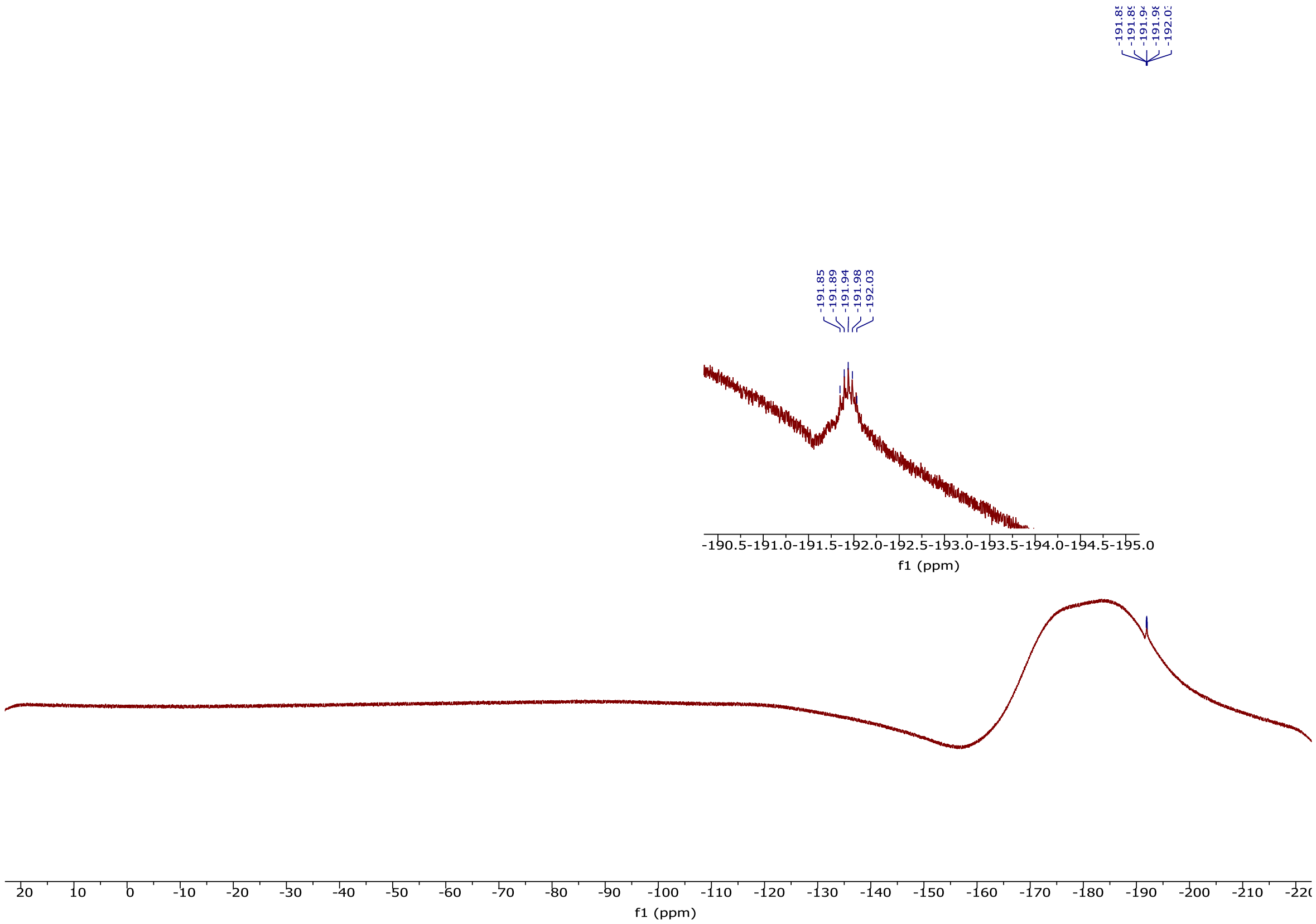

Supplementary Figure 146. ^{19}F NMR (564 MHz) of Compound (56)

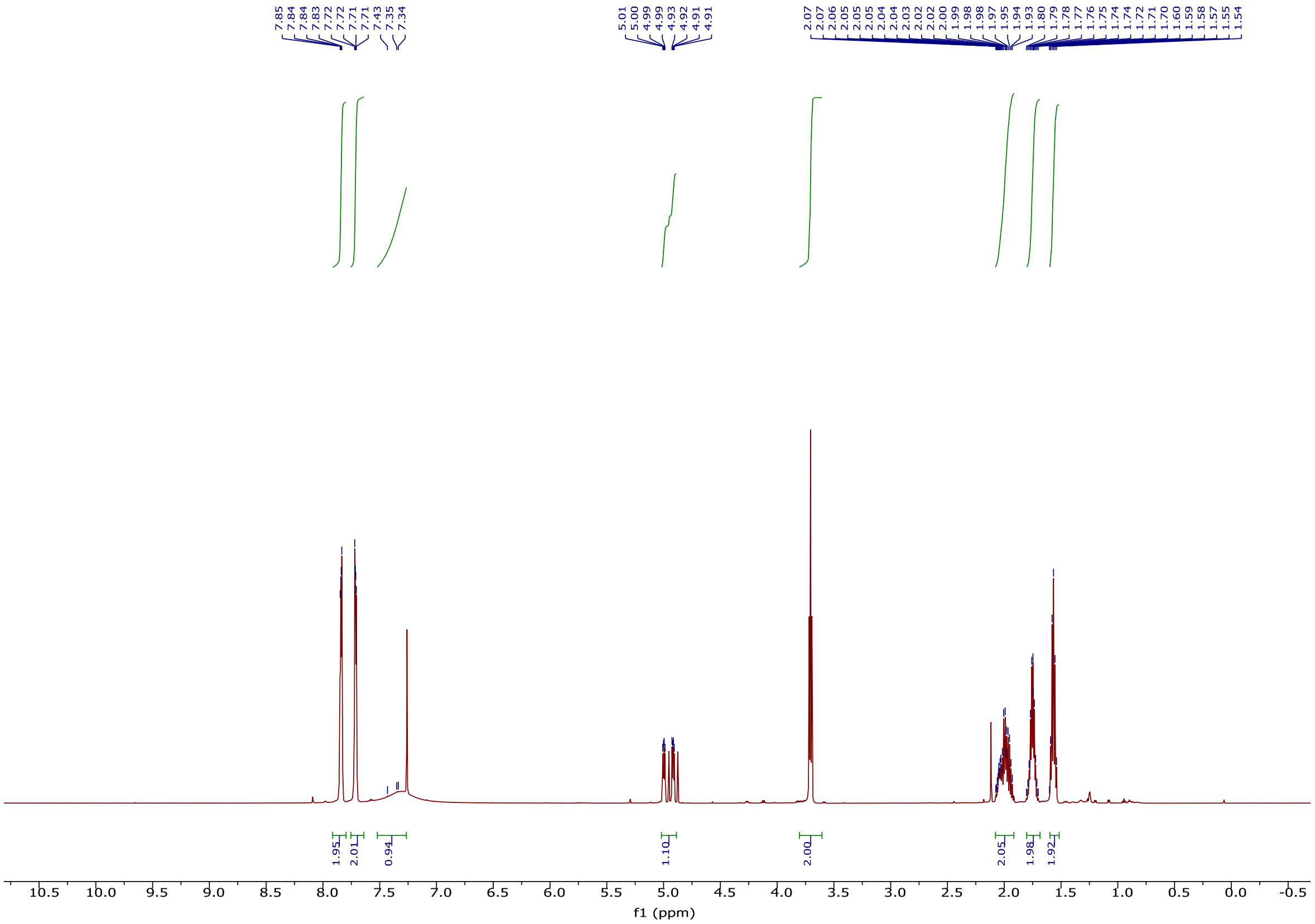

Supplementary Figure 147. ^{1}H NMR (600 MHz) of Compound (57)

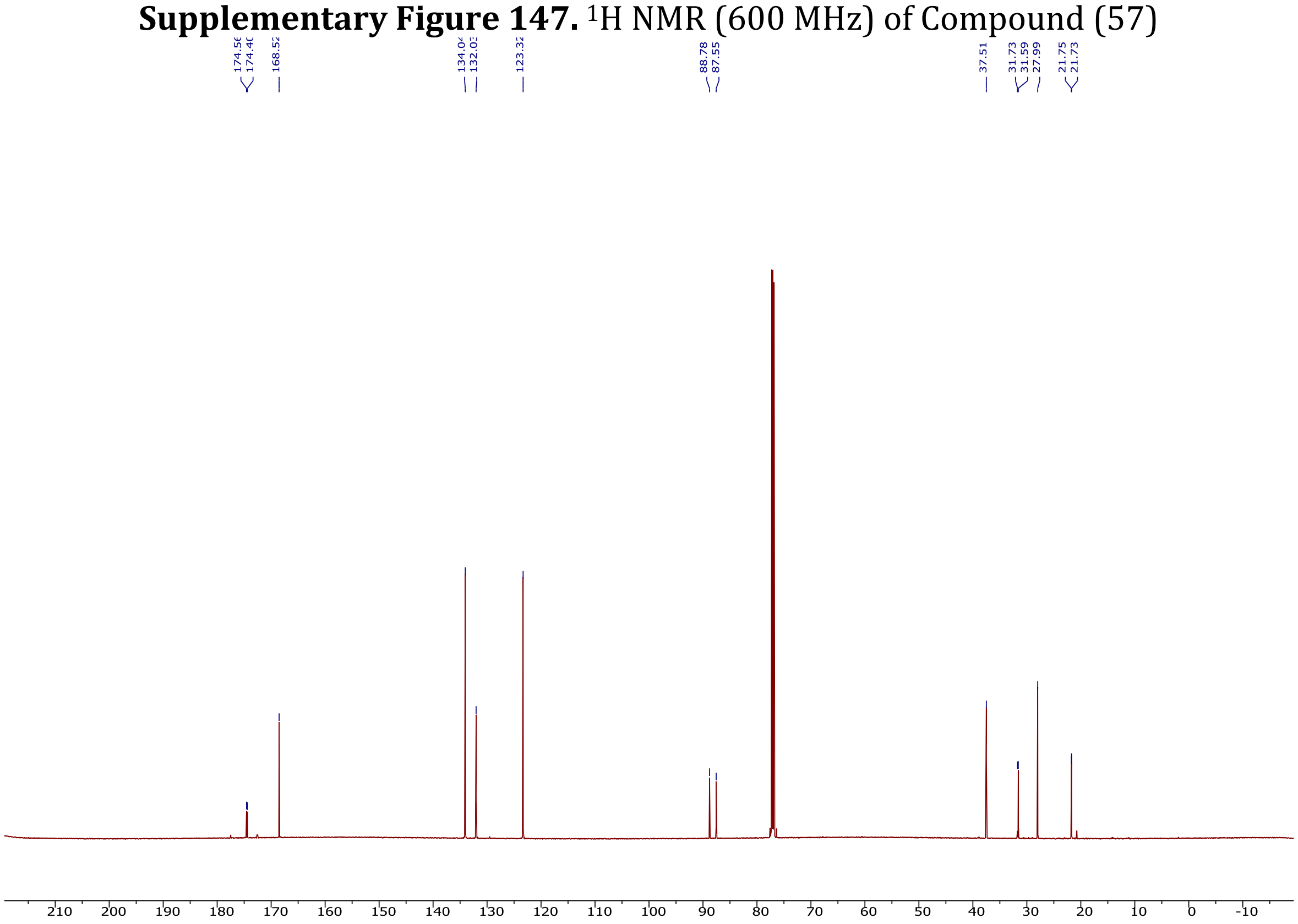

Supplementary Figure 148. ^{13}C NMR (151 MHz) of Compound (57)

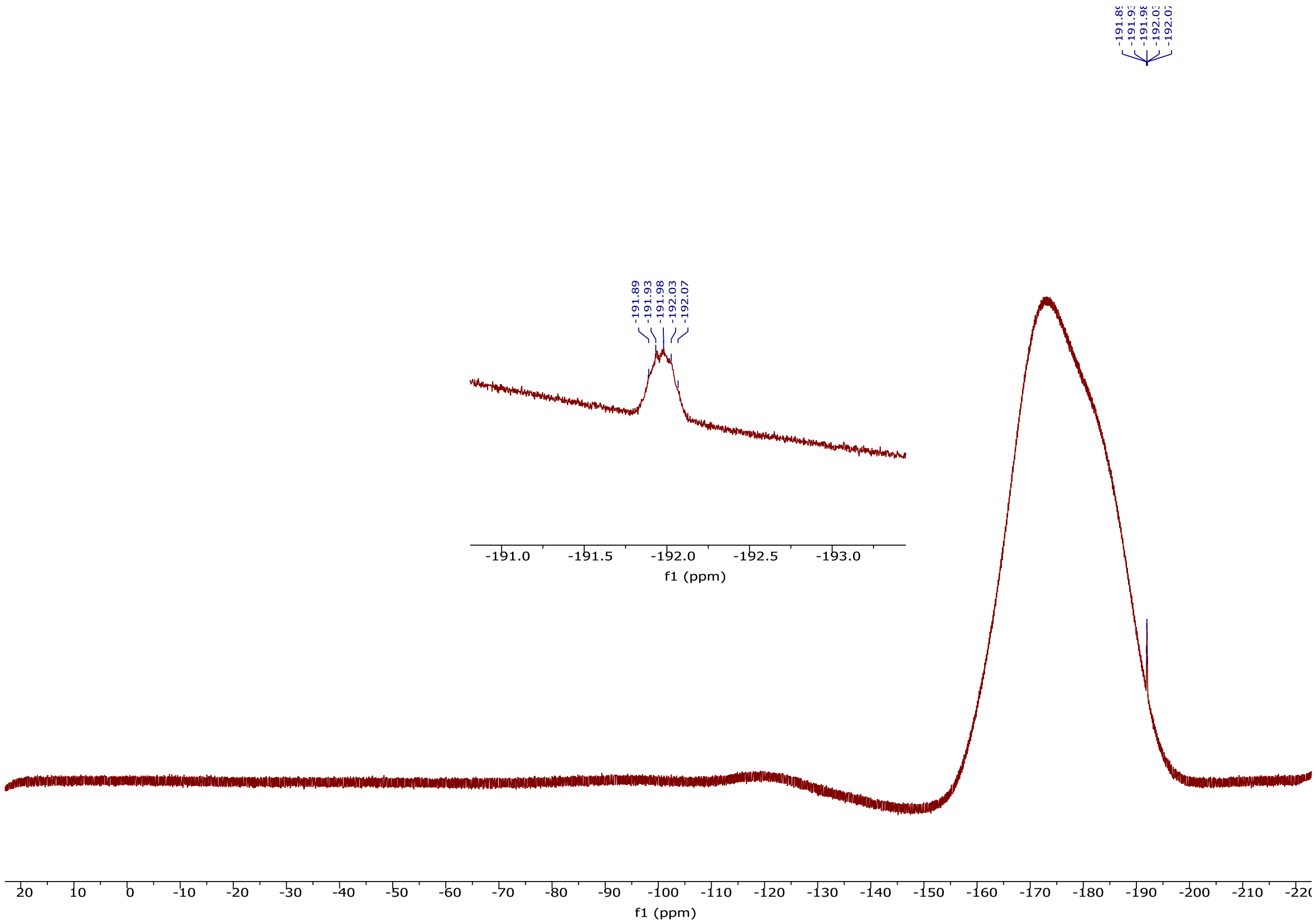

Supplementary Figure 149. ^{19}F NMR (564 MHz) of Compound (57)

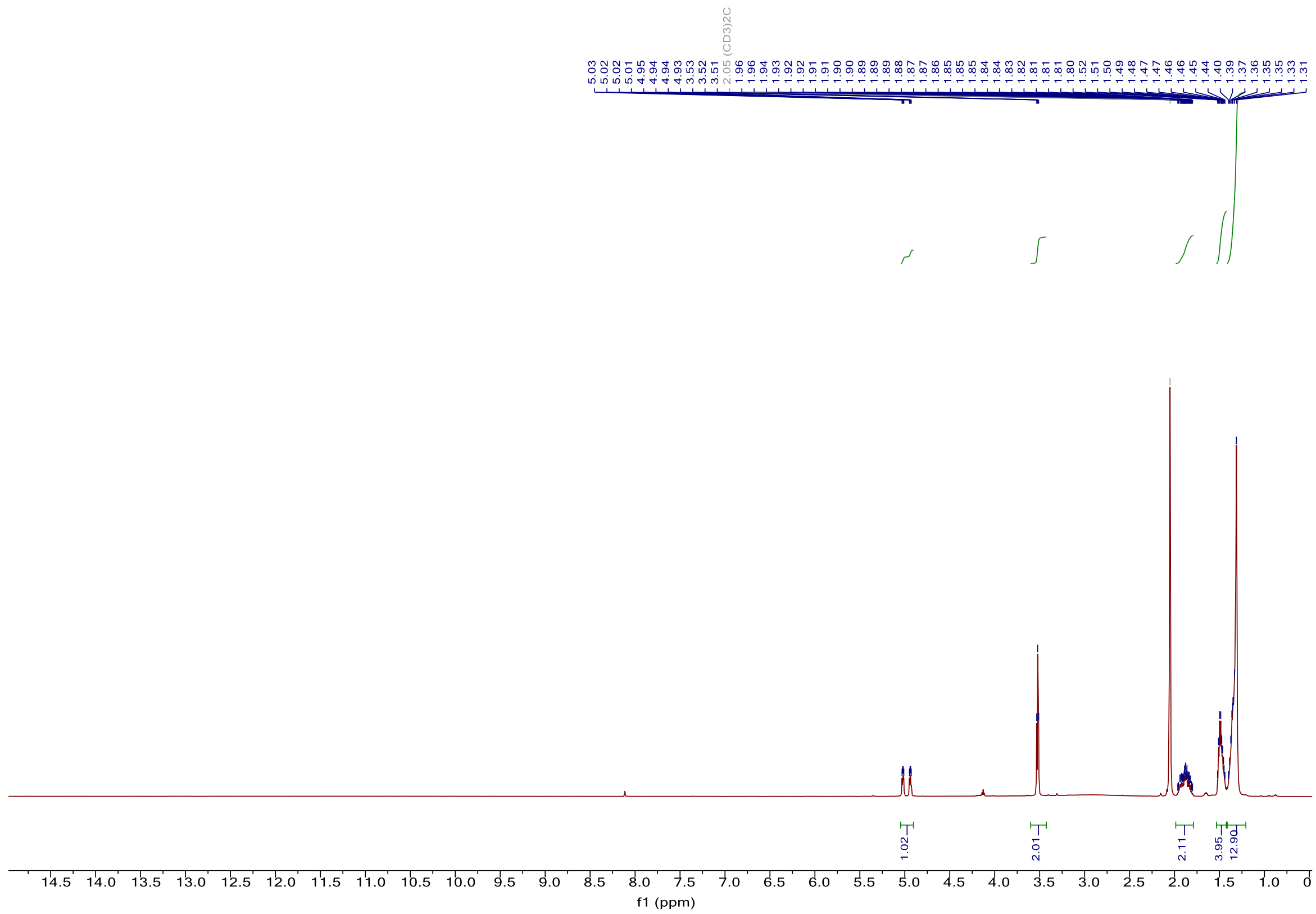

Supplementary Figure 150. ^{1}H NMR (600 MHz) of Compound (58)

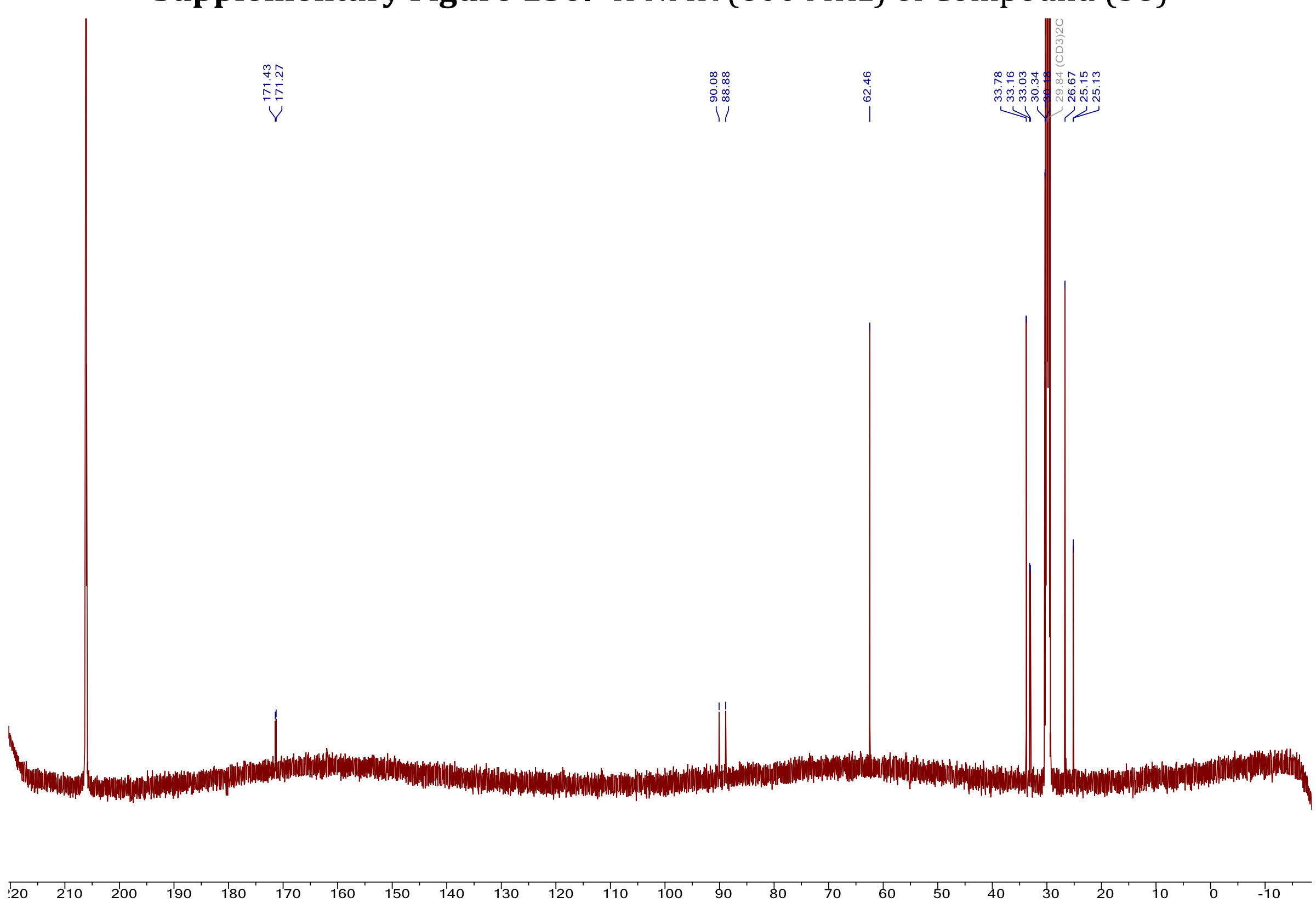

Supplementary Figure 151. ^{13}C NMR (151 MHz) of Compound (58)

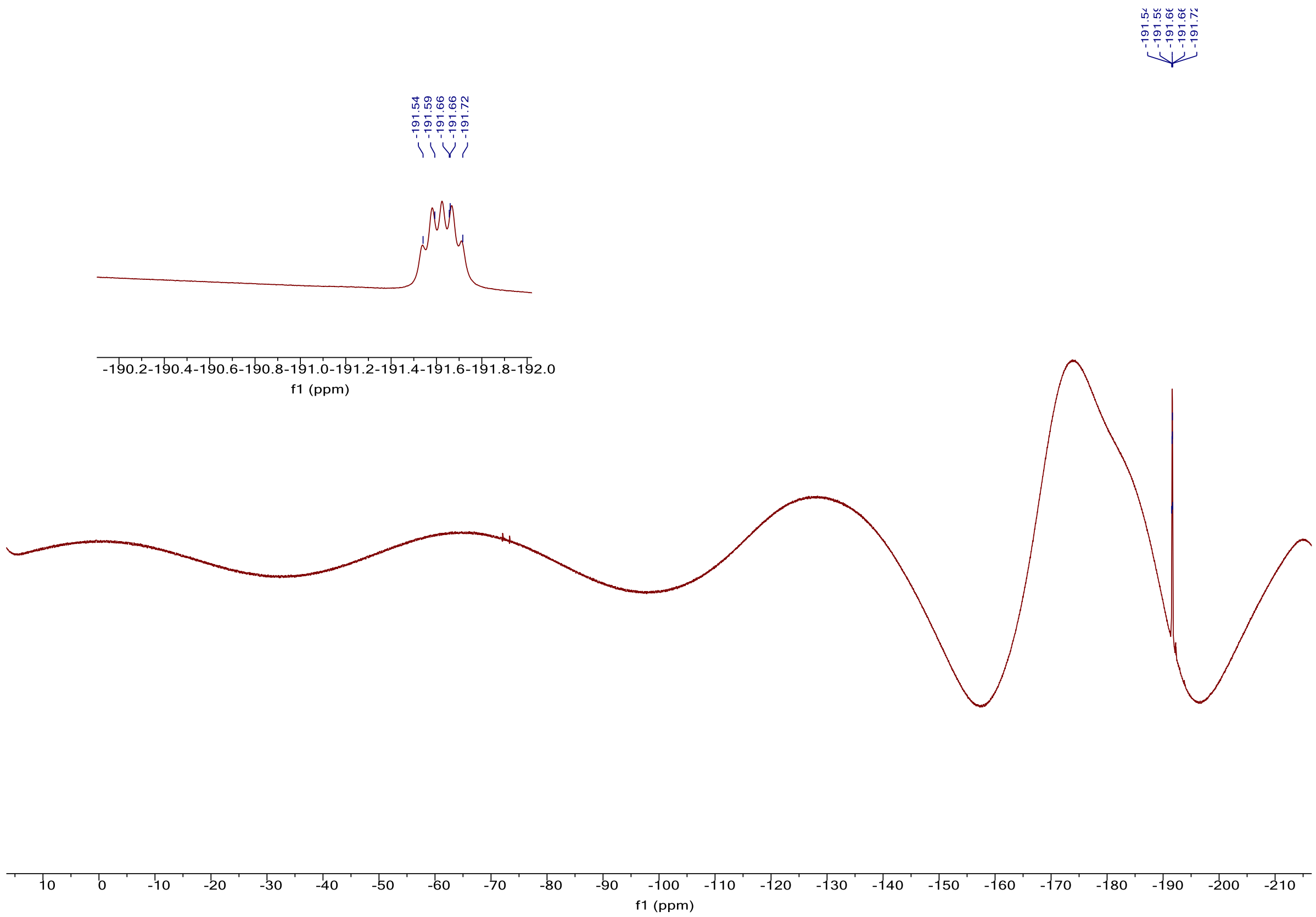

Supplementary Figure 152. ^{19}F NMR (564 MHz) of Compound (58)

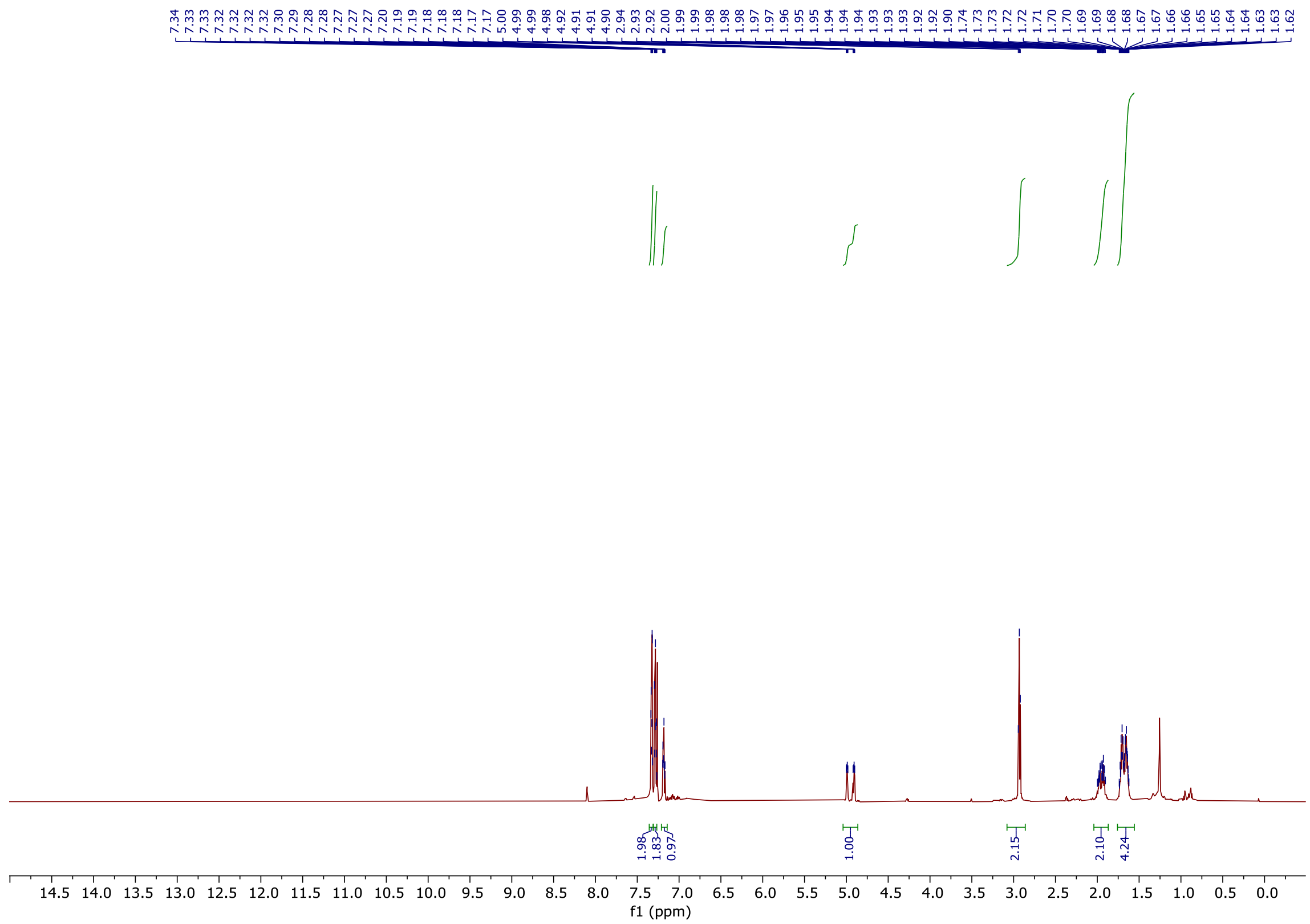

Supplementary Figure 153. ^{1}H NMR (600 MHz) of Compound (59)

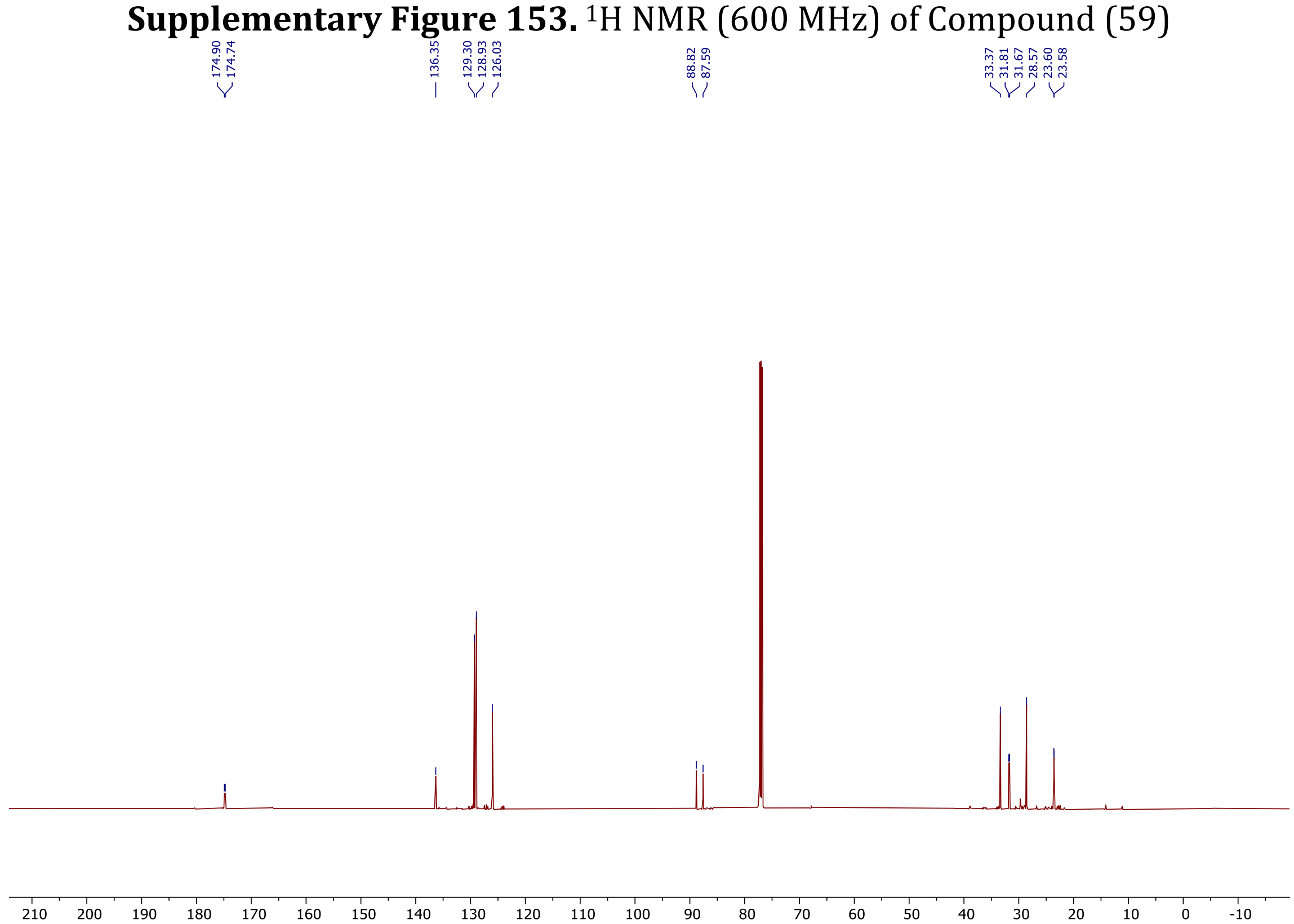

Supplementary Figure 154. ^{13}C NMR (151 MHz) of Compound (59)

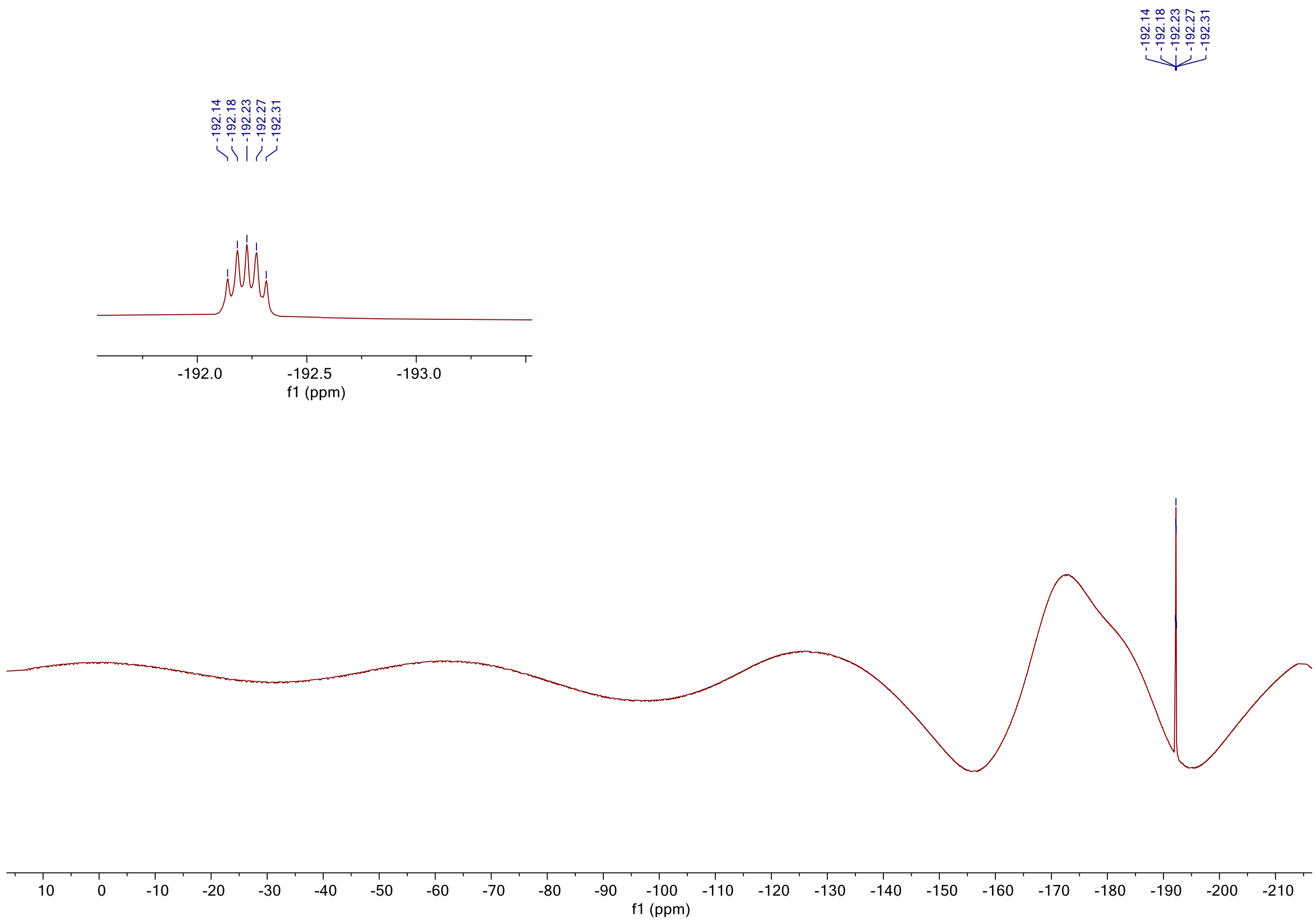

Supplementary Figure 155. ^{19}F NMR (564 MHz) of Compound (59)

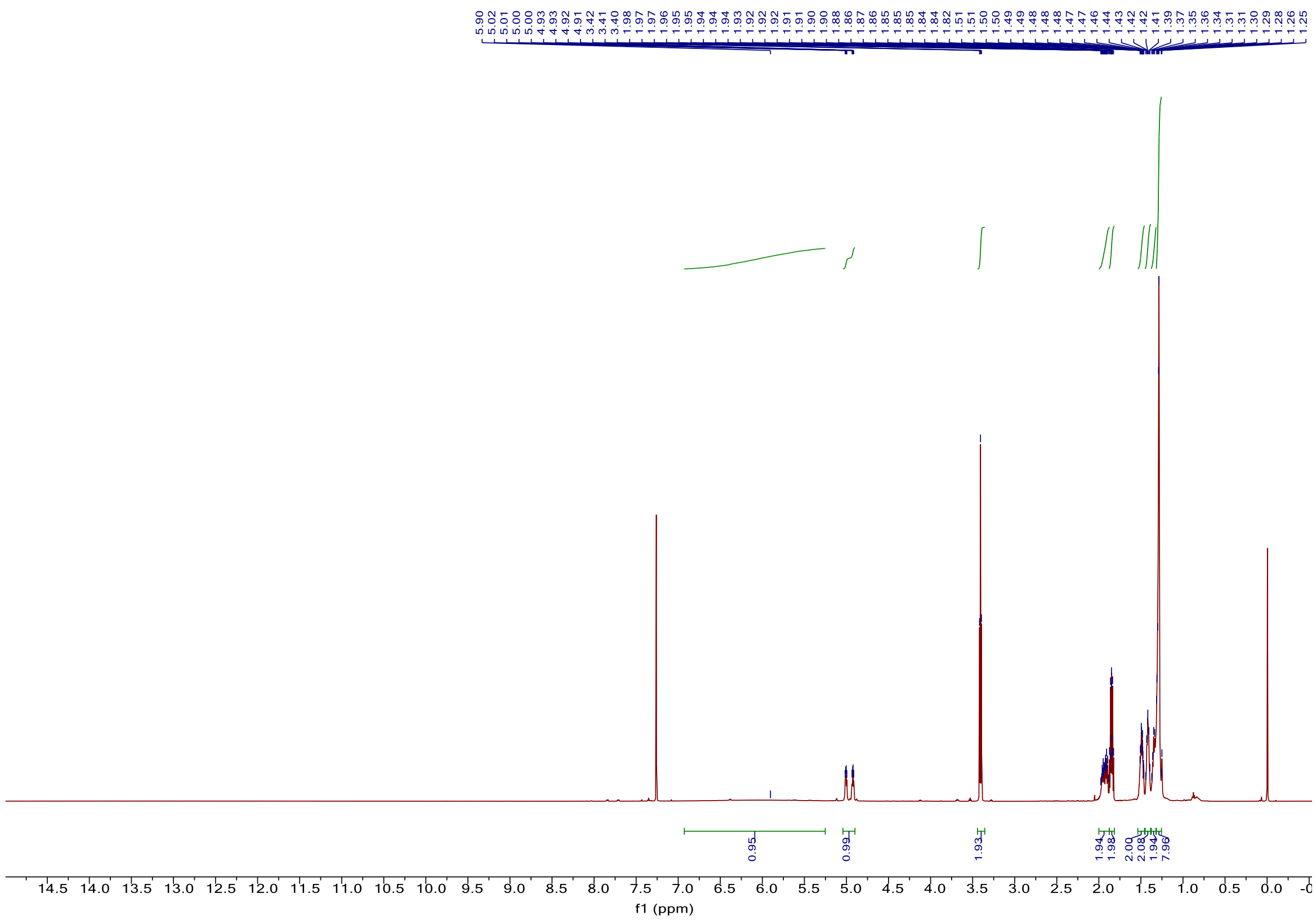

Supplementary Figure 156. ^{1}H NMR (600 MHz) of Compound (60)

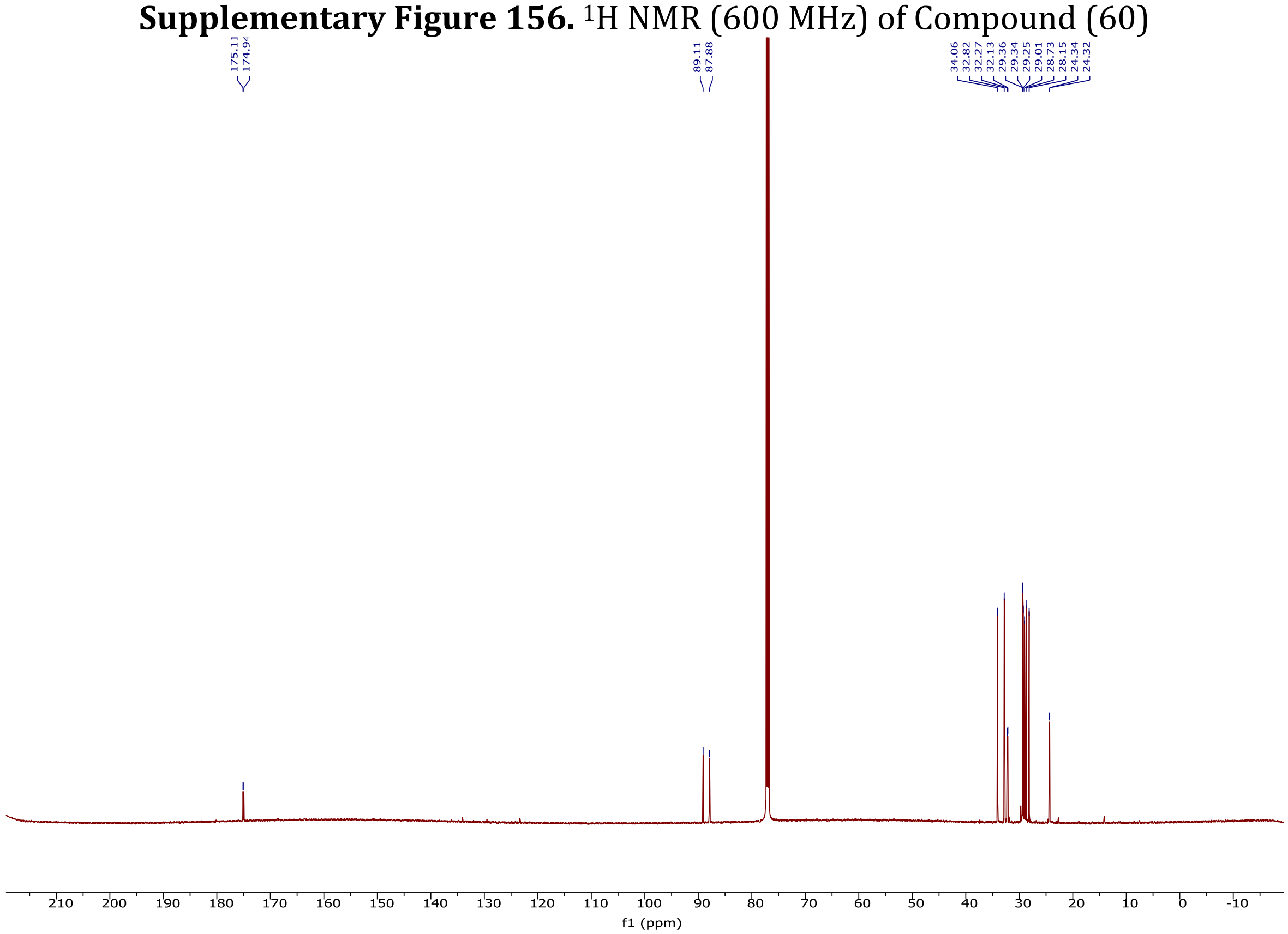

Supplementary Figure 157. ^{13}C NMR (151 MHz) of Compound (60)

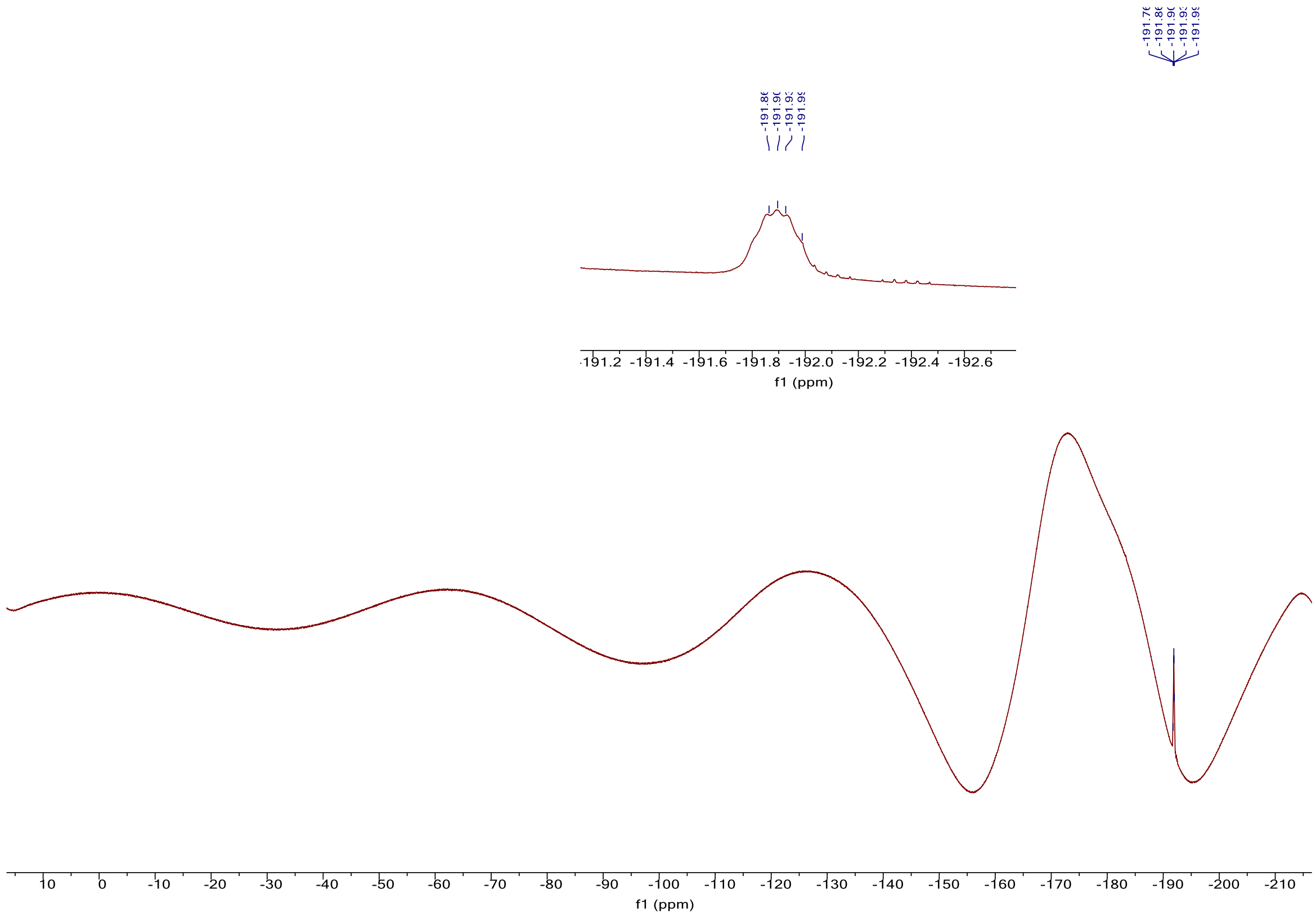

Supplementary Figure 158. ^{19}F NMR (564 MHz) of Compound (60)

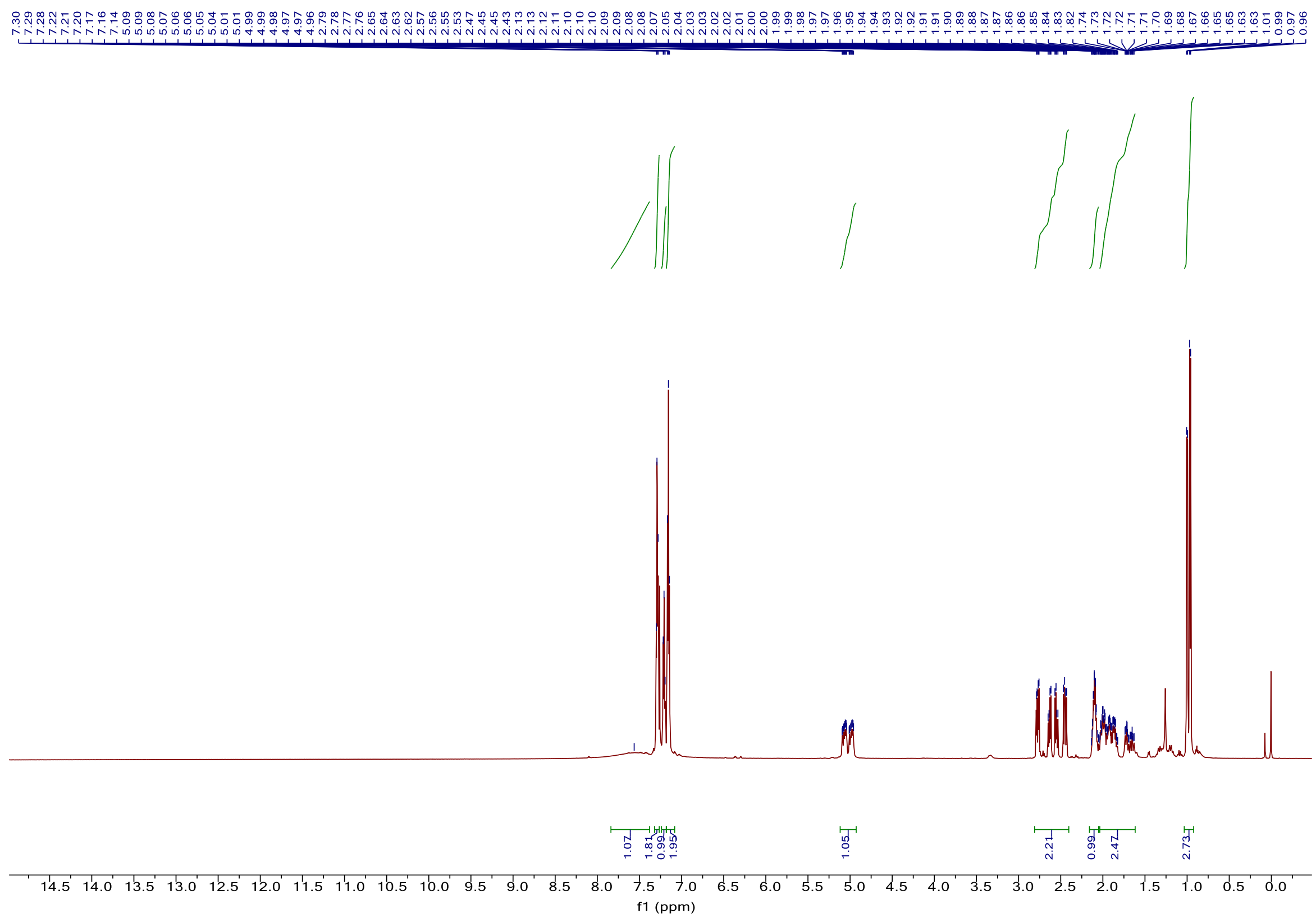

Supplementary Figure 159. ^{1}H NMR (600 MHz) of Compound (61)

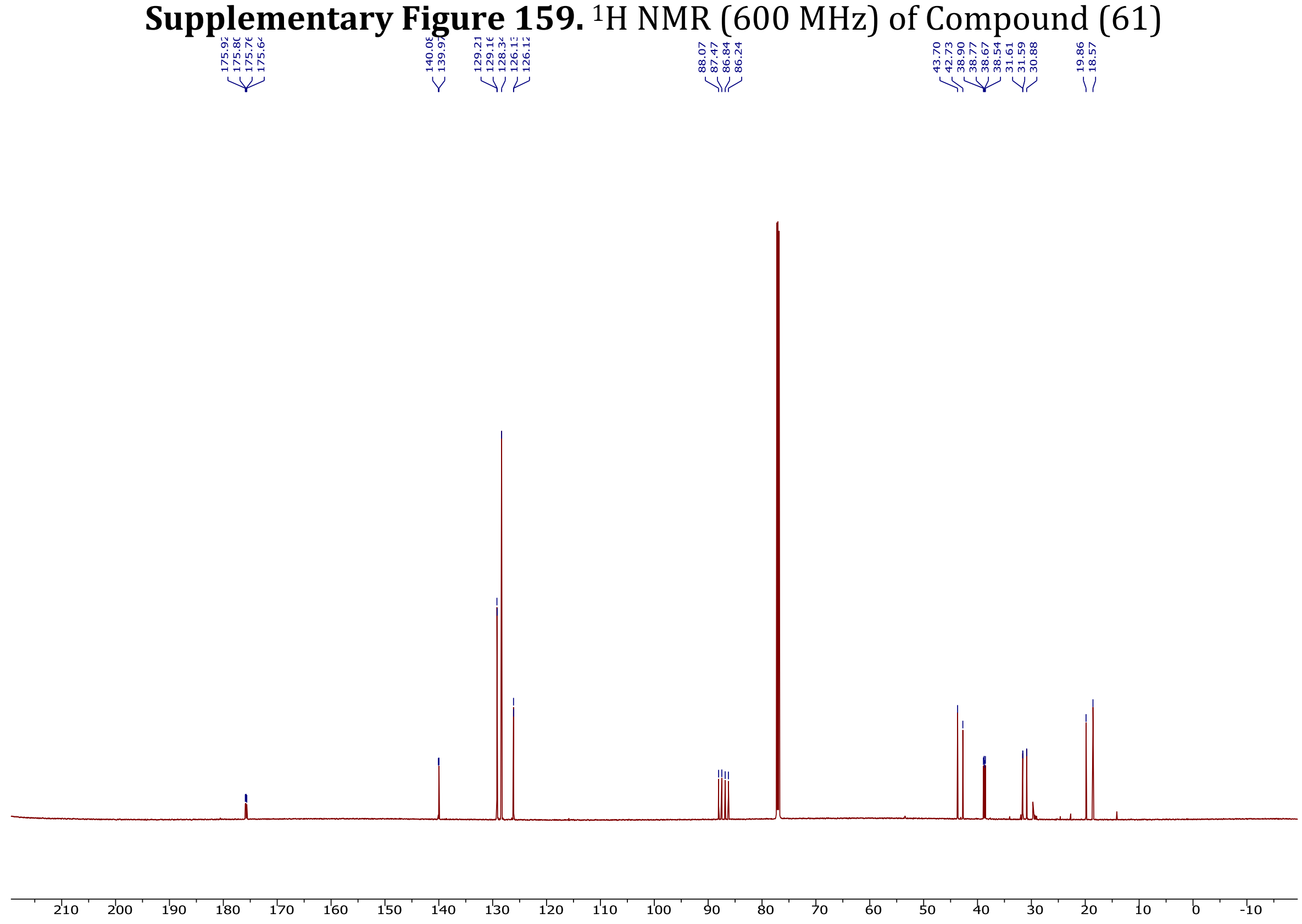

Supplementary Figure 160. ^{13}C NMR (151 MHz) of Compound (61)

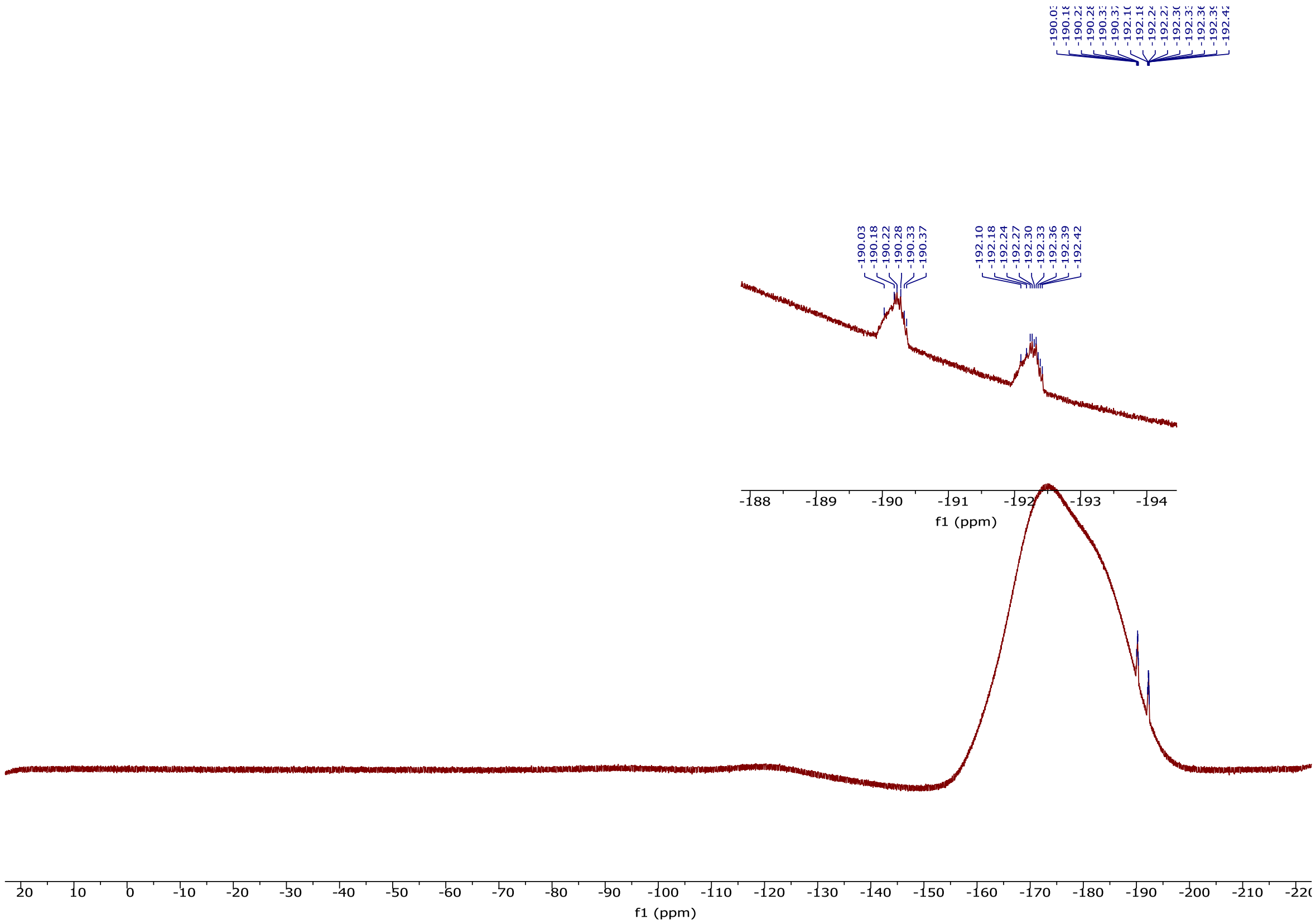

Supplementary Figure 161. ^{19}F NMR (564 MHz) of Compound (61)

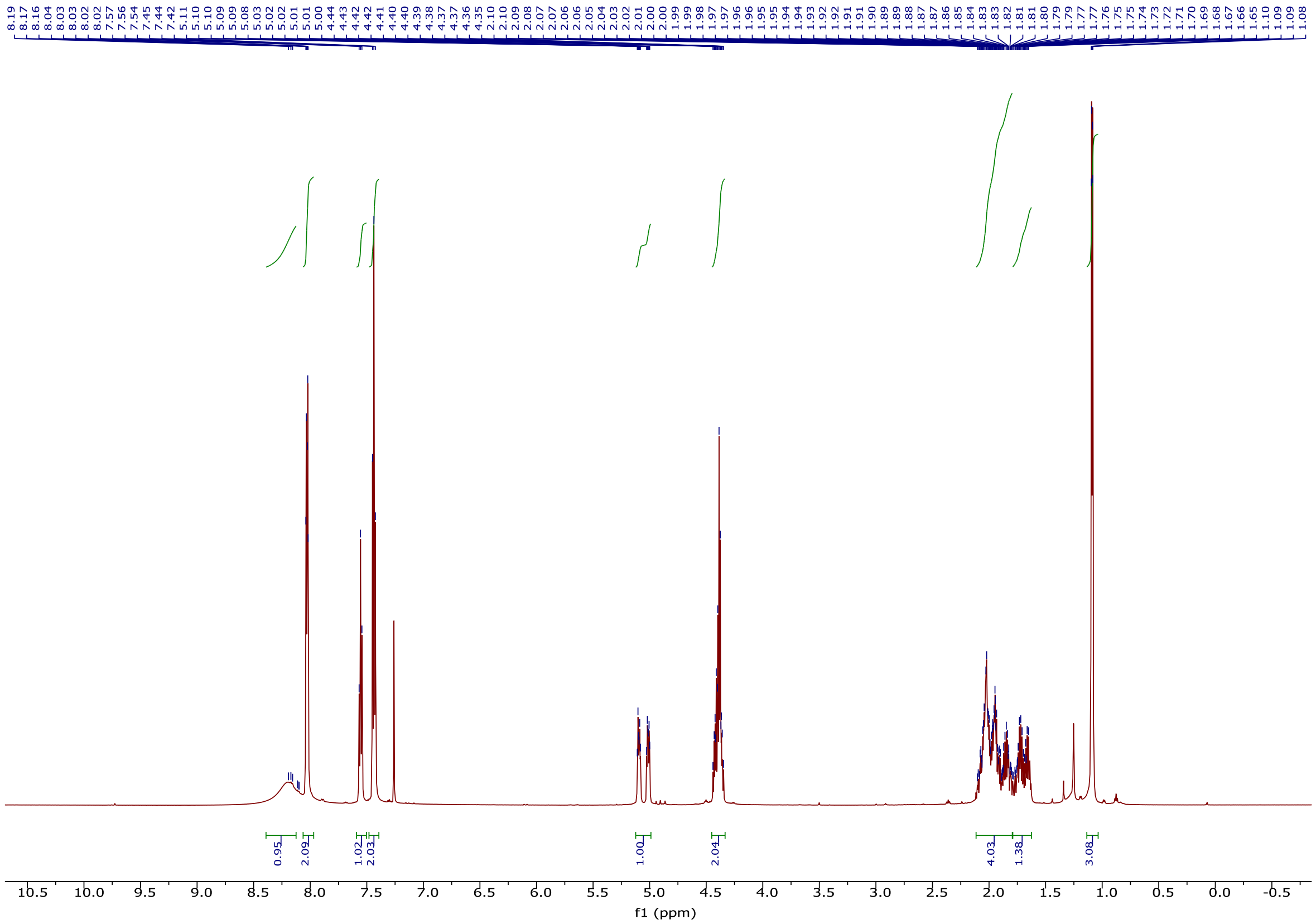

Supplementary Figure 162. ^{1}H NMR (600 MHz) of Compound (62)

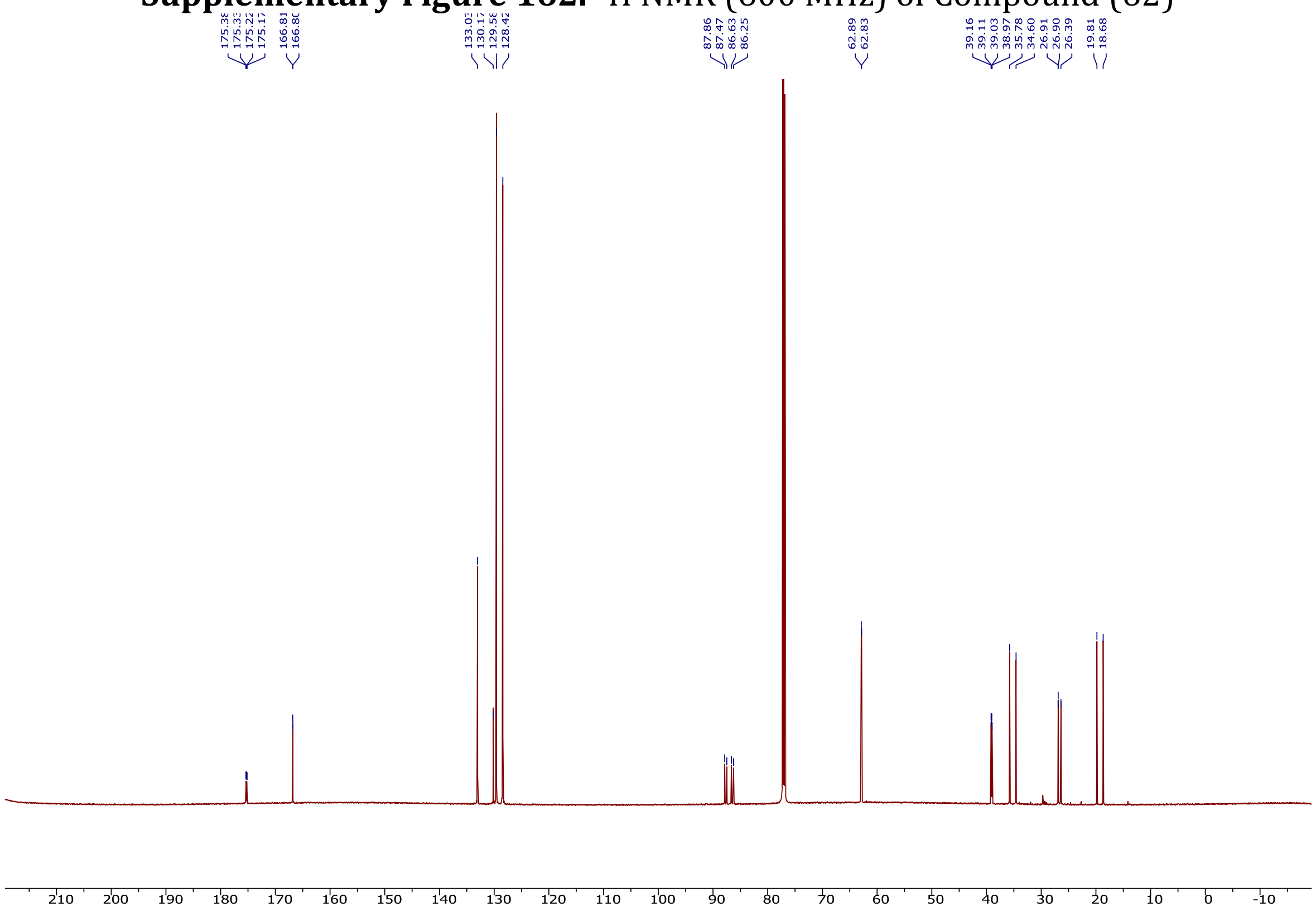

Supplementary Figure 163. ^{13}C NMR (151 MHz) of Compound (62)

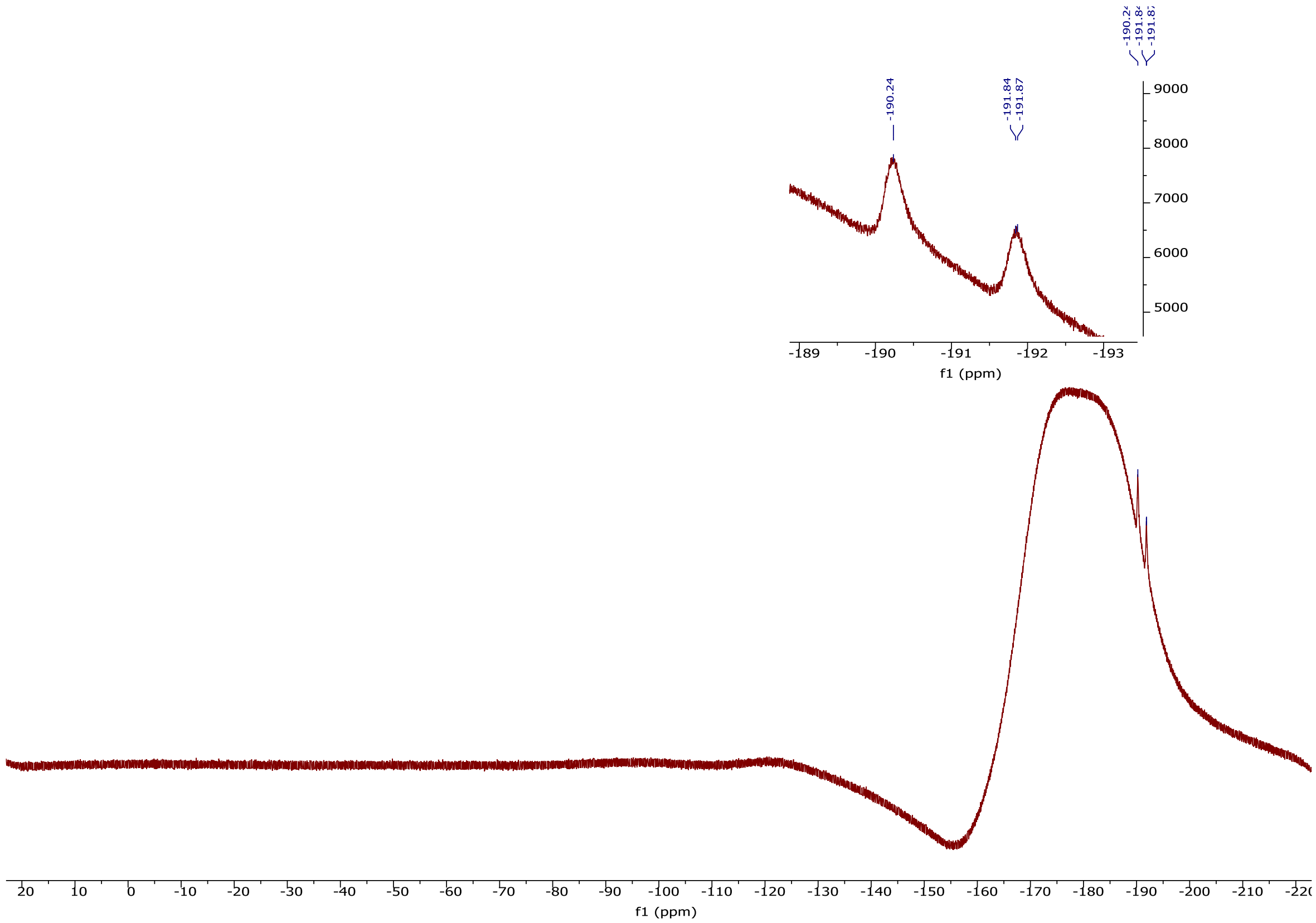

Supplementary Figure 164. ^{19}F NMR (564 MHz) of Compound (62)

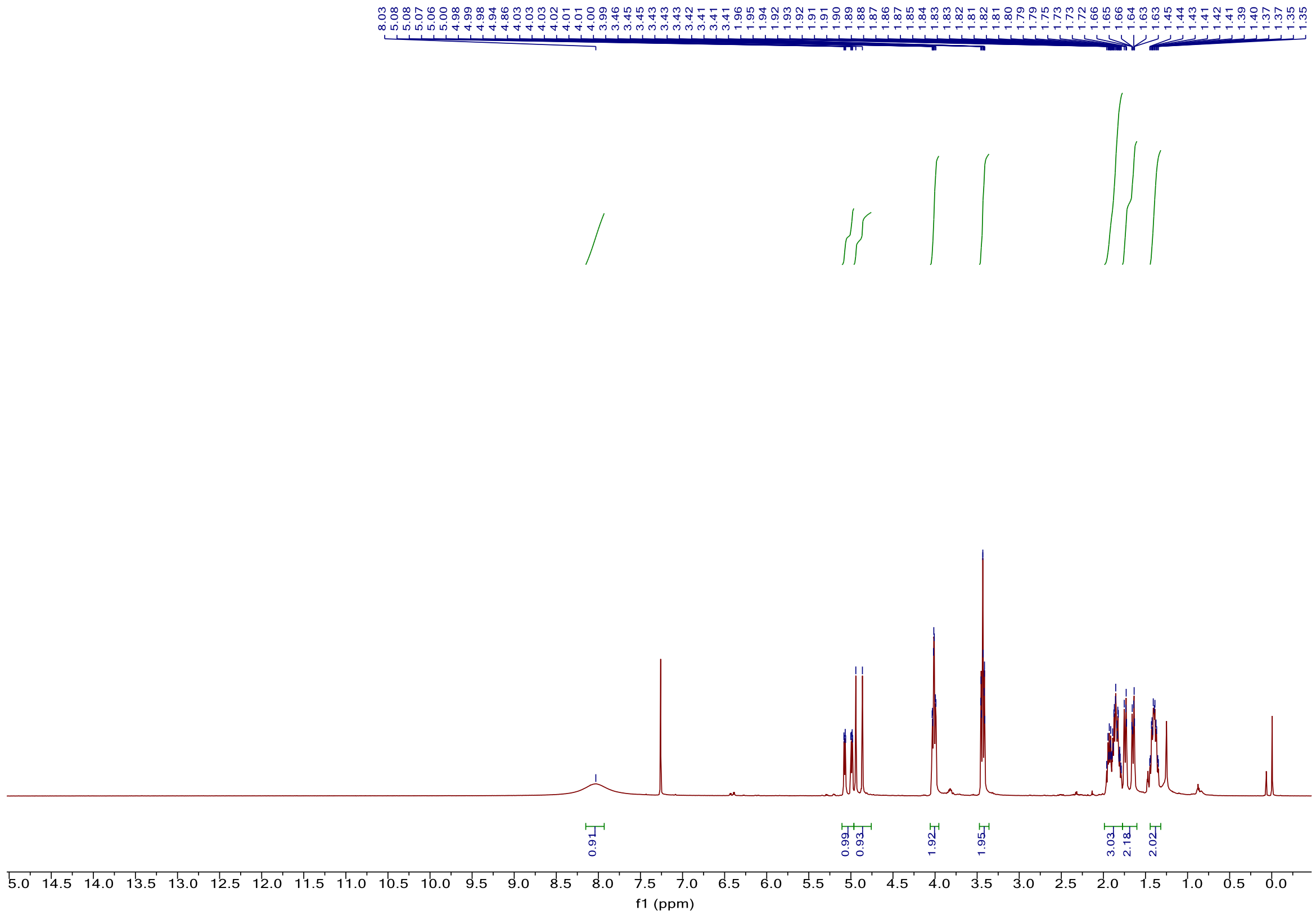

Supplementary Figure 165. ^{1}H NMR (600 MHz) of Compound (63)

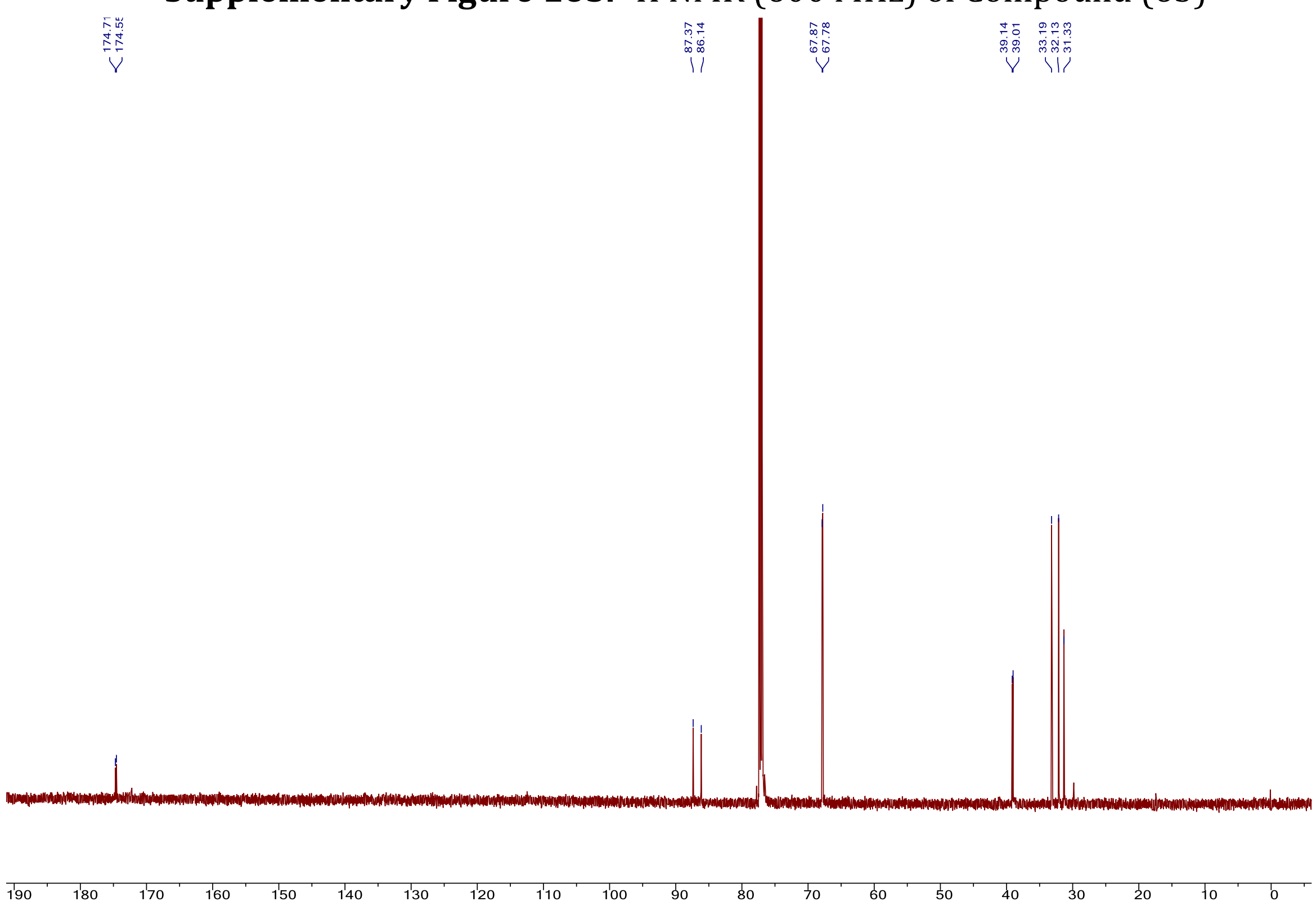

Supplementary Figure 166. ^{13}C NMR (151 MHz) of Compound (63)

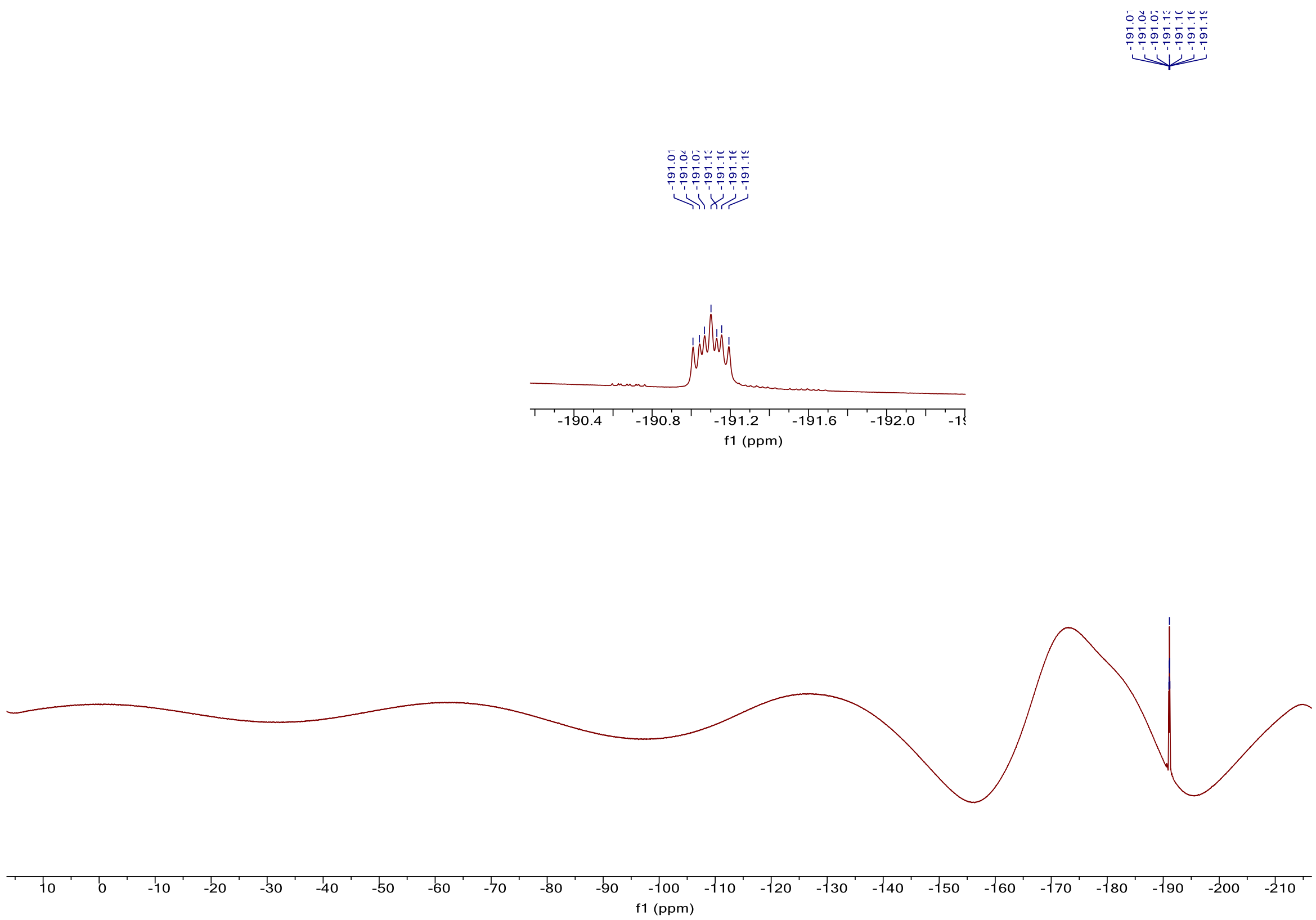

Supplementary Figure 167. ^{19}F NMR (564 MHz) of Compound (63)

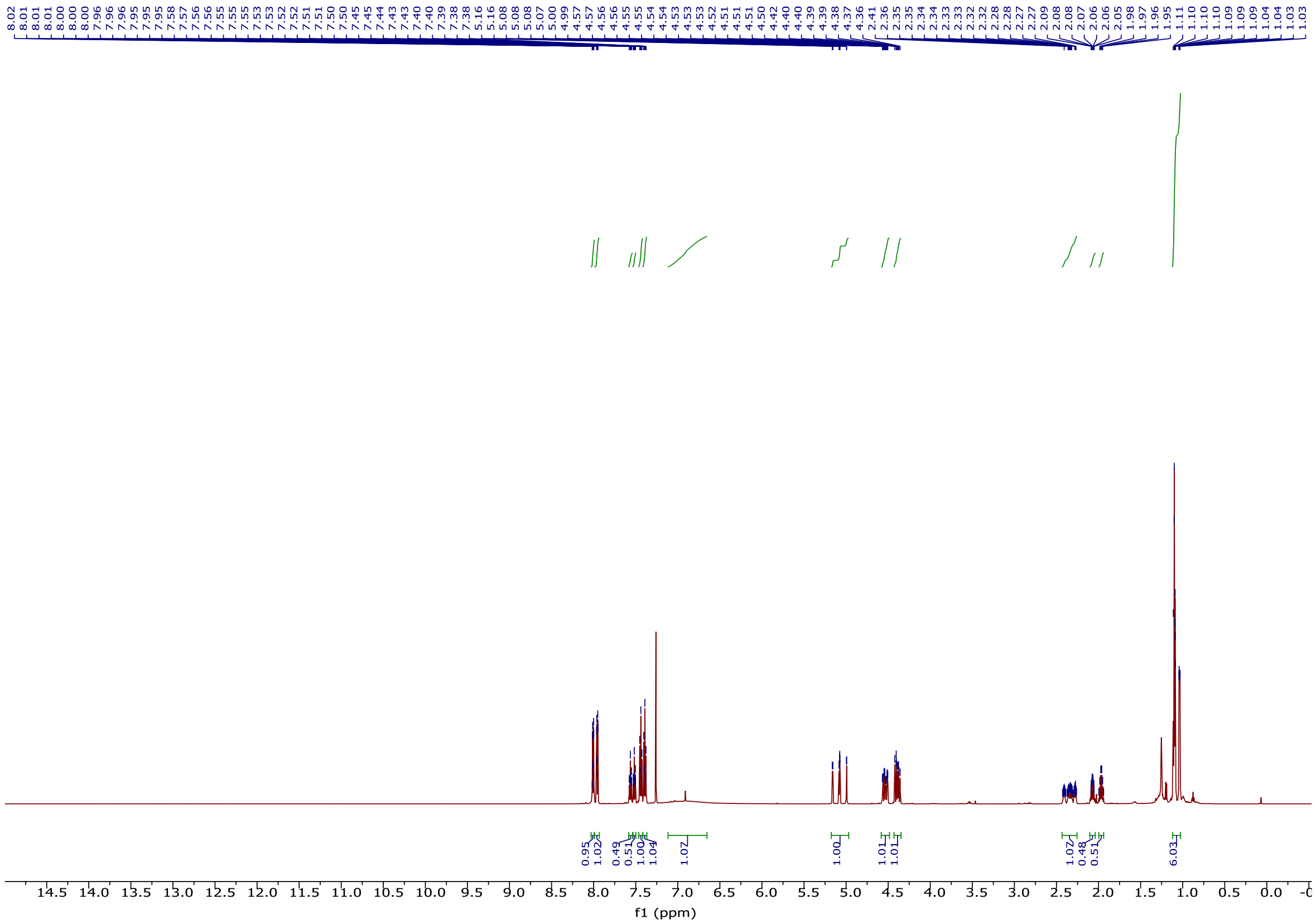

Supplementary Figure 168. ^{1}H NMR (600 MHz) of Compound (64)

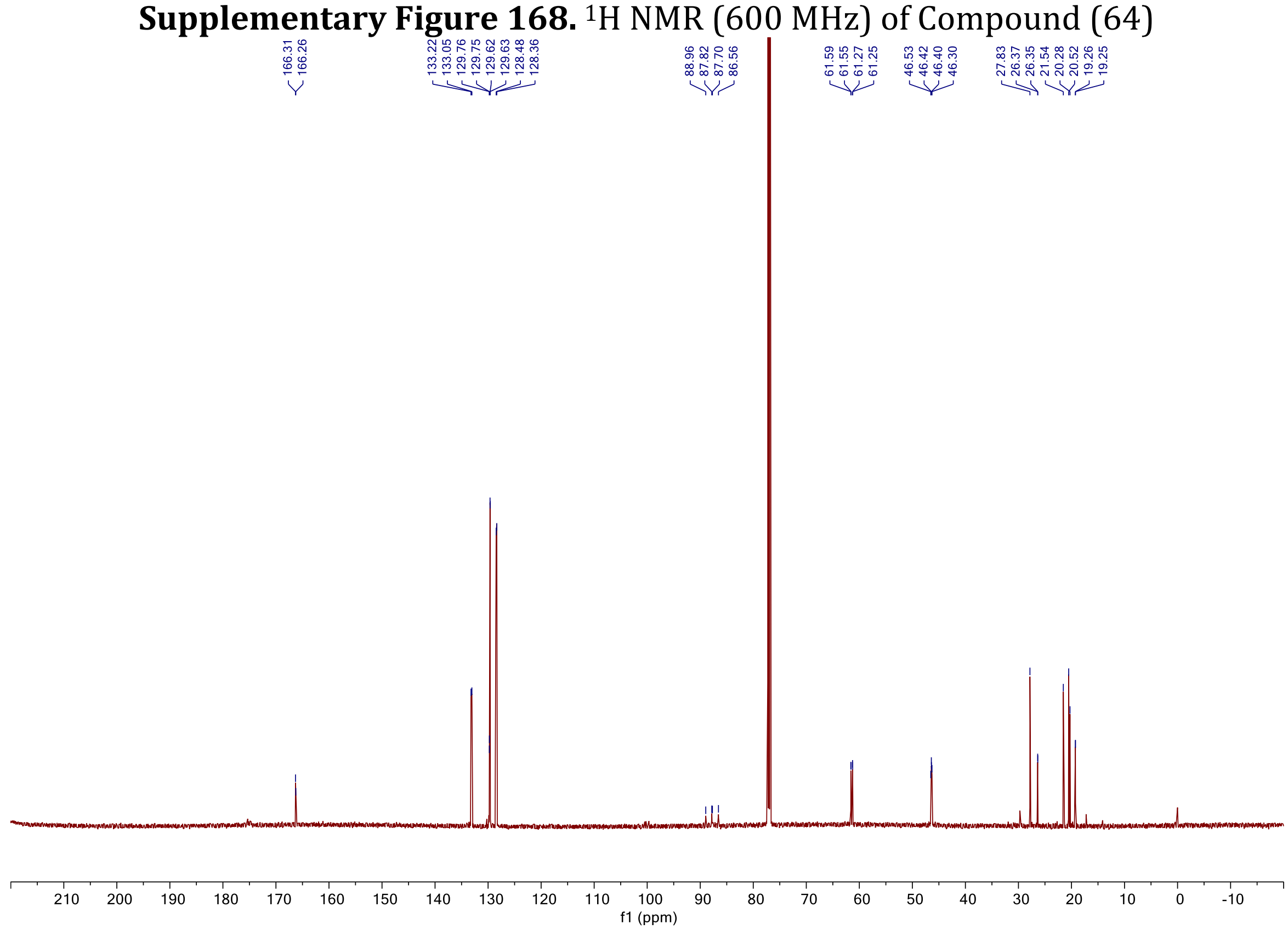

Supplementary Figure 169. ^{13}C NMR (151 MHz) of Compound (64)

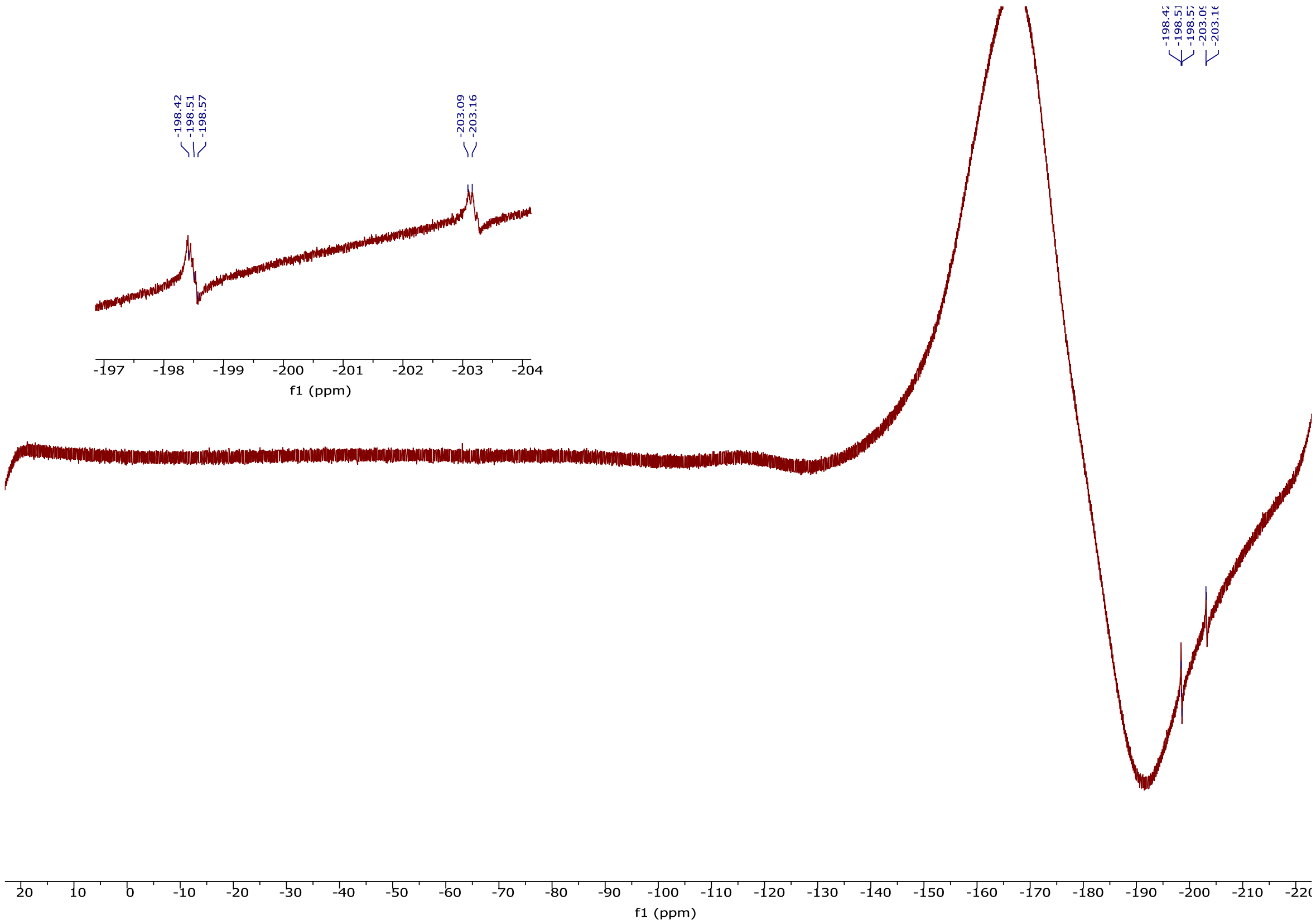

Supplementary Figure 170. ^{19}F NMR (564 MHz) of Compound (64)

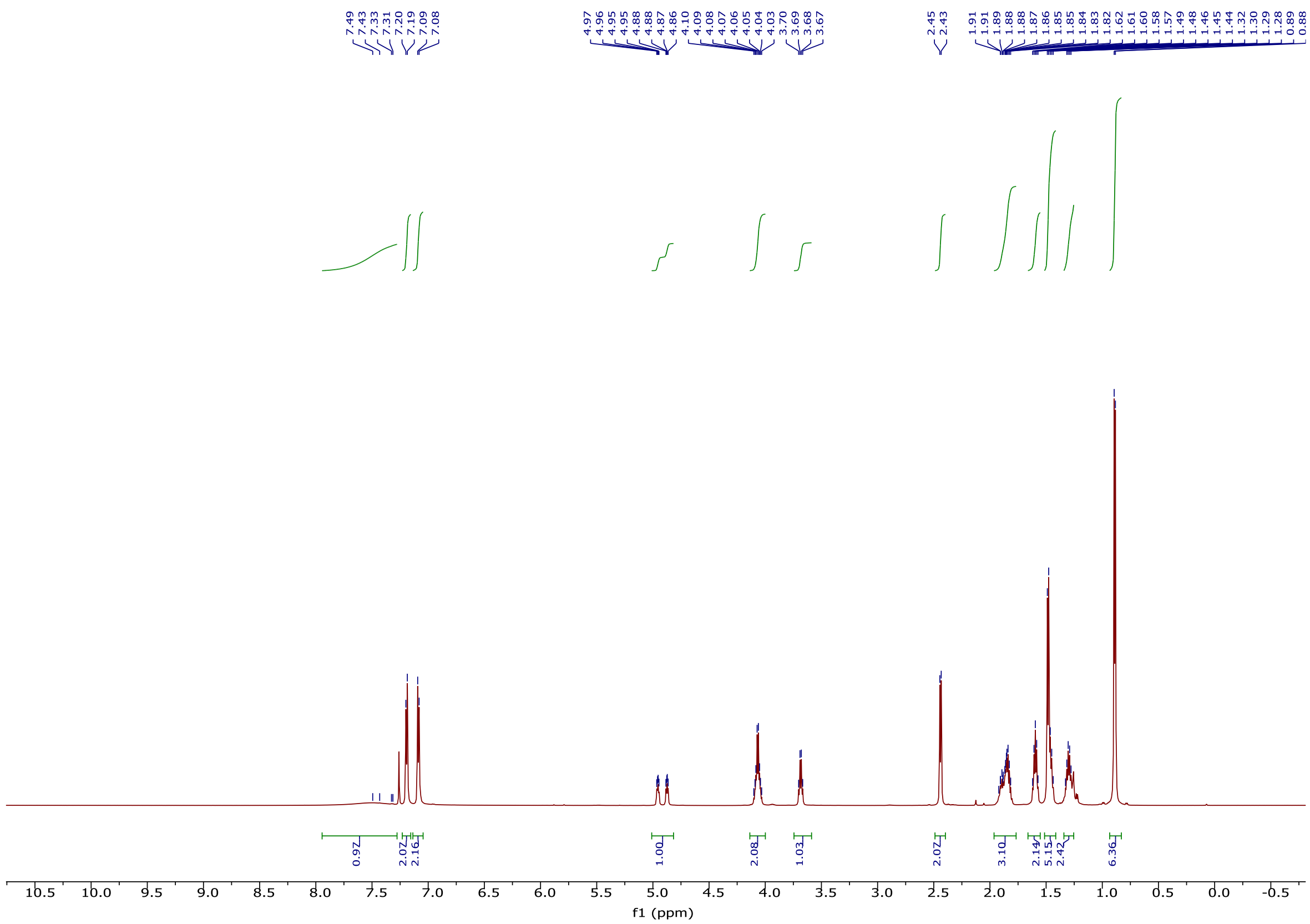

Supplementary Figure 171. ^{1}H NMR (600 MHz) of Compound (65)

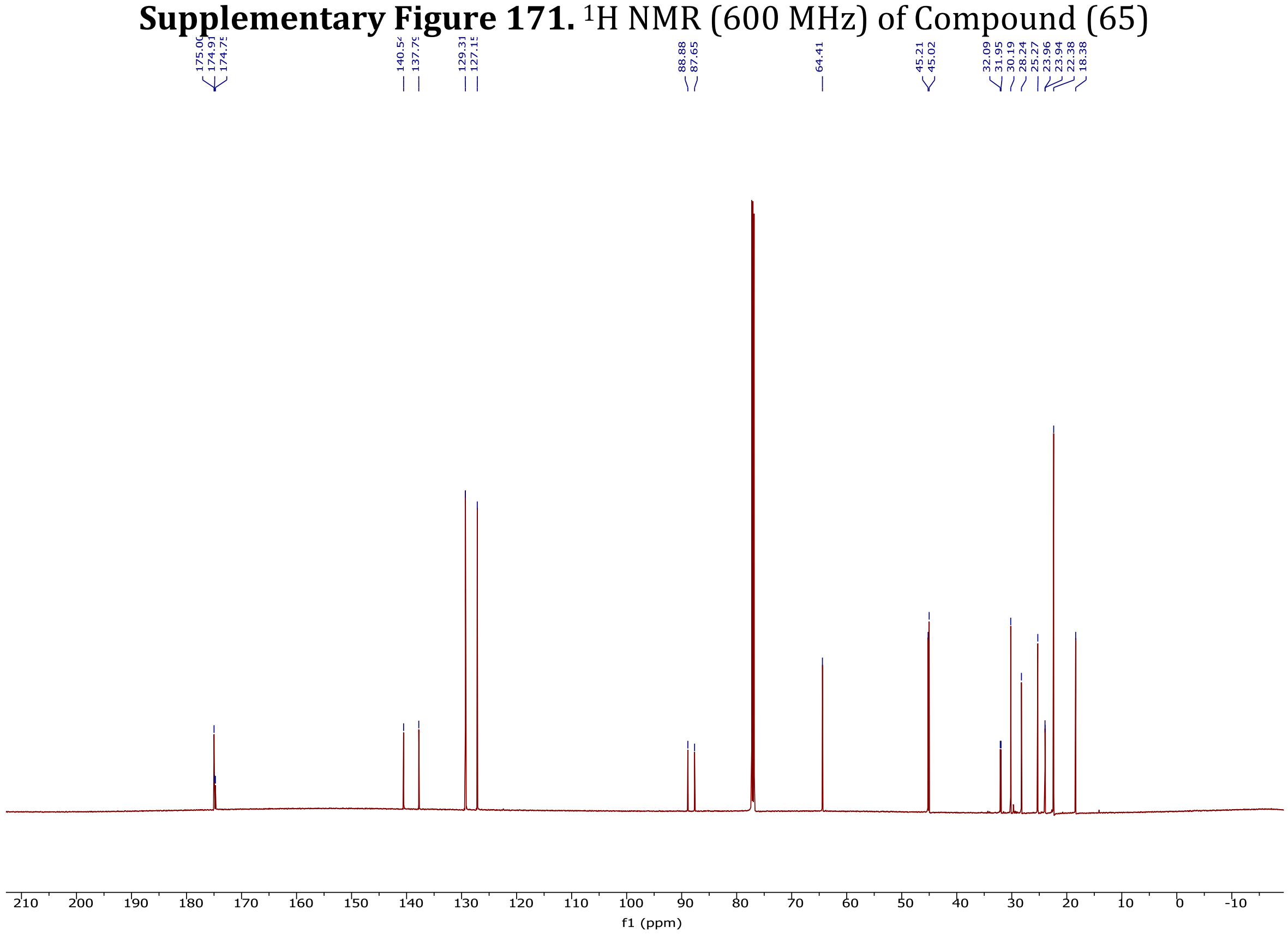

Supplementary Figure 172. ^{13}C NMR (151 MHz) of Compound (65)

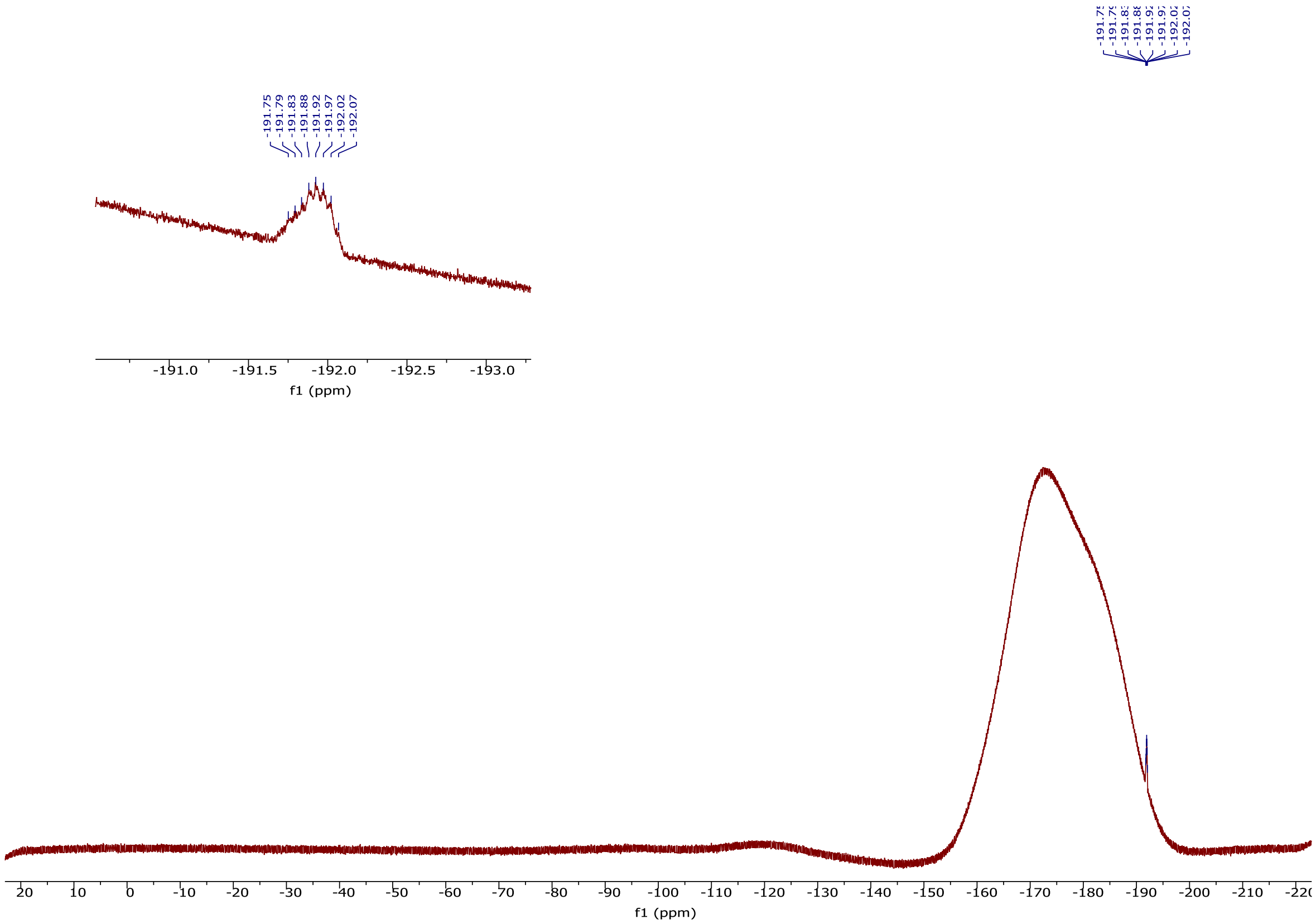

Supplementary Figure 173. ^{19}F NMR (564 MHz) of Compound (65)

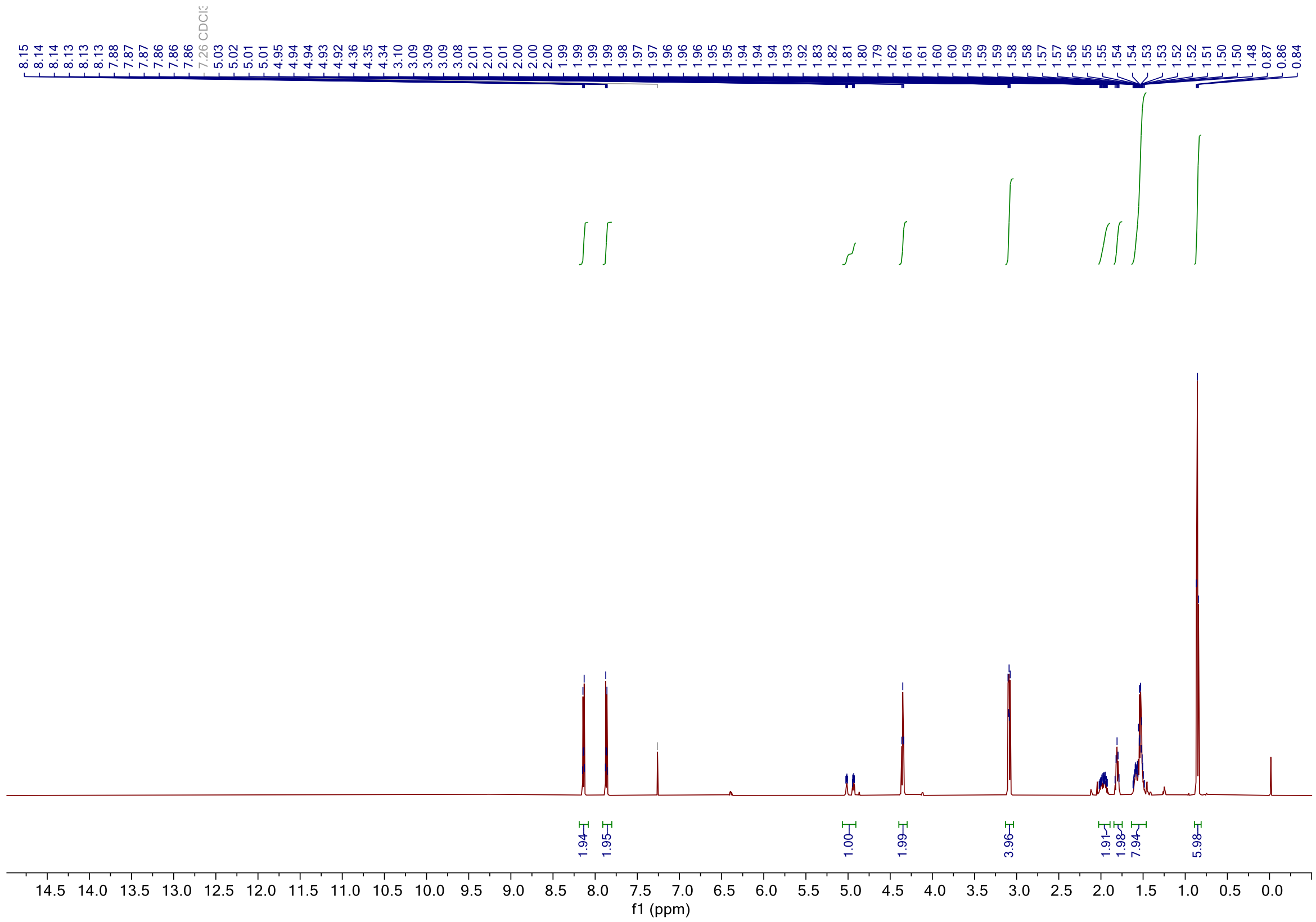

Supplementary Figure 174. ^{1}H NMR (600 MHz) of Compound (66)

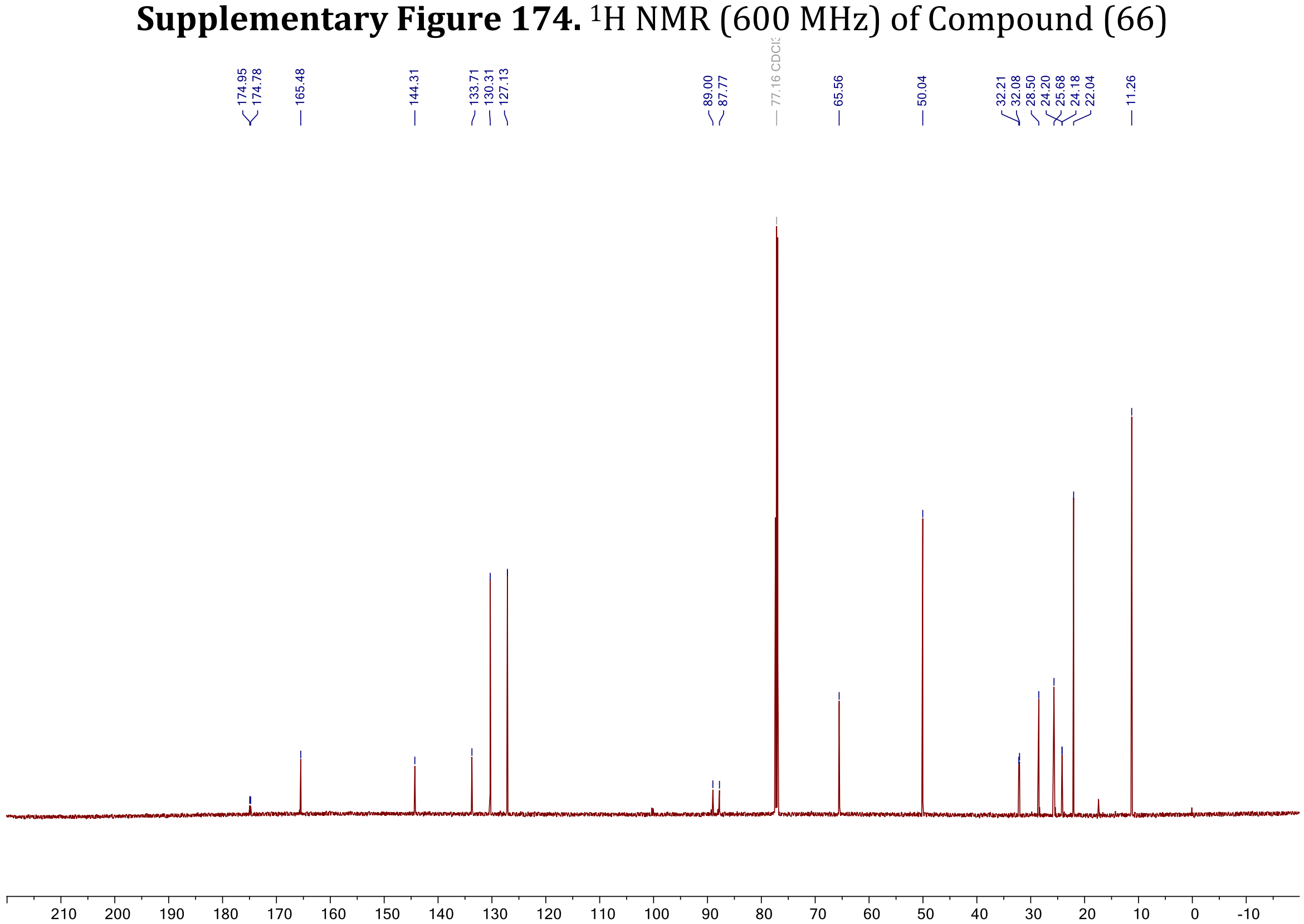

Supplementary Figure 175. ^{13}C NMR (151 MHz) of Compound (66)

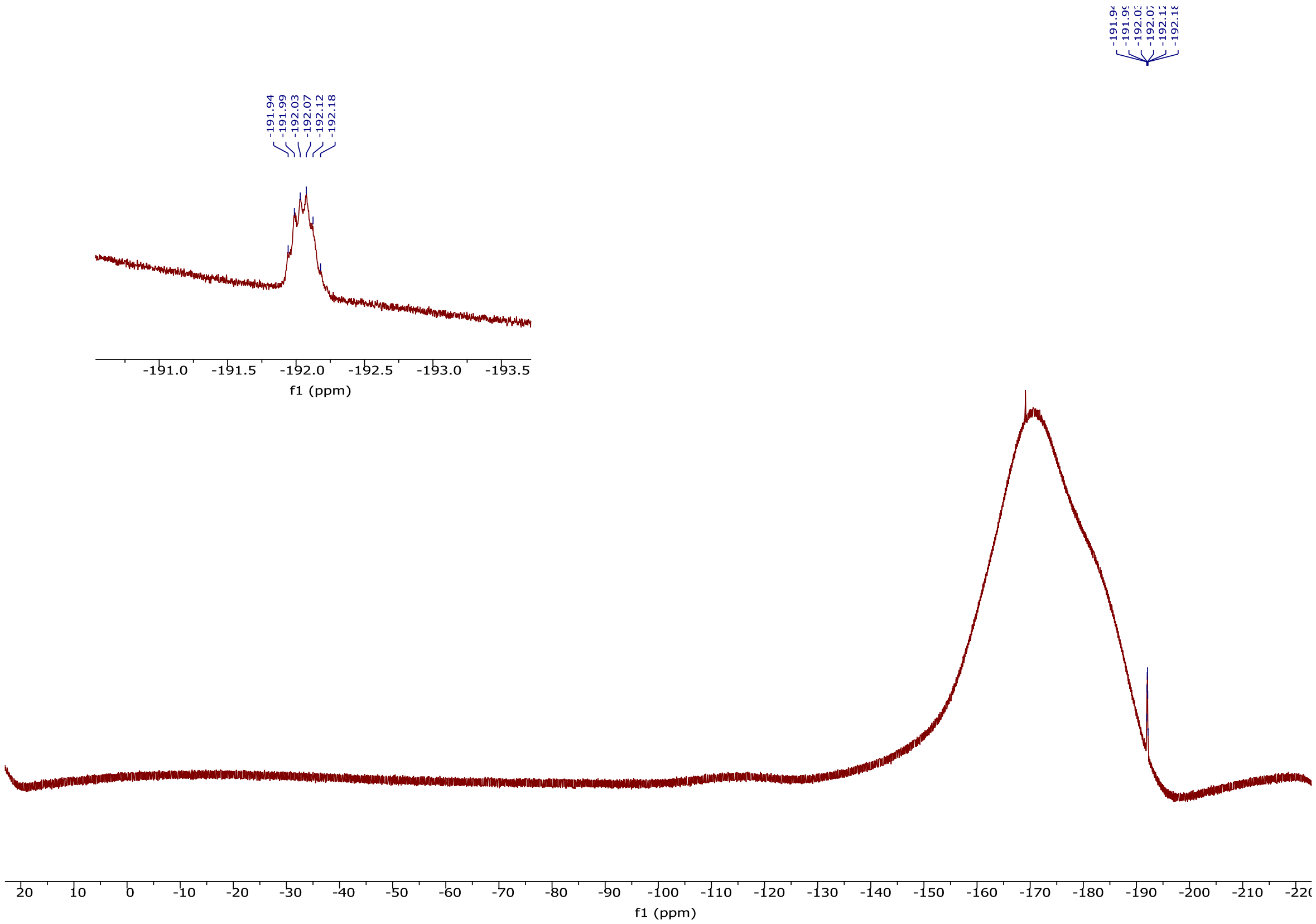

Supplementary Figure 176. ^{19}F NMR (564 MHz) of Compound (66)

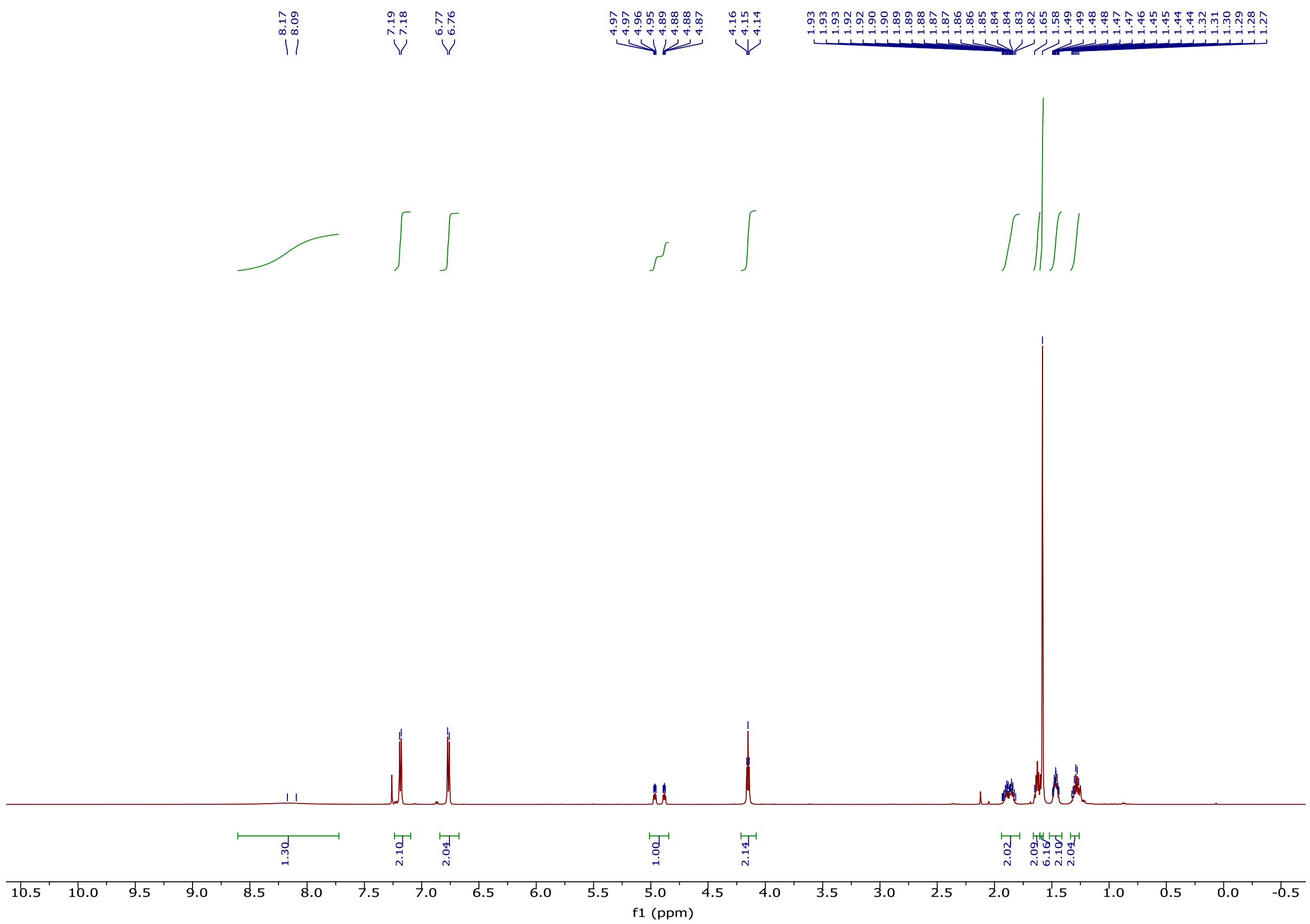

Supplementary Figure 177. ^{1}H NMR (600 MHz) of Compound (67)

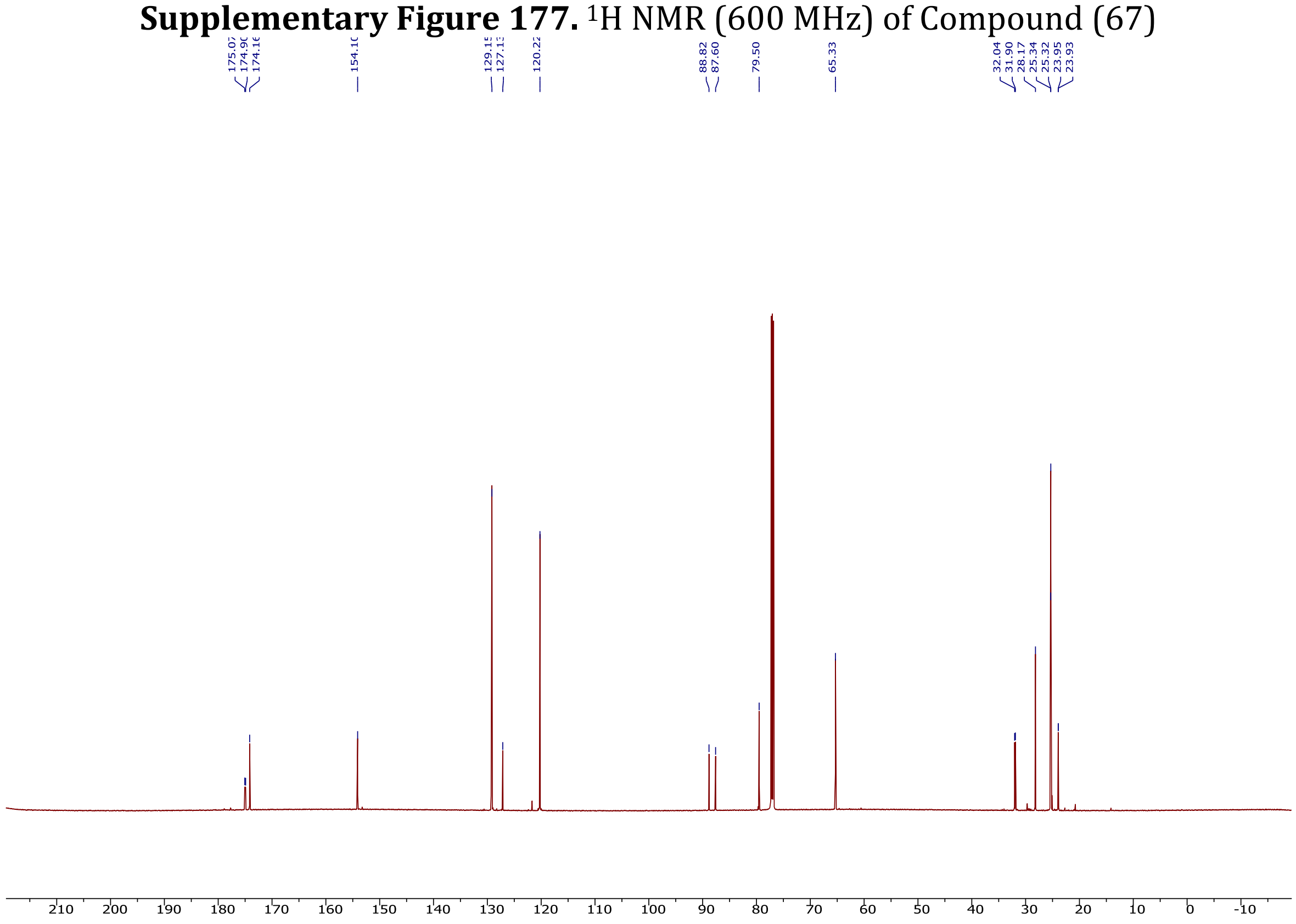

Supplementary Figure 178. ^{13}C NMR (151 MHz) of Compound (67)

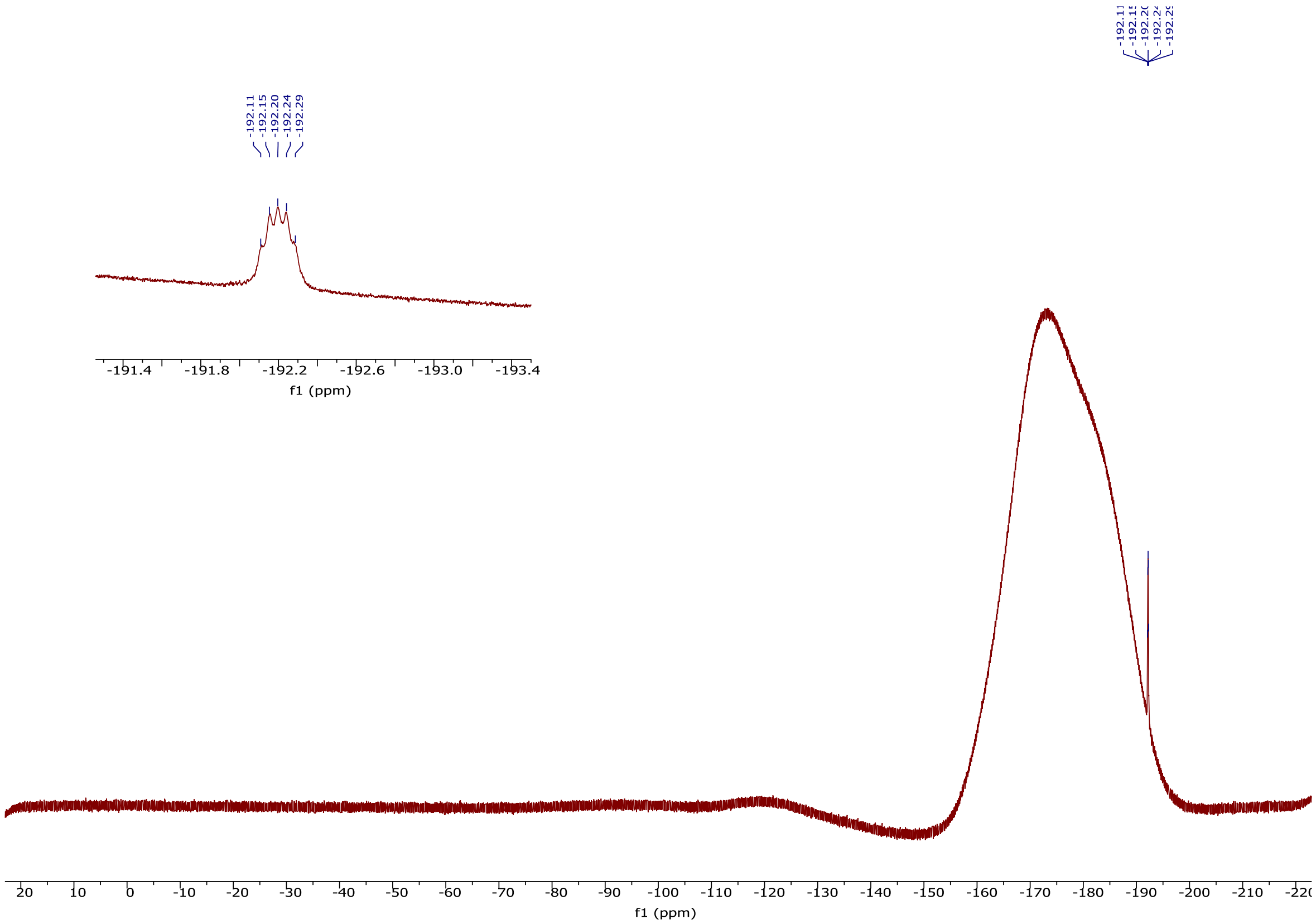

Supplementary Figure 179. ^{19}F NMR (564 MHz) of Compound (67)

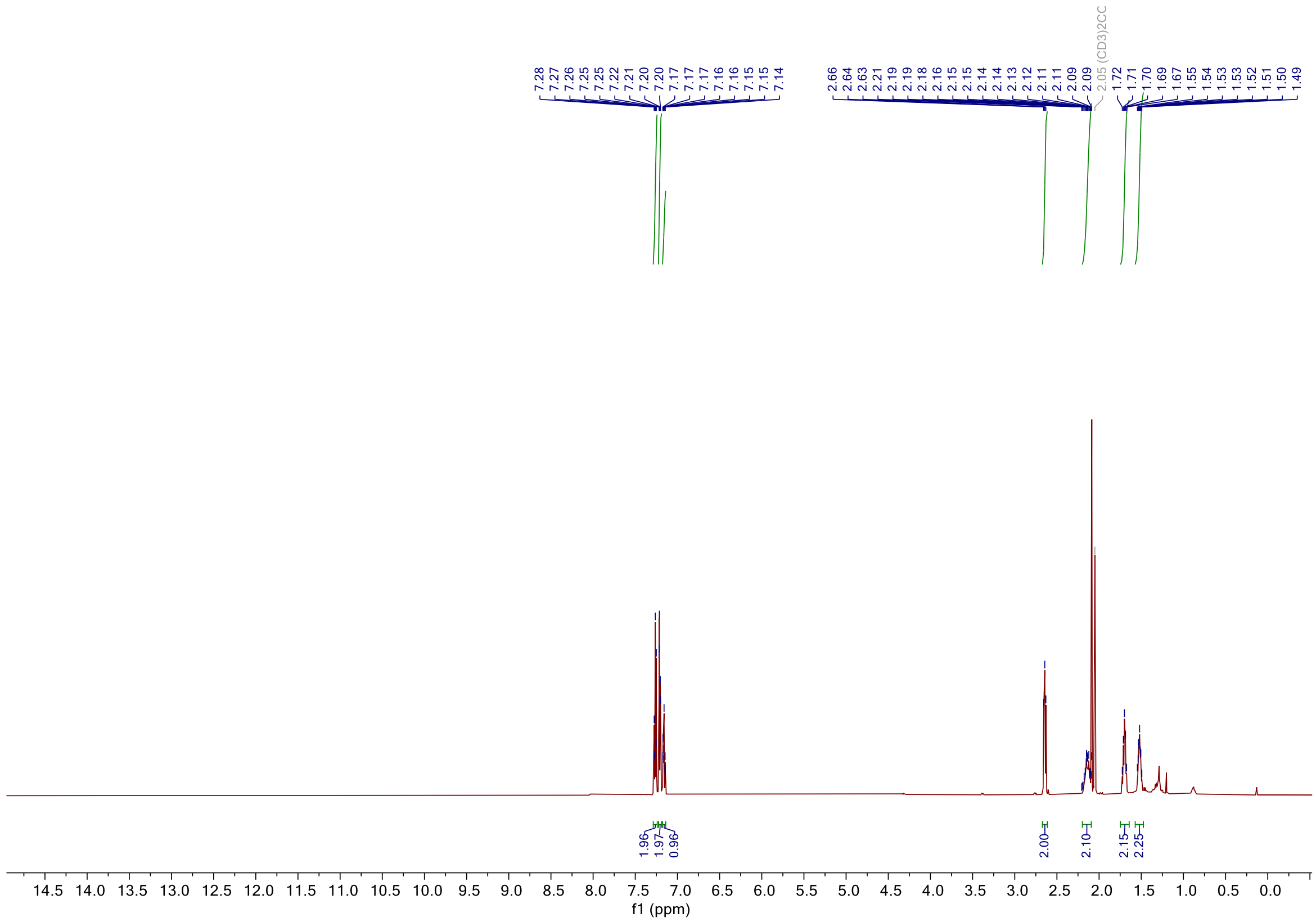

Supplementary Figure 180. ^{1}H NMR (600 MHz) of Compound (68)

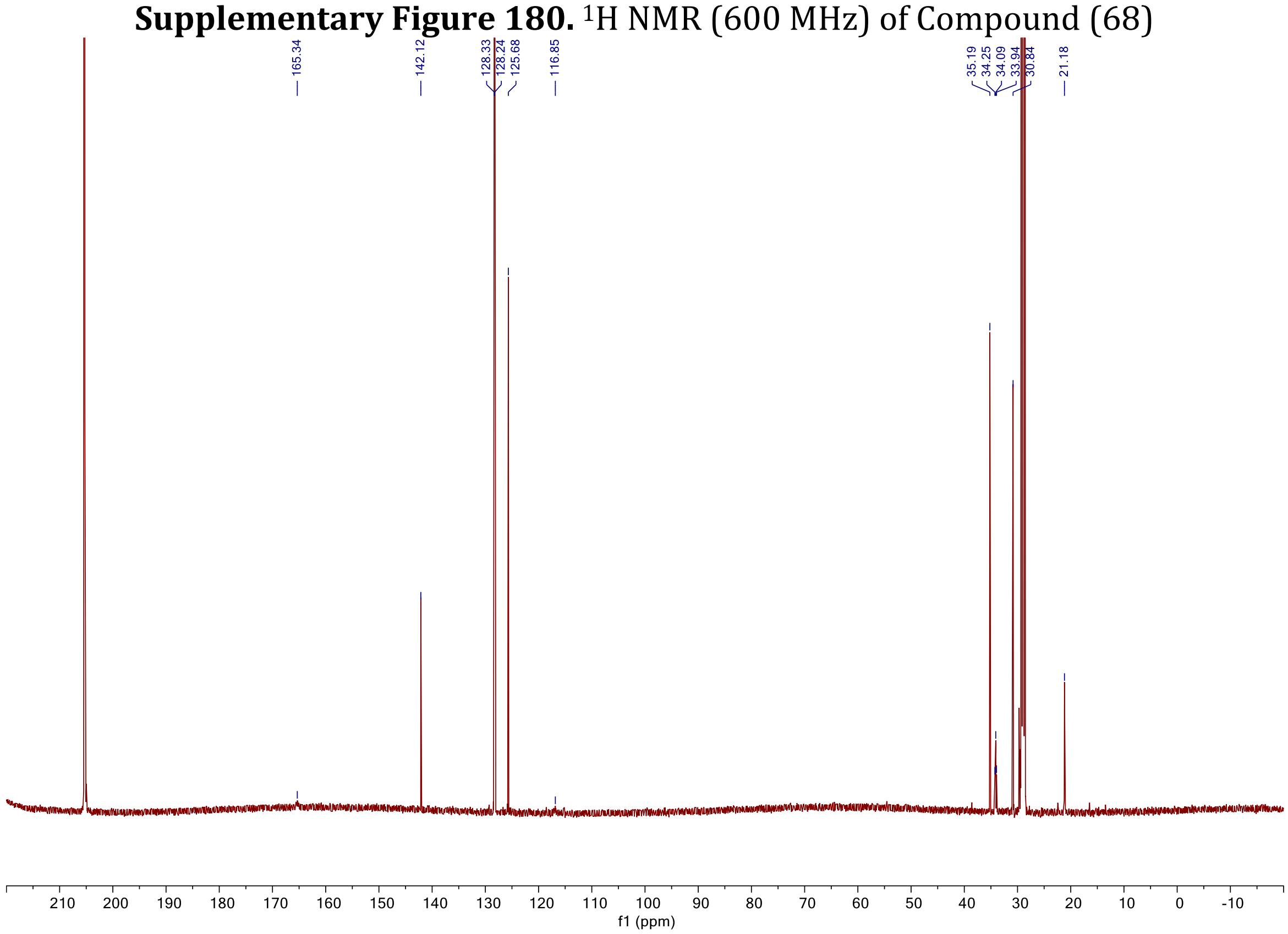

Supplementary Figure 181. ^{13}C NMR (151 MHz) of Compound (68)

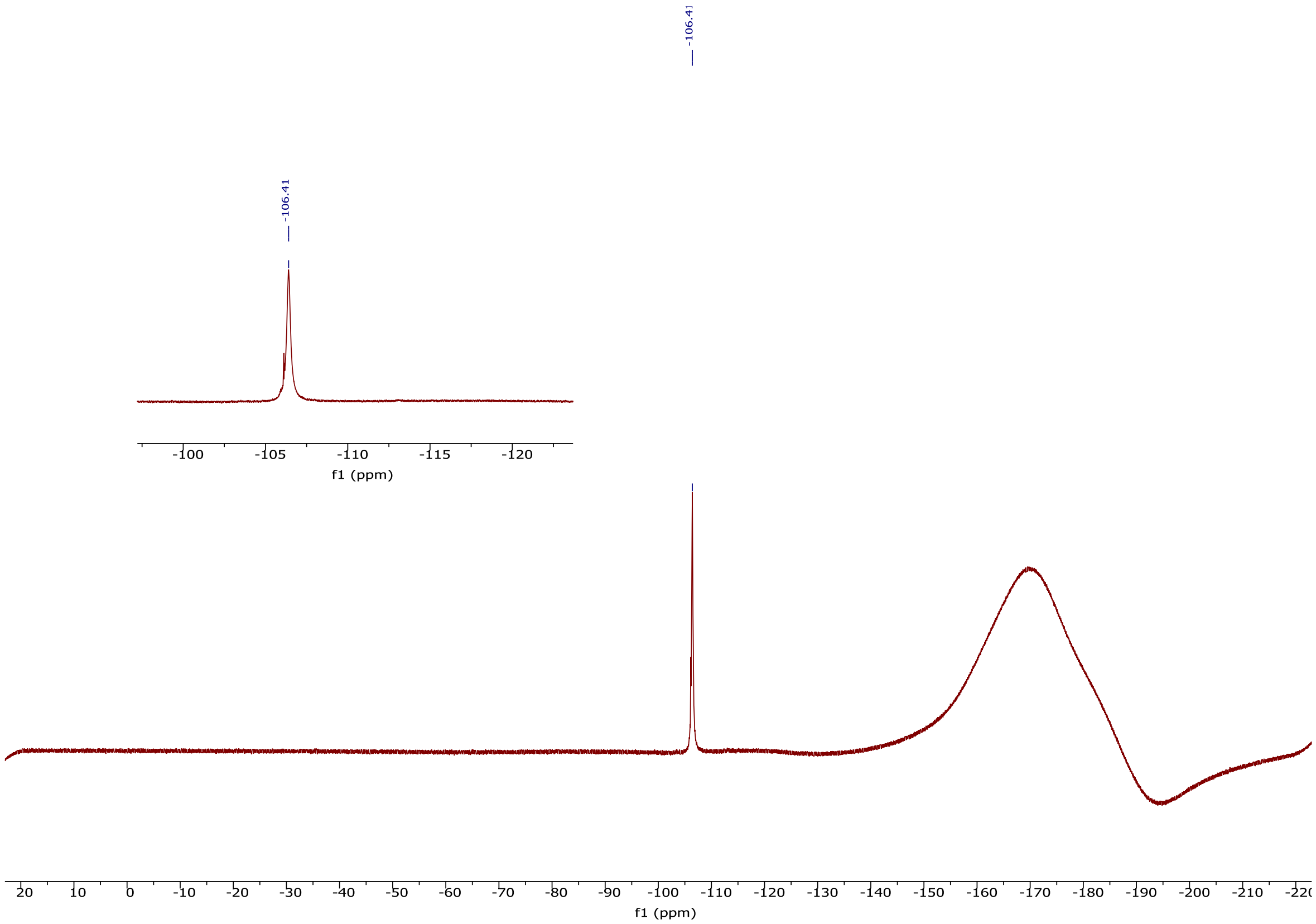

Supplementary Figure 182. ^{19}F NMR (564 MHz) of Compound (68)

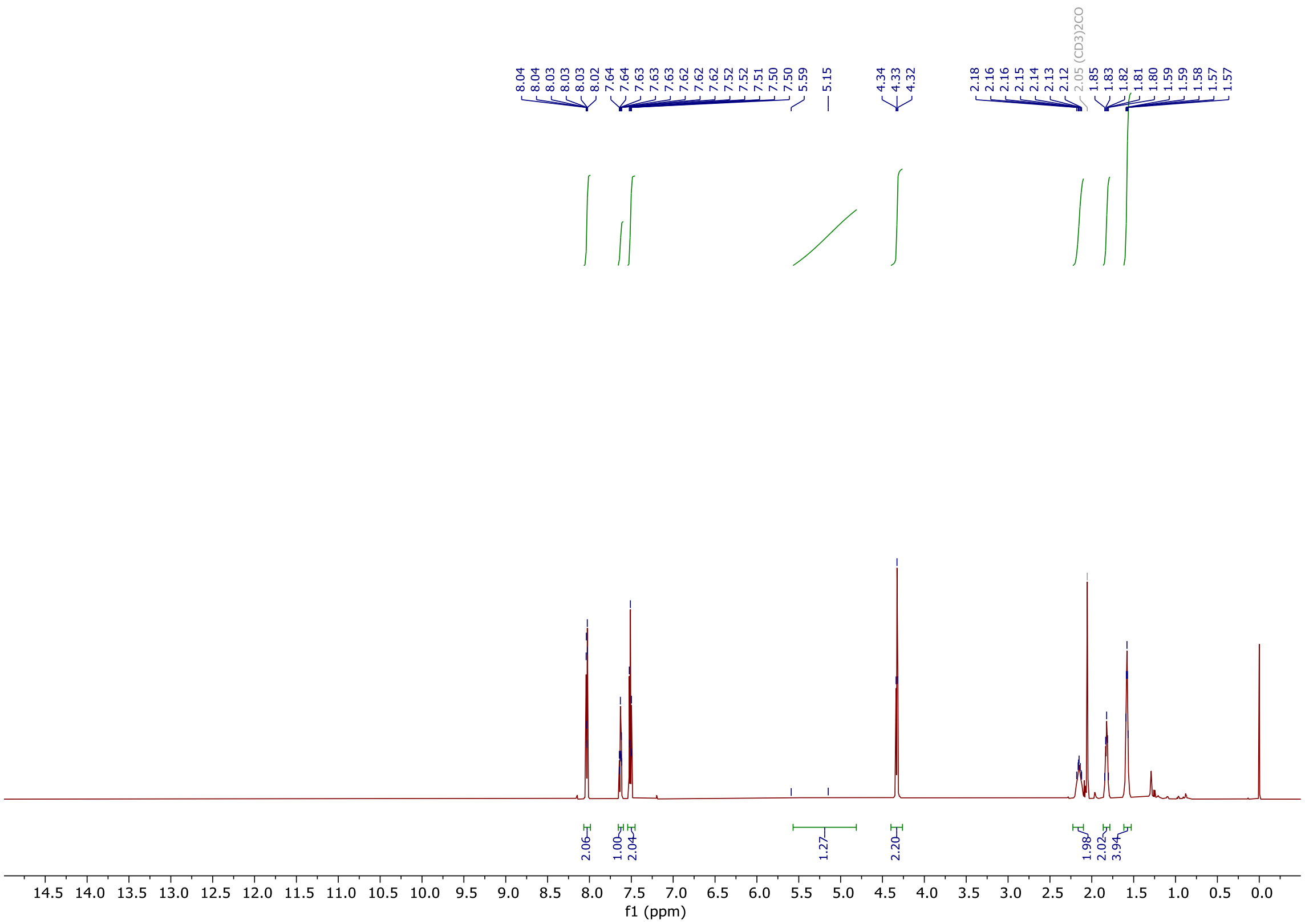

Supplementary Figure 183. ^{1}H NMR (600 MHz) of Compound (69)

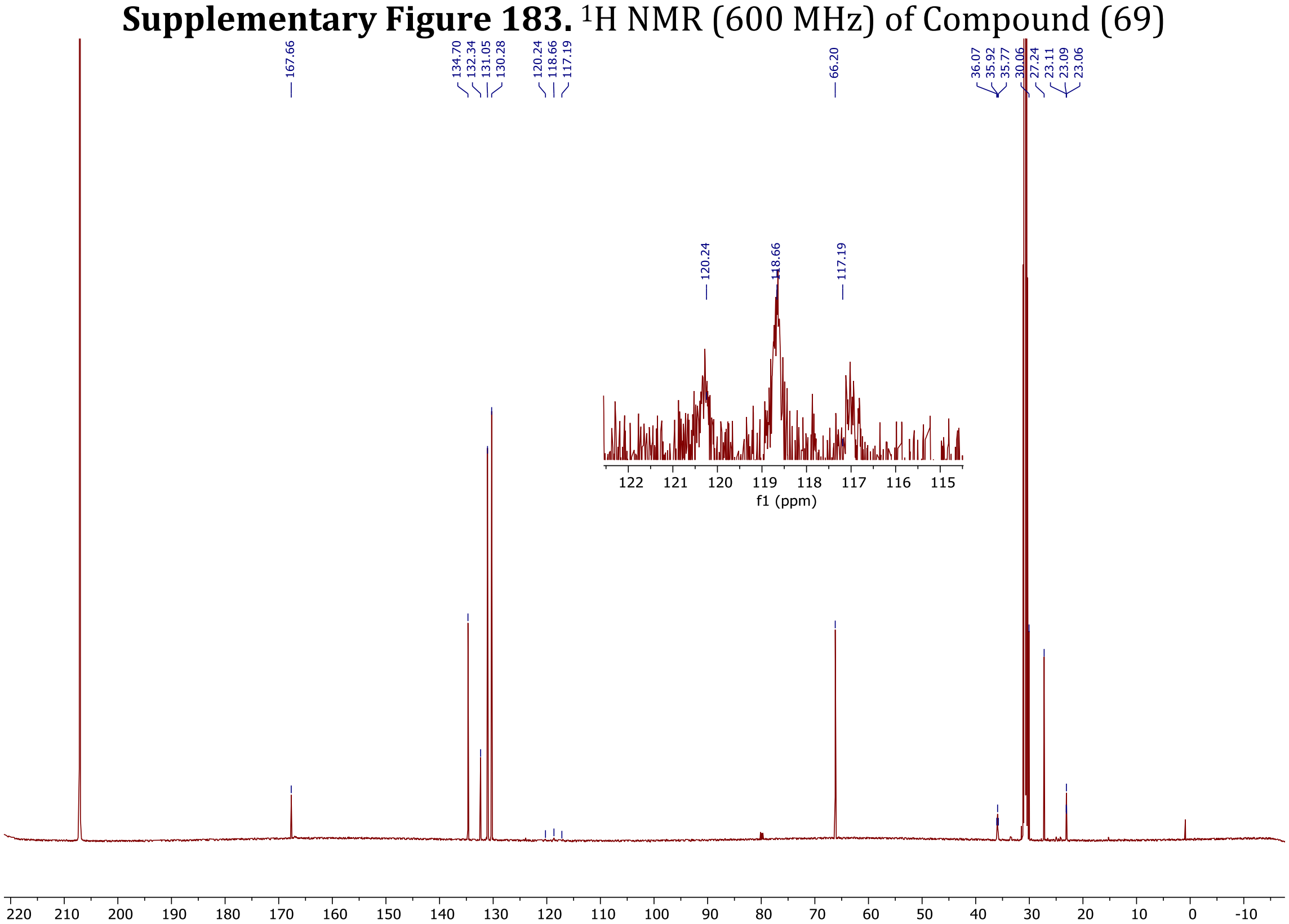

Supplementary Figure 184. ^{13}C NMR (151 MHz) of Compound (69)

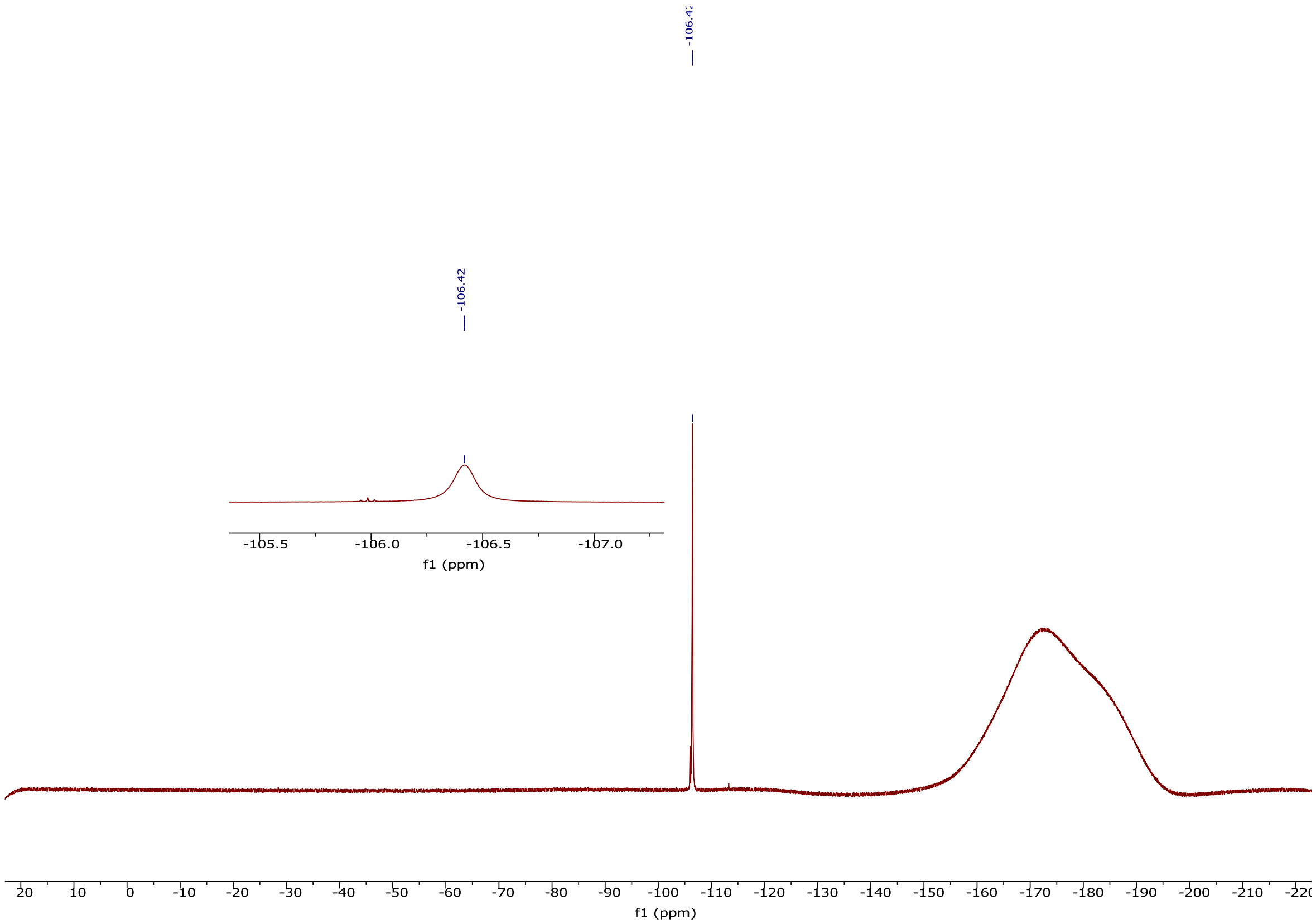

Supplementary Figure 185. ^{19}F NMR (564 MHz) of Compound (69)

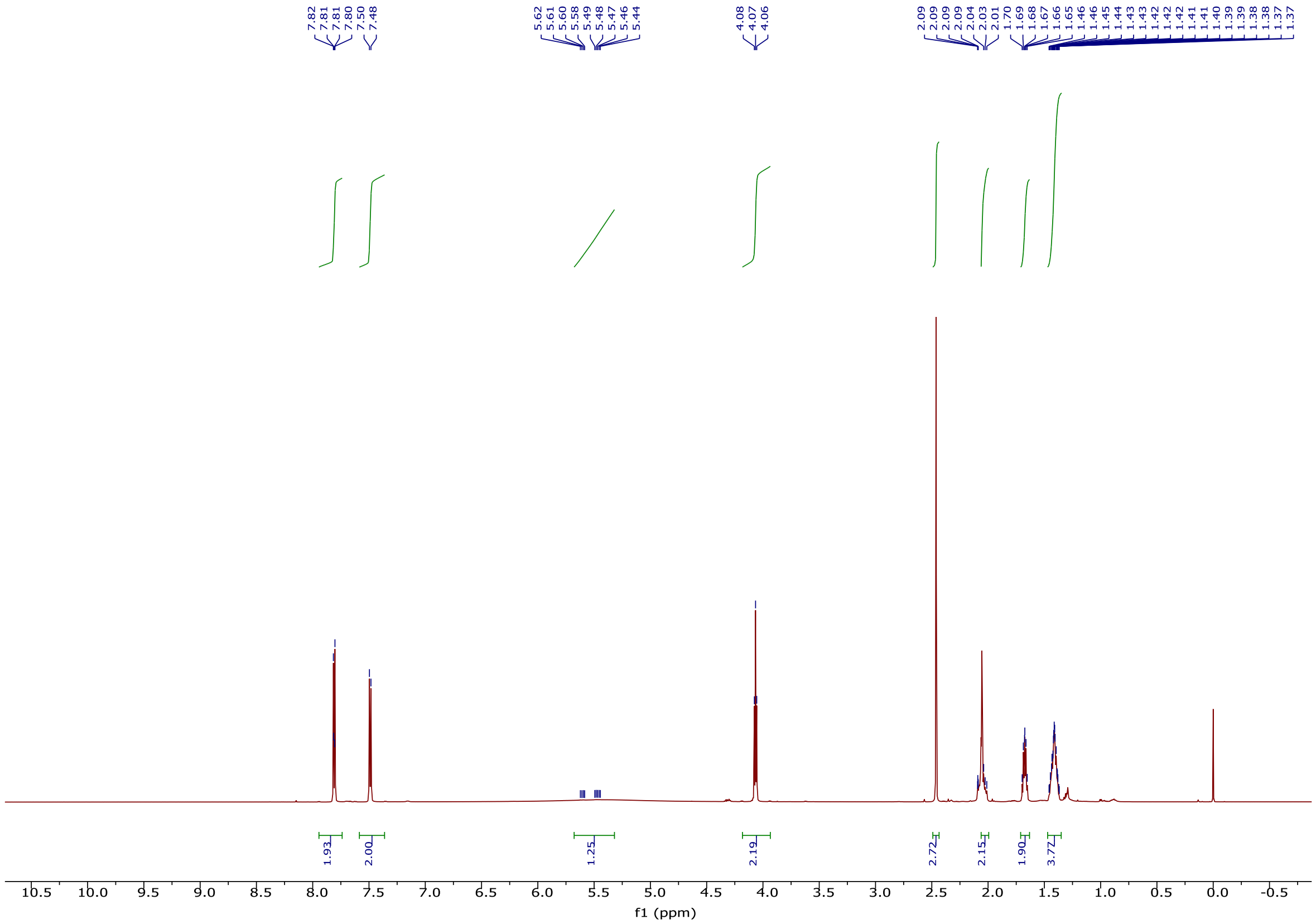

Supplementary Figure 186. ^{1}H NMR (600 MHz) of Compound (70)

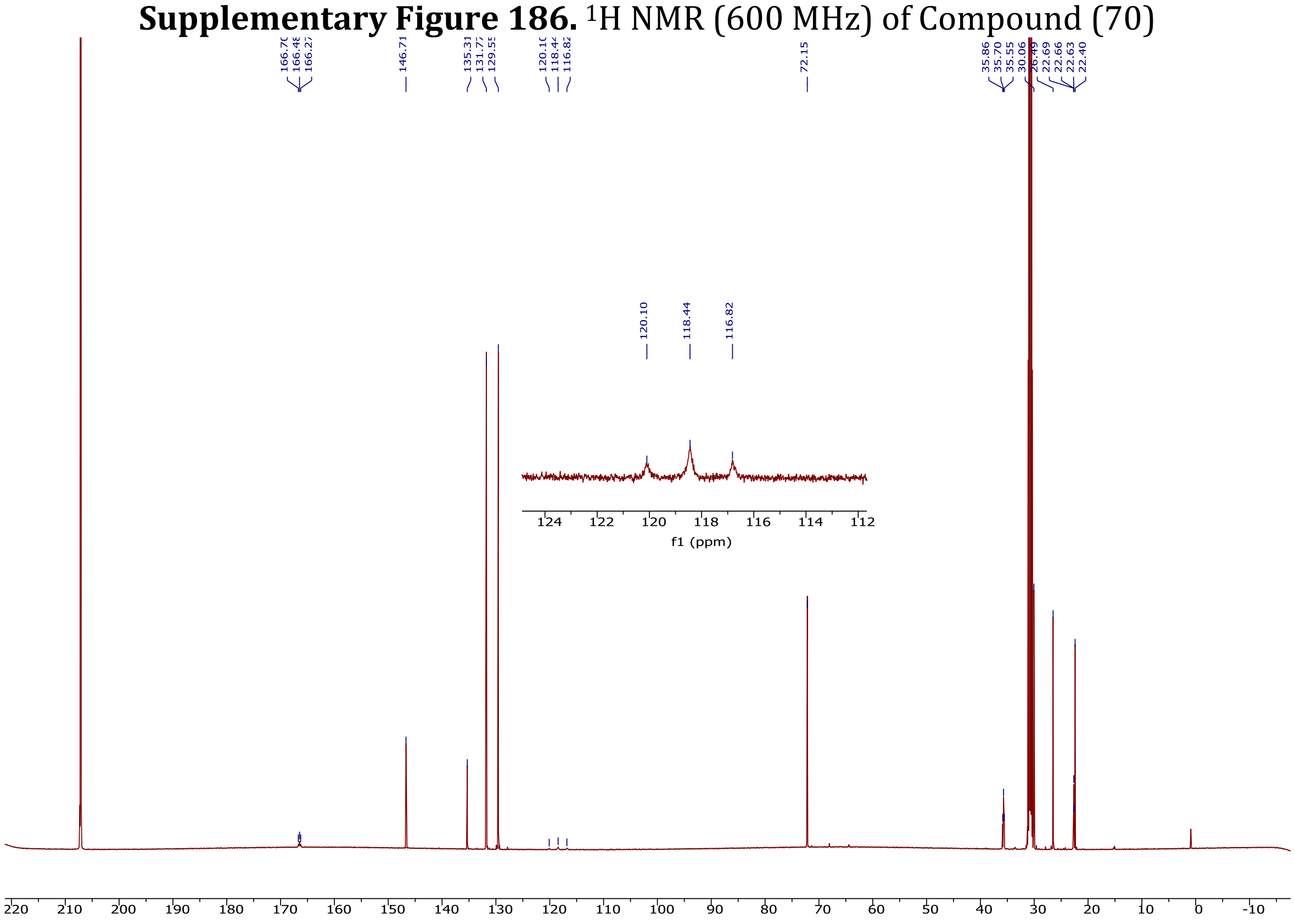

Supplementary Figure 187. ^{13}C NMR (151 MHz) of Compound (70)

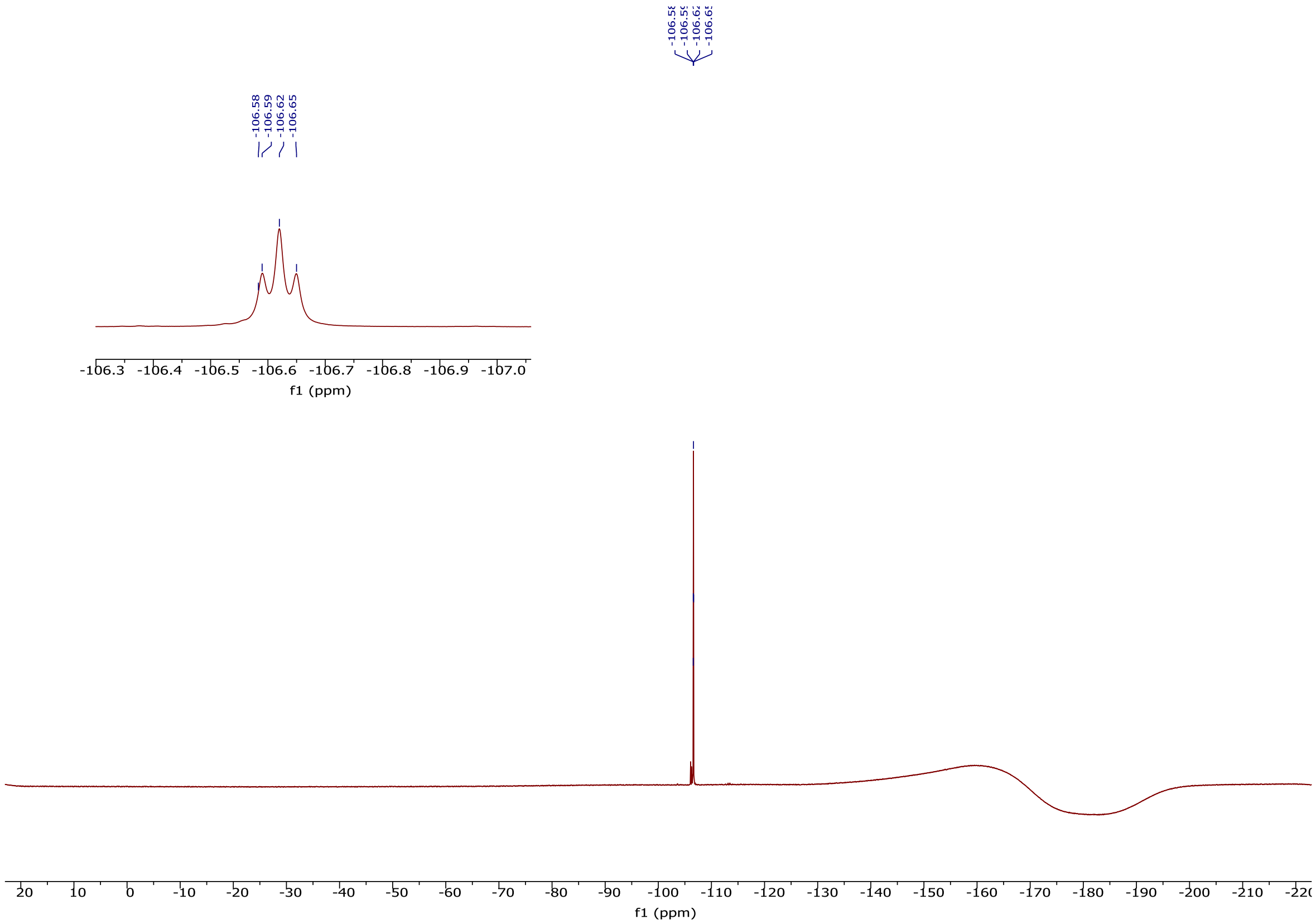

Supplementary Figure 188. ^{19}F NMR (564 MHz) of Compound (70)

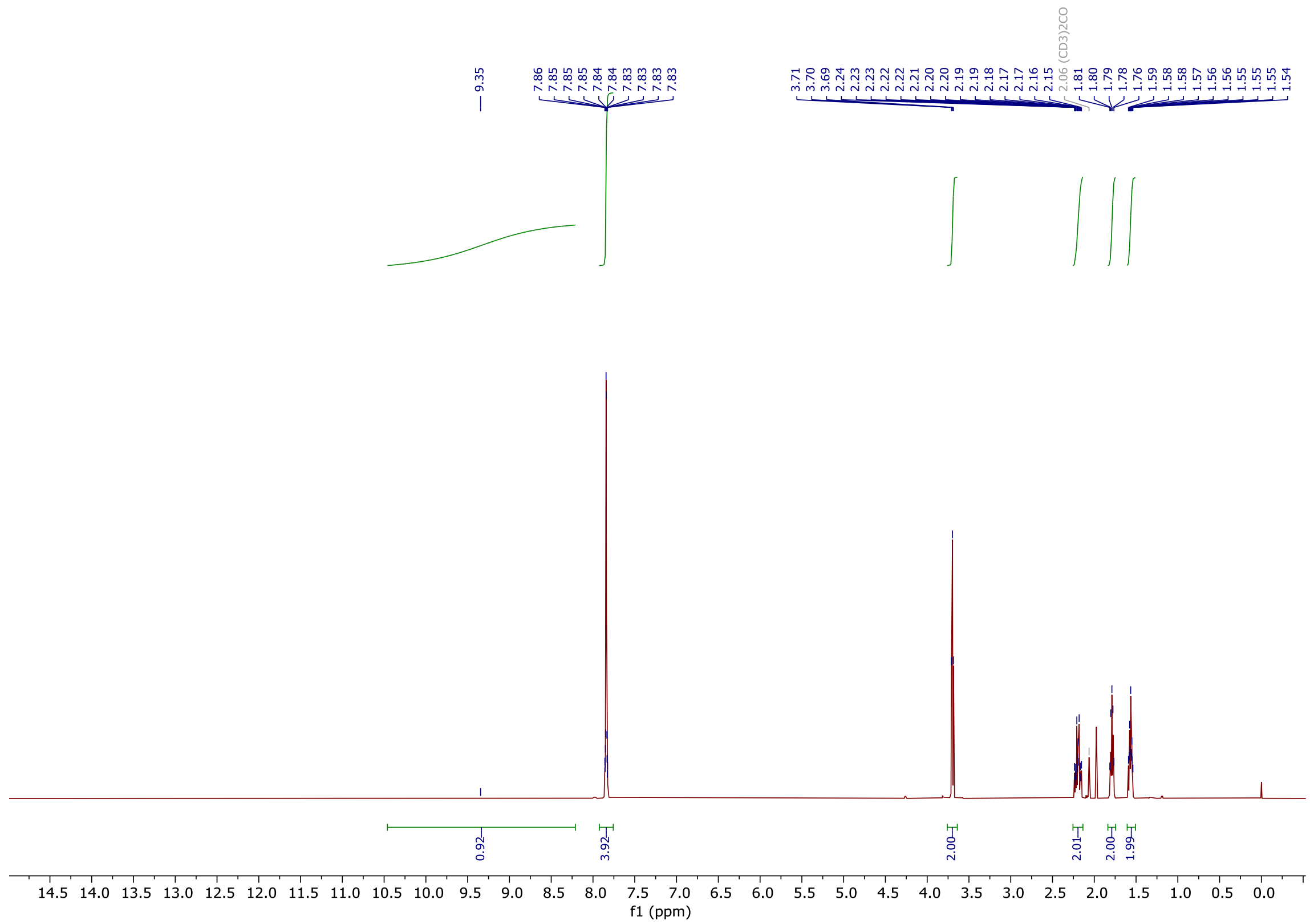

Supplementary Figure 189. ^{1}H NMR (600 MHz) of Compound (71)

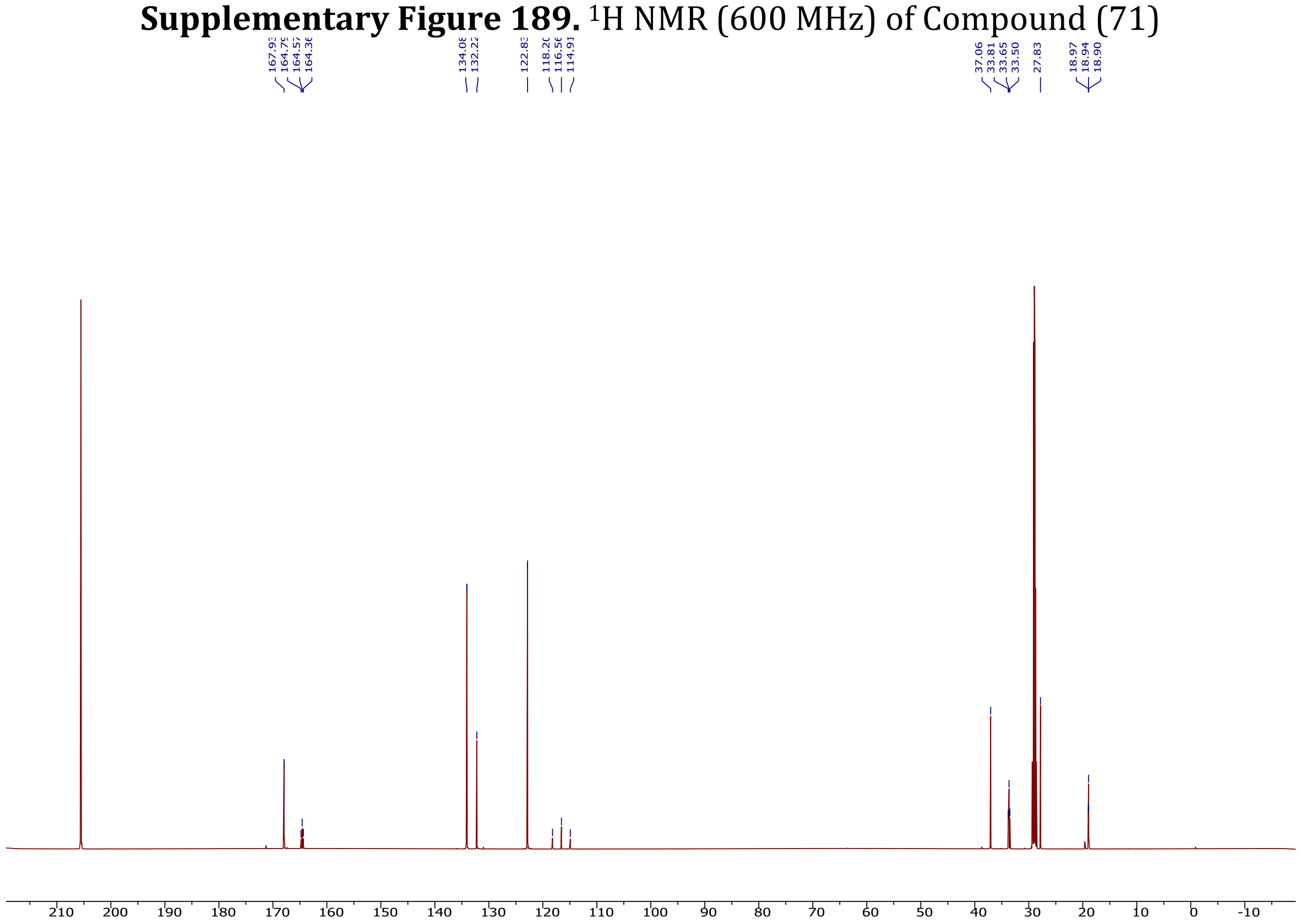

Supplementary Figure 190. ^{13}C NMR (151 MHz) of Compound (71)

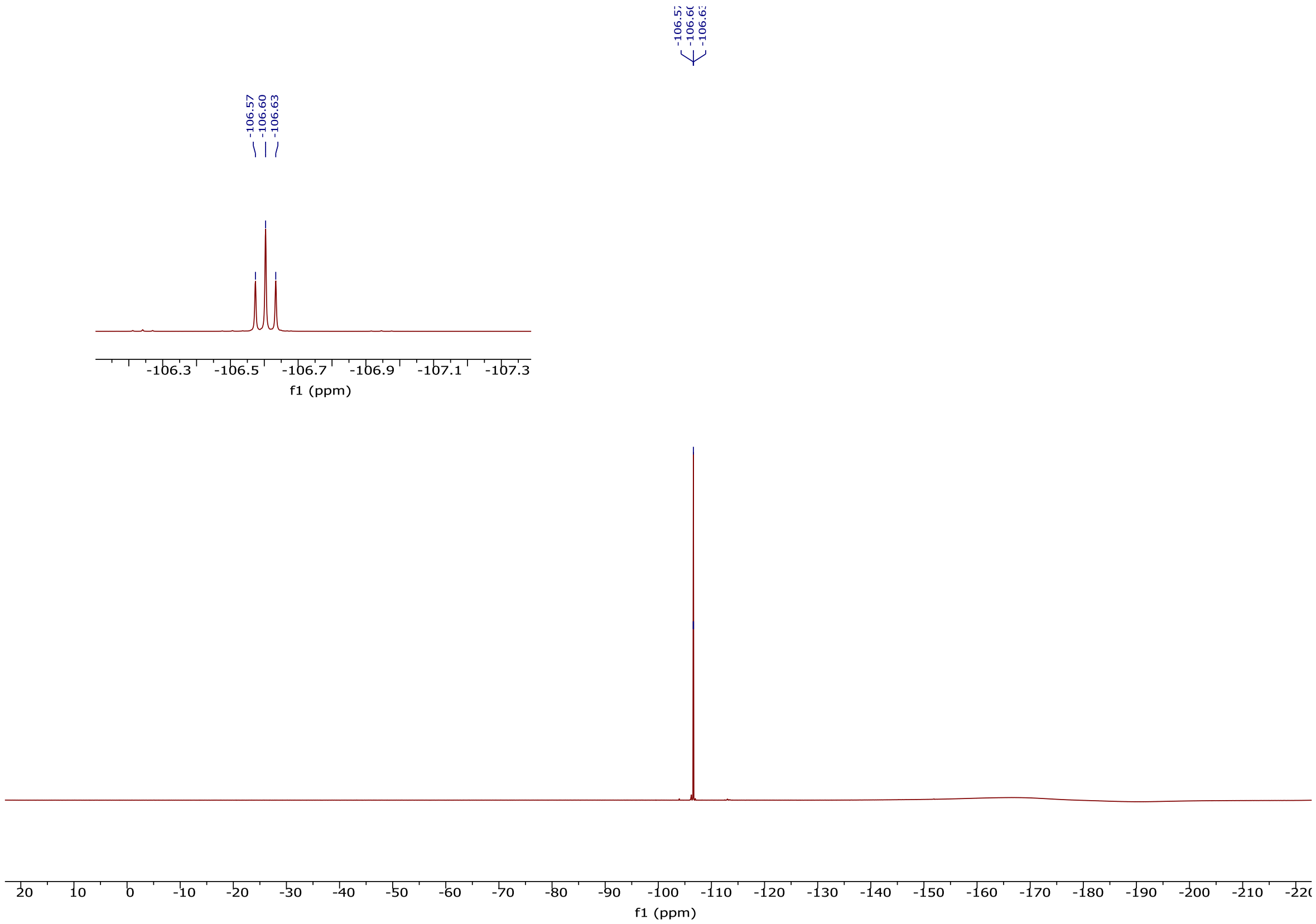

Supplementary Figure 191. ^{19}F NMR (564 MHz) of Compound (71)

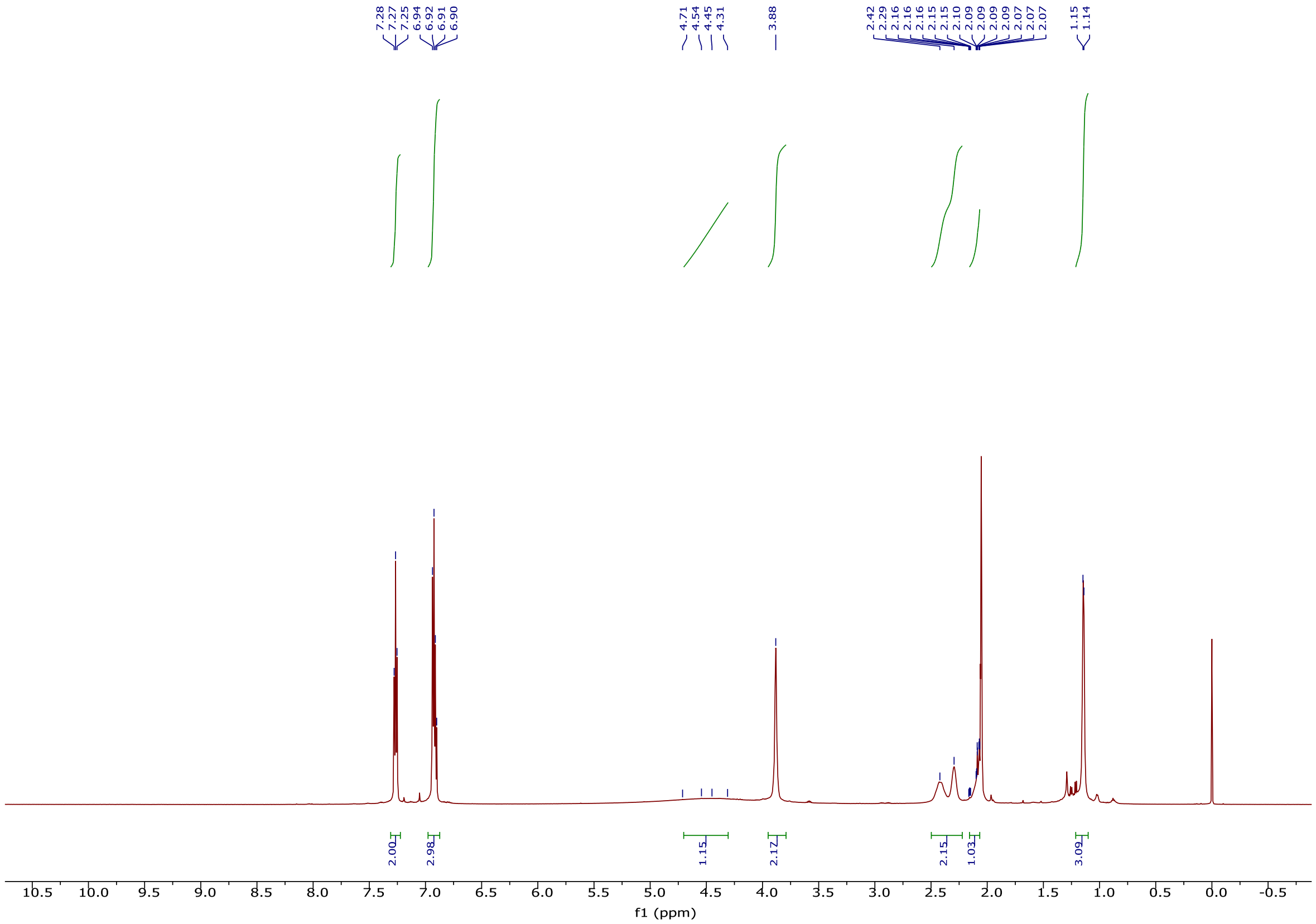

Supplementary Figure 192. ^{1}H NMR (600 MHz) of Compound (72)

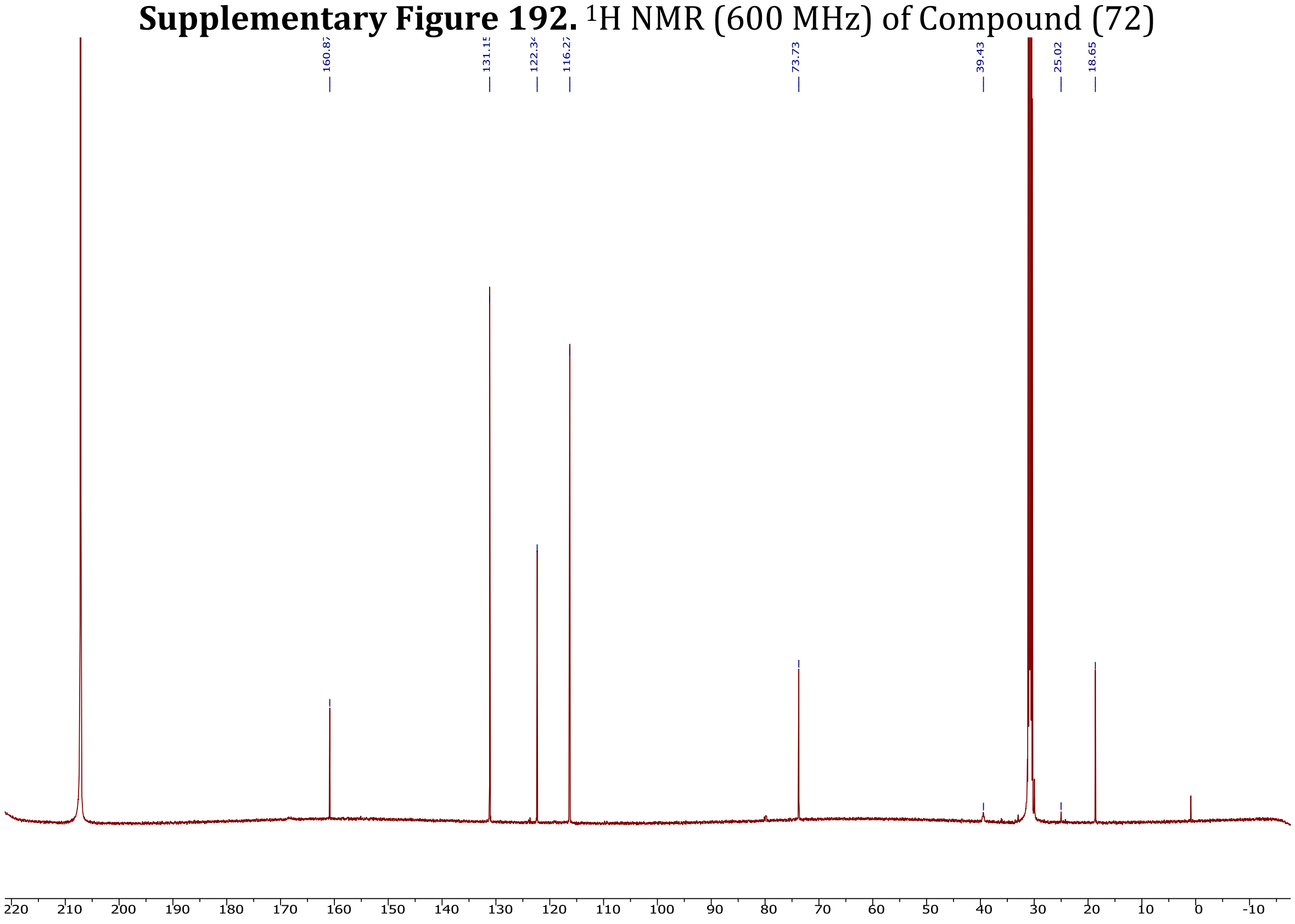

Supplementary Figure 193. ^{13}C NMR (151 MHz) of Compound (72)

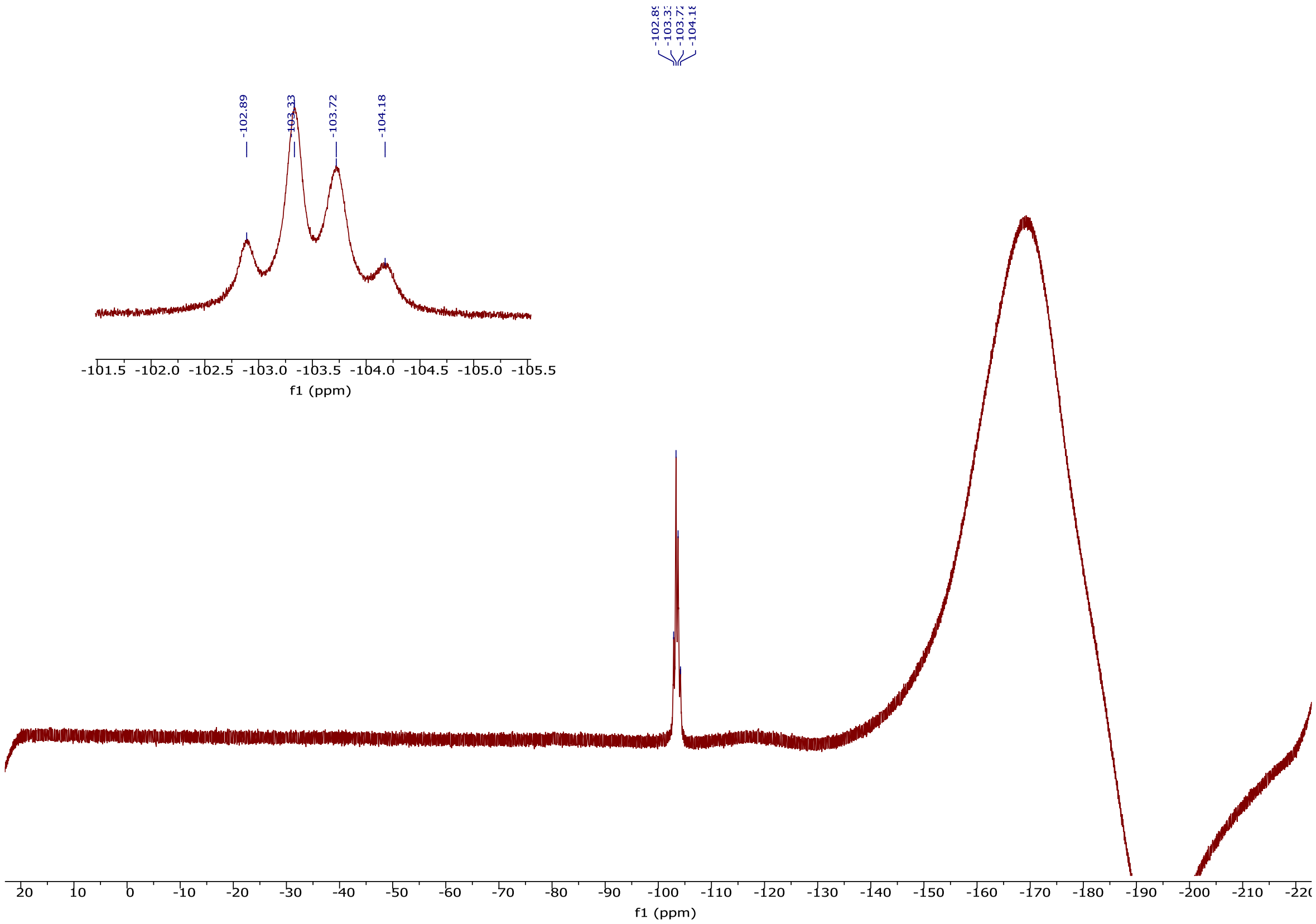

Supplementary Figure 194. ^{19}F NMR (564 MHz) of Compound (72)

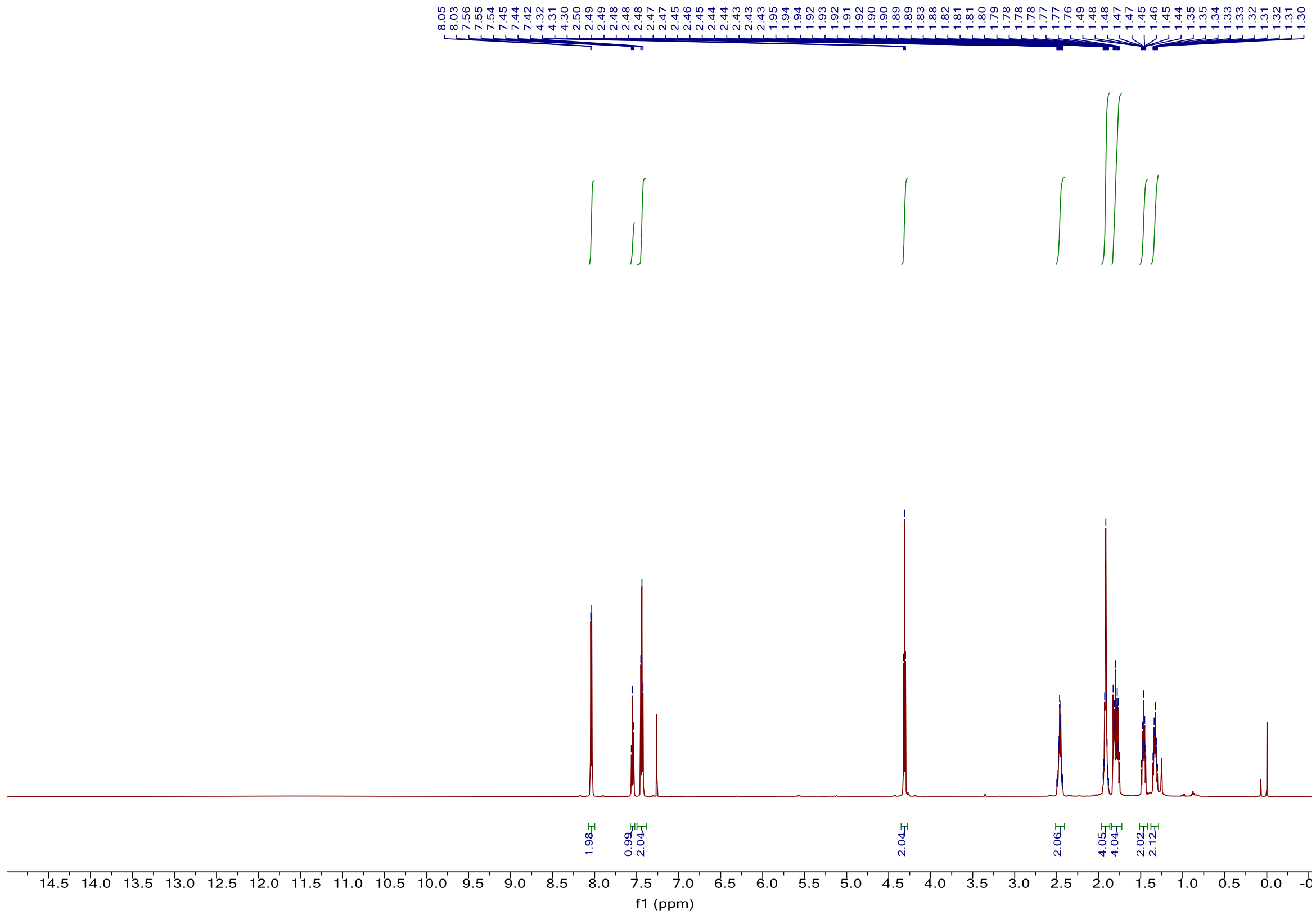

Supplementary Figure 195. ^{1}H NMR (600 MHz) of Compound (73)

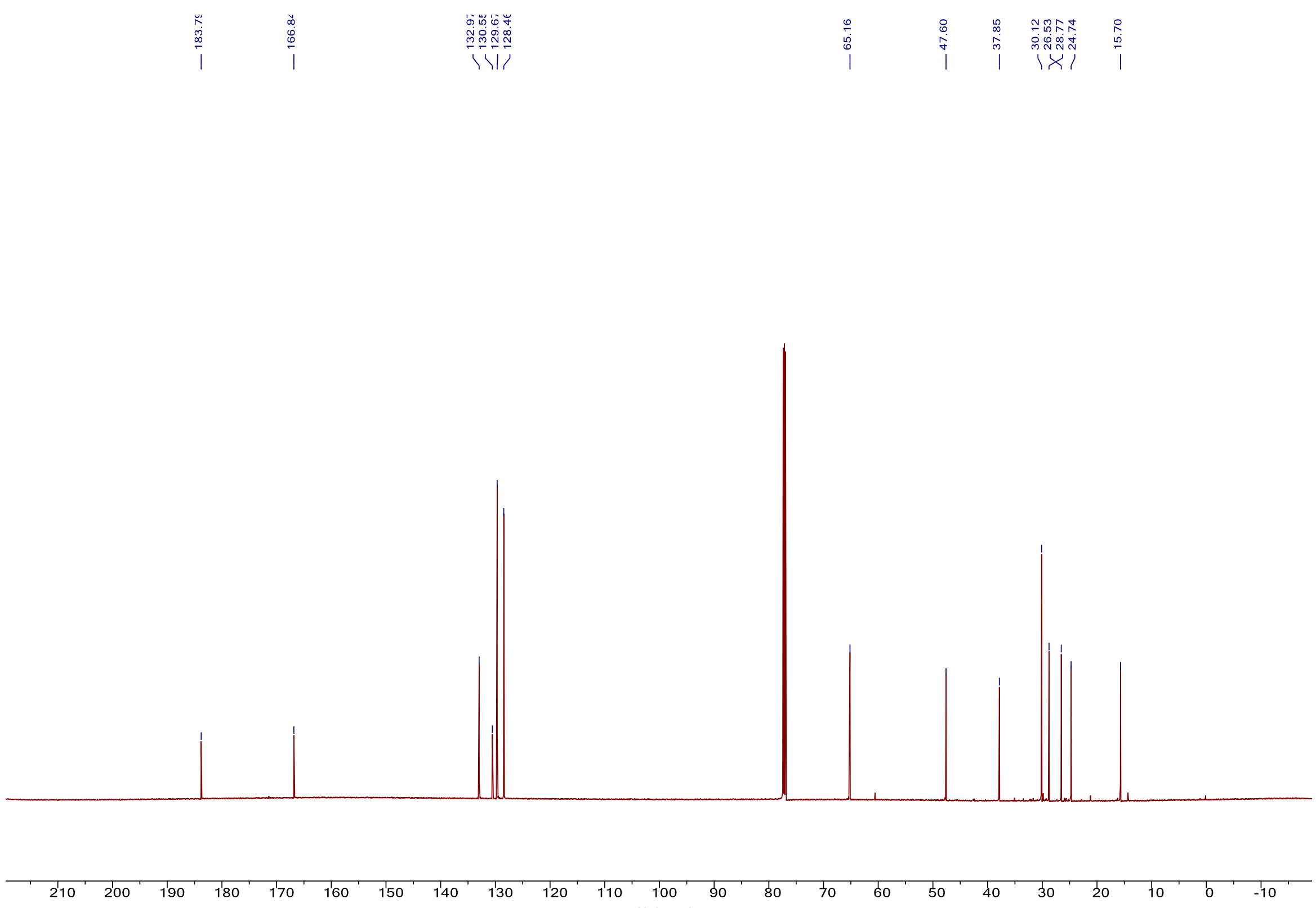

Supplementary Figure 196. ^{13}C NMR (151 MHz) of Compound (73)

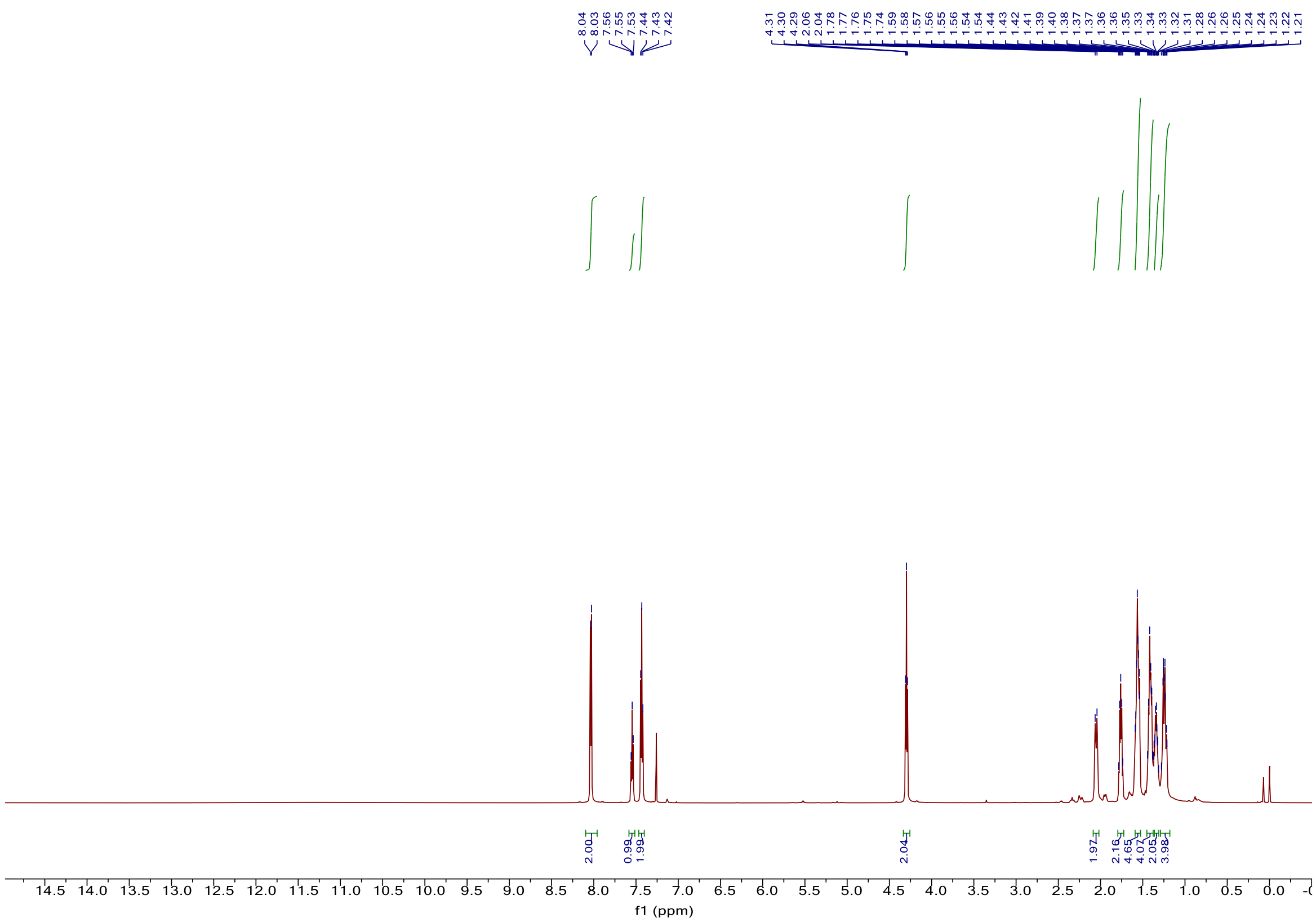

Supplementary Figure 197. ^{1}H NMR (600 MHz) of Compound (74)

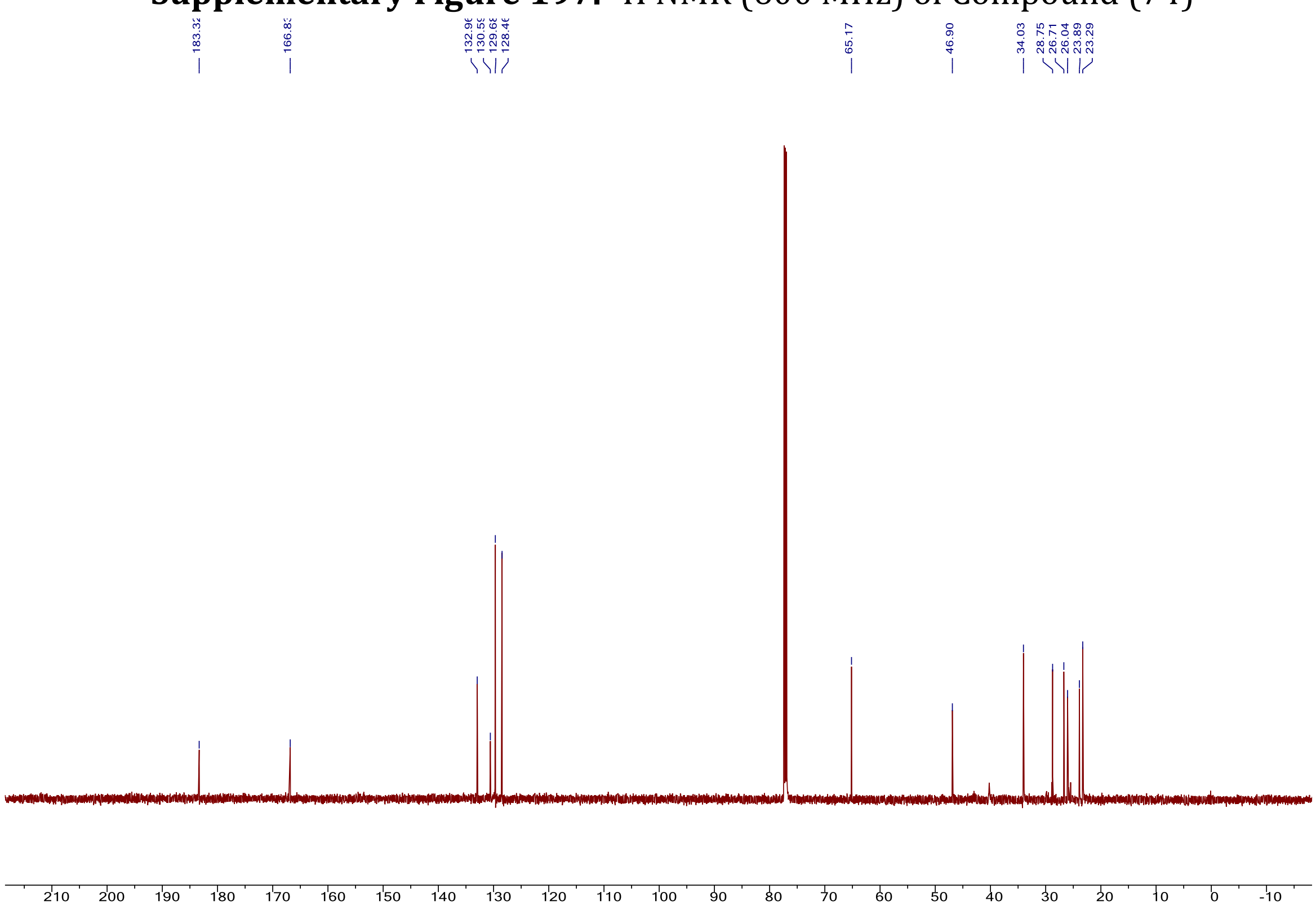

Supplementary Figure 198. ^{13}C NMR (151 MHz) of Compound (74)

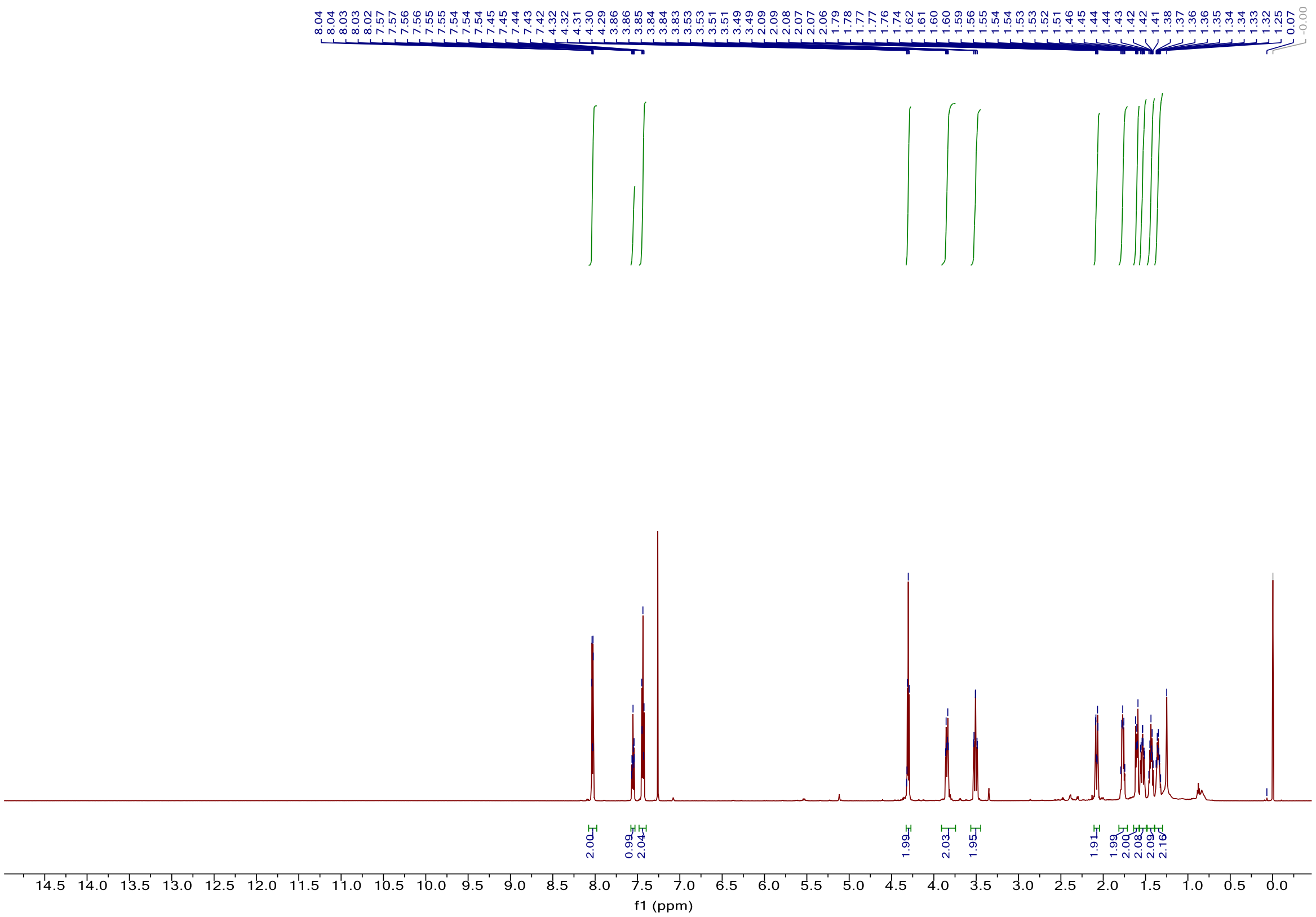

Supplementary Figure 199. ^{1}H NMR (600 MHz) of Compound (75)

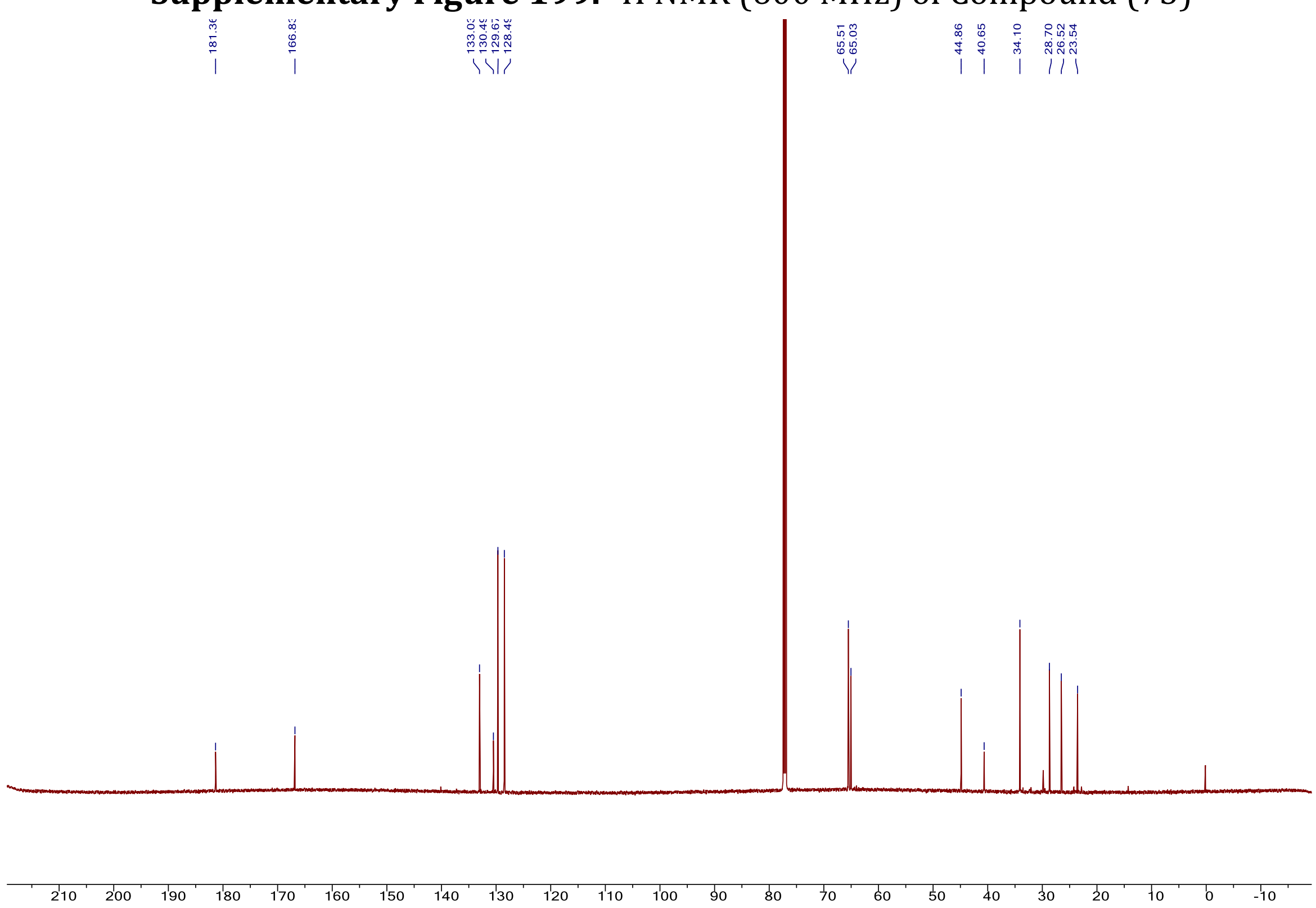

Supplementary Figure 200. ^{13}C NMR (151 MHz) of Compound (75)

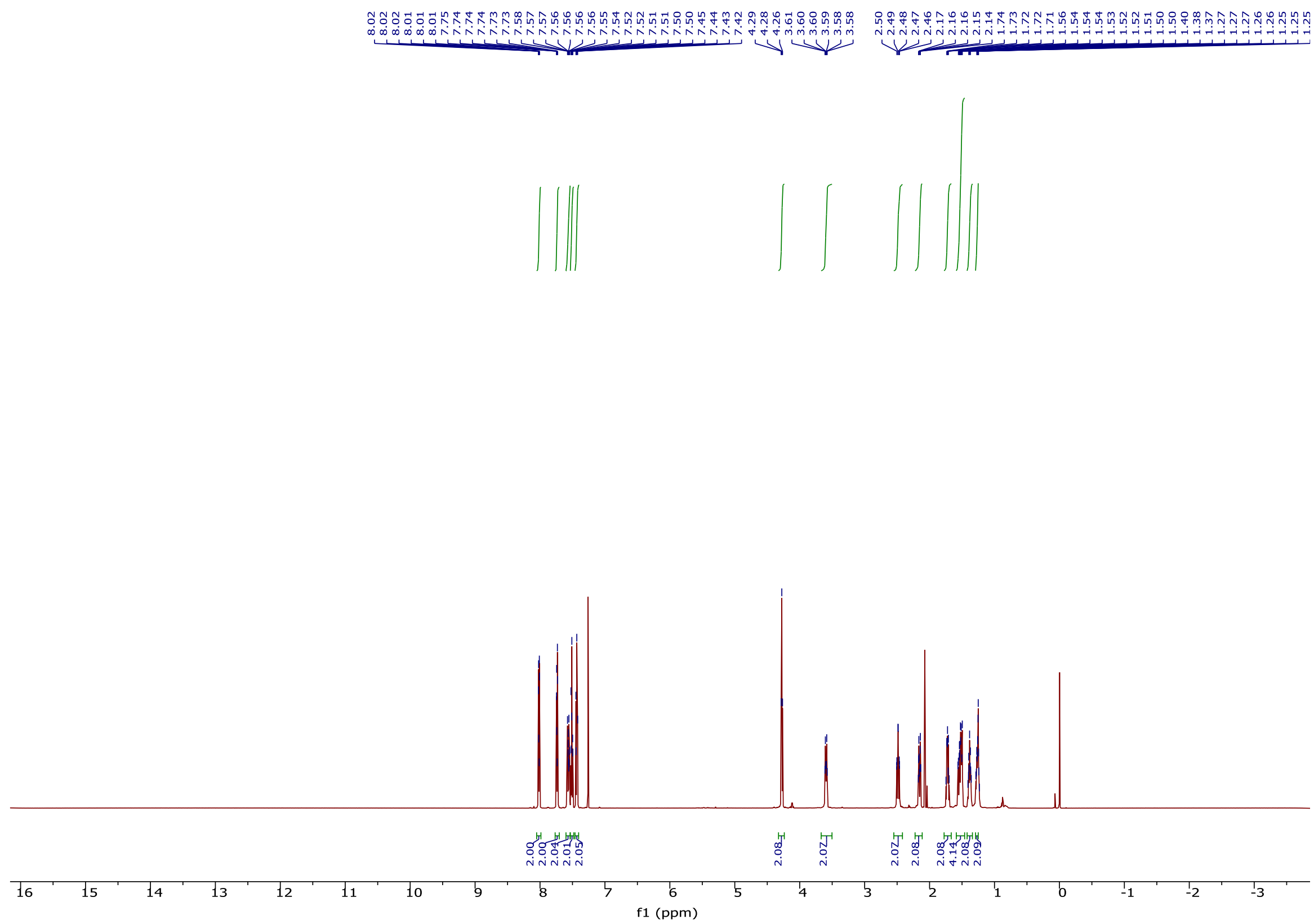

Supplementary Figure 201. ^{1}H NMR (600 MHz) of Compound (76)

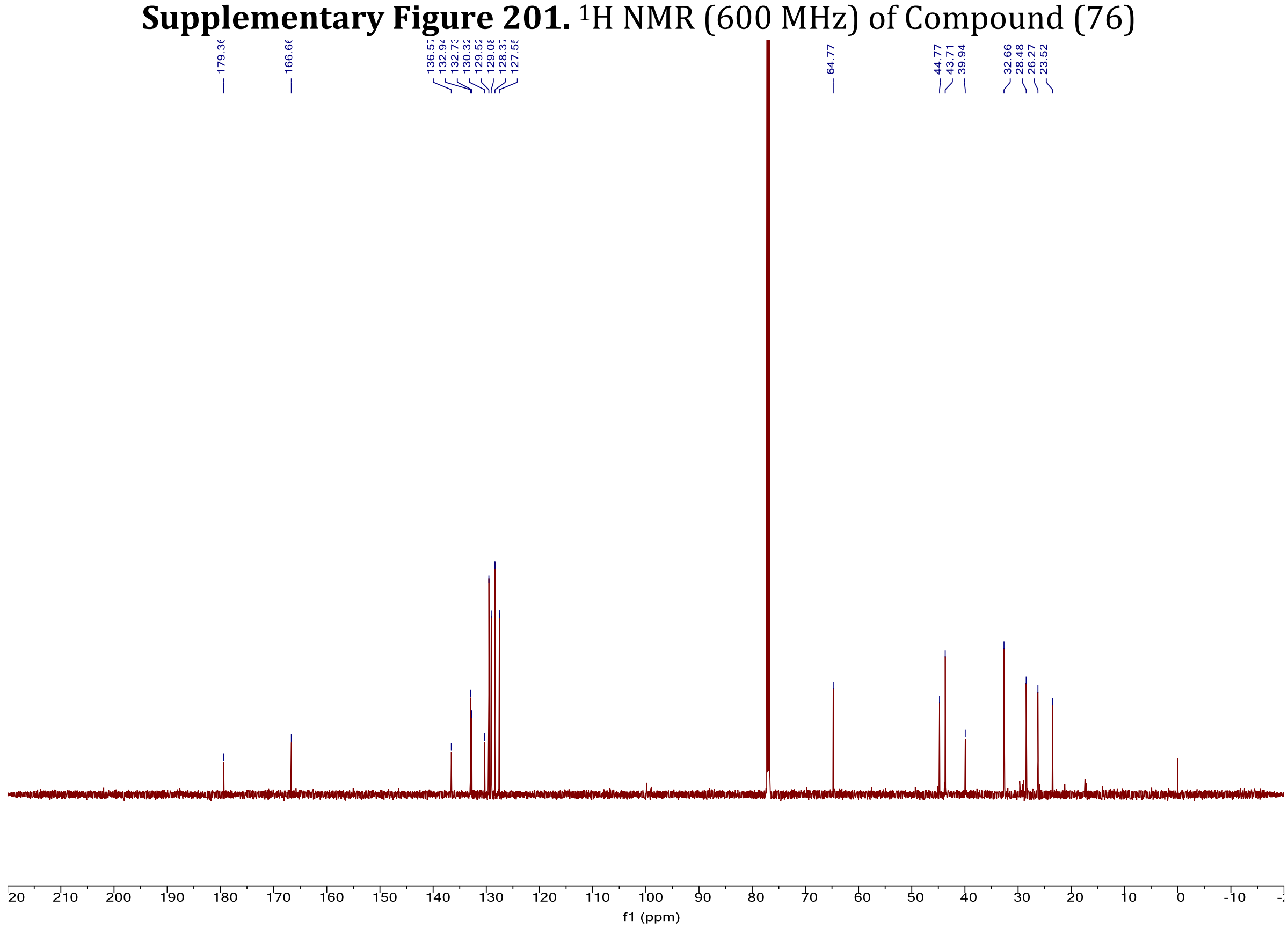

Supplementary Figure 202. ^{13}C NMR (151 MHz) of Compound (76)

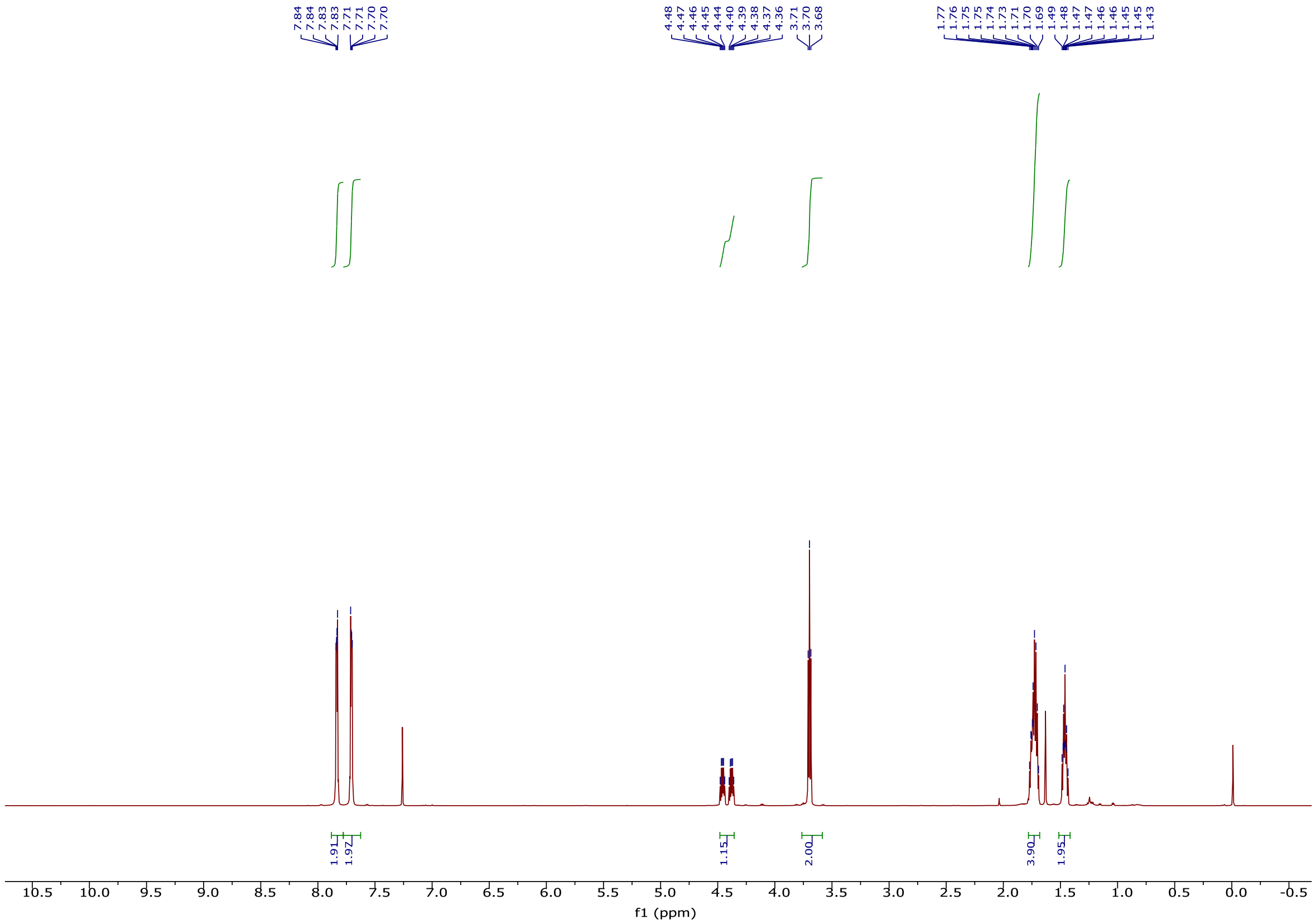

Supplementary Figure 203. ^{1}H NMR (600 MHz) of Compound (77)

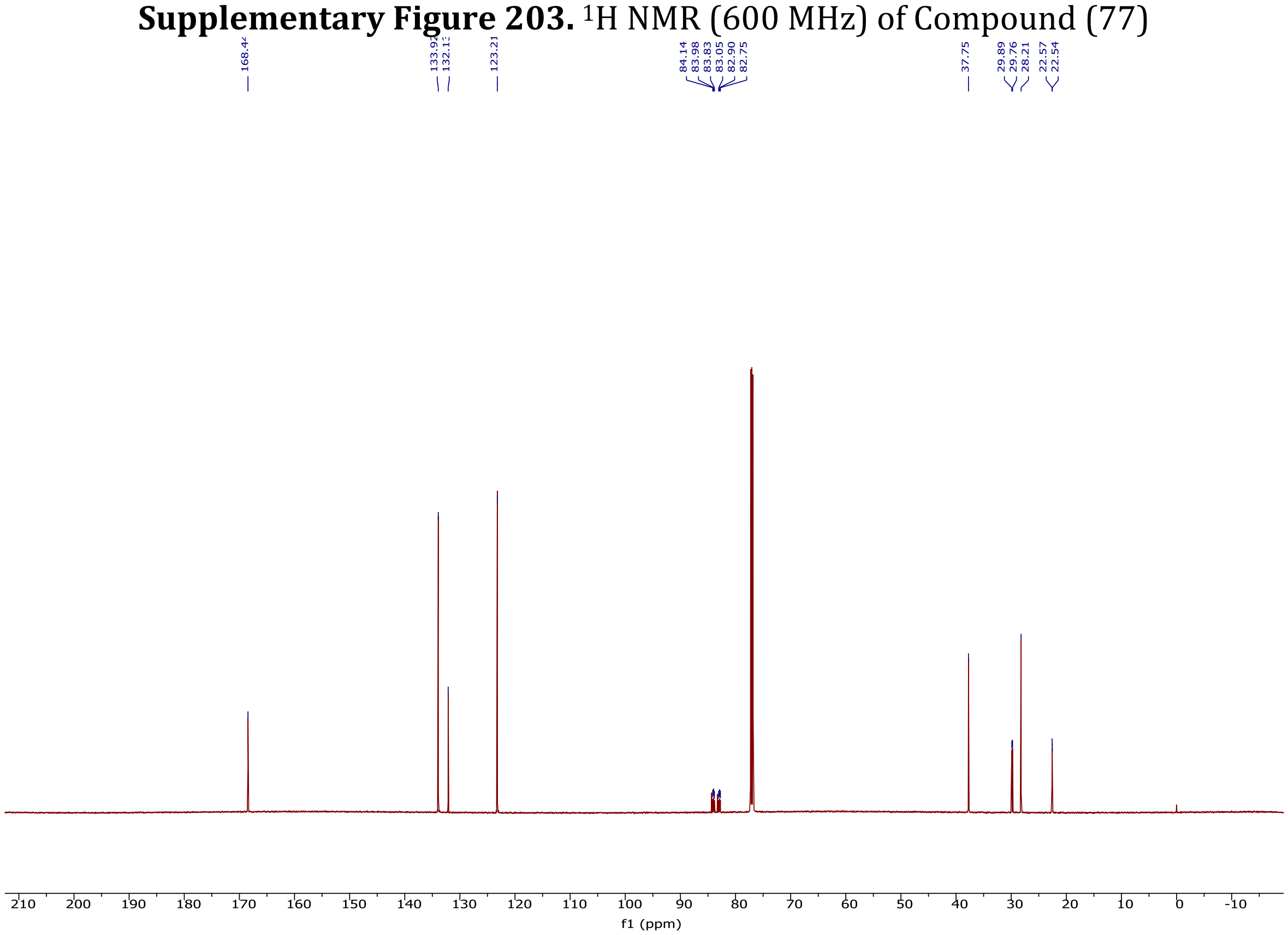

Supplementary Figure 204. ^{13}C NMR (151 MHz) of Compound (77)

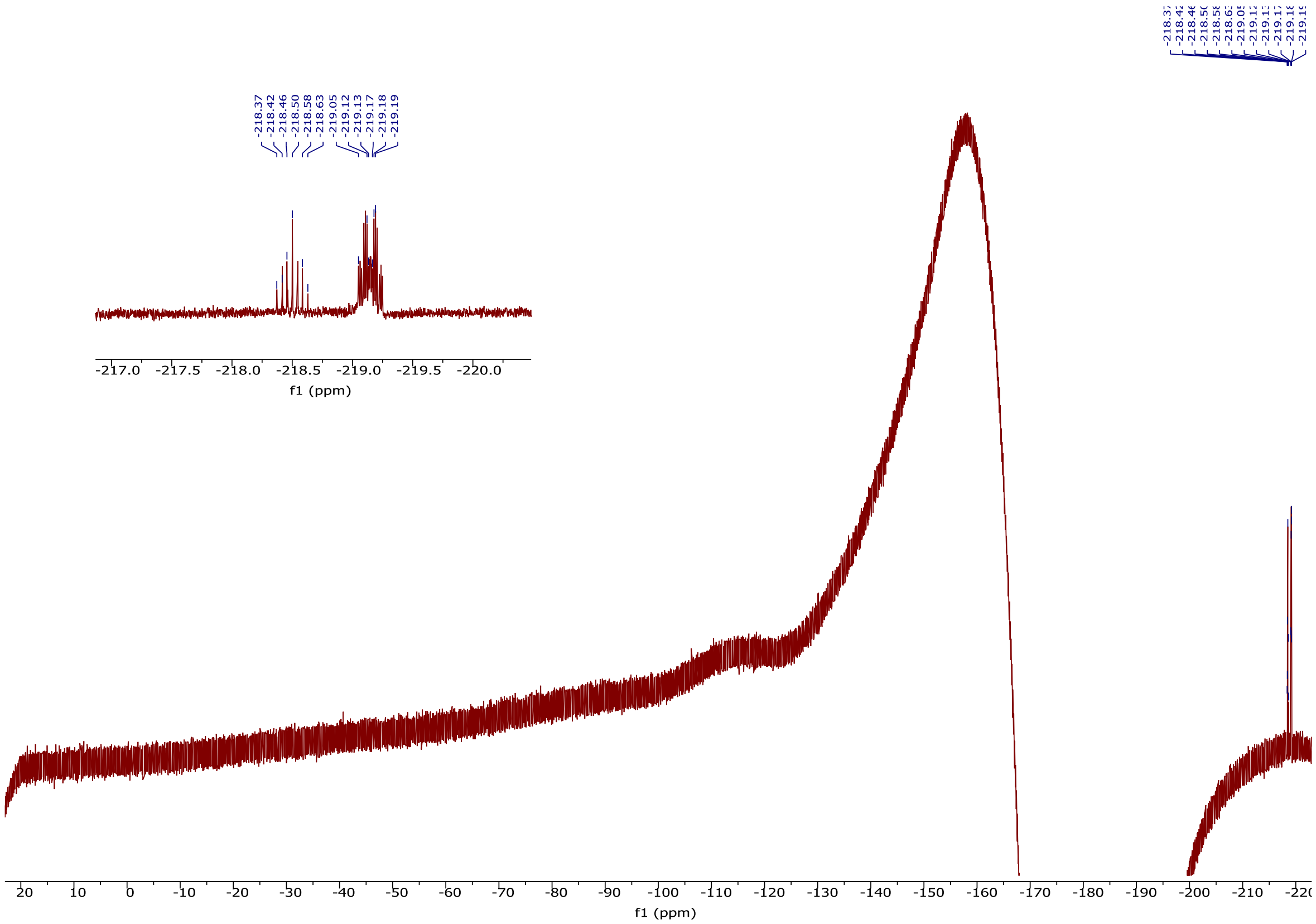

Supplementary Figure 205. ^{19}F NMR (564 MHz) of Compound (77)

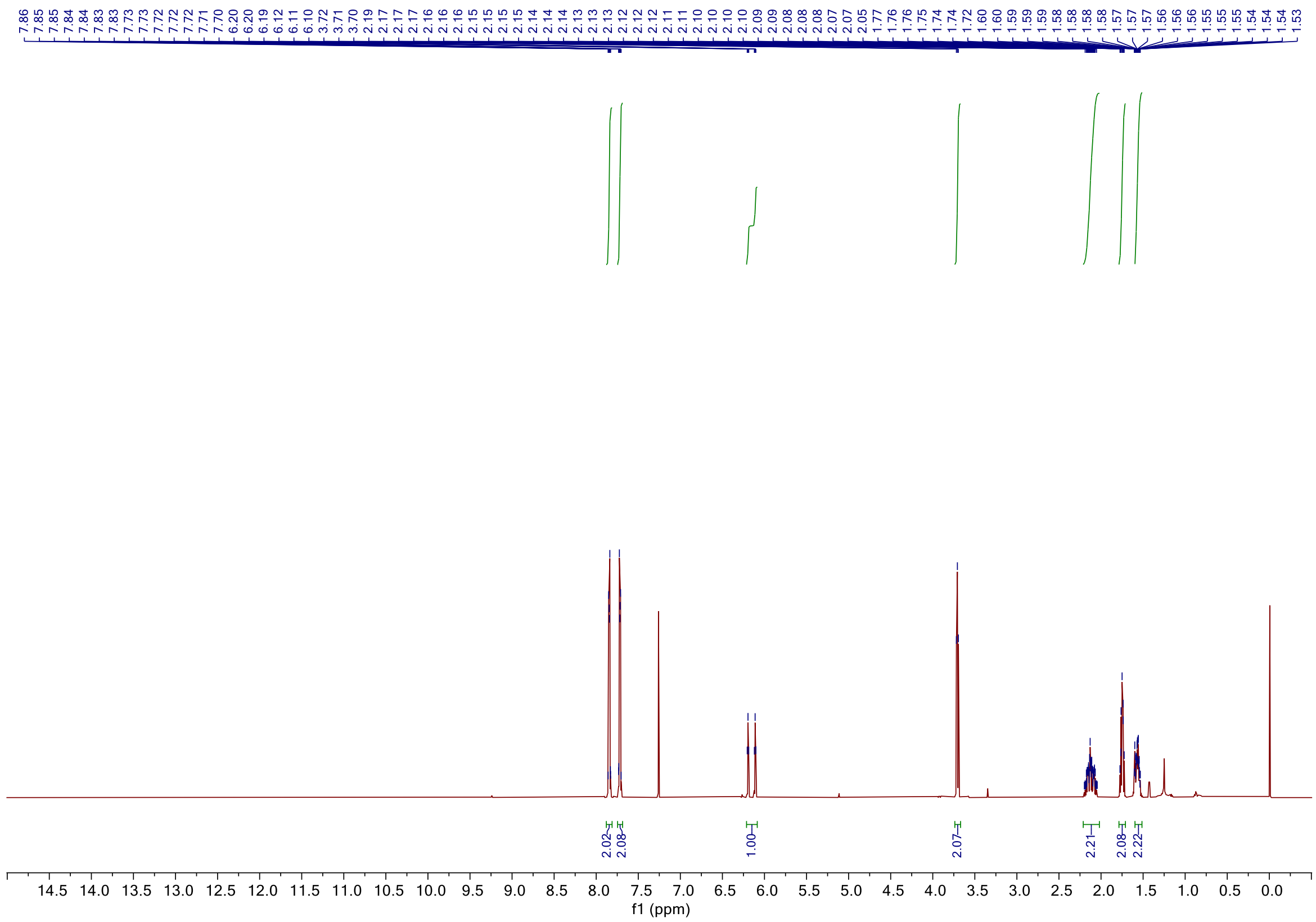

Supplementary Figure 206. ^{1}H NMR (600 MHz) of Compound (78)

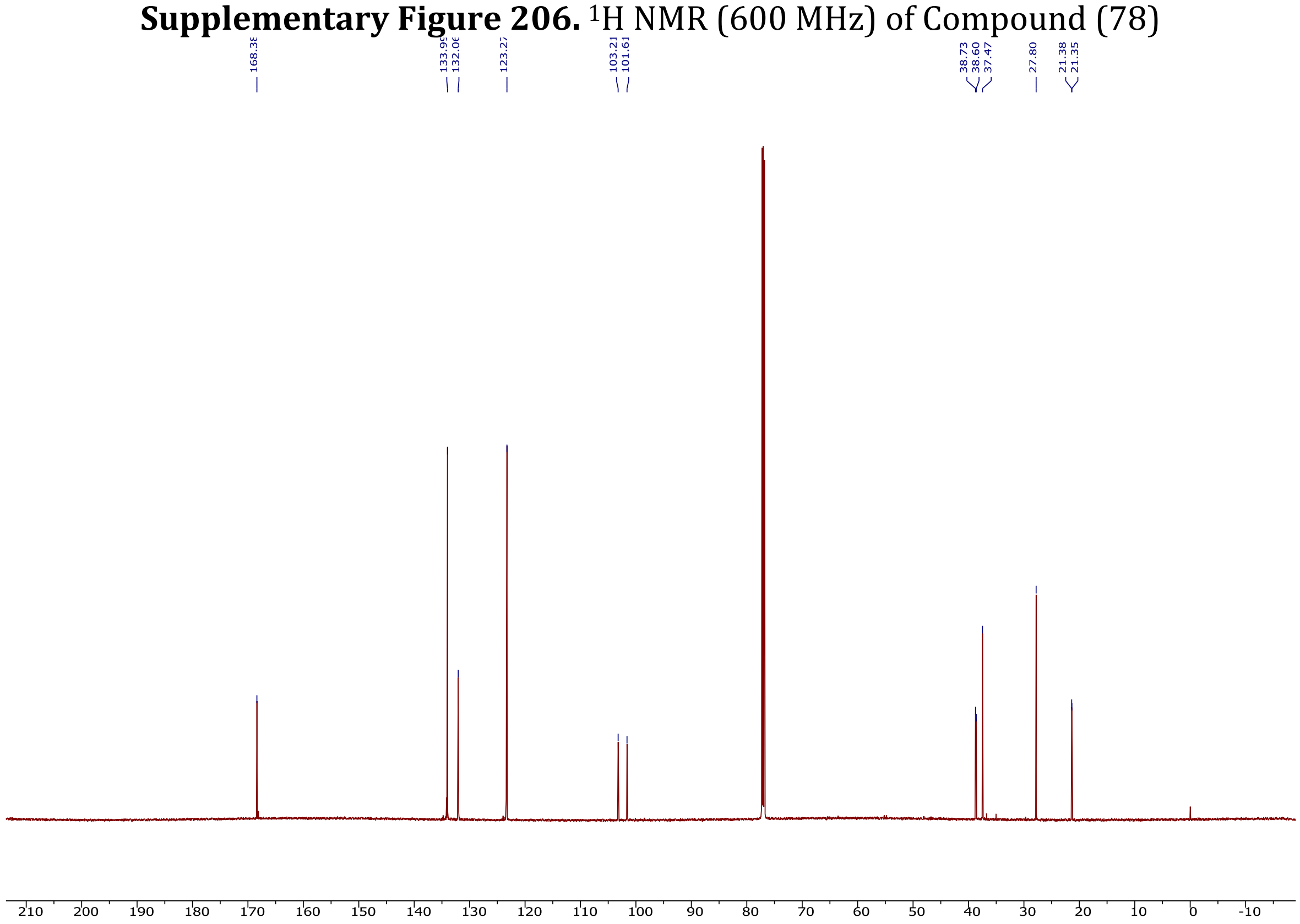

Supplementary Figure 207. ^{13}C NMR (151 MHz) of Compound (78)

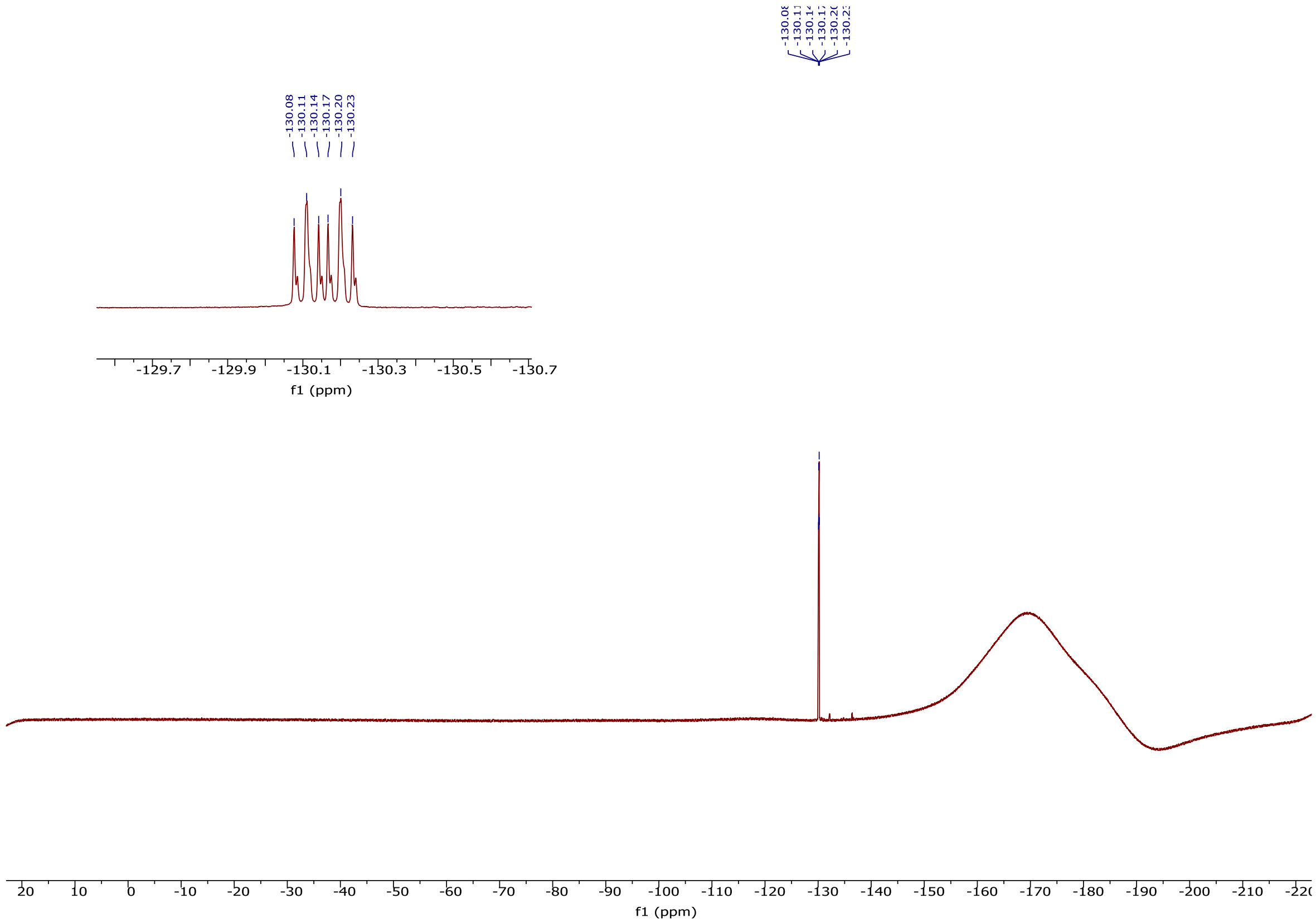

Supplementary Figure 208. ^{19}F NMR (564 MHz) of Compound (78)

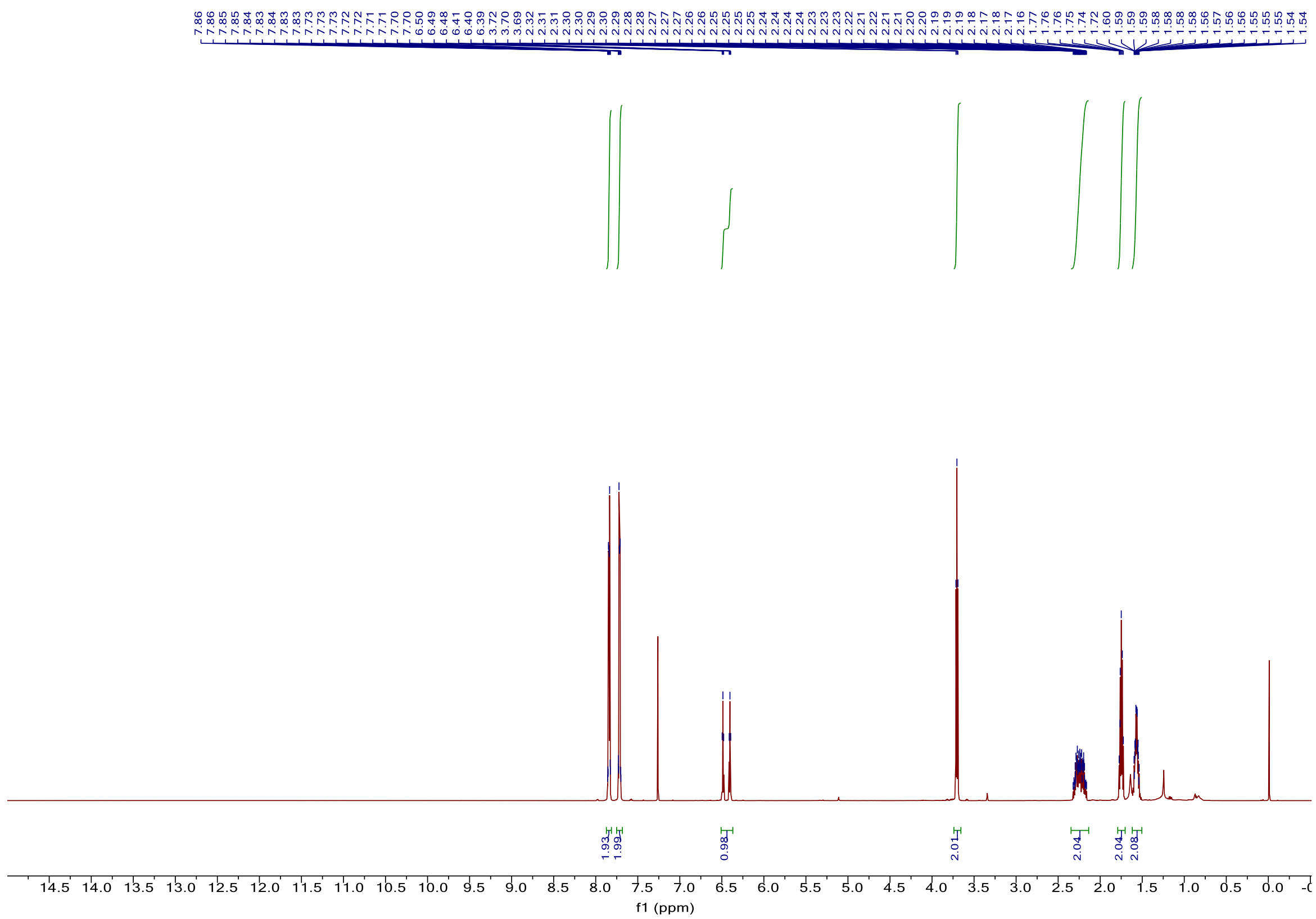

Supplementary Figure 209. ^{1}H NMR (600 MHz) of Compound (78')

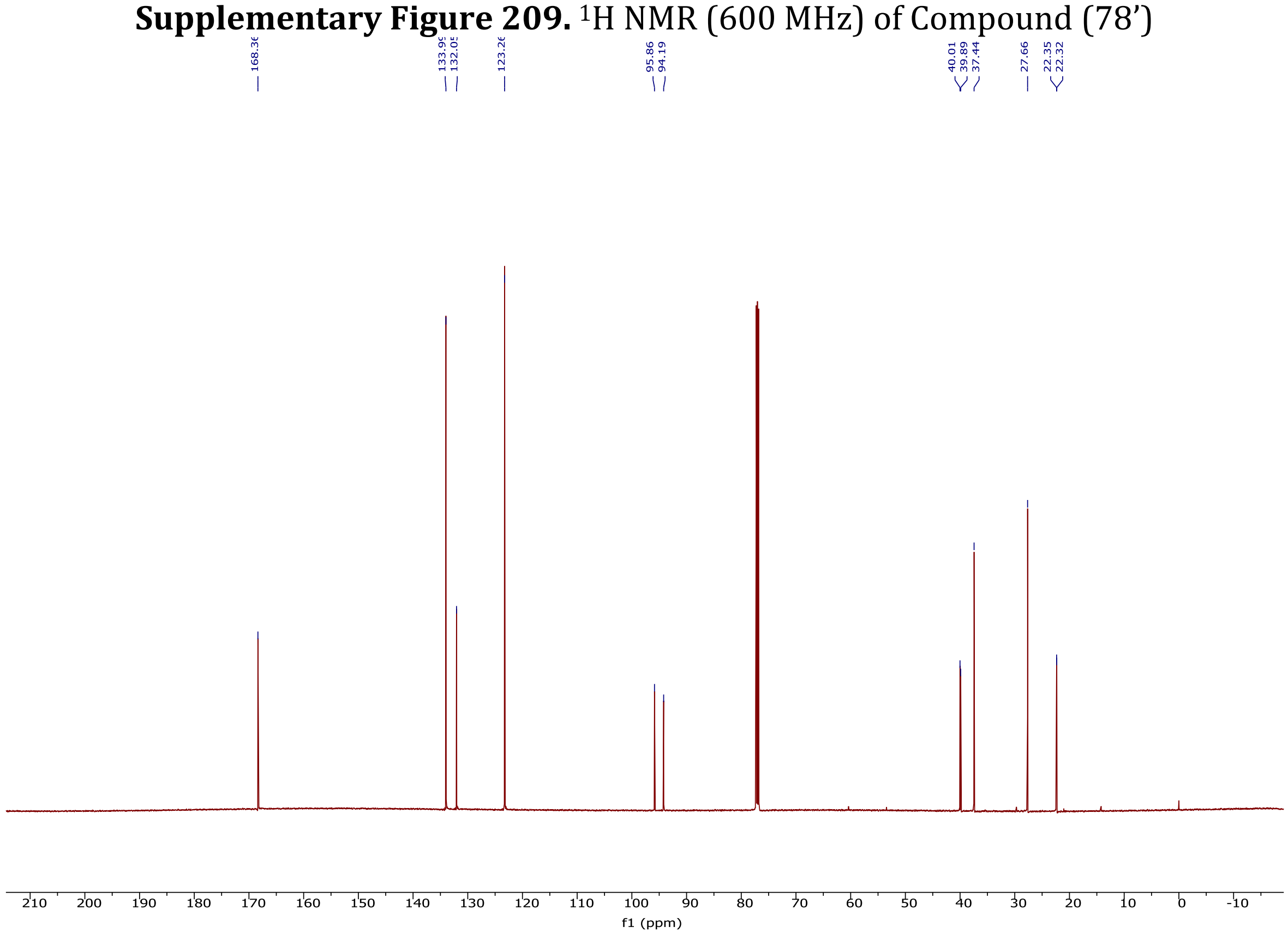

Supplementary Figure 210. ^{13}C NMR (151 MHz) of Compound (78')

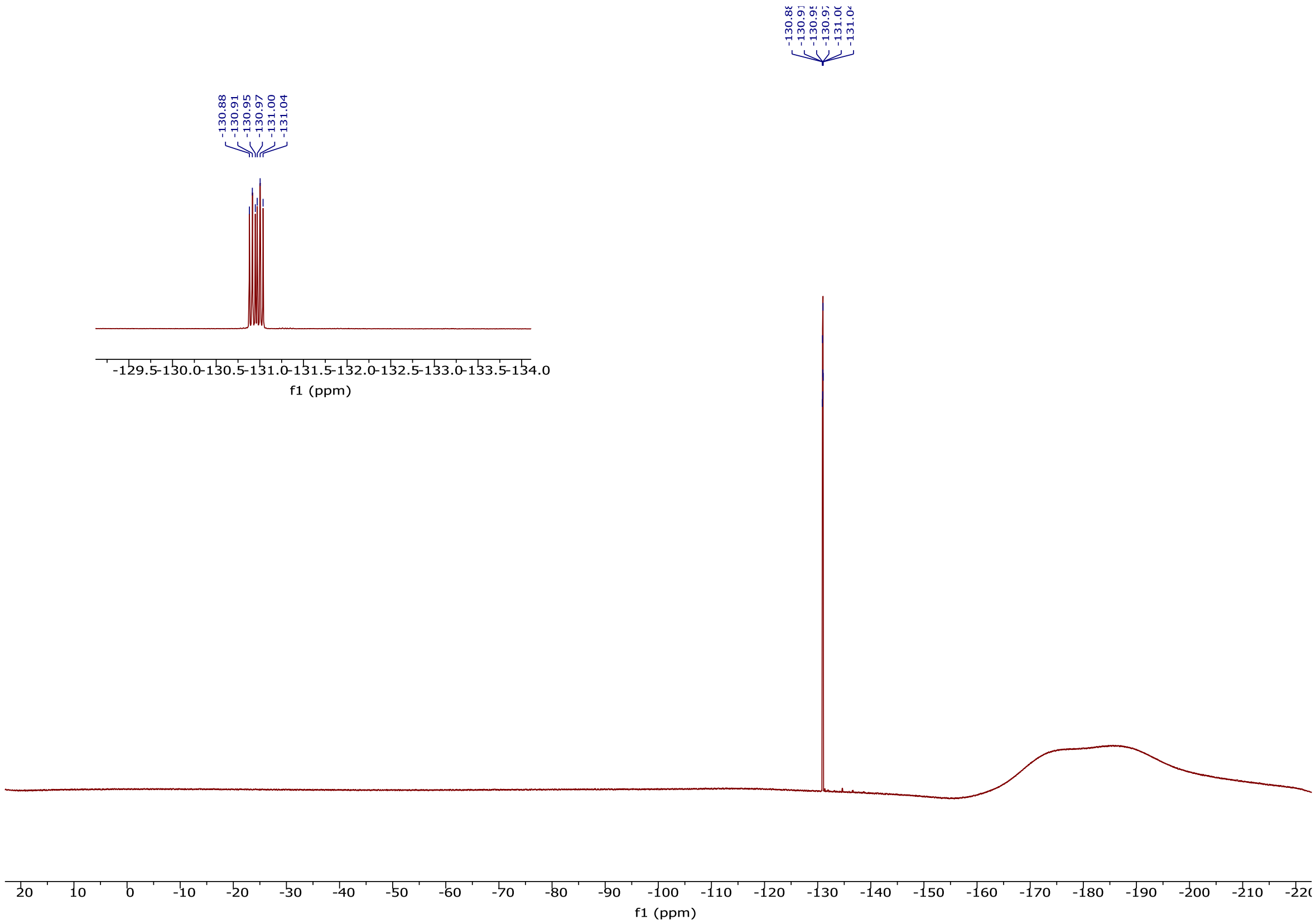

Supplementary Figure 211. ^{19}F NMR (564 MHz) of Compound (78')

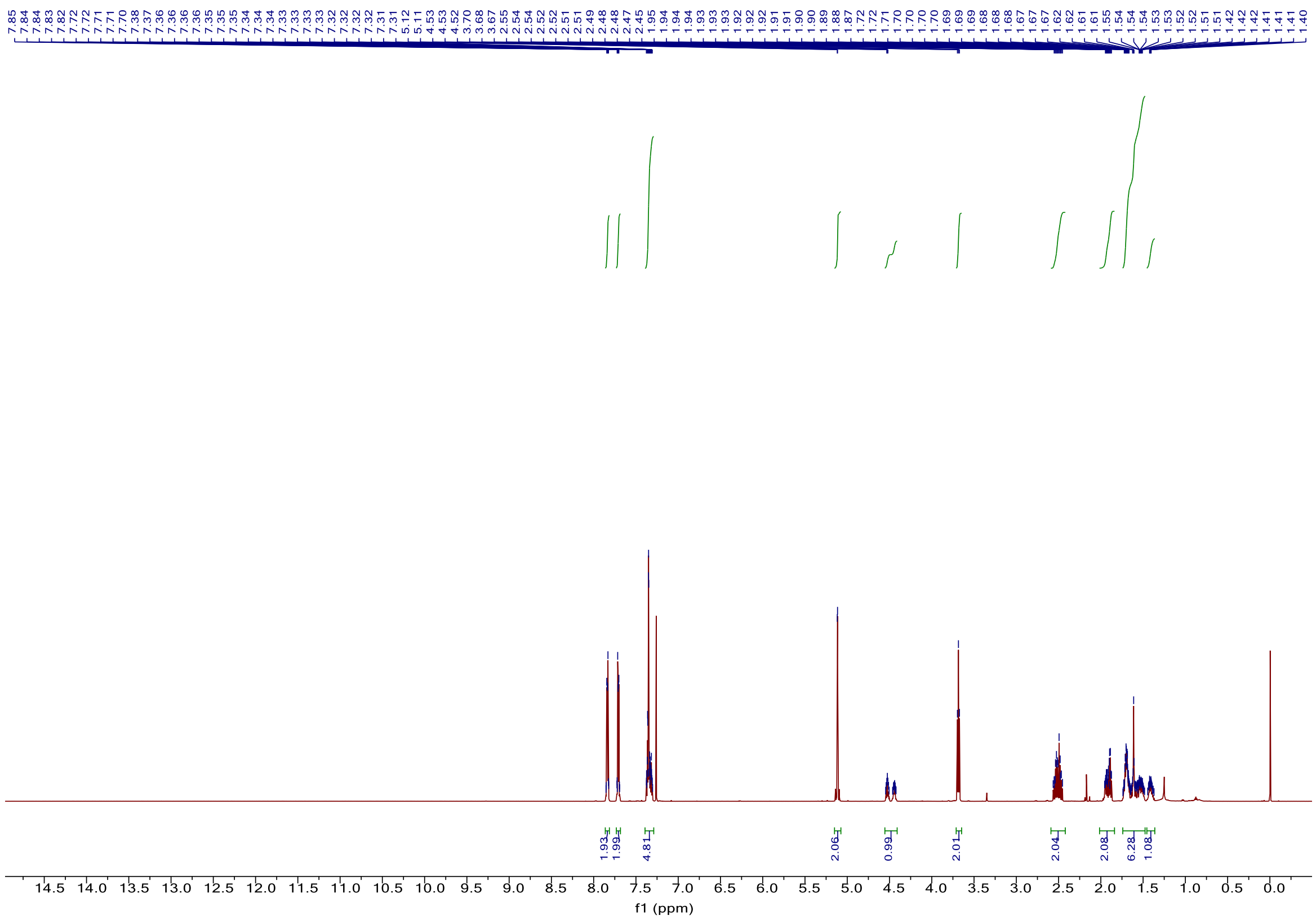

Supplementary Figure 212. ^{1}H NMR (600 MHz) of Compound (79)

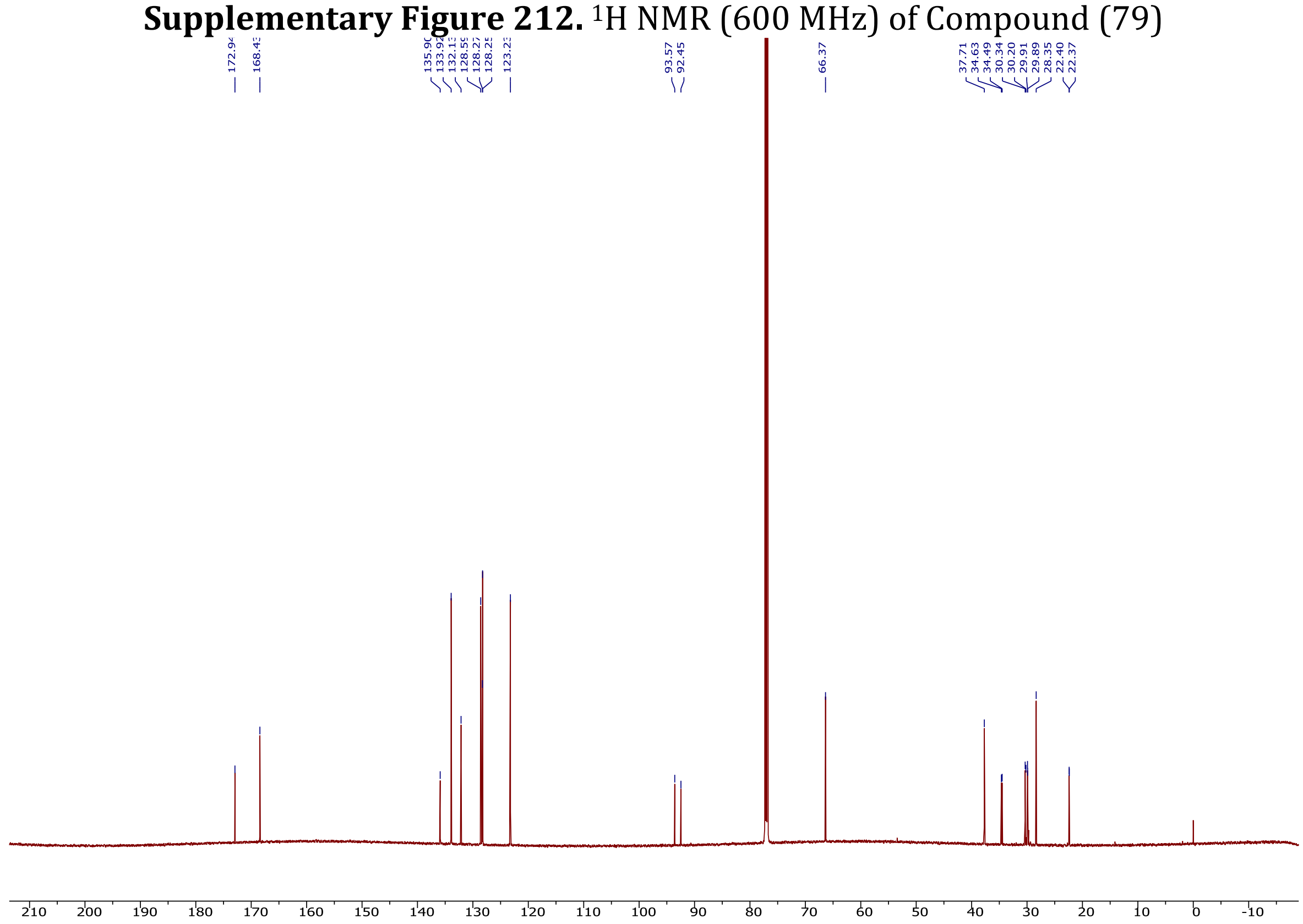

Supplementary Figure 213. ^{13}C NMR (151 MHz) of Compound (79)

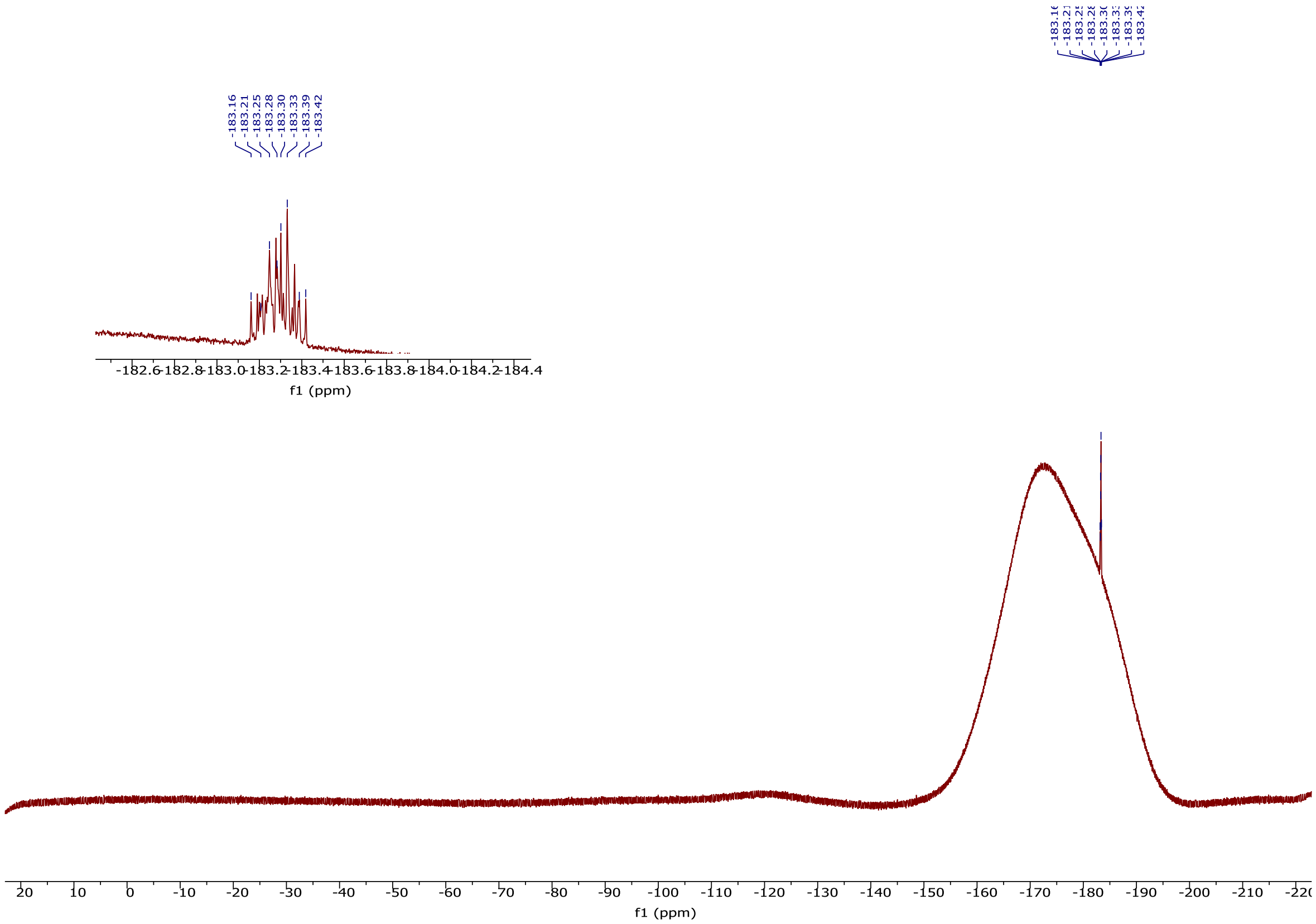

Supplementary Figure 214. ^{19}F NMR (564 MHz) of Compound (79)

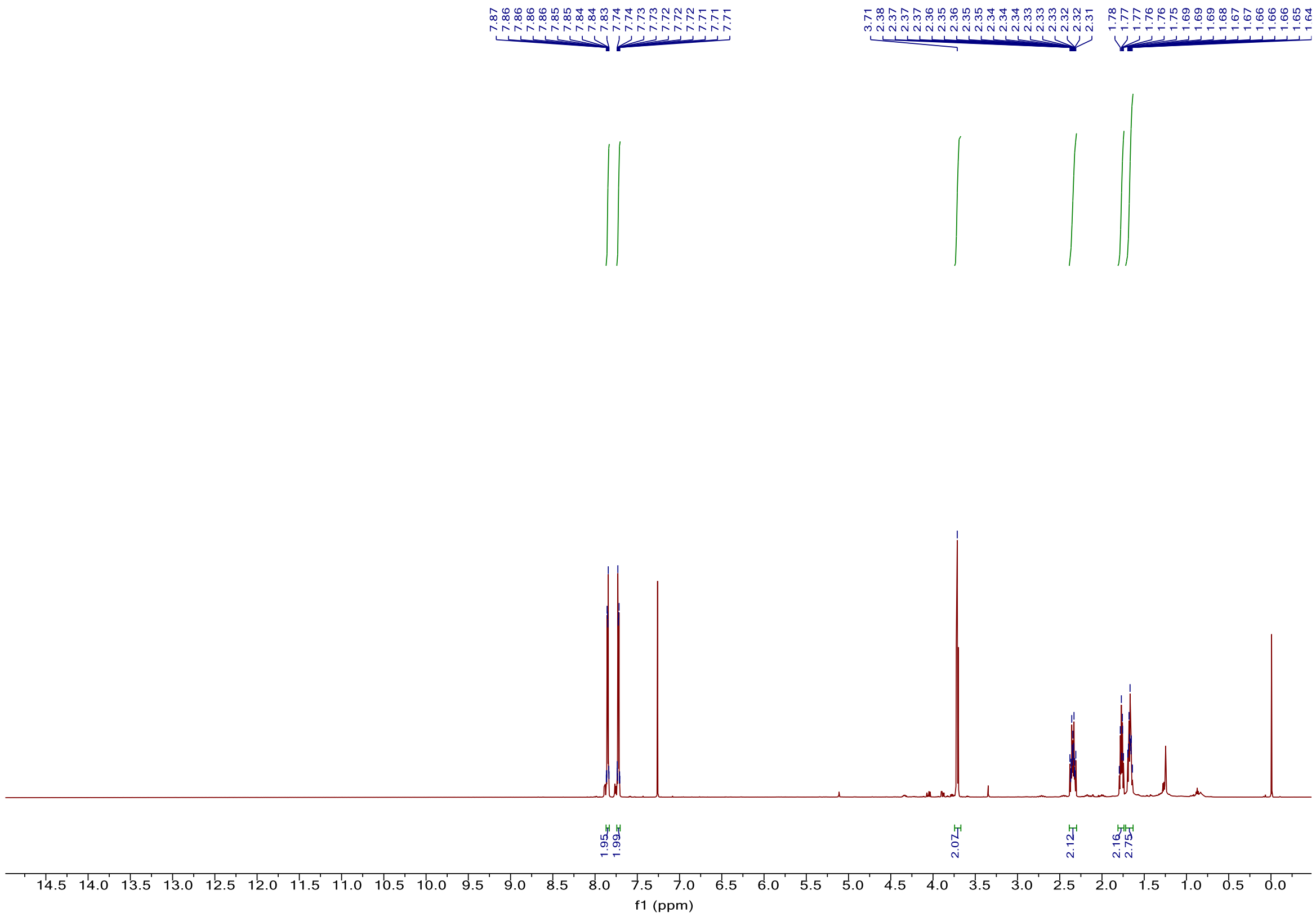

Supplementary Figure 215. ^{1}H NMR (600 MHz) of Compound (80)

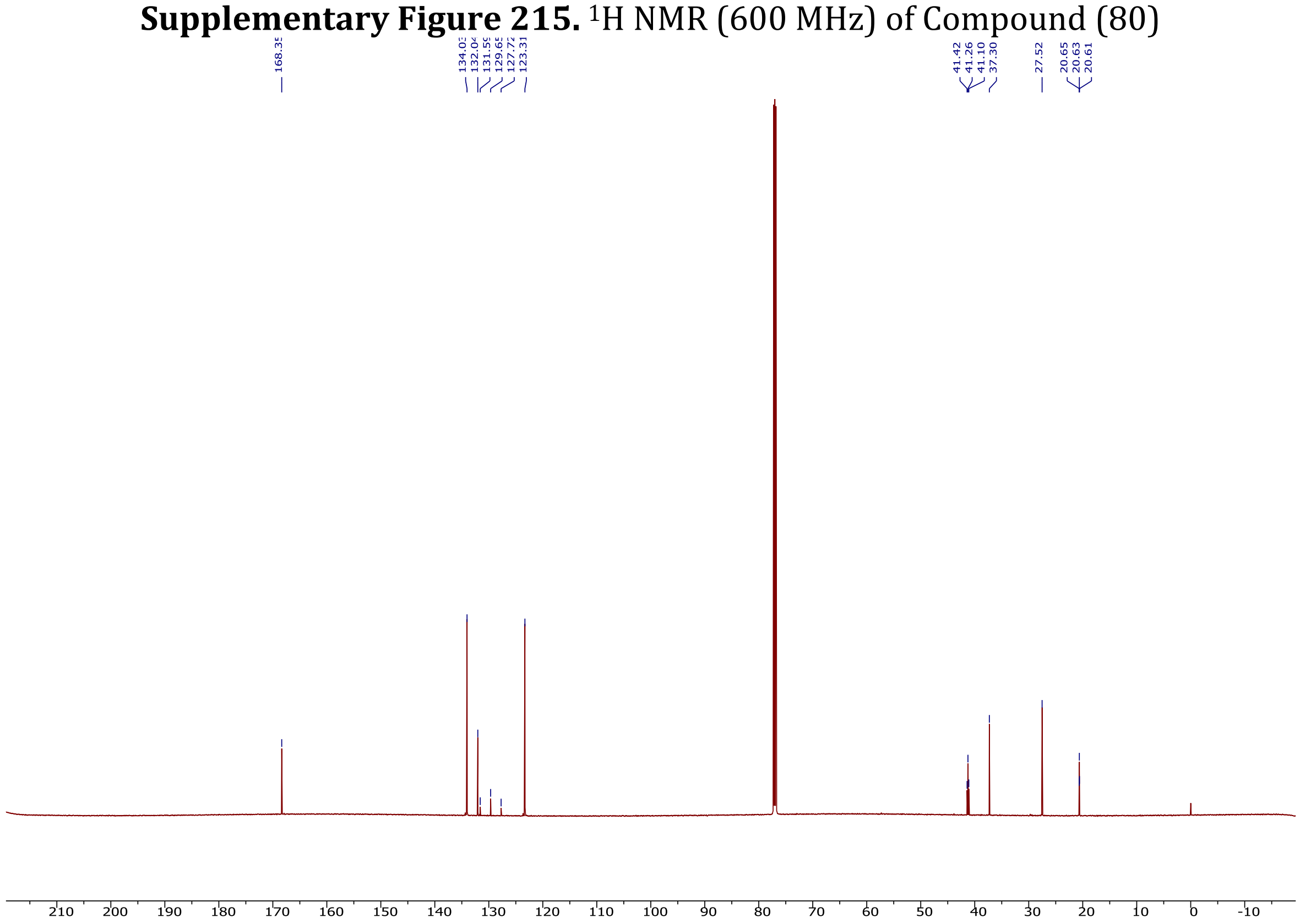

Supplementary Figure 216. ^{13}C NMR (151 MHz) of Compound (80)

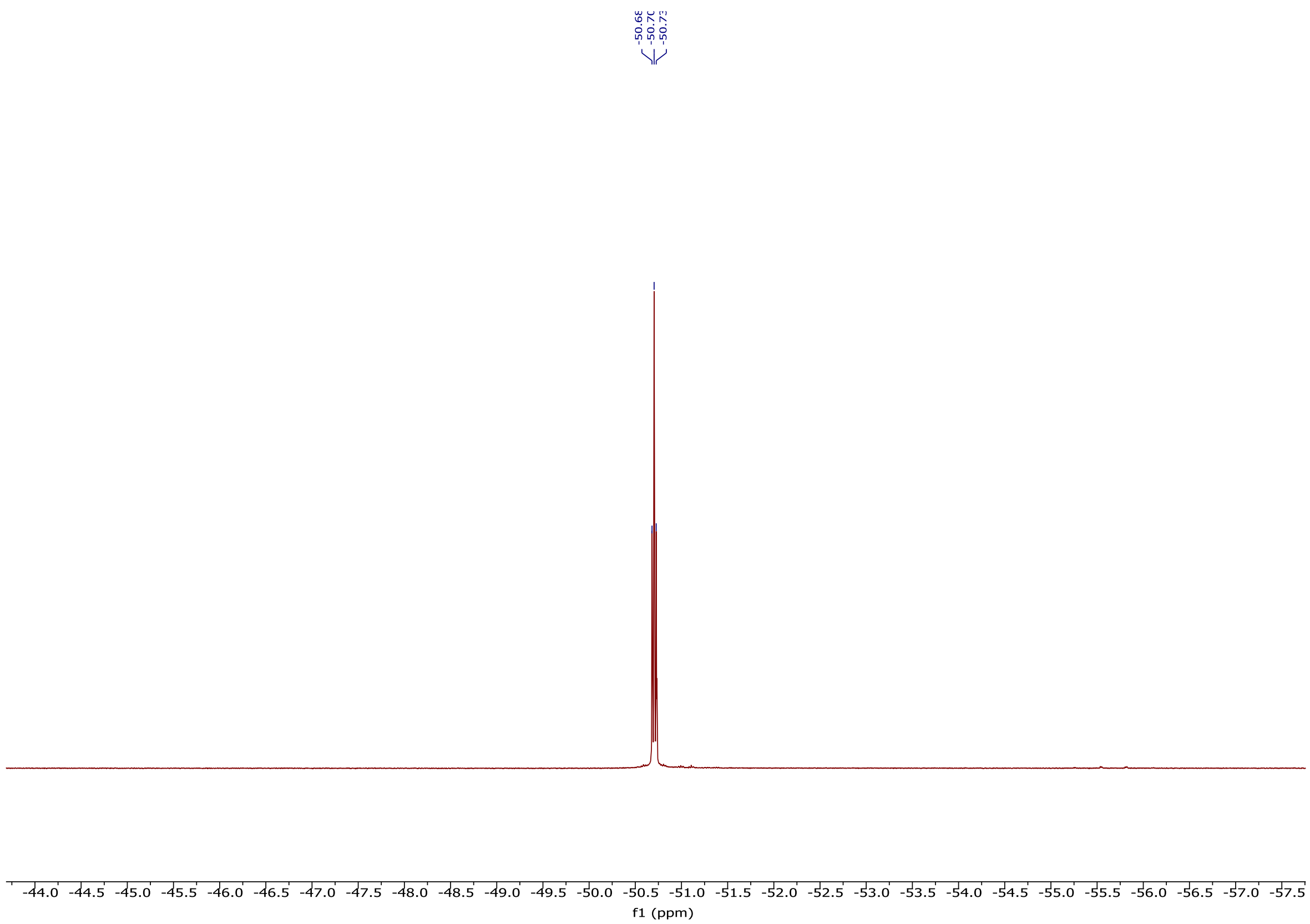

Supplementary Figure 217. ^{19}F NMR (564 MHz) of Compound (80)

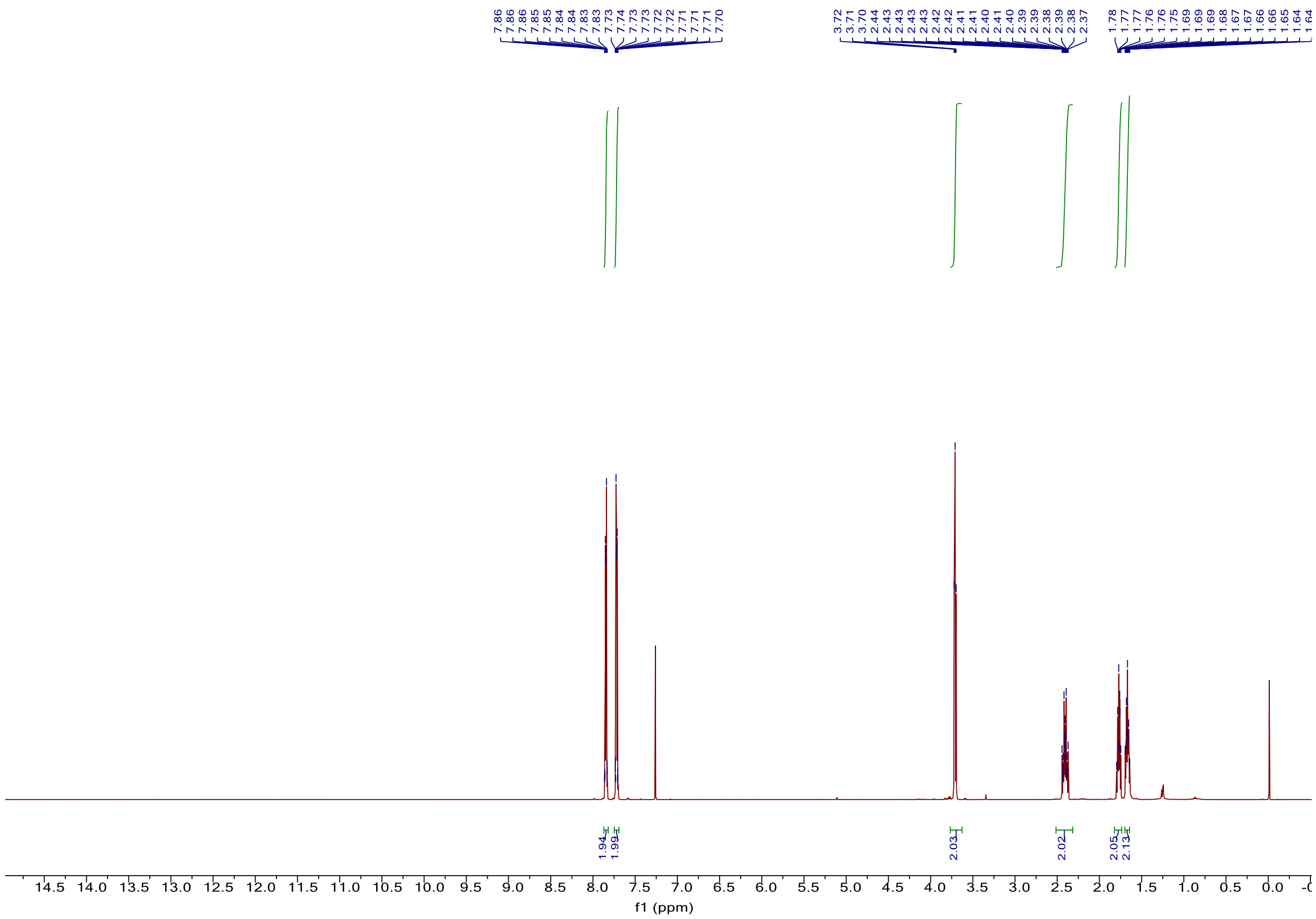

Supplementary Figure 218. ^{1}H NMR (600 MHz) of Compound (80')

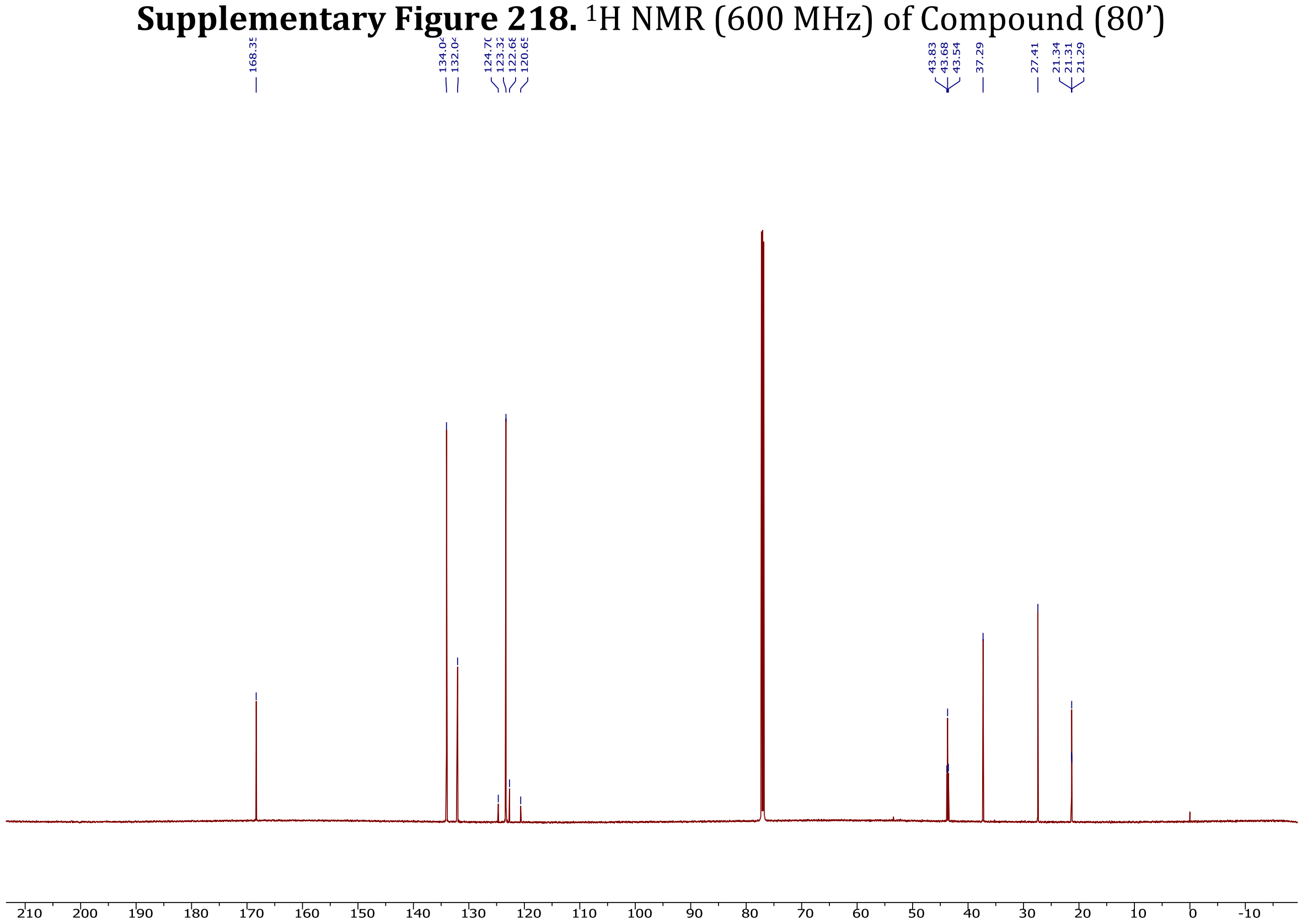

Supplementary Figure 219. ^{13}C NMR (151 MHz) of Compound (80')

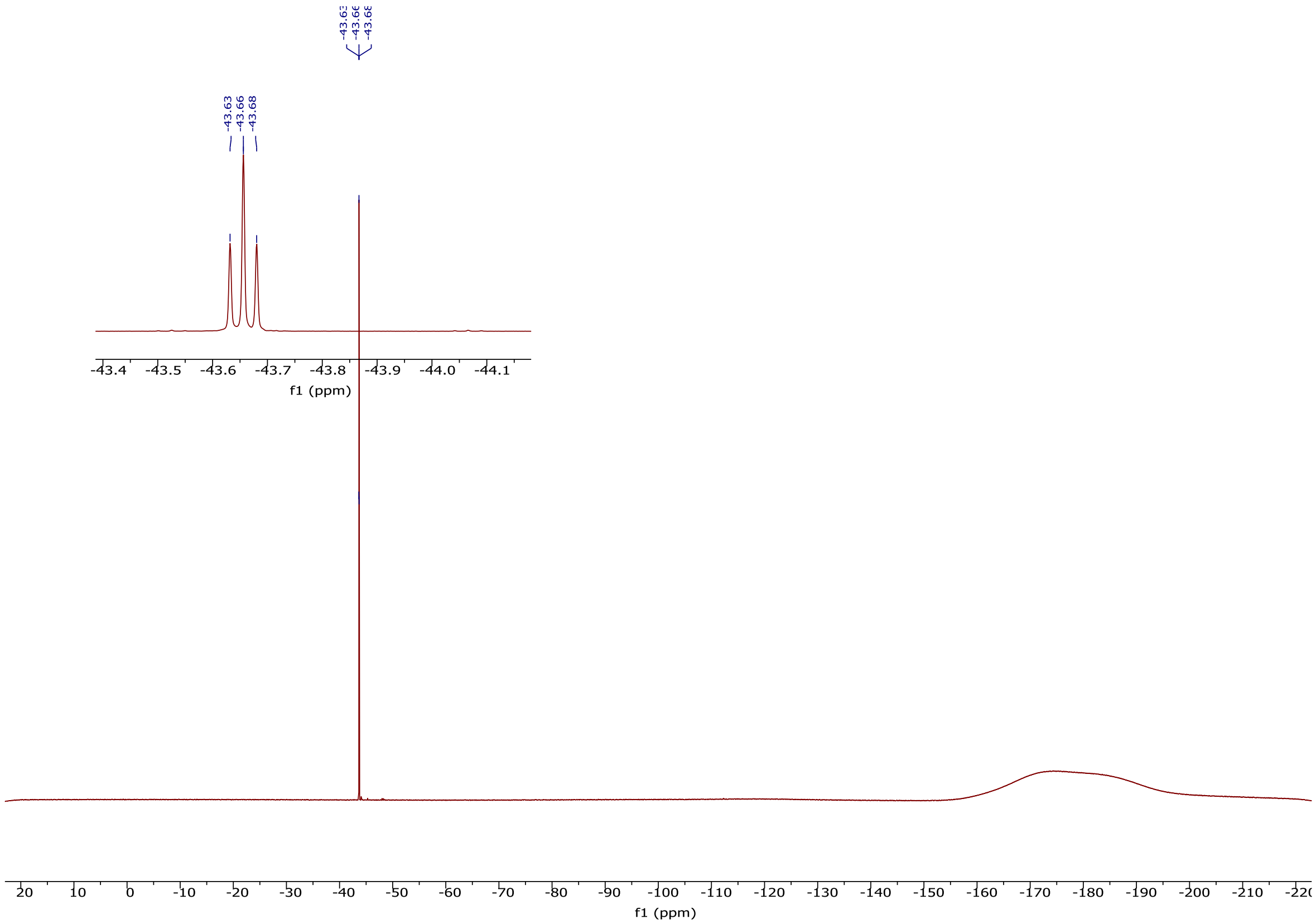

Supplementary Figure 220. ^{19}F NMR (564 MHz) of Compound (80’)

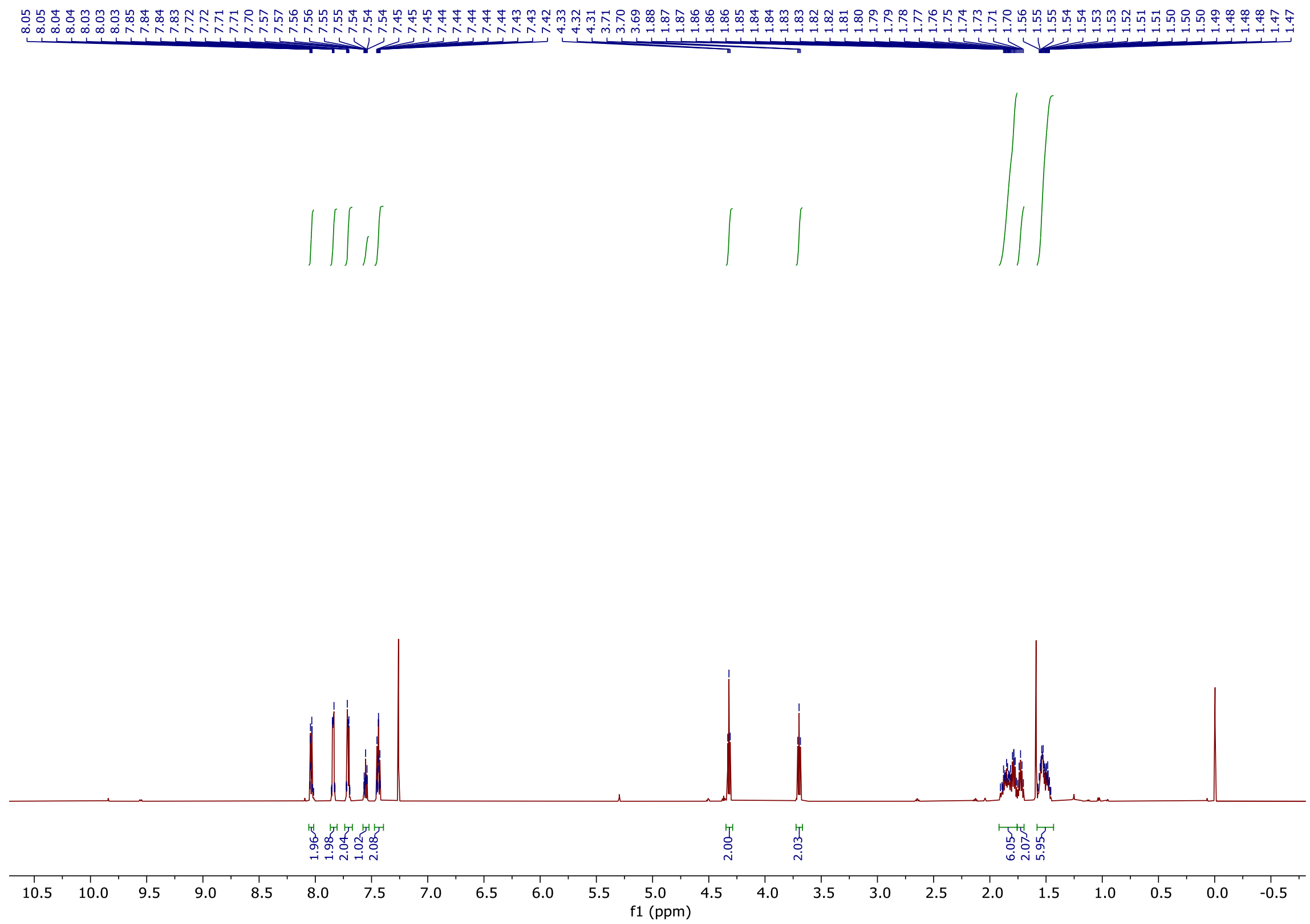

Supplementary Figure 221. ^{1}H NMR (600 MHz) of Compound (81)

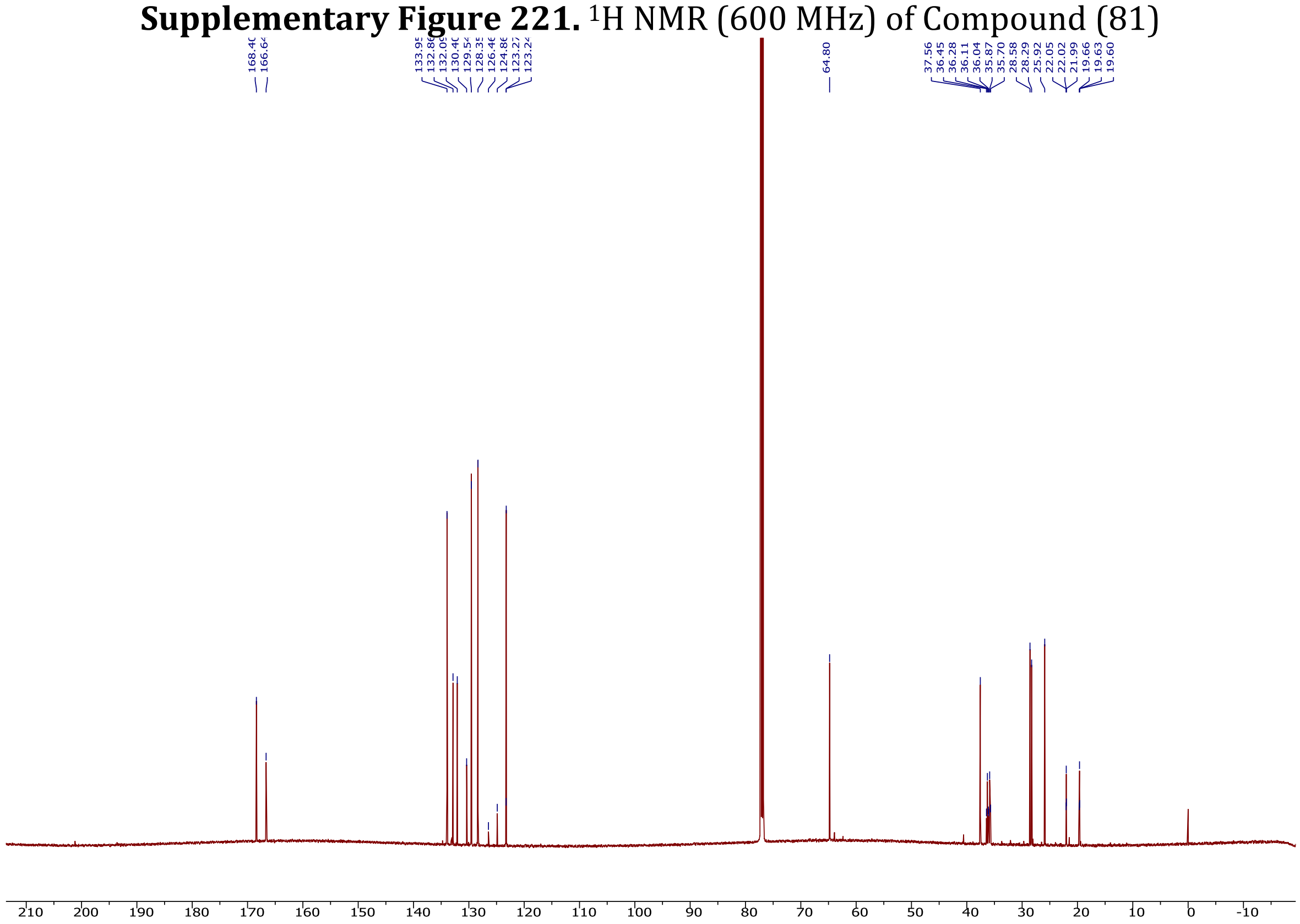

Supplementary Figure 222. ^{13}C NMR (151 MHz) of Compound (81)

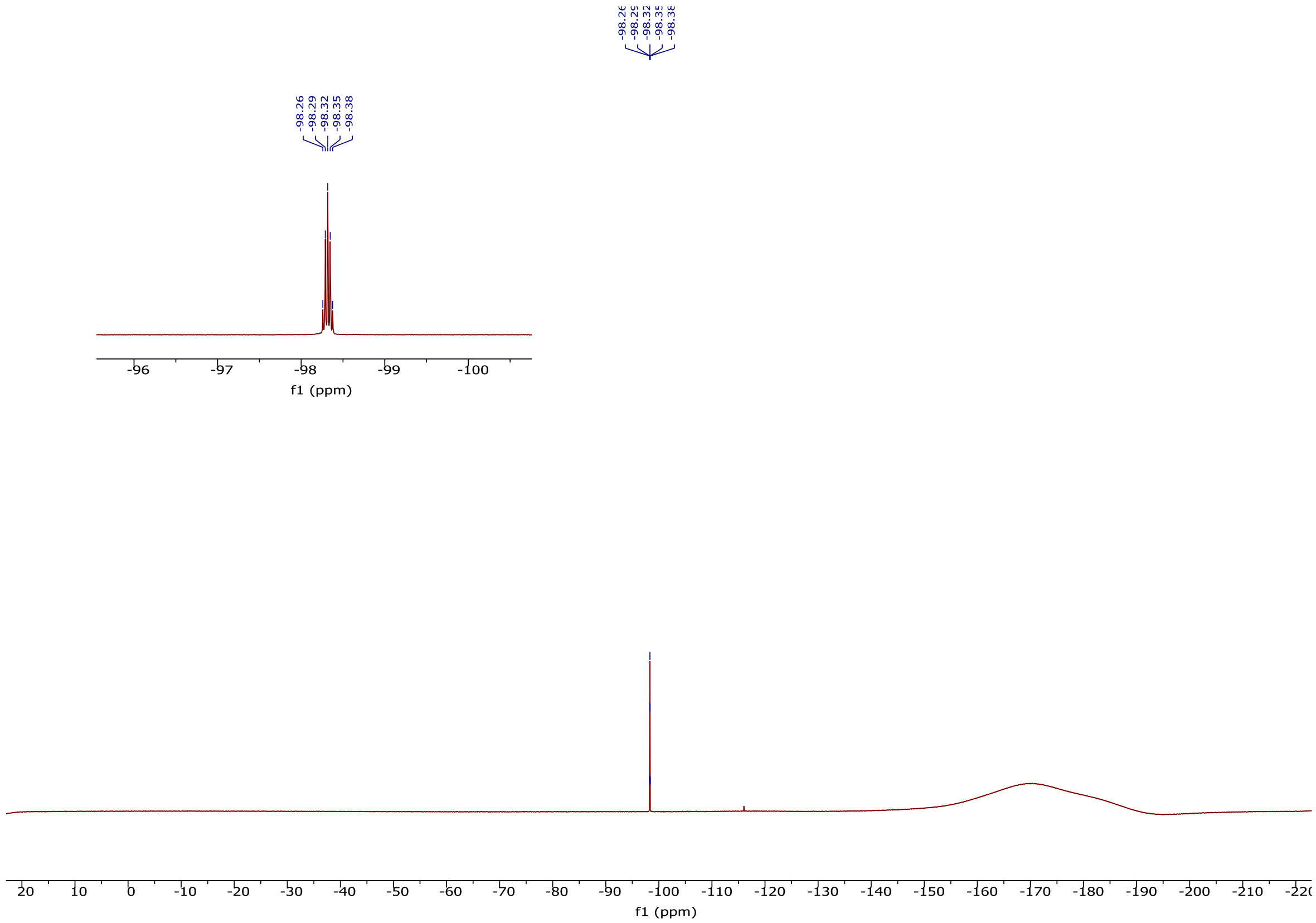

Supplementary Figure 223. ^{19}F NMR (564 MHz) of Compound (81)

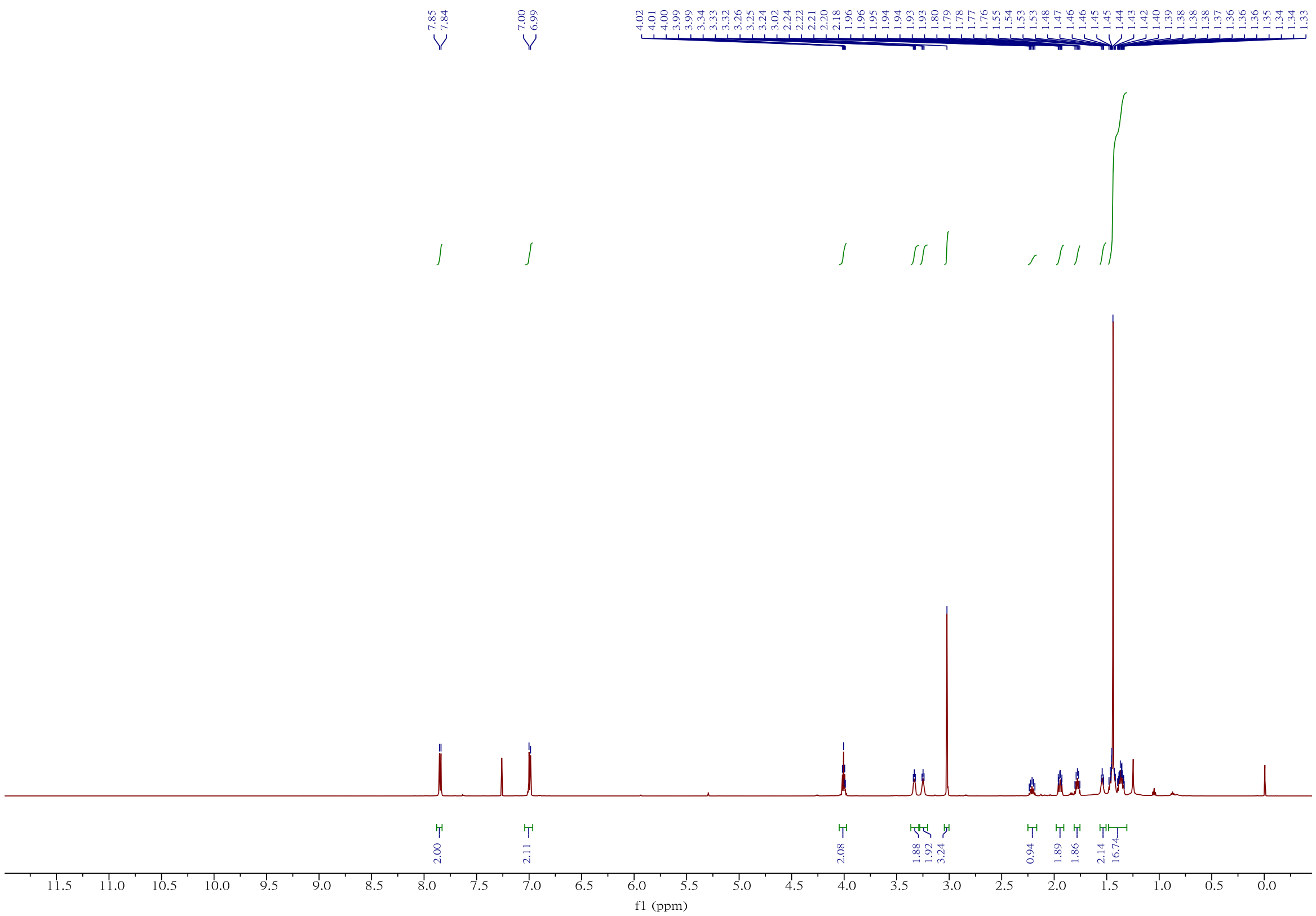

Supplementary Figure 224. ^{1}H NMR (600 MHz) of Compound (82)

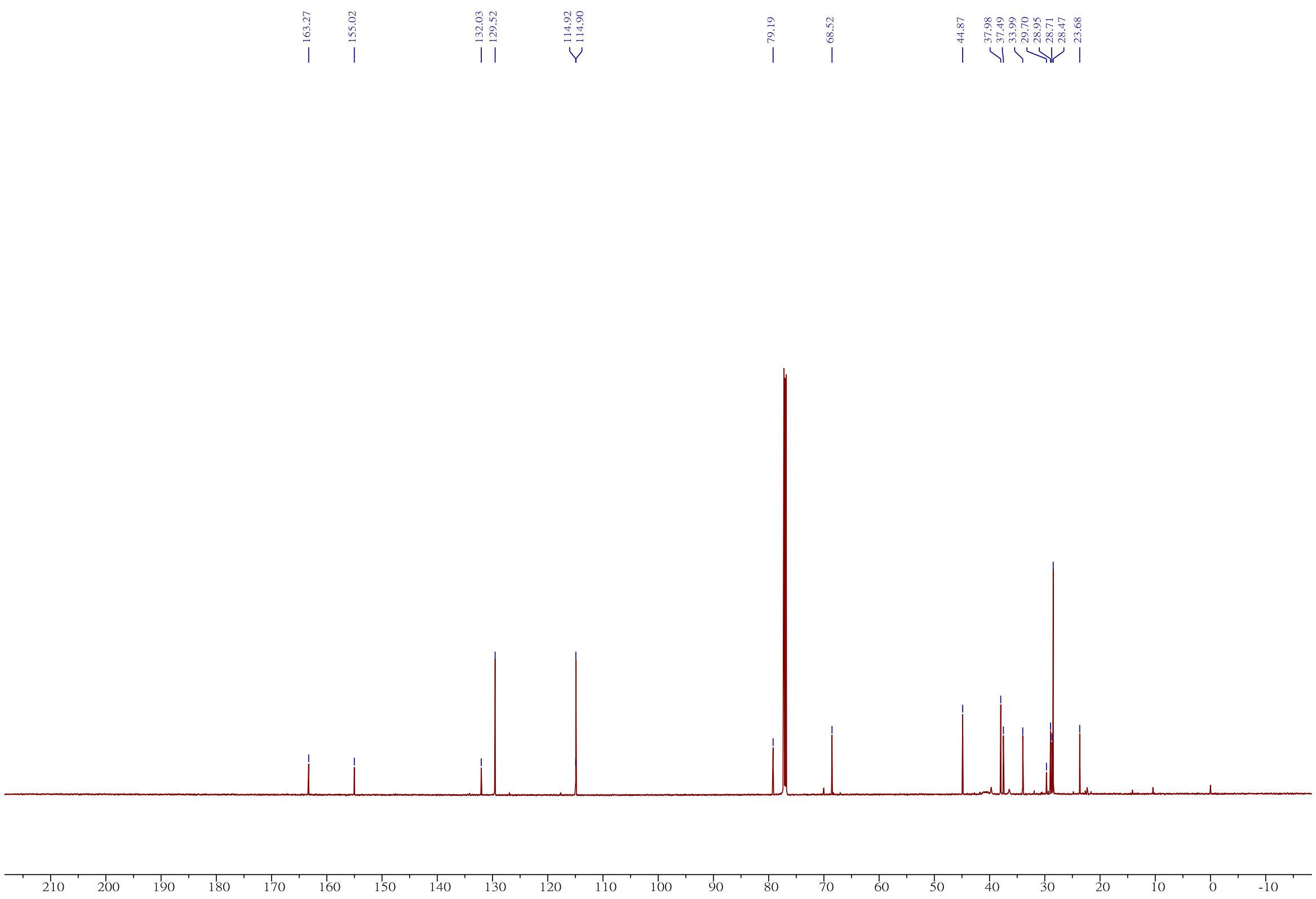

Supplementary Figure 225. ^{13}C NMR (151 MHz) of Compound (82)

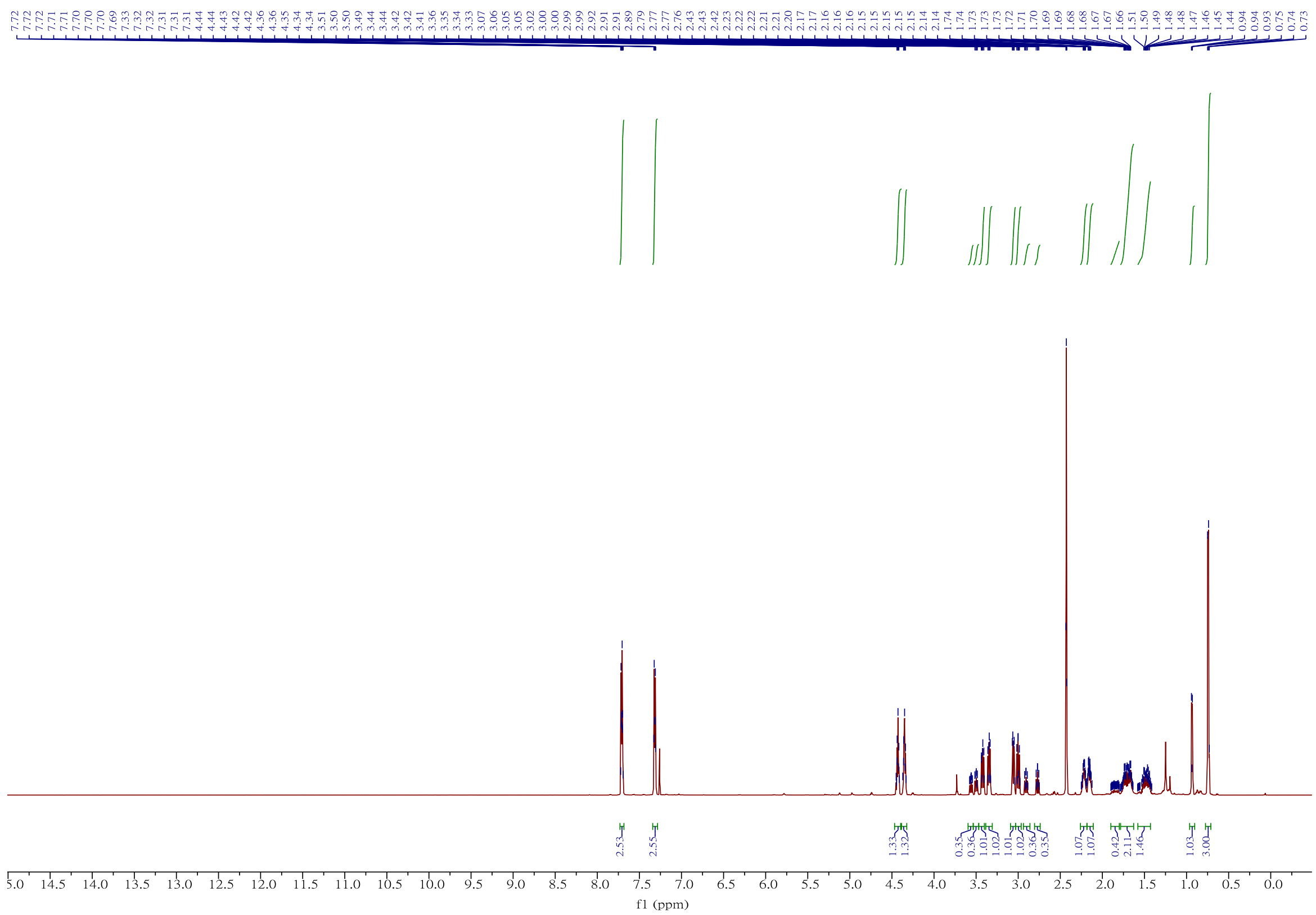

Supplementary Figure 226. ^{1}H NMR (600 MHz) of Compound (84)

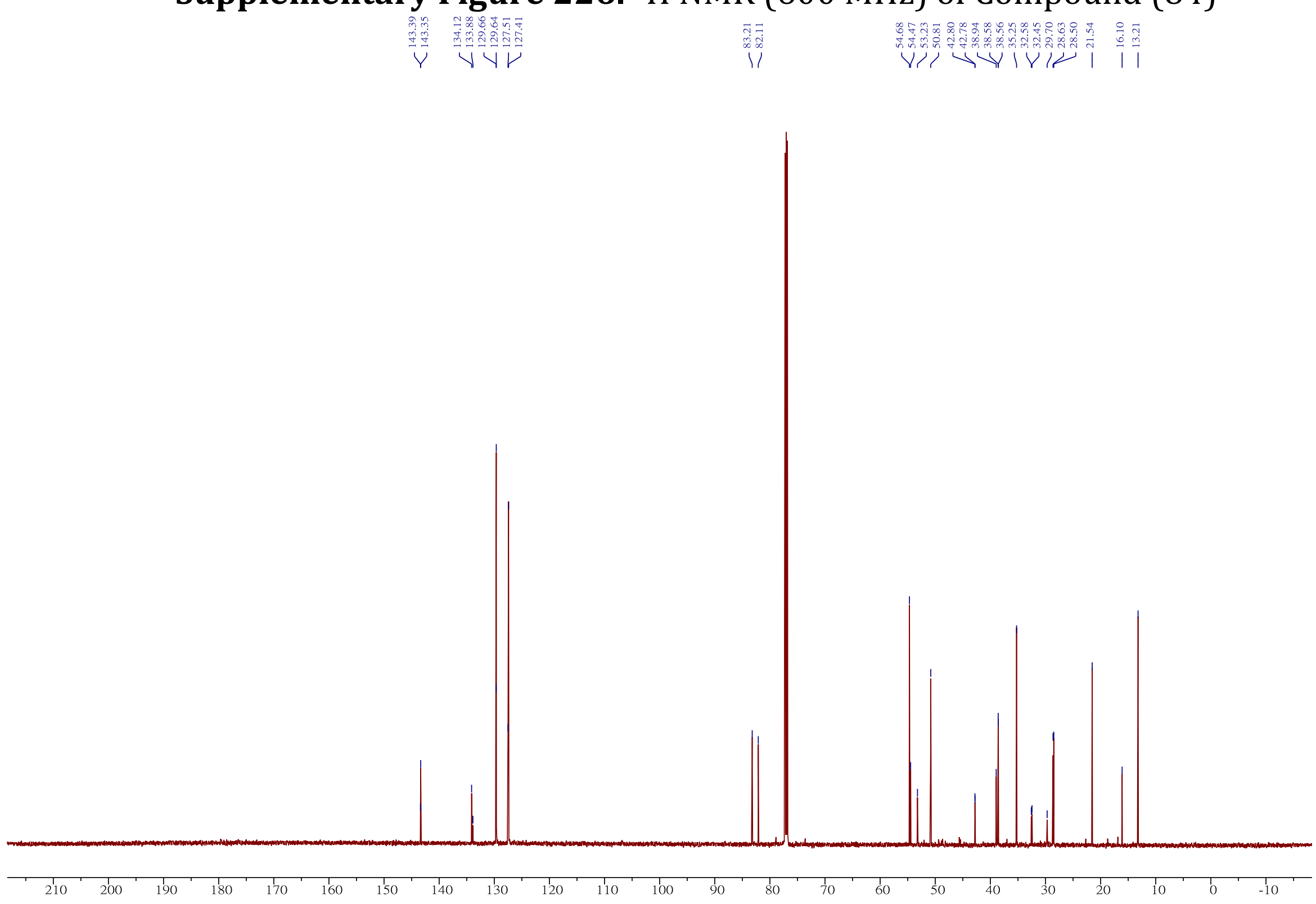

Supplementary Figure 227. ^{13}C NMR (151 MHz) of Compound (84)

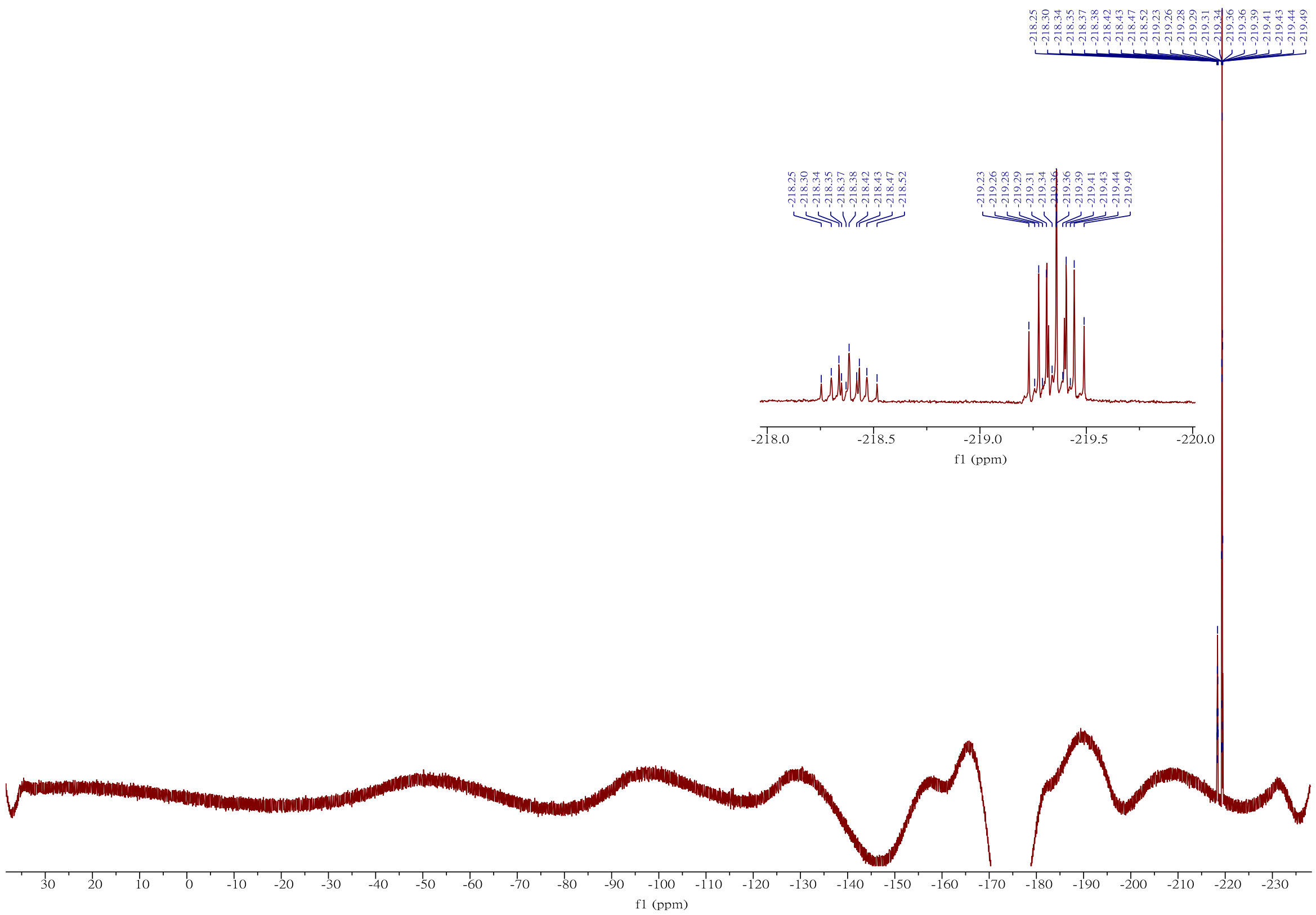

Supplementary Figure 228. ^{19}F NMR (564 MHz) of Compound (84)

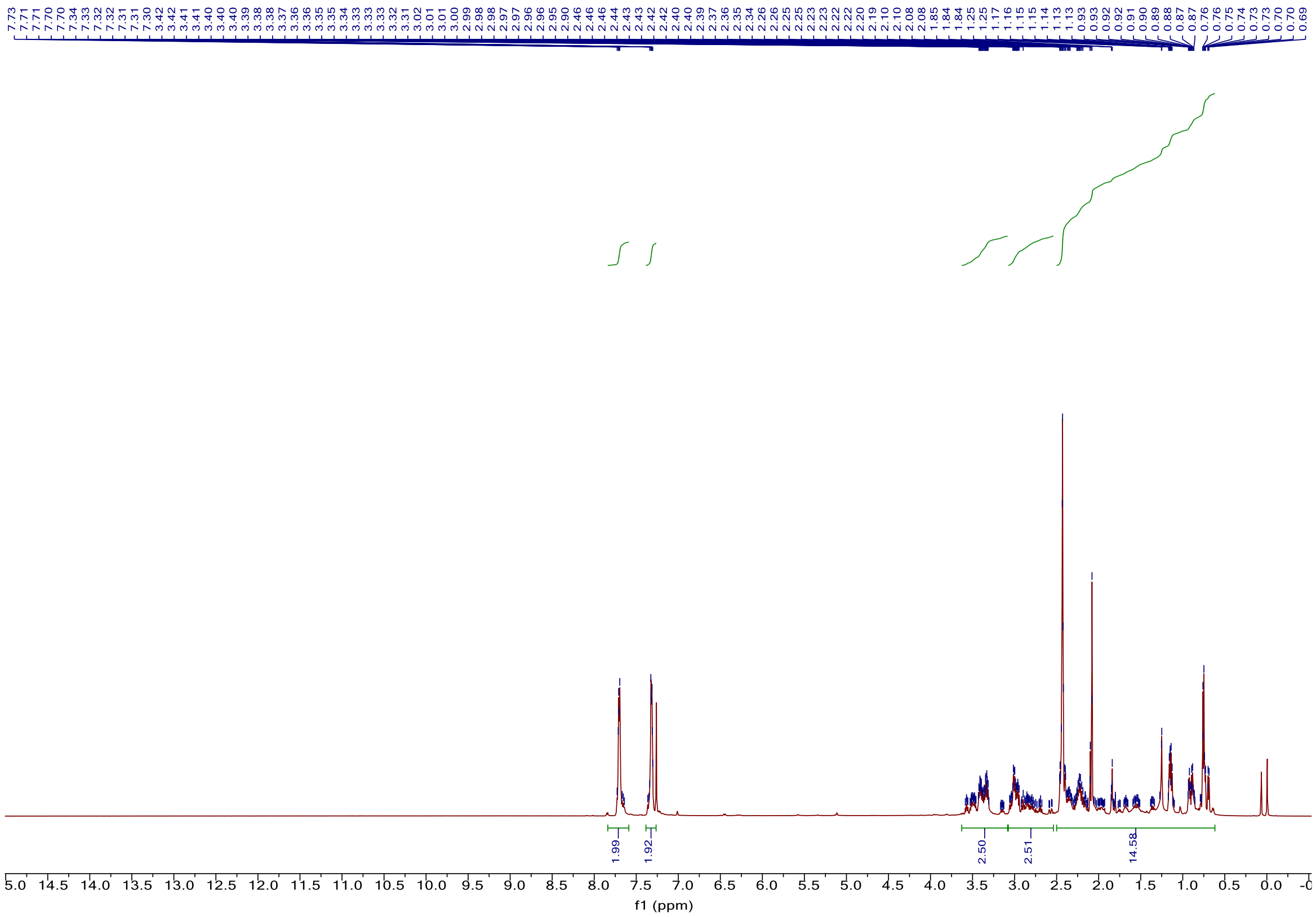

Supplementary Figure 229. ^{1}H NMR (600 MHz) of Compound (85)

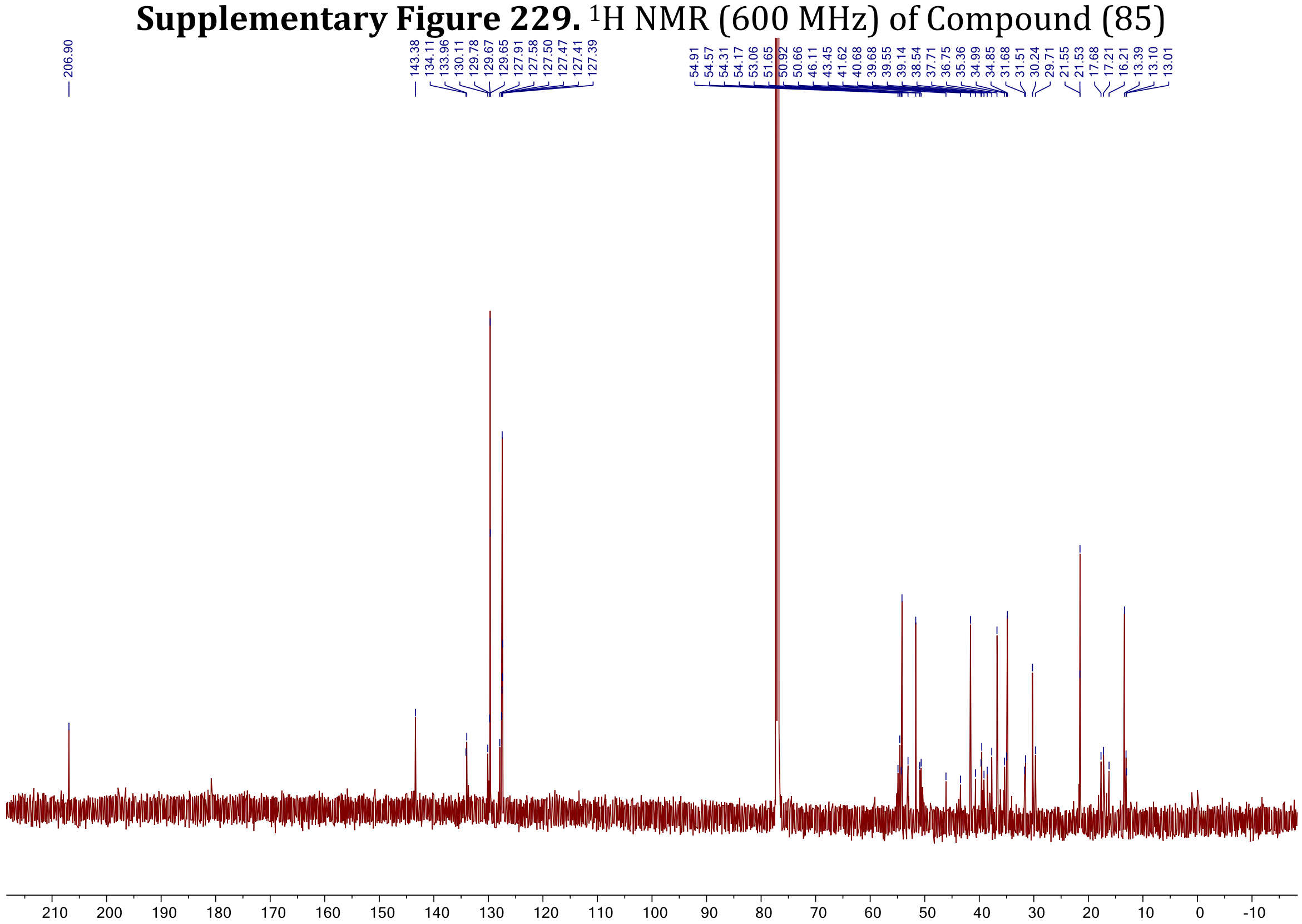

Supplementary Figure 230. ^{13}C NMR (151 MHz) of Compound (85)

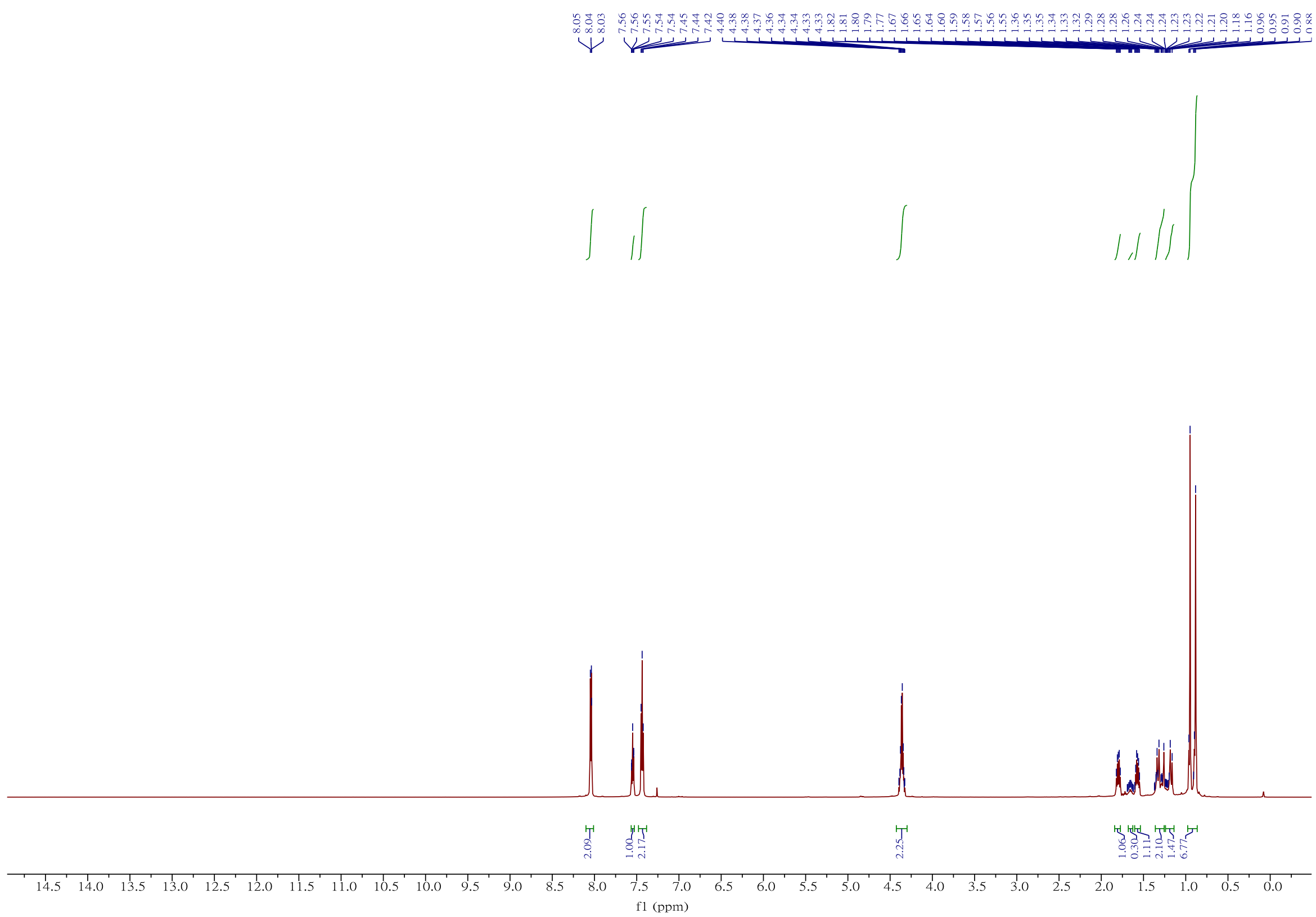

Supplementary Figure 231. ^{1}H NMR (600 MHz) of Compound (86)

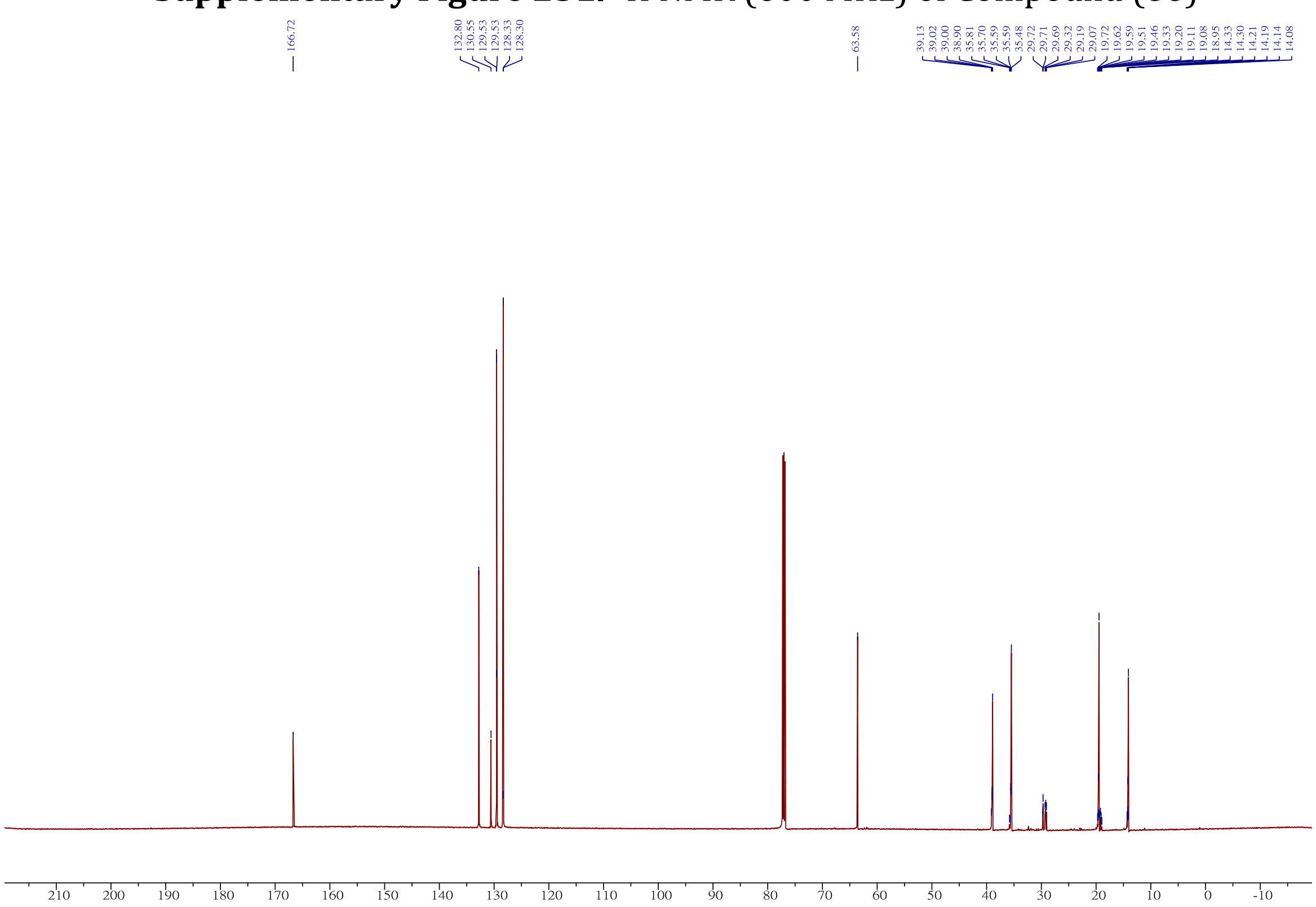

Supplementary Figure 232. ^{13}C NMR (151 MHz) of Compound (86)

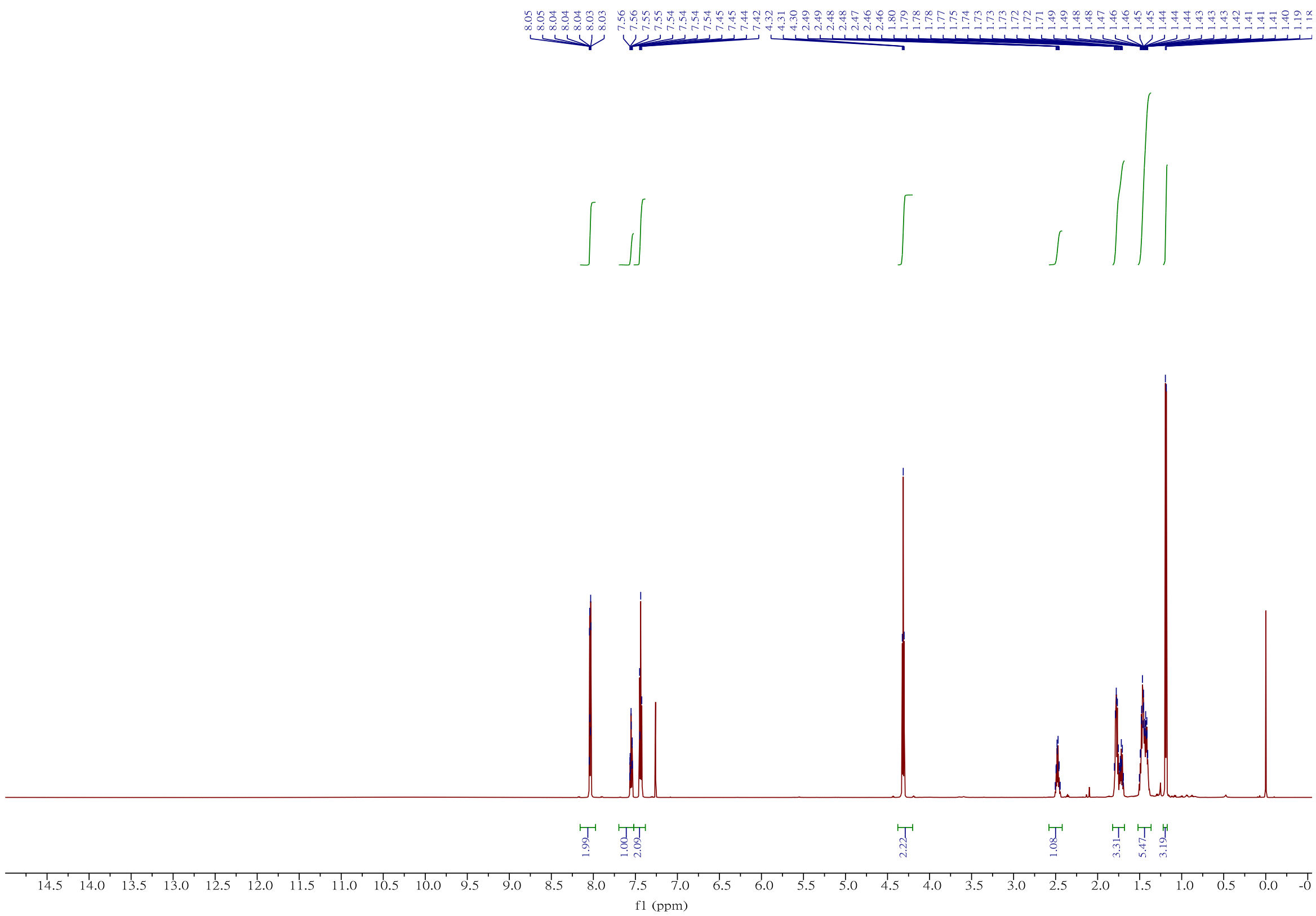

Supplementary Figure 233. ^{1}H NMR (600 MHz) of Compound (2’)

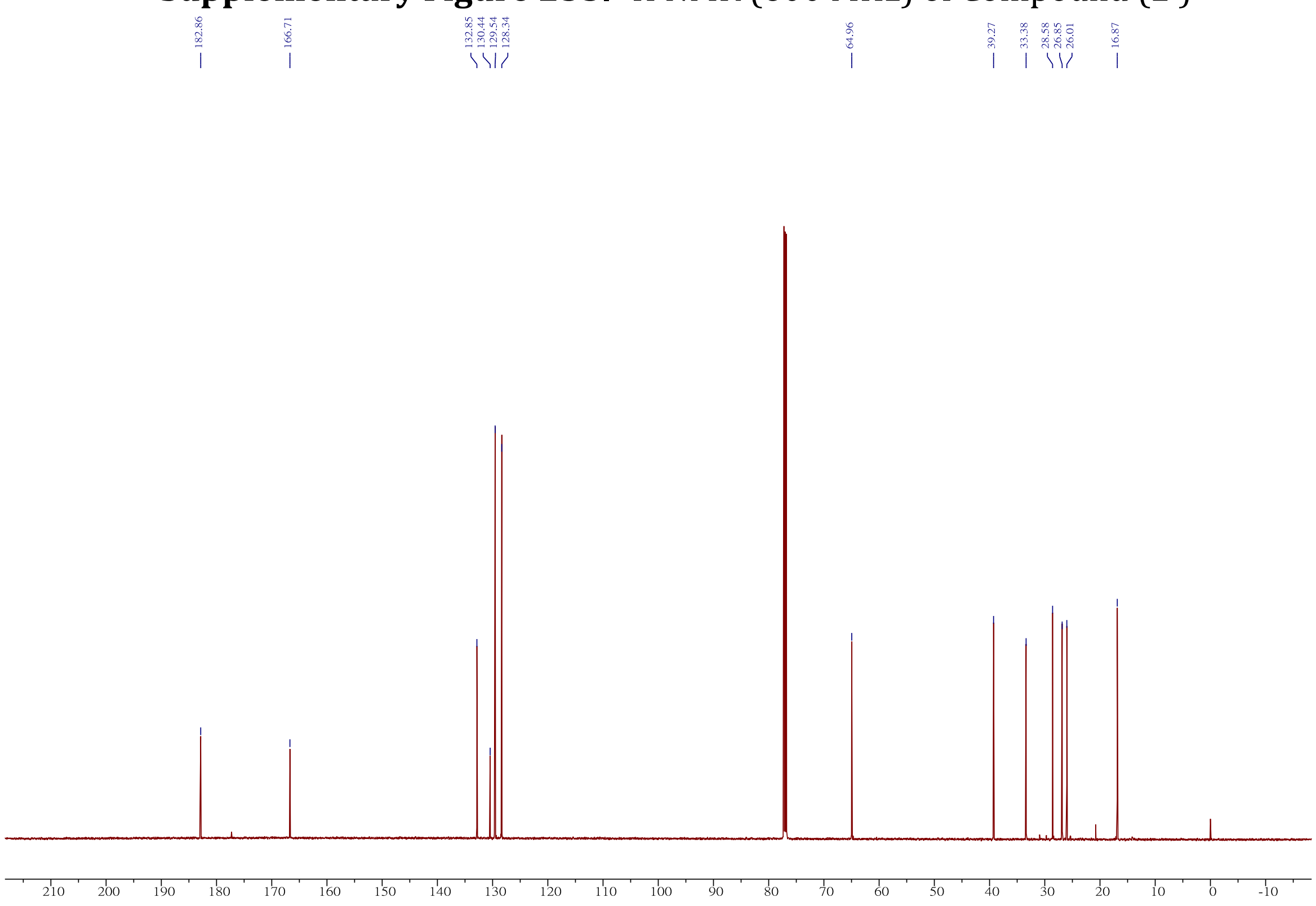

Supplementary Figure 234. ^{13}C NMR (151 MHz) of Compound (2’)

Supplementary References

1. Liu, J., Lu, C., Wu, S., Wu, X., Wu, Z., Xue, X.-S., Yu, J., Zhu, C. Polarity Umpolung Strategy for the Radical Alkylation of Alkenes. *Angew. Chem. Int. Ed. Engl.* **59**, 8195 (2020).
2. Lu, Y. C., West, J. G. Chemoselective Decarboxylative Protonation Enabled by Cooperative Earth-Abundant Element Catalysis. *Angew. Chem. Int. Ed. Engl.* **62**, e202213055 (2023).
3. Nikitas, N. F., Voutyritsa, E., Gkizis, P. L., Kokotos, C. G. Metal-free Photochemical Atom Transfer Radical Addition (ATRA) of $BrCCl_3$ to Alkenes. *Eur. J. Org. Chem.* **2021**, 96 (2021).
4. Liu, Y.-Y., Wang, H., Wang, C.-P., Wan, J.-P., Wen, C.-P. Bio-based green solvent mediated disulfide synthesis via thiol couplings free of catalyst and additive. *RSC Adv.* **3**, 21369-21372 (2013).
5. Ji, Y., DiRocco, D. A., Hong, C. M., Wismer, M. K., Reibarkh M. Facile Quantum Yield Determination via NMR Actinometry. *Org. Lett.* **20**, 8, 2156-2159 (2018).
6. Dekkiche, B. A., Seraghni, N., Debbache, N., Ghoul, I., Sehili, T. Effect of Natural and Artificial Light on Fe(III) Organic Complexes Photolysis: Case of Fe (III)-Malonate and Fe(III)-Malate. *Int. J. Chem. React. Eng.* **17**, 2, 20180106 (2019).
7. Dekkiche, B. A., Debbache, N., Ghoul, I., Seraghni, N., Sehili, T., Marín, Z., Santaballa, J.A., Canle, M. Evidence of non-photo-Fenton degradation of ibuprofen upon UVA irradiation in the presence of Fe (III)/malonate *J. Photochem. Photobiol. A Chem.* **382**, 111976 (2019).

8. Bhuyan, S., Gogoi, A., Basumatary, J., Gopal Roy, B. Visible-Light-Promoted Metal-Free Photocatalytic Direct Aromatic C−H Oxygenation. *Eur. J. Org. Chem.* **2022**, e202200148 (2022).
